# Differential DNA Methylation of MicroRNA Genes in Temporal Cortex from Alzheimer's Disease Individuals

**DOI:** 10.1155/2016/2584940

**Published:** 2016-04-26

**Authors:** Darine Villela, Rodrigo F. Ramalho, Aderbal R. T. Silva, Helena Brentani, Claudia K. Suemoto, Carlos Augusto Pasqualucci, Lea T. Grinberg, Ana C. V. Krepischi, Carla Rosenberg

**Affiliations:** ^1^Department of Genetics and Evolutionary Biology, Institute of Biosciences, University of São Paulo, Rua do Matão 277, 05508-090 São Paulo, SP, Brazil; ^2^International Research Center, CIPE, AC Camargo Hospital, Rua Taguá 440, 01508-010 São Paulo, SP, Brazil; ^3^Institute and Department of Psychiatry, University of São Paulo Medical School, Avenida Doutor Arnaldo 455, 01246-000 São Paulo, SP, Brazil; ^4^Discipline of Geriatrics, Department of Internal Medicine, University of São Paulo Medical School, Avenida Doutor Arnaldo 455, 01246-000 São Paulo, SP, Brazil; ^5^Brazilian Aging Brain Study Group, LIM22, Department of Pathology, University of São Paulo Medical School, Avenida Doutor Arnaldo 455, 01246-000 São Paulo, SP, Brazil; ^6^Department of Pathology, University of São Paulo Medical School, Avenida Doutor Arnaldo 455, 01246-000 São Paulo, SP, Brazil; ^7^Memory and Aging Center, Department of Neurology, University of California, 675 Nelson Rising Lane, P.O. Box 1207, San Francisco, CA 94143, USA

## Abstract

This study investigated for the first time the genomewide DNA methylation changes of noncoding RNA genes in the temporal cortex samples from individuals with Alzheimer's disease (AD). The methylome of 10 AD individuals and 10 age-matched controls were obtained using Illumina 450 K methylation array. A total of 2,095 among the 15,258 interrogated noncoding RNA CpG sites presented differential methylation, 161 of which were associated with miRNA genes. In particular, 10 miRNA CpG sites that were found to be hypermethylated in AD compared to control brains represent transcripts that have been previously associated with the disease. This miRNA set is predicted to target 33 coding genes from the neuregulin receptor complex (ErbB) signaling pathway, which is required for the neurons myelination process. For 6 of these miRNA genes (*MIR9-1*,* MIR9-3*,* MIR181C*,* MIR124-1*,* MIR146B*, and* MIR451*), the hypermethylation pattern is in agreement with previous results from literature that shows downregulation of miR-9, miR-181c, miR-124, miR-146b, and miR-451 in the AD brain. Our data implicate dysregulation of miRNA methylation as contributor to the pathogenesis of AD.

## 1. Introduction 

Increasing evidence indicates that epigenetic mechanisms may contribute to the pathogenesis of Alzheimer's disease (AD) [1, 2]. DNA methylation, the addition of methyl group to the 5′-position cytosine of CpG dinucleotides, is an important epigenetic mark involved in the control of gene expression. Usually, when located in the promoter region, DNA methylation is associated with gene silencing, either inhibiting binding of transcription factors, or through the recruitment of methyl-binding proteins and their associated chromatin remodeling factors [3]. It is estimated that ~70% of the CpGs are methylated in the human genome; however, the distribution of CpGs is not uniform and certain regions are referred as CpG islands, due to a higher than expected frequency of CpG dinucleotides [4]. In contrast to the general pattern, CpG islands are normally unmethylated or present low levels of methylation. Although DNA methylation was previously thought to be a static process after cellular differentiation, now it is known to be highly dynamic not only in early developmental phase, but also in adult human brain in response to neural activity [5].

Most recently, genomewide DNA methylation changes have been reported in AD brains [6]. Jager et al. demonstrated in AD prefrontal cortex that a subset of 71 CpG sites was differentially methylated and transcriptionally altered, affecting genes connected to a well-known AD susceptibility network. Also, the authors pointed out that these epigenetic changes might have occurred early in the pathological process, possibly contributing to extracellular *β* amyloid plaques accumulation. Although most AD methylation studies focus on protein coding genes, dysregulation of noncoding RNAs has become increasingly an important field of investigation.

MicroRNAs (miRNAs), a class of small noncoding RNAs that act as posttranscriptional regulators of gene expression, are the best characterized and most extensively studied class of noncoding RNAs [7]. Data from literature have shown that the expression of miRNAs is widely altered in AD brains [8]. Abnormal expression profile of a specific group of miRNAs in AD brains (let-7i, miR-9, miR-15, miR-146b, miR-181c, miR-210, miR-338, and miR-451) has been reported by different studies, but the mechanisms underlying such dysregulation still remain poorly understood [9, 10]. However, it is known that transcription silencing due to promoter CpG island hypermethylation is one of the most common mechanisms by which several tumor suppressor and miRNA genes are inactivated during tumorigenesis [11]. Considering that cancer and neurodegenerative disorders share common mechanisms of genetic and molecular alterations, the involvement of DNA methylation in the dysregulation of miRNA expression in AD brains is a reasonable supposition.

Here, we analyzed genomewide DNA methylation changes of noncoding RNAs, with particular interest on miRNAs, in postmortem temporal cortex samples from individuals with AD.

## 2. Casuistic and Methods

### 2.1. Postmortem Human Brain Samples

Postmortem human temporal cortex samples from 10 AD individuals with severe stage of the disease and their 10 age-matched controls were provided by the Brain Bank of the Brazilian Aging Brain Study Group (BBBABSG) [12].  Table 1 presents the characterization of the individuals included in this study. An independent sample set with the same clinical-pathological profile that the cohort used for methylation study (9 AD individuals and 10 controls, Supplementary Table 1 available online at http://dx.doi.org/10.1155/2016/2584940) was used for gene expression analysis. As a standard protocol for AD neuropathological diagnosis, the brain was examined macroscopically and 15 neurodegenerative disease-related structures were sampled for microscopic evaluation. Neuropathological examinations were carried out using immunohistochemistry following internationally accepted guidelines. CERAD (Consortium to Establish a Registry for Alzheimer's Disease) criteria were used to classify the *β*-amyloid neuritic plaque burden and the distribution of neurofibrillary tangles was classified according to the H. Braak and E. Braak staging system [13]. The neuropathological diagnosis of AD was made in those cases that showed at least Braak stage III and CERAD moderate. The usual neuropathological guidelines were used for other dementias and for Parkinson's disease [12]. The subject's clinical and functional statuses were assessed through a knowledgeable informant based on a validated clinical protocol that also includes cognitive evaluation by the Clinical Dementia Rating Scale (CDR) [14]. This protocol included a series of semistructured scales and questionnaires that cover major functional abilities and were validated for assessment with an informant. BBBABSG's procedures were approved by the ethical board of University of São Paulo Medical School and by the Brazilian Federal Health Department, which are based on international standards such as The Belmont Report [15] and The Helsinki Declaration [16]; also an informed written consent was obtained by the next-of kin [12]. Importantly, based on the informed consent provided by the BBBABSG's, there is no information whether the individuals were treated with drugs that could influence DNA methylation patterns.

### 2.2. Genomewide DNA Methylation Analysis

Genomic DNA was isolated from each postmortem temporal cortex sample of AD and control individuals using a standard phenol-chloroform extraction method. Next, bisulfite conversion of 500 ng of each DNA sample was performed using EZ DNA methylation kit according to the manufacturer's instructions (Zymo Research, Irvine, California, USA). Four *μ*L of bisulfite-converted DNA was used for hybridization on Infinium Human Methylation 450 K BeadChip, following the Illumina Infinium HD Methylation Protocol (Illumina, San Diego, California, USA). Analysis of differential methylation was conducted using the RnBeads package [17]. Among the 485,577 probes present in 450 K, we selected only those associated with noncoding RNA genes according to UCSC Genome Browser classification, resulting in a total of 15,258 CpG sites that were processed. DNA methylation of genomic regions includes promoter (TSS1500, TSS200, 1st Exon and 5′UTR), body, 3′UTR, and intergenic regions as well as CpG islands, shores (2 kb regions upstream or downstream of the CpG islands), shelves (2 kb regions upstream or downstream of the CpG island shores), and “open sea” (regions outside the CpG island context). Methylation score for each CpG site is represented as a beta value, which ranges from 0 (no methylated) to 1 (completed methylated). We removed one control sample from our analysis because it presented a low call rate. Normalization and probe type bias adjustment were applied using BMIQ method [18]. We removed probes that (1) lacked beta values, (2) targeted single-nucleotide polymorphisms (SNPs) in the last three bases, (3) mapped at sexual chromosomes, (4) had detection *p* values >0.05, or (5) overlapped repetitive sequences. As a result, 14,468 noncoding RNA probes fulfilled our selection criteria. Differential methylation *p* value for each probe was obtained from Welch* t*-test or, alternatively, from linear models employed in the limma package after conversion of beta values into *M*-values (*M* = log⁡2(Beta/1 − Beta)) [17]. Each *p* value was then corrected for multiple tests by using the method of Benjamini and Hochberg [19]. The adjusted *p* values resulted in no differentially methylated CpG sites, but Welch* t*-test showed significance, revealing that the average of methylation from CpG sites differs between the two compared groups. DNA methylation differences between groups were tested for significance using Mann-Whitney test. All statistical analyses were performed using GraphPad PRISM software v6.0 (GraphPad Software Inc., La Jolla, California, USA). Beta values are presented as mean ± standard error of the mean (SEM). Delta beta is referred to as the average of DNA methylation difference between the two compared groups.

### 2.3. Gene Expression Analysis

Total RNA was isolated using the RNeasy Mini kit (Qiagen, Hilden, Germany) according to the manufacturer's instructions. cDNA amplification, labeling, and hybridization were performed as previously described [20]. RNA expression experiments were performed using a customized cDNA platform containing 4,608 ORESTES representing human genes [21]. Hybridized arrays were scanned on the ScanArrayTM Express (Packard BioScience Biochip Technologies, Billerica, MA, USA) and Cy5/Cy3 signals were quantified using the histogram method, which is part of the ScanArray Express software (Perkin-Elmer Life Sciences, Boston, MA, USA). After normalization, data for each gene was reported as the logarithm of the expression ratio. Raw data was deposited at the Gene Expression Omnibus under accession number GSE13214. For analysis of differentially expressed genes, AD individuals were compared to controls by performing a Student's *t*-test (*p* ≤ 0.05) with 1000 permutations using MEV (MultiExperiment Viewer, Boston, MA, USA) software [22].

## 3. Results

**Figure 1:**
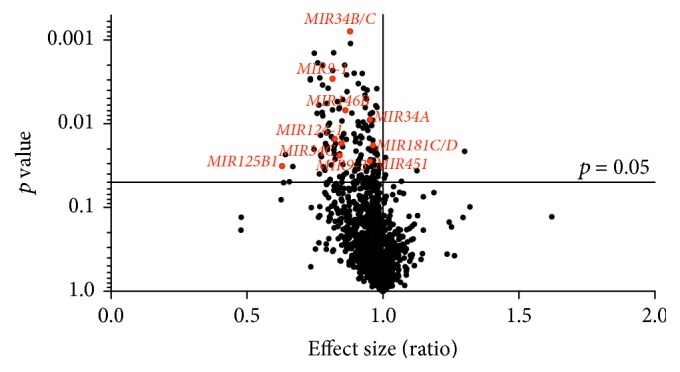
Volcano plot of methylation differences of all the CpG sites associated with microRNA genes. In total, 161 CpG sites were found to be differentially methylated (*p* < 0.05) in Alzheimer's disease (AD) group compared to controls, most of them hypermethylated; the 10 miRNA CpG sites highlighted in red map to transcripts previously reported as dysregulated in AD brains.

**Figure 2:**
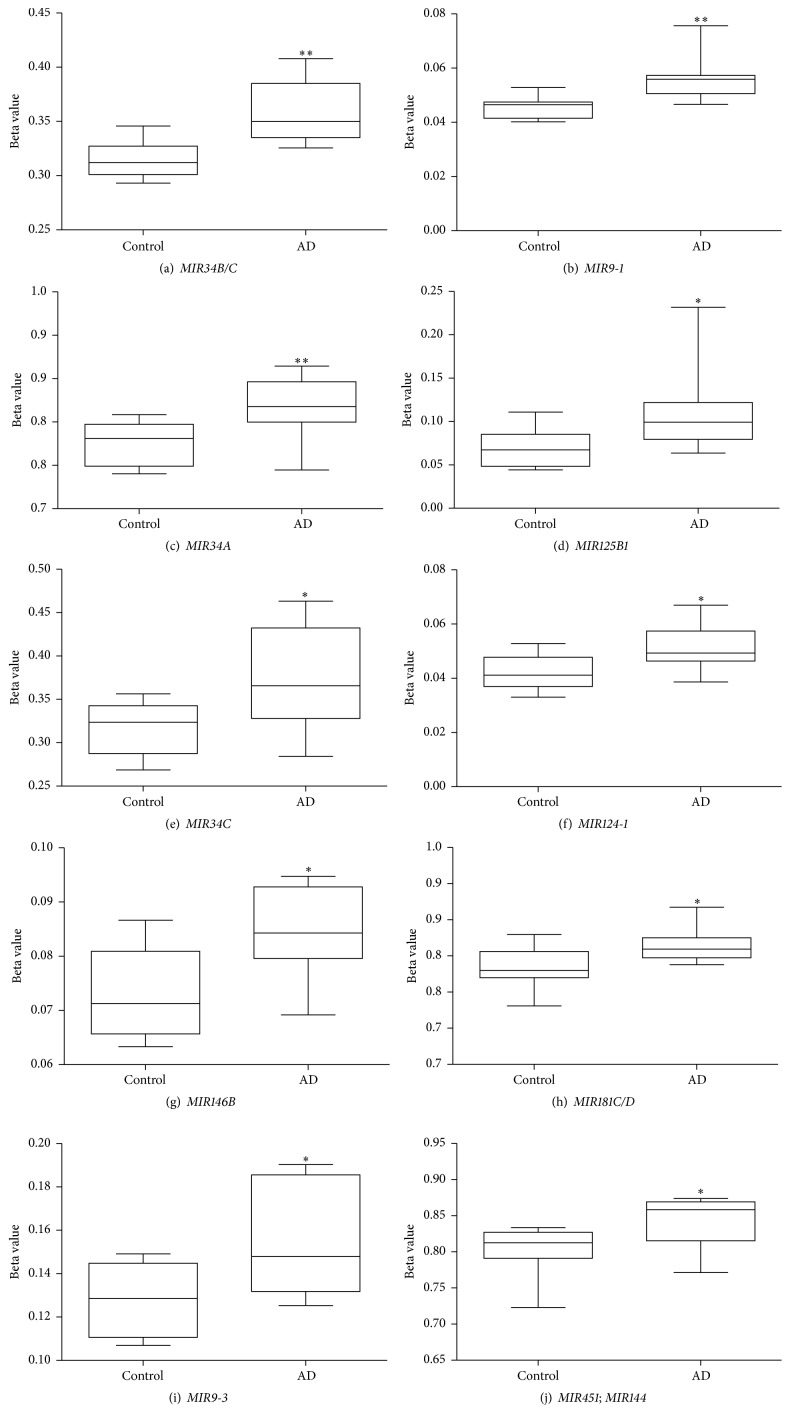
Box plots of DNA methylation level of microRNA genes found to be hypermethylated in Alzheimer's disease brains. The 10 hypermethylated CpG sites encompassing microRNA genes are presented (a–j). Mann-Whitney test, ^*∗∗*^ *p* < 0.01, ^*∗*^ *p* < 0.05. Beta values are displayed as means ± SEM.

**Figure 3:**
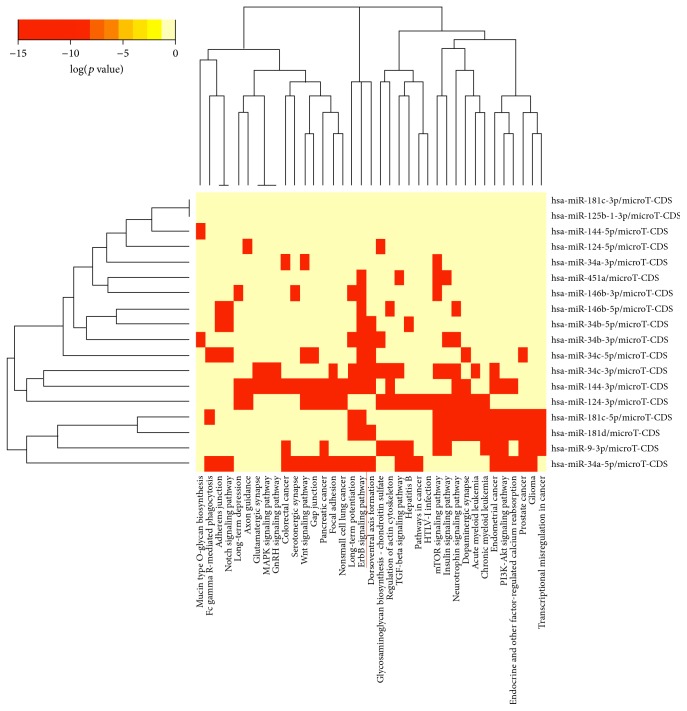
Heat map of hypermethylated microRNAs versus predicted target pathways. The figure illustrates hierarchical clustering results for miRNAs (*y*-axis) and pathways (*x*-axis). The neuregulin receptor complex (ErbB) signaling pathway is clearly targeted by most investigated miRNAs. Image derived from DNA Intelligent Analysis- (DIANA-) miRPath using microT-CDS as the target prediction algorithm.

A total of 2,095 out of 14,468 noncoding RNA CpG sites presented differential methylation *p* value (*p* < 0.05; Welch's* t*-test, Supplementary Table 2) in the temporal cortex samples of AD individuals compared to controls. Among them, 161 were associated with miRNA genes (*p* < 0.05; Welch's* t*-test, Supplementary Table 3).  Figure 1 shows DNA methylation differences of the probes associated with miRNA genes and reveals that the large majority of the 161 differentially methylated miRNA CpG sites found in our casuistic were hypermethylated in the AD group. Notably, 10 CpG sites, highlighted in Figure 1, overlapped miRNA genes whose transcripts had been previously reported as dysregulated in AD brains (*MIR34B/C, MIR9-1, MIR34A, MIR125B1, MIR146B, MIR124-1, MIR181C/D, MIR451,* and* MIR9-3*).  Table 2 presents the methylation status of these 10 CpG sites (all hypermethylated) and the statistical data comparing the AD group with controls. The average of methylation differences (delta beta) between the compared groups range from 0.9 to 5.6%. Except for* MIR125B1*, all miRNA genes presented methylation differences in probes mapped in promoter regions.* MIR9-1*,* MIR9-3*, and* MIR124-1* showed differential methylation in CpG islands within the promoters, while the other miRNA genes presented differential methylation either in shores, shelves, or outside the CpG island context “open sea”.  Figure 2 shows the box plot of differential methylation of all miRNA genes presented in Table 2. Using the DNA Intelligent Analysis- (DIANA-) miRPath v2.0 (http://www.microrna.gr/miRPathv2) [23], we retrieved the predicted target pathways of this 10 miRNA and found enrichment for the neuregulin receptor complex (ErbB) signaling pathway (Figure 3); 33 coding genes involved in this pathway are predicted targets of most of the investigated miRNA sets (Supplementary Table 4). To explore the expression of the predicted coding genes targeted by these 10 hypermethylated miRNA, a ~5 k microarray platform was used. Out of the 33 predicted genes, 10 were represented in this platform, three of which were overexpressed in AD individuals compared to controls (Table 3).

## 4. Discussion

So far, approximately 30 miRNAs have been implicated in the AD pathogenesis for presenting altered expression [8–10]; however, the mechanisms underlying such dysregulation remain unknown, neither miRNAs silenced by DNA methylation nor genomewide methylation changes of noncoding RNA genes have been described in AD brains. The present study is the first genomewide methylation analysis of noncoding RNA genes in AD brains. Among the 161 differentially methylated miRNA CpGs, we identified 10 miRNAs genes, all of them harboring hypermethylated CpG sites, whose transcripts are among those previously reported as dysregulated in AD brains. Interestingly, the hypermethylation in the promoter region of 6 of these miRNA genes (*MIR9-1*,* MIR9-3*,* MIR181C, MIR124-1, MIR146B,* and* MIR451*) is in agreement with previous data from literature that shows downregulation in AD brains of their corresponding transcripts (miR-9, miR-181c, miR-124, miR-146b, and miR-451).

It has been demonstrated that miR-9, which is encoded by 3 different genes (*MIR9-1*,* MIR9-2*, and* MIR9-3*), may be relevant in AD pathogenesis since it targets two of the most important proteins involved in the disease pathology: amyloid-*β* precursor protein (A*β*PP), which carries the amyloid-*β* peptide (A*β*) that precipitates in the amyloid plaques, and BACE1 that cleaves APP to originate A*β* [24]. Also, miR-9 is differentially expressed in cerebral regions that are significantly associated with disease progression [25, 26]. Downregulation of miR-9 may increase both APP and BACE1 in AD brains suggesting a posttranscriptional regulation of these two proteins under pathological conditions [27]. As observed with miR-9, downregulation of miR-181c in AD brains also correlates with increase of amyloid-*β* peptide levels [27–29]. Moreover, miR-181 is known to act as an additional repressor of SIRT1 expression, which via its histone deacetylase activity provides a feedback to the level of epigenetic control [28]; Donmez and colleagues revealed that overexpression of SIRT1 prevents A*β* production by deacetylating the retinoic acid receptor *β*, promoting nonamyloidogenic cleavage of APP through an upregulation of ADAM10, a major component of *α*-secretase [30]. It is worth to mention though that downregulation of miR-9 and miR-181c is not restricted to AD, as these miRNAs are also diminished in others neurodegenerative disorders [10]. It is reasonable to assume that aberrant profiles of neural miRNA expression shared by several neurodegenerative disorders may give rise to common patterns of cellular dysfunction in these conditions.

miR-124 has been found to be the most abundant microRNA expressed in the adult human brain. Three genes (*MIR124-1*,* MIR124-2*, and* MIR124-3*) at 3 independent loci in the human genome encode the same mature miR-124 [31]. An investigation using cellular AD models and cultured hippocampal neurons revealed that dysregulation of miR-124 is associated with AD pathology via its target, BACE1 [32]. The authors demonstrated that suppression or overexpression of miR-124 causes respectively an up and downregulation of BACE1 expression, and is well correlated with cell death induced by A*β* neurotoxicity. Ultimately, miR-146b and miR-451 are reported to be downregulated in cerebrospinal fluid from patients with AD, and both miRNAs are involved in multiple inflammatory pathways [27]. Notably, miR-146b is described to be consistently downregulated at different areas within AD brains [27]. Analysis of miR-146b expression in human monocytes revealed that its activation occurs in response to a variety of microbial components and proinflammatory cytokines [33]; it controls toll-like receptor (TLR) and cytokine signaling through a negative feedback loop that involves downregulation of IL-1 receptor-associated kinase 1 and TNF receptor-associated factor 6 protein levels [34]. It has been proposed that downregulation of miR-146b in AD brains releases translational repression of TLR signaling and exacerbates the innate immunity response, which in turn contributes to neurodegeneration [9, 27]. In fact, studies have demonstrated that A*β* can activate TLR signaling. This TLR signaling activation may lead to an increase of innate immune response from the brain due to microglia recruitment; therefore, downregulation of miR-146b on AD brain also lends support to the inflammation hypothesis of AD pathogenesis [9].

The hypermethylated miRNA set is predicted to target, among others, 33 coding genes from the neuregulin receptor complex (ErbB) signaling pathway, which is required for the neurons myelination process [35, 36]. Three genes of the (ErbB) signaling pathway (*MYC*,* MAP2K1*, and* ABL2*) were found to be overexpressed in AD brains, as expected for targets of hypermethylated miRNAs. The magnitude of gene expression changes observed was modest, but studies have demonstrated that miRNAs regulate gene expression in a subtle manner and the magnitude of repression is rarely higher than 2-fold [37].* MYC* and* MAP2K1* are known to be involved in many cellular processes including cell cycle progression, apoptosis, and differentiation, transcription regulation and* ABL2* plays a role in cytoskeletal rearrangements through its microtubule-binding sequences. Interestingly, all of them were already shown to be overexpressed in AD brain [38–40].* MYC*, which presented the greatest fold change expression in our casuistic is reported to be overexpressed in degenerating neurons, and its expression in transgenic mice induces neuronal-specific cell cycle re-entry, neurodegeneration, and, importantly, significant cognitive deficits [39].

The other differentially hypermethylated miRNA genes (*MIR34B/C*,* MIR125B1*, and* MIR181D*) found in our casuistic cannot be directly related to the already described upregulation pattern of expression of these transcripts in AD brains [9, 10]; thus further investigation is required to clarify the role of DNA methylation in the transcriptional regulation of these miRNA species. Of note, even though our work has focused mainly on miRNA genes, we also investigated whether some of the long noncoding RNAs already described as dysregulated in AD brains presented altered methylation (reviewed by [41]). No long noncoding RNA genes pointed out by the authors were found to be differentially methylated.

Differences in miRNA expression between male and female mice brains have been reported mainly associated with gonadal steroid regulation and sex chromosome bias [42]. In the present study, sex chromosome probes were removed and the analytical procedure used accounts for eventual bias introduced by gender, batch, and so forth [17]. However, albeit remote, we cannot exclude the possibility that the uneven number of male/females may have had some influence in the results. Even though the modest differences in methylation observed in our study are in agreement with previous genomewide and candidate gene reports that describe methylation differences below 10% in AD brains [6, 43, 44], the evidence presented here should be validated in larger cohorts. But together these findings support the involvement of DNA methylation in the disease, but the individual effects at each CpG site indeed seems to be subtle. Heterogeneity of population cells in the brain is one aspect that could contribute to make methylation changes less apparent. The human brain being composed by many different cell types, each one having distinct roles on AD progression, certainly adds considerable complexity into methylation analysis. AD brains have an active changing cell population including neuronal loss and glial activation that may in part be responsible for the observed results. However, due to technological limitations it is still not possible to distinguish cell type specific methylation changes. Additionally, likewise many other epigenomic studies in human brains, determining whether epigenomic changes are cause or consequence of the disease is a difficult challenge. Unless epigenomic studies are performed in a noninvasive manner or using small tissue biopsies, it will remain especially arduous to establish causality for neurodegenerative disorders.

## 5. Conclusion

In summary, our results suggest that DNA methylation is likely involved in the dysregulation of miRNA expression in the human brain and may therefore contribute to impact the pathogenesis of AD.

## Supplementary Material

Supplementary Table 1 describes the clinical and pathological data of individuals used in gene expression analysis. Supplementary Table 2 presents 2,095 differentially methylated noncoding RNA CpG sites in the temporal cortex of AD subjects compared to controls. Supplementary Table 3 presents 161 differentially methylated CpG sites associated with miRNA genes. Supplementary Table 4 presents the predicted targets of the investigated miRNAs.
We added this paragraph in the Casuistic and Methods section of the manuscript.

## Figures and Tables

**Table 1:** Clinical data of control and Alzheimer's disease individuals investigated in the methylation study.

	Age at death (years)	Gender	CDR	Stages of senile changes			Cause of death
				Braak	CERAD	LB	
Controls							
1	81	F	0	0	0	—	Pulmonary edema
2	97	F	0	0	0	—	Pulmonary embolism
3	85	M	0	0	0	—	Acute myocardial infarction
4	83	M	0	0	0	—	Pulmonary edema
5	83	M	0	0	0	—	Pulmonary edema
6	81	F	0	0	0	—	Acute myocardial infarction
7	85	M	0	0	0	—	Pulmonary edema
8	83	M	0	0	0	—	Ischemic myocardial disease
9	77	F	0	0	0	—	Acute myocardial infarction
10	81	F	0	0	0	—	Acute myocardial infarction
Alzheimer's disease							
1	84	F	3	6	C	—	Pulmonary edema
2	82	M	3	6	C	—	Ischemic myocardial disease
3	80	M	3	5	C	—	Pulmonary edema
4	83	F	3	6	C	—	Pulmonary embolism
5	79	F	3	6	C	—	Pneumonia
6	86	F	3	5	C	—	Pneumonia
7	94	F	3	6	C	—	Pulmonary edema
8	83	F	3	6	C	—	Pulmonary embolism
9	80	F	3	5	C	—	Pulmonary edema
10	83	F	3	6	C	—	Pneumonia

Braak stage = neurofibrillary tangle; CERAD (Consortium to Establish a Registry for Alzheimer's Disease) = neuritic plaques; LB = Lewy body; CDR = Clinical Dementia Rate.

**Table 2:** MicroRNAs CpG sites differentially methylated in Alzheimer's disease.

CpG probe ID	Chr: Start site	Associated gene	UCSC_RefGene_Group	Relation_to_CpG_Island	Enhancer	Control	AD	Delta beta	*p* value
cg22806002	11: 111383083	*BTG4; MIR34C; MIR34B*	TSS200; TSS1500; TSS1500	N_Shore	—	0.314 ± 0.005	0.358 ± 0.008	0.044 (4.4%)	0.0015
cg13149127	1: 156390835	*C1orf61; MIR9-1*	5′UTR; TSS1500	Island	TRUE	0.045 ± 0.001	0.055 ± 0.002	0.010 (1%)	0.0015
cg09994773	1: 9212514	*MIR34A*	TSS1500	“open sea”	—	0.826 ± 0.008	0.867 ± 0.011	0.041 (4.1%)	0.0057
cg16865908	11: 121970496	*LOC399959; MIR125B1*	Body; Body	“open sea”	TRUE	0.068 ± 0.007	0.109 ± 0.015	0.040 (4.0%)	0.0101
cg25851152	10: 10419537	*MIR146B*	TSS1500	S_Shelf	TRUE	0.072 ± 0.002	0.084 ± 0.002	0.011 (1.1%)	0.0101
cg09148270	11: 111384223	*BTG4; C11orf88; MIR34C*	TSS1500; TSS1500; Body	N_Shore	—	0.315 ± 0.010	0.372 ± 0.018	0.056 (5.6%)	0.0172
cg14278808	8: 9761141	*LOC157627; MIR124-1*	TSS1500; TSS200	Island	—	0.042 ± 0.002	0.051 ± 0.002	0.009 (0.9%)	0.0220
cg10822545	19: 13985513	*MIR181D; MIR181C*	TSS200; Body	S_Shore	—	0.833 ± 0.009	0.864 ± 0.007	0.031 (3.1%)	0.0220
cg02315626	17: 27188560	*MIR451; MIR144*	TSS200; Body	“open sea”	—	0.802 ± 0.011	0.841 ± 0.011	0.038 (3.8%)	0.0220
cg12530503	15: 89911148	*MIR9-3*	TSS200	Island	TRUE	0.129 ± 0.005	0.153 ± 0.007	0.024 (2.4%)	0.0349

AD = Alzheimer's disease; beta values are presented as means ± SEM; delta beta is the average of DNA methylation difference between the two compared groups; Mann-Whitney test, *p* values refer to the statistical analysis of the means of methylation levels from the two compared groups.

**Table 3:** Genes of ErbB signaling pathway analyzed in the cDNA microarray.

Gene symbol	fold_change (AD vs. Control)	*p* value
*MYC*	1.397076772	0.022^*∗*^
*MAP2K1*	1.324156793	0.029^*∗*^
*ABL2*	1.200432189	0.046^*∗*^
*AREG*	1.162855371	
*BRAF*	1.156142719	
*MTOR*	1.022415444	
*GAB1*	1.000617085	
*EGFR*	−1.008044545	
*PIK3CB*	−1.198562637	
*PIK3R3*	−1.405367210	

^*∗*^Differentially expressed genes between AD and controls (*p* ≤ 0.05, Student's *t*-test). AD = Alzheimer's disease.
