# Supplementary material for: Differential DNA Methylation of MicroRNA Genes in Temporal Cortex from Alzheimer's Disease Individuals
Source: Neural Plast. 2016 Apr 26;2016:2584940. doi: 10.1155/2016/2584940 (PMC4861808; doi:10.1155/2016/2584940)
Supplement: Supplementary file 1 — Supplementary Table 1 describes the clinical and pathological data of individuals used in gene expression analysis. Supplementary Table 2 presents 2,095 differentially methylated noncoding RNA CpG sites in the temporal cortex of AD subjects compared to controls. Supplementary Table 3 presents 161 differentially methylated CpG sites associated with miRNA genes. Supplementary Table 4 presents the predicted targets of the investigated miRNAs. We added this paragraph in the Casuistic and Methods section of the manuscript. [file 2584940.f1.pdf]

Supplementary Table 1. Clinical and pathological data of individuals used in gene expression analysis

	Age at death (yrs)	Gender	CDR	Stages of Senile changes		
Controls				Braak	CERAD	LB
1	71	F	0	0	0	-
2	79	M	0	1	0	-
3	81	F	0	1	0	-
4	77	M	0	1	A	-
5	57	M	0	0	0	-
6	65	F	0	0	0	-
7	59	F	0	0	0	-
8	89	M	0	2	0	-
9	82	F	0	0	0	-
10	94	F	0	2	0	-
Alzheimer’s disease						
1	99	F	3	5	B	-
2	82	F	2	4	B	-
3	86	F	1	4	C	-
4	83	F	3	5	A	-
5	69	M	2	6	C	-
6	87	F	3	5	B	-
7	82	F	2	5	C	-
8	77	F	3	4	A	-
9	83	F	3	6	C	-

Braak stage = neurofibrillary tangle; CERAD (Consortium to Establish a Registry for Alzheimer's Disease) = neuritic plaques; LB=Lewy body; CDR=Clinical Dementia Rate.

Name	CHR	Probe_SNPs	Probe_SNPs_10	UCSC_RefGene_Name
cg01391297	1			LOC645676;ASH1L
cg23676114	9			SUGT1P1;CHMP5;BAG1
cg23261413	11			LOC100130987
cg15700489	1			RNU11
cg11005826	11			IGF2AS;INS-IGF2;IGF2;IGF2AS
cg02587316	19			ZNF529;ZNF382;ZNF529;ZNF529
cg19768013	5			C5orf27
cg15951280	5			LOC728613
cg24789143	8	rs10090565		USP17L2;FAM66D
cg22725460	2			SH3RF3;LOC100287216
cg12974337	10			LOC283050;LOC283050;LOC283050
cg03438079	16			TRAF7;SNORD60
cg08307039	5			TRIM23;C5orf44;TRIM23;C5orf44;TF
cg13837916	6			GUSBL1
cg16696856	19			LOC113230
cg02282626	6	rs9391806		HLA-L
cg05446860	20			LOC284798;LOC284798;LOC284798
cg16503683	11	rs59856964		ZFP91;LPXN;ZFP91-CNTF
cg10098541	3			PDIA5;PDIA5
cg06241765	8			INTS9;INTS9;HMBOX1;HMBOX1;INT
cg11538417	6		rs34548063	DOM3Z;STK19;DOM3Z;STK19;STK19
cg02526277	11			LOC494141;LOC494141;LOC494141
cg01399860	9	rs71505264		NCRNA00094
cg27020253	6		rs80160561	HLA-L
cg14981132	12			HNRNPA1;HNRPA1L-2;HNRNPA1
cg22772418	7			HOXA11AS
cg12901283	17			COX11;COX11;COX11;COX11;COX11
cg27367045	16			MGC3771;MGC3771;ZNF205;ZNF20
cg24895779	19		rs62106093	UCA1
cg25358410	9			IARS;IARS;SNORA84;IARS
cg12605341	20			LOC388789
cg19376858	6			TNXB;TNXB;TNXA;STK19;STK19
cg13881327	3	rs2290311		RPL32;SNORA7A;RPL32;RPL32;RPL3
cg02613624	11	rs14367		INS-IGF2;IGF2;IGF2;IGF2
cg12432898	17			FLJ35220;LOC100294362;FLJ35220;
cg04582861	17			BRCA1;BRCA1;BRCA1;BRCA1;BRCA1
cg18801245	12			LARP4;LARP4;LARP4;LARP4;LARP4;L
cg07147033	1			MIB2;MIB2;MIB2;MIB2;MIB2
cg02213716	13			UBAC2;UBAC2;UBAC2;UBAC2
cg15742049	10			NCRNA00081;NCRNA00081;SHOC2;
cg06809295	11	rs2283194		KCNQ1OT1;KCNQ1;KCNQ1
cg12636325	17			RNF126P1
cg09677763	11	rs78285343		NUP98;PGAP2;NUP98;PGAP2;PGAP

cg23358625	19		RPSAP58
cg07074757	2		CLASP1;CLASP1;CLASP1;RNU4ATAC
cg00378218	17		SCARF1;SCARF1;SCARF1;SCARF1;SC/
cg13947830	1		MIB2;MIB2;MIB2;MIB2;MIB2;MIB2
cg13685329	2		CCT7;CCT7;CCT7;CCT7;C2orf7;CCT7;
cg11717189	11		INS-IGF2;IGF2;IGF2;IGF2
cg03940153	2	rs72223545	SMPD4;SMPD4;SMPD4;SMPD4;SMP
cg17455183	1		GORAB;GORAB;GORAB
cg07439409	6		MIR548H3;C6orf167
cg23010048	19	rs75075099	ZNF577;ZNF577;ZNF577
cg01101865	14		FLJ43390
cg27032957	6		HCG27
cg10146807	11		API5;API5;API5;API5
cg20503416	11		MALAT1
cg13556639	2		NEURL3
cg19031271	17		LOC100306951;INPP5K;INPP5K;INPF
cg16560453	3		ZBTB11;LOC100009676
cg27169539	14		RAB2B;RAB2B;TOX4;RAB2B;RAB2B
cg00485047	16		NDE1;MIR484;KIAA0430
cg08127941	6		TRIM39;HCG18;HCG18;TRIM39
cg11935027	4		SNHG8;SNORA24
cg02670096	5	rs79066055	LOC100268168;RPL26L1;LOC100268
cg04283751	10		C10orf41;ZNF503;C10orf41
cg16698201	6		SNORD50B;SNHG5;SNORD50A
cg21793016	6	rs3757333	ZNRD1;ZNRD1;NCRNA00171
cg02595819	19		ZNF547;TRAPPC2P1
cg19548283	14		FLJ31306;FLJ31306;ARID4A;ARID4A;
cg01640215	16	rs226058	CRYM;NCRNA00169
cg09545860	5		SAP30L;SAP30L;SAP30L;SAP30L
cg22012577	6		HLA-J;NCRNA00171
cg15066416	17		LOC100272146;C17orf57
cg01590439	8		NCRNA00051
cg20488697	17		LOC92659;MAFG
cg21751540	19	rs2914637	ZNF738
cg00301159	3		C3orf55;C3orf55;C3orf55;C3orf55
cg14025299	15		SPATA5L1;SPATA5L1
cg25828462	15		LOC283731;ISLR2;ISLR2;ISLR2;ISLR2
cg25233816	3	rs61735736	LOC344595;LOC344595;LOC100302(
cg16615348	5		VTRNA1-1
cg10605084	12		LOC144571
cg21782985	1		C1orf203;C1orf203;C1orf203;C1orf2
cg21271452	8		CHRAC1;CHRAC1
cg14833160	19		BAX;BAX;BAX;BAX;BAX
cg06808983	17		G6PC3;G6PC3;G6PC3;G6PC3

cg10621825	6			STL
cg01683044	12			HOXC4;HOXC6;HOXC5
cg05038466	2			TTC31;TTC31;CCDC142
cg03364381	21			NCRNA00111
cg02204046	2			MYCN;MYCN;MYCNOS
cg15855184	11			DKFZp779M0652
cg03402794	13			RPL21;RPL21P28
cg12634276	4			SNORA26;KIAA0114
cg17338969	7		rs3991551	PILRB;PMS2L1;PILRB
cg00559939	2	rs76690140		RAPGEF4;LOC91149
cg00187380	12			HOXC4;HOXC5;HOXC5
cg03041602	8	rs6984839		HAS2AS;HAS2
cg01811561	16			GFOD2;GFOD2;GFOD2
cg17512353	6			HLA-L
cg23246703	2			FLJ32063
cg01656770	3	rs3749386		LOC100128640;ACVR2B
cg27565645	19			BAX;BAX;BAX;BAX;BAX
cg18155032	2			TRIB2;TRIB2;TRIB2
cg07608496	21			MCM3APAS;LSS;LSS;LSS;LSS
cg10866755	2			ZNF385B;MIR1258
cg18433293	15			LOC729082
cg16127286	4	rs7678308	rs1573955	SNHG8;SNORA24
cg05411953	19			NCRNA00085
cg04539241	17			SNORA76
cg20727290	15	rs74895554		BBS4;HIGD2B
cg20431766	2			MYCN;MYCNOS
cg00891649	17			MAPT;MAPT;LOC100130148;LOC10(
cg24895178	17			HS3ST3B1;MGC12916
cg12148979	2			FAM128A;LOC150776
cg14915787	3	rs61740275		LOC344595;LOC344595;LOC100302(
cg09043226	6			AGPAT1;RNF5;RNF5P1
cg01931958	12	rs61742296		ERC1;ERC1;ERC1;ERC1;ERC1
cg08097631	14	rs61980703		C14orf48;C14orf48;C14orf48
cg04114368	1			BRP44;DCAF6;BRP44;BRP44;DCAF6
cg03163767	16			FAM96B;CES2;FAM96B;CES2
cg25102782	2			PRKRA;DFNB59;PRKRA;PRKRA;MIR5
cg26181880	10			MARVELD1;MARVELD1
cg11760602	12			LOC374443
cg10061567	6	rs17194824		TRIM39;HCG18;HCG18
cg23731991	7			FLJ43663;MKLN1
cg15226501	16			CHTF8;CHTF8;CHTF8;CHTF8;CIRH1A
cg17361803	10			C10orf41;C10orf41
cg11773816	4			LOC285548
cg15741124	17			CYB5D2;CYB5D2;CYB5D2

cg00580022	22			CECR4;CECR5;CECR5;CECR4
cg17051733	3			SOX2OT;SOX2
cg14460816	14			FLJ31306;ARID4A;FLJ31306;ARID4A;
cg08198480	5			LOC285696
cg10641615	12			HOTAIR
cg08598296	10			RHOBTB1;RHOBTB1;RHOBTB1;RHOE
cg06636001	8	rs12676034		FLJ10661;FLJ10661;FLJ10661
cg11844660	13			UBAC2;UBAC2;UBAC2
cg12336877	9			LOC100289341;MAN1B1
cg07203320	3	rs78321136		COPB2;COPB2
cg04739460	19		rs78042922	ZNF415;ZNF415;ZNF415;ZNF415
cg08365618	3			OXSM;NGLY1;OXSM;OXSM
cg04918358	2	rs56913233		LMAN2L;LMAN2L;LMAN2L;LMAN2L;
cg18755204	10			FAM45B;FAM45A
cg13844899	7			EFCAB10
cg16981259	6			BAT1;SNORD84;BAT1
cg04474007	19		rs76526663	NCRNA00085
cg13524302	6			HLA-DPB2
cg18296956	5			VTRNA1-1
cg02275292	7			CCM2;CCM2;CCM2;CCM2
cg10501065	11			IGF2AS;INS-IGF2;IGF2;IGF2AS
cg26397662	15		rs17115340	SNORD115-14
cg23478098	5			LOC645323;LOC645323
cg12587382	1			FAM54B;FAM54B;FAM54B;LOC6464
cg02891153	7			LOC100128822
cg08887961	10			RHOBTB1;RHOBTB1
cg20680460	1		rs74849649	BRP44;BRP44;DCAF6;BRP44;DCAF6
cg13795840	19			CIRBP;CIRBP;C19orf24
cg04621664	6			C6orf48;C6orf48;C6orf48;SNORD48;
cg15737319	2			MTHFD2;MTHFD2;MTHFD2
cg11496747	3			PDIA5;PDIA5
cg10319512	2			ZEB2;ZEB2;ZEB2
cg06868473	12	rs7301155		SHMT2;SHMT2;SHMT2;SHMT2;SHM
cg20560141	12			LOC338758
cg22358797	18			MBD2;SNORA37;MBD2
cg18470891	12			FOXM1;C12orf32;C12orf32;FOXM1;
cg21910709	5	rs72703166		BRD9;BRD9;BRD9;TRIP13;TRIP13
cg14752069	8			FAM66D
cg00449210	7			CBLL1;CBLL1
cg04891959	17			SHBG;SHBG;SAT2
cg17665927	11	rs78341923		INS-IGF2;INS-IGF2;IGF2;IGF2
cg12616721	3			LOC100128640;ACVR2B
cg04079206	9			LOC340508
cg16767968	17			SCARNA16;C17orf86

cg04580897	3	rs3772142		SNORA6;RPSA;RPSA
cg01101058	14			SFRS5;SFRS5;LOC100289511
cg26071823	14			DCAF11;DCAF11;DCAF11;DCAF11;D[illegible]
cg13304297	3			C3orf50
cg04134452	16	rs17135720		SNHG9;SNORA78;RPS2
cg12825059	10			SEC61A2;SEC61A2;SEC61A2;SEC61A
cg08880423	12			NCRNA00173;NCRNA00173
cg09178844	1			BTF3L4;BTF3L4;TXNDC12;BTF3L4
cg15662822	6			BTN2A3
cg02352653	5			SMAD5;SMAD5;SMAD5;SMAD5OS
cg12362060	15			LOC729082
cg19974448	10			LOC100169752
cg06503216	16			ATXN1L;ATXN1L
cg23628968	1			SPEN;FLJ37453
cg07022885	11			KIAA0652;KIAA0652;KIAA0652;HAR[illegible]
cg24401870	13			KLHL1;KLHL1;ATXN8OS
cg07203134	3			TNIK;TNIK;TNIK;TNIK;TNIK;TNIK;TNI[illegible]
cg25896734	7			EFCAB10
cg15842967	11			KDM2A;KDM2A
cg15846718	6			COX7A2;COX7A2
cg14629287	6			LOC285830;LOC285830
cg14820798	7			ZNF767;ZNF767
cg00704554	11			LOC100128239
cg04080595	1			PRDM16;FLJ42875;PRDM16;FLJ4287[illegible]
cg06254556	20		rs1203897	C20orf56
cg20095680	19			BAX;BAX;BAX;BAX;BAX
cg27294431	1			TPR;C1orf27;MIR548F1;C1orf27;C1[illegible]
cg00577578	1		rs75003897	GBAP1
cg08596028	7			SSBP1;FLJ40852
cg25349370	5			FGF1;FGF1;FGF1;FGF1;FGF1;FGF1
cg12426141	15			TCF12;TCF12;LOC145783;TCF12;TCF
cg14594481	12			MLF2;MLF2
cg18403792	5			LOC645323;LOC645323;LOC645323
cg15950337	11	rs77980976		KIAA0652;KIAA0652;KIAA0652;KIAA[illegible]
cg25326570	20			GNAS;GNAS;GNAS;GNASAS
cg05550612	16			GLG1;GLG1;GLG1;GLG1;GLG1
cg11119235	7			ST7;ST7;ST7OT4;ST7OT1;ST7;ST7
cg00735843	14			MUDENG;MUDENG;EXOC5;MUDEN[illegible]
cg01363734	12			TESC;TESC;TESC
cg25000382	16			LOC652276
cg03655940	16			MIR484;NDE1;NDE1;KIAA0430
cg04561804	3			LOC100128164;SEC62;LOC10012816[illegible]
cg27026909	14			FLJ31306;FLJ31306;ARID4A;ARID4A;
cg10336144	1			NFYC;NFYC;NFYC;LOC100130557;NF[illegible]

cg02599225	10			LOC219347;C10orf57;LOC219347;LC
cg09039689	11			KIAA0652;KIAA0652;KIAA0652;HARI
cg09694722	11			INS-IGF2;IGF2AS;IGF2;IGF2;IGF2AS;I
cg20196910	2			DCAF17;METTL8;METTL8;DCAF17;D
cg16298927	6			C6orf122;C6orf208
cg13167753	1		rs34437895	SYT14;SYT14;SYT14;SYT14;SYT14;SY
cg01872779	4			MFSD10;C4orf10;MFSD10
cg01187997	11			HCCA2;LOC338651
cg12364131	15			LOC254559
cg05777976	11	rs17881164		IGF2AS;INS-IGF2;IGF2;IGF2;IGF2AS
cg19356389	6			RING1;MIR219-1
cg12452106	3			C3orf49
cg07523753	1			FLJ42875
cg14526843	3			KPNA1;KPNA1
cg15131784	3	rs11706576		COPB2;COPB2
cg27578974	19		rs74924657	LOC284441
cg19440484	8			C8orf83
cg13915892	2			LMAN2L;LMAN2L;LMAN2L;LMAN2L;
cg01522721	19			MIR1181;CDC37
cg01134309	16		rs55973164	ACSF3;ACSF3;ACSF3
cg26781726	12		rs11043201	LOC338799
cg10531748	10			LOC100128292;DLG5
cg25428451	1			LOC339524;LOC339524;LOC339524;
cg19463885	2			DTYMK;DTYMK;DTYMK
cg09452728	11			LOC100128239
cg16085042	12			LOC253724;HSP90B1
cg21789280	1			HSPC157;HSPC157
cg10650127	11			IGF2;INS-IGF2;INS-IGF2
cg14134703	5			BRD9;BRD9;BRD9
cg26793905	1			RNU11
cg08215532	7			DPY19L2P4
cg03250742	4		rs922697	SPON2;LOC100130872-SPON2;SPON
cg00671225	6	rs15479		RPS12;SNORA33
cg19256675	7			PMS2L2;STAG3L1;STAG3L1
cg19342782	1			HHLA3;ANKRD13C;HHLA3;HHLA3;HI
cg26561998	4			LOC729338;BBS12
cg15559584	6	rs1059288		TAPBP;TAPBP;RGL2;RGL2
cg06193393	7		rs3735244	GATS;GATS;GATS;GATS
cg09763180	10			MIR202
cg26366417	3			SDHAP2
cg08892370	1			MGC12982
cg26979056	22			UBE2L3;UBE2L3;UBE2L3
cg04317940	7			PMS2;AIMP2;PMS2
cg16204552	7			RPS2P32

cg24534135	2			LOC643387;LOC151174;LOC151174
cg08039008	3	rs4927700		SDHAP1
cg02642822	17			LOC404266;LOC404266
cg10423910	5			SLC12A2;FLJ33630
cg12029639	13			MIR548F5;NBEA;MAB21L1
cg19271190	6			LST1;LST1;LST1;LST1;LST1;LST1;LST1
cg03754250	10	rs1612433		ZNF438;ZNF438;ZNF438;ZNF438;ZN
cg26180383	8			MIR1207;PVT1
cg13413744	9			SUGT1P1;NOL6;NOL6
cg13319286	10	rs12263149		RHOBTB1;RHOBTB1
cg11985680	1			SNORD45C;RABGGTB
cg09891393	16			LOC100129637
cg13443953	12	rs2231747		NECAP1;NECAP1
cg06384413	17			LOC404266;LOC404266;LOC404266;
cg09000356	17			SCARNA16;C17orf86
cg14674124	10		rs78846868	MIR202
cg05300697	1			BRP44;DCAF6;BRP44;BRP44;DCAF6
cg09741592	12			HNRNPA1;HNRPA1L-2;HNRNPA1
cg14431528	14			MIR453;MIR485;MIR668
cg01783662	3			SOX2OT
cg12829142	7			RPS2P32
cg20004406	19			ZNF542;ZNF542;ZNF542;ZNF542
cg11023442	17			LOC100306951;INPP5K;INPP5K;INPF
cg14971895	10			GPR158;LOC100128811
cg03841560	21			MCM3APAS;LSS;LSS;LSS;LSS
cg15207422	19			LOC729991-MEF2B;LOC729991-MEF
cg06938143	8			C8orf12
cg13378519	1			GNRHR2;PEX11B
cg26859841	1			DUSP5P
cg24842733	6			SNAP91;SNAP91
cg08445226	3			C3orf26;MIR548G
cg08268266	16			LITAF;LITAF;LITAF;LITAF
cg18323835	5	rs34933713		HINT1;HINT1;HINT1
cg22138461	2			DCAF17;METTL8;METTL8;DCAF17;D
cg11882478	17			SHBG;SAT2;SHBG
cg11346962	7			FLJ45340
cg14092529	5			LOC728411;LOC728411;LOC728411
cg02425416	11	rs17885389		IGF2AS;INS-IGF2;IGF2;IGF2;IGF2AS
cg13750180	7			ZNF815
cg11985277	14			FUT8;FUT8;FUT8;FUT8;FUT8;FUT8;L
cg08610383	16			GFOD2;GFOD2;GFOD2
cg18020072	6	rs9688644		SNORA38;BAT2
cg19747960	8		rs79576182	C8orf12
cg24949133	16		rs153211	RRN3P2

cg02238136	3			ZNF620;ZNF620
cg27304754	16			CALB2;CALB2;CALB2
cg14184693	15			SERF2;MIR1282
cg11321965	19			MORG1;MORG1;C19orf56;MORG1;
cg04628802	16			CALB2;CALB2;CALB2
cg02672678	3			NCBP2;LOC152217;NCBP2
cg18268988	11			TIMM8B;SDHD;TIMM8B
cg03658244	18		rs77243838	MAPRE2;MAPRE2;MAPRE2;MAPRE2
cg18159646	11			SNORD22;SNORD31;SNORD30;SNH
cg24569270	16			MGC23284;MVD;MGC23284
cg03810913	10			LOC283050;LOC283050;LOC283050;
cg15229153	17			RPAIN;RPAIN;RPAIN;RPAIN;RPAIN;R
cg17783086	17	rs75874866		LOC146880;LOC146880
cg23384340	11	rs12786431		MED19;TMX2;TMX2;TMX2
cg08016363	17	rs59253142		TMEM11;TMEM11
cg22806002	11			BTG4;MIR34C;MIR34B
cg00221327	6			DAXX;DAXX;DAXX;DAXX
cg00565075	22			MIR659;EIF3L
cg02662155	11			KIAA0652;KIAA0652;KIAA0652;HAR
cg00909926	1			ESRRG;ESRRG;ESRRG;ESRRG;ESRRG
cg06800444	8			TM2D2;TM2D2;ADAM9;ADAM9;AD
cg14286941	20			EDEM2;EDEM2;EDEM2
cg02398725	11			CREBZF;CREBZF;CREBZF;CREBZF;CRE
cg07599979	21			DSCR9
cg13303256	6			PHF1;PHF1;PHF1
cg18735641	6	rs17190183		HCG22
cg22226091	18			ATP5A1;HAUS1;HAUS1
cg16474117	12			CALCOCO1;CALCOCO1;CALCOCO1
cg01644741	20			MIR1259;SNORD12B;C20orf199;C20
cg05663643	6			ZNRD1;ZNRD1;NCRNA00171
cg14835962	22			THAP7;THAP7;FLJ39582;FLJ39582
cg26439916	16			TRAF7;SNORD60
cg13149127	1			C1orf61;MIR9-1
cg18183073	5			FLJ42709;FLJ42709
cg25267526	19			MORG1;MORG1;MAN2B1;MORG1
cg06951969	15			SMAD6;SMAD6
cg07679725	2			TMEM185B
cg15397506	4			LOC93622
cg03382121	6			DAXX;DAXX;DAXX;DAXX
cg10259392	11			LRTOMT;LRTOMT;NUMA1;LRTOMT;
cg13780516	9			GGTA1
cg15213565	6			TRIM39;HCG18;HCG18;TRIM39
cg16880856	7			FABP5L3;MLL3
cg10387807	1			C1orf27;C1orf27;MIR548F1;C1orf27

cg14037240	10	rs9424166	rs75365661	ASB13;ASB13
cg18846731	11			CREBZF;CREBZF;CREBZF;CREBZF;CRE
cg20863107	13			DLEU2
cg22841336	12			ATP5B;SNORD59A
cg05205351	20	rs73576051		NOP56;SNORD110;NOP56;SNORA51
cg00704289	14			RAB2B;TOX4;RAB2B;RAB2B
cg04270358	5			LOC645323;LOC645323;LOC645323
cg02780767	12		rs4883091	LOC653113
cg08329777	17			LOC92659;MAFG
cg04114269	3			C3orf26;FILIP1L;MIR548G;FILIP1L
cg15160445	20			GNAS;GNAS;GNAS;GNASAS
cg03626025	15			CCNDBP1;CCNDBP1;CCNDBP1;CCND
cg12480658	2			ATF2;MIR933
cg08375286	6			DAXX;DAXX;DAXX;DAXX
cg15460330	8	rs7840270		TM2D2;TM2D2;ADAM9;TM2D2;ADA
cg06685111	6			HCG18;TRIM39;TRIM39;HCG18;TRIM
cg22230395	5			FAM114A2;MFAP3;MFAP3;MFAP3
cg14088052	7			CCM2;CCM2;CCM2;CCM2
cg20440041	19			ZNF382;ZNF529;ZNF529;ZNF529
cg11136708	22			ZNF74;ZNF74
cg09522706	7			MCM7;MCM7;MIR25;MIR93
cg03046332	3			FYTTD1;FYTTD1;FYTTD1;KIAA0226
cg16311158	13			UBAC2;UBAC2;UBAC2;UBAC2
cg09537434	19	rs13346603		ATP5SL;ATP5SL;ATP5SL;ATP5SL;ATP5
cg04088697	7	rs1476605		DLX6AS
cg19910802	14		rs74086033	C14orf132
cg08335854	5			LOC285696;BASP1
cg00819055	2			UGGT1;UGGT1
cg27495572	2		rs11896779	TTN;TTN;TTN;MIR548N;TTN
cg06379531	1			TSEN15;TSEN15;TSEN15
cg11995506	6			RNF5;AGPAT1;RNF5P1;RNF5
cg21085351	17			SNORD104;SNORA76
cg16817891	11			IGF2AS;INS-IGF2;IGF2;IGF2AS
cg02000145	6	rs2239526		BAT1;SNORD84;BAT1
cg19182014	20	rs76425596		HMGB3L1
cg11111696	10			ZNF438;ZNF438;ZNF438;ZNF438;ZN
cg14230378	3			NUDT16P;NUDT16P
cg19543987	2		rs57336116	CCT7;CCT7;CCT7;CCT7;C2orf7;CCT7;
cg21356536	6			MOCS1;MOCS1
cg21358678	6			RNF5;RNF5P1
cg07793148	1			C1orf200;PIK3CD
cg14645481	5			HINT1;HINT1;HINT1
cg17606683	11			C11orf73;C11orf73;C11orf73;C11orf
cg14036884	19			XAB2;LOC100131801;LOC10013180

cg17752846	2		MTX2;MTX2;MTX2
cg18442830	17		LOC284009
cg17416608	12		ERC1;ERC1;ERC1;ERC1;ERC1
cg02505002	19	rs61739688	C3P1
cg13346869	8		LOC728024;ERLIN2
cg20333067	17		ZNF207;MIR632;ZNF207;ZNF207
cg26529556	17		SNORD104;SNORA76
cg02233558	5		SLC12A2;FLJ33630
cg07962315	9		OSTF1;C9orf95;C9orf95;C9orf95
cg27426882	7		PMS2L11
cg09994773	1		MIR34A
cg03997643	9		RNU6ATAC
cg07363270	2		LOC440925
cg11572390	1		SMG5;TMEM79;TMEM79
cg06868758	2		EEF1B2;EEF1B2;SNORD51;NDUFS1;E
cg15981195	9		FLJ35024;VLDLR;VLDLR
cg24451074	3		H1FX;C3orf47
cg20009101	22		psiTPTE22
cg17036441	3		NCBP2;LOC152217;NCBP2
cg18088712	1		CROCCL2
cg20289299	6	rs1059306	SNHG5;SNORD50B;SNORD50A
cg05200614	14		MEG3;MEG3;MEG3
cg23837897	17	rs12937360	COX11;COX11;COX11;COX11;STXBP4
cg23576358	4		LOC93622
cg15196165	6		HCG9
cg16682926	20		GNASAS
cg20787693	1		FAM54B;FAM54B;FAM54B;FAM54B
cg04690289	3	rs79296608	ZDHHC3;ZDHHC3;EXOSC7;ZDHHC3;E
cg25730804	7		DPY19L2P4
cg18080509	6		C6orf147
cg26580576	4		LOC93622
cg01356463	12	rs4767067	C12orf47;MAPKAPK5;MAPKAPK5
cg20027050	11		LRTOMT;LRTOMT;LRTOMT;LRTOMT;
cg26336277	3		FYTTD1;KIAA0226;KIAA0226;FYTTD1
cg04598683	11		NCRNA00167;PRDM10;PRDM10
cg14622857	15		LOC646214
cg21420531	2		SLC5A6;C2orf28;SLC5A6;C2orf28;C2
cg01171597	15		ADPGK;ADPGK;ADPGK
cg07664027	19		RPL13AP5;RPL13A
cg09670616	2		ZEB2;ZEB2;ZEB2;ZEB2;ZEB2
cg00617305	1		SHISA4;SHISA4;SHISA4
cg26419621	1	rs41270837	SNHG3-RCC1;SNHG3;SNHG3-RCC1;S
cg11540416	15		HEXA;C15orf34
cg16906300	20		HSPC072;LOC100270804;HSPC072

cg03019000	3			TEX264;TEX264;TEX264
cg02779230	1			TRNAU1AP;TRNAU1AP
cg23521835	6	rs13197636		STL
cg06048102	13			GUCY1B2
cg18912574	7			NCRNA00174
cg15671181	18			AQP4;AQP4;C18orf16
cg01194468	9			SUGT1P1;CHMP5;BAG1
cg08798701	3			RPL32P3
cg16865908	11			LOC399959;MIR125B1
cg06368721	6			STK19;STK19;DOM3Z;STK19
cg05384664	11	rs3213232		INS-IGF2;IGF2;IGF2;IGF2
cg10866856	20			FER1L4
cg19415743	11			TMEM41B;TMEM41B;TMEM41B
cg16065241	17			DNAJC7;NKIRAS2;DNAJC7;DNAJC7
cg20271396	1			TSEN15;TSEN15;TSEN15
cg04894169	3	rs3731055		XPC;XPC;XPC;LSM3
cg03876030	11			NCRNA00167;PRDM10;PRDM10
cg27228559	10			CASC2;RAB11FIP2;CASC2;CASC2
cg12376277	22			TUG1
cg16769595	3			MOBP;MOBP
cg08576598	7		rs3034485	WNT2;WNT2
cg03543403	12			DIABLO;DIABLO;DIABLO;DIABLO
cg03172947	20			DBNDD2;DBNDD2;DBNDD2;DBNDD
cg02997086	17			DNAJC7;DNAJC7;DNAJC7;NKIRAS2
cg14009632	6			TJAP1;TJAP1;TJAP1;TJAP1;TJAP1;TJA
cg05827631	1			LOC647121
cg26227225	1			C1orf86;LOC100128003;C1orf86
cg09697458	16			CALB2;CALB2;CALB2
cg00328284	8			ANK1;ANK1;ANK1;ANK1;ANK1;ANK1
cg17176195	11	rs72166545		LOC100133315;RNF121;RNF121;RN
cg04556854	4	rs61739499		BBS12;LOC729338
cg03054141	12			NECAP1;NECAP1
cg16265348	20			LOC284798;LOC284798;LOC284798
cg26900259	5			TRIM23;C5orf44;TRIM23;TRIM23;C5
cg04712457	11	rs35773617		OTUB1;OTUB1
cg23489587	5			LOC728554
cg23395310	9			SNHG7;SNHG7;SNHG7
cg17542751	22			PARVG;PARVG
cg23940614	15	rs923968		SH3GL3;SH3GL3
cg25477181	22			PITPNB;LOC284900;LOC284900
cg09250933	2			PAX8;PAX8;PAX8;PAX8;PAX8;LOC44
cg17143518	18			CABLES1;CABLES1
cg01492656	4			N4BP2;LOC344967
cg11456838	6			OSTCL

cg13947334	9			C9orf27
cg14244887	9			LOC554202
cg24343312	7			LOC401397;LOC401397
cg12608507	2			EEF1B2;EEF1B2;SNORD51;NDUFS1;S
cg11150068	10			XPNPEP1;XPNPEP1;XPNPEP1
cg00004963	6	rs259389		LOC729176;C6orf103
cg22877570	3			TNIK;TNIK;TNIK;TNIK;TNIK;TNIK;TNIK
cg02895602	14	rs76713753		MIR1185-2
cg23146741	8			RNF170;RNF170;RNF170;RNF170;RN
cg18340059	14			RTL1;MIR431;MIR433
cg19131227	11			IGF2AS;INS-IGF2;IGF2;IGF2;IGF2AS
cg02819921	2			E2F6;E2F6;E2F6;E2F6;E2F6
cg17602882	19			MIR1181;CDC37
cg04834666	20			EDEM2;EDEM2;EDEM2
cg18730744	20			SNORD119;SNRPB;SNRPB
cg10638415	19			ZNF542;ZNF542;ZNF542;ZNF542
cg06189265	1	rs75315904		GAS5;SNORD76;SNORD75;SNORD77
cg10781814	19		rs73922817	LOC729991-MEF2B;LOC729991-MEF
cg01047218	11			KDM2A;KDM2A
cg13247671	7			MKLN1;FLJ43663
cg07708721	6			SNORA38;BAT2
cg03108344	6	rs7762217		RAET1K
cg12760563	18			DLGAP1;FLJ35776;DLGAP1
cg10075436	19			MZF1;MZF1;MZF1;MZF1;LOC100131
cg05937182	7			ST7OT4;ST7OT1;ST7;ST7
cg16220802	4	rs75022636		MAPKSP1;MAPKSP1
cg14633504	5			VTRNA1-1
cg08322240	8			ZFHX4;LOC100192378
cg18090064	17			C17orf69;C17orf69
cg16259268	2			EFHD1
cg05648582	9			LOC100129066
cg10791580	6	rs9402287		LOC285733
cg05658236	1			MGC12982
cg21268653	1			C1orf203;C1orf203;C1orf203;C1orf2
cg01695023	22			P2RX6P
cg22872906	4			LOC285419;LOC285419
cg17002091	14			SLC25A29;MIR345;SLC25A29
cg17606003	10			FLJ41350;LBX1
cg22424444	11			LOC255512;TOLLIP
cg02413874	16			LOC100134368
cg03601585	11			SNORD27;SLC3A2;SLC3A2;SNORD28
cg10436540	17			ZZEF1;CYB5D2;CYB5D2;CYB5D2
cg08182240	11			TBRG1;TBRG1
cg04061361	15		rs41286586	SNORD116-1

cg25889944	7			SBDSP;SBDSP;SBDSP;TYW1B;TYW1B
cg21701531	2			ZEB2;ZEB2;ZEB2
cg24765924	5	rs79940373		LOC255167;LOC255167
cg24123518	15			ISLR2;LOC283731;ISLR2;ISLR2;ISLR2
cg12069073	11			KDM2A;KDM2A
cg07429629	14			MIR494
cg08421051	12			LOC338799
cg11581046	19			MIR639;TECR
cg10592336	4			LOC550112;UBA6
cg10285420	6			DAXX;DAXX;DAXX;DAXX
cg23156781	2			SLC5A6;C2orf28;SLC5A6;C2orf28;C2
cg17709884	4			H2AFZ;LOC256880
cg14983728	22			TOM1;TOM1;TOM1;TOM1;TOM1;T(
cg05355679	1			SNORD55;SNORD46;RPS8
cg16688046	9			NCRNA00094
cg02732671	3			CEP63;ANAPC13;ANAPC13;CEP63;AI
cg19844233	11			CREBZF;CREBZF;CREBZF;CREBZF;CRE
cg02649933	6	rs16893483		ZNRD1;ZNRD1;NCRNA00171
cg01284415	19			BAX;BAX;BAX;BAX;BAX
cg20365618	10	rs78311227		INPP5F;INPP5F
cg22209624	1			SERTAD4;C1orf133
cg01733045	3			TEX264;TEX264;TEX264
cg20189761	8			REXO1L2P;REXO1L1
cg12124911	12			RACGAP1P
cg14031414	20			ESF1;C20orf7;C20orf7;C20orf7
cg00285343	7			LOC285954;LOC285954;INHBA
cg20377766	10	rs71487871		LOC387646
cg10265786	6		rs75831035	DOM3Z;STK19;STK19;DOM3Z;STK19
cg03591594	9			VLDLR;VLDLR;VLDLR;VLDLR;FLJ3502
cg21871735	16			SNHG9;RPS2;SNORA78
cg12792526	21			NCRNA00111
cg05863098	14	rs61980703		C14orf48;C14orf48;C14orf48
cg03322749	9			RBM18;MRRF;RBM18;RBM18;MRRF
cg20254273	1			MDS2
cg03306972	3			NCBP2;LOC152217;NCBP2
cg17797815	10			CASC2;RAB11FIP2;CASC2;CASC2
cg24154325	14			C14orf23;C14orf23
cg25134231	2			CLASP1;CLASP1;CLASP1;RNU4ATAC
cg22889142	19			NCRNA00181;A1BG
cg01665432	12			SNORA2A;C12orf41
cg04091325	15			SH3GL3;SH3GL3
cg27210565	10			CDC10L
cg13443893	6			DAXX;DAXX;DAXX;DAXX
cg10020897	1			BRP44;BRP44;DCAF6;BRP44;DCAF6

cg13611065	22		LOC96610
cg06477715	13		LOC100288730;PAN3;PAN3
cg10956413	22		TMEM191A
cg18189409	22		LOC400891;P2RX6P
cg26332552	3		MIR922;KIAA0226;KIAA0226
cg11671290	6		BAT1;SNORD84;BAT1
cg03790236	11		ANKRD13D;ANKRD13D
cg22225943	11		IGF2AS;INS-IGF2;IGF2;IGF2AS;IGF2
cg11031889	15		NGRN;NGRN
cg08515869	4		MIR573
cg14387656	2		EFHD1
cg04648747	12		TBC1D15;TBC1D15;TBC1D15;TBC1D
cg25745713	1		TXNDC12;BTF3L4;BTF3L4;BTF3L4
cg15823872	1		LOC441869;LOC441869
cg09358388	15		SNORD109B;SNORD109A
cg15443793	19		ECSIT;ECSIT;ECSIT;ECSIT
cg06708720	12		ERC1;ERC1;ERC1;ERC1;ERC1
cg22236626	1		NENF;NENF
cg07211511	3		LOC729375
cg05017015	14		DCAF11;DCAF11;DCAF11;DCAF11;D[illegible]
cg01747862	4		NBLA00301
cg22817659	8	rs34819009	LY6E;LOC100133669;LY6E
cg09436420	3		LOC100128164;SEC62;LOC10012816[illegible]
cg12932539	22		TOM1;TOM1;TOM1;TOM1;TOM1;T[illegible]
cg06395988	2		MTERFD2;MTERFD2;MTERFD2;MTE[illegible]
cg14611084	7		FLJ35390;FLJ35390;RASA4P
cg07383130	1		FLJ37453
cg04838987	20		EDEM2;EDEM2;EDEM2
cg00797104	6		PPP1R2P1
cg06967120	10		LOC100128811;GPR158
cg06174989	16		LOC100130015;LOC100130015
cg04195831	1		MST1P9
cg26833449	7	rs73104620	Sep-13
cg20210449	7		FAM185A;FAM185A;FAM185A
cg01250407	7		WNT2;WNT2
cg21371335	17		DNAJC7;DNAJC7;DNAJC7;NKIRAS2
cg20272962	17		LPO;LPO;LPO
cg07535928	14		SLC25A21;SLC25A21;LOC100129794
cg14017196	12		HNRNPA1;HNRPA1L-2;HNRNPA1;CB
cg19470159	3		C3orf50
cg21158476	5		LOC389332
cg00366813	2		DCAF17;METTL8;DCAF17;DCAF17
cg08264435	3		H1FX;C3orf47
cg06599927	1		MST1P2

cg21444593	1			SNORA14B;TOMM20
cg11351987	19			C19orf30
cg03158561	11			RPL27A;SNORA45
cg19922435	4			LOC285419;LOC285419
cg18426224	15		rs74196431	GOLGA8B
cg08411735	13			TPT1;LOC100190939
cg11731114	10			FLJ45983;GATA3;GATA3;FLJ45983
cg13408636	14			PPP1R3E
cg17152135	6			PHF1;PHF1;PHF1
cg26728851	11			GUCY2E
cg26171815	5			MIR580;LMBRD2
cg23374762	18			TXNL1;TXNL1
cg11014109	11		rs11278358	NARS2;NARS2
cg21404028	1			TSEN15;TSEN15;TSEN15
cg19348206	10			MIR202
cg07717512	6			HCG22
cg01271129	3			KCNMB3;KCNMB3;KCNMB3;KCNMB
cg15564267	11			OTUB1;OTUB1
cg27212729	2			LOC440925;SP5
cg06459669	10			XPNPEP1;XPNPEP1;XPNPEP1
cg04406441	4		rs77043177	RPL34;RPL34;LOC285456
cg12488414	19			LOC284440
cg15986668	1			NFYC;NFYC;NFYC;LOC100130557;NF
cg07177756	1	rs12090169		SNORD75;SNORD78;ZBTB37;ZBTB37
cg25563983	22			CECR7
cg08721491	6			STK19;DOM3Z
cg04686204	16			MGC23284;MGC23284
cg01729066	9			MIR600;C9orf45
cg16198013	6		rs75648664	LOC285740;LOC285740
cg10985987	16			LOC440356;LOC440356;CDIPT;CDIPT
cg09439093	6			PECI;PECI;PECI;PECI
cg01765473	10			ATAD1;CFLP1
cg26039806	11			RNF121;LOC100133315;RNF121;RNI
cg18170098	16			ATXN1L;ATXN1L
cg18370923	17	rs4792430		CDRT15P
cg03817476	2			CLK1;CLK1;CLK1;CLK1
cg01928411	12			LARP4;LARP4;LARP4;LARP4;LARP4;L
cg00698126	7	rs4727033	rs78854238	ZNF783
cg14636534	5			LOC645323;LOC645323;LOC645323
cg06988003	5			FLJ42709;FLJ42709
cg02405517	19			SNAPC2;SNAPC2
cg07854954	4			HNRPDL;HNRPDL
cg09452846	15			LOC91948;LOC91948
cg22946150	15			SH3GL3;SH3GL3;SH3GL3

cg08131204	22			MIR1281
cg10715637	10	rs75284214		FAS;FAS;FAS;FAS;ACTA2;FAS;FAS;FA
cg01522430	12			RPSAP52;HMGA2;HMGA2
cg03981685	1			LOC339524
cg10961700	1			SETDB1;SETDB1;SETDB1
cg17871739	19			LOC148189
cg18801197	3			KPNA1;KPNA1
cg24980994	10			ZNF503;ZNF503;C10orf41
cg16370685	1			SETDB1;SETDB1;SETDB1
cg04746199	2			PAX8;PAX8;PAX8;PAX8;PAX8;LOC44
cg06158244	3			LOC401093;MBNL1;LOC401093;MBI
cg24486235	1		rs7512192	LOC400794
cg18595867	1			MGC12982
cg03539765	12			LOC144571
cg01734628	6			RDBP;SKIV2L;MIR1236
cg15232450	3			SETMAR;SETMAR
cg08165875	12			EP400NL
cg10519437	14			CFL2;CFL2;CFL2;CFL2
cg22231012	6			DKFZP686I15217;DKFZP686I15217
cg04073608	11			MALAT1
cg18039643	11			SNORD27;SNORD26;SNORD29;SNOF
cg10273096	9			C9orf130;C9orf102;C9orf130
cg22829917	12	rs60674771	rs74348153	HOTAIR
cg17512365	6			HCG4
cg13287964	18			LOC100130522;LOC100130522
cg24576735	10			MARVELD1;MARVELD1
cg18014500	10			GUCY2G
cg21626735	13		rs61944965	DKFZp686A1627
cg04095971	6			ZNRD1;ZNRD1;NCRNA00171
cg18806997	1			SNORD46;RPS8;SNORD38A
cg01026898	11	rs80322963		LOC221122
cg26532627	17			LOC100133991;LOC100133991;C17o
cg25100475	1			ZNF678;ZNF678
cg00893045	15			SNORD18A;RPL4;SNORD18B;SNORD
cg00028034	20			TSPYL3
cg09859768	4			LOC641518;LOC641518
cg05927268	14			ABCD4;ABCD4
cg10247798	3	rs75054057		ABHD14B;ABHD14B;ABHD14A;ABHI
cg15085006	3			DHX30;DHX30;MIR1226
cg19535073	7			LOC285954;LOC285954;INHBA
cg07095072	7			MLL5;LOC100216545;MLL5
cg27633139	4			MIR548I2
cg25584081	22	rs470082		RPL3;SNORD43;RPL3
cg20300514	1			C1orf86;LOC100128003

cg22089899	3			ALG3;ECE2;ALG3;ECE2;ALG3;ALG3
cg05207077	12			FAM138D
cg26364911	9			HIATL2
cg18455650	2			PP14571;GPC1
cg09158487	12			HNRNPA1;HNRPA1L-2;HNRNPA1;CB
cg07740199	18	rs61173354		LOC284233
cg19734870	5			LOC645323
cg15413793	17			MGC57346;MGC57346
cg21170636	9			SNORD24;MED22;RPL7A;MED22
cg16704590	20			C20orf199;C20orf199;SNORD12B;SN
cg25591418	22			LOC730668
cg08643007	2			ATF2;ATF2;MIR933
cg11208853	12			LOC653113
cg24647276	10			FLJ45983;GATA3;GATA3;FLJ45983
cg09448652	11			SNORD30;SNORD22;SNORD31;SNHC
cg24330775	19			GYS1;GYS1;GYS1;RUVBL2
cg02616186	1			NAV1;MIR1231;NAV1
cg16067752	22	rs60039951		LOC96610
cg10177030	20			C20orf199;C20orf199;SNORD12;C20
cg00434295	13			SUGT1L1
cg21110646	11			TMEM41B;TMEM41B;TMEM41B
cg04810755	1			MST1P9
cg05378690	3	rs2077960	rs11557630	IP6K2;IP6K2;IP6K2;IP6K2;IP6K2;IP6K
cg10656255	15			DET1;DET1;DET1
cg15629460	1	rs67396868		ESPNP
cg20206204	12			MIR614
cg06559547	6			SNHG5;SNORD50B;SNORD50A
cg11601000	22	rs28722907		LOC339674
cg02020829	5	rs7712899		FLJ42709;FLJ42709
cg02003624	2		rs75904319	UGGT1;UGGT1
cg11389773	16			LOC641298;RRN3P3
cg07401773	9			HIATL2
cg12622938	7	rs2527272		STAG3L2;PMS2L5;PMS2L5;PMS2L5
cg03639021	5	rs61736974		GNB2L1;SNORD95
cg26759552	7			LOC100134229;JHDM1D
cg02743050	15			GRINL1A;GCOM1;GRINL1A;GCOM1;
cg04425997	20			SNORD119;SNRPB;SNRPB
cg16273041	11	rs2233351		KBTBD4;KBTBD4;NDUFS3;KBTBD4
cg23007574	19			ATP5SL;ATP5SL;ATP5SL;ATP5SL;ATP5
cg22050893	2			MIR548N;FKBP7;FKBP7
cg23167644	17			LOC100130581;LOC100130581
cg20539321	10		rs7095041	LOC219347;LOC219347;C10orf57;LC
cg13770529	17			CCDC55;MIR423
cg11821796	11			LOC100130987

cg09293122	7			GATS;STAG3;GATS;GATS;GATS
cg11668917	2			SNORA80B;ODC1
cg21505509	8	rs77030027		LOC728024;ERLIN2
cg07925542	16			MIR484;NDE1;NDE1;KIAA0430
cg21973405	5			PPAP2A;PPAP2A;RNF138P1
cg14390758	10			LOC100128811;GPR158
cg24394891	10			XPNPEP1;XPNPEP1;XPNPEP1
cg17274080	22			UBE2L3;UBE2L3;UBE2L3
cg01356611	19			SNAR-C5;SNAR-C2;SNAR-C1
cg01404873	13			DLEU2
cg26410121	1			LOC728875
cg02767068	16			CORO1A;LOC606724
cg19982557	2			FAM128A;LOC150776
cg03742947	6			HLA-H
cg25151638	1			SHC1;SHC1;CKS1B;CKS1B
cg01244514	14			MIR494
cg00781110	15			LOC283731;ISLR2;ISLR2;ISLR2;ISLR2
cg19746906	1			HHLA3;HHLA3;HHLA3;HHLA3;ANKRD
cg03863549	9			NOL6;SUGT1P1;NOL6
cg01612532	15	rs1058828		HERC2P2
cg21187554	9			ANKRD18A;ANKRD18A;C9orf122
cg08235057	22			TMEM191A
cg24764810	6		rs62401701	HCG22
cg09510698	6			HLA-DPB2
cg12778580	11			MIR1237;RPS6KA4;RPS6KA4
cg05856015	4			ZNF721;ABCA11P
cg13627197	22			TOP1P2;PIWIL3
cg21076271	14			ARHGAP5;ARHGAP5;ARHGAP5;C14c
cg14445849	9			VPS13A;VPS13A;LOC100286938;VPS
cg02973270	6			AGPAT1;RNF5;RNF5P1
cg13310628	16			SOLH;C16orf10
cg02329425	10			LOC100188947;HECTD2;HECTD2
cg00328051	14			DIO3;MIR1247
cg14872693	11			NEAT1
cg05096838	17			FAM134C;TUBG1;FAM134C;TUBG1
cg11902380	12			MIR1228;LRP1
cg08200543	1	rs75034630		C1orf86;LOC100128003;C1orf86
cg23288521	20			SNORA39;SNHG11
cg16223220	6	rs61232101		HLA-H
cg26398848	2			ACP1;ACP1;SH3YL1;ACP1;SH3YL1;AC
cg20557687	4			EDNRA;EDNRA;EDNRA;EDNRA;EDNR
cg05547902	15			LOC283731;ISLR2;ISLR2;ISLR2;ISLR2
cg19199266	17			C17orf69;C17orf69;MGC57346;MGC
cg05377034	10	rs3842235		C10orf57;LOC219347;LOC219347;LC

cg12846583	3			MIR1324
cg27548270	12		rs79994810	LOC653113
cg24607140	20			HM13;HM13;HM13;PSIMCT-1
cg19712659	1			SNHG12;SNORD99;SNORA61
cg17722033	12			NECAP1;NECAP1
cg22396798	15			CCNDBP1;CCNDBP1;CCNDBP1;CCND
cg16473117	2			AAK1;SNORA36C
cg19671533	6			SNAP91;SNAP91
cg12974668	6			MIR1275
cg01693157	2			TUBA4B;TUBA4A
cg07301105	2	rs12464121		C2orf52
cg23802887	16			MGC23284;SNAI3
cg05221349	4	rs11554385		LOC256880;H2AFZ
cg14892570	19			MIR1470;WIZ
cg09398845	4			LOC729338;BBS12
cg04471822	2			OR7E91P
cg07391831	17	rs28742029		ALOX12P2
cg00997234	6			C6orf147
cg23512275	21			LOC284837
cg04271391	6			DAXX;DAXX;DAXX;DAXX
cg08508335	13	rs75534413		DKFZp686A1627
cg24682012	11			SMPD1;SMPD1;SMPD1
cg17200337	4			C4orf10;MFSD10;MFSD10
cg19438469	12			ORMDL2;SARNP;SARNP;SARNP
cg19292611	13			TFDP1;TFDP1
cg04886198	3			ALG3;ECE2;ALG3;ECE2;ALG3;ALG3
cg27151174	16			CD2BP2;CD2BP2
cg11645417	6			PACRG;PACRG;LOC285796;PACRG
cg04148753	6		rs78769809	CDYL;CDYL;CDYL;CDYL
cg25364861	12			TESC;TESC;TESC
cg07126399	14			MIR412;MIR541;MIR409;MIR369
cg16739247	1		rs61485328	SNORD45C;SNORD45A;RABGGTB
cg09730836	5			NCRNA00219;SNORA13
cg11496432	8			LOC728024;ERLIN2
cg01966129	22			PRAME;LOC648691;PRAME;PRAME;
cg22469435	6			NCRNA00120;AKIRIN2
cg04216650	9			FAM27C
cg08285587	7			LOC100128822
cg03834909	19			CIRBP;C19orf23;CIRBP;CIRBP
cg26993102	6	rs28729266		HLA-L
cg17432135	20		rs78076898	SYS1;SYS1-DBNDD2
cg24832215	19		rs2123852	NAPSB
cg11565911	12			TBC1D15;TBC1D15;TBC1D15;TBC1D
cg26944449	16			ABCC6P1

cg03616030	8			TMEM67;TMEM67;TMEM67
cg08111922	14		rs34711197	MGC23270
cg00049502	12	rs4767068		C12orf47;MAPKAPK5;MAPKAPK5
cg08844770	19	rs2230562		EEF2;SNORD37
cg13632959	10			FAS;FAS;FAS;FAS;ACTA2;FAS;FAS;FA
cg03230579	10			ZNF37B
cg26719629	11			IGF2AS;INS-IGF2;IGF2;IGF2;IGF2AS
cg08160980	1			C1orf190;POMGNT1
cg15427004	3			ACAA1;ACAA1;ACAA1;MYD88
cg13518216	12			ATP5B;SNORD59A
cg08064553	16			CENPBD1;AFG3L1;AFG3L1;CENPBD1
cg13134825	14		rs2025146	SNHG10;GLRX5;SCARNA13;SNHG10
cg25306442	1			LOC400752
cg23447792	12			EP400NL
cg07516476	9			LOC100131193;C9orf86;C9orf86
cg24638668	12			H2AFJ;H2AFJ;H2AFJ
cg00974689	13			TFDP1;TFDP1
cg18654971	5			MIR449C;CDC20B;CDC20B;CDC20B
cg25996835	15	rs7178130		BBS4;HIGD2B
cg05066959	8		rs72638959	ANK1;ANK1;ANK1;ANK1;ANK1;ANK1
cg09965267	7			POLR2J4
cg09554007	16			RPL13;RPL13;SNORD68;RPL13
cg05781294	6			BAT1;SNORD117;BAT1
cg05325193	20			C20orf56
cg23893792	6	rs76592591		CDYL
cg15275313	11			C11orf36
cg05144089	1			SNORA14B;TOMM20
cg26979143	17			SCARNA16;C17orf86
cg04155793	16			CDK10;CDK10;CDK10;CDK10;CDK10;
cg12278467	5			LOC645323;LOC645323
cg08151281	1			DCAF6;BRP44;BRP44;BRP44;DCAF6
cg06319713	1			LOC440926;H3F3A
cg14156148	15			WASH3P
cg05461153	7			ZNF783
cg01629329	20			NOP56;MIR1292;NOP56;NOP56
cg14094543	4			LOC256880;H2AFZ
cg18090577	3	rs78923843		COL29A1;COL29A1
cg12163508	10		rs2800551	HSD17B7P2
cg10248302	11			MIR611;FEN1;C11orf10;FEN1
cg04415427	7	rs13245475	rs72106726	MGC72080
cg07456645	19			C19orf30
cg00049323	5			LOC25845
cg16372632	16			CES4
cg02698926	7			SDK1;SDK1

cg14937036	3			COPB2;COPB2
cg24569399	3			CCDC66;CCDC66;CCDC66
cg24357849	19			DHPS;MORG1;DHPS;DHPS;MORG1;
cg23022819	7			TBXAS1;TBXAS1;TBXAS1;TBXAS1;TB
cg02315626	17			MIR451;MIR144
cg21108361	7			LOC286016;TNPO3;TNPO3
cg17441804	6		rs9258230	LOC285830;LOC285830
cg18668382	14			GLRX5;SNHG10;SNHG10
cg27200833	15			ISLR2;LOC283731;ISLR2;ISLR2;ISLR2;
cg16181099	10	rs11186623		LOC100188947
cg04735310	17			MIR196A1
cg10051588	4		rs75453246	FLJ39653;FLJ39653
cg19484886	12			C12orf61;MIRLET7I
cg22601058	10			LOC283070
cg17493193	1			PDIK1L;PDIK1L;PDIK1L
cg26102503	20	rs45596642		GNASAS;GNAS
cg22756156	10			MARVELD1;LOC100270710;MARVEL
cg25666433	6			ZSCAN12;ZSCAN12
cg20310319	15			SNORD115-15;SNORD115-21;HBII-5
cg07936672	2			NEURL3
cg02837279	8			ZNF252;C8orf77
cg07913197	2			FLJ32063
cg13305165	3			NGLY1;OXSM;OXSM;OXSM
cg23523765	7			LOC349114
cg20624698	2			LOC339788
cg05030574	11			NEAT1
cg06197492	11	rs3741219		H19
cg17992509	1			MIR760
cg06048191	16			AFG3L1;CENPBD1;AFG3L1;AFG3L1
cg10591652	5			FBLL1
cg09906647	17		rs76606395	FLJ25006;LOC645851
cg12810221	12			ATP5B;SNORD59B;SNORD59A
cg00475112	12			LOC642846
cg22662482	11			FEN1;C11orf10;MIR611
cg04860656	1		rs11559315	WDR65;WDR65;EBNA1BP2;EBNA1B
cg17423209	22			POM121L9P
cg22901322	4			MAPKSP1;MAPKSP1;MAPKSP1
cg00253182	9			FLJ35024
cg21634218	19			FLJ26850
cg22406469	22	rs5756693		MFNG;MFNG;MFNG
cg25936380	2			TMEM185B
cg24876683	5			RPL26L1;LOC100268168;LOC100268
cg08461781	1			HIST2H2BA
cg07350076	8	rs79443365		FAM66A;DEFB109P1

cg17283752	6			CDYL;CDYL;CDYL;CDYL
cg19642877	11			IGF2AS;INS-IGF2;INS-IGF2;IGF2;IGF2
cg16543923	3	rs79224085		LOC220729
cg26371346	8			SNORD54;RPS20;RPS20
cg21300318	19			MIR7-3;C19orf30
cg25769852	12			CD69;CD69;CD69
cg02162069	19			ZIM2;ZIM2;PEG3;MIMT1;PEG3;PEG3
cg09148270	11			BTG4;C11orf88;C11orf88;MIR34C
cg02963846	17			SNORD7
cg00460092	10			ATAD1;CFLP1
cg10447247	12			NME2P1
cg06214925	4			LOC641518;LOC641518
cg19123296	2			ZEB2;ZEB2;ZEB2;ZEB2;ZEB2
cg08184640	14			FLJ45244;DICER1
cg02639007	12			CCDC41;CCDC41;LOC144486;CCDC4
cg21171800	5			LOC647859
cg22455660	9			PBX3;PBX3;PBX3;PBX3
cg12218193	1			CROCCL1
cg02114909	17			YWHAE;YWHAE
cg18319687	5			MIR1229;MGAT4B;MGAT4B
cg09570958	17		rs77690302	HS3ST3B1;MGC12916
cg01068014	13			MIR17HG;MIR17HG
cg19978242	10			INPP5F;INPP5F
cg07148743	5	rs77141166	rs35581525	BRD9;BRD9;BRD9
cg27567880	18			MBD2;SNORA37;MBD2
cg13974505	9			LOC389705
cg07830110	22			LOC400927
cg13360150	11			LIN7C;BDNFOS
cg03151540	4			TAPT1;FLJ39653;FLJ39653
cg24005950	16			SNORA10;RPS2
cg25531857	20		rs8590	C20orf199;C20orf199;C20orf199
cg18672602	3	rs2636939		LOC220729
cg16744883	8			FAM66B
cg27061903	6			COX7A2;COX7A2
cg12312819	2	rs3977003	rs2592451	CBWD2;LOC440839
cg05778739	2			LOC729234
cg14278808	8	rs75492345		LOC157627;MIR124-1
cg07291923	2		rs75602512	BRE;BRE;BRE;BRE;BRE;RBKS;LOC100
cg03183345	11		rs74901138	TBRG1;TBRG1
cg23083277	3			COL29A1;COL29A1
cg04278444	15			SNORD115-15;SNORD115-21;PAR4;[illegible]
cg18334734	5			LOC645323
cg04385236	3			C3orf55;C3orf55;C3orf55;C3orf55;[illegible]
cg12960248	16			GFOD2;GFOD2;GFOD2

cg02979839	5			FAM193B;FAM193B
cg02525307	20		rs73353672	LOC100134868
cg04250926	1			CRYZ;CRYZ;TYW3;CRYZ;TYW3;CRYZ;
cg20804661	11			LOC100133315;RNF121;RNF121;RN
cg00556742	2			C2orf60;C2orf60;C2orf47
cg08288469	12			HNRPA1L-2;HNRNPA1;HNRNPA1;CB
cg10430690	3			KALRN;KALRN;KALRN;KALRN;KALRN
cg00859352	3			TNIK;TNIK;TNIK;TNIK;TNIK;TNIK;TNI
cg06333167	2			PRKRA;DFNB59;PRKRA;PRKRA;MIR5
cg08634133	7			ATP6V0E2;LOC401431;ATP6V0E2;A
cg26359174	2			UGGT1;UGGT1
cg25214792	22			P2RX6;P2RX6;MGC16703
cg06872010	8			FAM66A
cg13838528	11			RPL27A;SNORA3
cg00678361	8			C8ORFK29
cg23029801	22			DGCR5
cg09039664	20			DBNDD2;DBNDD2;SYS1-DBNDD2;D
cg14158407	7			LOC401397;LOC401397
cg13857186	9			C9orf43;POLE3;C9orf43;POLE3
cg04122568	5	rs1060613		LOC100129716;ARRDC3
cg14244648	12		rs4767067	C12orf47;MAPKAPK5;MAPKAPK5
cg23685650	14		rs79849469	KTN1;C14orf33;KTN1;KTN1;KTN1
cg10661904	17		rs7406828	PDE6G;PDE6G
cg10154310	7			SSBP1;FLJ40852
cg09458394	1			SNORD45C;RABGGTB
cg08085357	3			SOX2OT
cg04221355	2			BRE;RBKS;BRE;BRE;BRE;BRE;LOC100
cg27248887	2	rs11689362		EEF1B2;EEF1B2;SNORD51;NDUFS1;
cg22732432	12	rs34470343		LOC400027
cg13131095	2			MYCN;MYCNOS
cg08252387	5	rs34698314		LOC645323;LOC645323
cg19751897	1			TXNDC12;TXNDC12;BTF3L4;BTF3L4;
cg00354542	16	rs56687322		LOC100134368
cg02484633	6			C6orf227
cg24082174	3			SNORA63;MIR1248;EIF4A2;SNORA8
cg11598872	17			FLJ45079
cg07720160	6	rs75519604		HLA-L
cg05374451	17			FLJ90757;BAIAP2;BAIAP2;BAIAP2;B
cg03479278	7			BCAP29;BCAP29
cg12885549	2			MIR548N
cg00960395	17			PRAC;C17orf93
cg01299854	22			PI4KAP2
cg25951256	10			HSD17B7P2
cg01191395	17			SHBG;SAT2;SHBG;SAT2

cg13413996	9			ADAMTS13;ADAMTS13;ADAMTS13;
cg08685077	7			NT5C3;NT5C3
cg04156464	16			GNAO1;GNAO1;LOC283856
cg01332827	20			EDEM2;EDEM2;EDEM2
cg16740905	19			SEC1;DBP
cg04600299	7			ATP6V0E2;LOC401431;ATP6V0E2;AT
cg00103783	17			MPDU1;MPDU1
cg23257859	7	rs2923266		MIR550-2
cg24775832	6	rs9360898		COX7A2;COX7A2
cg14305763	2			BRE;RBKS;BRE;BRE;BRE;BRE;LOC100
cg05323345	11			IGF2AS;INS-IGF2;IGF2;IGF2;IGF2AS
cg20694303	4			LOC348926
cg11142248	14			FLJ43390
cg19956605	22			UBE2L3;UBE2L3;UBE2L3
cg22838281	11			TMEM138;TMEM138
cg07236884	12			MIR614
cg15666516	19			RPS11;SNORD35B
cg02719245	14			FLJ45244;DICER1
cg21142158	13			COG6;COG6;COG6
cg18393046	17			MSX2P1
cg16948320	3		rs13097486	HRASLS;MGC2889
cg25504988	6	rs17194824		TRIM39;HCG18;HCG18
cg17819420	19			LOC100131801;LOC100131801;XAB
cg25976804	22			AIFM3;AIFM3;AIFM3;AIFM3;LZTR1
cg16850687	18			DLGAP1;FLJ35776;DLGAP1
cg17819520	5			NCRNA00219;SNORA13
cg14059830	9		rs62578961	C9orf45
cg17747171	20			NOP56;NOP56;SNORD110
cg00106420	12			DIABLO;DIABLO;DIABLO;DIABLO
cg19631845	7			ZNF138;ZNF138;ZNF138;ZNF138
cg02318516	18			LOC400657
cg27586255	7			SDK1;SDK1
cg09275980	15			SNORD116-29
cg01531665	9			NOL6;SUGT1P1;NOL6
cg27607898	6			PECI;PECI;PECI;PECI
cg06579192	19	rs75508674		C19orf56;MORG1;MORG1;MORG1;
cg03747177	16			EARS2;EARS2;UBFD1;EARS2
cg27599211	12			TESC;TESC;TESC
cg06194119	2			TMEM185B
cg11955373	2			TTC31;TTC31;CCDC142
cg05795849	3	rs1866997		ITPR1;ITPR1;ITPR1;EGOT
cg23287902	2			LMAN2L;LMAN2L;LMAN2L;LMAN2L;
cg03719693	4			LOC641518;LOC641518
cg02690648	8			PINX1;MIR1322

cg04145065	4			N4BP2;LOC344967
cg13272108	20			NOP56;SNORD110;NOP56;SNORA51
cg01517832	11			TOLLIP;LOC255512
cg20152539	17			HS3ST3B1;MGC12916
cg00982136	2			CLK1;CLK1;CLK1;CLK1
cg00040423	6			TRIM39;HCG18;HCG18;TRIM39
cg15369601	15			ADPGK;ADPGK;ADPGK
cg27370696	6	rs57301060		AGPAT1;RNF5;RNF5P1
cg16278716	3			KALRN;KALRN;KALRN;KALRN;KALRN
cg10149336	16		rs34142986	EARS2;EARS2
cg14534967	7			ABHD11;ABHD11;ABHD11;ABHD11;[illegible]
cg17597639	5			SDHAP3
cg12265892	19			LOC100134317
cg00404641	3			NUDT16P;NUDT16P
cg20550790	5			FLJ42709;FLJ42709;FLJ42709
cg19945937	14			MIR665
cg11168235	3			MIR548G;C3orf26
cg17462140	11			IGF2AS;INS-IGF2;IGF2;IGF2AS;IGF2
cg05741161	6			LOC285847
cg25228625	17			C17orf44
cg03778895	13			DLEU2
cg00090787	15			RPL4;SNORD16;SNORD18B
cg03113502	6			STL
cg27145044	3			RPL32P3
cg01190037	4			C4orf10;MFSD10;MFSD10
cg05512483	11			SNORD30;SNORD22;SNORD29;SNO[illegible]
cg01899260	11			TMEM41B;TMEM41B;TMEM41B
cg17050100	2			GPN1;CCDC121;GPN1;CCDC121;GP[illegible]
cg03888520	22			RRP7B
cg04500185	3		rs62288198	LOC100128023
cg22498840	9			MCART1;MCART1;MCART1
cg22394119	7			GATS;GATS;GATS;GATS
cg17079757	6			DAXX;DAXX;DAXX;DAXX
cg08014499	11			IGF2AS;INS-IGF2;IGF2;IGF2;IGF2AS
cg12688265	1			GBAP1
cg27168976	1			TXNDC12;BTF3L4;BTF3L4;BTF3L4
cg24931632	1			MGC12982
cg02871659	16			SNHG9;SNORA64;SNORA78;RPS2
cg17875611	3			SNORA7A;RPL32;RPL32;RPL32
cg15883181	17			RNF213;LOC100294362
cg09933836	8			C8orf75
cg05627663	7			SNORA22;CCT6P1
cg09007244	22			RPL23AP82;RABL2B;RABL2B;RABL2[illegible]
cg21722785	17			C17orf44

cg04296434	20			TMEM189;TMEM189-UBE2V1;TMEM
cg05348535	20			HSPC072;LOC100270804;HSPC072
cg17526424	20			SNHG11
cg01033175	3			ZBTB11;LOC100009676
cg19573567	10			ACTA2;FAS;FAS;FAS;FAS;FAS;FAS;FA
cg19231290	10			KLF6;KLF6;KLF6;KLF6;KLF6;KLF6;KLF6
cg22407942	7	rs3834336		SNORD93
cg19088572	1			LOC284632
cg25447652	8			TDH
cg18110419	19			FLJ45445
cg09242901	15	rs1058823		HERC2P2
cg04375784	6			RPS12;SNORD100;SNORA33
cg02395779	3			ZBTB11;LOC100009676
cg10806562	17			FLJ40504
cg25640176	16			GFOD2;GFOD2;GFOD2
cg24936032	5			HINT1;HINT1;HINT1
cg20672430	6			DAXX;DAXX;DAXX;DAXX
cg15119375	16			C16orf67
cg04768630	22			TOM1;TOM1;TOM1;TOM1;TOM1;TO
cg16383389	1			MIR760
cg07052796	10			ASB13;ASB13
cg00039016	16		rs60257999	FBXO31
cg05727186	17		rs631440	MGC57346;MGC57346
cg23620822	3			LOC285375
cg06760162	17			FLJ35220;FLJ35220;FLJ35220;LOC10
cg05804598	15		rs1058828	HERC2P2
cg25851152	10			MIR146B
cg08972632	16			LOC283922
cg00323100	4			N4BP2;LOC344967
cg25211938	22			PANX2;PANX2;PANX2
cg16278124	1			CCNL2;CCNL2;CCNL2;LOC148413;CC
cg05865670	22			THAP7;THAP7;FLJ39582;FLJ39582
cg03230852	20			LOC647979
cg01067809	6			DOM3Z;STK19;STK19;STK19
cg12624825	9			LOC100128076
cg09791459	16			GFOD2;GFOD2;GFOD2
cg20184247	17	rs4395118		LOC404266;LOC404266;LOC404266;
cg15145341	13	rs79470870		LOC646405
cg02824291	7			PAXIP1;LOC202781
cg16567044	14			MEG3;MEG3;MEG3
cg13258700	7			C7orf13;RNF32
cg00459975	8			INTS9;INTS9;HMBOX1;INTS9;HMBOX
cg25446612	2			PP14571;GPC1
cg18042632	21			C21orf130

cg15204036	4			AGXT2L1;AGXT2L1;AGXT2L1;AGXT2L
cg09538129	7			MIR25;MCM7;MCM7;MIR106B;MIR
cg06216926	14			ATXN3;ATXN3;ATXN3;ATXN3;ATXN3
cg26106778	6			RING1;MIR219-1
cg19658046	5			FAM193B;FAM193B
cg23797424	12			LOC642846
cg13253729	22			LOC91316;RGL4
cg26804995	13			TFDP1;TFDP1
cg26813301	16			LOC652276
cg15539395	19			MGC2752
cg19646028	19			MIR7-3;C19orf30
cg11053740	14	rs36066039		RAB2B;TOX4;RAB2B;RAB2B
cg11094161	14			MUDENG;EXOC5;MUDENG
cg09091424	11			ANKRD13D;ANKRD13D
cg17353431	22	rs370030		FLJ39582;THAP7;FLJ39582;THAP7
cg26212163	5			LOC134466
cg02697500	2			CLASP1;CLASP1;CLASP1;RNU4ATAC
cg00996986	22			SERHL
cg18963800	10			HSD17B7P2
cg05640342	11			LOC494141;LOC494141;LOC494141
cg04143736	4			MGC45800
cg26074723	7			RPS2P32
cg04328701	17			FAM134C;FAM134C;TUBG1
cg08258539	8			C8orf51;RHPN1
cg21199854	3			GNL3;GNL3;GNL3;SNORD19B
cg26403608	17			LOC284009;METT10D
cg15583349	6			DAXX;DAXX;DAXX;DAXX
cg15212137	19			MIR519A1
cg11553721	13			TFDP1;TFDP1
cg06808967	3			ACAA1;ACAA1;MYD88;ACAA1
cg15474754	4			HPGD;HPGD;HPGD
cg20737712	11	rs529083		TBRG1;TBRG1;TBRG1
cg09507884	11			LOC100130987
cg22175052	5		rs11540179	SNORD95;GNB2L1;GNB2L1
cg01940867	16			TMEM8A;LOC100134368
cg04662010	2			MTX2;MTX2
cg13505469	10	rs7095041		LOC219347;LOC219347;C10orf57;LC
cg00735591	19			DNM2;DNM2;DNM2;DNM2;MIR638
cg10763638	17			FLJ35220;FLJ35220;FLJ35220;LOC10
cg01243544	1			LOC100130093;LOC100130093
cg12992112	6		rs2028973	CMAH;CMAH
cg17605235	9			ANKRD18A;C9orf122
cg02844341	7			ST7OT4;ST7OT1;ST7;ST7
cg13130934	15			SNORD115-21;SNORD115-20;SNORD

cg13587740	15	rs1800428		HEXA;C15orf34
cg11915671	19			TUBB4;MIR220B
cg14732828	3			FYTTD1;KIAA0226;KIAA0226;FYTTD1
cg00506354	3			ALG3;ALG3;ALG3;ALG3;ECE2;ECE2
cg00941983	6			TJAP1;TJAP1;TJAP1;TJAP1;TJAP1;TJA
cg25574024	11			IGF2AS;INS-IGF2;IGF2;IGF2;IGF2AS
cg18912560	7			LOC100132832
cg11787839	3			LMLN;IQCG;LMLN;LMLN;LMLN
cg02807948	11	rs11510		INS-IGF2;IGF2;IGF2;IGF2
cg25423280	15	rs7174732		PDIA3;CATSPER2P1
cg02145916	8			PVT1
cg19045894	2			ATF2;MIR933
cg16413842	6			C6orf122;C6orf208
cg10822545	19			MIR181D;MIR181C
cg13340272	11			GUCY2E
cg08946731	7	rs56664420		RP9P
cg12029507	7	rs5026069		RNF216L;RNF216L;RNF216L
cg03894789	5			MIR874;KLHL3
cg06850285	22	rs140489	rs5998522	UBE2L3;UBE2L3;UBE2L3
cg00614969	17			SNORA76;SNORD104
cg25695041	4			UBA6;LOC550112
cg17833341	1			SHC1;SHC1;CKS1B;CKS1B
cg26271690	1	rs16850674		LOC148709
cg22793458	3			ZBTB11;LOC100009676
cg05941212	1	rs7418389		MIB2;MIB2;MIB2;MIB2;MIB2;MIB2
cg15982308	6			RNF5P1;RNF5;AGPAT1;AGPAT1
cg01612476	22			MIAT
cg08484337	6			SNORA38;BAT2
cg10608004	19			RPL13AP5;RPL13A
cg25255679	8			RHPN1;C8orf51
cg05659741	1			HIST2H2BA
cg18353837	14			C14orf132
cg06618047	16			LOC146336
cg11080530	14			RAB2B;RAB2B;TOX4;RAB2B
cg03791497	17			TMEM11;TMEM11
cg19273756	17			SKA2;SKA2;MIR301A
cg09393453	19			MIR517B;MIR520G
cg04030905	17			SHBG;SHBG;SAT2
cg16285215	12			FAM60A;FLJ13224;FAM60A;FAM60A
cg22453529	8			C8ORFK29
cg26495109	16			LOC283856;GNAO1;GNAO1;GNAO1;
cg09184043	7			ZNF815
cg13756879	11			INS-IGF2;IGF2AS;IGF2;IGF2;IGF2AS;I
cg11604379	6			HCG9

cg11889478	19			RDH13;RDH13;RDH13;RDH13
cg08247852	8			LOC728024;ERLIN2
cg06296570	7			FLJ43663;FLJ43663
cg08888916	11			SCN4B;SCN4B;SCN4B;SCN4B
cg07201017	10			FLJ41350
cg11904906	2			LOC151174;LOC643387;LOC151174
cg09169617	15			LOC254559
cg09409435	16			PDXDC2
cg14148088	14			MIR494
cg18586095	17			EXOC7;EXOC7;EXOC7;EXOC7;EXOC7;
cg15201877	1			PTGER3;PTGER3;PTGER3;PTGER3;PT
cg16652639	8			TM2D2;ADAM9;TM2D2;ADAM9;TM
cg03594819	11			TIMM8B;SDHD;TIMM8B
cg11111423	6			DAXX;DAXX;DAXX;DAXX
cg13575925	12			LOC144571
cg05793193	7			LOC100128822
cg23002708	19	rs34998465		ATG4D;MIR1238
cg26916936	11			LOC399959;MIR125B1
cg08261702	7	rs3800784		LOC728743
cg01891736	16			LITAF;LITAF;LITAF;LITAF
cg22031629	22			THAP7;THAP7;FLJ39582;FLJ39582
cg11718707	18			MBD2;SNORA37;MBD2
cg24063120	10			FAM178A;FAM178A;FAM178A
cg26577169	6			LOC441177;C6orf176;C6orf176
cg03979284	3	rs71325629		LOC220729
cg17547708	22			LOC100144603;CHKB-CPT1B;CHKB
cg21616552	19			LOC148189
cg02109793	22			C22orf45;C22orf45;UPB1
cg13731761	11			C11orf21;TSPAN32;C11orf21
cg05913233	17			FXR2;SHBG
cg07613278	11			API5;API5;API5;API5
cg25806558	4			N4BP2;LOC344967
cg09154356	1			RPS10P7
cg00476358	2			TUBA4A;TUBA4B
cg08811309	5			LOC645323;LOC645323;LOC645323
cg11209892	7			LOC286016;TNPO3
cg21934311	17			MSX2P1
cg20206277	21		rs34699934	NCRNA00111
cg07007986	14			SERPINA13
cg25705558	15			ISLR2;LOC283731;ISLR2;ISLR2;ISLR2;
cg09639631	6	rs259389		LOC729176;C6orf103
cg08267319	17			LOC92659;MAFG
cg02580195	2			RPL23AP7;RPL23AP7;RPL23AP7;RAB
cg24685761	1			SMG5;TMEM79;TMEM79

cg07491444	2	rs1135617		ARL6IP6;ARL6IP6
cg11537707	1			GAS5;SNORD76;SNORD75;SNORD77
cg24060037	1			SERTAD4;C1orf133
cg06750038	16			FBXO31;MAP1LC3B
cg05975219	16	rs2241038		GAS8;GAS8
cg05241470	15			SLC30A4;C15orf21
cg11621113	19			MORG1;MORG1;MAN2B1
cg00944580	1			DAB1;MIR548D2
cg04444086	16		rs2164339	LOC146336
cg18484665	2			THUMPD2;THUMPD2
cg00283662	19			MIR642;GIPR
cg13343238	15			MIR548H4;GLCE
cg05340865	11			GUCY2E
cg10062290	1			SNORD55;SNORD46;RPS8
cg01551258	17		rs34649800	RHOT1;ARGFXP2;RHOT1;RHOT1
cg10884908	5			PCDHB18
cg22884857	15			PWRN1
cg03144714	12			HOXC4;HOXC5;HOXC5
cg21037184	5	rs6859589		GPR98;GPR98
cg24409539	2			PAX8;PAX8;PAX8;PAX8;PAX8;LOC44
cg01038149	22			P2RX6;P2RX6;MGC16703
cg24642065	12			LOC100190940
cg04160046	19			LOC100129935
cg02371301	11			ANO1;ANO1
cg04103317	9	rs547958		SUGT1P1;SPINK4
cg13855261	17			HS3ST3B1;MGC12916
cg11235787	17			MIR195
cg01827781	16			LOC440356;LOC440356;CDIPT
cg03555433	16			GFOD2;GFOD2;GFOD2
cg07423399	18			CXADRP3
cg08629647	22			GTSE1;CN5H6.4
cg01579841	6			C6orf176;C6orf176;LOC441177
cg02229841	5		rs1047093	PPAP2A;PPAP2A;PPAP2A;RNF138P1
cg15364784	14			DCAF11;DCAF11;DCAF11;DCAF11;D
cg23954461	19			MAN2B1;MORG1;MORG1
cg17025683	1			SNORD78;ZBTB37;SNORD75;ZBTB37
cg13811448	19			MORG1;MORG1;MAN2B1
cg09914581	2			E2F6;E2F6;E2F6;E2F6;E2F6
cg05468584	10			MIR202
cg15703365	7			ZNF815
cg05051957	8	rs58524912		CHRAC1;CHRAC1
cg12691651	15			ADPGK;ADPGK;ADPGK
cg08820497	17			C17orf91;C17orf91;WDR81;C17orf9
cg13440083	11			DKFZp686O24166

cg08767710	7			ATP6V0E2;LOC401431;ATP6V0E2;AT
cg24196814	1			TSNAX-DISC1;TSNAX-DISC1;TSNAX-D
cg01241390	5			RNU5E;RNU5D;ZCCHC9;ZCCHC9;ZCC
cg01477908	22			GSTTP2
cg04970106	6			HLA-DPB2
cg06401019	5			FAM172A;FAM172A;FAM172A;FAM
cg00938929	6			ZNF204P
cg08460812	4			LOC641518;LEF1;LEF1;LEF1;LEF1
cg10844118	14			FLJ31306;ARID4A;FLJ31306;ARID4A;
cg06097557	3			FYTTD1;KIAA0226;FYTTD1;FYTTD1
cg21161173	5			VTRNA1-2
cg14042711	19			SAE1;SAE1;SAE1;SAE1
cg13285968	2			ZNF385B;MIR1258
cg08869031	15	rs76137333		AP3S2;AP3S2
cg16510548	8			LOC401463
cg19862918	17	rs9898532		FAM134C;FAM134C;TUBG1;FAM134
cg11967431	17			LOC440461
cg10068793	21			C21orf34
cg12530503	15			MIR9-3
cg16022748	4			LOC285456;RPL34;RPL34
cg07560681	7			DMTF1;DMTF1;DMTF1;DMTF1;DMT
cg04184179	5			MIR874;KLHL3
cg05382797	6	rs3757333		ZNRD1;ZNRD1;NCRNA00171
cg18382744	5			LOC728411;LOC728411
cg10918927	12	rs985125		HOXC4;HOXC5;HOXC4;HOXC6;HOXC
cg10159607	17			C17orf69;C17orf69
cg10460130	2			DTYMK;DTYMK;DTYMK
cg24616828	7			MIR96
cg07364638	15			NGRN;NGRN
cg25246723	4		rs3796935	LOC285456;RPL34;RPL34
cg17315964	5			FAM193B;FAM193B
cg08508227	20			C20orf166;MIR133A2
cg26144265	7			BZW2;ANKMY2;BZW2;BZW2
cg13404951	12			LOC144742
cg17054054	12			CCDC59;CCDC59;C12orf26
cg02502849	2	rs72969386		CLASP1;CLASP1;CLASP1;RNU4ATAC
cg17994050	6	rs7770557		NCRNA00171;ZNRD1;ZNRD1;ZNRD1
cg25764464	12			PLEKHA5;PLEKHA5
cg09955730	1			TMED5;TMED5;TMED5;CCDC18
cg17666689	10		rs74143906	LOC283050;LOC283050;LOC283050
cg19185384	13			LOC100190939;TPT1
cg03588221	7			MEST;MEST;MESTIT1;MEST
cg16242219	14			DIO3OS
cg04211303	6	rs73414465		HLA-J;NCRNA00171

cg26152485	19			LOC729991-MEF2B;LOC729991-MEF
cg12322132	11			IGF2AS;INS-IGF2;IGF2;IGF2AS
cg23905216	11	rs74050127		INS-IGF2;IGF2AS;IGF2;IGF2;IGF2AS;I
cg21599336	11			LOC100128239
cg11031564	15			SNORD115-15;SNORD115-21;SNORD
cg00953154	7			SNORA9;C7orf40
cg10002564	8	rs35348043		HAS2AS;HAS2
cg05793930	1			GSTM4;GSTM4;GSTM4
cg06686383	7			FAM185A;FAM185A;FAM185A
cg14103106	19	rs35193945		LOC113230
cg12061113	18			LOC100130522;LOC100130522
cg05739816	6			C6orf217
cg03748310	8			KIAA1875
cg11960677	1			MTMR9L
cg10271981	19			MIR1181;CDC37
cg15003194	11			LOC100128239
cg17504698	13	rs9508887		PSPC1;PSPC1;PSPC1
cg09958760	8	rs6991968		TM2D2;TM2D2;ADAM9;ADAM9;TM
cg21024122	6			BAT1;SNORD84;BAT1
cg09422614	19			CACNG8;MIR935
cg06838090	6			LOC154449
cg05732300	21			POFUT2;POFUT2;LOC642852;POFUT
cg09122458	10			BMS1P4
cg25762195	8			RHPN1;C8orf51
cg17093877	17		rs80007821	MGC16275
cg20618109	6			DAXX;DAXX;ZBTB22;DAXX;DAXX
cg01020987	1			C1orf174;LOC100133612
cg00161450	2	rs2422001		LOC388955
cg04299389	20			LOC284798;LOC284798;LOC284798
cg05721281	3			FYTTD1;FYTTD1;FYTTD1;KIAA0226
cg12991050	5			LOC645323
cg24210813	17			LOC404266;LOC404266;LOC404266;
cg11881599	12			CLLU1OS;CLLU1;CLLU1
cg24466873	4			NBLA00301
cg03440944	7			C7orf40
cg01109243	16			NDE1;MIR484;KIAA0430
cg16513467	10			WAC;WAC;WAC;WAC;WAC
cg25890582	10			ZNF487
cg22575379	3			NUDT16P;NUDT16P
cg20659378	11			RNF121;LOC100133315;RNF121;RNI
cg01680573	19			LOC729991-MEF2B;MEF2B;LOC7299
cg02772682	19			SNAPC2;SNAPC2
cg02924425	18			LOC100130522;LOC100130522;PARI
cg21184629	9			C9orf122;ANKRD18A

cg00581541	1			SNORD103A;PUM1;PUM1
cg25391820	5			MATR3;SNHG4
cg19823971	1			PUM1;SNORD103A;PUM1
cg03317826	2			CCT7;CCT7;CCT7;CCT7;CCT7;C2orf7;
cg07439975	6			STL
cg00753676	4			FLJ13197
cg09895527	16			NUDT7;NUDT7
cg11137578	5			RNU5E;RNU5D;ACOT12
cg00491180	16	rs77261105		TMPRSS8
cg24825482	5			NCRNA00219;SNORA13
cg21565150	10			FAS;FAS;FAS;FAS;FAS;ACTA2;ACTA2;
cg01869750	5			TRIM23;C5orf44;TRIM23;C5orf44;TF
cg08981282	16			LOC652276
cg05679027	9			HIATL2
cg16483916	19	rs77608060		PLAC2
cg13791131	11			IGF2AS;INS-IGF2;IGF2;IGF2;IGF2AS
cg00219543	6			C6orf122;C6orf208
cg23214285	7			BZW2;ANKMY2;BZW2;BZW2
cg09638003	15			SNORD115-4
cg02662576	14			MIR495
cg26949796	14			CFL2;CFL2;CFL2;CFL2;CFL2
cg00375691	10			EMX2OS
cg03387256	10			FAS;FAS;FAS;FAS;FAS;ACTA2;ACTA2;
cg14373189	6	rs3130059		BAT1;SNORD84;BAT1
cg01656221	2			MIR548N
cg27283993	10	rs2388864		KLF6;KLF6;KLF6;KLF6
cg06746360	17			RPL23A;SNORD42B
cg00467529	11	rs10892202		IL10RA;IL10RA
cg16630982	17	rs8176075	rs35436937	BRCA1;BRCA1;BRCA1;BRCA1;BRCA1
cg13075363	3			KPNA1;KPNA1
cg18645493	1			MIR190B
cg23636419	16			MAP1LC3B;FBXO31
cg27043582	8			HOOK3;RNF170;RNF170;RNF170;RN
cg02287710	14			DIO3;MIR1247
cg07343367	7			LSM5;LSM5;LSM5;LSM5
cg03505995	14			C14orf23;C14orf23
cg02086493	17			LOC404266;LOC404266;LOC404266;
cg03700287	5			PCDHB19P
cg19275050	8			HAS2;HAS2AS;HAS2
cg06235973	19			LOC113230
cg12346673	10			LOC399815;FAM24B
cg26591684	7	rs7789911		ZNF783
cg18273501	10			CALHM2;CALHM2;CALHM2
cg16652462	17			LOC644172

cg01717517	15		rs3958545	DNM1P35
cg13965908	11			PPFIA1;PPFIA1;MIR548K
cg23050873	2	rs3792109		ATG16L1;ATG16L1;SCARNA5
cg02399652	3			LOC100128164;LOC100128164;SEC6
cg12254291	6			C6orf176;C6orf176;LOC441177
cg03372852	19			LILRP2
cg03794862	7			WNT2;WNT2
cg13112154	10			FLJ41350
cg20612559	17			LOC100306951;INPP5K;INPP5K;INPF
cg00726786	2			MTERFD2;MTERFD2;MTERFD2;MTEI
cg24900244	12			DIABLO;DIABLO;DIABLO;DIABLO
cg21063758	15			ISLR2;ISLR2;LOC283731;ISLR2;ISLR2;
cg03420356	6			LOC730101;LOC730101
cg09976774	12	rs12425068		FOXM1;FOXM1;C12orf32;C12orf32;
cg15236196	17			FLJ32065
cg05665136	5			TRIM23;C5orf44;TRIM23;TRIM23;C5
cg14126688	17			LOC404266;LOC404266;LOC404266;
cg01044849	6			NCRNA00171
cg06150369	3	rs62266955		RPL32P3
cg21643217	7			SDK1;SDK1
cg23278066	11			C11orf73;C11orf73;C11orf73;C11orf
cg14477135	3			LOC100128164;SEC62;LOC10012816
cg01514668	11			MIR129-2
cg10756578	16			MTHFSD;MTHFSD;MTHFSD;MTHFSD
cg05016401	16			LOC440356;LOC440356;CDIPT
cg06014227	12			RPL13AP20
cg09484214	19			MIR372;MIR373
cg19098268	19			ZIM2;ZIM2;PEG3;PEG3;PEG3;PEG3;Z
cg01773692	22			GTSE1;CN5H6.4
cg17212073	17			LOC284023
cg09963123	4			FLJ13197;KLF3
cg06611310	2			KLRAQ1;KLRAQ1;KLRAQ1;KLRAQ1
cg11845785	2			LOC150568
cg07660236	6			ZSCAN12;ZSCAN12
cg07639783	7			PSMG3;KIAA1908;KIAA1908;KIAA19
cg02197629	21	rs76309452		C21orf81
cg02132760	11			LDHA;LDHA;LDHA;LDHA;LDHA;LDHA
cg03689456	12			HNRNPA1;HNRPA1L-2;HNRNPA1;CB
cg13461273	3			ZNF620;ZNF620
cg06738169	19			POP4;POP4
cg14780004	12			NCRNA00173;NCRNA00173
cg05158692	17			PLEKHM1;PLEKHM1;PLEKHM1
cg01606998	22			PANX2;PANX2;PANX2
cg00911551	12			NECAP1;NECAP1

cg09743375	15		rs55653836	JMJD7;JMJD7-PLA2G4B;JMJD7-PLA2
cg26624726	12	rs57594211		NUDT4;NUDT4;NUDT4P1
cg10486879	11			NCRNA00167;PRDM10;PRDM10
cg02486253	17			RNF126P1
cg03606774	2			SLC5A6;SLC5A6
cg04251368	11			KCNQ1DN
cg15057250	1	rs35435329		LOC728448
cg19165105	1			LOC647121
cg01807919	17			SHBG;FXR2
cg18934293	7		rs79538994	MEST;MEST;MEST;MESTIT1
cg07935727	4		rs79508665	LOC340017
cg09010812	16	rs13336336		CENPBD1;AFG3L1;CENPBD1;AFG3L1
cg06136185	17			LOC388428;AATK
cg18365211	4	rs62636595		AFAP1;AFAP1;LOC84740
cg06108374	8		rs74523900	CSGALNACT1;CSGALNACT1;CSGALN
cg09276355	13			C13orf1;C13orf1;C13orf1
cg16937057	7			ST7;ST7OT4;ST7OT1;ST7
cg14306451	5	rs77101434		BASP1;LOC285696
cg09429153	9		rs74704804	PBX3;PBX3;PBX3;PBX3
cg13927290	15		rs364085	GOLGA6L5
cg25936922	2			C2orf60;C2orf60;C2orf47
cg04552737	2			GLB1L;STK16;STK16
cg10916494	6			SNHG5;SNORD50B;SNORD50A
cg12320676	2			E2F6;E2F6;E2F6;E2F6;E2F6
cg20011833	6			LYRM2;LYRM2;LYRM2;LYRM2
cg09341448	19			SEC1
cg06964756	17			RAB34;RAB34;SNORD42B;RAB34;RP
cg09099312	7			Sep-13
cg03143365	19			MAN2B1;MORG1;MORG1
cg15015416	10			FRMPD2;FRMPD2;FRMPD2L1;FRMP
cg23659044	9		rs62581042	TUBBP5
cg25105745	1			MIR197
cg19866040	11	rs35925287		HCCA2;KRTAP5-2;LOC338651
cg06784232	8			CSGALNACT1;CSGALNACT1
cg20847110	4		rs34614751	LOC401127
cg05249271	14			MIR496;MIR154
cg01717031	15	rs397530		HERC2P2
cg01813761	14			PARP2;PARP2;RPPH1
cg19539667	17			LOC100133991;LOC100133991;C17c
cg06023161	6			SNORA38;BAT2
cg12615982	3			TERC
cg06942685	19			ZNF542;ZNF542;ZNF542;ZNF542
cg07064066	21	rs74397289		DSCR8;DSCR8;DSCR8;DSCR4;DSCR4;
cg01769968	1			SNORA42;KIAA0907

cg01787047	11			LOC100130987;RAD9A
cg25356214	12			POU6F1
cg12967093	2			DIRC3
cg27386837	1			NFYC;NFYC;NFYC;LOC100130557;NF
cg10470963	22			ANKRD54;MIR658
cg04036898	1			POMGNT1;POMGNT1
cg00594560	8			ZFHX4;LOC100192378
cg13081644	1			TMEM51;TMEM51;C1orf126;TMEM
cg26508537	10	rs62621207		FAM178A;FAM178A;FAM178A
cg18499530	3			FYTTD1;KIAA0226;FYTTD1;FYTTD1
cg05562080	4		rs2927269	KLF3;FLJ13197
cg12374775	15			SCAND2;SCAND2
cg10153335	17			LOC404266;LOC404266;LOC404266;
cg21545390	7			DLX6AS
cg16251399	6			GUSBL2
cg27293016	10			LOC219347;LOC219347;C10orf57;LC
cg27048959	17			SNORD1C
cg05858136	12			ALKBH2;ALKBH2;ALKBH2;ALKBH2;Al
cg01077764	3			LOC285375
cg21550360	15			ADPGK;ADPGK;ADPGK
cg07745418	19	rs73934853		ZNF83
cg25860143	6	rs28832872		HLA-DRB6
cg06237697	17			MIR365-2
cg14124066	9			RBM18;RBM18;RBM18
cg19826864	10			ZNF438;ZNF438;ZNF438;ZNF438;ZN
cg01986205	4			KLF3;FLJ13197
cg02402630	16			LOC100129637
cg02756451	7			LOC100133091
cg10044179	21			C21orf81
cg00023174	19			ZNF788
cg18384960	14			MIR299;MIR411
cg22530053	3	rs35095647		SOX2OT;SOX2
cg00845626	3			GNL3;GNL3;GNL3;SNORD69
cg12977686	17			SNORA76;SNORD104
cg02276831	9			LOC158381;LOC158381
cg14198101	19			CIRBP;C19orf23;CIRBP;CIRBP
cg04320595	12	rs73278987		RACGAP1P
cg03889876	1			EFCAB2;EFCAB2;EFCAB2;EFCAB2;EF(
cg05541640	3			ABHD14B;ABHD14B;ABHD14A;ABHD
cg22628064	1	rs4345829		RPS10P7
cg10273420	10			ZNF487
cg00630781	6			RGL2;RGL2
cg06954720	6	rs57583084		HLA-DRB6
cg27353919	3			IP6K2;IP6K2;IP6K2;IP6K2;IP6K2;IP6K

cg17226584	7			FLJ43663;FLJ43663
cg16907514	14			KTN1;C14orf33;KTN1;KTN1;KTN1
cg13870510	7			INTS4L1
cg19160209	11	rs7105138		C11orf73;C11orf73;C11orf73;C11orf
cg24265610	10			FLJ41350;LBX1
cg12938003	4			LOC641518;LOC641518
cg20041381	1			C1orf27;C1orf27;MIR548F1;C1orf27
cg27131176	22			C22orf26;LOC150381
cg14946299	7			ZNF273;ZNF273
cg03076771	20			C20orf24;C20orf24;C20orf24
cg16267491	4			LOC256880;H2AFZ
cg19422019	21			C21orf34
cg08599229	7	rs75917398		Sep-13
cg25389463	1			RBM34;RBM34;RBM34
cg21143441	17			C17orf44
cg07894162	22			MORC2;TUG1
cg11919003	13			DLEU1;DLEU2
cg27401698	16			LOC283922
cg02503815	7			DPY19L2P2;DPY19L2P2
cg09980686	2		rs12464121	C2orf52
cg02549834	19			SEC1;NTN5
cg01841828	16			CHTF8;CHTF8;CHTF8;CHTF8;CIRH1A
cg14503796	3			DVWA;CAPN7
cg01308549	21		rs11911729	DSCR9
cg21990144	2			CLK1;CLK1;CLK1;CLK1
cg02771392	7	rs35692753		ZNF273;ZNF273
cg00767560	19	rs16996320		LOC284440
cg03814063	22			C22orf34
cg01800614	6			IFITM4P
cg13173909	2			UGGT1;UGGT1;UGGT1
cg18170076	7		rs78097622	POMZP3;LOC100133091;POMZP3
cg08771706	11			KCNQ1DN
cg18197998	9			PPP2R4;PPP2R4;PPP2R4;CRAT;CRAT
cg09230350	6	rs3180876	rs41267110	STK19
cg09577425	6			PACRG;PACRG;LOC285796;PACRG
cg07216112	15			ISLR2;LOC283731;ISLR2;ISLR2;ISLR2;
cg05618183	16		rs77688126	FUS;FUS;FUS;FUS
cg18780276	2			TMEM150A;TMEM150A;TMEM150A
cg18061847	8			HAS2;HAS2AS
cg03855656	22			SERHL
cg04045419	10	rs35136724		WAC;WAC;WAC
cg21858113	11		rs57371658	SCN4B;SCN4B;SCN4B;SCN4B
cg10275180	20	rs1138756		FRG1B
cg16893174	17			ALOX12P2

cg14641774	21		rs13046079	C21orf49;C21orf49
cg10766021	7			ZNF783
cg07873320	6			C6orf227
cg15482500	11			MIR210
cg21481141	19			CIRBP;C19orf23;CIRBP;CIRBP
cg04513422	13			C13orf29
cg13402773	19			SEC1;DBP
cg01405107	17			LOC404266;LOC404266;LOC404266;
cg13340335	11			LOC100128239
cg07490142	22	rs79301878		FLJ39582;THAP7;FLJ39582;THAP7
cg20086597	1			LOC731275
cg16649915	1			OR2M1P
cg27519555	1	rs16863397		GORAB;GORAB;GORAB
cg01675238	15			JMJD7-PLA2G4B;JMJD7;JMJD7-PLA2
cg23181861	19			RPL18AP3;RPL18A
cg17038116	1			ASH1L;LOC645676
cg17050693	17	rs12603630		GHDC;GHDC;GHDC;GHDC;GHDC;GH
cg12768770	1			SNORD55;SNORD46;RPS8
cg21406217	8			INTS9;INTS9;HMBOX1;HMBOX1;INT
cg19429740	11			RNF121;RNF121;LOC100133315;RN
cg22202401	15	rs78163310		CSNK1A1P
cg25724246	3			SNAR-I
cg09114153	11			C11orf21;TSPAN32;C11orf21
cg00832101	20			SNHG11;SNORA39
cg09526697	4			LOC348926
cg21112719	3			ZNF620;ZNF620
cg03177025	2			BRE;BRE;BRE;BRE;RBKS;BRE;LOC100
cg18954434	22			CN5H6.4;GTSE1
cg20962137	3			COL29A1;COL29A1;COL29A1
cg13078388	19			CIRBP;C19orf23;CIRBP;CIRBP
cg20309061	15			CAPN3;CAPN3;CAPN3;CAPN3;CAPN
cg12446939	1			ASH1L;MIR555
cg02178774	19			LOC729991;RFXANK;LOC729991-ME
cg18076500	19			ECSIT;ECSIT;ECSIT;ECSIT
cg09650667	3			COPB2;COPB2
cg07926598	7			SDK1;SDK1
cg12950665	7			SDK1;SDK1
cg27416261	9			LOC158381;LOC158381
cg21812670	1			SNORD45C;RABGGTB
cg14793891	2			GLB1L;STK16;GLB1L;STK16
cg18254356	5	rs534655		LOC153684
cg21075870	1		rs605506	MSTO2P
cg10497951	11			LOC100133315
cg07988989	2			TMEM185B

cg03654106	19	rs62126040		SNAR-G1;CGB1
cg19152770	4			LOC84740
cg20498895	3			SNORA7A;RPL32;RPL32;RPL32
cg23506561	15	rs3825768		SNORD116-20;SNORD116-21
cg12047086	2			LOC151174;LOC151174
cg25271404	15	rs6495689		GOLGA8B
cg06788790	17			RNF126P1
cg24148841	6			CDYL;CDYL;CDYL;CDYL
cg11227237	6	rs73439096		C6orf52;PAK1IP1;C6orf52;C6orf52
cg18588811	14			MIR377
cg04117643	17			EIF4A1;SNORA48
cg16621560	13			MIR548F5;DCLK1
cg21565017	3			C3orf49
cg05329135	13			LOC121952
cg22142142	15			GABARAPL3
cg06781135	6			LOC100270746;C6orf41
cg04374231	1			LOC647121
cg09083108	6			PACRG;PACRG;LOC285796;PACRG
cg09410084	1			ASH1L;LOC645676
cg25719236	6			LYRM2;LYRM2;LYRM2;LYRM2
cg15987431	1		rs61740910	MIR1182;FAM89A
cg03549146	16			MIR140;WWP2;WWP2
cg09831207	2		rs71410494	PRDXDD1P
cg21857017	2			LOC100133985
cg23684682	19			ZNF30;ZNF30;ZNF30;ZNF30
cg07959070	22			C22orf34
cg26178446	2			OBFC2A;OBFC2A
cg27617225	8			CPSF1;MIR1234
cg23068797	19			DNM2;MIR199A1;DNM2;DNM2;DNI
cg21538216	22			PARVG;PARVG;PARVG;PARVG;PARV
cg11933757	17	rs11540320		NCRNA00188;NCRNA00188;NCRNA(
cg19844955	15			SNORD116-15;SNORD116-16
cg26111518	17			GHDC;GHDC;GHDC;GHDC
cg16032841	13	rs2277425		C13orf29
cg19255853	4			LOC93622
cg13663218	12			LOC283392;TRHDE;LOC283392
cg06266993	17			LOC404266;LOC404266;LOC404266;
cg05891181	6	rs72545987		HCG27
cg01965047	16			PKD1;MIR1225;PKD1
cg16204151	19	rs60314070		MIR523
cg18923803	1			FLVCR1;LQK1;LQK1
cg02139796	6	rs2229049		LOC442245
cg01508796	10			C10orf75
cg20731875	17			HS3ST3B1;MGC12916

cg01928553	7			ATP6V0E2;ATP6V0E2;LOC401431
cg09815638	7	rs73361998		CCT6P1
cg10592710	3			COL29A1;COL29A1
cg03724640	12			CD27;LOC678655
cg24576225	13			LOC100190939;TPT1
cg26560928	7			MKLN1;FLJ43663
cg00884680	3			ACAA1;ACAA1;ACAA1;MYD88;ACAA
cg05582165	3			C3orf17;C3orf17;C3orf17
cg14188650	15			SNORD115-8
cg13374648	19			PEG3;PEG3;ZIM2;ZIM2;PEG3;ZIM2;N
cg25588635	7			ZNF273;ZNF273
cg01638101	2			LOC100189589
cg26120756	8			TATDN1;TATDN1;TATDN1;NDUFB9
cg18843682	12			HOXC4;HOXC5
cg25196296	8	rs13274853	rs5024323	FAM66A
cg21648425	2	rs36226881		CD8A;CD8A;CD8A;CD8A
cg10763594	2		rs78470215	DIRC3
cg04112787	22		rs13053481	psiTPTE22
cg16201596	6	rs9261106		HLA-J;NCRNA00171
cg11456756	6			HLA-L
cg06752340	21	rs11910504		C21orf49;C21orf66;C21orf66;C21orf
cg00407150	3			HHATL;HHATL
cg14421309	2			ZEB2;ZEB2;ZEB2;ZEB2;ZEB2
cg00610228	6		rs35168508	FKBP5;LOC285847
cg25945303	10			KLF6;KLF6;KLF6;KLF6
cg15149095	1			SYT14;SYT14;SYT14;SYT14;SYT14;SY
cg05767306	3			AMT;AMT;AMT;AMT;AMT
cg14425733	22			LOC150381;C22orf26
cg07573279	11			LOC645332
cg01358993	5			LOC645323;LOC645323;LOC645323
cg07095783	10			ZEB1;ZEB1;ZEB1;ZEB1;ZEB1;ZEB1
cg27331871	11			INS-IGF2;IGF2;INS-IGF2
cg20104055	3	rs77327593		LSM3;XPC;XPC;XPC
cg13697715	5			LOC202181
cg01273384	20			MIR1257
cg11905407	11			CASP5;CASP5;CASP5;CASP5;CASP5;C
cg01348570	4			UBA6;LOC550112
cg23108728	2			TUBA4B;TUBA4A
cg11517045	2	rs59694669		MTHFD2;MTHFD2
cg01472299	6			CDYL
cg11740927	20			LOC643406
cg05919625	18		rs73426565	ZNF271;ZNF397OS;ZNF271;ZNF397C
cg15654127	7			POT1;POT1;POT1;POT1;POT1;POT1;
cg05776603	14			CFL2;CFL2;CFL2;CFL2;CFL2

cg13547299	11			TOLLIP;LOC255512
cg03136625	6			C6orf48;C6orf48;C6orf48;SNORD48;
cg10716163	19			MUM1;MUM1
cg03955764	10			LOC100133308
cg17354052	16			UBFD1;EARS2;EARS2
cg10715527	8			ANK1;MIR486;ANK1;ANK1;ANK1;AN
cg11783815	6			C6orf227
cg09141338	17			FXR2;SHBG
cg01311102	9			PTGES2;PTGES2;PTGES2
cg25064146	11	rs12789541		TIMM8B;SDHD;TIMM8B
cg02649063	16	rs7200011		CES4
cg17504991	6			DAXX;DAXX;DAXX;DAXX
cg20266104	17		rs9901071	TMEM11;TMEM11
cg23572163	22	rs58514871		psiTPTE22
cg14814323	11			SMPD1;SMPD1;SMPD1
cg25877179	17			SNORD42B;RPL23A
cg02593924	16			IGFALS;IGFALS;IGFALS
cg24823137	19			MIR372;MIR371;MIR373
cg01322165	2	rs11556398		TRIB2;TRIB2;TRIB2
cg18311708	7			ZNF767;ZNF767
cg08884624	2			MYCNOS;MYCN
cg04188086	1			FLJ37453;SPEN
cg04288299	4			WHSC2;MIR943
cg25012898	1			TMEM79;SMG5
cg04627849	22			POM121L1P
cg05774978	1	rs1053423		PDIA3P
cg03693749	6			HLA-DPB2
cg07314523	17			AFMID;AFMID;AFMID;TK1;AFMID
cg26053291	2	rs34513596		FLJ40330
cg14821382	2			C2orf14
cg01852272	19			LOC284440
cg08264885	8		rs78882264	POLR3D;MIR320A
cg06545143	6			LOC441177;C6orf176;C6orf176
cg21525314	7			DPY19L2P2;DPY19L2P2
cg08839210	17			RAB34;RAB34;RAB34;RAB34;RAB34;
cg01229567	1			MIB2;MIB2;MIB2;MIB2;MIB2;MIB2
cg07527324	14			C14orf159;C14orf159;C14orf159;SN
cg10884885	1			LOC149134
cg05492270	11			RNF121;LOC100133315;RNF121;RNI
cg07940593	22			RPL3;SNORD83B;RPL3
cg16403299	1			C1orf97
cg13206706	1			RPL5;SNORD21
cg15837838	17		rs59489829	CCDC144C
cg16385939	1			LOC728875

cg09242100	19	rs35441287		ZNF525
cg22725901	19			MIR518C
cg24059871	19			POP4;POP4
cg07187233	1			DISC1;DISC1;TSNAX-DISC1;DISC1;DIS
cg06317622	16			GFOD2;GFOD2;GFOD2
cg07529750	2	rs72492115		LOC654342
cg08165151	6			NCRNA00120;AKIRIN2
cg25776856	7	rs55859862		SLC26A4;LOC286002
cg22772180	13			PSPC1;PSPC1
cg18981338	14			DHRS4;C14orf167;C14orf167;C14orf
cg23522832	1			LOC400752
cg16638920	5			LOC645323;LOC645323
cg09663204	5			LOC100129716;ARRDC3
cg01521378	6			NCRNA00171
cg15719339	21	rs75623773		C21orf34;C21orf34
cg06906869	13			NEK3;NEK3;NEK3;NEK3
cg00261534	10			KLF6;KLF6;KLF6;KLF6
cg12486710	1			C1orf220;C1orf220
cg18366455	3			SOX2OT
cg13959647	9			PSMD5;LOC253039
cg04992930	5	rs16880430		BRD9;BRD9;BRD9
cg08423149	7			RNF216L;RNF216L;RNF216L
cg20653009	17	rs73301318		LOC284100
cg04402891	20			NCRNA00029
cg21532432	11			IGF2AS;INS-IGF2;IGF2;IGF2AS
cg12119029	16			CDK10;CDK10;CDK10;CDK10;CDK10;
cg11867420	7			STAG3L4;PMS2L4
cg07927379	7		rs74424274	C7orf13;RNF32
cg21287936	19			ZNF667;ZNF667
cg19678029	11			KCNQ1OT1;KCNQ1;KCNQ1
cg16635550	6			STK19;STK19;DOM3Z;STK19
cg07218344	13			TRIM13;TRIM13;TRIM13;TRIM13;TR
cg26137121	8			C8ORFK29
cg17539315	12			LARP4;LARP4;LARP4;LARP4;LARP4;L
cg15164573	17			LOC100128288
cg12528452	11			INS-IGF2;IGF2;IGF2;IGF2
cg05308293	7			RPS2P32
cg26039305	11			MIR670
cg14257107	1		rs71664096	GNRHR2;RBM8A
cg07987890	15			MIR548H4
cg00808170	5			PCDHGA4;PCDHGA11;PCDHGA11;PC
cg12503971	12			LOC100240735;FLJ12825
cg24874705	15			LOC645752
cg00543443	20			LOC647979

cg05173737	14			FLJ43390
cg10432093	6			HCG9
cg04915414	1			SHC1;SHC1;CKS1B;CKS1B
cg05641843	17		rs11552638	EIF4A1;SNORA48
cg24844423	19			PEG3;PEG3;ZIM2;ZIM2;PEG3;ZIM2;N
cg03367679	17			MED24;MED24;SNORD124
cg01820463	2			FTHL3;PPM1G;PPM1G
cg27611263	2			THUMPD2;THUMPD2
cg19660618	12			LOC100286844;NCKAP5L;NCKAP5L;L
cg01105073	11			PGAP2;PGAP2;PGAP2;PGAP2;PGAP2
cg01630479	22			MIR1306;DGCR8
cg03082779	20			ZMYND8;LOC100131496;ZMYND8;Z
cg09281979	2			DCAF17;METTL8;DCAF17;DCAF17
cg21633052	4			C4orf38
cg25576352	19			ECSIT;ECSIT;ECSIT;ECSIT
cg09916234	4			WHSC1;WHSC1;WHSC1;SCARNA22;W
cg17339147	7			WNT2;WNT2
cg07273415	11			TMEM138;CYBASC3;CYBASC3;TMEM
cg14485809	10			FLJ41350;LBX1
cg13500842	19		rs76389034	GYS1;GYS1;GYS1
cg03332810	10			PPP2R2D;PPP2R2D;PPP2R2D
cg02659673	1			LOC100133612;C1orf174
cg22619824	7			ST7;ST7;ST7OT2
cg08408453	11	rs34193719		PDE2A;PDE2A;PDE2A;PDE2A
cg18997137	13			FLJ37307;FLJ37307
cg11136251	15			ZWILCH;ZWILCH;RPL4
cg01027728	5			LOC255167;LOC255167
cg19843451	1		rs61437216	LOC400752
cg05697832	7			SDK1;SDK1
cg07220782	6			LOC100270746;C6orf41
cg16579431	16			TMEM8A;LOC100134368
cg24051554	1			LOC100132111;C2CD4D
cg17774634	6			C6orf167;MIR548H3
cg25114075	3			CEP63;ANAPC13;CEP63;ANAPC13;CE
cg05175337	5			RNU5E;RNU5D;ZCCHC9;ZCCHC9;ZCC
cg04419707	22	rs17555272		RPL3;SNORD43;RPL3
cg22358276	6			C6orf59;AGPAT4
cg26702985	10			ZNF37B
cg03964851	5		rs77830550	MIR1974;C5orf36;C5orf36
cg09872104	7			C7orf49;C7orf49;C7orf49;C7orf49
cg04144321	12			NCRNA00173;NCRNA00173
cg09559971	16			TRAF7;SNORD60
cg16424519	16			SLC7A5P2
cg08306955	6			CMAH;CMAH

cg03562868	6			PLAGL1;PLAGL1;PLAGL1;PLAGL1;PLA
cg24255269	3			PISRT1
cg18002437	1			LOC728875
cg06158985	3			C3orf74
cg18598029	6			HCG27
cg12339904	22			RFPL1S;RFPL1
cg13135459	10			LOC387646
cg18162350	1			PABPC4;PABPC4;PABPC4;SNORA55
cg12110333	5	rs72719206		LOC728613
cg07553475	6			FLJ22536
cg21482745	2			NEURL3
cg17201760	4			ZNF876P
cg21871203	3			KCNMB3;KCNMB3;KCNMB3;KCNMB
cg02232751	11			LDHA;LDHA;LDHA;LDHA;LDHA
cg11379605	1	rs77252600		CD58;CD58;CD58
cg07604732	16			LOC728276
cg20996579	14			FLJ31306;FLJ31306;ARID4A;ARID4A;
cg23435915	5			LOC153684
cg27333886	19			SAE1;SAE1;SAE1;SAE1
cg03043822	22			TOP1P2;PIWIL3
cg14753458	3			IP6K2;IP6K2;IP6K2;IP6K2;IP6K2;IP6K
cg08855729	12			H2AFJ;H2AFJ
cg02585417	12			MIR618;LIN7A
cg00097536	2	rs71707059		ZFP36L2;LOC100129726
cg02596499	7			FLJ43663;FLJ43663
cg13305186	11			TMX2;TMX2;TMX2;C11orf31
cg06688014	10			XPNPEP1;XPNPEP1;XPNPEP1
cg23718418	6			PHF1;PHF1;PHF1
cg15772366	6			TRAF3IP2;TRAF3IP2;TRAF3IP2
cg26550938	1		rs74678936	LOC100133612
cg03047554	14			THTPA;THTPA;THTPA;THTPA;THTPA
cg22705386	7			MEST;MEST;MEST;MESTIT1
cg08639244	19			ATP5SL;ATP5SL;ATP5SL;ATP5SL;ATP5
cg05691383	9			LOC340508
cg26469244	15			SNORD115-21;SNORD115-20;SNORD
cg13858747	11	rs5743851		LOC255512;TOLLIP
cg13553147	1			SNORD38B;RPS8
cg22369818	15			GABPB1;GABPB1;GABPB1;FLJ10038;
cg00786256	6			C6orf147
cg09218524	15			LOC283663
cg08049736	6			HLA-L
cg24245352	22	rs3986050	rs3986049	RNF185;RNF185;RNF185;RNF185;RN
cg22178096	3			RPL32P3
cg21417675	7			LOC286016;TNPO3;TNPO3

cg15679153	3			LSM3;XPC;XPC;XPC;LSM3
cg14048487	17			BRCA1;BRCA1;BRCA1;BRCA1;BRCA1
cg04925196	12			POU6F1;POU6F1
cg15691003	1			HSPC157;HSPC157
cg22065500	7			TNPO3;LOC286016
cg25477839	8			ZNF252;C8orf77
cg07655479	4			LOC441046
cg09318162	6	rs77813053		PPP1R2P1
cg14014525	17			G6PC3;G6PC3;G6PC3
cg20121513	6			GUSBL2
cg06083642	7			CHPF2;MIR671
cg07960084	17			TMEM11;TMEM11;TMEM11
cg25268451	20			GNASAS;GNAS
cg08969198	22	rs9623641		RNU12;POLDIP3;POLDIP3
cg02606403	2			LOC100132215
cg05670472	5			PCDHGA4;PCDHGA11;PCDHGA11;P(
cg27395922	11	rs58491120		LOC441601
cg26988585	17			CDRT15P
cg07324381	9			GPSM1;GPSM1;LOC26102
cg00074348	11			APLNR;APLNR
cg12212101	7			RP9P
cg24087783	1			TRNAU1AP;TRNAU1AP
cg00450487	8			ZFAND1;ZFAND1;ZFAND1;ZFAND1;Z
cg10205287	4			MAPKSP1;MAPKSP1;MAPKSP1
cg27490387	6			HCG11
cg09400566	19		rs11555033	C19orf23;CIRBP;CIRBP;CIRBP
cg12007399	19			ZFR2;ZFR2;ZFR2
cg21019820	3			MIR191;NDUFAF3;NDUFAF3;NDUFA
cg05137443	6	rs17221401		HLA-DPB2
cg27592126	17			RPL23;SNORA21
cg24155748	7			POLR2J4
cg02975060	14			MIR1185-2
cg20177310	2		rs56254527	ZEB2;ZEB2;ZEB2
cg16970804	5			LOC153684
cg07800806	22			LOC91316
cg00346898	1			TSNAX-DISC1;DISC1;DISC1;DISC1;DIS
cg25707676	7			DLX6AS
cg16745104	20			MIR941-1;MIR941-3;MIR941-2;MIR9
cg25361850	19			ZNF577;ZNF577;ZNF577
cg05285228	7			POT1;POT1;POT1;POT1;POT1
cg12930819	2	rs6751132		LMAN2L;LMAN2L;LMAN2L;LMAN2L;
cg26856583	16	rs74605057		MAP1LC3B;FBXO31;MAP1LC3B
cg19537184	19			MIR520C;MIR526A1
cg10145584	11			IL10RA;IL10RA

cg26590411	4			SNORA24;SNHG8
cg01254948	5			GPR98;GPR98
cg26361313	10			LOC282997;PDCD4;PDCD4
cg06905762	5	rs1279736		GNB2L1;SNORD95
cg02228481	6	rs3130463		HCG27
cg02967546	17			C17orf91;C17orf91;WDR81;C17orf9
cg20335185	1			MSTO2P
cg16251576	12	rs4842677		LOC338758
cg10836887	5			LOC645323
cg14550519	17			LOC653653
cg09661284	5		rs41285575	GNB2L1;SNORD95
cg17274400	12			NECAP1;NECAP1
cg08737296	20			MIR124-3
cg15168906	11			IGF2AS;INS-IGF2;IGF2;IGF2;IGF2AS
cg24753148	11			APLP2;APLP2;APLP2;APLP2;APLP2;A
cg02774282	6			CCDC162
cg13038025	15			JMJD7-PLA2G4B;JMJD7-PLA2G4B;PL
cg04155485	7			MIR589;FBXL18
cg17109533	19			MIR515-2;MIR515-1
cg10036368	6			LOC285796
cg05352541	22			PITPNB;LOC284900;LOC284900
cg09758490	4	rs74322951		LOC100130872;LOC100130872-SPO
cg07618928	2	rs2241879		ATG16L1;SCARNA5;ATG16L1
cg12654189	7			LOC100134713;NDUFB2
cg02627887	5			LOC645323
cg01462607	19			NCRNA00085
cg26257814	19			FLJ26850
cg14051842	6			LOC100270746;C6orf41
cg12959622	20			SNORD12B;C20orf199;SNORD12C;M
cg13197733	7	rs11983460		FAM185A;FAM185A;FAM185A
cg08066497	7			SLC25A13;SLC25A13;SLC25A13
cg14113010	5			GNB2L1;SNORD95
cg10245314	1			UBE2J2;UBE2J2;LOC100128842;UBE
cg04107539	7	rs1128461		TP53TG1
cg01537486	11		rs11558674	SNORD27;SNORD26;SNORD29;SNOR
cg06627801	4			WHSC1;WHSC1;WHSC1;SCARNA22;\
cg22420044	7			MCM7;MCM7;MIR25
cg15379025	21			C21orf82;C21orf82
cg10706100	6			LOC441177;C6orf176;C6orf176
cg16539629	14			C14orf132
cg01572694	17			MIR10A
cg14903612	10			LOC283050;LOC283050;LOC283050
cg04927033	17			LOC644172
cg16922466	6			DAXX;DAXX;DAXX;DAXX

cg19603557	17			COX11;COX11;COX11;COX11;STXBP4
cg19695521	6	rs6906180		HCG18;HCG18
cg07422345	19	rs41428545		LOC80054;CEBPA
cg07953034	19			ECSIT;ECSIT;ECSIT;ECSIT;ECSIT;ECSIT
cg00147751	7			GATS;GATS;GATS;GATS;PVRIG
cg16572540	19			MIR24-2
cg11608241	8	rs35229339		FLJ10661;FLJ10661;FLJ10661
cg24260710	22			MIR33A;SREBF2
cg01521220	17	rs77574787		MIR1203;SKAP1;SKAP1
cg26143338	5	rs35952611		SDHAP3
cg00431236	2			ACP1;SH3YL1;ACP1;ACP1;ACP1;SH3Y
cg08229366	7			MESTIT1;MEST;MEST;MEST
cg01520105	6			TRAF3IP2;TRAF3IP2;TRAF3IP2;TRAF3
cg07242427	6			MGC26597
cg07116997	7			HOXA11AS;HOXA11
cg07788369	7	rs34413484		DLX6AS
cg16357834	4			MGC45800
cg11700800	5			FLJ42709;FLJ42709;FLJ42709
cg15157453	1	rs71763394	rs80107141	POMGNT1;POMGNT1
cg22537343	19			MIR525
cg22658229	13			SUGT1L1
cg15062711	1			TMED5;CCDC18;TMED5;TMED5;CCD
cg07516483	8			LOC100192378
cg19981839	16			CD2BP2;CD2BP2
cg23942884	6			PPP1R2P1
cg24195169	7			LOC349114
cg06969395	5	rs62387132		LOC100133050
cg11891983	18			RPL17;SNORD58A;SNORD58B;RPL17
cg05055326	6			HCG9
cg14282222	7	rs62481876		C7orf49;C7orf49;C7orf49
cg01983504	20			PANK2;MIR103-2;PANK2;PANK2
cg04255278	5			FLJ42709;FLJ42709;NR2F1
cg12469954	11			LOC143666;PHRF1
cg03892356	12	rs74089890		HOXC4;HOXC5;HOXC6;HOXC6
cg09060275	22			MIAT
cg16847051	12			CALCOCO1;CALCOCO1;CALCOCO1
cg05115468	11	rs2269353		SNORD27;SLC3A2;SLC3A2;SNORD26
cg11688874	10			WAC;WAC;WAC;WAC;WAC
cg17594424	15	rs11555369	rs1049531	MIR1282;SERF2
cg08754067	5			SNORD72;RPL37
cg15364169	6	rs6939616		HLA-J;NCRNA00171
cg16391783	15	rs78774742		C15orf34;HEXA
cg11032038	16		rs80101105	MIR1826
cg05988158	22			CHKB-CPT1B;CHKB;LOC100144603;C

cg04516152	9	rs2275157		SNHG7;SNORA17;SNHG7;SNHG7
cg05821634	1	rs77695076		FLJ37453
cg18563153	8			PVT1
cg04543156	10			ATAD1;ATAD1;CFLP1
cg00661523	3			MBNL1;MBNL1;LOC401093;LOC401(
cg26534477	17	rs35067771		G6PC3;G6PC3;G6PC3
cg13997013	2			LOC285074
cg10972625	1			LOC100286793;FLJ39739
cg02484047	19			MORG1;MORG1;MAN2B1
cg20521277	8			C8orf75
cg11128021	5			RNU5E;RNU5D
cg20523169	17			FLJ45079
cg19269520	17			LOC90586
cg12296644	1			GNRHR2;PEX11B
cg14123427	14			MEG3;MEG3;MEG3
cg06559318	6	rs28679786		HLA-DRB6
cg08775774	12			CCDC62;CCDC62;CCDC62
cg00537203	17			LOC92659;MAFG
cg01605984	9			SNORD24;SNORD36B;MED22;RPL7A
cg01250961	10			EMX2OS
cg09278885	1		rs12034613	C1orf86;LOC100128003;C1orf86
cg10281852	8	rs35229339		FLJ10661;FLJ10661;FLJ10661
cg15869022	2			GPR17;GPR17;LIMS2;LIMS2;LIMS2;G
cg09615909	6			DAXX;DAXX;DAXX;DAXX
cg07895657	22			PANX2;PANX2;PANX2
cg14245347	6		rs2523801	HLA-H
cg18224077	11			LOC399959
cg06324048	20	rs6026561		GNAS;GNAS;GNAS;GNASAS

UCSC_RefGene_Accession	UCSC_RefGene_Group	Relation_to_I	DMR
NR_027023;NM_018489	TSS1500;5'UTR	N_Shore	
NR_003667;NM_016410;NM_	Body;TSS200;TSS200	Island	
NR_024469	Body	Island	
NR_004407	TSS200		
NR_028044;NR_003512;NM_	Body;Body;5'UTR;Body	S_Shore	
NR_027239;NM_032825;NM_	TSS200;5'UTR;TSS200;TSS200	Island	
NR_026936	Body		
NR_003713	TSS200	Island	
NM_201402;NR_027425	TSS1500;Body		
NM_001099289;NR_029193	TSS1500;Body	Island	DMR
NR_024431;NR_024429;NR_(	Body;Body;Body	Island	
NM_032271;NR_002736	TSS1500;TSS200	N_Shore	
NM_001656;NM_001093755	5'UTR;TSS1500;5'UTR;TSS150	Island	
NR_003504	Body	Island	
NR_024282	TSS1500	Island	
NR_027822	Body	Island	DMR
NR_027092;NR_027093;NR_(	Body;TSS1500;Body	Island	
NM_053023;NM_001143995	TSS1500;TSS200;TSS1500	Island	
NR_028444;NM_006810	TSS1500;TSS1500	N_Shore	
NR_026826;NM_001145159;	TSS1500;TSS1500;5'UTR;1stE:	Island	
NM_005510;NM_004197;NM	5'UTR;1stExon;1stExon;1stEx	Island	
NR_026564;NR_026541;NR_(	TSS200;TSS200;TSS1500	Island	
NR_015427	TSS1500	N_Shore	
NR_027822	TSS1500	N_Shore	
NM_002136;NR_002944;NM_	Body;Body;Body	S_Shore	
NR_002795	Body	N_Shore	RDMR
NM_004375;NR_027942;NR_	1stExon;Body;Body;1stExon;1	Island	
NR_024167;NR_024166;NM_	TSS200;TSS200;Body;Body	N_Shelf	
NR_015379	TSS1500		
NM_013417;NM_002161;NR_	5'UTR;5'UTR;TSS1500;1stExon	Island	
NR_015432	TSS200	Island	
NM_032470;NM_032470;NR_	5'UTR;1stExon;TSS200;TSS1500;TSS1500		
NM_000994;NR_002582;NM_	5'UTR;TSS1500;TSS1500;1stE:	Island	
NR_003512;NM_001127598;	Body;Body;Body;Body	Island	
NM_173627;NR_029376;NM_	Body;TSS200;Body;Body	Island	
NM_007300;NR_027676;NM_	5'UTR;Body;5'UTR;TSS1500;5	N_Shore	
NM_052879;NM_001170803	Body;Body;Body;Body;Body;B	S_Shore	
NM_001170687;NM_001170	TSS1500;TSS1500;TSS1500;TS	N_Shore	
NM_177967;NM_001144072	5'UTR;Body;Body;1stExon	Island	
NR_015402;NR_024141;NM_	Body;Body;TSS200;Body;TSS1	Island	
NR_002728;NM_000218;NM_	TSS1500;Body;Body	S_Shore	
NR_002818	TSS200	N_Shore	
NM_139131;NM_001145439	5'UTR;TSS1500;5'UTR;TSS150	N_Shore	RDMR

NR_003662	TSS200	N_Shore	
NM_001142273;NM_015282	Body;Body;Body;Body	S_Shore	
NM_145352;NR_028075;NM_	Body;Body;Body;Body;Body	Island	
NM_001170687;NM_001170	Body;Body;Body;5'UTR;Body;	Island	
NM_001166284;NR_029402;	TSS1500;TSS1500;TSS1500;TS	Island	
NR_003512;NM_001007139;	Body;3'UTR;3'UTR;3'UTR	Island	
NR_033231;NR_033230;NM_	TSS200;TSS200;TSS200;TSS20	Island	
NR_027397;NM_001146039;	TSS1500;TSS1500;TSS1500		
NR_031679;NM_198468	Body;TSS1500	S_Shore	
NR_024181;NM_032679;NM_	TSS200;TSS200;TSS200	Island	
NR_015358	Body	S_Shore	
NR_026791	Body	Island	
NR_024625;NM_001142931;	TSS1500;TSS1500;TSS1500;TS	N_Shore	
NR_002819	TSS200	Island	
NR_026875	Body	N_Shore	
NR_028514;NM_130766;NM_	TSS1500;5'UTR;5'UTR;Body	N_Shore	
NM_014415;NR_024407	1stExon;Body	Island	
NM_032846;NR_028074;NM_	Body;Body;TSS1500;5'UTR;1s	N_Shore	
NM_001143979;NR_030159;	TSS1500;TSS1500;5'UTR		
NM_021253;NR_024052;NR_	TSS1500;Body;Body;TSS1500	N_Shore	
NR_003584;NR_002963	Body;TSS200	S_Shore	
NR_026682;NM_016093;NR_	Body;TSS200;TSS1500	Island	
NR_024421;NM_032772;NR_	Body;TSS1500;TSS1500	Island	
NR_003044;NR_003038;NR_(	TSS200;Body;TSS1500	N_Shore	
NM_014596;NM_170783;NR	TSS1500;TSS1500;Body	N_Shore	
NM_173631;NR_002166	TSS1500;TSS1500	N_Shore	DMR
NR_029435;NR_029434;NM_	Body;Body;TSS1500;TSS1500	N_Shore	
NM_001014444;NR_026675	5'UTR;Body	Island	
NR_024084;NM_024632;NM_	TSS1500;TSS1500;TSS1500;TS	N_Shore	
NR_024240;NR_026751	TSS1500;Body	N_Shore	
NR_027416;NM_152347	Body;Body	Island	
NR_024378	TSS1500		
NR_015454;NM_002359	TSS200;TSS200	Island	
NR_027130	TSS200		
NM_001130001;NM_001099	TSS1500;TSS1500;TSS1500;TS	Island	
NR_027635;NM_024063	TSS200;TSS200	N_Shore	
NR_027073;NM_020851;NM_	Body;TSS1500;TSS200;TSS15(	Island	
NR_028301;NR_028302;NR_(	Body;Body;TSS1500	Island	DMR
NR_026703	TSS200		
NR_026971	TSS200	S_Shore	
NR_027645;NR_024126;NR_(	TSS1500;TSS1500;TSS1500;TS	S_Shore	
NM_017444;NR_023360	Body;Body	Island	
NM_138764;NM_004324;NM	TSS1500;TSS1500;TSS1500;TS	N_Shore	
NM_138387;NR_028582;NR_	1stExon;Body;Body;5'UTR	Island	

NR_026876	Body	Island	
NM_014620;NM_153693;NR_	TSS200;TSS200;TSS200	N_Shore	
NM_022492;NR_027749;NM_	Body;Body;TSS200	S_Shore	
NR_024367	TSS200		
NM_005378;NM_005378;NR_	1stExon;5'UTR;Body	Island	
NR_027134	TSS200		
NM_000982;NR_026911	TSS1500;TSS1500	N_Shore	
NR_003016;NR_024031	TSS1500;TSS1500	N_Shore	
NM_175047;NR_003613;NM_	5'UTR;TSS1500;5'UTR	S_Shore	
NM_007023;NR_026995	Body;TSS200	Island	
NM_014620;NR_003084;NM_	5'UTR;Body;Body	Island	
NR_002835;NM_005328	Body;5'UTR	Island	
NR_027399;NR_027398;NM_	TSS200;TSS200;TSS200	Island	
NR_027822	Body	Island	DMR
NR_026830	Body	N_Shore	RDMR
NR_028389;NM_001106	Body;Body	Island	
NM_138764;NM_004324;NM	TSS1500;TSS1500;TSS1500;TS	N_Shore	
NM_021643;NM_021643;NR_	1stExon;5'UTR;Body	Island	
NR_002776;NM_001145436;	Body;TSS1500;TSS1500;TSS15	Island	
NM_152520;NR_031659	5'UTR;TSS200	Island	
NR_026757	Body	S_Shore	
NR_003584;NR_002963	TSS200;TSS1500	Island	
NR_024330	Body	Island	DMR
NR_002995	Body	Island	
NM_033028;NR_002780	TSS1500;Body	N_Shore	
NM_005378;NR_026766	Body;TSS1500	Island	
NM_001123067;NM_016835	5'UTR;5'UTR;TSS1500;Body;5	Island	DMR
NM_006041;NR_026880	Body;Body	S_Shore	CDMR
NM_001085365;NR_026922	Body;TSS1500	Island	
NR_028301;NR_028302;NR_(	Body;Body;TSS200	Island	DMR
NM_032741;NM_006913;NR_	TSS1500;TSS200;TSS200		
NM_178040;NR_027949;NR_	TSS1500;TSS1500;TSS1500;TS	N_Shore	RDMR
NR_024184;NR_024182;NR_(	TSS1500;TSS1500;TSS1500		
NR_026550;NM_018442;NM_	TSS1500;Body;TSS1500;TSS15	Island	
NR_024525;NM_003869;NM_	TSS1500;1stExon;TSS1500;1st	Island	
NM_001139518;NM_001042	TSS1500;TSS200;TSS200;TSS1	Island	
NR_026753;NM_031484	Body;1stExon	Island	
NR_002814	TSS200	Island	
NM_021253;NR_024052;NR_	TSS1500;Body;Body	N_Shore	
NR_015431;NM_001145354	TSS1500;5'UTR	Island	
NM_001040144;NR_033227;	TSS200;TSS200;TSS1500;TSS2	Island	
NR_024421;NR_024422	Body;TSS200	Island	
NR_015450	TSS1500	Island	
NM_144611;NR_023347;NR_	3'UTR;Body;Body		

NR_024483;NM_017829;NM_	TSS1500;Body;Body;TSS1500	Island	
NR_004053;NM_003106	Body;TSS200	N_Shore	DMR
NR_029434;NM_002892;NR_	TSS200;TSS1500;TSS200;TSS1	Island	
NR_027253	Body	N_Shore	
NR_003716	Body	Island	DMR
NR_024555;NM_014836;NR_	Body;TSS200;Body;TSS200	Island	
NR_024362;NR_024363;NR_(	TSS1500;TSS1500;TSS1500	Island	
NM_001144072;NM_177967	Body;Body;Body	S_Shore	
NR_027447;NM_016219	TSS200;TSS200	Island	
NR_023350;NM_004766	TSS1500;TSS1500	Island	
NM_001164309;NR_028343;	5'UTR;Body;5'UTR;5'UTR	N_Shore	CDMR
NM_001145391;NM_001145	TSS1500;Body;TSS1500;TSS1500		
NR_024520;NR_024519;NM_	TSS200;TSS200;TSS200;TSS20	Island	
NR_027141;NM_207009	Body;Body	Island	
NR_027068	TSS200	N_Shore	
NM_004640;NR_003065;NM_	5'UTR;TSS1500;5'UTR	N_Shore	
NR_024330	Body	Island	DMR
NR_001435	Body	Island	
NR_026703	Body		
NM_031443;NM_001167935	TSS1500;TSS1500;TSS1500;Bo	Island	
NR_028044;NR_003512;NM_	Body;Body;5'UTR;Body	N_Shore	
NR_003306	TSS200		
NR_024383;NR_015436	Body;Body	N_Shore	
NM_001099625;NM_001099	5'UTR;5'UTR;5'UTR;Body;5'UT	S_Shore	RDMR
NR_027387	TSS1500	N_Shore	
NR_024555;NR_024554	Body;TSS1500	N_Shore	
NR_026550;NM_001143674;	TSS1500;5'UTR;TSS200;TSS15	Island	
NR_023313;NR_023312;NM_	Body;Body;TSS1500	Island	
NM_001040437;NM_001040	5'UTR;5'UTR;1stExon;TSS200;	S_Shore	
NR_027405;NM_006636;NM_	Body;1stExon;5'UTR	Island	
NR_028444;NM_006810	TSS1500;TSS1500	N_Shore	
NR_033258;NM_014795;NM_	Body;Body;Body	Island	DMR
NM_005412;NR_029416;NR_	TSS1500;TSS1500;TSS1500;TS	N_Shore	
NR_028138	Body	Island	
NM_003927;NR_002970;NM_	Body;TSS1500;Body	N_Shore	
NM_021953;NR_027363;NR_	TSS1500;Body;Body;TSS1500	S_Shore	
NM_023924;NR_027633;NM_	TSS1500;TSS1500;TSS1500;Bo	S_Shore	
NR_027425	Body	S_Shelf	
NM_024814;NR_024199	Body;Body	S_Shore	RDMR
NR_027463;NR_027462;NM_	Body;Body;TSS200	S_Shore	
NR_003512;NM_001042376;	Body;Body;1stExon;5'UTR		
NR_028389;NM_001106	Body;TSS1500	N_Shore	RDMR
NR_002942	Body	Island	
NR_003013;NR_027058	Body;Body	S_Shore	

NR_002325;NM_001012321;	TSS1500;TSS1500;5'UTR	S_Shore	
NM_001039465;NM_006925	Body;Body;TSS1500	S_Shore	RDMR
NR_028099;NM_025230;NM_	TSS1500;TSS1500;TSS1500;TS	N_Shore	
NR_021485	Body	S_Shore	DMR
NR_003142;NR_003020;NM_	TSS200;TSS1500;TSS200	Island	
NR_024576;NM_001142628;	Body;Body;Body;Body;Body	S_Shore	
NR_027346;NR_027345	TSS1500;TSS1500		
NR_024350;NM_001136497;	TSS200;TSS200;TSS1500;TSS2	N_Shore	
NR_027795	TSS200		
NM_001001420;NM_005903	5'UTR;5'UTR;5'UTR;Body	Island	
NR_026757	TSS1500	N_Shore	
NR_023362	TSS200		
NR_024612;NM_001137675	TSS1500;TSS1500	N_Shore	
NM_015001;NR_024279	TSS1500;Body	Island	
NR_024587;NM_001142673;	TSS200;TSS200;TSS200;5'UTR	Island	
NM_020866;NM_020866;NR_	1stExon;5'UTR;Body	S_Shore	
NM_001161562;NM_015028	1stExon;1stExon;5'UTR;1stEx	Island	
NR_027068	Body	N_Shore	
NR_027473;NM_012308	Body;Body		
NM_001865;NR_029466	Body;Body		
NR_026972;NR_026973	Body;Body	Island	DMR
NR_027788;NR_027789	Body;Body	Island	
NR_027276	Body		CDMR
NM_022114;NR_015440;NM_	TSS200;TSS1500;TSS200;TSS1	Island	
NR_001558	Body	S_Shelf	
NM_138761;NM_138763;NR_	TSS200;TSS200;TSS200;TSS20	Island	
NM_003292;NM_017847;NR_	TSS1500;TSS200;Body;TSS20	Island	
NR_002188	TSS1500	S_Shore	
NM_003143;NR_015392	TSS200;TSS200	Island	
NM_000800;NM_033136;NM	5'UTR;5'UTR;5'UTR;5'UTR;Body;Body		
NM_207037;NM_207038;NR_	5'UTR;5'UTR;TSS200;1stExon;	Island	
NR_026581;NM_005439	TSS1500;TSS1500	S_Shore	
NR_024383;NR_015436;NR_(	Body;Body;Body		RDMR
NR_024588;NM_014741;NM_	Body;1stExon;5'UTR;1stExon;	Island	
NM_080425;NM_001077490	TSS1500;TSS1500;3'UTR;TSS1	Island	CDMR
NM_001145666;NR_027265;	TSS1500;TSS1500;TSS1500;TS	S_Shore	
NM_018412;NM_021908;NR_	5'UTR;5'UTR;TSS1500;Body;1	N_Shore	
NM_018229;NR_026895;NM_	5'UTR;Body;TSS1500;1stExon	S_Shore	
NR_031766;NM_017899;NM_	Body;Body;Body	N_Shore	
NR_015441	Body	Island	
NR_030159;NM_001143979;	Body;1stExon;5'UTR;TSS1500		
NR_024409;NM_003262;NR_	Body;TSS1500;Body	N_Shore	
NR_029435;NR_029434;NM_	Body;Body;TSS1500;TSS1500	Island	
NM_001142587;NM_001142	TSS1500;TSS1500;TSS1500;B	N_Shore	

NR_027430;NM_025125;NR_	Body;1stExon;Body;Body;Boc	Island	
NR_024588;NM_014741;NM_	Body;1stExon;5'UTR;TSS1500	Island	
NR_003512;NR_028044;NM_	Body;TSS1500;Body;5'UTR;TS	Island	
NM_001164821;NM_024770	Body;1stExon;5'UTR;Body;Bo	S_Shore	
NR_026781;NR_026780	Body;Body	S_Shore	
NM_153262;NR_027458;NR_	Body;Body;Body;Body;Body;E	Island	
NM_001146069;NR_015453;	TSS1500;TSS1500;TSS1500	Island	
NM_053005;NR_021489	Body;Body	N_Shore	
NR_015411	Body	S_Shore	RDMR
NR_028044;NR_003512;NM_	Body;Body;5'UTR;TSS1500;Bc	N_Shore	
NM_002931;NR_029633	TSS1500;TSS1500	N_Shore	
NR_026866	TSS1500		
NR_015440	Body	Island	
NR_026698;NM_002264	Body;5'UTR	Island	
NR_023350;NM_004766	TSS200;TSS200	Island	
NR_003128	Body		
NR_015339	Body	N_Shore	
NR_024519;NR_024518;NR_(	Body;Body;Body;1stExon;1stE	Island	RDMR
NR_031592;NM_007065	TSS1500;TSS1500	Island	
NM_174917;NR_023316;NM_	Body;Body;Body	S_Shelf	
NR_002809	Body	Island	
NR_024585;NM_004747	TSS1500;1stExon	Island	
NR_026989;NR_026985;NR_(	Body;TSS200;Body;TSS200;TS	Island	
NR_033255;NM_012145;NM_	TSS1500;TSS1500;TSS1500	S_Shore	
NR_027276	TSS200		CDMR
NR_027249;NM_003299	Body;TSS1500	Island	
NR_023918;NR_023919	TSS1500;TSS1500	N_Shore	
NM_001007139;NR_003512;	TSS200;Body;Body		
NR_027633;NM_001009877;	Body;Body;Body		
NR_004407	TSS1500		
NR_003551	TSS1500	Island	DMR
NM_001128325;NR_033182;	Body;Body;Body	Island	
NM_001016;NR_002436	Body;TSS200	S_Shelf	
NR_003614;NM_018991;NM_	TSS1500;5'UTR;5'UTR	Island	
NM_001036646;NM_030816	Body;TSS1500;Body;Body;Bo	S_Shore	
NR_024041;NM_152618	TSS1500;5'UTR	S_Shore	
NM_172209;NM_003190;NM	3'UTR;3'UTR;TSS1500;TSS150	S_Shore	
NR_028040;NR_028038;NM_	Body;Body;Body;Body	Island	
NR_030170	TSS200		
NR_003265	TSS200	Island	
NR_026878	Body	N_Shore	RDMR
NR_028437;NR_028436;NM_	TSS200;TSS200;TSS200	N_Shore	
NM_000535;NM_006303;NR_	Body;TSS1500;Body	Island	
NR_026676	Body	S_Shore	

NR_026923;NR_026925;NR_(	Body;TSS200;TSS200	Island	
NR_003264	TSS1500	Island	
NR_033201;NR_033202	TSS1500;TSS1500	N_Shelf	
NM_001046;NR_015360	1stExon;TSS1500	Island	
NR_031646;NM_015678;NM	Body;Body;TSS200	N_Shore	RDMR
NR_029461;NM_007161;NM	Body;TSS200;5'UTR;5'UTR;TSS200;TSS200;Body;5'UTR		
NM_001143766;NM_001143	5'UTR;5'UTR;5'UTR;5'UTR;5'U	N_Shore	
NR_031612;NR_003367	TSS1500;Body		
NR_003667;NM_139235;NM	Body;TSS1500;TSS1500		
NR_024555;NR_024554	TSS1500;TSS1500	Island	
NR_003042;NM_004582	TSS1500;TSS200		
NR_024488	Body	Island	
NM_015509;NR_024260	TSS1500;TSS1500	N_Shore	
NR_033203;NR_033204;NR_(	Body;Body;Body;Body;TSS20(	Island	
NR_003013;NR_027058	TSS1500;TSS200	Island	
NR_030170	TSS200		
NR_026550;NM_018442;NM	TSS1500;TSS1500;5'UTR;TSS1	Island	
NM_002136;NR_002944;NM	Body;Body;Body	S_Shore	RDMR
NR_029969;NR_030160;NR_(	TSS1500;TSS1500;TSS200		
NR_004053	Body	N_Shore	CDMR
NR_026676	TSS200	N_Shore	
NR_003127;NR_024055;NR_(	Body;Body;Body;Body	Island	DMR
NR_028514;NM_130766;NM	Body;TSS1500;TSS1500;TSS1!	S_Shore	
NM_020752;NR_027333	Body;TSS200	Island	
NR_002776;NM_001145436;	Body;TSS1500;TSS1500;TSS1!	Island	
NR_027308;NM_005919;NM	Body;5'UTR;5'UTR;Body	N_Shore	
NR_026814	Body		
NR_002328;NM_003846	TSS200;TSS200	Island	
NR_002834	Body	Island	
NR_026669;NM_014841	TSS200;TSS200	Island	
NM_032359;NR_031662	Body;Body	Island	
NM_001136473;NM_004862	5'UTR;TSS200;5'UTR;Body	Island	
NM_005340;NR_024611;NR_	Body;Body;Body	Island	
NM_001164821;NM_024770	Body;1stExon;5'UTR;Body;Bo	S_Shore	
NR_027463;NM_133491;NR_	Body;TSS200;TSS200	Island	
NR_024368	TSS1500		
NR_027026;NR_027028;NR_(	Body;Body;Body	Island	
NR_028044;NR_003512;NM_	Body;Body;5'UTR;TSS1500;Bc	N_Shore	
NR_023382	TSS1500	N_Shore	
NM_178156;NM_004480;NM	5'UTR;1stExon;5'UTR;5'UTR;1	Island	
NR_027399;NR_027398;NM_	TSS1500;TSS1500;TSS1500	S_Shore	
NR_002971;NM_080686	TSS1500;Body	S_Shore	
NR_026814	Body		
NR_003369	Body	Island	

NR_024114;NM_175888	Body;5'UTR	S_Shore	
NR_027910;NM_001740;NM	TSS1500;TSS1500;TSS1500	N_Shore	
NM_001018108;NR_031695	Body;Body	S_Shore	
NM_001099737;NM_032332	5'UTR;5'UTR;TSS200;Body;1st	Island	
NR_027910;NM_007088;NM	Body;3'UTR;Body		
NM_001042540;NR_024388;	Body;TSS1500;Body	Island	
NM_012459;NM_003002;NR	Body;TSS1500;Body	N_Shore	
NR_026570;NM_014268;NM	TSS1500;TSS1500;5'UTR;Body	N_Shore	
NR_000008;NR_002560;NR_(	TSS1500;TSS1500;Body;Body	N_Shelf	
NR_024399;NM_002461;NR_	Body;TSS1500;Body	Island	
NR_024431;NR_024429;NR_(	TSS1500;TSS1500;TSS1500;TS	Island	
NM_001033002;NR_027684;	Body;Body;Body;Body;Body;B	S_Shore	
NR_027487;NR_026899	Body;Body	Island	
NM_153450;NM_001144012	TSS200;TSS1500;TSS1500;TSS	Island	
NR_024547;NM_003876	TSS1500;TSS1500	S_Shore	
NM_017589;NR_029840;NR_	TSS200;TSS1500;TSS1500	N_Shore	
NM_001141970;NR_024517;	TSS1500;TSS1500;TSS1500;TS	S_Shore	
NR_030396;NM_016091	TSS1500;TSS1500	N_Shore	
NR_024588;NM_014741;NM	Body;1stExon;5'UTR;TSS1500	Island	
NM_206594;NM_206595;NM	Body;Body;Body;Body;Body	N_Shore	
NM_001024380;NM_031940	TSS200;TSS200;TSS1500;TSS1	Island	
NR_026728;NM_001145025;	TSS1500;TSS1500;TSS1500	S_Shore	
NR_028024;NM_001039618;	TSS200;TSS200;TSS200;TSS20	Island	
NR_026719	Body	N_Shore	
NR_027692;NM_002636;NM	TSS1500;TSS1500;TSS1500	Island	
NR_003948	Body		
NM_001001937;NR_026978;	5'UTR;TSS1500;TSS1500	N_Shore	RDMR
NM_001143682;NR_026554;	TSS1500;TSS1500;TSS1500		
NR_031660;NR_003695;NR_(	Body;Body;Body;Body;TSS15(	S_Shore	RDMR
NM_014596;NM_170783;NR	TSS1500;TSS1500;Body	N_Shore	
NM_030573;NM_001008695	Body;Body;TSS1500;TSS1500	N_Shore	
NM_032271;NR_002736	TSS200;TSS1500	Island	
NM_006365;NR_029691	5'UTR;TSS1500	Island	
NR_021490;NR_021491	Body;Body	Island	
NM_001099737;NM_001099	1stExon;5'UTR;TSS200;Body	S_Shore	
NR_027654;NM_005585	Body;1stExon	Island	
NR_000034	Body	Island	
NR_015433	Body	Island	
NM_001141970;NR_024517;	TSS1500;TSS1500;TSS1500;TS	S_Shore	
NM_001145308;NM_001145	TSS200;TSS200;5'UTR;TSS150	N_Shore	RDMR
NR_003191	TSS1500	Island	
NM_021253;NR_024052;NR_	TSS1500;Body;Body;TSS1500	N_Shore	
NR_002935;NM_170606	TSS200;TSS1500		
NM_001164246;NM_017847	TSS1500;TSS1500;Body;TSS15	N_Shore	

NR_024581;NM_024701	Body;Body	Island	
NM_001039618;NM_001039	1stExon;5'UTR;Body;Body;Bo	Island	
NR_002612	Body	Island	
NM_001686;NR_002737	TSS200;TSS1500	S_Shore	
NM_006392;NR_003078;NR_	Body;TSS200;Body;TSS1500	S_Shore	
NR_028074;NM_014828;NM_	TSS200;TSS200;TSS1500;TSS2	Island	
NR_024383;NR_015436;NR_(	Body;Body;TSS1500	N_Shore	
NR_024254	TSS1500	Island	
NR_015454;NM_002359	TSS200;TSS200	Island	
NM_032359;NM_182909;NR_	Body;5'UTR;Body;5'UTR		
NM_080425;NM_001077490	TSS1500;TSS1500;3'UTR;TSS1	Island	CDMR
NM_037370;NM_012142;NR_	TSS200;Body;Body;Body	S_Shore	
NM_001880;NR_030630	TSS1500;TSS1500	Island	
NM_001141970;NR_024517;	TSS1500;TSS1500;TSS1500;TS	S_Shore	
NM_001024381;NM_001024	1stExon;5'UTR;TSS1500;1stEx	Island	
NR_024053;NM_172016;NM_	TSS1500;5'UTR;5'UTR;TSS150	S_Shore	
NM_018691;NM_001135037	5'UTR;TSS1500;TSS1500;TSS1	Island	
NM_031443;NM_001167935	TSS1500;TSS1500;TSS1500;TS	N_Shore	
NM_032825;NR_027239;NM_	5'UTR;TSS1500;TSS1500;TSS1	S_Shore	
NM_003426;NR_003253	TSS200;TSS200	Island	
NM_005916;NM_182776;NR_	Body;Body;TSS200;Body		
NM_001011537;NR_027840;	5'UTR;TSS1500;1stExon;TSS1!	Island	
NM_001144072;NR_026644;	5'UTR;TSS200;TSS200;1stExo	Island	
NM_001167869;NM_001167	5'UTR;TSS1500;5'UTR;Body;5	Island	
NR_015448	Body		CDMR
NR_023938	Body		
NR_027253;NM_006317	Body;TSS1500	N_Shore	
NM_020120;NR_027671	TSS1500;TSS1500	N_Shore	
NM_133378;NM_133432;NM	Body;Body;Body;Body;Body		
NR_023349;NM_052965;NM_	TSS1500;TSS1500;TSS1500	N_Shore	
NM_006913;NM_032741;NR_	1stExon;TSS1500;Body;5'UTR		
NR_004380;NR_002995	Body;TSS1500	Island	
NR_028044;NR_003512;NM_	Body;Body;5'UTR;Body	S_Shore	
NM_004640;NR_003065;NM_	5'UTR;TSS1500;5'UTR	N_Shore	
NR_002165	TSS200		
NM_001143768;NM_182755	5'UTR;5'UTR;1stExon;1stExon	Island	
NR_027766;NR_002949	TSS1500;TSS1500	N_Shore	
NM_001166284;NR_029402;	TSS1500;TSS1500;TSS1500;TS	Island	
NR_033233;NM_001075098	TSS200;TSS200	Island	
NM_006913;NR_003129	Body;Body		
NR_027045;NM_005026	Body;5'UTR	Island	
NR_024610;NM_005340;NR_	TSS200;TSS200;TSS200	S_Shore	
NR_024596;NR_024597;NM_	TSS1500;Body;1stExon;Body;5'UTR		
NM_020196;NM_001171155	TSS1500;1stExon;5'UTR;Body	S_Shore	

NR_027850;NM_006554;NM_	Body;1stExon;5'UTR	Island	
NR_028335	Body		
NM_178040;NR_027948;NR_	5'UTR;Body;Body;5'UTR;Body	S_Shore	
NR_027300	Body	Island	
NR_003671;NM_007175	Body;Body		
NM_001032293;NR_030362;	TSS1500;TSS200;TSS1500;TSS	N_Shore	
NR_004380;NR_002995	TSS1500;TSS1500	Island	
NM_001046;NR_015360	TSS1500;TSS200	Island	
NM_012383;NR_023352;NM_	Body;TSS1500;TSS1500;TSS1	S_Shore	
NR_023383	Body	N_Shore	
NR_029610	TSS1500		
NR_023344	TSS1500	S_Shore	
NR_027433	Body	N_Shore	
NM_015327;NR_026678;NM_	TSS1500;Body;TSS1500		
NM_001037663;NM_001959	Body;Body;TSS1500;TSS1500	S_Shore	
NR_015375;NM_001018056;	TSS1500;Body;Body	Island	
NM_006026;NR_026991	TSS1500;Body	Island	
NR_001591	Body	Island	
NM_001042540;NR_024388;	Body;TSS1500;Body	Island	
NR_023386	Body	N_Shore	
NR_003038;NR_003044;NR_(	Body;TSS1500;TSS1500	Island	
NR_003531;NR_003530;NR_(	Body;Body;Body		
NM_001162861;NM_001162	TSS1500;TSS1500;TSS1500;TS	Island	
NR_015433	TSS1500	N_Shore	
NR_028032	Body	Island	
NR_002785	Body	N_Shelf	
NM_001099626;NM_001099	1stExon;5'UTR;5'UTR;5'UTR;B	S_Shore	
NM_001135179;NM_001135	TSS1500;TSS1500;Body;TSS1	Island	
NR_003551	TSS1500	N_Shore	
NR_027005	Body	Island	
NR_015433	TSS200	Island	
NR_015404;NM_003668;NM_	Body;TSS200;TSS200	Island	
NM_001145308;NM_001145	5'UTR;1stExon;5'UTR;Body;1s	Island	
NM_001011537;NM_001145	TSS200;1stExon;5'UTR;TSS15(	Island	RDMR
NR_024233;NM_199437;NM_	TSS1500;5'UTR;5'UTR	Island	
NR_027053	TSS1500		
NR_028323;NM_080592;NM_	TSS1500;Body;TSS1500;5'UTI	S_Shore	
NR_023319;NR_023318;NM_	Body;Body;Body	Island	
NR_026712;NM_012423	TSS1500;TSS1500	N_Shore	
NR_033258;NM_014795;NM_	Body;5'UTR;1stExon;1stExon;	N_Shelf	DMR
NM_198149;NR_030775;NM_	5'UTR;Body;1stExon		
NR_030725;NR_002909;NR_(	Body;Body;Body;5'UTR		
NM_000520;NR_027262	TSS1500;Body	S_Shore	
NR_026883;NR_026885;NR_(	Body;Body;TSS200		

NR_024012;NM_001129884;	TSS1500;TSS1500;TSS1500	N_Shore	
NR_003109;NM_017846	TSS200;TSS200	Island	
NR_026876	Body	Island	
NR_003923	Body		
NR_026873	Body	S_Shore	
NM_004028;NM_001650;NR_	TSS1500;Body;TSS1500	S_Shore	
NR_003667;NM_016410;NM_	Body;TSS200;TSS200	Island	
NR_003111	TSS200		
NR_024430;NR_029671	Body;Body		
NM_004197;NM_032454;NM	Body;Body;TSS1500;Body	Island	
NR_003512;NM_001127598;	Body;Body;Body;Body	S_Shore	
NR_024377	TSS1500		
NM_001165030;NR_028491;	TSS1500;TSS1500;TSS1500	S_Shore	
NM_001144766;NM_001001	TSS200;TSS1500;Body;Body	Island	
NR_023349;NM_052965;NM_	TSS200;TSS200;TSS200	N_Shore	
NR_027299;NM_001145769;	TSS1500;TSS1500;TSS1500;Bo	Island	
NR_024233;NM_020228;NM_	Body;TSS200;TSS200	Island	
NR_026940;NM_014904;NR_	Body;TSS1500;Body;Body	Island	
NR_002323	Body	S_Shore	
NM_182935;NR_003090	5'UTR;Body	N_Shelf	
NM_003391;NR_024047	Body;Body	N_Shore	CDMR
NR_024600;NM_138929;NM_	Body;5'UTR;5'UTR;Body	Island	
NM_001048224;NM_001048	5'UTR;5'UTR;TSS1500;TSS150	Island	
NM_001144766;NR_029431;	TSS1500;TSS200;TSS200;5'UT	S_Shore	
NM_001146016;NM_001146	TSS1500;TSS1500;TSS1500;TSS1500;TSS1500;TSS1500		
NR_003955	Body	Island	
NM_001146310;NR_024445;	Body;Body;3'UTR	Island	
NR_027910;NM_007088;NM_	Body;1stExon;1stExon	Island	
NM_020476;NM_020478;NM	Body;Body;Body;Body;Body;Body;Body;TSS1500;Body		
NR_029192;NR_024148;NR_(	TSS200;TSS1500;TSS1500;TSS1500		
NM_152618;NR_024041	TSS1500;Body	N_Shore	
NR_024260;NM_015509	TSS200;TSS200	Island	
NR_027093;NR_027092;NR_(	TSS1500;TSS200;TSS200	Island	DMR
NM_001656;NM_024941;NM	TSS200;TSS1500;TSS200;TSS2	Island	
NM_017670;NR_003089	Body;Body	Island	
NR_003615	Body	S_Shore	
NR_024542;NR_024543;NR_(	TSS1500;TSS1500;TSS1500	Island	
NR_024427;NM_001137605	TSS200;TSS200		
NM_003027;NR_026799	TSS1500;TSS1500	N_Shore	CDMR
NM_012399;NR_026963;NR_	Body;TSS1500;TSS1500	Island	
NM_003466;NM_013951;NM	Body;Body;Body;Body;Body;E	Island	
NM_001100619;NR_023359	1stExon;Body	Island	
NM_018177;NR_027277	TSS1500;Body	N_Shore	
NR_028496	TSS200		

NR_024032	TSS1500		
NR_027054	TSS200	Island	DMR
NR_024412;NR_015442	TSS200;TSS200		
NM_001037663;NM_001959	Body;Body;TSS1500;TSS1500	S_Shore	
NR_030724;NM_001167604;	TSS200;TSS200;TSS200	Island	
NR_003954;NM_024694	TSS200;Body		
NM_001161563;NM_001161	Body;Body;Body;Body;Body;B	N_Shore	RDMR
NR_031571	TSS200		
NR_027668;NM_001160225;	Body;Body;Body;Body;Body;Body		
NM_001134888;NR_029965;	1stExon;TSS200;TSS1500	N_Shore	
NR_028044;NR_003512;NM_	Body;Body;5'UTR;TSS1500;Bo	S_Shore	
NR_003094;NR_003092;NR_(	TSS1500;TSS1500;TSS1500;TS	S_Shore	
NR_031592;NM_007065	TSS200;TSS200	Island	
NR_026728;NM_018217;NM	TSS200;TSS200;TSS200	Island	
NR_003684;NM_198216;NM	TSS1500;Body;Body		
NR_024056;NR_024057;NR_(	TSS1500;TSS1500;TSS200;TSS	N_Shore	
NR_002578;NR_003942;NR_(	Body;TSS1500;TSS1500;TSS15	N_Shore	
NR_027308;NM_005919;NM	Body;5'UTR;TSS1500;Body	Island	
NR_027473;NM_012308	Body;Body		
NM_001145354;NR_015431	TSS200;TSS1500	Island	
NR_002971;NM_080686	TSS1500;Body	S_Shore	
NR_024045	TSS1500	Island	
NM_001003809;NR_024101;	Body;Body;Body		
NM_198055;NM_003422;NM	5'UTR;1stExon;1stExon;5'UTR	Island	
NR_002329;NR_002330;NM_	TSS1500;Body;1stExon;1stExo	N_Shore	
NR_024170;NM_021970	TSS200;TSS200		
NR_026703	TSS200		
NM_024721;NR_024360	5'UTR;Body	S_Shore	CDMR
NR_026906;NR_026905	Body;Body		
NR_027663	TSS200	Island	
NR_024280	TSS1500		
NR_015397	TSS1500		
NR_026878	Body	N_Shore	RDMR
NR_027645;NR_024126;NR_(	TSS1500;TSS1500;TSS1500;TS	S_Shore	
NR_002829	TSS1500	N_Shore	RDMR
NR_027105;NR_027106	Body;TSS1500		
NM_001039355;NR_029906;	1stExon;TSS1500;5'UTR	Island	
NR_029380;NM_006562	Body;TSS1500	Island	DMR
NR_029409;NM_019009	Body;TSS1500	S_Shore	
NR_024453	Body	S_Shore	RDMR
NR_002563;NM_001012664;	TSS1500;TSS200;TSS200;TSS1	N_Shore	
NM_015113;NR_023347;NM	TSS1500;Body;Body;Body	Island	
NR_016021;NM_032811	TSS1500;TSS1500	N_Shore	
NR_003316	TSS1500		

NR_001588;NR_024111;NR_(	TSS200;TSS200;TSS200;TSS15	Island	
NR_033258;NM_014795;NM_	Body;5'UTR;5'UTR	Island	DMR
NR_024423;NR_024424	Body;TSS1500	Island	
NM_001130136;NR_027073;	5'UTR;TSS1500;TSS200;TSS20	Island	
NR_027473;NM_012308	Body;Body		
NR_030174	TSS1500		
NR_002809	Body	S_Shore	
NR_030369;NM_138501	TSS1500;TSS1500	Island	
NR_015439;NM_018227	Body;TSS200	Island	
NM_001141969;NR_024517;	5'UTR;Body;Body;5'UTR	N_Shore	
NR_028323;NM_080592;NM_	TSS1500;Body;TSS1500;5'UTF	Island	
NM_002106;NR_002799	TSS1500;Body	S_Shore	
NR_024195;NM_001135732;	TSS1500;TSS1500;TSS1500;TS	N_Shore	
NR_000015;NR_000024;NM_	TSS200;TSS1500;Body	Island	
NR_015427	Body	Island	
NM_001042383;NM_015391	TSS200;5'UTR;Body;TSS1500;	Island	
NR_028024;NM_001039618;	TSS200;TSS200;TSS200;TSS20	Island	
NM_014596;NM_170783;NR_	TSS1500;TSS1500;Body	N_Shore	
NM_138764;NM_004324;NM	TSS1500;TSS1500;TSS1500;TS	N_Shore	
NM_014937;NR_003252	Body;TSS1500	Island	
NM_019605;NR_024337	5'UTR;Body	Island	
NM_001129884;NR_024012;	TSS200;TSS200;TSS200	Island	
NR_003594;NM_172239	Body;1stExon	Island	
NR_026583	Body		
NM_016649;NM_024120;NM	TSS200;TSS200;TSS200;TSS20	Island	
NR_027119;NR_027118;NM_	TSS200;TSS200;Body		
NR_003525	TSS1500	Island	
NM_005510;NM_004197;NM	5'UTR;Body;Body;1stExon;Bo	Island	
NM_001018056;NM_003383	1stExon;5'UTR;1stExon;5'UTF	Island	
NR_003142;NM_002952;NR_	Body;TSS1500;TSS200	Island	
NR_024367	TSS1500		
NR_024184;NR_024182;NR_(	TSS1500;TSS1500;TSS1500		
NR_027126;NM_138777;NR_	Body;TSS200;Body;5'UTR;TSS	Island	
NR_027042	Body		
NM_001042540;NR_024388;	Body;TSS1500;Body	N_Shore	
NR_026940;NM_014904;NR_	Body;TSS1500;Body;Body	S_Shore	
NR_026732;NR_026731	Body;Body	S_Shore	
NM_001142273;NM_015282	Body;Body;Body;TSS1500	Island	
NR_015380;NM_130786	TSS1500;Body	Island	
NR_002950;NM_017822	Body;Body		
NM_003027;NR_026799	TSS1500;TSS1500	N_Shore	CDMR
NR_027269	TSS1500	S_Shore	
NM_001141970;NR_024517;	TSS1500;TSS1500;TSS1500;TS	S_Shore	
NR_026550;NM_001143674;	TSS1500;5'UTR;TSS200;TSS15	Island	

NR_027293	TSS1500		
NR_029383;NM_175854;NM_	Body;TSS1500;TSS1500	N_Shore	
NR_026815	Body	Island	
NR_027006;NR_002829	TSS1500;TSS1500	N_Shore	RDMR
NR_030627;NM_014687;NM_	TSS1500;Body;Body		
NM_004640;NR_003065;NM_	5'UTR;TSS1500;5'UTR	N_Shore	
NM_207354;NR_030767	Body;Body	Island	
NR_028044;NR_003512;NM_	Body;Body;5'UTR;Body;TSS20	S_Shore	
NM_001033088;NR_028052	TSS1500;TSS1500	N_Shore	
NR_030299	TSS1500		
NR_027663	Body	Island	
NM_022771;NM_001146213	Body;Body;5'UTR;Body	S_Shore	RDMR
NM_015913;NM_001136497	TSS200;TSS1500;TSS1500;TSS	N_Shore	
NR_026871;NM_001145210	TSS1500;TSS1500	S_Shore	
NR_001289;NR_001295	TSS1500;TSS1500		
NM_001142464;NR_024551;	5'UTR;TSS200;5'UTR;5'UTR	Island	
NM_178040;NR_027949;NR_	TSS1500;TSS1500;TSS1500;TS	N_Shore	RDMR
NM_013349;NR_026598	Body;Body	Island	
NR_024252	Body		
NM_001163484;NM_025230	5'UTR;5'UTR;TSS200;Body;TS	S_Shore	
NR_003679	Body	N_Shore	RDMR
NM_001127213;NR_026913;	5'UTR;TSS1500;5'UTR	Island	DMR
NR_024409;NM_003262;NR_	Body;TSS1500;Body	N_Shore	
NR_024194;NR_024195;NM_	Body;Body;Body;Body;Body;Body		
NR_028050;NR_028051;NR_(	Body;Body;Body;Body	Island	
NR_024416;NR_015401;NR_(	TSS200;Body;Body	Island	
NR_024279	Body	Island	
NM_001145025;NM_018217	Body;Body;Body	N_Shore	
NR_027771	Body	Island	
NR_027333;NM_020752	Body;TSS200	Island	
NR_027335;NR_027336	TSS1500;TSS1500		
NR_002729	Body	S_Shelf	
NR_024271	TSS200	Island	
NM_001145268;NM_001145	TSS200;TSS200;TSS200	N_Shore	
NM_003391;NR_024047	TSS1500;TSS1500	Island	DMR
NM_001144766;NR_029431;	TSS1500;TSS1500;TSS1500;5'	N_Shore	
NM_006151;NR_027647;NM_	Body;Body;Body	Island	
NM_030631;NM_001171170	Body;Body;Body	Island	DMR
NM_002136;NR_002944;NM_	TSS1500;TSS1500;TSS1500;TS	Island	
NR_021485	Body	Island	
NR_024418	Body	Island	
NM_001164821;NM_024770	TSS1500;5'UTR;TSS1500;TSS1	N_Shore	
NM_006026;NR_026991	TSS200;Body	Island	
NR_027504	Body	Island	

NR_002956;NM_014765	TSS1500;TSS200	Island	
NR_027148	TSS200		
NM_000990;NR_002977	Body;TSS1500	S_Shore	
NR_027105;NR_027106	Body;TSS1500		
NR_027410	Body	Island	DMR
NM_003295;NR_024458	TSS200;TSS200	Island	
NR_024255;NM_002051;NM_	TSS1500;TSS1500;TSS1500;TS	Island	DMR
NR_026862	TSS1500	S_Shore	
NR_027692;NM_002636;NM_	Body;Body;Body	N_Shelf	
NR_024042	Body		
NR_030306;NM_001007527	TSS1500;5'UTR	N_Shore	
NR_024546;NM_004786	Body;Body	Island	
NM_024678;NR_027479	TSS1500;TSS1500	S_Shore	
NR_023349;NM_052965;NM_	TSS200;TSS200;TSS200	N_Shore	
NR_030170	TSS200		
NR_003948	Body		
NM_171829;NM_001163677	TSS1500;Body;TSS1500;Body	N_Shore	
NM_017670;NR_003089	Body;Body	S_Shore	
NR_027433;NM_001003845	Body;TSS1500	Island	
NR_030724;NM_020383;NM_	Body;Body;Body	Island	
NM_000995;NM_033625;NR_	5'UTR;5'UTR;TSS1500		
NR_026956	TSS200		
NM_001142587;NM_001142	TSS1500;TSS1500;TSS1500;Bo	N_Shore	
NR_003941;NR_003944;NM_	TSS1500;TSS1500;TSS1500;TS	N_Shore	
NR_015352	TSS200	Island	
NR_026717;NM_005510	TSS1500;Body	N_Shore	
NR_024399;NR_024402	Body;Body		
NR_030331;NR_026677	Body;Body		
NR_027113;NR_027114	Body;Body		
NR_024370;NR_015396;NM_	TSS1500;TSS1500;1stExon;5'U	Island	
NM_206836;NM_006117;NR_	Body;5'UTR;Body;5'UTR	Island	
NM_032810;NR_028492	TSS1500;Body	Island	
NR_024148;NR_029192;NR_(	TSS1500;Body;TSS1500;TSS1500		
NM_001137675;NR_024612	5'UTR;Body	Island	
NR_003261	TSS1500		
NM_004071;NR_027855;NM_	5'UTR;Body;Body;Body	N_Shore	RDMR
NM_001170803;NM_199188	TSS1500;TSS1500;TSS1500;TS	N_Shore	
NR_015357	Body	Island	
NR_024383;NR_015436;NR_(	Body;Body;Body	N_Shore	
NR_021490;NR_021491	Body;Body	Island	
NR_030717;NM_003083	TSS1500;TSS1500	N_Shore	
NR_003249;NM_031372	Body;Body	N_Shore	
NR_024172;NR_024173	TSS1500;TSS1500		
NR_026799;NM_003027;NM_	Body;1stExon;5'UTR	Island	DMR

NR_031694	TSS1500	N_Shore	
NM_152872;NR_028034;NM_	Body;Body;Body;Body;TSS15(	S_Shore	
NR_026825;NM_003484;NM_	Body;Body;Body	Island	
NR_026989	Body	N_Shore	
NR_026977;NM_012432;NM_	TSS1500;TSS200;TSS200	Island	
NR_027301	TSS200	Island	
NM_002264;NR_026698	TSS1500;TSS1500	S_Shore	
NM_032772;NM_032772;NR_	1stExon;5'UTR;TSS200	Island	
NR_026977;NM_012432;NM_	Body;5'UTR;5'UTR	S_Shore	
NM_003466;NM_013951;NM	Body;Body;Body;Body;Body;E	N_Shore	
NR_027038;NM_021038;NR_	Body;5'UTR;Body;5'UTR	Island	
NR_026744	TSS200		
NR_026878	Body	Island	
NR_026971	TSS1500	Island	
NM_002904;NM_006929;NR_	Body;TSS1500;TSS1500	N_Shore	
NM_006515;NR_024022	Body;Body	S_Shore	
NR_003290	Body	N_Shore	
NM_138638;NR_028131;NR_	TSS1500;TSS1500;TSS1500;TS	S_Shore	
NR_026855;NR_026856	Body;Body	S_Shore	
NR_002819	Body	S_Shelf	
NR_002563;NR_002564;NR_(	TSS1500;TSS200;TSS1500;TSS	N_Shore	
NR_023389;NM_001010895;	Body;TSS1500;Body	Island	
NR_003716	Body	S_Shore	
NR_002139	Body	Island	
NR_028339;NR_028340	TSS200;TSS200	S_Shore	
NR_026753;NM_031484	Body;1stExon	Island	
NR_028134	Body		
NR_002801	TSS200		
NM_014596;NM_170783;NR_	TSS1500;TSS1500;Body	N_Shore	
NR_000024;NM_001012;NR_	TSS200;Body;TSS1500	S_Shore	
NR_026681	TSS200		
NR_024435;NR_024434;NM_	Body;Body;TSS1500	Island	
NR_033184;NM_178549	TSS1500;TSS1500	N_Shore	
NR_002441;NM_000968;NR_	TSS200;Body;TSS1500;TSS1500		
NR_002781	TSS1500	S_Shore	
NR_029373;NR_029374	TSS1500;Body	N_Shore	RDMR
NR_003256;NM_005050	Body;Body	Island	
NR_027476;NM_032750;NM_	TSS200;TSS200;TSS1500;TSS2	N_Shore	
NM_138615;NM_014966;NR_	Body;Body;TSS1500	N_Shore	
NR_027118;NR_027119;NM_	Body;Body;TSS1500	N_Shore	
NM_182931;NR_024586;NM_	5'UTR;TSS1500;5'UTR	S_Shore	
NR_031688	TSS1500		
NM_000967;NR_002439;NM_	Body;Body;Body	N_Shore	
NM_001146310;NR_024445	Body;Body	N_Shore	

NR_024534;NM_032331;NM_	TSS1500;TSS200;TSS1500;TSS	Island	
NR_026823	Body		
NR_002894	TSS200	Island	
NR_024014;NM_002081	Body;Body	N_Shelf	
NM_002136;NR_002944;NM_	TSS1500;TSS1500;TSS1500;TS	Island	
NR_026756	TSS1500		
NR_015436	Body	Island	DMR
NR_026680;NR_027295	Body;Body	S_Shelf	
NR_002447;NM_181491;NM_	TSS1500;TSS200;Body;TSS20(	Island	
NR_003605;NR_003606;NR_(	Body;Body;TSS200;TSS1500;T	S_Shore	RDMR
NR_027240	TSS1500		
NM_001880;NM_001880;NR_	1stExon;5'UTR;TSS1500	Island	
NR_024254	TSS1500	Island	
NR_024255;NM_002051;NM_	TSS1500;TSS1500;TSS1500;TS	Island	DMR
NR_002561;NR_000008;NR_(	TSS200;TSS1500;TSS1500;Bo(	N_Shore	
NM_002103;NM_001161587	TSS1500;TSS1500;TSS1500;1s	S_Shore	
NM_001167738;NR_031599;	Body;TSS200;Body		
NR_027293	TSS1500		
NR_003605;NR_003606;NR_(	Body;Body;TSS200;Body	S_Shelf	RDMR
NR_003365	Body	N_Shore	
NM_001165030;NR_028491;	TSS1500;TSS1500;TSS1500	S_Shore	
NR_002729	Body	Island	
NM_001005909;NM_016291	Body;Body;3'UTR;Body;Body;3'UTR;3'UTR;3'UTR		
NM_017996;NR_026645;NM_	TSS200;TSS200;TSS200		
NR_026567	Body	Island	DMR
NR_030345	TSS1500		
NR_003038;NR_003044;NR_(	Body;Body;TSS1500	N_Shore	
NR_024355	TSS200		
NR_021490;NR_021491	Body;Body	Island	
NM_020120;NR_027671	Body;Body	Island	
NR_027154;NR_027460	TSS200;Body	Island	
NR_002894	Body	Island	
NM_001025202;NR_027777;	TSS1500;Body;Body;Body	Island	
NM_006098;NR_002591	TSS200;TSS1500	Island	
NR_024451;NM_030647	Body;TSS1500	Island	
NR_027390;NM_001018090;	Body;Body;Body;Body;Body	Island	
NR_003684;NM_198216;NM_	TSS200;Body;Body		
NM_018095;NR_024222;NM_	TSS1500;TSS1500;Body;TSS1!	S_Shore	
NM_001167869;NR_030765;	Body;Body;Body;Body;Body;E	N_Shelf	
NR_031666;NM_001135212;	Body;Body;Body	N_Shore	
NR_027412;NR_027413	Body;Body		
NR_027428;NR_027430;NM_	TSS200;TSS200;Body;TSS200;	Island	RDMR
NM_032141;NR_029945	TSS200;TSS1500	Island	
NR_024469	TSS1500	N_Shore	

NR_028040;NM_012447;NM_	Body;3'UTR;3'UTR;Body;Body		
NR_028374;NM_002539	Body;5'UTR	N_Shore	
NR_003671;NM_007175	TSS200;Body		
NR_030159;NM_001143979;	Body;1stExon;5'UTR;TSS200		
NM_176895;NM_003711;NR	Body;Body;Body	N_Shore	
NR_027333;NM_020752	Body;1stExon	Island	
NR_030724;NM_020383;NM_	Body;Body;Body		
NR_028437;NR_028436;NM_	TSS200;TSS200;TSS200	N_Shore	
NR_024219;NR_024217;NR_(	TSS1500;TSS1500;TSS1500		
NR_002612	TSS1500	Island	RDMR
NR_024584	Body	Island	
NM_007074;NR_002454	Body;TSS1500	S_Shelf	CDMR
NM_001085365;NR_026922	TSS1500;TSS200	Island	
NR_001434	Body	Island	
NM_003029;NM_001130041	5'UTR;5'UTR;TSS1500;TSS150	Island	
NR_030174	TSS200		
NR_027073;NM_020851;NM_	Body;TSS1500;TSS200;TSS15(	Island	
NR_027404;NM_001036645;	TSS1500;TSS1500;TSS1500;TS	Island	
NM_139235;NR_003667;NM_	TSS200;Body;TSS200		
NR_002824	Body		
NM_147195;NM_147195;NR	1stExon;5'UTR;TSS1500	Island	DMR
NR_026815	Body	Island	
NR_003948	Body		
NR_001435	Body		
NR_031602;NM_001006944;	TSS1500;Body;Body	N_Shore	
NM_133474;NR_002451	5'UTR;Body	N_Shore	
NR_001283;NM_001008496	TSS200;5'UTR	S_Shore	
NM_001173;NM_001030055	1stExon;5'UTR;1stExon;TSS15	Island	
NM_001018038;NM_001018	TSS1500;TSS1500;Body;TSS15	N_Shore	
NM_032741;NM_006913;NR	TSS1500;Body;Body		
NM_005632;NR_024121	TSS1500;Body	Island	
NR_024467;NM_182765;NM_	Body;Body;Body	Island	
NM_001362;NR_031649	TSS1500;TSS200	Island	
NR_028272	TSS200	Island	
NR_026697;NM_001070;NM_	TSS200;5'UTR;TSS200;1stExo	Island	
NR_031597;NM_002332	TSS1500;Body		
NM_001146310;NR_024445;	Body;Body;Body	Island	
NR_002972;NR_003239	TSS1500;Body	Island	
NR_001434	Body	Island	
NR_024080;NM_004300;NM_	TSS1500;TSS1500;Body;TSS15	Island	
NR_028596;NM_001957;NM_	Body;1stExon;5'UTR;1stExon;	Island	DMR
NR_027073;NM_020851;NM_	Body;TSS1500;TSS200;TSS15(	Island	
NR_026906;NR_026905;NR_(	TSS1500;TSS1500;Body;Body		
NM_025125;NR_027430;NR_	TSS1500;Body;Body;Body;Bo	N_Shore	

NR_031714	TSS1500		
NR_024254	TSS1500	Island	
NM_178580;NM_030789;NM	Body;Body;Body;TSS1500	N_Shore	
NR_024127;NR_003077;NR_(	Body;TSS1500;Body	N_Shore	
NR_024260;NM_015509	Body;Body	S_Shore	
NM_037370;NM_012142;NR_	TSS200;Body;Body;Body	S_Shore	
NM_014911;NR_003705	Body;TSS1500		
NR_026669;NM_014841	TSS200;TSS200	Island	
NR_031681	TSS1500		
NR_003063;NM_006000	Body;Body	Island	
NR_024079	TSS1500	Island	
NR_024399;NM_178310	Body;Body	Island	
NR_002799;NM_002106	TSS1500;Body	Island	
NR_031716;NM_021241	TSS1500;5'UTR		CDMR
NR_024041;NM_152618	TSS200;TSS200	Island	
NR_002185	TSS1500		
NR_002710	Body	Island	
NR_027005	TSS1500	S_Shore	
NR_026961	TSS200		
NM_001141970;NR_024517;	TSS1500;TSS1500;TSS1500;TS	S_Shore	
NR_002801	Body		
NR_027400;NM_000543;NM_	TSS1500;TSS1500;TSS1500	N_Shore	
NR_015453;NM_001146069;	TSS1500;TSS200;TSS1500	Island	
NM_014182;NR_026723;NR_	TSS1500;Body;Body;Body	Island	
NR_026580;NM_007111	Body;Body	N_Shore	
NR_024534;NM_032331;NM_	TSS1500;TSS200;TSS1500;TSS	Island	
NR_024465;NM_006110	TSS1500;TSS1500	S_Shore	
NM_152410;NM_001080378	Body;Body;Body;Body		
NM_001143971;NM_001143	5'UTR;5'UTR;Body;Body		
NR_031766;NM_017899;NM_	Body;Body;Body	S_Shore	
NR_030155;NR_030594;NR_(	TSS1500;TSS1500;TSS1500;TS	N_Shore	
NR_003042;NR_002749;NM_	TSS1500;TSS1500;Body		
NR_015370;NR_002922	Body;TSS1500	Island	
NR_003671;NM_007175	Body;Body		
NM_206953;NR_027426;NM_	TSS200;Body;TSS200;TSS150(	S_Shore	
NR_002767;NM_018064	TSS1500;TSS1500	S_Shore	
NR_027421	Body		
NR_027387	TSS200	Island	
NR_023313;NR_027271;NM_	Body;TSS200;5'UTR;Body	S_Shore	
NR_027822	Body	Island	
NM_033542;NR_003189	TSS200;TSS200	Island	
NR_002798	Body	Island	
NR_027449;NM_001146214;	TSS1500;TSS1500;TSS1500;TS	N_Shore	
NR_003569	Body	S_Shore	

NM_001142301;NM_153704	TSS200;TSS200;TSS200	N_Shore	
NR_024396	TSS200		
NR_015404;NM_003668;NM_	TSS1500;Body;Body	S_Shore	
NM_001961;NR_002602	Body;TSS1500	Island	
NM_152872;NR_028034;NM_	Body;Body;Body;Body;TSS15(	S_Shore	
NR_026777	Body	N_Shore	
NR_028044;NR_003512;NM_	Body;Body;5'UTR;TSS1500;Bo	N_Shore	
NM_001013615;NR_024332	TSS200;Body	Island	
NR_024024;NM_001607;NM_	TSS1500;TSS1500;TSS1500;TS	Island	
NM_001686;NR_002737	TSS200;TSS1500	S_Shore	
NM_145039;NR_003228;NR_	5'UTR;TSS200;TSS200;1stExo	Island	
NR_003138;NM_016417;NR_	Body;TSS200;TSS1500;Body	Island	
NR_024270	TSS1500	Island	
NR_003290	Body	S_Shelf	
NR_024580;NM_017995;NM_	Body;TSS200;TSS200	Island	
NM_177925;NM_177925;NR_	5'UTR;1stExon;Body	Island	
NR_026580;NM_007111	Body;Body	N_Shore	
NR_031572;NM_001170402;	TSS1500;TSS1500;TSS1500;TS	S_Shore	
NM_033028;NR_002780	TSS1500;Body	N_Shore	
NM_020476;NM_020478;NM	Body;Body;Body;Body;Body;Body;Body;TSS1500;Body		
NR_003655	TSS200	Island	
NM_000977;NM_033251;NR_	5'UTR;1stExon;TSS1500;5'UT	Island	
NM_080598;NR_003140;NM_	Body;TSS200;Body		
NR_001558	Body	Island	DMR
NR_026590	TSS1500		
NR_027138	TSS1500	N_Shore	
NR_002956;NM_014765	TSS1500;TSS200	Island	
NR_003013;NR_027058	TSS1500;TSS200	Island	
NM_001098533;NR_027703;	TSS1500;TSS1500;TSS1500;TS	N_Shore	
NR_024383;NR_015436	Body;Body	Island	DMR
NM_018442;NM_001143674	TSS1500;5'UTR;TSS200;TSS20	Island	
NR_002315;NM_002107	TSS1500;TSS1500	N_Shore	
NR_003659	Body	S_Shore	
NR_015357	Body	S_Shore	
NM_006392;NR_031699;NR_	1stExon;TSS200;Body;5'UTR	Island	
NR_002799;NM_002106	TSS1500;Body	Island	
NR_022012;NM_153264	Body;Body		
NR_003086	TSS200	Island	
NR_030342;NM_004111;NM_	TSS200;5'UTR;TSS200;1stExo	Island	
NR_002822	Body	N_Shore	
NR_027148	Body		
NR_024158	Body	N_Shore	
NR_003276	Body	N_Shore	
NR_027816;NM_152744	Body;Body	Island	

NR_023350;NM_004766	TSS1500;TSS1500	S_Shore	
NM_001141947;NR_024460;	TSS200;TSS200;TSS200	Island	
NM_013406;NM_001099737	3'UTR;3'UTR;3'UTR;3'UTR;3'UTR;Body		
NM_001166253;NR_029394;	Body;Body;Body;Body;Body;Body		
NR_029970;NR_029685	TSS200;Body		
NR_002187;NM_012470;NM_	TSS200;1stExon;5'UTR	Island	
NR_026972;NR_026973	Body;Body	N_Shelf	
NM_016417;NR_003138;NR_	Body;TSS1500;TSS1500	S_Shore	
NM_001130136;NR_027073;	5'UTR;TSS1500;TSS1500;TSS1	Island	
NR_024467	Body		
NR_029582	TSS1500	Island	
NR_027697;NR_027696	Body;Body	N_Shelf	
NM_175895;NR_029661	TSS1500;TSS200	Island	
NR_027322	TSS1500		
NM_152835;NR_026686;NR_	Body;Body;Body	S_Shore	
NR_002785;NM_016592	TSS200;3'UTR	N_Shore	CDMR
NR_026753;NR_026754;NM_	Body;Body;3'UTR	S_Shelf	
NR_028077;NM_001163391	Body;5'UTR	Island	DMR
NR_003307;NR_003313;NR_(	Body;Body;TSS1500		
NR_026875	Body	N_Shore	
NR_023392;NR_026974	Body;TSS1500	N_Shore	
NR_026830	Body	N_Shore	RDMR
NM_001145294;NR_026937;	TSS1500;Body;5'UTR;5'UTR		RDMR
NR_026999	TSS1500	N_Shore	
NR_015405	Body		
NR_028272	TSS1500	N_Shore	RDMR
NR_002196	Body	N_Shore	
NR_030621	TSS200	Island	
NR_003228;NM_145039;NR_	Body;TSS200;Body;Body	Island	
NR_024356	TSS200	Island	DMR
NM_144610;NR_024395	Body;TSS1500		
NM_001686;NR_003046;NR_	Body;TSS1500;Body	N_Shore	
NR_024374	Body		
NM_004111;NM_014206;NR	5'UTR;TSS1500;TSS1500	S_Shore	
NM_001167966;NR_030778;	TSS1500;TSS1500;Body;Body	Island	
NR_003714	TSS1500	N_Shore	
NM_021970;NM_021970;NR	1stExon;5'UTR;Body		
NR_015375	Body	N_Shore	
NR_027257	TSS1500	N_Shore	
NM_001166343;NR_029413;	TSS1500;TSS1500;TSS1500		
NR_000034	TSS1500	S_Shore	
NM_016093;NR_026683;NR_	5'UTR;TSS1500;TSS200	Island	
NR_027337	Body	S_Shore	
NR_026789;NR_024044	Body;Body		

NM_004824;NR_026590;NM_	Body;Body;Body;Body		
NR_028044;NM_001042376;	Body;3'UTR;Body;5'UTR;Body	S_Shelf	
NR_003266	Body	N_Shore	
NR_002437;NM_001023;NM_	TSS200;Body;Body	Island	RDMR
NR_029607;NR_027148	TSS1500;Body		
NR_026671;NR_026672;NM_	TSS1500;TSS1500;TSS1500		
NM_015363;NM_001146327	TSS200;TSS200;TSS200;TSS2C	Island	
NM_017589;NM_001100388	TSS1500;TSS1500;TSS1500;Bc	N_Shore	
NR_003037	TSS200		
NM_032810;NR_028492	TSS1500;Body	Island	
NR_001577	Body		
NR_029373;NR_029374	TSS1500;Body	Island	RDMR
NR_033258;NM_014795;NM_	Body;5'UTR;1stExon;1stExon;	N_Shelf	DMR
NR_015415;NM_177438	Body;TSS1500	Island	
NM_016122;NM_001042399	1stExon;5'UTR;TSS1500;5'UTI	Island	
NR_026578	Body		
NM_006195;NR_024123;NM_	1stExon;Body;TSS1500;Body	Island	
NR_026752	Body		
NM_006761;NR_024058	Body;Body		
NR_031598;NM_014275;NM_	TSS200;Body;Body	S_Shore	
NM_006041;NR_026880	Body;TSS1500	Island	CDMR
NR_027349;NR_027350	Body;Body	Island	
NR_003252;NM_014937	Body;Body	S_Shore	RDMR
NR_027633;NM_001009877;	Body;Body;Body	N_Shore	
NM_003927;NR_002970;NM_	Body;TSS1500;Body	N_Shore	
NR_003920	Body		
NR_002821	Body	Island	
NM_018362;NR_002832	TSS1500;Body	S_Shore	
NM_153365;NR_027697;NR_	TSS1500;Body;Body	Island	
NR_002327;NM_002952	TSS200;Body	N_Shore	
NR_003605;NR_003606;NR_(	Body;Body;Body	S_Shelf	RDMR
NR_003266	TSS1500	Island	
NR_027423	Body		
NM_001865;NR_029466	TSS200;TSS200		
NM_172003;NR_029399	Body;TSS1500		
NR_003698	TSS200	Island	
NR_024281;NR_029668	TSS1500;TSS200	Island	
NM_199191;NM_199193;NM	TSS1500;TSS1500;TSS1500;TS	Island	
NR_016021;NM_032811	TSS1500;TSS1500	N_Shore	
NM_153264;NR_022012	5'UTR;Body	S_Shore	
NR_003307;NR_003313;NR_(	Body;Body;Body;TSS200		
NR_015436	Body	S_Shelf	
NM_001130001;NM_001130	5'UTR;5'UTR;1stExon;1stExon	S_Shore	
NR_027399;NR_027398;NM_	TSS1500;TSS1500;TSS1500	S_Shore	

NM_019057;NR_024019	Body;Body	N_Shore	
NR_004846	TSS1500	N_Shore	
NM_001130042;NM_001889	TSS1500;TSS1500;Body;TSS1500;Body;TSS1500;Body		
NR_029192;NR_024148;NR_(	TSS200;TSS1500;TSS1500;TSS1500		
NM_001039693;NR_004862;	TSS1500;TSS1500;Body	Island	
NR_002944;NM_031157;NM_	TSS200;TSS200;TSS200;TSS15	Island	
NM_001024660;NM_001024	1stExon;5'UTR;Body;5'UTR;1stExon		
NM_001161563;NM_001161	Body;Body;Body;Body;Body;E	N_Shore	
NM_001139518;NM_001042	TSS1500;TSS200;TSS200;TSS1	Island	
NM_001100592;NR_027040;	5'UTR;Body;5'UTR;1stExon;1s	N_Shore	
NM_020120;NR_027671	TSS1500;TSS1500	N_Shore	
NM_001159554;NM_005446	1stExon;1stExon;TSS1500	S_Shore	
NR_026789	Body		
NM_000990;NR_002580	Body;TSS1500	Island	
NR_015428	Body	Island	
NR_002733	Body	Island	
NM_001048223;NM_001048	TSS1500;TSS1500;Body;TSS15	N_Shore	
NR_024412;NR_015442	TSS1500;TSS1500		DMR
NM_152786;NR_027261;NM_	5'UTR;TSS1500;1stExon;TSS15	S_Shore	
NR_027435;NM_020801	TSS1500;Body	N_Shore	
NR_015404;NM_003668;NM_	Body;TSS200;TSS200	Island	
NM_001079522;NR_027123;	TSS1500;Body;TSS1500;TSS15	N_Shore	RDMR
NR_026872;NM_002602	Body;Body	S_Shelf	
NM_003143;NR_015392	5'UTR;TSS1500	Island	
NR_003042;NM_004582	TSS1500;Body		
NR_004053	Body	N_Shore	
NM_199193;NM_022128;NM	5'UTR;TSS1500;5'UTR;5'UTR;5	S_Shore	
NM_001037663;NM_001959	Body;Body;TSS1500;TSS1500;	S_Shore	
NR_028408	TSS200	Island	
NM_005378;NR_026766	5'UTR;TSS1500	Island	
NR_024383;NR_015436	Body;Body	Island	
NM_015913;NM_015913;NM	1stExon;5'UTR;TSS1500;TSS15	Island	
NR_024453	Body	S_Shore	RDMR
NR_027908	Body	N_Shore	
NR_002586;NR_031650;NM_	TSS1500;TSS1500;Body;TSS15	S_Shore	RDMR
NR_028337	TSS1500	N_Shelf	
NR_027822	Body		
NR_026857;NM_017450;NM_	TSS1500;Body;Body;Body;Bo	Island	DMR
NM_018844;NR_027830	TSS1500;TSS1500	N_Shore	
NR_031666	Body	S_Shore	
NM_032391;NR_024103	TSS200;TSS1500	N_Shore	
NR_003700	Body	N_Shelf	
NR_003086	Body	S_Shore	
NR_027463;NM_133491;NR_	Body;5'UTR;TSS1500;1stExon	Island	

NR_024514;NM_139027;NM_	Body;Body;Body;TSS1500;TSS	N_Shore	
NR_029372;NM_001002010	TSS200;TSS200	Island	
NM_020988;NM_138736;NR_	TSS1500;TSS1500;Body	N_Shore	DMR
NR_026728;NM_018217;NM_	TSS200;TSS200;TSS200	Island	
NR_004401;NM_001352	TSS1500;1stExon	Island	
NM_001100592;NR_027040;	5'UTR;Body;5'UTR;1stExon;1s	Island	
NR_024603;NM_004870	Body;1stExon	Island	
NR_030320	TSS200	S_Shelf	
NM_001865;NR_029466	TSS200;TSS200		
NM_199193;NM_022128;NM	5'UTR;TSS1500;5'UTR;5'UTR;5	S_Shore	
NR_028044;NR_003512;NM_	Body;Body;5'UTR;TSS1500;Bo	N_Shore	
NR_024253	Body		
NR_015358	Body		
NR_028437;NR_028436;NM_	TSS200;TSS200;TSS200	N_Shore	
NR_028473;NM_016464	Body;Body	S_Shelf	
NR_030345	TSS200		
NM_001015;NR_001285	TSS200;TSS1500	Island	
NR_015415;NM_177438	Body;TSS1500	S_Shore	
NR_026745;NM_001145079;	TSS200;TSS200;TSS200	N_Shore	
NR_002307	TSS200	N_Shore	
NM_020386;NR_026877	TSS1500;TSS1500	N_Shore	
NM_021253;NR_024052;NR_	TSS1500;Body;Body	N_Shore	
NM_001171155;NR_033242;	TSS1500;TSS1500;Body	N_Shore	
NM_144704;NR_027464;NM_	3'UTR;Body;3'UTR;3'UTR;TSS1	N_Shore	
NM_001003809;NR_024101;	Body;Body;Body		
NR_015370;NR_002922	Body;TSS1500	Island	
NR_026677	TSS1500		
NM_006392;NR_027700;NR_	Body;Body;TSS1500	Island	
NR_024600;NM_138929;NM_	Body;5'UTR;5'UTR;Body	Island	
NR_027660;NM_001160183;	TSS1500;TSS1500;TSS1500;TS	N_Shore	
NR_024484	Body	Island	
NR_027816;NM_152744	Body;Body		
NR_003360	TSS1500		
NM_139235;NR_003667;NM_	Body;Body;Body		
NM_006117;NM_206836;NR_	TSS1500;TSS1500;TSS1500;TS	Island	
NM_016145;NM_032332;NM	1stExon;TSS200;5'UTR;Body;5	Island	
NM_001083614;NR_003501;	1stExon;Body;TSS200;5'UTR	Island	
NR_031766;NM_017899;NM_	Body;Body;Body	N_Shore	
NR_000034	TSS1500	Island	
NM_022492;NR_027749;NM_	Body;Body;TSS200	S_Shore	
NM_002222;NM_001099952	Body;Body;Body;TSS1500		
NR_024519;NR_024518;NR_(	Body;Body;Body;Body;Body;B	N_Shore	RDMR
NR_029373;NR_029374	Body;Body	Island	DMR
NM_017884;NR_031711	Body;TSS200		

NM_018177;NR_027277	TSS1500;Body	Island	
NM_006392;NR_003078;NR_	Body;TSS200;Body;TSS1500	S_Shore	
NM_019009;NR_029409	Body;TSS1500	N_Shore	
NM_006041;NR_026880	Body;TSS200	Island	CDMR
NR_027855;NM_001162407;	Body;Body;Body;Body	N_Shelf	RDMR
NM_021253;NR_024052;NR_	TSS1500;Body;Body;TSS1500	N_Shore	
NR_023318;NR_023319;NM_	TSS200;TSS200;TSS200	Island	
NM_032741;NM_006913;NR	TSS1500;Body;Body		
NM_001024660;NM_001024	1stExon;5'UTR;Body;5'UTR;1stExon		
NR_003501;NM_001083614	Body;Body		
NM_001145363;NR_026910;	1stExon;Body;5'UTR;1stExon;	Island	
NR_003263	TSS200	Island	
NR_029389	TSS1500	S_Shelf	
NR_002949;NR_027766	TSS200;TSS1500	N_Shore	
NR_021490;NR_021491;NR_(	Body;Body;Body	Island	
NR_030617	Body		
NR_031662;NM_032359	Body;TSS200	Island	
NR_028044;NR_003512;NM_	Body;Body;5'UTR;Body;TSS2(	Island	
NR_027117	Body	Island	
NR_026951	TSS200	S_Shore	
NR_002612	TSS200	Island	
NM_000968;NR_002440;NR_	Body;TSS200;TSS1500		
NR_026876	Body	N_Shore	DMR
NR_003111	TSS1500		
NR_015453;NM_001146069;	Body;TSS1500;TSS1500	S_Shore	
NR_002561;NR_000008;NR_(	TSS1500;TSS1500;TSS1500;TS	N_Shore	
NM_001165030;NR_028491;	TSS200;TSS200;TSS200	Island	
NM_001145049;NM_001142	5'UTR;TSS1500;Body;TSS1500;Body;5'UTR;TSS1500;Body		
NR_002184	TSS1500	S_Shore	
NR_027764	Body		
NM_033412;NR_024872;NR_	5'UTR;Body;Body	N_Shore	
NR_028040;NM_178831;NR_	Body;1stExon;Body;Body	Island	
NM_001141970;NR_024517;	TSS1500;TSS1500;TSS1500;TS	S_Shore	
NR_028044;NR_003512;NM_	Body;Body;5'UTR;TSS1500;Bo	S_Shore	
NR_002188	TSS1500	S_Shore	
NM_015913;NM_001136497	TSS1500;TSS1500;TSS1500;TS	N_Shore	
NR_026878	Body	Island	CDMR
NR_003142;NR_002326;NR_(	TSS1500;TSS1500;TSS1500;Bo	N_Shore	
NR_002582;NM_001007074;	TSS1500;TSS1500;TSS200;TSS	S_Shore	
NM_020914;NR_029376	Body;Body		
NR_026765	Body		
NR_002961;NR_003110	TSS1500;Body	S_Shelf	
NR_026981;NM_001003789;	Body;5'UTR;5'UTR;5'UTR;5'UT	N_Shore	
NR_026951	TSS200	S_Shore	

NM_001162505;NM_199203	Body;Body;Body;Body	Island	
NR_026883;NR_026885;NR_(	Body;TSS200;TSS200		
NR_003239	TSS200	N_Shore	
NM_014415;NR_024407	TSS200;Body	Island	
NM_001141945;NM_152872	TSS200;Body;Body;Body;Body	S_Shore	
NM_001300;NR_027653;NM_	5'UTR;Body;1stExon;1stExon;	Island	
NR_003075	TSS1500	S_Shore	CDMR
NR_027087	TSS1500		
NR_001578	Body	S_Shore	DMR
NR_028324	Body		
NR_002824	Body		
NM_001016;NR_002435;NR_	Body;TSS200;TSS1500	S_Shore	
NM_014415;NR_024407	TSS1500;Body	S_Shore	
NR_028334	TSS200	S_Shore	
NM_030819;NR_027399;NR_	5'UTR;Body;Body	Island	
NR_024610;NM_005340;NR_	TSS200;TSS200;TSS200	Island	
NM_001141969;NR_024517;	Body;Body;Body;Body	N_Shore	
NR_024034	Body	Island	
NR_024194;NR_024195;NM_	Body;Body;Body;Body;Body;5	S_Shore	
NR_030621	Body	Island	
NR_024581;NM_024701	TSS200;TSS200	Island	
NR_024568	Body	N_Shelf	
NR_027295;NR_026680	TSS1500;TSS1500	N_Shore	
NR_027103	TSS200		
NM_173627;NM_001164637	TSS1500;TSS1500;TSS1500;Bo	Island	
NR_002824	Body		
NR_030169	TSS1500	S_Shelf	
NR_026950	TSS1500	S_Shore	
NM_018177;NR_027277	5'UTR;TSS1500	S_Shore	
NM_052839;NR_027691;NM_	Body;Body;Body	S_Shore	
NM_030937;NM_001144868	Body;Body;Body;TSS1500;Bo	N_Shore	
NM_030573;NM_001008695	Body;Body;TSS1500;TSS1500	N_Shore	
NR_027451	TSS200	S_Shore	
NM_005510;NM_004197;NR_	5'UTR;TSS200;Body;TSS200	N_Shore	
NR_015444	Body		
NR_027399;NR_027398;NM_	TSS200;TSS200;TSS200	Island	
NR_033203;NR_033204;NR_(	Body;Body;Body;TSS1500;TS	N_Shore	
NR_026730	Body	Island	
NM_007349;NR_028090	TSS1500;Body	Island	
NR_002766;NR_003531;NR_(	TSS1500;TSS1500;TSS1500	N_Shore	
NR_026865;NM_030936	TSS1500;5'UTR	S_Shore	
NR_026826;NM_001145159;	TSS1500;TSS1500;5'UTR;TSS1	S_Shore	
NR_024014;NM_002081	TSS1500;Body	S_Shore	
NR_024100	TSS1500		

NR_027475;NR_027474;NM_	TSS1500;TSS1500;TSS1500;TS	S_Shore	
NR_029498;NM_005916;NM_	TSS1500;Body;Body;TSS200;TSS1500		
NM_030660;NM_001164781	5'UTR;5'UTR;Body;1stExon;5'	Island	
NM_002931;NR_029633	TSS1500;TSS1500	N_Shore	
NR_024019;NM_019057	TSS1500;TSS1500	S_Shore	
NR_024374	Body		
NR_024448;NM_153615	Body;TSS1500		
NR_026580;NM_007111	Body;Body	S_Shelf	
NR_015441	TSS200	Island	
NR_026052	TSS200	Island	
NR_029607;NR_027148	TSS1500;Body		
NR_028074;NM_014828;NM_	TSS200;TSS200;TSS1500;TSS2	Island	
NR_026895;NM_006544;NM_	Body;TSS1500;1stExon	S_Shore	
NM_207354;NR_030767	Body;Body	S_Shore	
NR_027051;NM_030573;NR_	Body;TSS1500;Body;TSS1500	Island	
NR_026867	TSS200	S_Shore	
NM_001142273;NM_015282	Body;Body;Body;TSS200	Island	
NR_027786	TSS200	N_Shore	
NR_003086	TSS1500	N_Shore	
NR_026541;NR_026564;NR_(	Body;Body;Body	S_Shore	
NR_027107	TSS1500	Island	DMR
NR_026676	Body	Island	
NM_178126;NR_026697;NM_	1stExon;Body;TSS200	Island	
NR_026785;NM_052924	Body;TSS1500	Island	
NM_206826;NM_014366;NM	Body;Body;Body;TSS1500	S_Shelf	
NR_028335;NM_024086	TSS1500;3'UTR		
NM_001141969;NR_024517;	Body;Body;Body;Body	N_Shore	
NR_030218	TSS1500		
NR_026580;NM_007111	Body;Body	Island	
NR_024024;NM_001130410;	TSS200;TSS200;TSS1500;TSS2	Island	
NR_027332;NM_000860;NM_	TSS200;TSS1500;TSS1500	S_Shore	
NM_032811;NM_032811;NR_	1stExon;5'UTR;Body	Island	
NR_024469	TSS1500	Island	
NR_002591;NM_006098;NM_	TSS1500;1stExon;5'UTR	Island	
NM_021259;NR_024453	Body;TSS1500	Island	
NM_006554;NR_027850	TSS200;TSS200	N_Shore	
NR_027428;NR_027430;NM_	TSS200;TSS200;Body;TSS200;	Island	RDMR
NM_004945;NM_001005362	TSS200;TSS200;TSS200;TSS20	Island	
NM_173627;NM_001164637	TSS1500;TSS1500;TSS1500;Bo	Island	
NR_024485;NR_024486	Body;Body	N_Shore	
NR_027626;NR_002174	TSS200;TSS1500	N_Shore	
NM_147195;NR_027294	Body;TSS1500	Island	
NR_002329;NR_002330;NM_	TSS200;Body;Body;Body	N_Shore	
NR_003313;NR_003312;NR_(	TSS1500;TSS1500;TSS1500		

NM_000520;NR_027262	1stExon;TSS200	Island	
NM_006087;NR_030585	Body;TSS200	Island	
NM_001011537;NM_001145	1stExon;1stExon;5'UTR;5'UTR	Island	
NR_024534;NM_005787;NM_	TSS1500;TSS1500;Body;TSS15	N_Shore	
NM_080604;NM_001146020	Body;Body;Body;Body;Body;E	N_Shelf	
NR_028044;NR_003512;NM_	Body;Body;5'UTR;5'UTR;Body	Island	DMR
NR_028058	Body		
NR_026786;NM_032263;NR_	Body;TSS1500;Body;Body;Bo	S_Shore	RDMR
NR_003512;NM_001007139;	Body;3'UTR;3'UTR;3'UTR	N_Shore	
NM_005313;NR_002318	TSS200;Body	N_Shore	
NR_003367	TSS1500	Island	
NM_001880;NR_030630	TSS1500;TSS1500	S_Shore	
NR_026781;NR_026780	Body;Body	S_Shore	
NR_030179;NR_029613	TSS200;Body	S_Shore	
NR_024042	TSS1500		
NR_003500	Body	N_Shore	
NR_015449;NR_023384;NR_(	Body;Body;Body	Island	
NR_030588;NM_017415	TSS200;Body		
NR_028436;NM_003347;NR_	TSS1500;TSS1500;TSS1500	N_Shore	
NR_002995;NR_004380	TSS1500;TSS200	Island	
NM_018227;NR_015439	TSS1500;Body	S_Shore	
NM_001130041;NM_003029	TSS1500;TSS1500;Body;Body	Island	
NR_002929	TSS1500	Island	
NM_014415;NR_024407	1stExon;Body	Island	
NM_001170687;NM_001170	Body;Body;Body;5'UTR;Body;	Island	
NR_003129;NM_006913;NM_	TSS1500;TSS1500;1stExon;5'UTR		
NR_003491	Body	S_Shore	DMR
NR_002971;NM_080686	TSS1500;5'UTR	S_Shore	
NR_026712;NM_012423	Body;Body	Island	
NM_052924;NR_026785	TSS200;TSS200	Island	
NR_027337	Body	Island	
NR_023938	TSS1500	N_Shore	DMR
NR_027242	Body	N_Shore	
NM_032846;NR_028074;NM_	Body;Body;TSS1500;TSS200	N_Shore	
NR_024547;NM_003876	TSS1500;TSS1500	S_Shore	
NM_182620;NM_001100595	Body;Body;TSS1500	N_Shelf	
NR_030205;NR_030206	Body;TSS1500		
NR_027463;NR_027462;NM_	Body;Body;TSS200	S_Shore	
NM_021238;NR_026806;NM_	5'UTR;TSS200;5'UTR;5'UTR	Island	
NR_015428	Body	Island	
NR_027078;NM_138736;NM_	TSS1500;1stExon;5'UTR;1stEx	Island	DMR
NR_023382	TSS1500	N_Shore	
NR_003512;NR_028044;NM_	Body;TSS1500;Body;5'UTR;TS	Island	
NR_028032	Body	Island	

NR_027382;NM_138412;NM_	TSS200;5'UTR;TSS200;TSS200	Island	
NR_003671;NM_007175	TSS1500;Body		
NR_024153;NR_015431	TSS1500;Body	Island	
NR_024527;NM_001142348;	TSS200;Body;Body;TSS200	Island	
NR_029380	Body	Island	DMR
NR_026926;NR_026923;NR_(	Body;TSS1500;Body	N_Shore	
NR_015411	Body	Island	
NR_003610	Body	Island	
NR_030174	TSS200		
NR_028133;NM_001145299;	TSS1500;TSS200;TSS200;TSS2	Island	
NM_198718;NR_028294;NM_	1stExon;Body;1stExon;Body;1	Island	
NM_001024380;NR_027638;	5'UTR;TSS1500;1stExon;TSS15	Island	
NM_012459;NM_003002;NR_	Body;TSS1500;Body	N_Shore	
NM_001141969;NR_024517;	5'UTR;Body;Body;5'UTR	Island	
NR_026971	TSS200	Island	
NR_027387	Body	Island	
NM_032885;NR_031603	Body;TSS1500		
NR_024430;NR_029671	Body;TSS200		
NR_027237	Body	N_Shore	
NM_001136473;NM_001136	5'UTR;5'UTR;Body;5'UTR		
NM_030573;NM_001008695	Body;Body;TSS1500;TSS1500	N_Shore	
NM_003927;NR_002970;NM_	Body;TSS1500;Body	N_Shore	
NM_001136123;NR_024245;	Body;Body;Body	S_Shore	
NR_027284;NR_026861;NR_(	Body;TSS200;TSS200	Island	DMR
NR_003266	TSS200	Island	
NR_021492;NR_027928;NM_	Body;TSS200;TSS200	Island	
NR_027301	Body	N_Shore	
NR_028484;NR_028483;NM_	Body;Body;TSS1500	Island	DMR
NR_024621;NM_139022;NM_	Body;TSS1500;Body		
NM_004860;NR_027463	TSS1500;Body	Island	
NR_024625;NM_001142931;	TSS200;TSS200;TSS200;TSS20	N_Shore	
NM_018177;NR_027277	5'UTR;Body	Island	
NR_026667	TSS1500		
NM_006000;NR_003063	TSS200;Body	Island	
NR_024383;NR_015436;NR_(	Body;Body;Body	Island	
NR_002187;NM_012470	Body;TSS1500	N_Shore	
NR_002307	Body	S_Shore	
NR_024367	TSS1500		
NR_015340	TSS1500		
NM_001130136;NR_027073;	5'UTR;TSS1500;TSS1500;TSS1	Island	
NR_003954;NM_024694	TSS200;Body		
NR_015454;NM_002359	Body;TSS1500	Island	
NR_024529;NR_000029;NR_(	TSS1500;TSS1500;TSS1500;5'	Island	
NM_015327;NR_026678;NM_	TSS1500;Body;TSS1500		

NM_152522;NR_024526	Body;Body	S_Shore	
NR_002578;NR_003942;NR_(	Body;TSS1500;TSS1500;TSS1!	N_Shore	
NM_019605;NR_024337	5'UTR;Body	Island	
NR_024568;NM_022818	Body;TSS1500	N_Shore	
NR_023348;NM_001481	TSS200;TSS200	Island	
NM_013309;NR_022014	TSS1500;Body	S_Shore	
NR_029375;NM_001099737;	TSS1500;TSS1500;Body	N_Shore	RDMR
NM_021080;NR_030385	5'UTR;TSS1500		
NR_027242	Body	Island	
NR_028102;NM_025264	Body;Body	N_Shore	
NR_030372;NM_000164	TSS200;Body	N_Shelf	
NR_031680;NM_015554	Body;5'UTR	Island	
NR_024042	TSS200		
NR_000015;NR_000024;NM_	TSS1500;TSS1500;TSS1500	N_Shore	
NM_001033568;NR_002222;	Body;TSS1500;Body;Body		
NR_001281	Body	N_Shore	RDMR
NR_026646	TSS200		
NM_014620;NM_018953;NR_	5'UTR;TSS200;Body	N_Shore	
NR_003149;NM_032119	Body;Body		
NM_003466;NM_013951;NM	Body;Body;Body;Body;Body;E	Island	
NM_005446;NM_001159554	TSS1500;TSS1500;TSS200	Island	
NR_024457	TSS200	Island	DMR
NR_026870	TSS1500		
NM_018043;NR_030691	Body;Body	Island	
NR_003667;NM_014471	Body;Body		
NM_006041;NR_026880	Body;TSS1500	Island	CDMR
NR_029712	Body	S_Shelf	
NR_024370;NR_015396;NM_	Body;TSS200;TSS1500	S_Shore	
NR_027399;NR_027398;NM_	TSS1500;TSS1500;TSS1500	S_Shore	
NR_024076	Body	N_Shore	
NM_016426;NR_024009	TSS200;TSS200	Island	
NR_026860;NR_026861;NR_(	Body;Body;TSS1500	N_Shore	
NM_176895;NM_003711;NM	5'UTR;1stExon;5'UTR;TSS200;	Island	
NM_001163484;NM_025230	5'UTR;5'UTR;Body;Body;5'UT	S_Shore	
NM_000528;NM_001099737	TSS1500;5'UTR;Body	N_Shore	
NR_003944;NM_032522;NR_	TSS1500;TSS1500;TSS200;TSS	N_Shore	
NM_001099737;NR_029375;	TSS200;TSS200;1stExon	Island	
NM_198256;NR_003093;NR_	TSS200;TSS200;TSS200;TSS20	S_Shore	
NR_030170	TSS200		
NR_023382	Body	Island	
NM_017444;NR_023360	Body;Body	Island	
NR_023319;NR_023318;NM_	Body;Body;Body	N_Shore	
NR_028504;NR_028502;NM_	TSS1500;TSS1500;Body;TSS1!	S_Shore	
NR_026750	Body	N_Shore	

NM_001100592;NR_027040;	5'UTR;TSS200;5'UTR;1stExon;	Island	
NR_028398;NR_028394;NR_(	Body;Body;Body;Body;Body;E	S_Shore	RDMR
NR_002754;NR_002755;NM_	Body;Body;TSS1500;TSS1500;	N_Shore	RDMR
NR_003082	TSS1500		
NR_001435	Body		
NR_028080;NM_032042;NM	Body;Body;Body;Body;TSS15(	Island	
NR_024553	Body	Island	DMR
NR_029374;NM_001166119;	TSS1500;Body;Body;Body;Bo	N_Shore	RDMR
NR_029434;NM_002892;NR_	TSS200;TSS1500;TSS200;TSS1	Island	
NM_001011537;NM_001145	TSS1500;5'UTR;TSS1500;TSS1	N_Shore	RDMR
NR_026704	TSS1500		
NM_005500;NR_027280;NM	Body;Body;Body;Body	S_Shore	
NM_152520;NR_031659	5'UTR;TSS200	N_Shore	
NM_005829;NR_023361	Body;Body	N_Shore	
NR_015374	Body	N_Shore	
NM_178126;NR_026697;NM	1stExon;Body;TSS200;5'UTR	Island	
NR_027283	TSS1500	Island	
NR_027790	TSS1500		
NR_029692	TSS200	Island	
NR_026968;NM_033625;NM	TSS200;TSS200;TSS200		
NM_001142327;NM_021145	5'UTR;TSS200;1stExon;1stExo	Island	
NR_030588;NM_017415	TSS200;Body		
NM_014596;NM_170783;NR	TSS1500;TSS1500;Body	N_Shore	
NR_027026;NR_027028	Body;Body		
NM_014620;NR_003084;NM	5'UTR;Body;1stExon;1stExon;	N_Shore	
NR_026906;NR_026905	Body;Body		
NM_001165031;NM_012145	Body;Body;Body	Island	
NR_029512	TSS200	N_Shelf	
NM_001033088;NR_028052	TSS1500;TSS1500	Island	
NR_026968;NM_033625;NM	TSS200;TSS200;TSS200		
NM_019057;NR_024019	Body;Body	N_Shore	
NM_178463;NR_029676	Body;TSS200		
NM_001159767;NM_020319	TSS200;TSS1500;TSS200;TSS2	Island	
NR_024246	TSS1500		
NM_014167;NR_033192;NM	TSS1500;TSS200;Body	Island	
NM_001142273;NM_015282	Body;Body;Body;TSS200	S_Shore	
NR_026751;NM_170783;NM	TSS200;1stExon;1stExon;5'UT	Island	
NM_019012;NR_026568	Body;Body	Island	
NM_016040;NR_030761;NM	TSS1500;TSS1500;TSS1500;Bo	S_Shore	
NR_024431;NR_024429;NR_(	Body;Body;Body	Island	
NR_024458;NM_003295	TSS1500;Body	Island	
NM_177524;NM_177525;NR	5'UTR;5'UTR;TSS1500;Body	Island	
NR_002770	Body		
NR_024240;NR_026751	Body;Body	S_Shore	

NR_027308;NM_005919;NM_	Body;5'UTR;TSS1500;Body	Island	
NR_028044;NR_003512;NM_	Body;Body;5'UTR;Body	Island	DMR
NR_003512;NR_028044;NM_	Body;TSS1500;Body;5'UTR;TS	Island	
NR_027276	Body	S_Shelf	
NR_003307;NR_003313;NR_(	Body;Body;TSS200		
NR_002952;NR_003697	TSS1500;Body	N_Shore	
NR_002835;NM_005328	Body;5'UTR	S_Shore	
NR_024538;NM_000850;NM_	Body;3'UTR;Body		
NM_001145269;NR_026879;	Body;Body;Body	S_Shore	
NR_024282	TSS200	Island	
NR_028339;NR_028340	TSS200;TSS200	S_Shore	
NR_026805	Body		
NR_024207	Body	N_Shore	
NR_026850	Body	Island	
NR_031592;NM_007065	TSS1500;TSS200	Island	
NR_027276	Body	S_Shelf	
NR_003272;NM_001042414;	Body;1stExon;5'UTR	Island	
NM_078473;NM_001024380	Body;5'UTR;TSS1500;TSS150(	N_Shore	
NM_004640;NR_003065;NM_	5'UTR;TSS200;5'UTR	N_Shore	
NM_031895;NR_030632	Body;TSS1500	N_Shore	
NR_002787	Body	S_Shore	
NR_004858;NM_133635;NR_	TSS1500;TSS1500;Body;TSS15	Island	
NR_026592	Body	Island	
NM_052924;NR_026785	TSS200;TSS200	Island	
NR_026914	Body	N_Shelf	
NM_001141969;NR_024517;	Body;Body;TSS1500;Body;Bo	N_Shore	
NM_207356;NR_024455	Body;TSS1500	N_Shore	
NR_003131	Body	Island	
NR_027092;NR_027093;NR_(	Body;TSS1500;Body	Island	DMR
NR_027840;NM_032288;NM_	Body;Body;5'UTR;TSS1500	Island	
NR_015436	Body	N_Shore	DMR
NR_033204;NR_033201;NR_(	TSS200;Body;TSS200;Body;Bo	Island	
NM_001025232;NR_027932;	3'UTR;TSS1500;TSS1500		
NR_003679	Body	Island	DMR
NR_003697	Body	N_Shelf	
NM_001143979;NR_030159;	TSS1500;TSS1500;5'UTR		
NM_100486;NM_016628;NM	1stExon;5'UTR;1stExon;5'UTR	Island	
NR_026693	Body	Island	
NR_027766;NR_002949	TSS1500;TSS1500	N_Shore	
NM_018320;NR_029192;NR_	TSS200;TSS1500;TSS200;TSS200		
NR_027308;NM_001145785;	Body;Body;Body;Body	Island	
NR_030717;NM_003083	Body;Body	Island	RDMR
NR_028340;NR_028339;NM_	Body;Body;3'UTR	N_Shelf	
NR_027294;NM_147195	Body;TSS1500	Island	

NR_004054;NM_014676;NM_	TSS1500;Body;Body		
NM_199189;NR_003141	TSS1500;TSS1500	N_Shore	
NM_014676;NR_004054;NM_	Body;TSS1500;Body		
NR_029402;NR_029403;NM_	Body;Body;Body;Body;Body;T	Island	
NR_026876	Body	Island	
NR_026804	Body	N_Shore	
NR_024154;NM_001105663	TSS1500;TSS1500	N_Shore	
NR_002754;NR_002755;NM_	Body;Body;TSS1500	S_Shore	
NR_026864	Body	Island	
NR_015370;NR_002922	Body;TSS1500	Island	
NM_152872;NM_152871;NM	Body;Body;Body;Body;Body;5	Island	
NM_001656;NM_001093755	5'UTR;TSS1500;5'UTR;TSS150	Island	
NR_015441	TSS200	Island	
NR_002894	Body	Island	
NR_027064	Body	Island	DMR
NR_028044;NR_003512;NM_	Body;Body;5'UTR;5'UTR;Body	Island	DMR
NR_026781;NR_026780	Body;Body	Island	
NM_001159767;NM_020319	TSS200;TSS1500;TSS200;TSS2	Island	
NR_003296	TSS1500		
NR_030175	TSS200		
NM_138638;NR_028130;NM_	Body;Body;TSS1500;Body;Bo	N_Shore	
NR_002791	Body	Island	
NM_152872;NM_152871;NM	Body;Body;Body;Body;Body;5	Island	
NM_004640;NR_003065;NM_	5'UTR;TSS1500;5'UTR	N_Shore	
NR_031666	Body	Island	DMR
NR_027653;NM_001160124;	Body;Body;Body;Body	N_Shore	
NM_000984;NR_000013	Body;TSS200	S_Shore	
NR_026691;NM_001558	Body;Body	Island	
NM_007294;NM_007299;NR	5'UTR;5'UTR;TSS200;5'UTR;1s	N_Shore	
NR_026698;NM_002264	Body;5'UTR	N_Shore	
NR_030600	TSS1500		
NM_022818;NR_024568	Body;TSS1500	Island	
NM_032410;NR_027669;NM_	Body;TSS1500;TSS1500;TSS15	Island	
NM_001362;NR_031649	TSS200;TSS1500	Island	
NM_012322;NR_024466;NM_	TSS200;Body;5'UTR;5'UTR	Island	
NR_026732;NR_026731	Body;Body	S_Shore	
NR_033203;NR_033205;NR_(	Body;Body;Body;Body;Body;B	Island	
NR_001282	Body	Island	
NM_005328;NR_002835;NM_	1stExon;Body;5'UTR	S_Shore	
NR_024282	TSS1500	Island	
NR_027282;NM_152644	Body;TSS200	Island	DMR
NR_015357	Body	N_Shore	
NM_015916;NR_024552;NM_	5'UTR;Body;1stExon	Island	
NR_026901	Body	Island	

NR_024595	Body	N_Shore	
NM_003626;NM_177423;NR_	Body;Body;TSS1500		
NM_030803;NM_017974;NR_	Body;Body;Body		
NR_027622;NR_024409;NM_	TSS200;TSS200;1stExon	Island	
NR_026860;NR_026861;NR_(	Body;Body;TSS200	N_Shore	DMR
NR_003061	Body		
NM_003391;NR_024047	Body;Body	N_Shore	
NR_029380	Body	Island	DMR
NR_028514;NM_130766;NM_	TSS1500;5'UTR;5'UTR;Body	Island	
NR_028050;NR_028051;NR_(	Body;Body;Body;Body	N_Shore	
NR_024600;NM_138929;NR_	Body;3'UTR;Body;3'UTR	S_Shelf	
NM_001130136;NM_001130	5'UTR;5'UTR;TSS1500;1stExoı	Island	
NR_024405;NR_024403	TSS1500;TSS1500	N_Shore	
NM_021953;NM_202002;NR_	5'UTR;5'UTR;TSS1500;TSS150	Island	
NR_026903	Body	N_Shelf	
NM_033228;NM_024941;NM	Body;TSS1500;Body;Body;TSS	N_Shore	
NR_033203;NR_033204;NR_(	Body;Body;Body;TSS1500;Bo	S_Shore	DMR
NR_026751	Body		
NR_003111	TSS1500		
NR_027816;NM_152744	Body;Body		
NR_024596;NM_016401;NR_	TSS1500;TSS200;TSS200;TSS200		
NR_024409;NM_003262;NR_	Body;TSS1500;Body	N_Shore	
NR_029697	TSS1500	N_Shore	CDMR
NR_027489;NM_001159377;	TSS1500;TSS1500;TSS1500;TS	Island	
NR_024370;NR_015396;NM_	TSS200;TSS1500;TSS1500	S_Shore	
NR_003932	TSS1500		
NR_029865;NR_029866	TSS200;TSS1500		
NM_015363;NM_001146327	TSS200;TSS200;TSS200;TSS2C	Island	
NM_016426;NR_024009	Body;TSS1500	Island	
NR_024349	Body	Island	
NR_026804;NM_016531	Body;TSS1500	Island	
NM_001135629;NM_001135	TSS1500;TSS1500;TSS1500;TS	N_Shore	
NR_015399	Body		
NM_001163391;NR_028077	TSS1500;TSS1500	S_Shore	
NM_001134340;NR_027329;	TSS1500;Body;Body;Body;TSS	S_Shore	
NR_027270	TSS200	S_Shore	
NM_001165415;NM_001165	5'UTR;TSS1500;5'UTR;Body;5	Island	
NM_002136;NR_002944;NM_	TSS1500;TSS1500;TSS1500;5'	Island	
NR_024114;NM_175888	TSS1500;TSS1500	N_Shore	
NR_027368;NM_006627	Body;Body	S_Shore	
NR_027345;NR_027346	TSS200;TSS200		
NR_027774;NR_027782;NM_	TSS1500;TSS1500;TSS1500	S_Shore	
NM_001160300;NM_052839	TSS1500;TSS1500;TSS1500	Island	
NR_024260;NM_015509	TSS200;TSS200	N_Shore	

NM_001114632;NM_005090	TSS200;TSS200;TSS200	Island	
NM_019094;NM_199040;NR_	Body;Body;Body	S_Shore	
NR_024233;NM_020228;NM_	Body;TSS200;TSS200	Island	
NR_002818	TSS200	Island	
NM_021095;NR_028323	5'UTR;Body	N_Shore	CDMR
NR_024627	TSS1500	Island	
NR_003929	TSS200		
NR_003955	Body	S_Shore	
NR_027463;NM_004860	Body;TSS200	Island	
NM_002402;NM_177524;NM	TSS1500;5'UTR;5'UTR;TSS150	Island	
NR_026992	Body		
NM_145039;NR_003228;NM_	3'UTR;TSS1500;1stExon;TSS1!	N_Shore	
NR_027255;NM_001080395	TSS1500;Body	N_Shore	
NM_198595;NM_001134647	Body;Body;Body	N_Shelf	
NM_018371;NM_001130518	Body;Body;Body	Island	
NM_020456;NR_023351;NM_	TSS200;TSS200;TSS200	Island	
NM_018412;NR_002329;NR_	TSS200;TSS1500;Body;TSS20(	N_Shore	
NM_006317;NR_027253	5'UTR;TSS1500	Island	DMR
NM_006195;NR_024123;NM_	1stExon;Body;TSS1500;Body	Island	
NR_003246	TSS200		
NR_004862;NM_001039693;	TSS200;TSS200;Body	Island	
NM_024506;NM_001008910	5'UTR;TSS1500;TSS1500	N_Shore	
NR_003038;NR_003044;NR_(	Body;TSS1500;TSS1500	Island	
NR_003093;NM_198256;NR_	Body;Body;Body;Body;Body	N_Shore	
NR_028495;NR_028494;NR_(	TSS1500;TSS1500;TSS1500;TS	S_Shore	
NR_004401	Body	N_Shore	
NM_001144943;NM_001142	TSS1500;TSS1500;TSS1500;TS	N_Shore	
NR_024271	TSS200	S_Shore	
NM_000528;NM_001099737	TSS1500;5'UTR;Body	N_Shore	
NM_001042512;NM_001017	Body;Body;Body;Body;Body;Body;Body		
NR_027156	Body	Island	
NR_029583	TSS1500		
NM_053005;NM_001004325	Body;1stExon;Body		
NR_024040;NM_018371	Body;5'UTR	S_Shelf	
NR_026854	Body	S_Shore	
NR_030176;NR_029704	TSS1500;TSS200		
NR_002824	Body		
NM_005484;NM_001042618	Body;Body;TSS1500		
NR_024435;NR_024434;NM_	Body;Body;TSS1500	Island	
NR_002971;NM_080686	TSS1500;Body	S_Shore	
NR_001566	TSS1500	S_Shore	
NR_024056;NR_024057;NR_(	TSS1500;TSS1500;TSS200;TSS	Island	
NR_026838;NR_026840;NR_(	TSS200;TSS200;TSS200;5'UTR;1stExon;TSS200;TSS200		
NR_002974;NM_014949	TSS1500;Body		

NR_024469;NM_004584	Body;TSS1500	N_Shore	
NR_026893	TSS1500	S_Shore	
NR_026597	TSS200	Island	
NM_001142587;NM_001142	TSS1500;TSS1500;TSS1500;Bo	N_Shore	
NM_138797;NR_030395	TSS1500;TSS200	S_Shore	
NR_024332;NM_017739	Body;TSS1500		
NM_024721;NR_024360	TSS1500;Body	N_Shore	CDMR
NM_018022;NM_001136217	TSS1500;TSS200;TSS200;TSS2	N_Shore	
NM_001136123;NM_018121	TSS200;TSS200;TSS200	N_Shore	
NM_001011537;NM_001145	TSS1500;5'UTR;TSS1500;TSS1	N_Shore	RDMR
NM_016531;NR_026804	5'UTR;TSS200	Island	
NR_003654;NR_004859	TSS1500;TSS1500	N_Shore	
NR_033203;NR_033204;NR_(	Body;Body;Body;Body;TSS20(	Island	
NR_015448	Body		CDMR
NR_003660	TSS200	Island	
NR_027430;NR_027428;NM_	Body;Body;Body;Body;Body;E	Island	
NR_004397	TSS1500	Island	
NM_001145374;NR_026930;	Body;Body;Body;Body;Body	N_Shore	
NR_027103	TSS200		
NR_023319;NR_023318;NM_	Body;Body;Body	N_Shore	
NR_003936	TSS200	Island	DMR
NR_001298	TSS1500		
NR_029856	TSS200		
NR_027126;NR_027125;NM_	Body;Body;Body		
NM_001143771;NM_001143	TSS200;TSS200;TSS200;TSS2C	Island	
NM_016531;NR_026804	TSS200;Body	Island	
NR_024488	Body	Island	
NR_029411	Body	Island	
NR_027270	TSS1500	S_Shore	
NR_027049	TSS1500	N_Shore	
NR_029841;NR_030389	TSS1500;TSS200		
NR_004053;NM_003106	Body;TSS1500	N_Shore	
NM_206826;NM_014366;NM	Body;Body;Body;TSS1500		
NR_002995;NR_004380	TSS1500;TSS200	Island	
NR_003581;NR_003582	TSS1500;TSS1500		
NR_023313;NR_027271;NM_	Body;TSS200;5'UTR;Body	S_Shore	
NR_026583	Body		
NR_026587;NM_001143943;	Body;Body;Body;Body;Body	Island	
NR_027476;NM_032750;NM	TSS200;TSS200;TSS1500;TSS2	N_Shore	
NR_026667	Body		
NR_026693	Body	S_Shelf	
NR_028387;NM_004761	Body;Body	N_Shelf	
NR_001298	Body		
NM_001146178;NM_016291	TSS1500;TSS1500;TSS1500;TS	S_Shore	

NR_024153;NR_015431	TSS200;Body	Island	
NM_001079522;NR_027123;	TSS1500;Body;TSS1500;TSS15	N_Shore	
NR_027393	Body	Island	
NR_024596;NM_016401;NR_	TSS1500;TSS200;TSS200;TSS200		
NR_029380;NM_006562	TSS200;TSS1500	Island	DMR
NR_029373;NR_029374	TSS1500;Body	Island	RDMR
NM_001164246;NM_017847	TSS1500;TSS1500;Body;TSS15	Island	
NM_018280;NR_027034	TSS200;Body	Island	
NR_003099;NM_021148	TSS200;TSS200		
NM_018840;NR_026562;NM_	TSS1500;TSS1500;TSS1500	N_Shore	
NR_002799;NM_002106	TSS1500;Body	Island	
NR_027790	TSS200		
NR_024271	TSS1500	S_Shore	
NR_027762;NM_015014;NM_	TSS1500;TSS1500;TSS1500	S_Shore	
NR_026951	Body	N_Shore	
NM_014941;NR_002323	TSS1500;TSS1500	Island	
NR_002605;NR_002612	Body;Body	S_Shore	
NR_026950	Body	Island	
NR_003561;NR_027768	TSS1500;TSS1500	S_Shore	
NR_024079	TSS1500	S_Shore	
NR_004401;NM_145807	Body;3'UTR	Island	
NM_001040144;NM_001039	TSS1500;TSS1500;TSS1500;TS	Island	
NR_027927;NM_014296	TSS1500;Body	S_Shore	
NR_026719	Body	N_Shore	
NM_004071;NR_027855;NM_	5'UTR;Body;Body;Body	N_Shelf	RDMR
NR_003099;NM_021148	TSS1500;TSS1500		
NR_026956	TSS200		
NR_026997	Body	Island	
NR_001590	TSS200	N_Shore	
NR_027671;NM_020120;NM_	Body;1stExon;5'UTR	Island	
NM_012230;NR_029411;NM_	3'UTR;Body;Body		
NR_024627	TSS1500	Island	
NM_021131;NM_178000;NM	5'UTR;TSS200;TSS200;TSS150	Island	
NR_026717	TSS1500	N_Shelf	
NM_152410;NM_001080378	Body;Body;Body;Body		
NM_001130136;NR_027073;	5'UTR;TSS200;TSS1500;TSS15	Island	
NM_001170634;NR_028388;	TSS1500;TSS1500;TSS1500;TS	N_Shore	
NM_001031738;NM_153342	5'UTR;5'UTR;Body	Island	
NM_005328;NR_002835	TSS1500;Body	S_Shore	
NR_027786	TSS1500	N_Shore	
NM_100486;NM_016628;NR_	Body;Body;Body	Island	
NM_001142348;NM_001142	Body;5'UTR;Body;Body	N_Shore	CDMR
NR_003579	Body	Island	
NR_002710	Body	Island	

NR_024623;NR_024622	Body;Body	S_Shelf	
NR_015357	Body	S_Shore	
NR_027908	TSS200	S_Shore	
NR_029623	TSS200	Island	
NM_001280;NR_027271;NR_	TSS1500;Body;TSS1500;TSS1!	Island	
NR_027701	Body		
NR_004401;NM_001352	TSS1500;TSS200	Island	
NR_033203;NR_033204;NR_(	Body;Body;Body;TSS1500;Bo	N_Shore	
NR_027276	Body	S_Shore	
NR_027051;NM_030573;NR_	Body;TSS1500;Body;TSS1500	S_Shore	
NR_029401	TSS1500	S_Shore	
NR_002141	Body		
NR_027397;NM_001146039;	TSS200;TSS200;TSS200		
NR_015346;NM_001114632;	Body;Body;Body	S_Shelf	
NR_001593;NM_000980	TSS200;TSS200	Island	
NM_018489;NR_027023	TSS1500;Body	S_Shore	
NR_024573;NM_032484;NM_	TSS200;5'UTR;1stExon;1stExon;5'UTR;1stExon;5'UTR		
NR_000015;NR_000024;NM_	TSS1500;TSS1500;TSS1500	N_Shore	
NR_026826;NM_001145159;	TSS1500;TSS1500;5'UTR;1stE:	S_Shore	
NM_018320;NR_024148;NR_	Body;Body;TSS1500;Body		
NR_027320	TSS200	S_Shore	
NR_024343	Body		
NR_024621;NM_139022;NM_	Body;TSS1500;Body		
NR_003239;NR_002972	TSS200;TSS1500	Island	
NR_024253	Body	N_Shelf	
NM_175888;NR_024114	TSS200;TSS200	N_Shore	
NM_199191;NM_199193;NM	TSS1500;TSS1500;TSS1500;TS	N_Shore	
NR_024009;NM_016426	TSS1500;5'UTR	Island	
NM_153264;NR_022012;NM_	5'UTR;Body;1stExon	Island	
NM_001280;NR_027271;NR_	TSS1500;Body;TSS1500;TSS1!	Island	
NM_212465;NM_173088;NR_	Body;TSS1500;Body;Body;Body;Body;Body		
NM_018489;NR_030282	Body;TSS1500		
NM_001145783;NM_134440	Body;TSS1500;Body;5'UTR;Bo	N_Shore	
NM_001142464;NR_024551;	TSS200;TSS1500;TSS200;TSS2	S_Shore	
NR_023350;NM_004766	TSS1500;TSS1500	Island	
NR_027816;NM_152744	Body;Body		
NR_027816;NM_152744	Body;Body		
NR_003582;NR_003581	TSS200;TSS200		
NR_003042;NM_004582	TSS1500;TSS1500		
NM_024506;NM_001008910	5'UTR;TSS1500;1stExon;TSS1!	Island	
NR_015447	Body	S_Shore	RDMR
NR_024117	TSS200	Island	
NR_029192	Body		
NR_000034	TSS1500	Island	

NR_004383;NM_033377	TSS1500;Body		
NR_026892	TSS200		
NR_002582;NM_001007074;	TSS1500;TSS1500;TSS1500;TS	S_Shore	
NR_003334;NR_003335	Body;TSS1500		
NR_026926;NR_026925	Body;Body		
NR_027410	Body	Island	DMR
NR_002818	Body	Island	
NM_004824;NR_026590;NM_	Body;Body;Body;Body		
NR_026736;NM_017906;NM_	Body;TSS1500;TSS200;TSS20	Island	
NR_029869	TSS1500		
NM_001416;NR_002918	Body;TSS1500	Island	
NR_031646;NM_004734	Body;Body		
NR_026866	TSS1500		
NR_026965	TSS200		
NR_028287	Body	N_Shelf	
NR_026776;NR_026775	TSS200;Body	S_Shore	
NR_003955	Body	Island	
NM_152410;NM_001080378	Body;Body;Body;Body		
NM_018489;NR_027023	TSS1500;Body	Island	
NR_028493;NM_020466;NR_	TSS200;Body;TSS200;TSS200	Island	
NR_031593;NM_198552	TSS200;Body		
NR_029681;NM_199424;NM_	TSS200;Body;Body		
NR_027258	TSS200	Island	
NR_024444	TSS1500	S_Shore	
NM_194325;NR_024018;NM_	5'UTR;Body;5'UTR;5'UTR	Island	
NR_026997	Body	Island	
NM_001031716;NR_024415	TSS1500;TSS1500	N_Shore	
NM_013291;NR_031600	Body;TSS200	S_Shelf	
NM_004945;NR_029586;NM_	Body;TSS1500;Body;Body;Body		
NM_001137605;NR_024427;	5'UTR;Body;TSS200;1stExon;TSS200;Body;5'UTR		
NR_027165;NR_027168;NR_(	Body;Body;Body;Body;Body;E	Island	
NR_003330;NR_003331	Body;TSS1500		
NM_001142623;NM_032484	Body;Body;Body;Body		
NR_027701	Body		
NR_015433	TSS1500	N_Shore	
NR_026837;NM_013381;NR_	Body;1stExon;Body	Island	
NR_033203;NR_033204;NR_(	Body;Body;Body;Body;TSS20	Island	
NR_026791	Body	Island	
NM_001009944;NR_030646;	Body;TSS1500;Body	Island	
NR_030193	TSS1500		
NM_014053;NR_027286;NR_	1stExon;TSS1500;TSS1500	Island	
NR_002932	Body		
NR_026762	TSS1500	N_Shore	
NM_006041;NR_026880	Body;Body	Island	

NM_001100592;NM_145230	Body;Body;TSS1500	S_Shore	
NR_003110	Body	Island	
NM_153264;NR_022012	TSS1500;TSS1500	N_Shore	
NM_001242;NR_015382	TSS1500;Body		
NR_024458;NM_003295	TSS1500;Body	N_Shore	
NM_001145354;NR_015431	TSS200;TSS1500	Island	
NM_001607;NR_024024;NM_	1stExon;Body;5'UTR;TSS1500	Island	
NM_015412;NR_027794;NR_	TSS200;TSS200;TSS200	Island	
NR_003300	TSS1500		
NM_001146184;NM_001146	TSS1500;TSS1500;TSS1500;TS	S_Shore	RDMR
NR_003099;NM_021148	TSS200;TSS200		
NR_024463	TSS200		
NM_032026;NM_001146160	Body;5'UTR;Body;TSS1500	N_Shore	
NM_014620;NR_003084	5'UTR;Body	Island	
NR_026789	Body		
NM_001145873;NM_001768	5'UTR;TSS1500;TSS1500;TSS1	S_Shore	RDMR
NR_026597	Body		
NR_001591	Body	Island	
NR_024240;NR_026751	Body;Body	Island	
NR_027822	Body	Island	DMR
NR_024623;NM_013329;NM_	Body;TSS1500;TSS1500;TSS15	S_Shore	
NM_020707;NR_027753	5'UTR;TSS1500		
NR_033258;NM_014795;NM_	Body;5'UTR;1stExon;1stExon;	S_Shelf	DMR
NM_001145775;NR_027117	5'UTR;Body	Island	
NR_027653;NM_001160125;	TSS1500;TSS1500;TSS1500;TS	Island	
NM_153262;NR_027458;NR_	Body;Body;Body;Body;Body;B	Island	DMR
NR_028435;NM_001164710;	Body;Body;Body;Body;Body		
NR_027034;NM_018280	Body;TSS1500	S_Shore	
NR_024249	TSS1500	S_Shore	
NR_024383;NR_015436;NR_(	Body;Body;Body	N_Shore	CDMR
NM_001128128;NM_001128	1stExon;5'UTR;Body;Body;Bo	Island	DMR
NR_003512;NM_001007139;	Body;TSS1500;Body		RDMR
NM_014463;NR_027299;NM_	TSS1500;Body;Body;Body	N_Shore	
NR_026921	TSS1500	S_Shore	
NR_031658	TSS1500		
NR_024239;NM_001136111;	Body;Body;Body;Body;Body;Body		
NM_018227;NR_015439	Body;TSS1500	Island	
NR_003063;NM_006000	TSS200;Body	N_Shore	
NR_027405;NM_006636	Body;Body	S_Shore	
NR_026590	Body	N_Shore	RDMR
NR_029405	TSS200		
NR_024565;NM_001166012;	TSS200;5'UTR;TSS200;1stExon	Island	
NR_003102;NM_015450;NR_	Body;1stExon;Body;Body;5'U	Island	
NM_138638;NR_028131;NM_	TSS1500;TSS1500;TSS1500;TS	S_Shore	

NM_019009;NR_029409	Body;TSS1500	Island	
NM_001040437;NM_001040	5'UTR;5'UTR;1stExon;TSS200;	S_Shore	
NM_032853;NR_024247	5'UTR;TSS1500	S_Shore	RDMR
NR_024472	Body		
NM_019116;NM_001083614	TSS200;TSS200;TSS200	Island	
NM_000037;NR_030161;NM_	3'UTR;TSS200;Body;3'UTR;Body;Body;Body;Body;Body		
NR_027908	Body	N_Shelf	
NM_004860;NR_027463	Body;TSS200	N_Shore	
NR_027812;NR_027811;NM_	Body;Body;Body	N_Shore	
NM_012459;NM_003002;NR_	TSS1500;Body;TSS1500	S_Shore	
NR_003276	Body	Island	
NM_001141970;NR_024517;	TSS1500;TSS1500;TSS1500;TS	S_Shore	
NR_024547;NM_003876	Body;Body	Island	
NR_001591	Body	S_Shore	
NM_001007593;NR_027400;	Body;Body;Body	S_Shore	
NR_000013;NM_000984	TSS1500;TSS200	Island	
NR_027389;NM_004970;NM_	TSS200;TSS1500;TSS1500	S_Shelf	
NR_029865;NR_029864;NR_(	TSS1500;TSS200;TSS1500		
NM_021643;NM_021643;NR_	1stExon;5'UTR;Body	Island	
NR_027789;NR_027788	TSS1500;TSS1500	S_Shore	
NR_026766;NM_005378	TSS200;5'UTR	Island	
NR_024279;NM_015001	TSS1500;Body	Island	
NM_005663;NR_030641	Body;TSS1500	S_Shore	
NR_026678;NM_015327	TSS1500;Body		
NR_024591	TSS200	Island	
NR_002305	Body	Island	
NR_001435	Body	S_Shore	
NM_001010982;NM_001145	1stExon;TSS200;5'UTR;TSS20(	Island	
NR_015424	TSS1500	Island	
NR_023391	Body		
NR_026956	TSS1500		
NM_001722;NR_029714	Body;TSS1500	S_Shore	
NR_027284;NR_026861;NR_(	Body;TSS1500;TSS1500	Island	DMR
NR_027768;NR_003561	Body;TSS200	Island	
NM_001144942;NM_001144	TSS200;Body;Body;Body;TSS2	Island	
NM_001170689;NM_001170	TSS1500;TSS1500;TSS1500;TS	N_Shore	
NM_001102367;NM_001102	5'UTR;5'UTR;5'UTR;TSS1500;5'UTR;5'UTR		
NR_015422	Body	S_Shore	
NR_024148;NR_029192;NM_	Body;TSS1500;5'UTR;Body;1stExon		
NM_000967;NR_000028;NM_	Body;TSS200;Body		
NR_026761	Body	Island	
NM_000969;NR_000006	Body;TSS1500		
NR_023380	Body		
NR_024584	Body		

NR_003699	Body	Island	DMR
NR_030199	TSS1500		
NR_027368;NM_006627	Body;Body	Island	
NM_001012957;NM_001164	Body;Body;Body;Body;Body		
NR_027399;NR_027398;NM_	TSS200;TSS200;TSS200	Island	
NR_027238	TSS1500		
NR_002767;NM_018064	TSS200;Body	N_Shore	
NM_000441;NR_028137	Body;TSS200	Island	
NR_003272;NM_001042414	TSS1500;TSS1500	S_Shore	
NM_021004;NR_023921;NR_	TSS1500;Body;Body;Body;Bo	N_Shore	
NR_024270	Body	Island	
NR_024383;NR_015436	Body;Body	Island	
NR_027435;NM_020801	Body;Body	Island	
NR_026751	Body		
NR_027790;NR_027791	Body;Body		RDMR
NM_152720;NM_001146099	TSS1500;TSS200;TSS200;TSS2	S_Shore	
NR_027653;NM_001160124;	Body;Body;Body;Body	Island	
NR_033186;NM_207467	Body;5'UTR	S_Shore	
NR_004053	Body	Island	DMR
NM_005047;NR_024408	TSS200;TSS200	Island	
NR_027633;NM_001009877;	Body;Body;Body	Island	
NR_023385;NR_023384;NR_(	TSS200;TSS200;TSS200	N_Shore	
NR_024178	Body		
NR_028295	TSS200	S_Shore	
NR_028044;NR_003512;NM_	Body;Body;5'UTR;Body	Island	DMR
NR_027703;NM_052988;NM	TSS200;TSS200;TSS200;TSS2C	Island	
NM_022906;NR_022007	5'UTR;TSS1500	S_Shore	
NR_026865;NM_030936	Body;TSS1500	Island	DMR
NM_022103;NR_030740	TSS1500;TSS1500	S_Shore	
NR_002728;NM_000218;NM	TSS1500;Body;Body	S_Shore	
NM_004197;NM_032454;NM	Body;Body;TSS1500;Body	Island	
NM_005798;NM_001007278	5'UTR;5'UTR;5'UTR;5'UTR;1st	S_Shore	
NR_015428	TSS200	S_Shore	
NM_052879;NM_001170803	Body;Body;Body;Body;Body;E	Island	
NR_024447	TSS1500		
NR_003512;NM_001007139;	Body;3'UTR;3'UTR;3'UTR	Island	
NR_026676	TSS1500	N_Shore	
NR_031577	TSS1500		
NR_002328;NM_005105	Body;3'UTR	S_Shelf	
NR_031680	Body	Island	
NM_018917;NM_032092;NM	Body;Body;Body;Body;Body;E	Island	
NR_026658;NR_026655	TSS200;Body	Island	
NR_027024	Body		
NR_027451	Body	Island	

NR_015358	TSS200	Island	DMR
NR_028032	Body	Island	
NM_001130041;NM_003029	TSS1500;TSS1500;Body;Body	S_Shore	
NM_001416;NR_002918	Body;TSS200	S_Shore	RDMR
NM_001146184;NM_001146	TSS1500;TSS1500;TSS1500;TS	S_Shore	RDMR
NM_014815;NM_001079518	Body;Body;TSS1500		
NR_002201;NM_002707;NM_	Body;Body;Body	N_Shore	
NM_025264;NR_028102	TSS1500;TSS1500	S_Shore	
NR_027500;NM_001037806;	TSS200;1stExon;5'UTR;TSS20	Island	
NM_001145438;NM_001145	Body;Body;Body;Body;Body;Body;Body;Body		
NR_031706;NM_022720	TSS1500;5'UTR	N_Shore	
NM_183048;NR_024594;NM_	Body;TSS1500;Body;Body		
NM_001164821;NM_024770	TSS1500;5'UTR;TSS1500;TSS1	N_Shore	
NR_024008	Body	N_Shore	RDMR
NM_001142464;NR_024551;	TSS200;TSS1500;TSS200;TSS2	S_Shore	
NM_001042424;NM_133330	Body;Body;Body;TSS200;Body		
NR_024047;NM_003391	TSS200;TSS200	Island	DMR
NM_016464;NM_001161454	5'UTR;TSS1500;TSS1500;Body	S_Shore	
NR_029380;NM_006562	TSS200;TSS1500	Island	DMR
NM_001161587;NR_027763;	Body;Body;Body	S_Shelf	
NM_001003656;NR_033191;	5'UTR;Body;Body	N_Shore	
NR_024455;NM_207356	Body;TSS1500	Island	
NM_018412;NM_021908;NR_	Body;Body;TSS1500		
NR_026572;NM_001146209;	TSS1500;Body;TSS1500;Body	S_Shore	
NR_027048;NR_027047	TSS1500;TSS1500		
NM_017975;NR_003105;NM_	TSS200;TSS200;TSS1500		
NR_024423;NR_024424	Body;TSS1500	N_Shore	
NR_024270	TSS200	Island	
NR_027816;NM_152744	Body;Body		RDMR
NR_026776;NR_026775	Body;Body	Island	
NM_021259;NR_024453	TSS1500;Body	S_Shore	RDMR
NR_024237;NM_001136003	Body;5'UTR	Island	RDMR
NM_198468;NR_031679	5'UTR;Body	N_Shore	
NM_001042383;NR_024400;	5'UTR;TSS1500;5'UTR;TSS150	S_Shore	RDMR
NR_002754;NR_002755;NM_	Body;Body;TSS1500;TSS1500	N_Shore	RDMR
NM_000967;NR_002439;NM_	TSS1500;TSS1500;TSS1500	S_Shore	
NR_024277;NM_020133	TSS1500;Body		
NR_026777	Body	Island	
NR_031738;NM_173665;NM_	TSS200;5'UTR;5'UTR		
NM_024033;NR_024185;NR_	1stExon;Body;Body;5'UTR	Island	
NR_027345;NR_027346	TSS200;TSS200		
NM_032271;NR_002736	TSS1500;TSS200	Island	
NR_002594	Body	N_Shore	
NR_002174;NR_027626	Body;Body	N_Shore	

NM_006718;NM_002656;NM	TSS1500;TSS1500;5'UTR;TSS1	S_Shore	
NR_027070	TSS200		
NR_024584	TSS1500	S_Shore	
NR_027331	TSS200		
NR_026791	TSS1500	N_Shore	
NR_002727;NM_021026	Body;Body		
NR_003525	TSS200	Island	
NM_001135654;NM_003819	Body;Body;Body;Body		
NR_003713	TSS1500	S_Shore	
NR_015410	TSS1500	Island	
NR_026875	TSS200	Island	
NR_027481	Body	Island	
NM_171829;NM_171830;NM	Body;TSS200;TSS1500;Body;Body;Body		
NM_001165415;NM_005566	TSS200;TSS200;TSS200;TSS20	N_Shore	
NM_001779;NR_026665;NM_	Body;Body;Body	N_Shelf	
NR_024436	TSS1500		
NR_029434;NR_029435;NM_	TSS1500;TSS1500;TSS200;TSS	Island	
NR_015447	Body	S_Shore	RDMR
NM_001145714;NM_001145	3'UTR;3'UTR;Body;3'UTR		
NR_001283;NM_001008496	TSS1500;5'UTR	Island	
NM_001005911;NM_001005	5'UTR;5'UTR;5'UTR;5'UTR;Bo	N_Shelf	
NR_027716;NM_177925	TSS200;TSS200	N_Shore	
NR_030349;NM_004664	TSS1500;Body	Island	DMR
NM_006887;NR_027251	TSS1500;Body	Island	
NR_015431;NR_024153	Body;Body		
NM_001144012;NM_015959	3'UTR;3'UTR;Body;TSS1500	N_Shore	
NR_030724;NM_020383;NM_	TSS1500;TSS1500;TSS1500	S_Shore	
NR_027692;NM_002636;NM_	TSS1500;TSS1500;TSS1500	N_Shore	
NR_028338;NM_147686;NM_	Body;5'UTR;5'UTR		
NR_024455	Body	S_Shelf	
NM_001126339;NM_001126	1stExon;5'UTR;5'UTR;Body;1s	Island	
NM_177524;NM_002402;NM	5'UTR;TSS200;5'UTR;TSS1500	Island	
NR_030765;NM_001167867;	TSS200;TSS1500;TSS1500;TSS	S_Shore	
NR_002942	Body	Island	
NR_003313;NR_003312;NR_(	TSS1500;TSS1500;TSS1500		
NR_029409;NM_019009	Body;TSS1500	S_Shore	
NR_001457;NM_001012	TSS200;Body	S_Shelf	
NM_002041;NM_005254;NM	TSS1500;TSS1500;TSS1500;TS	S_Shore	
NR_027005	Body	N_Shore	
NR_024433	Body	N_Shore	
NR_027822	TSS1500	N_Shore	
NM_001135824;NR_024211;	3'UTR;Body;Body;3'UTR;Body;3'UTR;Body		
NR_003111	TSS1500		
NR_002187;NM_012470;NM_	TSS200;1stExon;5'UTR	Island	

NM_014463;NR_027299;NM_	1stExon;TSS200;TSS200;TSS20	Island	
NM_007298;NM_007300;NR_	Body;Body;Body;5'UTR;Body;Body		
NR_026893;NM_002702	Body;TSS200		
NR_023918;NR_023919	Body;TSS200	N_Shore	
NM_012470;NR_002187	TSS200;Body	Island	
NR_023392;NR_026974	TSS1500;Body	S_Shore	
NR_003675	TSS200	Island	
NR_027771	Body	Island	
NR_028582;NM_138387;NR_	TSS200;TSS200;TSS200	Island	
NR_003660	TSS200	Island	
NM_019015;NR_030407	Body;TSS1500	S_Shore	
NR_024547;NM_003876;NM_	Body;1stExon;5'UTR	Island	
NR_002785;NM_016592	Body;TSS1500	N_Shore	
NR_029422;NM_032311;NM_	TSS200;TSS200;TSS200	Island	
NR_027069	TSS1500	Island	
NM_018917;NM_032092;NM	Body;Body;Body;Body;Body;T	N_Shore	
NR_003034	TSS200	Island	
NR_003261	TSS1500		
NM_001145638;NM_015597	Body;Body;TSS1500		
NM_005161;NR_027991	TSS1500;TSS1500		
NR_003500	Body	Island	
NR_003109;NM_017846	Body;Body	Island	
NM_001170797;NR_033195;	Body;Body;Body;Body;Body;E	Island	
NM_021970;NM_021970;NR_	1stExon;5'UTR;Body		
NR_026790	TSS1500	N_Shore	
NR_027271;NR_023313;NR_(	TSS1500;Body;Body;Body	S_Shore	
NM_001145640;NM_015174	Body;Body;Body	Island	
NR_029690;NM_199070;NM_	TSS1500;5'UTR;5'UTR;5'UTR;5	Island	
NR_001435	Body		
NM_000978;NR_002576	TSS1500;TSS1500	S_Shore	DMR
NR_003655	Body	N_Shore	
NR_031571	TSS1500		
NR_033258;NM_014795;NM_	Body;5'UTR;5'UTR	S_Shore	DMR
NR_015447	TSS200	Island	
NR_024448	TSS1500	S_Shore	
NR_028394;NM_001164556;	Body;TSS1500;TSS1500;TSS15	N_Shore	
NR_015448	TSS200	N_Shelf	
NR_030637;NR_030639;NR_(	TSS1500;TSS1500;TSS1500;TS	N_Shore	
NR_024181;NM_001135590;	TSS1500;TSS1500;TSS1500	S_Shore	
NM_001042594;NM_015450	TSS1500;TSS1500;TSS1500;TS	S_Shore	
NR_024520;NR_024519;NM_	TSS200;TSS200;TSS200;TSS20	Island	
NM_022818;NR_024568;NM_	1stExon;TSS200;5'UTR	Island	
NR_030198;NR_030197	TSS1500;TSS200		
NR_026691;NM_001558	Body;Body	S_Shelf	

NR_002963;NR_003584	TSS1500;TSS1500	Island	
NM_032119;NR_003149	TSS200;TSS200	Island	DMR
NR_026932;NM_014456;NM_	TSS1500;TSS1500;TSS1500	N_Shore	
NM_006098;NR_002591	TSS1500;TSS1500	N_Shore	
NR_026791	Body	Island	
NR_028505;NR_028503;NM_	Body;Body;TSS1500;Body;TSS	Island	
NR_024117	TSS1500	N_Shore	
NR_028138	TSS1500	Island	
NR_015436	Body	S_Shore	
NR_027408	Body		
NM_006098;NR_002591	TSS1500;TSS1500	N_Shore	
NM_015509;NR_024260	1stExon;Body	Island	
NR_029670	TSS1500	Island	
NR_028044;NR_003512;NM_	Body;Body;5'UTR;TSS1500;Bc	S_Shore	
NR_024516;NR_024515;NM_	Body;Body;Body;Body;Body;E	Island	
NR_028595	TSS200	S_Shelf	
NR_015346;NM_005090;NM_	Body;Body;TSS1500		
NR_030318;NM_024963	TSS1500;Body	S_Shore	
NR_030187;NR_030184	TSS1500;TSS1500		
NR_028390	Body		
NM_012399;NR_026962;NR_	TSS1500;Body;Body	Island	
NR_024569;NR_033182	Body;Body		
NM_030803;NR_003008;NM_	Body;TSS1500;Body		
NR_024454;NM_004546	TSS1500;Body	Island	
NR_015436	Body	Island	
NR_024330	Body	Island	RDMR
NR_027257	TSS1500	N_Shore	
NR_026776;NR_026775	TSS200;Body	S_Shore	
NR_003695;NR_003605;NR_(	TSS1500;Body;TSS200;TSS15(	S_Shore	
NM_001145268;NM_001145	TSS200;TSS200;TSS200	N_Shore	
NM_001160210;NR_027662;	TSS1500;TSS1500;TSS1500	S_Shore	
NM_006098;NR_002591	TSS1500;TSS1500	N_Shore	
NM_194457;NM_194315;NR_	Body;Body;Body;Body;Body	S_Shelf	
NR_015381	Body	N_Shelf	
NR_002563;NR_002564;NR_(	TSS1500;TSS200;TSS1500;TSS	N_Shore	
NM_001042424;NM_133330	Body;Body;Body;TSS1500;Body		
NM_005916;NM_182776;NR_	Body;Body;TSS200		
NR_027267;NR_027266	TSS1500;TSS1500		
NR_027284;NR_026861;NR_(	Body;TSS200;TSS200	Island	
NR_023938	Body	Island	
NR_029608	TSS1500	N_Shore	RDMR
NR_024431;NR_024429;NR_(	Body;Body;Body		
NR_026901	Body	Island	
NM_001141970;NR_024517;	TSS1500;TSS1500;TSS1500;TS	S_Shore	

NR_027942;NM_001162861;	Body;Body;Body;Body;TSS150	N_Shore	
NR_024052;NR_024053	Body;Body	N_Shelf	
NR_026887;NM_004364	Body;TSS1500	Island	
NM_001142464;NR_024551;	5'UTR;TSS1500;1stExon;1stEx	S_Shore	
NR_028040;NM_178831;NR_	Body;3'UTR;Body;Body;Body		
NR_029497	TSS200	S_Shelf	
NR_024362;NR_024363;NR_(	TSS1500;TSS1500;TSS1500	Island	
NR_029507;NM_004599	TSS1500;Body		
NR_031607;NM_003726;NM_	Body;Body;Body		
NR_003263	Body	S_Shore	
NM_001040649;NM_001159	1stExon;TSS1500;1stExon;1st	Island	
NR_004382;NM_002402;NM_	Body;TSS1500;5'UTR;TSS150(	Island	
NR_028338;NM_001164281;	Body;Body;Body;Body;Body		
NR_027712	Body	S_Shore	
NR_002795;NM_005523	Body;TSS1500	N_Shore	RDMR
NR_015448	Body		
NR_027107	Body	Island	DMR
NR_021490;NR_021491;NR_(	Body;Body;TSS200	Island	
NM_017739;NR_024332	5'UTR;Body		
NR_030192	TSS1500		
NR_003365	TSS1500	Island	
NM_001167830;NM_206886	1stExon;1stExon;Body;1stExo	Island	
NR_024360	Body	N_Shelf	
NR_024465;NM_006110	TSS1500;TSS200	S_Shore	
NR_027771	Body	Island	
NR_026999	Body	N_Shore	
NR_027503	TSS1500		
NM_000985;NR_002571;NR_	TSS200;TSS1500;TSS1500;TSS	S_Shore	
NR_028032	Body	Island	
NR_024186;NM_024033;NR_	TSS200;TSS200;TSS200	Island	
NM_153638;NR_029519;NM_	Body;TSS200;Body;Body		
NR_021491;NR_021490;NM_	TSS1500;TSS1500;TSS1500	N_Shore	
NR_026967;NM_020901	TSS200;TSS1500	Island	
NM_014620;NR_003084;NM_	5'UTR;Body;TSS1500;5'UTR	N_Shelf	
NR_003491	Body	Island	DMR
NM_001143682;NR_026554;	TSS1500;TSS1500;TSS1500		
NR_002563;NM_002394;NM_	TSS1500;TSS1500;TSS1500;TS	N_Shore	
NM_100486;NM_016628;NM	1stExon;5'UTR;1stExon;5'UTR	Island	
NR_031695;NM_001018108	TSS200;3'UTR	S_Shore	
NR_002583;NM_000997	TSS1500;Body	N_Shelf	
NR_024240;NR_026751	Body;Body	Island	DMR
NR_027262;NM_000520	TSS1500;Body	N_Shore	
NR_031727	TSS200	Island	
NR_027928;NM_005198;NR_	Body;5'UTR;TSS200;1stExon	Island	

NR_003672;NR_002958;NR_(	Body;TSS1500;Body;Body	Island	
NR_024279	Body	Island	
NR_003367	Body		
NM_032810;NM_032810;NR_	5'UTR;1stExon;TSS1500	Island	
NM_207292;NM_021038;NR_	TSS1500;TSS1500;Body;Body	N_Shore	RDMR
NR_028582;NR_028581;NM_	TSS1500;TSS1500;TSS1500	N_Shore	
NR_026846	Body	N_Shore	
NR_027469;NR_027468	TSS1500;TSS1500	S_Shore	
NR_029375;NM_001099737;	TSS1500;TSS1500;Body	Island	RDMR
NR_026765	TSS1500		
NR_002754;NR_002755	Body;Body	N_Shelf	RDMR
NR_028337	TSS200	N_Shelf	
NR_002773	TSS1500		
NR_002328;NM_003846	TSS1500;TSS200	Island	
NR_003531;NR_003530;NR_(	Body;Body;Body	Island	RDMR
NR_001298	Body		
NM_201435;NR_027918;NM_	TSS200;TSS200;TSS200		
NR_015454;NM_002359	Body;TSS1500	Island	
NR_002447;NR_000017;NM_	TSS200;TSS1500;TSS1500;Bo(	S_Shore	
NR_002791	Body		
NM_001146310;NR_024445;	Body;TSS1500;TSS200	Island	
NR_024362;NR_024363;NR_(	TSS1500;TSS1500;TSS1500	Island	
NM_005291;NM_001161415	5'UTR;5'UTR;Body;Body;Body;5'UTR;Body;Body;5'UTR		
NM_001141969;NR_024517;	5'UTR;Body;Body;5'UTR	Island	
NM_052839;NR_027691;NM_	Body;Body;Body	Island	
NR_001434	Body	Island	
NR_024430	Body		
NM_080425;NM_001077490	TSS1500;TSS1500;3'UTR;TSS1	N_Shore	CDMR

Enhancer	CONT1	CONT2	CONT3	CONT4	CONT5	CONT6
NA	0.125082016	0.114232995	0.111208411	0.102083839	0.109666144	0.086928838
NA	0.035836828	0.035417393	0.027840071	0.028868151	0.023696639	0.034664911
NA	0.044419514	0.034240819	0.031041931	0.033890847	0.039572344	0.025323966
TRUE	0.032006664	0.035334547	0.028902706	0.030043345	0.024653864	0.038566155
NA	0.125222288	0.099194052	0.121468403	0.103858828	0.076028929	0.111851797
NA	0.018450685	0.021358408	0.018632620	0.027865381	0.018285199	0.024753456
TRUE	0.024860348	0.025022921	0.018497334	0.020660368	0.023749787	0.025022720
NA	0.046524754	0.048931620	0.038146925	0.039046148	0.040813364	0.051934290
NA	0.794602410	0.803978279	0.781754977	0.709398503	0.748022248	0.801710365
NA	0.023533360	0.017423157	0.022432678	0.026455307	0.018498812	0.022076518
NA	0.022219273	0.024144025	0.017635097	0.020311778	0.020342278	0.029955970
NA	0.018547127	0.017020282	0.014060141	0.019213305	0.016942574	0.020009153
NA	0.031305341	0.030383024	0.028652293	0.027584617	0.030097698	0.035808273
NA	0.794134725	0.867958485	0.825309743	0.784882011	0.787525564	0.809997481
NA	0.033750226	0.039753031	0.034377757	0.035052035	0.028972409	0.039070148
NA	0.019906842	0.018016161	0.019173501	0.016108906	0.020964877	0.023697125
TRUE	0.186544725	0.175317776	0.193350745	0.187804935	0.150983462	0.201623247
NA	0.020667003	0.021759174	0.021736705	0.020898913	0.018820734	0.032597118
NA	0.131184907	0.115660867	0.115858042	0.097357489	0.141144288	0.105307138
NA	0.085282578	0.128356274	0.118971998	0.091386285	0.081032238	0.166549395
NA	0.025075433	0.029102061	0.030820613	0.037592235	0.033886311	0.036752779
NA	0.577642398	0.560548369	0.621170992	0.499607571	0.538106479	0.593587141
NA	0.720065216	0.817168644	0.761792721	0.769034877	0.793982930	0.768254870
NA	0.033528384	0.047789021	0.029006925	0.030650968	0.025741081	0.030404157
NA	0.037561600	0.031631886	0.026704781	0.031920163	0.024021189	0.041076224
TRUE	0.026505672	0.029741982	0.031789686	0.026581813	0.023977375	0.035039806
NA	0.038626447	0.034785157	0.032358398	0.032376148	0.033473471	0.046678667
NA	0.561846601	0.593548862	0.493697646	0.559935997	0.555925883	0.506041957
NA	0.663545311	0.478441324	0.606249451	0.543578499	0.601241509	0.468164727
NA	0.031155257	0.027318504	0.024021304	0.025154091	0.025825168	0.036303320
NA	0.027817603	0.025261916	0.020574125	0.023264378	0.026258670	0.026461600
NA	0.896156964	0.903889519	0.924479998	0.911085714	0.874072240	0.880843566
NA	0.032395235	0.043921766	0.029813467	0.037140777	0.032255344	0.043877480
NA	0.473616232	0.506287206	0.478296598	0.397280695	0.418042631	0.373145776
NA	0.025465891	0.024164463	0.015921642	0.016620364	0.022681740	0.023353082
NA	0.201706937	0.201241582	0.194393394	0.182680347	0.257661824	0.191925676
NA	0.033772402	0.038173357	0.028192666	0.025979129	0.035501478	0.034435821
NA	0.323477751	0.372399729	0.331669790	0.329039117	0.392834567	0.411048959
NA	0.016763736	0.018162636	0.012955149	0.020493173	0.016809627	0.018773210
NA	0.034309801	0.027712399	0.025456862	0.039478739	0.031913659	0.034343755
NA	0.666930688	0.722820289	0.621051791	0.680242343	0.731636524	0.665602520
TRUE	0.478684606	0.432427469	0.406867365	0.344268175	0.464270008	0.370072904
NA	0.077220135	0.051448582	0.060945949	0.043366693	0.083299828	0.087879725

NA	0.064020382	0.113312330	0.107320731	0.061588771	0.102159065	0.141638695
NA	0.018846786	0.016892635	0.018730850	0.017291165	0.015385894	0.017575891
NA	0.059505312	0.042864534	0.048072417	0.047291147	0.053047279	0.047122569
NA	0.261937490	0.255035518	0.217160518	0.208784082	0.262843022	0.221311746
NA	0.049973695	0.048757797	0.039351890	0.045653160	0.041364727	0.059206050
NA	0.226796481	0.211370464	0.194033998	0.135604740	0.114889997	0.190695967
NA	0.094241140	0.076642475	0.071012249	0.070776906	0.098707279	0.084865213
NA	0.050751758	0.042574949	0.038354156	0.059852144	0.052553124	0.043779196
NA	0.068773384	0.062169880	0.058395704	0.066832338	0.043929448	0.083291261
NA	0.087289433	0.061128922	0.097603359	0.094325011	0.126722170	0.110362529
NA	0.033970226	0.041735678	0.030995412	0.038442389	0.033484690	0.038451419
NA	0.034037338	0.021370634	0.016574953	0.022205854	0.028261622	0.030320082
NA	0.038234191	0.046946973	0.043594512	0.038128528	0.036707855	0.057230271
NA	0.027077475	0.021791266	0.026374878	0.020634554	0.019496234	0.032028103
NA	0.369116523	0.331391459	0.354763837	0.303835177	0.296090872	0.322057792
NA	0.023642666	0.022896722	0.019155225	0.024170060	0.024017190	0.027037363
NA	0.062369379	0.062541805	0.050917361	0.040329196	0.060438172	0.049684967
NA	0.027093161	0.028088674	0.025031291	0.026711560	0.026891631	0.039754960
NA	0.039851622	0.038286796	0.031896941	0.029424940	0.032383500	0.036342912
NA	0.045640772	0.032951725	0.037478405	0.038975904	0.029984771	0.056395639
NA	0.033316525	0.033527092	0.030535135	0.036499864	0.024421664	0.023635108
NA	0.016506451	0.020516913	0.014707842	0.014593276	0.012012635	0.017410462
NA	0.037630432	0.028882584	0.027479393	0.031318372	0.036782398	0.028366682
NA	0.042780659	0.037762897	0.037420006	0.032875230	0.025725865	0.045834070
NA	0.027780199	0.033896414	0.033767277	0.024480376	0.027119270	0.031237941
NA	0.026747601	0.023671953	0.026630567	0.027703876	0.029137744	0.039325831
NA	0.032660812	0.034756416	0.033940238	0.028468907	0.025389655	0.056018957
NA	0.032221373	0.039065734	0.040950498	0.030347806	0.025941322	0.031711414
NA	0.031215849	0.042298070	0.029074997	0.036086251	0.038408253	0.043290914
NA	0.493680784	0.482140407	0.465340563	0.393658823	0.469250465	0.503719292
NA	0.305875458	0.224556572	0.264530570	0.268066416	0.364757985	0.236749375
NA	0.803604744	0.763789417	0.745039521	0.718131989	0.749662288	0.681447192
NA	0.032339145	0.040433308	0.021302738	0.034386600	0.033681500	0.037795468
NA	0.595791070	0.644710551	0.607579746	0.551332425	0.537817038	0.625724665
NA	0.179844591	0.167283002	0.171349354	0.128681265	0.189704916	0.156530570
NA	0.014326863	0.018485287	0.014518523	0.015455271	0.013004644	0.021016728
NA	0.189236583	0.182373301	0.142640314	0.158880289	0.214609005	0.196596860
NA	0.058374631	0.053277772	0.045225515	0.045152283	0.045465888	0.054445234
NA	0.032685698	0.032516764	0.027061222	0.030307821	0.034048815	0.040405075
NA	0.214792429	0.266318666	0.291009653	0.247105806	0.309681320	0.223431110
NA	0.357488687	0.431448678	0.383632789	0.357199595	0.348567843	0.418169895
NA	0.044412696	0.037952629	0.034994964	0.034333763	0.039848977	0.050529385
NA	0.119206580	0.109418745	0.113986719	0.110105558	0.121834261	0.129201151
NA	0.018616730	0.016818067	0.014471830	0.015491048	0.015969305	0.021985290

NA		0.036895129	0.039679461	0.028835831	0.035734328	0.027665348	0.046381559
NA		0.023396191	0.025595529	0.022896699	0.025785009	0.017855057	0.031952326
NA		0.017238816	0.014979743	0.015236044	0.015997387	0.017385830	0.018750643
	TRUE	0.509419790	0.478328939	0.488551902	0.416748311	0.399621339	0.373671120
NA		0.031361934	0.042164947	0.036636934	0.044386638	0.041523585	0.053164916
	TRUE	0.023412248	0.015591973	0.019910379	0.026775816	0.022142209	0.020289974
NA		0.026094147	0.037872135	0.024122192	0.022161353	0.026144542	0.033337361
NA		0.063292108	0.070150670	0.053348051	0.066949603	0.066184595	0.065733021
NA		0.842673074	0.872657134	0.789295574	0.795260814	0.770623966	0.801176337
NA		0.055937681	0.061852281	0.051891113	0.059163119	0.062402514	0.076815923
NA		0.171407751	0.158270208	0.138200546	0.137954857	0.114193769	0.176377508
NA		0.034125094	0.041234099	0.028848051	0.032845677	0.033249828	0.031219000
NA		0.021580546	0.021190518	0.022967509	0.018994527	0.021179762	0.028554906
NA		0.037746194	0.030981758	0.021996425	0.024224782	0.023157617	0.029391807
NA		0.103560754	0.067677994	0.078600220	0.095916827	0.070909453	0.071946887
NA		0.029033368	0.017459763	0.021932392	0.020541277	0.021499312	0.023431485
NA		0.385362929	0.404682581	0.376615983	0.410091170	0.406083166	0.375672441
NA		0.015068528	0.013819826	0.013903785	0.013174560	0.018329639	0.015588960
NA		0.025088239	0.026132778	0.023794539	0.025962203	0.021010854	0.042751669
NA		0.044879592	0.046145224	0.041395606	0.044457896	0.046245515	0.058884337
NA		0.034849819	0.034298405	0.047841988	0.032570960	0.031945072	0.067216868
NA		0.019140526	0.028748491	0.022933826	0.021238141	0.017224424	0.022493516
NA		0.026869772	0.025925727	0.020305578	0.030516559	0.018195786	0.028866812
NA		0.040416137	0.036844197	0.037412738	0.032965090	0.028055689	0.048336325
NA		0.026671684	0.023615458	0.021080155	0.013419342	0.019961361	0.025080785
NA		0.097785833	0.072350490	0.056517258	0.102594308	0.090219857	0.080909495
NA		0.046828952	0.052015069	0.028666251	0.065245336	0.074787795	0.056558911
NA		0.401086714	0.465435345	0.386386251	0.430065106	0.504251985	0.439277813
NA		0.089385404	0.088924270	0.067834009	0.084712757	0.104919353	0.095087333
NA		0.028256342	0.024011968	0.021902183	0.017270300	0.027894399	0.030158899
NA		0.063958849	0.042668034	0.060805317	0.045281916	0.036138348	0.053507842
NA		0.016673410	0.021550791	0.016287988	0.014841390	0.018568946	0.020450056
	TRUE	0.400673132	0.443843384	0.384516553	0.375261227	0.405129130	0.346448077
NA		0.022982151	0.017887288	0.019930898	0.022626989	0.024507128	0.022679865
NA		0.157574968	0.242704381	0.188550055	0.165696695	0.190186599	0.196584410
NA		0.029786126	0.021477580	0.025487568	0.024750865	0.026170617	0.028693265
NA		0.053294347	0.046631059	0.046106822	0.040398416	0.056205762	0.065381120
NA		0.027445462	0.034410782	0.023829554	0.023970624	0.025561083	0.031552169
NA		0.093562963	0.089700285	0.105247857	0.057460504	0.064575047	0.158120225
NA		0.063058291	0.049078830	0.045908421	0.053495220	0.067518378	0.053297406
NA		0.022242093	0.018450715	0.018933249	0.017334064	0.019166076	0.019006125
NA		0.043189409	0.044074267	0.043358320	0.033522096	0.030886189	0.042260647
NA		0.030238443	0.045542865	0.031034257	0.031974156	0.038002983	0.028879298
NA		0.638083357	0.647212671	0.595236726	0.578060111	0.601351552	0.558540560

NA		0.093179685	0.095265976	0.083083256	0.094597152	0.102429347	0.096403359
NA		0.037798551	0.037646691	0.042662630	0.044864373	0.041779017	0.054889593
NA		0.026689630	0.022177683	0.024157108	0.018296996	0.023104302	0.028817873
NA		0.027787452	0.030662213	0.025677973	0.021345460	0.023903847	0.039478028
NA		0.039728258	0.042284131	0.036683812	0.053418351	0.031715204	0.063905558
NA		0.039460736	0.035645104	0.040234056	0.054315019	0.040182138	0.060250361
NA		0.026055849	0.025820753	0.039735742	0.025099255	0.019850583	0.036335284
NA		0.045192164	0.047677372	0.069900889	0.052712461	0.040455922	0.082829233
NA		0.028214206	0.023818542	0.023292360	0.029811053	0.023189594	0.034224712
NA		0.048448338	0.034939511	0.042808169	0.043322745	0.039178010	0.045682754
	TRUE	0.043339326	0.035362908	0.030142962	0.031224746	0.034634598	0.037878573
	TRUE	0.031200952	0.037508725	0.034295799	0.027812165	0.028168767	0.055160631
NA		0.030832196	0.032090595	0.032167097	0.028750621	0.030678884	0.045280985
NA		0.028583565	0.033012624	0.025487035	0.024896996	0.032365474	0.024437091
NA		0.031866185	0.040573607	0.040120706	0.026819261	0.028283587	0.038750851
NA		0.017537858	0.023093953	0.018749948	0.018384270	0.017689297	0.024965157
NA		0.031361806	0.031578938	0.031917832	0.022371222	0.026565010	0.029317143
NA		0.034624401	0.038453676	0.054494511	0.041681903	0.037604691	0.055270424
NA		0.025675326	0.030567636	0.030681384	0.023899305	0.034222407	0.045746549
NA		0.025295129	0.021710760	0.023522354	0.019919439	0.024878860	0.027526565
NA		0.106265610	0.113577671	0.088734572	0.077072751	0.085875036	0.075320302
NA		0.551293945	0.653411026	0.587409203	0.522325469	0.502423134	0.516713343
NA		0.064505403	0.060538896	0.051394187	0.054986973	0.045676302	0.044587429
NA		0.189570037	0.190066618	0.160113597	0.190044905	0.257361196	0.196241357
NA		0.066423241	0.052984052	0.057806118	0.054357499	0.054566375	0.078917508
NA		0.021297706	0.029540478	0.028866416	0.025242648	0.018644274	0.024754684
NA		0.037263005	0.029806156	0.024822093	0.024111424	0.033414638	0.030949752
NA		0.229319017	0.256302642	0.206360833	0.192929143	0.177623946	0.181564214
NA		0.047805600	0.037757102	0.033351001	0.035666460	0.035138948	0.041291636
NA		0.042407029	0.041988991	0.040380100	0.032202805	0.023451930	0.034010493
NA		0.667326967	0.695108422	0.692977470	0.667980825	0.730920900	0.715540708
NA		0.026599104	0.026403930	0.029076953	0.022137217	0.024585293	0.039020841
NA		0.666779291	0.680717114	0.662359404	0.624177523	0.660238659	0.623401756
NA		0.035871884	0.036380057	0.039133284	0.038144417	0.033359697	0.037542911
NA		0.046219166	0.058300607	0.047808717	0.042481604	0.043127043	0.065549353
NA		0.038927829	0.032592036	0.037721218	0.046564609	0.039781999	0.041738564
NA		0.135495330	0.159241150	0.127032131	0.113772846	0.105265392	0.107544330
NA		0.816808093	0.804047441	0.788104761	0.770365038	0.793704716	0.770058858
NA		0.021532383	0.020638877	0.018566978	0.019642942	0.020182027	0.023779489
NA		0.025046407	0.023171965	0.016123732	0.018366798	0.019082459	0.023673274
NA		0.693784680	0.647001563	0.629210515	0.506130041	0.552038577	0.493172478
NA		0.046436228	0.030480493	0.044291554	0.031749270	0.042695439	0.052074486
NA		0.365777390	0.395306390	0.325086634	0.340831105	0.324882967	0.356512842
NA		0.030231906	0.034002206	0.028389134	0.032351852	0.027104976	0.038322080

NA	0.033899614	0.030576481	0.030760095	0.024416908	0.026861791	0.040826794
NA	0.397894754	0.416935301	0.354901396	0.360633029	0.420706747	0.435437609
NA	0.038004597	0.036642899	0.037284612	0.037112181	0.032169375	0.045521624
NA	0.079897174	0.096553099	0.079230889	0.060875285	0.050801524	0.105732555
NA	0.025777480	0.022201848	0.017858005	0.017567097	0.015272562	0.022648331
NA	0.045624274	0.046637631	0.035531042	0.040097720	0.035292845	0.064852234
NA	0.021771197	0.034860976	0.028198807	0.024197983	0.020578176	0.025695309
NA	0.023173106	0.022838596	0.019345077	0.021968207	0.016860826	0.030577350
NA	0.026284036	0.031029779	0.028742665	0.025363065	0.022257590	0.043482208
NA	0.020093736	0.022146739	0.025576822	0.028908285	0.016828302	0.025402843
NA	0.037937794	0.033364490	0.028758577	0.041986627	0.029035104	0.035377274
NA	0.879590088	0.884792970	0.856561811	0.841191859	0.885830896	0.844254774
NA	0.041471161	0.042335334	0.043430593	0.024060075	0.024650082	0.031179468
NA	0.037298690	0.038161823	0.030108804	0.031154266	0.031660860	0.037108745
NA	0.028815128	0.021900097	0.023193718	0.022873944	0.022276185	0.031961663
NA	0.193199997	0.224097235	0.178700188	0.188376656	0.182863560	0.227176259
NA	0.032727544	0.035198414	0.030154661	0.032931340	0.028232555	0.045775377
NA	0.054196788	0.067607162	0.047562652	0.048054772	0.033892220	0.044001757
NA	0.031408181	0.035829952	0.029824339	0.024849682	0.025737833	0.022206303
NA	0.035874969	0.038251900	0.026222557	0.022989183	0.030249728	0.041303337
NA	0.061347150	0.062602058	0.053634512	0.059185599	0.054528995	0.074019167
NA	0.047013113	0.063467954	0.044882439	0.052741476	0.053587311	0.074511412
NA	0.380576473	0.389244844	0.311927389	0.301518815	0.336918846	0.290598403
NA	0.060531128	0.064524560	0.055073770	0.057163769	0.058048365	0.076257475
NA	0.148419124	0.177505754	0.189574869	0.209314886	0.150861970	0.174878834
NA	0.030462196	0.027915642	0.022465377	0.024805732	0.018652602	0.034050546
NA	0.023024216	0.029313263	0.029925139	0.021097640	0.029536889	0.028093019
NA	0.091015351	0.088690025	0.100453581	0.056473897	0.094179718	0.067804927
NA	0.022659559	0.020677831	0.027432003	0.029507219	0.023303781	0.029395388
TRUE	0.030328024	0.026348078	0.026471244	0.027844159	0.026762530	0.031910242
NA	0.016255215	0.013652247	0.011953992	0.012927212	0.015256391	0.017623631
NA	0.039965341	0.030363567	0.030330166	0.024390670	0.034446967	0.027369782
NA	0.029269914	0.030120909	0.028728070	0.027451099	0.027178312	0.029445597
NA	0.034018917	0.019726355	0.015902364	0.019565475	0.019006661	0.028542755
NA	0.455772550	0.448994658	0.414699413	0.475854192	0.513157114	0.378425231
NA	0.032180744	0.028432570	0.030148775	0.027975746	0.025851498	0.037141451
NA	0.027117704	0.025236573	0.022394045	0.026438159	0.021470258	0.036227618
NA	0.023716456	0.025415676	0.023127922	0.020116049	0.021988720	0.039176878
NA	0.330323983	0.295271582	0.309298670	0.396307172	0.389354596	0.309018874
NA	0.027782242	0.016928192	0.017593083	0.021229843	0.024566521	0.025797622
NA	0.027513338	0.018275248	0.019085640	0.021327418	0.020050709	0.023801563
NA	0.139030273	0.157068008	0.142218367	0.093613911	0.117002428	0.149209624
NA	0.037806060	0.037544227	0.037185150	0.033025994	0.030845058	0.041602364
NA	0.050316504	0.051317441	0.047893452	0.046122549	0.043962294	0.074056682

NA		0.021040350	0.023238602	0.016375254	0.018166256	0.015306715	0.019848841
NA		0.018929655	0.017017517	0.014453642	0.015639908	0.015771488	0.028112054
NA		0.146505678	0.140154759	0.105856758	0.092551198	0.083817582	0.094954844
NA		0.039709955	0.032033516	0.023915408	0.031145710	0.028576213	0.034877084
NA		0.262162569	0.344000241	0.227333207	0.268932857	0.248636587	0.303801207
NA		0.094795660	0.087467087	0.081711855	0.101540821	0.108205234	0.074956563
NA		0.052675506	0.064028281	0.046304891	0.057432558	0.045917714	0.077900497
NA		0.081286186	0.088340378	0.082301517	0.111886671	0.107593280	0.099581605
	TRUE	0.246276827	0.283354931	0.203257575	0.235682141	0.240753751	0.258597701
NA		0.086194516	0.083074217	0.050858286	0.038246410	0.028839687	0.060438133
NA		0.167160914	0.201724093	0.150945658	0.157404552	0.122809505	0.170123806
NA		0.024011333	0.019823322	0.031058217	0.022389170	0.020411232	0.045087717
NA		0.226202694	0.315890772	0.237768600	0.224574594	0.241399902	0.249794244
NA		0.031914438	0.020228994	0.024520758	0.033324055	0.022365986	0.035276761
NA		0.059641276	0.054838836	0.053271996	0.056661238	0.050671527	0.065433971
NA		0.588958725	0.673830032	0.615804605	0.581830164	0.583888689	0.584348549
NA		0.017601153	0.021612175	0.017229397	0.015139583	0.018643715	0.021813112
NA		0.038117400	0.036107701	0.033799167	0.029252123	0.036627587	0.034996900
NA		0.017777390	0.019823577	0.014612273	0.015350735	0.016607144	0.019548987
NA		0.768048159	0.516763673	0.781755502	0.748576725	0.732319125	0.601830636
NA		0.069484688	0.075111769	0.053364194	0.063152737	0.059117322	0.066732425
NA		0.038174998	0.034605591	0.037787268	0.033735802	0.040503077	0.032733823
NA		0.039003584	0.041793643	0.041216706	0.037961441	0.037299782	0.038277236
NA		0.061190858	0.063473997	0.048194818	0.042100646	0.054472056	0.061661800
NA		0.362635902	0.353460369	0.302631673	0.298330853	0.390092796	0.323036933
NA		0.041330186	0.043610271	0.030838992	0.040672452	0.028923743	0.045650698
NA		0.033711829	0.047676061	0.036150015	0.040502468	0.037177189	0.047838252
NA		0.797515478	0.428855900	0.771861324	0.670486135	0.712812043	0.379877583
NA		0.846916689	0.865131970	0.816960827	0.765260532	0.801214360	0.848044177
	TRUE	0.024054503	0.023874947	0.034887776	0.027881358	0.027346138	0.028194227
NA		0.073788546	0.044116310	0.060332928	0.081814433	0.110684315	0.061342749
NA		0.123592094	0.112235610	0.123287714	0.161717152	0.103603119	0.131841696
NA		0.393396520	0.415855097	0.361197492	0.367036603	0.357847315	0.379076812
NA		0.052206004	0.056752638	0.038496513	0.059930608	0.036130565	0.049941363
NA		0.709556242	0.730289240	0.631868212	0.652971155	0.757527948	0.753212321
	TRUE	0.026430116	0.035775260	0.027717728	0.024425596	0.026474249	0.039026081
NA		0.055901340	0.052843733	0.054475343	0.054933314	0.053351940	0.055899298
NA		0.061398248	0.064708508	0.053099446	0.049491852	0.059106030	0.063328404
NA		0.679864211	0.720727591	0.656116392	0.665915883	0.645775350	0.562981426
NA		0.060809677	0.052911305	0.080330002	0.059929520	0.068977549	0.066579493
	TRUE	0.032303597	0.046965978	0.034681135	0.033200078	0.027755128	0.039424452
NA		0.046291912	0.051901053	0.040951667	0.066803970	0.056213547	0.056222834
NA		0.031181261	0.020423086	0.022903695	0.019704313	0.024577610	0.025641321
	TRUE	0.707979130	0.739964505	0.712829890	0.673310380	0.736914480	0.640411323

NA		0.283213151	0.187914179	0.347678533	0.146025358	0.128724278	0.201319070
NA		0.042583141	0.061694349	0.069041024	0.058753093	0.060224154	0.057868245
NA		0.127081378	0.146271556	0.118807535	0.103566413	0.104949507	0.123878821
NA		0.054824055	0.041437265	0.042987463	0.045886776	0.037097450	0.047387106
NA		0.337832473	0.423247068	0.370383435	0.263324512	0.374736263	0.273106737
NA		0.556584331	0.583602155	0.564880548	0.540493873	0.578224702	0.579128913
NA		0.048788474	0.054906607	0.048899626	0.043662069	0.033512900	0.076852229
	TRUE	0.757838717	0.731057063	0.765757220	0.748180995	0.822594584	0.753971338
NA		0.033755287	0.040485406	0.032263540	0.028542350	0.031989990	0.044641141
NA		0.119023094	0.140131672	0.107864821	0.119754493	0.123997139	0.132264846
NA		0.026553549	0.038768445	0.031327831	0.025847622	0.021015215	0.038789818
NA		0.640438309	0.608914399	0.599052077	0.466600775	0.570652869	0.430950058
NA		0.036714729	0.029554154	0.036885177	0.029040620	0.035105120	0.045233099
NA		0.025777946	0.029357272	0.026008613	0.022824896	0.020001978	0.030150711
NA		0.015439369	0.014009081	0.017268491	0.017400038	0.018144544	0.022403792
NA		0.763241562	0.804200037	0.776685986	0.754931399	0.813133682	0.693345357
NA		0.014666600	0.011959736	0.010884427	0.010945501	0.010112724	0.015262892
NA		0.291949418	0.280508932	0.263286271	0.254957258	0.236099078	0.302340845
	TRUE	0.738873058	0.796005660	0.682992781	0.688695389	0.768264255	0.730058658
	TRUE	0.042162584	0.029764190	0.036855682	0.046246126	0.023982146	0.038655981
NA		0.210977436	0.173969040	0.192697561	0.162437730	0.312352360	0.176670579
NA		0.030742799	0.024968259	0.026240221	0.028914150	0.021874029	0.035937656
NA		0.019533271	0.014193625	0.013966130	0.013179219	0.014514959	0.019136160
NA		0.072722914	0.061925409	0.058085127	0.045339488	0.069611139	0.068162523
NA		0.051404236	0.042279731	0.044322943	0.051884114	0.038507779	0.049385879
NA		0.047582682	0.082988388	0.085445800	0.048840883	0.082468512	0.080977322
NA		0.833277476	0.836849116	0.780345639	0.784456128	0.874656867	0.790188647
NA		0.018049824	0.018619028	0.019019441	0.015315675	0.017145653	0.022950972
	TRUE	0.265437226	0.197038581	0.411314600	0.356621571	0.370022727	0.247539922
NA		0.054124061	0.061787263	0.045292362	0.046616551	0.057363446	0.058679869
NA		0.080047799	0.083905551	0.071151009	0.078138113	0.061658678	0.084061122
NA		0.052745986	0.058701492	0.059590883	0.045391662	0.059836847	0.061611034
NA		0.026323750	0.032270621	0.022438747	0.026514592	0.020447404	0.036514480
NA		0.045465264	0.046785412	0.036373089	0.042344832	0.038273382	0.049843862
NA		0.043810781	0.046047949	0.036945248	0.043692072	0.041604433	0.048485276
NA		0.642649910	0.794153461	0.676764411	0.683290699	0.643231732	0.728464221
NA		0.032417164	0.033406579	0.031685432	0.031073111	0.037975000	0.045469504
NA		0.195530767	0.157975524	0.174792724	0.106539577	0.104743403	0.126565244
NA		0.039807322	0.038681883	0.055724512	0.042874306	0.044215509	0.066356783
NA		0.049055785	0.045233675	0.044852416	0.048706692	0.055069775	0.040046034
NA		0.025118778	0.011761268	0.021123898	0.023622760	0.022387394	0.023782257
NA		0.342244686	0.373120160	0.279099764	0.283448291	0.331527162	0.300091595
	TRUE	0.029220920	0.028065824	0.024520861	0.026547429	0.028824987	0.030225200
NA		0.027163986	0.031070525	0.020222374	0.020451330	0.022637479	0.030799736

NA		0.025092437	0.030328329	0.019214234	0.021113058	0.021281043	0.029465126
	TRUE	0.120189090	0.145833604	0.111342368	0.110951946	0.104095937	0.111676629
NA		0.497735421	0.515140873	0.510079027	0.463730553	0.492161267	0.508843448
NA		0.016404973	0.018811708	0.014818849	0.015007258	0.016338901	0.018748111
	TRUE	0.460057255	0.507110114	0.396096322	0.413885695	0.453587268	0.451935797
NA		0.031880057	0.023336025	0.022490028	0.024678889	0.025577882	0.037017830
NA		0.066756397	0.058258928	0.048336315	0.048633299	0.050845055	0.067673771
NA		0.038709316	0.038021185	0.032647574	0.035090126	0.034373034	0.053375819
NA		0.439838994	0.447479596	0.363752383	0.331346349	0.400110237	0.333916147
NA		0.046258637	0.042965681	0.034904398	0.040888503	0.048536072	0.045598643
NA		0.055882849	0.052039144	0.057377083	0.054082814	0.053807429	0.059754833
NA		0.018458398	0.019149649	0.019418331	0.026475600	0.027810226	0.027192725
NA		0.288446824	0.317946555	0.259781521	0.261460987	0.346852793	0.284360905
NA		0.024743628	0.023090174	0.022641753	0.028832093	0.019918102	0.036048291
NA		0.061570487	0.061949305	0.053774202	0.049169092	0.058827295	0.084009323
NA		0.315545459	0.337638594	0.294684315	0.316978140	0.293063046	0.307406468
NA		0.020525571	0.018930980	0.018656388	0.023222690	0.016515434	0.022601130
NA		0.096217527	0.117560831	0.136475424	0.133921486	0.123713938	0.139500325
NA		0.020274101	0.013198154	0.018092035	0.015422081	0.018791969	0.028244858
NA		0.031950264	0.040740199	0.034572336	0.028247227	0.034333061	0.042137817
NA		0.046908054	0.050622748	0.040179725	0.046718152	0.049822994	0.062373346
NA		0.064070426	0.054343363	0.059364621	0.062727323	0.058793871	0.080006183
NA		0.037199144	0.027998164	0.031941520	0.022705286	0.026814042	0.041507936
NA		0.323023196	0.340821944	0.328633541	0.405800024	0.520570950	0.401039262
NA		0.043713208	0.057094936	0.044043307	0.049997713	0.041869010	0.050873099
NA		0.750141447	0.769316327	0.784885763	0.692305810	0.751575619	0.754057067
NA		0.064795051	0.036215053	0.053985378	0.044116606	0.052087011	0.052721688
	TRUE	0.033185800	0.031811641	0.037541008	0.031229681	0.032018391	0.059734362
NA		0.389846394	0.378102448	0.354883991	0.322067676	0.319944276	0.296782676
NA		0.130498803	0.096970775	0.112554865	0.107117525	0.088399671	0.168540886
NA		0.050283561	0.040494602	0.042573906	0.037566763	0.043042657	0.050314880
NA		0.019793374	0.019062973	0.018829514	0.026803577	0.029024868	0.026565610
	TRUE	0.047090486	0.040163661	0.042411288	0.045455739	0.046863764	0.040649271
NA		0.030100103	0.036435231	0.020717938	0.035489967	0.030216956	0.039274983
NA		0.053242924	0.053205956	0.041448200	0.043881457	0.035701271	0.045976611
NA		0.062613352	0.069515222	0.047323600	0.051321994	0.046712668	0.058904168
NA		0.040713176	0.031585941	0.030556037	0.038304188	0.038239116	0.039154667
NA		0.030613124	0.022648196	0.020838082	0.017645539	0.019288758	0.025726288
NA		0.175228456	0.162562096	0.138190117	0.132659598	0.154117952	0.159800316
NA		0.027159358	0.023542942	0.022900824	0.028202752	0.031962047	0.029416317
NA		0.135665166	0.095510968	0.114646659	0.109681456	0.124082690	0.106598788
NA		0.020012434	0.035575812	0.026445522	0.019859154	0.029322519	0.024225788
NA		0.036290902	0.024947035	0.023728551	0.032813701	0.025296345	0.030744577
NA		0.019144480	0.022260689	0.019316880	0.024203923	0.018253191	0.024605306

NA	0.021086681	0.017242831	0.021266851	0.026244761	0.018615587	0.027639393
NA	0.030485167	0.022681706	0.028054777	0.027723982	0.029785128	0.031091072
NA	0.032714117	0.034167829	0.023190861	0.040873148	0.031013448	0.031296579
NA	0.031005161	0.042720075	0.033033834	0.032567612	0.029127683	0.043324777
NA	0.379208432	0.349424894	0.321446881	0.346426758	0.451919277	0.373870371
NA	0.033783911	0.039201048	0.030320623	0.030689547	0.028744929	0.038044426
NA	0.083468986	0.073573330	0.068819409	0.046030140	0.048596075	0.111195981
NA	0.043890049	0.048973775	0.039634563	0.041625811	0.035448600	0.055745604
NA	0.037420522	0.037798613	0.035890310	0.037509398	0.028419304	0.046421035
TRUE	0.737716799	0.755371074	0.690173396	0.709373090	0.708185962	0.739438468
NA	0.486362832	0.490889403	0.430681638	0.520266301	0.524886901	0.397346423
NA	0.024647695	0.028388179	0.017966191	0.024723601	0.020456195	0.033892164
NA	0.043381009	0.035212510	0.040123442	0.043232631	0.035584526	0.039738152
NA	0.295531693	0.294528594	0.258309067	0.270860045	0.275752564	0.259565219
NA	0.048977197	0.047945140	0.037702419	0.046441615	0.053008015	0.056601723
NA	0.207977116	0.195161776	0.134893253	0.175152611	0.196360882	0.204303674
NA	0.023028966	0.026867877	0.025725848	0.029369351	0.028408746	0.037859638
NA	0.032117241	0.020180255	0.021234363	0.023688120	0.026115680	0.035635355
NA	0.077706612	0.098541013	0.056364215	0.067635910	0.063034303	0.085452341
NA	0.020923090	0.019470971	0.016902435	0.024739885	0.022910083	0.025054850
NA	0.270185959	0.314069061	0.214080766	0.246232759	0.249199872	0.217452560
NA	0.055837689	0.063064289	0.047741436	0.050418966	0.052995753	0.069465106
NA	0.031655072	0.039945177	0.022468738	0.027923070	0.028459227	0.051111085
NA	0.024631497	0.017066501	0.021428248	0.021468319	0.021668908	0.025286066
NA	0.071646052	0.086197604	0.074939212	0.092245672	0.095001972	0.095245802
TRUE	0.420863561	0.424084432	0.327893889	0.436478172	0.325736770	0.346523539
NA	0.033450500	0.033548303	0.024361677	0.032931066	0.028890817	0.040464020
NA	0.017807542	0.020814334	0.015206277	0.013990044	0.014786748	0.017661108
NA	0.773203223	0.808425166	0.759249624	0.720330825	0.734673252	0.796721224
NA	0.028603946	0.023648049	0.020941987	0.021245689	0.017492774	0.023781268
NA	0.041152489	0.034740366	0.039501819	0.033002564	0.035793135	0.051779750
NA	0.012887638	0.010389390	0.013353540	0.015318517	0.010916126	0.018152241
NA	0.079742525	0.042707040	0.057377605	0.038050873	0.038654218	0.044639236
NA	0.026169748	0.024722755	0.038562883	0.045800077	0.027747743	0.042080735
NA	0.032214202	0.031686247	0.030755965	0.032334941	0.032799435	0.043398867
NA	0.049514542	0.050982660	0.042425106	0.053194095	0.049191396	0.055951060
NA	0.262479812	0.295611176	0.245943364	0.269359803	0.239141535	0.264360758
NA	0.037572078	0.044822044	0.028829225	0.038712916	0.034112524	0.039791909
NA	0.041480381	0.039024737	0.038938883	0.041461065	0.037962226	0.049770005
NA	0.859453567	0.906258276	0.815498841	0.835643802	0.843137341	0.827528008
NA	0.411153123	0.361363362	0.372452402	0.327159376	0.328595957	0.326293476
NA	0.022390378	0.024653716	0.023667194	0.021152869	0.023431547	0.035451093
NA	0.025143847	0.027170213	0.026721403	0.020508177	0.019355080	0.032092009
NA	0.029360410	0.025826613	0.019537358	0.019350897	0.027861086	0.025659419

NA		0.018730213	0.013456099	0.014302815	0.019449941	0.020480166	0.017495867
NA		0.718761712	0.763539927	0.732147790	0.720100531	0.755122761	0.711748134
NA		0.036083572	0.035446644	0.030542873	0.028172303	0.031548874	0.042568168
NA		0.500277328	0.529434453	0.452722834	0.441909877	0.397124998	0.455298373
NA		0.395538217	0.384455183	0.400495135	0.281895721	0.366805259	0.352002817
NA		0.316824966	0.335811537	0.294533699	0.309572869	0.308164812	0.307984725
NA		0.036435432	0.036970087	0.030564847	0.037335612	0.029669967	0.043262510
NA		0.030929551	0.034947541	0.031627084	0.032823450	0.025500996	0.032310206
NA		0.219848468	0.243183369	0.201397905	0.245380713	0.323897348	0.341376778
NA		0.282097160	0.256600740	0.304349015	0.319892354	0.310559286	0.222638937
NA		0.793646886	0.854205895	0.831231398	0.804493567	0.826989351	0.858473965
NA		0.214175463	0.240226768	0.165196796	0.191528695	0.189735491	0.224452436
NA		0.395448180	0.376241215	0.366176060	0.287303978	0.428170298	0.329685072
NA		0.058752962	0.084422849	0.098747262	0.053129417	0.080059388	0.103429560
NA		0.355776411	0.381804637	0.314672339	0.319219010	0.355204897	0.358038958
NA		0.032654924	0.021805012	0.023132985	0.023752738	0.022531022	0.024069977
NA		0.023525412	0.022001975	0.022928010	0.016714941	0.019346593	0.025931841
NA		0.095091679	0.078743298	0.080017604	0.100688866	0.183589118	0.112115990
NA		0.038021525	0.023488232	0.030228028	0.029243905	0.025524361	0.038528273
NA		0.609277832	0.551846219	0.545232308	0.536055141	0.572487560	0.522476897
NA		0.045911274	0.032870606	0.039991867	0.042968670	0.032845117	0.064185399
	TRUE	0.602243513	0.667561522	0.623346946	0.594019560	0.593809402	0.646832691
NA		0.032893452	0.034456293	0.027579896	0.026638019	0.032189901	0.038251407
NA		0.031596180	0.021738155	0.017903593	0.022849153	0.024821595	0.028108613
NA		0.042567785	0.053265825	0.058901383	0.059476706	0.040495636	0.070880526
NA		0.552953957	0.614897710	0.556210660	0.526018833	0.676478809	0.483533242
NA		0.099862295	0.103754130	0.089034325	0.091617725	0.111705992	0.112743618
NA		0.020330951	0.015700118	0.019223132	0.014040038	0.011333777	0.021787004
NA		0.257257722	0.265936350	0.203468851	0.226988953	0.255473058	0.231895903
	TRUE	0.237253320	0.310310694	0.286027400	0.258554663	0.249451888	0.232811097
NA		0.043305294	0.038818337	0.028859832	0.034507370	0.030134171	0.053154217
NA		0.091883818	0.081192989	0.081692548	0.064145392	0.082321456	0.081332641
NA		0.022258676	0.024116477	0.015881417	0.017234580	0.020676958	0.026995459
NA		0.027765020	0.026160599	0.025063990	0.023668748	0.023860162	0.027117193
NA		0.040093490	0.038224757	0.035274423	0.038049560	0.042791555	0.044622103
NA		0.266618003	0.260169603	0.271228063	0.249921846	0.234376213	0.218375703
NA		0.029157876	0.040128641	0.034988254	0.037513326	0.035041900	0.052203966
NA		0.026673075	0.024547279	0.024894560	0.024069924	0.034911654	0.028294551
NA		0.811240053	0.819763523	0.755006055	0.697188542	0.724094149	0.730875429
NA		0.018968169	0.018764743	0.016535900	0.016382624	0.017325474	0.025110420
	TRUE	0.025564922	0.029742856	0.022310474	0.021957245	0.020925767	0.027973605
NA		0.035883445	0.031740165	0.023997495	0.030417497	0.026064210	0.038905938
NA		0.031776230	0.034482677	0.032911453	0.030905170	0.037030357	0.042661487
	TRUE	0.039812940	0.028700992	0.029364336	0.027115619	0.029518750	0.043548840

NA		0.757550995	0.740364752	0.629586274	0.781053499	0.819192342	0.768715856
NA		0.031452717	0.035696843	0.026355912	0.045850796	0.033322511	0.039569554
NA		0.037167170	0.023503394	0.026676679	0.028234282	0.029641802	0.036060535
	TRUE	0.324341730	0.350327850	0.294015180	0.264451135	0.321540935	0.311301595
NA		0.762119621	0.821214869	0.797347580	0.810619039	0.836673947	0.786560603
	TRUE	0.237248053	0.272891887	0.250096861	0.238298580	0.250039476	0.238360637
NA		0.039030927	0.031894754	0.036943973	0.038680546	0.039885755	0.049141990
NA		0.024716717	0.033074406	0.032162729	0.025550558	0.033135546	0.051354025
	TRUE	0.058683843	0.080164992	0.069153274	0.047010595	0.044318973	0.110956978
NA		0.035468415	0.032935316	0.030709554	0.045170129	0.039713470	0.038620921
NA		0.402023176	0.409308152	0.344062510	0.350022831	0.320781243	0.345711133
NA		0.438011053	0.442123327	0.373090225	0.404491170	0.391421658	0.398655840
NA		0.119200869	0.145402889	0.120100236	0.090403208	0.098515907	0.126704445
NA		0.025803341	0.025762517	0.022851918	0.021973385	0.021742037	0.024712438
NA		0.023733382	0.028502975	0.017186739	0.028820367	0.023884177	0.026774311
NA		0.026229974	0.019446001	0.017034431	0.021130870	0.018748238	0.021015612
NA		0.020446302	0.019931340	0.017783001	0.023191473	0.016252399	0.021239444
NA		0.028828090	0.029773572	0.019266574	0.023118991	0.022840981	0.028139452
NA		0.025919703	0.025919106	0.018268441	0.023512490	0.022592713	0.028723783
NA		0.067339202	0.059439733	0.052959512	0.048444361	0.029216860	0.093066528
NA		0.065832387	0.066984183	0.058064792	0.042562298	0.054481394	0.044502067
NA		0.028311118	0.025752113	0.020817384	0.020143503	0.019453146	0.023102767
NA		0.029363964	0.026813675	0.021504570	0.032028628	0.027157787	0.034114156
NA		0.021711425	0.018826364	0.014243362	0.015054589	0.016264691	0.022825023
NA		0.047328818	0.054038135	0.057265344	0.041524131	0.041874468	0.059621053
NA		0.055707106	0.055370288	0.044461659	0.040504275	0.051818168	0.070100978
NA		0.060911916	0.048841884	0.061911137	0.051969780	0.033316559	0.070863605
NA		0.031872593	0.025049148	0.025496505	0.020977166	0.017470133	0.023370155
NA		0.727547794	0.699015182	0.719229954	0.748961833	0.753087233	0.725513921
NA		0.053443447	0.055557151	0.046907232	0.049885470	0.061069352	0.049820909
	TRUE	0.027507654	0.030791538	0.027408454	0.031279809	0.018879589	0.045122197
NA		0.033809614	0.027939415	0.026122559	0.028637096	0.019315137	0.025745365
	TRUE	0.110278561	0.085336901	0.073969804	0.083300948	0.102528502	0.091020549
NA		0.030630832	0.026255825	0.028177367	0.034531612	0.025415780	0.034321372
NA		0.024956216	0.027976858	0.025601102	0.025991699	0.025606542	0.028693925
NA		0.070569198	0.081258319	0.061667184	0.064619570	0.065903099	0.085074239
NA		0.288148098	0.273673215	0.232266894	0.295108678	0.275770543	0.256351596
	TRUE	0.413713535	0.406399424	0.354968758	0.358042066	0.379602723	0.337504019
NA		0.237678934	0.269687855	0.230093295	0.235908027	0.238846136	0.195762876
NA		0.016192758	0.014247865	0.012809602	0.014820713	0.013411425	0.018174186
NA		0.313799119	0.325829418	0.267966582	0.252734259	0.164417427	0.245623536
NA		0.088735028	0.082150355	0.072228125	0.081409887	0.086595812	0.085761081
NA		0.259942667	0.204421090	0.219091019	0.207278947	0.217628819	0.271603114
NA		0.796094632	0.863174926	0.871209564	0.810698368	0.847517361	0.813429848

NA	0.645546910	0.667080313	0.603986235	0.594497056	0.613832235	0.584153999
NA	0.035504052	0.027519108	0.027599747	0.027771523	0.038080164	0.034578704
NA	0.027597947	0.019843739	0.028643675	0.017299217	0.021278675	0.029847376
NA	0.520259915	0.532626814	0.397376093	0.488223142	0.540086355	0.579571509
NA	0.027472450	0.028512585	0.023702227	0.027646392	0.028113211	0.030686006
NA	0.625324620	0.656663378	0.669072367	0.524990007	0.572128389	0.693278120
NA	0.106179277	0.131661981	0.141153790	0.092878145	0.083749758	0.156618278
NA	0.897562752	0.918057250	0.877568410	0.873531534	0.883813613	0.860742694
TRUE	0.491404741	0.489177855	0.459381741	0.358861929	0.393788943	0.371270622
NA	0.553118622	0.583111489	0.500861364	0.491020910	0.537974189	0.484234553
NA	0.234106537	0.210447851	0.227226990	0.231169723	0.203176175	0.225181276
NA	0.612764017	0.660492618	0.671524081	0.564075190	0.651201072	0.591712477
NA	0.040974484	0.025090475	0.029710346	0.024343749	0.024107696	0.035985086
NA	0.024419300	0.028066423	0.020756086	0.020854873	0.030491313	0.026189160
NA	0.895684432	0.913142643	0.891908568	0.888863623	0.888583232	0.868559120
NA	0.043640792	0.037535669	0.039520122	0.038233173	0.045392867	0.049859281
NA	0.031240812	0.030842329	0.029068532	0.031620843	0.038272034	0.042387424
NA	0.301160436	0.368675344	0.290914413	0.312138422	0.381519693	0.326338324
NA	0.030145574	0.035791759	0.023011874	0.026236552	0.030037973	0.033780995
NA	0.035006511	0.041845366	0.032278062	0.043383760	0.038609867	0.039889881
NA	0.314523045	0.350597715	0.257875854	0.263739948	0.266960491	0.259441015
NA	0.198005322	0.368779357	0.250958347	0.264258130	0.198822136	0.193435725
TRUE	0.047451022	0.049440495	0.044884500	0.041596926	0.037309707	0.079000896
NA	0.032845341	0.034445583	0.024331472	0.024088090	0.022038715	0.039044671
NA	0.014999042	0.012792059	0.014369433	0.013600967	0.016182940	0.018742433
NA	0.023850225	0.017051968	0.017612414	0.019579626	0.019353213	0.022555862
NA	0.029314320	0.016721175	0.026001618	0.019802564	0.021711749	0.036159571
NA	0.033995854	0.035875213	0.030695889	0.022535443	0.025819951	0.023911323
NA	0.863533964	0.891607867	0.835464111	0.866399432	0.918404005	0.881183155
NA	0.021499955	0.021215222	0.026627265	0.024678463	0.023773317	0.028874672
NA	0.903889549	0.910412695	0.877976236	0.902394062	0.887152453	0.880000015
NA	0.827526282	0.881697790	0.824201913	0.786642103	0.833020709	0.806728797
NA	0.270215581	0.309795000	0.248937190	0.248862767	0.207005223	0.236694820
NA	0.070561084	0.075612134	0.067000138	0.066149437	0.062118586	0.106389169
NA	0.247640320	0.278039889	0.203757727	0.199638383	0.183072201	0.193596449
NA	0.526408207	0.492219002	0.498613364	0.458086843	0.439181286	0.392233849
NA	0.048226686	0.040000226	0.046191921	0.048997349	0.045242048	0.057463823
NA	0.110403166	0.114111298	0.103756416	0.109360156	0.096490764	0.114357069
NA	0.804881488	0.912817431	0.824930492	0.800864948	0.883590420	0.873180785
NA	0.325112036	0.327465858	0.296485059	0.213995574	0.336965674	0.255090807
NA	0.020228619	0.023458693	0.015433374	0.017035576	0.017013217	0.026314094
NA	0.039813312	0.027866892	0.031075813	0.038193836	0.028096572	0.039309588
NA	0.016023011	0.012992369	0.018290957	0.017296117	0.022055599	0.018169399
NA	0.445277098	0.491666372	0.458568368	0.437824038	0.458656995	0.547336201

NA		0.019485984	0.023813561	0.016914426	0.015991238	0.014725371	0.019048036
NA		0.034162833	0.033098737	0.030892377	0.029772445	0.023250420	0.032759009
	TRUE	0.567369745	0.550122357	0.465567340	0.469802201	0.456683285	0.478984419
	TRUE	0.048171405	0.052559224	0.042497266	0.052590713	0.059878969	0.079267816
NA		0.024455177	0.021104087	0.018203343	0.022830313	0.025364232	0.024630034
NA		0.804157720	0.822024718	0.808187944	0.805755561	0.870557819	0.852320505
	TRUE	0.133077370	0.217018258	0.128757225	0.127683566	0.184883996	0.169879549
NA		0.030279195	0.030455016	0.029913069	0.026506560	0.027946860	0.029343320
NA		0.031005413	0.023042166	0.026354860	0.023534414	0.026409967	0.038340406
NA		0.258390269	0.264323490	0.218695700	0.213774116	0.269616388	0.203589174
NA		0.022544478	0.027637620	0.022864124	0.020404886	0.023824681	0.030127682
NA		0.022471732	0.018887984	0.019042043	0.018167486	0.022627197	0.030898443
NA		0.118033549	0.105875480	0.114944489	0.139686335	0.138044444	0.110234974
NA		0.041413290	0.038818044	0.029223360	0.034711874	0.044598681	0.051298037
NA		0.022290001	0.020387982	0.017025759	0.016795558	0.018547022	0.022897365
NA		0.030979449	0.028535556	0.021831140	0.023350879	0.026790242	0.028329779
NA		0.036167911	0.028869728	0.021705107	0.024958357	0.025121815	0.032009270
NA		0.015787408	0.021109755	0.017179377	0.019952910	0.021055065	0.020592659
NA		0.264526312	0.309796156	0.279654713	0.285585573	0.410236929	0.373854763
NA		0.481737021	0.516308366	0.445466726	0.452412431	0.450902443	0.424286173
NA		0.040386338	0.027252634	0.028948546	0.037115233	0.045425402	0.043293607
NA		0.020625021	0.020973788	0.019415192	0.019517749	0.013681786	0.020126337
NA		0.874835448	0.922727402	0.851207824	0.913585649	0.875670736	0.866196729
NA		0.801396183	0.887051014	0.828763130	0.822148795	0.804171595	0.864299333
NA		0.021956153	0.019961089	0.016483049	0.020498157	0.027330043	0.028990466
NA		0.433007723	0.452948792	0.410810406	0.306226976	0.346647269	0.327463301
NA		0.231003899	0.208281409	0.175342154	0.214270285	0.191550604	0.225653484
NA		0.044907680	0.030659134	0.031191370	0.026959670	0.038593027	0.037293207
NA		0.031820772	0.028364635	0.027765612	0.031377081	0.031398540	0.031609290
NA		0.043277875	0.033055805	0.032263734	0.034096472	0.034459344	0.032397064
	TRUE	0.469406304	0.449685644	0.447980116	0.368018036	0.310287863	0.350486478
	TRUE	0.565319693	0.626238493	0.577167722	0.570581669	0.584600469	0.568408541
NA		0.040117090	0.054441696	0.034289978	0.025686507	0.030144657	0.039790675
	TRUE	0.066066148	0.069500881	0.055479874	0.041067088	0.040368753	0.076092622
NA		0.047014416	0.045733584	0.064686927	0.052465690	0.055809835	0.104309479
NA		0.565818108	0.605741289	0.533637420	0.545857357	0.574385614	0.488143433
NA		0.043078690	0.059994550	0.055186912	0.079138570	0.062256007	0.079051712
NA		0.015751332	0.013848623	0.011040277	0.015843430	0.015609380	0.014382695
NA		0.557629880	0.535423997	0.546275693	0.444900361	0.453206044	0.444629821
NA		0.920462704	0.926573005	0.913460041	0.911829531	0.896989279	0.896237392
NA		0.363887683	0.413158205	0.330090798	0.311511177	0.180963774	0.256563522
NA		0.209007889	0.194426224	0.133246660	0.096402738	0.124351756	0.150696128
NA		0.020806149	0.028606147	0.017228014	0.032409566	0.019729667	0.025810459
NA		0.020289289	0.025408768	0.019292974	0.019967933	0.021239403	0.029666066

NA	0.092523803	0.108625161	0.080345606	0.079555865	0.098851872	0.085850101
NA	0.056274708	0.053223943	0.040053674	0.052195426	0.031535911	0.062445322
NA	0.028094931	0.025666896	0.019457672	0.021981257	0.023593264	0.025769161
TRUE	0.116701659	0.117467404	0.085045242	0.094397214	0.094671888	0.101826825
NA	0.716635960	0.705489162	0.748209696	0.747326439	0.821971995	0.788309172
NA	0.026731587	0.040481024	0.032591696	0.031448172	0.026003530	0.042386542
NA	0.034951523	0.031063848	0.027972129	0.023183909	0.027773714	0.033867980
NA	0.148708341	0.117037748	0.091358259	0.103517364	0.072861380	0.072416758
NA	0.022560408	0.019215596	0.022557950	0.020282238	0.017756768	0.027088651
NA	0.906495777	0.899155608	0.890822844	0.872400769	0.899930617	0.870045367
NA	0.015975083	0.016415922	0.021186217	0.018994938	0.020604593	0.028842873
NA	0.030578362	0.024525814	0.027753077	0.027148578	0.028207241	0.052838515
NA	0.019541783	0.017053052	0.018344173	0.019952697	0.019135130	0.026026778
NA	0.871334217	0.867857572	0.829355586	0.845027053	0.813281995	0.755624260
NA	0.606902300	0.690509134	0.524456681	0.566645620	0.658618544	0.697788893
NA	0.022528269	0.017355050	0.012987759	0.017436025	0.022120344	0.020209795
NA	0.079300653	0.077394054	0.058028261	0.083991912	0.117027667	0.072194663
NA	0.047065246	0.049653294	0.031855546	0.042010843	0.029031952	0.043125185
NA	0.776578938	0.895308517	0.872433180	0.880689243	0.895123948	0.860628314
NA	0.019998528	0.027730276	0.020359130	0.020474930	0.020439491	0.026653229
NA	0.119728848	0.116605091	0.093436214	0.100151154	0.177752093	0.154245450
NA	0.018824335	0.027141985	0.023707272	0.034542736	0.027936833	0.022193787
NA	0.034749743	0.034362764	0.026143542	0.022387432	0.040478257	0.041463182
TRUE	0.461521431	0.460810758	0.393049873	0.458938570	0.457066415	0.451704146
NA	0.055937996	0.056318672	0.048425924	0.054727033	0.056830998	0.047967972
NA	0.329537815	0.322390039	0.287864325	0.286169799	0.327656851	0.285684876
NA	0.045135678	0.040321674	0.031118553	0.042435730	0.035488811	0.040172575
NA	0.038135699	0.033932279	0.026444716	0.034858711	0.036251506	0.038924265
NA	0.463301557	0.491721449	0.453010325	0.537818525	0.560565110	0.545040016
TRUE	0.017871027	0.015911904	0.012698854	0.014203913	0.013664674	0.023995349
NA	0.448084281	0.523109920	0.397735384	0.385157433	0.387888184	0.492240436
NA	0.584629691	0.593410768	0.496873587	0.513334846	0.539500350	0.499376296
NA	0.028940808	0.040351824	0.032271303	0.033016034	0.034506568	0.037319329
NA	0.035471357	0.027409952	0.031790519	0.030518012	0.025232877	0.043066180
NA	0.029449363	0.040011858	0.029067326	0.028030422	0.026244717	0.039117581
NA	0.016953228	0.023085579	0.021377301	0.016661539	0.017207355	0.025138354
TRUE	0.041578645	0.062119536	0.056466344	0.067492023	0.055791937	0.067901335
NA	0.034115005	0.040320353	0.021334183	0.033237895	0.042838921	0.036906237
NA	0.024965677	0.034626675	0.022173648	0.025399833	0.023871557	0.038083883
NA	0.047950560	0.028545505	0.026468715	0.026451036	0.032854094	0.038948616
NA	0.055925582	0.054898742	0.049765281	0.053583162	0.053247143	0.059322320
NA	0.044457619	0.038879633	0.042765750	0.043292281	0.038637729	0.083749383
NA	0.031071654	0.030613328	0.031233894	0.032541376	0.030832872	0.043027008
NA	0.217490594	0.262847470	0.207240524	0.227766615	0.247010122	0.240094198

NA		0.053956712	0.048003617	0.034177424	0.039453280	0.048647603	0.059450050
NA		0.256666084	0.301079132	0.282953768	0.327779871	0.346998063	0.340292226
NA		0.433431595	0.420109592	0.382026646	0.402117339	0.560989614	0.469639445
NA		0.487574599	0.449745331	0.437961861	0.450520180	0.380551508	0.405364448
NA		0.036671108	0.041227733	0.037171219	0.048370027	0.065338008	0.061900407
NA		0.040418156	0.051927165	0.041280528	0.046308627	0.049797165	0.059900848
NA		0.046930449	0.036087225	0.042169757	0.051209585	0.037907062	0.055632784
NA		0.449707903	0.503212084	0.429170679	0.430295682	0.497002465	0.468044298
NA		0.556799887	0.644808716	0.478700973	0.552145173	0.581631669	0.601993989
NA		0.978361702	0.980001670	0.975778617	0.977080096	0.976634802	0.974409386
NA		0.828446685	0.802962048	0.796798646	0.838690243	0.873817217	0.817064467
NA		0.033213816	0.039669190	0.024724732	0.028275456	0.021117942	0.041491763
NA		0.049135888	0.043998585	0.056614031	0.050538225	0.046254019	0.058384063
NA		0.025080766	0.031031571	0.022689934	0.022377777	0.019654965	0.031021449
NA		0.497669583	0.554195709	0.439563498	0.403313077	0.385791462	0.372708115
NA		0.221652577	0.168932527	0.155744845	0.089285354	0.076346696	0.155624966
NA		0.218930964	0.267696076	0.150826773	0.220024216	0.279861447	0.260027492
NA		0.315575040	0.354258051	0.281476232	0.287234079	0.290036277	0.282912656
	TRUE	0.190184975	0.134034570	0.107168306	0.074156985	0.076689474	0.095229101
NA		0.034320422	0.028315492	0.043023431	0.051254227	0.036074450	0.063784471
NA		0.019127569	0.016063763	0.020042468	0.022118551	0.018716815	0.027713999
NA		0.022179257	0.031953396	0.012710278	0.020396864	0.021238130	0.029486679
NA		0.231167468	0.275699077	0.222871872	0.169850052	0.208827696	0.244463905
NA		0.194958617	0.253294171	0.168630843	0.177630076	0.227782604	0.225077626
NA		0.249293595	0.140851774	0.184982437	0.133887445	0.121997502	0.148326518
NA		0.366228483	0.420493249	0.314040532	0.372067284	0.402152521	0.371120476
	TRUE	0.455665373	0.547299757	0.430328825	0.493181391	0.535074058	0.465634482
NA		0.241062689	0.223738924	0.167570435	0.151673949	0.133401509	0.129569450
NA		0.622490933	0.696599517	0.631590932	0.700445322	0.750580725	0.692609651
NA		0.033163436	0.040572369	0.030227254	0.030403577	0.037530427	0.034526033
NA		0.039964337	0.030280813	0.026056649	0.036832305	0.035012687	0.042811482
NA		0.027654178	0.024344986	0.018875460	0.023956829	0.022819276	0.028958173
NA		0.015618213	0.019190523	0.015257385	0.017144021	0.016333452	0.017982129
NA		0.024698200	0.024142541	0.024248452	0.029715654	0.027422525	0.041348995
NA		0.864899957	0.874198349	0.863036873	0.864441849	0.854309351	0.849362185
NA		0.048017519	0.029903534	0.035238763	0.035255147	0.033832795	0.041458136
NA		0.591139635	0.600822329	0.620103330	0.573010138	0.525196613	0.469496784
NA		0.045183864	0.029121712	0.038310141	0.032218258	0.027458472	0.043935855
NA		0.057041730	0.072660430	0.052523761	0.047680336	0.046555425	0.078715214
NA		0.021792315	0.017165962	0.018869567	0.020590383	0.019016217	0.025484063
NA		0.275091846	0.378809670	0.268865337	0.208350981	0.296049184	0.347665547
NA		0.268418325	0.350866324	0.259074749	0.270693425	0.211220062	0.254656728
NA		0.769383560	0.817593411	0.775782127	0.833905506	0.844840447	0.780481270
NA		0.031599536	0.025809518	0.031782728	0.023342058	0.024115756	0.026421403

NA	0.044680595	0.059179646	0.053458438	0.042564536	0.033486000	0.068942301
NA	0.036810453	0.042903400	0.037540246	0.032949391	0.040462586	0.050547812
NA	0.020077581	0.021177782	0.020848297	0.016988897	0.015599930	0.025201854
TRUE	0.560972377	0.632766838	0.564093713	0.565741887	0.706571488	0.651077392
NA	0.034925671	0.043505332	0.039450118	0.029440219	0.021912400	0.062930324
NA	0.038190032	0.040124462	0.033889477	0.036392651	0.028209382	0.048561875
NA	0.956323713	0.958314700	0.942960584	0.949316702	0.961806752	0.907434494
NA	0.048025801	0.036836532	0.044670673	0.041283492	0.037246207	0.039733700
NA	0.066117562	0.086071191	0.072583645	0.059850625	0.050251454	0.107291452
NA	0.194164956	0.211907777	0.191939965	0.203228263	0.149404835	0.192382061
NA	0.059401555	0.056353063	0.035368989	0.044981114	0.044617266	0.064171413
NA	0.501098329	0.500762142	0.494944995	0.468776429	0.488028220	0.469743510
NA	0.218117109	0.191943918	0.171251272	0.160199065	0.128337082	0.165409555
NA	0.057271607	0.045715928	0.040914660	0.032855172	0.047823515	0.048710606
NA	0.684633985	0.703934715	0.633836629	0.619246823	0.641105980	0.692574284
NA	0.052699652	0.058354418	0.050009771	0.044086112	0.050087766	0.060237115
NA	0.972723344	0.981501988	0.931528746	0.976722927	0.973534579	0.955143296
NA	0.516137501	0.304708682	0.291451927	0.431009606	0.267776818	0.211852029
TRUE	0.025982461	0.031060841	0.026626784	0.024542104	0.027880403	0.036643187
NA	0.051204468	0.054850659	0.046089898	0.051848497	0.050963138	0.074700746
NA	0.036575951	0.037105381	0.038817941	0.027990020	0.026834313	0.056480085
NA	0.023089763	0.019363511	0.024710985	0.022863502	0.025437035	0.039364365
NA	0.102644465	0.101258426	0.092638828	0.126538175	0.131281307	0.123365625
NA	0.074170584	0.080284066	0.095809544	0.088887558	0.100418527	0.085222929
NA	0.362409593	0.314241413	0.404500601	0.215598292	0.452113228	0.259364014
NA	0.024837587	0.039612568	0.036409933	0.035113414	0.030816183	0.042904677
NA	0.114921040	0.122914054	0.098541964	0.074182814	0.068469927	0.187524620
NA	0.740536630	0.789142295	0.778943322	0.713398022	0.789335547	0.706180941
NA	0.030265615	0.027723493	0.026172677	0.023307326	0.021451114	0.028822841
NA	0.151462460	0.162961594	0.109531094	0.137279496	0.199540107	0.177853550
NA	0.515386323	0.510891879	0.472001352	0.505724542	0.571042374	0.509353247
TRUE	0.149758169	0.146548121	0.111944408	0.140490256	0.241317279	0.172379268
NA	0.744880918	0.784630750	0.664382058	0.772198867	0.755659451	0.725424013
NA	0.499025821	0.545047463	0.588764421	0.543031965	0.541894052	0.548129993
NA	0.526598395	0.579472022	0.489824879	0.418666254	0.477670074	0.423960187
NA	0.099344603	0.108753577	0.086619329	0.100302958	0.098975008	0.102331118
NA	0.042174059	0.028855336	0.033853093	0.032972825	0.029939996	0.035056660
TRUE	0.043260861	0.051452573	0.020376385	0.027700568	0.030528758	0.035394350
NA	0.837835262	0.828113717	0.766312602	0.815762186	0.830379404	0.787710653
NA	0.455617871	0.580066345	0.433504357	0.496345592	0.514338188	0.510839972
NA	0.050808998	0.037418988	0.037994814	0.044138815	0.035638019	0.054690858
NA	0.542854950	0.524974248	0.486414315	0.503033873	0.523305995	0.491154980
NA	0.047779865	0.044086135	0.044004179	0.036326666	0.034877260	0.072568302
NA	0.032944443	0.041865581	0.039094883	0.022910365	0.019968700	0.031981830

NA		0.029744695	0.025693085	0.018066713	0.024036768	0.020508482	0.029337314
NA		0.322094128	0.344381600	0.283523457	0.322304396	0.347019789	0.313681235
NA		0.035284075	0.039688861	0.047408116	0.035020370	0.031483546	0.048728016
NA		0.487332579	0.575767697	0.498588212	0.471092479	0.623626334	0.584641944
NA		0.030825523	0.036313015	0.035022361	0.029696307	0.032127237	0.044828146
NA		0.291406071	0.341515077	0.288985817	0.288847681	0.281396770	0.304393466
	TRUE	0.065273704	0.057929812	0.055234462	0.048445424	0.049963925	0.058448731
NA		0.738035690	0.769162050	0.676666171	0.785236413	0.872282324	0.818585038
NA		0.021816763	0.022677668	0.015124855	0.023690228	0.017806748	0.022370810
NA		0.458437315	0.518768404	0.470684041	0.446593738	0.542052213	0.571732309
NA		0.482791510	0.482308268	0.377398762	0.492935764	0.543533518	0.554894355
NA		0.015667878	0.016804944	0.018756226	0.022210022	0.016830285	0.021474076
NA		0.020116050	0.019396402	0.024157470	0.020149680	0.021178153	0.033620208
NA		0.025470255	0.027154573	0.016779923	0.029535240	0.023281303	0.026113935
NA		0.507679210	0.527224217	0.498356903	0.344654404	0.427502873	0.391364957
NA		0.028741441	0.027712313	0.024443108	0.022869349	0.027992284	0.027947983
NA		0.768636202	0.776088773	0.683879713	0.698336932	0.708689722	0.695200342
NA		0.488650018	0.454818320	0.416249947	0.469344266	0.443991969	0.397111621
NA		0.540640250	0.572848717	0.467060259	0.527042221	0.687956442	0.625691002
NA		0.018217897	0.018852234	0.020720816	0.017972169	0.018448627	0.029584783
NA		0.014476815	0.016938161	0.017010159	0.017763432	0.012897689	0.019409726
NA		0.242993113	0.290018205	0.222528765	0.237005024	0.261081472	0.272399597
NA		0.380916525	0.417520410	0.322149451	0.373457194	0.511677025	0.390040199
NA		0.029897689	0.026879356	0.029826734	0.025499894	0.022919566	0.043188115
	TRUE	0.181756137	0.162714659	0.209254734	0.119871952	0.078611702	0.127601628
NA		0.709764868	0.831022022	0.776885330	0.795513930	0.799914318	0.765162774
NA		0.033665289	0.027121733	0.028503093	0.022616734	0.023724888	0.041046193
	TRUE	0.130162252	0.172111365	0.116009924	0.122259163	0.101197689	0.153817322
NA		0.260713234	0.251811343	0.200550188	0.177006171	0.260935270	0.162140811
NA		0.022245831	0.017159847	0.015988649	0.019376331	0.018662117	0.019826540
NA		0.012917760	0.014001826	0.012585299	0.017372404	0.014705057	0.015438024
NA		0.051167159	0.026377662	0.034668034	0.041350836	0.044618539	0.045644547
NA		0.026803208	0.023604842	0.023409510	0.021605018	0.017580110	0.025139450
NA		0.028555524	0.021531751	0.019378749	0.019946962	0.019720401	0.029413852
NA		0.045470069	0.048700720	0.043293126	0.041578431	0.036217346	0.047467363
NA		0.028964079	0.026818421	0.022961500	0.033848919	0.026326050	0.024119782
NA		0.813208574	0.868399890	0.801889379	0.794756407	0.829263699	0.814232770
NA		0.013177199	0.014964178	0.014284737	0.013626208	0.013379972	0.021886061
NA		0.814197193	0.879813469	0.819959782	0.857138993	0.853531061	0.785312440
NA		0.037960891	0.040536119	0.030763368	0.039642227	0.026319439	0.032132690
NA		0.025083430	0.025272221	0.020414221	0.030414527	0.022161651	0.029340245
NA		0.031211425	0.027684558	0.023510199	0.035559945	0.033079280	0.031125287
NA		0.022995723	0.024557346	0.021431194	0.026311146	0.019244574	0.025583466
NA		0.031267115	0.041497520	0.026826785	0.031314229	0.033031594	0.043643584

NA		0.638775614	0.704924298	0.598060309	0.598233505	0.601779378	0.750068882
NA		0.486150616	0.532086686	0.471776652	0.508246829	0.538964186	0.587762094
NA		0.401216328	0.519030833	0.417964090	0.396416373	0.516096436	0.431842278
NA		0.017252039	0.023937871	0.015456373	0.017017360	0.019992166	0.022741515
NA		0.564784616	0.591368877	0.525034403	0.465672965	0.496591010	0.458138476
	TRUE	0.041975158	0.058150360	0.041406929	0.053192880	0.052154616	0.072132617
	TRUE	0.672114261	0.678804854	0.587752687	0.618648375	0.699449712	0.647022609
NA		0.054766705	0.049587220	0.035510295	0.053284679	0.052322864	0.058636679
NA		0.616809991	0.646572161	0.563552679	0.558989116	0.584710514	0.541578254
NA		0.709282207	0.799637534	0.810947802	0.737102376	0.792301028	0.720815275
NA		0.066349731	0.080880822	0.061382289	0.058196962	0.058163558	0.099880392
NA		0.390202641	0.426464537	0.349768447	0.354337910	0.455529489	0.409034078
NA		0.015115164	0.010111241	0.012073807	0.016271338	0.015711530	0.016470058
NA		0.127713105	0.197208470	0.167076893	0.099916503	0.107306536	0.148183162
NA		0.037561219	0.026655986	0.031951042	0.029903964	0.025292624	0.039626672
NA		0.566487481	0.635372509	0.552858509	0.609753666	0.673517516	0.673855493
NA		0.129030370	0.083721240	0.077753281	0.117273518	0.154260346	0.093807838
NA		0.039221568	0.040240590	0.041181795	0.028020459	0.036773304	0.034178560
NA		0.019825827	0.028177062	0.021226213	0.026137339	0.027442023	0.043038153
NA		0.639512494	0.625433221	0.603451239	0.661949037	0.684178981	0.565919017
NA		0.056229692	0.035322922	0.062129489	0.062523202	0.043620109	0.067192814
NA		0.043699462	0.043193210	0.036265164	0.038989623	0.039676855	0.048526719
	TRUE	0.619009898	0.653676359	0.585041732	0.609678053	0.469707740	0.620890184
NA		0.876611320	0.028634151	0.455900132	0.025042569	0.841612342	0.031961177
NA		0.856560149	0.862606849	0.855895865	0.854145920	0.873646410	0.845201343
NA		0.054095701	0.099779300	0.039364653	0.050201793	0.052291532	0.064808093
NA		0.633711848	0.693588276	0.669018462	0.591902684	0.611502096	0.631114942
NA		0.035796799	0.032188701	0.026211270	0.030520954	0.028813619	0.051919353
NA		0.029779803	0.035019724	0.029478853	0.035626184	0.032394166	0.054834430
NA		0.038314687	0.053723625	0.041149641	0.035136361	0.040362904	0.059252950
NA		0.022486292	0.024405458	0.025953551	0.029451322	0.030163161	0.033272135
NA		0.025063196	0.026158395	0.023447256	0.021701289	0.022808569	0.031802755
NA		0.037950127	0.041726826	0.036080463	0.033734597	0.025608876	0.041812364
NA		0.090254960	0.111639231	0.077081630	0.107806555	0.057380061	0.088589216
NA		0.016495224	0.012818318	0.011903865	0.015623009	0.018707832	0.017342749
NA		0.453868597	0.297615035	0.377244628	0.414432330	0.541306875	0.239843392
NA		0.056278788	0.061576287	0.054913037	0.043765897	0.052224425	0.074597279
NA		0.043520263	0.050101785	0.042536098	0.050242692	0.043392886	0.060515966
NA		0.054404002	0.040199017	0.044133062	0.024867614	0.027321580	0.034301922
NA		0.186422575	0.238512964	0.136194500	0.171251018	0.213300960	0.198574353
NA		0.065267906	0.066904750	0.062356893	0.060079981	0.067513987	0.065960745
NA		0.152621427	0.131896786	0.116912982	0.143736028	0.170016859	0.178753354
NA		0.703138648	0.757077972	0.651402594	0.623108524	0.741398770	0.695154835
NA		0.195119635	0.165473403	0.155586748	0.171843832	0.200935709	0.227599494

NA		0.746523206	0.73638393	0.75367473	0.710023803	0.736686292	0.68718291
NA		0.02593131	0.02172918	0.02891712	0.02612977	0.026508576	0.031562761
NA		0.44535160	0.52798589	0.44456472	0.45680041	0.462762810	0.45829810
NA		0.50942301	0.53847523	0.44518296	0.51533805	0.598061661	0.54042230
NA		0.05152685	0.05116553	0.042585052	0.04881854	0.041443013	0.062306668
NA		0.02264912	0.02150991	0.02485931	0.02697570	0.023708862	0.03331943
NA		0.80220640	0.84625275	0.74413685	0.63380164	0.753332895	0.69274160
NA		0.04628370	0.03681537	0.026776841	0.03091712	0.045376293	0.047124703
NA		0.47842977	0.56357145	0.44200759	0.53742068	0.485561642	0.49287163
NA		0.01359551	0.01389859	0.01415803	0.02064929	0.013561979	0.02619802
NA		0.08520646	0.06229006	0.06838714	0.08711109	0.055183744	0.09148433
NA		0.53649769	0.50611575	0.50802104	0.44498147	0.468102369	0.42877176
NA		0.35382915	0.33493416	0.32475417	0.30838957	0.330574410	0.32087782
NA		0.22370328	0.226530677	0.16115024	0.16696712	0.199296555	0.20540371
	TRUE	0.05468701	0.05076060	0.05968602	0.05115073	0.040686784	0.08714787
NA		0.58889953	0.64998719	0.555384716	0.56859631	0.585786934	0.59807627
	TRUE	0.11442117	0.142910935	0.09696278	0.12976041	0.177081089	0.13003134
NA		0.51625939	0.53240117	0.452057886	0.46604170	0.491880610	0.48524873
NA		0.39665022	0.38455590	0.35439522	0.36914407	0.437672868	0.35049923
NA		0.03002645	0.02186389	0.02008446	0.02065121	0.019025638	0.02612870
NA		0.58081889	0.70459177	0.62724499	0.58515807	0.626144352	0.62451716
NA		0.11774965	0.16853000	0.12002990	0.10369233	0.091749912	0.11962459
NA		0.07188394	0.06258492	0.06615445	0.04593132	0.058248133	0.05848090
NA		0.02288010	0.02052874	0.02516526	0.02266140	0.017255270	0.03224544
NA		0.92824084	0.95913521	0.914582647	0.96346788	0.967700934	0.92767485
NA		0.03631763	0.04259032	0.02974369	0.03931523	0.043836848	0.05165047
NA		0.01897001	0.01634115	0.032840822	0.025247107	0.027183737	0.02166314
NA		0.83631974	0.934260775	0.89146064	0.911326011	0.906473278	0.88971457
NA		0.85557562	0.92709431	0.87045591	0.88719772	0.880954220	0.868322830
NA		0.85731164	0.89024169	0.86777842	0.87213644	0.930195933	0.86769376
NA		0.80569965	0.823841293	0.74827869	0.79834557	0.785949998	0.77164445
NA		0.05427638	0.04494539	0.03288693	0.046637850	0.046604905	0.03729586
NA		0.02091738	0.02080453	0.02281127	0.02630964	0.020882524	0.02513928
NA		0.51834265	0.54117385	0.48916374	0.44760528	0.499653402	0.43628642
NA		0.52616554	0.49635605	0.526099724	0.50774533	0.482669031	0.40980370
NA		0.04130001	0.04126684	0.03682600	0.03810811	0.029614274	0.04615516
NA		0.38898160	0.46818563	0.38094352	0.357503875	0.336691473	0.39568630
NA		0.02739329	0.02836335	0.02477859	0.03233004	0.031294265	0.03158559
NA		0.07379554	0.06838574	0.05609804	0.07034499	0.064379808	0.05635386
NA		0.408272613	0.448693664	0.35113419	0.41383463	0.459801316	0.41841202
NA		0.03319389	0.03622658	0.02080082	0.02532613	0.021338587	0.02521744
NA		0.04701360	0.06042399	0.04955192	0.06356905	0.058233963	0.06156043
NA		0.26312346	0.30722761	0.29540577	0.23081986	0.293362796	0.36280958
NA		0.89179582	0.886895357	0.86323549	0.88218267	0.879244706	0.86590271

	TRUE	0.060622834	0.056310533	0.046537607	0.033724805	0.038344075	0.062458983
NA		0.283023494	0.312543818	0.230771266	0.267205341	0.303912579	0.251309838
NA		0.042325709	0.025683609	0.025302956	0.022879499	0.022405038	0.028503919
NA		0.424028568	0.501114535	0.344763521	0.452114832	0.497822513	0.430131206
NA		0.031409196	0.036571607	0.028189966	0.024462825	0.028748349	0.039206672
NA		0.320126756	0.355627881	0.267525571	0.274512920	0.354094137	0.315106702
NA		0.349533605	0.302449960	0.330440106	0.249285745	0.214685411	0.272246967
NA		0.027061716	0.038559955	0.027208489	0.029021742	0.025092554	0.037302703
NA		0.100863266	0.132604209	0.099877638	0.114271507	0.116835525	0.108540143
NA		0.034478284	0.029002741	0.030131895	0.024461738	0.027022655	0.047793872
NA		0.033823045	0.033562081	0.026190899	0.027659411	0.031876073	0.030582009
NA		0.039117129	0.034915631	0.028553978	0.034659926	0.030709520	0.040599633
NA		0.091122339	0.076059043	0.083923287	0.088912857	0.075116124	0.123825434
NA		0.902019621	0.922517959	0.876304639	0.872151306	0.891718978	0.855898984
NA		0.040594716	0.040918854	0.041017640	0.048453490	0.037884248	0.050644100
NA		0.029014034	0.030097701	0.034832533	0.027479661	0.021949174	0.037839943
NA		0.970628074	0.971499277	0.964734744	0.968273843	0.970506162	0.950158774
	TRUE	0.053143890	0.059111402	0.044445962	0.043184300	0.047900717	0.057482333
NA		0.026620174	0.028413932	0.028833551	0.032437277	0.056825991	0.035727155
NA		0.770869801	0.844389543	0.910892516	0.881622576	0.930200365	0.890537982
NA		0.033189620	0.019485161	0.027639845	0.027551592	0.020611387	0.035387190
NA		0.052779618	0.040825022	0.044338145	0.049685812	0.046775102	0.054153860
NA		0.938393260	0.946615861	0.920567088	0.930096402	0.944384090	0.886072041
	TRUE	0.034453223	0.039549284	0.042596697	0.027073928	0.017987393	0.040213888
NA		0.595599158	0.497512016	0.522051369	0.582163356	0.632602117	0.572215504
NA		0.428521845	0.467280867	0.365820035	0.403608749	0.428203810	0.411871934
NA		0.025371198	0.021450933	0.017772771	0.025356740	0.032314704	0.024122507
NA		0.021484480	0.015149128	0.019325615	0.019163906	0.023928740	0.022436992
NA		0.441123648	0.398495579	0.389681717	0.374346633	0.386716006	0.317265312
NA		0.058219912	0.067581937	0.058564023	0.049746151	0.048280063	0.101469408
NA		0.054708083	0.055117779	0.046924230	0.055367345	0.041994771	0.057721333
NA		0.042093385	0.047283579	0.036836349	0.055464427	0.049929695	0.029306484
NA		0.042115222	0.044676490	0.039131343	0.040189627	0.038608969	0.055228747
NA		0.486072486	0.437972402	0.376857113	0.424802013	0.452997867	0.420036037
NA		0.030334216	0.024905751	0.017763166	0.014367370	0.015251647	0.026790851
NA		0.024238403	0.019616396	0.018614002	0.017754756	0.020749462	0.030819013
NA		0.837664713	0.815924092	0.786805999	0.807963550	0.855417188	0.838498783
NA		0.381471222	0.311963637	0.126545619	0.102852194	0.088032196	0.338760459
NA		0.023096929	0.033705024	0.022071265	0.027751256	0.024880364	0.024219737
NA		0.064286573	0.054644886	0.055482204	0.063734855	0.038675668	0.096798099
NA		0.056656459	0.063228119	0.042408316	0.054630348	0.051523491	0.063961337
NA		0.192337329	0.133197026	0.236990470	0.181360789	0.104647245	0.107779208
NA		0.481336008	0.840566453	0.522577580	0.659904313	0.508334127	0.725269887
NA		0.819883041	0.830466737	0.786992379	0.804217723	0.767499494	0.802802929

NA		0.026520061	0.022706656	0.016266833	0.026415910	0.016921805	0.029113356
NA		0.029315816	0.019169004	0.036661653	0.035792605	0.023499342	0.030248856
NA		0.561779640	0.591324155	0.478736448	0.538792211	0.541900960	0.531350667
NA		0.743461634	0.826498838	0.710623939	0.723426008	0.702089696	0.705575343
NA		0.812500536	0.832338950	0.788843995	0.793279189	0.814290412	0.723189275
NA		0.021406954	0.023266732	0.016180005	0.020714005	0.014269006	0.020885741
NA		0.927757563	0.867867439	0.656728715	0.922398666	0.889154875	0.707860638
NA		0.034969997	0.041618376	0.031804161	0.035746532	0.032926557	0.060572465
NA		0.064637275	0.048931379	0.040614534	0.045121928	0.066377796	0.049067201
	TRUE	0.807210979	0.805860656	0.709939968	0.741857848	0.761502182	0.816814233
NA		0.135006862	0.129533553	0.121465604	0.117485519	0.122618056	0.121319995
	TRUE	0.523272363	0.568130992	0.467355550	0.425580485	0.523498901	0.441992951
NA		0.041026054	0.032272462	0.032704599	0.027582861	0.035143328	0.042941569
NA		0.541309990	0.550357907	0.404448176	0.442183312	0.491770920	0.449734952
NA		0.683949483	0.747065452	0.681765609	0.629636841	0.597233440	0.626739523
NA		0.403388452	0.440136529	0.387875363	0.398197721	0.441853764	0.372105410
NA		0.859076274	0.843998987	0.830472309	0.827996019	0.840804764	0.806070870
NA		0.036599633	0.041405539	0.039079685	0.051372816	0.033373197	0.047341803
NA		0.854340183	0.877628401	0.808594450	0.853521974	0.825445927	0.836402917
NA		0.283683398	0.304094858	0.310472155	0.258025204	0.201549917	0.230892564
NA		0.046670888	0.063281115	0.045409635	0.050372948	0.039568148	0.080687805
NA		0.124990816	0.097613630	0.090991894	0.116056986	0.078574573	0.121533150
	TRUE	0.023932058	0.029372291	0.027267185	0.020862897	0.020445284	0.037311101
	TRUE	0.018581077	0.020018026	0.019860481	0.022981839	0.016896468	0.023563697
	TRUE	0.911507066	0.942417492	0.915506944	0.941508080	0.935464604	0.929073477
NA		0.055965846	0.057307779	0.033561647	0.041004242	0.028274284	0.031239222
NA		0.858792195	0.887626993	0.836058352	0.856519606	0.823257365	0.838334192
NA		0.055087265	0.073701581	0.063607993	0.058849323	0.069347049	0.061390554
NA		0.015347873	0.012852963	0.019606332	0.020641217	0.019064058	0.022415717
NA		0.027305021	0.038522370	0.029201980	0.026691541	0.029065521	0.045308203
NA		0.359020233	0.397938799	0.327125045	0.353603520	0.469461000	0.404472400
NA		0.048624551	0.064792927	0.052992520	0.046989724	0.075208057	0.082313468
NA		0.849191687	0.866329455	0.853896951	0.824426896	0.830929477	0.843738334
NA		0.020081987	0.016656580	0.022576118	0.020730079	0.023844927	0.025334590
NA		0.018851076	0.020768469	0.022171011	0.020072531	0.018798983	0.032076893
NA		0.836242776	0.873407196	0.851045301	0.858873009	0.832155898	0.849154533
NA		0.075457773	0.063685435	0.056411202	0.052613930	0.052468375	0.067811195
NA		0.602721399	0.660874088	0.505260097	0.509043632	0.514144228	0.522260024
NA		0.051055518	0.108233497	0.074644902	0.051735595	0.050270062	0.061807309
NA		0.365156521	0.439385595	0.321691604	0.244520995	0.199724551	0.189154256
NA		0.369701967	0.396617500	0.325315614	0.391675837	0.509547849	0.384943800
NA		0.044002442	0.048471189	0.029695737	0.036641534	0.035737652	0.034248341
NA		0.916726157	0.934478327	0.881987869	0.890867177	0.908808963	0.891055832
NA		0.726366206	0.751977533	0.735893420	0.699846168	0.785881445	0.781147323

	TRUE	0.877675144	0.877464217	0.859958797	0.878817748	0.892898472	0.86808192
	TRUE	0.394754721	0.454514477	0.375169899	0.382597170	0.347444633	0.36272224
NA		0.027909238	0.022466361	0.020576349	0.024437488	0.025970391	0.02486963
NA		0.017527244	0.019369698	0.015982017	0.019133354	0.018747242	0.02890229
NA		0.218814417	0.319168082	0.217510540	0.291601275	0.288094342	0.32890242
NA		0.771333388	0.770458640	0.782088834	0.831567989	0.880004824	0.69459530
NA		0.380135893	0.418723112	0.354283390	0.348215908	0.365783633	0.34125256
NA		0.323546926	0.356354924	0.277538368	0.268608594	0.297781433	0.30323772
NA		0.804634002	0.823350350	0.750734754	0.799308959	0.765895997	0.829199264
NA		0.020950281	0.020409064	0.019319556	0.023723995	0.019881216	0.02776035
NA		0.528201971	0.597416681	0.508585998	0.498849930	0.525822854	0.56248037
NA		0.194836674	0.162301791	0.153995311	0.160429344	0.161848779	0.17012690
NA		0.047829916	0.045493374	0.045787239	0.036437194	0.030802412	0.06449386
NA		0.019935554	0.019453786	0.018525171	0.018580051	0.018186692	0.02176630
NA		0.023182220	0.019509058	0.022185804	0.020252808	0.021363298	0.03283254
NA		0.893579966	0.868468786	0.881107330	0.895302936	0.891642500	0.826871386
NA		0.051591854	0.054733172	0.043576028	0.049925737	0.041645595	0.04940662
NA		0.740237606	0.748181039	0.708943606	0.701374324	0.723301667	0.65708417
	TRUE	0.076088874	0.145970005	0.091476013	0.079628522	0.068833544	0.098870008
NA		0.807004263	0.845354254	0.805736992	0.816100662	0.830050392	0.77131758
NA		0.208585190	0.203479742	0.187224700	0.161966542	0.166157919	0.16838467
NA		0.059895171	0.050774531	0.054557564	0.050550761	0.074591729	0.06511696
NA		0.511031127	0.591882428	0.484024087	0.507331426	0.499968356	0.50461547
NA		0.830639944	0.913411683	0.844363124	0.843991444	0.825093399	0.83900399
NA		0.060160347	0.079780416	0.077315733	0.071971667	0.066534820	0.11302089
NA		0.034480862	0.026150569	0.036987326	0.025606818	0.019338670	0.03067873
NA		0.054394333	0.042914240	0.044550023	0.048574826	0.075921334	0.05166465
NA		0.019801856	0.013487093	0.018378892	0.020259914	0.015235496	0.01994329
NA		0.220323475	0.228369242	0.202937525	0.204559890	0.232384309	0.17763275
NA		0.627375585	0.653423298	0.605950635	0.562488988	0.589978066	0.58036913
NA		0.419411399	0.456959319	0.402608618	0.438861069	0.477501761	0.48203451
NA		0.046293270	0.063187456	0.044490111	0.041072501	0.037195469	0.05379604
NA		0.767892315	0.812860045	0.739822823	0.752402724	0.772105545	0.76416293
NA		0.024675001	0.023075550	0.022450455	0.027700804	0.022970895	0.03264104
NA		0.792988416	0.818479430	0.783555999	0.721915432	0.754366487	0.765487901
NA		0.054590513	0.052896039	0.041201931	0.053619805	0.046159486	0.05947124
NA		0.033056443	0.041531331	0.041150253	0.037961859	0.035991315	0.04514842
NA		0.154976716	0.122590129	0.120221885	0.170199116	0.169648858	0.15659506
NA		0.052530945	0.056126077	0.038518422	0.047683441	0.041909064	0.044329026
	TRUE	0.103764495	0.118668104	0.100965935	0.135676879	0.116108354	0.09168105
NA		0.519278459	0.518139241	0.470079522	0.468430114	0.493826161	0.52158109
NA		0.050811043	0.073530180	0.049731106	0.044914622	0.046748988	0.098898196
	TRUE	0.125961365	0.144151064	0.119701580	0.117658777	0.136107046	0.10331270
NA		0.021786324	0.018638256	0.027283450	0.026907095	0.021347601	0.03344227

NA		0.837435457	0.810866093	0.753079401	0.812397917	0.776493024	0.804265645
NA		0.887114344	0.874840877	0.874273894	0.879086973	0.884251955	0.870681241
NA		0.052675743	0.047263724	0.053777457	0.055305169	0.038201341	0.078220705
NA		0.016771401	0.016822675	0.018699287	0.017514452	0.018406645	0.021668194
NA		0.036300473	0.039418965	0.029318044	0.030849067	0.028335949	0.036619363
NA		0.031088459	0.047263182	0.022634237	0.024390192	0.027695413	0.028253054
NA		0.438837007	0.473630655	0.393042888	0.342952740	0.371094810	0.378817590
NA		0.036844606	0.033468893	0.030981062	0.029604638	0.031908616	0.046446561
NA		0.033769518	0.031979214	0.028075365	0.036042176	0.027777848	0.037569153
NA		0.027485689	0.038819652	0.024445770	0.022210379	0.022023906	0.043349309
NA		0.035258251	0.038087397	0.024084429	0.032529001	0.019507697	0.040284907
	TRUE	0.154630688	0.161074362	0.127898237	0.101291167	0.081653853	0.100204608
NA		0.832538442	0.780026944	0.787793776	0.745428798	0.746562697	0.792886783
NA		0.013876494	0.012091987	0.010180913	0.010025118	0.009200418	0.015153530
NA		0.302057161	0.348706408	0.285036171	0.247198495	0.255176912	0.271919888
NA		0.046890660	0.045420844	0.038582139	0.058548483	0.068952234	0.062906223
NA		0.301080922	0.300530685	0.252768403	0.240796446	0.209735114	0.212079604
NA		0.061036722	0.075125656	0.065042905	0.044382239	0.036626532	0.098430257
NA		0.029693104	0.029422468	0.029662115	0.030204695	0.026840956	0.037125000
NA		0.040960374	0.028595324	0.033620105	0.031001669	0.038480850	0.051845130
NA		0.027095362	0.030982585	0.029095351	0.025837582	0.036067598	0.035021859
NA		0.099601105	0.131680899	0.113237331	0.091055739	0.177226944	0.145557071
NA		0.864363447	0.919073006	0.848594628	0.876799534	0.914423002	0.855374749
NA		0.043515008	0.035139784	0.042490737	0.042109950	0.035798153	0.085754174
NA		0.028298088	0.029585445	0.019680702	0.025658536	0.023791819	0.034548507
NA		0.066912998	0.094830677	0.060440466	0.082880117	0.031189157	0.078490426
NA		0.033307676	0.036195300	0.034545642	0.034526496	0.025021749	0.046797324
NA		0.410628425	0.400961128	0.385508733	0.421866287	0.627559337	0.514644251
NA		0.035280692	0.045149143	0.039815017	0.027276965	0.032005815	0.095552361
NA		0.033424113	0.026746279	0.039568474	0.029272671	0.037201139	0.031739042
	TRUE	0.061016495	0.062256470	0.048660786	0.048803718	0.047010679	0.053903888
NA		0.025607910	0.015199027	0.023132179	0.020614803	0.023779023	0.023624628
NA		0.282707325	0.347097692	0.278735386	0.282368997	0.393326068	0.346507845
NA		0.920569911	0.914684687	0.908315903	0.910231731	0.929576524	0.900419443
NA		0.369974858	0.442613072	0.321275661	0.375764846	0.438878769	0.378369432
NA		0.251955480	0.307689830	0.204471614	0.182233237	0.142997104	0.153850567
	TRUE	0.434654747	0.426857867	0.389738748	0.429372050	0.561194163	0.461510224
NA		0.144142586	0.164590822	0.160361479	0.159684710	0.201307378	0.186888787
NA		0.879310344	0.907326710	0.834325305	0.848036453	0.868193139	0.831478950
NA		0.037880388	0.051204669	0.046409626	0.041318161	0.029938492	0.068943331
NA		0.089027554	0.074236248	0.064206724	0.074842605	0.085885172	0.096304405
NA		0.631183237	0.633842226	0.622363366	0.613413447	0.635265999	0.589657659
NA		0.264834258	0.242807783	0.087207761	0.085945816	0.068527017	0.251225722
NA		0.024292883	0.020496193	0.017326836	0.022356792	0.021930439	0.021502126

NA		0.530331968	0.606053759	0.489801369	0.533707150	0.456634746	0.459262413
NA		0.020112393	0.025926591	0.021098963	0.018724609	0.024061807	0.030268397
NA		0.037454484	0.029853775	0.022563695	0.024908484	0.030556573	0.030162895
NA		0.025156516	0.028685367	0.022007145	0.029168514	0.023054828	0.030885995
NA		0.068438560	0.071502822	0.048367925	0.063557798	0.051824957	0.066822245
NA		0.036131771	0.041756003	0.035227164	0.033546232	0.037913184	0.045304695
NA		0.017524597	0.015868181	0.012990851	0.013989960	0.014053090	0.017320497
NA		0.705434020	0.720020018	0.665894984	0.698528357	0.734036070	0.703023802
NA		0.029499832	0.030236382	0.024007774	0.021589504	0.026060680	0.043456450
NA		0.024407479	0.020987208	0.017526708	0.021205464	0.021738833	0.025435040
NA		0.168446063	0.193385704	0.153120251	0.185183653	0.159856465	0.160859139
NA		0.949227628	0.969544840	0.948727998	0.945004384	0.847895824	0.938572277
NA		0.036797842	0.048349666	0.041343500	0.032609177	0.033218172	0.074095656
NA		0.023334164	0.020524130	0.021669729	0.034334761	0.030779056	0.035569618
NA		0.796483670	0.833500569	0.745470577	0.783110847	0.780335139	0.793938975
	TRUE	0.544521464	0.662860846	0.539848708	0.576831525	0.583240144	0.553950460
NA		0.041364777	0.032250344	0.034529981	0.044493690	0.040274539	0.039075130
NA		0.032463442	0.021643310	0.022375237	0.027411137	0.016496770	0.034977134
	TRUE	0.053134053	0.055122444	0.031810408	0.043004776	0.034474841	0.052479114
	TRUE	0.555587965	0.552738914	0.434122314	0.395900198	0.379290072	0.517186258
NA		0.125829069	0.177987794	0.130266068	0.111952716	0.121129745	0.156554560
NA		0.080243638	0.101976761	0.079585905	0.067725996	0.071176509	0.206401156
NA		0.035001757	0.035918392	0.029985397	0.034265206	0.026461369	0.046487106
NA		0.610718587	0.712179327	0.542054574	0.664517931	0.639684801	0.778667538
	TRUE	0.059141828	0.043852148	0.062849489	0.041671659	0.046180296	0.076404314
NA		0.034235982	0.045744165	0.030369985	0.025819363	0.031158456	0.030519079
NA		0.869685504	0.904388783	0.801752110	0.887938212	0.901096078	0.888818471
NA		0.023089754	0.026837994	0.025330933	0.027120670	0.023003531	0.028341626
NA		0.024353359	0.027132146	0.021315335	0.023913641	0.029451982	0.026492881
NA		0.036749008	0.033522730	0.029359587	0.035486060	0.024109362	0.055019390
NA		0.014483524	0.011536762	0.010551098	0.010032547	0.013312671	0.012802490
NA		0.963613267	0.966579966	0.952226773	0.957965240	0.961809314	0.954928210
NA		0.618647873	0.614183780	0.571396900	0.610605678	0.716291071	0.663414379
NA		0.032480903	0.032888223	0.020974268	0.028770912	0.029297837	0.040823923
NA		0.057831620	0.063873598	0.061878565	0.051597735	0.053474787	0.072828258
NA		0.024023482	0.025101724	0.020114305	0.018548233	0.018538692	0.025414555
NA		0.039569603	0.042614990	0.035885327	0.031693608	0.029324192	0.048039254
NA		0.173532304	0.141671397	0.215198866	0.246705750	0.225491092	0.134956328
NA		0.017102079	0.020101582	0.017923529	0.017454925	0.024370007	0.024081998
NA		0.014984724	0.020541628	0.013978891	0.011571780	0.013178848	0.018099012
	TRUE	0.539656699	0.541734993	0.507221460	0.517156600	0.494018844	0.563362780
NA		0.049510104	0.039060733	0.040477298	0.028994125	0.039507852	0.043099742
	TRUE	0.167675816	0.174096240	0.156594203	0.176574037	0.164832034	0.171199513
NA		0.030193293	0.035563438	0.028881646	0.026300771	0.024655584	0.038313797

NA		0.03236471	0.02921227	0.02360668	0.02467276	0.024819866	0.03045843
NA		0.25148894	0.24038410	0.206354311	0.19714751	0.271049487	0.20827041
NA		0.16986117	0.22509969	0.13026026	0.13445821	0.135563547	0.15138622
NA		0.09565444	0.10653250	0.05237186	0.04914653	0.066815002	0.06841419
NA		0.03143634	0.04973770	0.04273165	0.04281668	0.029901001	0.07693589
NA		0.07230607	0.06819575	0.057745627	0.06285363	0.063312357	0.08381042
NA		0.01688538	0.01316512	0.01690308	0.01709535	0.012088471	0.01719751
NA		0.024750396	0.01475310	0.02337569	0.02499656	0.024113456	0.02067094
NA		0.42737258	0.50703663	0.37196107	0.32661245	0.481227271	0.37185100
	TRUE	0.88076399	0.91531381	0.90142888	0.89287955	0.896683683	0.88445575
NA		0.03716162	0.04846558	0.02753189	0.03386392	0.036151116	0.03861560
NA		0.07853111	0.06290342	0.07944165	0.06408358	0.060349228	0.05979432
NA		0.86427803	0.83039653	0.69451093	0.78008774	0.718879176	0.73815673
NA		0.35967319	0.37731472	0.36447687	0.39199216	0.394613531	0.40461589
NA		0.43938723	0.50663578	0.40969345	0.26846771	0.469826744	0.42287125
	TRUE	0.85707125	0.88374229	0.85906394	0.87233145	0.878181845	0.82246707
NA		0.02622050	0.02060608	0.02206464	0.02332114	0.019182358	0.02841965
NA		0.20192857	0.19918738	0.16132804	0.13076022	0.099293309	0.12783436
NA		0.04746399	0.04486760	0.03630166	0.03737392	0.037360943	0.04571618
NA		0.23929255	0.30566769	0.21917634	0.19690747	0.162046207	0.20791341
NA		0.02492088	0.01728051	0.01690892	0.01480129	0.018284523	0.02293554
NA		0.62696035	0.68116949	0.57516205	0.59799667	0.698841615	0.66296018
NA		0.04063513	0.04700294	0.04672158	0.02897311	0.035414365	0.04734165
NA		0.04604254	0.04921287	0.06766939	0.04123587	0.047509283	0.09001198
NA		0.03157409	0.03149784	0.030965964	0.02629355	0.029272107	0.052620058
NA		0.21241495	0.19444345	0.13971446	0.17632822	0.205383546	0.16133932
NA		0.02981011	0.02855755	0.02559624	0.02959366	0.024691393	0.046529035
NA		0.03112968	0.02513783	0.02225124	0.02435929	0.017923148	0.02755683
NA		0.03564679	0.03622785	0.03265245	0.03247517	0.027139490	0.04483957
NA		0.96227418	0.95855431	0.95068874	0.95996549	0.964884754	0.92641821
NA		0.02286391	0.02585570	0.01969038	0.02555200	0.020095400	0.02715652
NA		0.04068559	0.05256499	0.03394543	0.04128553	0.040508057	0.053328016
NA		0.21953054	0.24009567	0.19553766	0.23475678	0.260998142	0.24565717
NA		0.11274292	0.09585374	0.11816891	0.10164120	0.109551344	0.09040429
NA		0.02970022	0.032977174	0.02845824	0.03063849	0.026334212	0.03917256
NA		0.02619597	0.02526881	0.02342983	0.02389236	0.023419591	0.025318544
NA		0.18206802	0.15611017	0.13043191	0.13157876	0.118273197	0.13197422
NA		0.22324624	0.31681724	0.18237279	0.23990974	0.235111259	0.25803887
NA		0.020065874	0.01738074	0.02072281	0.02080896	0.017380030	0.02713076
	TRUE	0.903541115	0.924973702	0.90731926	0.91896762	0.951015063	0.88619659
	TRUE	0.38221940	0.428502188	0.36948617	0.45238296	0.415655962	0.49486742
NA		0.93274500	0.93603552	0.900108133	0.93105930	0.910764888	0.87632317
NA		0.01727342	0.01391849	0.010994097	0.01431223	0.016017485	0.022407246
NA		0.25107295	0.30541886	0.23762440	0.19755555	0.179322932	0.22117771

NA		0.018304435	0.016520328	0.013352125	0.015000694	0.017988979	0.016295207
	TRUE	0.016965793	0.019592555	0.018871673	0.018398569	0.018150995	0.019773607
NA		0.054712954	0.049075963	0.052636464	0.047271951	0.042795585	0.066227993
NA		0.035858695	0.022692256	0.025958288	0.033845151	0.024515830	0.026896466
NA		0.058357848	0.055637026	0.060148909	0.045391663	0.049881211	0.069511242
NA		0.019772819	0.021270358	0.016659074	0.022040518	0.022015059	0.023913680
NA		0.815816189	0.809937129	0.797732670	0.783463215	0.818565900	0.823857531
NA		0.057474765	0.046951465	0.037394808	0.041958423	0.041018566	0.040969908
NA		0.017911298	0.020345301	0.017917270	0.013192449	0.019555483	0.019103621
NA		0.602030004	0.619847922	0.566574629	0.596337217	0.604285997	0.599897794
NA		0.457685686	0.435632140	0.396463169	0.416605420	0.441899028	0.402029028
NA		0.765048389	0.807176149	0.735642947	0.748037105	0.773268178	0.712486147
NA		0.601097238	0.651967735	0.546044251	0.468801142	0.579597353	0.528179925
NA		0.416011795	0.484451483	0.423459707	0.412852525	0.438922645	0.456323115
NA		0.025040658	0.029851884	0.020781050	0.029416307	0.022180097	0.026840990
NA		0.023473567	0.019838804	0.018767083	0.022253153	0.019273375	0.028472985
NA		0.848908713	0.909942363	0.823111938	0.804943232	0.824383910	0.861103530
NA		0.024130841	0.024903987	0.029303339	0.020944935	0.020122194	0.026966339
NA		0.019945677	0.022621156	0.020148097	0.022568333	0.024440036	0.025089013
NA		0.053956770	0.034766948	0.048659913	0.055113802	0.043528723	0.037082531
NA		0.079457513	0.075577009	0.052325973	0.060247098	0.058324728	0.079084275
NA		0.680465364	0.766180304	0.706524834	0.690918287	0.731161371	0.712058621
NA		0.029555776	0.023894497	0.024919466	0.028349336	0.030769380	0.038196517
NA		0.026962765	0.021107849	0.035245657	0.037984424	0.033616728	0.049337697
NA		0.024754072	0.018519419	0.016713350	0.027764803	0.030589860	0.033120731
NA		0.426847301	0.459306671	0.389028541	0.388202704	0.511671530	0.408081504
	TRUE	0.072077994	0.083767789	0.071283014	0.064422270	0.063326458	0.066922174
NA		0.864983049	0.854042667	0.893163126	0.867837921	0.896067056	0.888202963
NA		0.039141780	0.032905963	0.040666714	0.032105903	0.036217082	0.062046249
NA		0.870893707	0.874641820	0.822428693	0.847355803	0.866339030	0.809338364
NA		0.056825757	0.045283577	0.059214798	0.048574300	0.062590035	0.054150925
NA		0.036612094	0.026716236	0.019476454	0.038914790	0.021677217	0.035321196
	TRUE	0.023011027	0.025951013	0.020802137	0.020276329	0.017174066	0.025417947
NA		0.201609405	0.205814905	0.197049221	0.193265624	0.193208796	0.207243853
NA		0.431136023	0.446829547	0.393619323	0.405047821	0.405221969	0.414672708
NA		0.047833638	0.038562730	0.033003924	0.058439233	0.035164540	0.038031571
NA		0.159226409	0.233949603	0.153071067	0.168960164	0.160522379	0.170331156
NA		0.285347563	0.218915640	0.272893734	0.192664168	0.317841886	0.266428008
NA		0.041963661	0.034345933	0.024694720	0.026269183	0.028518147	0.027149914
NA		0.409907526	0.397996162	0.379669569	0.340867382	0.357878037	0.320864847
NA		0.106235407	0.117247582	0.091404389	0.135729942	0.105271095	0.146817081
NA		0.045926715	0.051141218	0.037211684	0.038226734	0.053114356	0.051332646
	TRUE	0.629389586	0.697331604	0.639242537	0.725949643	0.776455735	0.662931163
NA		0.831921873	0.866703024	0.863592430	0.847028527	0.893517855	0.842801358

NA		0.311792570	0.268665790	0.361224206	0.295739880	0.300023719	0.404251158
NA		0.439401774	0.467902672	0.371820906	0.455286292	0.500233280	0.449890717
NA		0.057463307	0.037646438	0.032552665	0.037259072	0.040994721	0.039528274
NA		0.076507769	0.073867634	0.053140238	0.054437809	0.058570617	0.073650508
NA		0.235385169	0.260103400	0.191208779	0.209818336	0.196389231	0.209132087
NA		0.971795600	0.976422957	0.966422334	0.977110936	0.979510473	0.956750136
NA		0.785584180	0.775439146	0.786690611	0.771840152	0.792274492	0.788448231
NA		0.586610423	0.628802913	0.538822174	0.555972553	0.559214103	0.587507094
NA		0.050769083	0.057563514	0.065729647	0.055468581	0.035690052	0.164674980
NA		0.053136691	0.045699360	0.043193191	0.052827254	0.047895534	0.044196059
NA		0.084685600	0.128424872	0.098527654	0.117998291	0.098678653	0.159224947
NA		0.032592516	0.026499320	0.029850597	0.024208791	0.024878340	0.031469951
NA		0.040204411	0.038948851	0.033709598	0.033409529	0.028921376	0.060782992
NA		0.113403073	0.109862142	0.099306089	0.100755228	0.154356171	0.132843260
NA		0.028944109	0.036649484	0.020527152	0.018416480	0.023921608	0.042475852
NA		0.048301308	0.063105695	0.031744322	0.048743251	0.037979928	0.065717503
NA		0.023175015	0.021166559	0.015200927	0.017609442	0.015806321	0.023784749
NA		0.090456704	0.093624651	0.071714195	0.079558149	0.068964641	0.098293154
NA		0.241920649	0.211966033	0.093738603	0.087412848	0.070811968	0.196160899
NA		0.434535952	0.401172916	0.421777060	0.337604517	0.378187867	0.366938800
NA		0.040691707	0.040379065	0.033843148	0.039967260	0.051487514	0.043800966
	TRUE	0.369582336	0.382280362	0.328846140	0.323740400	0.379119734	0.324104621
NA		0.033059847	0.027811934	0.021093953	0.021798934	0.024020669	0.022420317
NA		0.053384090	0.073096393	0.051157777	0.055182336	0.047179872	0.063888247
NA		0.644439052	0.637340048	0.593766663	0.584981889	0.646806029	0.578976117
NA		0.088703399	0.127840138	0.067784508	0.075145181	0.054871675	0.090319158
	TRUE	0.574876647	0.614012180	0.519765388	0.592493617	0.685579341	0.613172732
NA		0.601300501	0.666259253	0.604380235	0.570652209	0.529210321	0.654116174
NA		0.699665845	0.735074656	0.658912859	0.659170151	0.664849811	0.734227651
NA		0.032632393	0.032168612	0.033337348	0.034285954	0.033361418	0.048601157
	TRUE	0.038583632	0.054344265	0.041292829	0.030892241	0.037522378	0.053462946
NA		0.035719304	0.043390183	0.031471532	0.037182627	0.041935869	0.039239387
NA		0.020516509	0.020246563	0.017635665	0.020723046	0.013889578	0.022626272
NA		0.048255661	0.047020491	0.055413355	0.045964870	0.049042717	0.055911796
NA		0.018811974	0.018776251	0.017742097	0.018874950	0.020243836	0.020182650
NA		0.043100349	0.053568523	0.052075039	0.046316059	0.040116577	0.079428633
NA		0.049701384	0.040081922	0.035032240	0.037510015	0.033474670	0.045241837
NA		0.023586105	0.024279664	0.020895129	0.016823852	0.023892128	0.027172182
NA		0.054516438	0.055568925	0.045805265	0.055020921	0.063343246	0.071692540
NA		0.029056955	0.017758749	0.018056637	0.021206193	0.028165731	0.029413353
	TRUE	0.046893934	0.047247013	0.038972002	0.041042671	0.042328430	0.041653926
NA		0.087138386	0.051244501	0.064426924	0.054069984	0.069827270	0.040527661
NA		0.024262507	0.020995557	0.022356737	0.021746751	0.027563873	0.019048153
NA		0.856151255	0.893143501	0.877992001	0.907053295	0.922925785	0.866386349

NA		0.052813675	0.050217845	0.048125411	0.040553633	0.043761653	0.055217962
NA		0.562002160	0.592288408	0.515801895	0.517667830	0.495212858	0.495876460
NA		0.049977805	0.044493900	0.037655772	0.044025275	0.049581737	0.054231028
NA		0.049302220	0.052924822	0.037576257	0.046252715	0.033551107	0.051549151
NA		0.620865213	0.643546181	0.513149435	0.534650787	0.507968364	0.502236186
NA		0.143477747	0.136194672	0.103135831	0.070088561	0.039724777	0.088630404
NA		0.672983711	0.700742697	0.637548547	0.645988612	0.671722004	0.665547750
NA		0.018473923	0.031453832	0.021230125	0.020000474	0.024667909	0.033166320
NA		0.355069961	0.376141194	0.293160833	0.247568656	0.226353199	0.246167259
NA		0.046333639	0.044863698	0.038276574	0.043988484	0.035663611	0.042495044
NA		0.155120129	0.136324163	0.137821904	0.141091540	0.145964755	0.129054601
NA		0.050713772	0.062283266	0.077428918	0.058269315	0.053091725	0.082453475
NA		0.343356081	0.469082233	0.324645378	0.343813574	0.351363938	0.464433931
NA		0.780872201	0.879518480	0.818780791	0.853007660	0.820837909	0.834709687
	TRUE	0.031125041	0.042463431	0.026086821	0.023465650	0.037004555	0.028889646
	TRUE	0.069778309	0.076007164	0.056604851	0.052839590	0.059183541	0.082251910
NA		0.081571619	0.085144709	0.077510304	0.079615559	0.097363224	0.097263289
NA		0.870559134	0.892957336	0.900528071	0.885732834	0.887524488	0.855574981
NA		0.387588892	0.422258013	0.346375025	0.339965275	0.321665017	0.286608311
NA		0.018530577	0.016646287	0.018402099	0.018559111	0.020235227	0.025547506
NA		0.083681973	0.149224178	0.101786993	0.086635336	0.120941307	0.165654891
NA		0.055149062	0.052570903	0.041905707	0.041370058	0.037050788	0.056721516
NA		0.032408028	0.034024455	0.020814767	0.028601159	0.030300812	0.032220885
NA		0.028798812	0.034589779	0.047255190	0.027093685	0.038397471	0.051501674
NA		0.060723384	0.089359590	0.053813468	0.053721922	0.066644030	0.070440892
NA		0.028997416	0.026255730	0.023250242	0.028094413	0.029088930	0.026960356
	TRUE	0.052350408	0.065299160	0.035766811	0.042489569	0.050717913	0.050171605
NA		0.496083120	0.588421189	0.396134799	0.407352341	0.446195132	0.410624412
NA		0.035610427	0.035960056	0.033696303	0.029791353	0.038884542	0.047942253
NA		0.036103001	0.023052859	0.027342837	0.035801808	0.023701132	0.031296909
NA		0.073456513	0.252347298	0.112085735	0.103516830	0.143371492	0.157207658
NA		0.059495793	0.050360117	0.037084255	0.048420259	0.044940467	0.041424845
NA		0.523997230	0.576694879	0.628434580	0.533782593	0.481668633	0.521974041
NA		0.026770030	0.025819780	0.030164169	0.023039149	0.023502008	0.038044593
NA		0.038750527	0.026518241	0.022339899	0.033650696	0.025579314	0.034216034
NA		0.801179179	0.852797530	0.829629712	0.753283555	0.660064219	0.662444865
NA		0.423320623	0.436654471	0.386402840	0.365245378	0.386248598	0.406869613
NA		0.023894506	0.030154757	0.023316653	0.024920318	0.020415448	0.033801639
NA		0.028344707	0.022379595	0.024062932	0.024938889	0.032338772	0.033384247
NA		0.678614696	0.715843961	0.701033388	0.697926744	0.836355248	0.738243991
NA		0.061848706	0.063948313	0.057270348	0.047213145	0.060846064	0.064350032
NA		0.032731202	0.052265870	0.045477446	0.032931136	0.033405005	0.058065503
NA		0.103472065	0.112600157	0.082964249	0.054066432	0.030829117	0.050914058
NA		0.023261993	0.030149503	0.026894113	0.033661074	0.023110794	0.035597605

NA		0.034234040	0.037662676	0.028356423	0.035728399	0.037120083	0.035344413
NA		0.366234282	0.482946430	0.443410681	0.382589296	0.509002412	0.479645401
NA		0.022137882	0.021834216	0.017660376	0.025721376	0.023482281	0.021669942
	TRUE	0.033322946	0.039961905	0.030146012	0.033509850	0.023741532	0.042747909
	TRUE	0.178122893	0.178753711	0.137993922	0.178165043	0.168552860	0.186051944
NA		0.333872033	0.051755549	0.382704446	0.023835555	0.033558324	0.101231725
	TRUE	0.148720100	0.175491894	0.145518944	0.165847843	0.230267537	0.202524869
NA		0.015834286	0.017582502	0.023958655	0.021149837	0.015988195	0.022755211
NA		0.777354544	0.742230392	0.722039610	0.717967518	0.778059179	0.806578822
NA		0.019668531	0.015034109	0.020425395	0.029260451	0.019457969	0.026177487
	TRUE	0.167534362	0.171677660	0.161874733	0.173285232	0.155111944	0.221012929
NA		0.028185607	0.015843068	0.017335576	0.019692575	0.022223398	0.022618538
NA		0.023234878	0.026962962	0.023680115	0.027536765	0.029122060	0.030637198
NA		0.039061494	0.034734335	0.056521120	0.039696641	0.035019690	0.053631928
NA		0.121995229	0.162273520	0.124593712	0.064824817	0.132216572	0.191072002
NA		0.024101242	0.022729887	0.019109831	0.020412558	0.019599679	0.027420465
NA		0.848014924	0.878946143	0.871910163	0.870124723	0.852836869	0.820547489
	TRUE	0.066528889	0.083200621	0.083587578	0.067433294	0.046114886	0.149111290
NA		0.125992929	0.102325313	0.089659240	0.103884112	0.153707741	0.125538399
	TRUE	0.957901696	0.954488706	0.921677909	0.960980608	0.961692569	0.944841493
NA		0.021489816	0.020259388	0.013451370	0.021799331	0.025460237	0.026821609
NA		0.066047597	0.062630006	0.081669089	0.069070203	0.064949994	0.123910500
NA		0.045590559	0.047352665	0.054664483	0.045857822	0.038664211	0.054966068
NA		0.057903377	0.049334324	0.048912947	0.053670317	0.051970940	0.061790478
NA		0.030792046	0.041047695	0.031170854	0.027048420	0.031702660	0.040058012
NA		0.036011426	0.025928055	0.029309678	0.037565907	0.039031919	0.041277459
NA		0.028653179	0.034365988	0.033382380	0.039604032	0.039941751	0.034731591
	TRUE	0.311830820	0.314948732	0.230032813	0.245969924	0.149935000	0.221369969
NA		0.569849576	0.582205930	0.573188346	0.585865971	0.568923881	0.556638873
NA		0.024884152	0.019006593	0.021284767	0.023897638	0.021668089	0.028686449
NA		0.071962430	0.102312258	0.061188983	0.083147271	0.062827941	0.089500824
NA		0.034352274	0.030961948	0.023025013	0.027321924	0.032253565	0.022437068
NA		0.959183607	0.962677174	0.942059153	0.956002106	0.967840138	0.907683744
NA		0.047823286	0.040383150	0.036197126	0.038539834	0.033933313	0.039593779
NA		0.040744696	0.053005252	0.035698255	0.030072521	0.031221372	0.047966364
NA		0.042304119	0.039837773	0.039396222	0.028422059	0.028867713	0.060017085
	TRUE	0.134872915	0.131335651	0.118909620	0.119869749	0.111821988	0.136972511
	TRUE	0.738833424	0.762882953	0.780975955	0.753309909	0.773481159	0.742290323
NA		0.831927832	0.830037797	0.772692131	0.790216262	0.811909894	0.786640123
NA		0.082558486	0.088250067	0.054465323	0.066546801	0.088409753	0.066277728
NA		0.706744274	0.714066996	0.748554154	0.602682401	0.712012290	0.755624594
NA		0.307170736	0.353378811	0.239915542	0.313819966	0.313887815	0.285640674
NA		0.046103418	0.055028535	0.044924431	0.044120985	0.043743794	0.053695113
NA		0.103812861	0.106299693	0.104022869	0.089032931	0.069162125	0.163846303

NA	0.037774609	0.038960562	0.037648507	0.032031808	0.034901605	0.054026395
NA	0.045405414	0.034123126	0.048280633	0.036885229	0.039025730	0.066229631
NA	0.049420202	0.046781197	0.045590166	0.040415198	0.041977405	0.069532716
NA	0.279431327	0.314330887	0.228604802	0.263409187	0.275197125	0.274965387
NA	0.027869926	0.039942295	0.029882653	0.027871957	0.038784653	0.039803854
TRUE	0.026962439	0.019792191	0.018503888	0.019591962	0.016285949	0.022469222
NA	0.485300649	0.500231584	0.424959305	0.495213856	0.503771182	0.526795650
NA	0.646964121	0.742805582	0.755722449	0.731370591	0.775981211	0.815161654
NA	0.058311196	0.050235016	0.049118754	0.054500260	0.037964103	0.053275570
NA	0.038426130	0.037976340	0.034505183	0.041824800	0.038412174	0.052797708
NA	0.841549204	0.834665121	0.799349937	0.779566295	0.794865223	0.736289393
NA	0.048498324	0.036689557	0.028535794	0.030693061	0.035454989	0.047616540
TRUE	0.047592579	0.025660039	0.037331348	0.035901186	0.039227684	0.043884551
NA	0.056380196	0.053922281	0.066409622	0.053440631	0.048900746	0.076941478
NA	0.737707235	0.774137633	0.760363511	0.782504210	0.766052958	0.729438887
NA	0.078844371	0.107252908	0.063422063	0.042903898	0.065733257	0.120899965
NA	0.562859314	0.572200211	0.511737682	0.461213062	0.531749175	0.538982180
NA	0.106836105	0.076119329	0.098332723	0.104559274	0.078338877	0.140675177
TRUE	0.082954659	0.122689074	0.053209874	0.075452854	0.045352545	0.087104780
NA	0.310520744	0.318699970	0.284927329	0.340360709	0.255246454	0.355416464
NA	0.024477201	0.027743412	0.019368289	0.029155778	0.019119007	0.023189932
NA	0.037156757	0.048759462	0.058260671	0.057850840	0.065796566	0.056671780
NA	0.579149843	0.658055659	0.631731643	0.473926693	0.596793601	0.630074128
NA	0.850943324	0.912837546	0.881757201	0.859969166	0.872425931	0.856506842
NA	0.723815200	0.759280289	0.702964625	0.684536631	0.637439098	0.667796787
NA	0.126206027	0.116409448	0.108523669	0.106428099	0.069669307	0.086007843
NA	0.492285170	0.507969031	0.414857177	0.425628433	0.451572528	0.455059755
NA	0.424358535	0.404566775	0.314251662	0.348229678	0.376238600	0.446506966
NA	0.026712250	0.026634733	0.024017644	0.031118650	0.025601526	0.038778662
NA	0.490616655	0.552891985	0.516850510	0.480550780	0.450644185	0.507222504
NA	0.031210600	0.034738135	0.014758931	0.018722597	0.024636367	0.033843011
NA	0.067334020	0.074250502	0.047186377	0.084507854	0.059949188	0.077264849
NA	0.045286758	0.055766642	0.046597425	0.035724801	0.037813760	0.047753406
NA	0.047978274	0.040129320	0.032310633	0.040168675	0.038347226	0.046884928
NA	0.068526383	0.087038179	0.056342064	0.070346979	0.062920113	0.063433327
NA	0.157358520	0.185626071	0.148935868	0.129596982	0.203628487	0.175084543
NA	0.024818129	0.028876129	0.028812556	0.039889632	0.029642036	0.027314314
NA	0.062275284	0.053389395	0.051045998	0.044654179	0.048368496	0.048457869
NA	0.631426479	0.635955515	0.601103005	0.562201072	0.546544836	0.506143818
NA	0.177300362	0.295725617	0.208442919	0.233914063	0.256355400	0.295812637
NA	0.039358155	0.035099057	0.033265544	0.023069564	0.031294626	0.037263859
NA	0.334008151	0.376112100	0.291931059	0.280108777	0.308875239	0.298084234
NA	0.028001413	0.026042270	0.020265974	0.027007786	0.036880872	0.037751599
NA	0.036339012	0.032639966	0.027023977	0.023751809	0.020362573	0.032630182

NA		0.033964191	0.029192152	0.028947048	0.035126760	0.033417437	0.034952552
NA		0.031751511	0.045609162	0.032335916	0.034677772	0.036052711	0.041034026
NA		0.493142926	0.624725224	0.431692696	0.451806260	0.481866124	0.524479279
NA		0.848384721	0.856893410	0.824925570	0.820734734	0.839813907	0.802812432
NA		0.438677349	0.451610361	0.411537026	0.409729908	0.418650792	0.465992674
NA		0.027366758	0.044432910	0.020810074	0.033764345	0.025098828	0.054009175
NA		0.051024553	0.040808657	0.042549672	0.040722019	0.053919362	0.043544651
	TRUE	0.489241626	0.526184585	0.509988807	0.523485953	0.540165581	0.571850051
NA		0.050046035	0.050173604	0.054555565	0.045766533	0.045335120	0.074671675
NA		0.091001801	0.074761328	0.117639387	0.099513845	0.159914826	0.108414947
NA		0.293689313	0.474003376	0.408697423	0.408716480	0.453520237	0.465297320
NA		0.055909383	0.054659559	0.061612750	0.052509620	0.052234645	0.073799743
NA		0.083099079	0.080915426	0.072768480	0.067383200	0.032137618	0.066136207
NA		0.039899408	0.038390126	0.041955709	0.034453356	0.028208153	0.045202912
NA		0.098088529	0.086041560	0.058540749	0.082934476	0.052472188	0.060715079
NA		0.042316645	0.026081450	0.021894229	0.025870044	0.026901867	0.026840930
NA		0.263931218	0.312629237	0.211189913	0.208280845	0.228717348	0.242254051
NA		0.341731786	0.364725107	0.292903936	0.260188273	0.213035586	0.208962158
	TRUE	0.107105794	0.149158561	0.106979147	0.124387086	0.128547661	0.143897945
NA		0.020617024	0.016841252	0.026550328	0.021417036	0.020598259	0.028182761
NA		0.048762802	0.052543370	0.045563487	0.048590330	0.037698879	0.041240375
NA		0.845548331	0.852939983	0.824396535	0.857071530	0.853797858	0.841543928
NA		0.024931104	0.022452835	0.034866815	0.027598348	0.021857583	0.034458878
NA		0.865488430	0.892127150	0.867061046	0.881785347	0.862348476	0.840001929
	TRUE	0.151283908	0.147276763	0.132167402	0.153603192	0.123156758	0.142137212
NA		0.845647258	0.895762970	0.759779353	0.891441950	0.918725758	0.886951868
NA		0.059101890	0.040250204	0.042161972	0.046603519	0.039458394	0.052223836
NA		0.619169013	0.620317940	0.549157067	0.529257196	0.674483593	0.532407308
NA		0.017181281	0.013728276	0.015201711	0.013901363	0.015415904	0.017853571
NA		0.023772999	0.014451721	0.020330850	0.019963173	0.017236682	0.026342583
NA		0.621411380	0.742303257	0.655368849	0.730547173	0.839400528	0.738740554
NA		0.840651712	0.853285236	0.820408833	0.847435304	0.824029176	0.798973977
NA		0.031990112	0.034900999	0.029979541	0.033479458	0.032982116	0.047841443
NA		0.874942655	0.892234621	0.861950404	0.855929209	0.868942503	0.855764066
NA		0.035002988	0.034246967	0.040984652	0.039490928	0.035310006	0.054652607
NA		0.026312207	0.030021620	0.027763283	0.029461808	0.024350667	0.033369096
NA		0.035632929	0.019999065	0.019510340	0.024748361	0.024293209	0.024616704
NA		0.024169183	0.016257979	0.018332506	0.021460595	0.016429888	0.026804705
NA		0.028192403	0.037469744	0.033118822	0.027945250	0.025689754	0.041817464
NA		0.044432718	0.041259958	0.032579566	0.037644225	0.039142969	0.044614985
NA		0.046543197	0.037738431	0.031308994	0.038839722	0.031342663	0.049925206
NA		0.492304346	0.514954693	0.454325189	0.474309417	0.479328017	0.461674446
NA		0.858925426	0.893278143	0.867046895	0.879523308	0.877595724	0.883784643
NA		0.779233799	0.761537538	0.728151039	0.664409077	0.738424682	0.698741826

NA		0.201532545	0.274946483	0.211721461	0.154225572	0.213592974	0.232172490
NA		0.157764811	0.136625551	0.136867769	0.101619786	0.092122652	0.081314419
NA		0.149052837	0.112792056	0.111699540	0.084610052	0.071063022	0.081104839
	TRUE	0.678911622	0.713263045	0.661853178	0.716462847	0.760706811	0.706078994
NA		0.752764879	0.829817088	0.710105838	0.727756049	0.701998988	0.737693320
NA		0.215639240	0.174405085	0.190520691	0.242367370	0.294410516	0.185783634
NA		0.088189316	0.092168247	0.059359548	0.063385605	0.058752624	0.068088570
NA		0.593810283	0.635207096	0.589155659	0.581222708	0.545735250	0.605120001
NA		0.493786282	0.506398717	0.502737384	0.469421222	0.449851383	0.493370478
NA		0.200263051	0.221524090	0.192334176	0.246306991	0.249522200	0.247484498
NA		0.339225825	0.196680134	0.403732104	0.175999805	0.425547315	0.219565069
	TRUE	0.042774945	0.048239469	0.036880214	0.032179542	0.028556503	0.052652133
NA		0.964070754	0.955585084	0.925301094	0.966163643	0.951657929	0.958467385
NA		0.287361795	0.338465110	0.282159791	0.246365171	0.265568518	0.226900492
NA		0.018227038	0.022591813	0.019642475	0.025313483	0.024653621	0.029677052
	TRUE	0.505683130	0.584691644	0.481144852	0.463952827	0.562759416	0.423336336
NA		0.049545676	0.067162075	0.168774591	0.039750422	0.145853585	0.088340561
NA		0.027867873	0.026852651	0.040000263	0.028977098	0.037902578	0.052027945
NA		0.061551893	0.064270369	0.067849779	0.043146407	0.038161957	0.093949650
NA		0.524682174	0.638409569	0.666151954	0.660447816	0.655444383	0.643876551
NA		0.920502961	0.914222370	0.896049608	0.930068794	0.939484681	0.865544812
NA		0.064558506	0.097106609	0.051132823	0.067465380	0.071929867	0.075828665
NA		0.028483240	0.036729217	0.030040608	0.028828346	0.036584814	0.031915785
NA		0.055281307	0.054729098	0.045470122	0.041736050	0.045989234	0.055429310
NA		0.159657015	0.195022674	0.124143085	0.119992130	0.128273910	0.135577877
NA		0.682423271	0.710612977	0.658545369	0.562316097	0.645579847	0.616767036
NA		0.207282382	0.229645049	0.203368216	0.169367642	0.184041482	0.170213256
NA		0.579976574	0.636200601	0.555223807	0.592947907	0.578657180	0.578490458
	TRUE	0.095602498	0.080339574	0.070846076	0.066879391	0.066270535	0.050624172
NA		0.049740999	0.042956142	0.043641519	0.044296749	0.045934089	0.045469464
NA		0.033998898	0.037275502	0.036731804	0.037515844	0.039689800	0.035627532
NA		0.290606882	0.286115708	0.236210639	0.239383500	0.232162446	0.249318016
NA		0.809852276	0.779526850	0.855955653	0.797216756	0.835657821	0.800014486
	TRUE	0.095804709	0.114772148	0.093660628	0.101500073	0.093369022	0.095180978
NA		0.697097826	0.694244736	0.640544903	0.702676653	0.574102106	0.626346331
NA		0.027968080	0.020501608	0.023168285	0.022339365	0.018997640	0.025434018
NA		0.069407055	0.073499590	0.065292192	0.065953422	0.057920276	0.101437704
NA		0.058204979	0.054409135	0.050430091	0.049527058	0.021981517	0.045420094
NA		0.559766280	0.567214874	0.483572784	0.536500144	0.545439166	0.521059010
NA		0.024286725	0.019976666	0.014608160	0.028173846	0.017348077	0.023591458
	TRUE	0.054413993	0.069130412	0.056654818	0.038553852	0.059267550	0.072257755
NA		0.100612527	0.101152731	0.116144764	0.110203599	0.098947772	0.172322679
NA		0.657181283	0.707273041	0.626919116	0.546390541	0.592799084	0.516856179
NA		0.081268835	0.069713097	0.059925697	0.060787391	0.046302641	0.064441206

NA		0.64061916	0.68275847	0.64265087	0.51568442	0.671797737	0.60677007
	TRUE	0.43336543	0.40815378	0.38379077	0.48954854	0.580327458	0.55695119
NA		0.76017747	0.78645097	0.722801869	0.67526604	0.788995649	0.74346524
NA		0.04478482	0.03279589	0.03558235	0.03859445	0.038517007	0.03423127
NA		0.03504767	0.02368553	0.04422467	0.03882302	0.029736199	0.03465372
NA		0.69276164	0.67175622	0.710128599	0.65895792	0.721476993	0.67686697
NA		0.18234898	0.08413715	0.09358159	0.13628389	0.155315413	0.10853802
NA		0.28255273	0.31959821	0.26155959	0.303844548	0.346480888	0.32861283
NA		0.20376263	0.19648339	0.198074911	0.35448291	0.186103097	0.20177225
NA		0.05152756	0.05475536	0.04133865	0.04381405	0.051984053	0.05922732
NA		0.03734862	0.02533445	0.03788753	0.03552647	0.043182988	0.04043147
NA		0.02317627	0.02789633	0.02010291	0.02092612	0.021208870	0.02733293
NA		0.06426897	0.06433974	0.08744660	0.07451005	0.061926525	0.10759421
NA		0.02683086	0.03387588	0.02696793	0.02868449	0.022222034	0.04090776
NA		0.09922217	0.11255017	0.09179353	0.11887093	0.120209612	0.12727434
NA		0.14990433	0.13335774	0.10059970	0.07593878	0.040338379	0.09784356
	TRUE	0.02869773	0.02548221	0.02028822	0.032580705	0.021973088	0.02600998
NA		0.02483532	0.02337783	0.01549927	0.02151030	0.020639204	0.04054830
NA		0.84217133	0.83182319	0.77213257	0.79977938	0.780565179	0.75438588
NA		0.88484775	0.915981413	0.87346317	0.87754810	0.892347964	0.86811015
NA		0.06797597	0.06929457	0.04468458	0.05112084	0.063932086	0.05130593
NA		0.16876301	0.16812397	0.14256577	0.19240716	0.256654402	0.18292381
NA		0.03927830	0.03358609	0.03114989	0.032111263	0.043146431	0.03540072
NA		0.042381088	0.03943348	0.03736491	0.02998568	0.031190556	0.06213172
NA		0.05517436	0.06063657	0.05895219	0.05569052	0.063792395	0.07416846
NA		0.05395631	0.067510830	0.04970770	0.04285635	0.037035646	0.06400742
NA		0.02521040	0.02162521	0.02035213	0.01852541	0.016775058	0.02578214
	TRUE	0.06899321	0.04168970	0.04915631	0.06011788	0.055954015	0.041484160
NA		0.02701597	0.02613295	0.02939211	0.03082263	0.029966791	0.03450217
NA		0.03192350	0.01875513	0.02356276	0.03341895	0.029907207	0.03731065
NA		0.68368506	0.686566961	0.67037582	0.65804911	0.663674055	0.64652750
NA		0.03280064	0.03924605	0.03006291	0.040920012	0.035504194	0.04055308
NA		0.032808340	0.02976561	0.02551393	0.02531290	0.022754226	0.034121449
NA		0.05297540	0.07375100	0.08851376	0.10117678	0.088403949	0.12019586
NA		0.02194797	0.01838629	0.02416329	0.02061290	0.020507881	0.02211043
	TRUE	0.02544127	0.03090568	0.02226118	0.026833806	0.029728243	0.03705287
NA		0.11279514	0.09781752	0.09068110	0.09605355	0.081155699	0.11590432
NA		0.25676917	0.29224728	0.242755561	0.27888244	0.251079218	0.31474077
NA		0.04533760	0.02967596	0.03240637	0.027499180	0.026507166	0.04108950
NA		0.10210739	0.08305133	0.07961286	0.10316033	0.116850870	0.08454927
NA		0.05725640	0.02491432	0.02994187	0.03449536	0.022982676	0.04336387
NA		0.83691755	0.86904813	0.81448783	0.82309810	0.822533931	0.81844675
	TRUE	0.03620291	0.04560642	0.03477579	0.03221358	0.023063216	0.04629346
NA		0.43985838	0.49314272	0.53858518	0.43452282	0.120349250	0.44624298

NA		0.821299034	0.856988056	0.754468637	0.833657442	0.813675632	0.843519009
NA		0.829942425	0.858483832	0.779891175	0.801656604	0.835763743	0.772814643
NA		0.827590366	0.856623486	0.762563688	0.802618890	0.820939910	0.741797404
NA		0.062218132	0.062426959	0.051647921	0.053429653	0.055758762	0.057028110
NA		0.029926626	0.032088471	0.031361371	0.028165712	0.037780793	0.044330767
NA		0.362303418	0.361992133	0.308213695	0.376635247	0.338619037	0.347528115
NA		0.688966898	0.703238679	0.669584643	0.615556903	0.696596784	0.624909607
	TRUE	0.068373430	0.068484504	0.062047325	0.066737789	0.075242335	0.076785445
NA		0.023149000	0.020003766	0.020020780	0.018326777	0.021229340	0.024869915
NA		0.815990844	0.805797010	0.701427928	0.705387344	0.673232993	0.732964546
NA		0.633999661	0.613489061	0.550151026	0.510975381	0.511474532	0.566556264
	TRUE	0.025551906	0.031844564	0.024449000	0.034445335	0.032836446	0.037555843
NA		0.040733077	0.050120945	0.053010810	0.042909967	0.027742944	0.135606440
NA		0.049909648	0.049296566	0.049958170	0.051864875	0.052842677	0.060172776
NA		0.793818661	0.818345436	0.762399444	0.772049975	0.910890184	0.822370507
NA		0.026583187	0.027159597	0.017648715	0.020335926	0.015929970	0.021317713
NA		0.105886294	0.078147601	0.125784176	0.156201244	0.132083705	0.119295647
NA		0.863394989	0.895601809	0.879789921	0.846042064	0.863307065	0.825146939
NA		0.045231854	0.047206138	0.056839332	0.045774310	0.034803427	0.085658570
NA		0.789863926	0.807520797	0.770167527	0.814629162	0.789903292	0.734556511
NA		0.030810391	0.028463634	0.024748454	0.021311765	0.022921189	0.029625421
NA		0.052029684	0.064567684	0.064451920	0.050740558	0.045798622	0.081478665
	TRUE	0.077790590	0.082967596	0.089736641	0.085770397	0.063856584	0.137576148
NA		0.032949170	0.038723542	0.035312721	0.035991060	0.032382242	0.041695145
NA		0.020941912	0.016987035	0.018057053	0.015600137	0.021891513	0.021904112
NA		0.821285784	0.862979059	0.858669312	0.856526974	0.879567455	0.844265410
NA		0.705541756	0.799895716	0.792065150	0.742516338	0.783107651	0.756859229
NA		0.448543896	0.480806407	0.416574393	0.413807247	0.398783501	0.411245028
NA		0.081078366	0.060226257	0.063919591	0.067936441	0.053008862	0.058955870
NA		0.051398075	0.032446322	0.037287668	0.044919061	0.034190725	0.052519091
NA		0.167543495	0.150994775	0.131490473	0.137785066	0.121949529	0.092242589
NA		0.021228218	0.016352320	0.018707098	0.019891133	0.018570515	0.033491774
NA		0.539338770	0.562371489	0.450385873	0.438351126	0.483990376	0.413898112
NA		0.085893249	0.096201323	0.056115480	0.107075734	0.089271037	0.091289546
NA		0.767826161	0.893409258	0.703686216	0.856241585	0.908154335	0.786860076
NA		0.377488968	0.426045328	0.489265614	0.544564810	0.492183597	0.369096321
NA		0.032257201	0.044179800	0.028105039	0.030783647	0.037229164	0.037612605
NA		0.038076951	0.038461334	0.050499999	0.044582509	0.034101801	0.065206257
	TRUE	0.191079090	0.235610421	0.164440150	0.162767028	0.279183620	0.200615225
NA		0.551925404	0.616211169	0.495825425	0.504528542	0.590728361	0.617349756
NA		0.025550570	0.019221810	0.024485062	0.024811261	0.024224956	0.027929170
NA		0.034499399	0.034947399	0.038401929	0.032845499	0.039124322	0.037861896
NA		0.030746682	0.039846535	0.027300228	0.042971746	0.025803757	0.065961521
NA		0.031223586	0.031689248	0.025939419	0.027834119	0.027196836	0.039516620

NA	0.025742104	0.023516515	0.022554583	0.022176302	0.024043426	0.025992527
NA	0.026723334	0.035927173	0.031863641	0.029099478	0.026778303	0.039412729
NA	0.023182776	0.017230291	0.013172905	0.015932600	0.013661660	0.022870923
TRUE	0.106112236	0.060452865	0.085674455	0.073711876	0.076659657	0.092453513
NA	0.763612015	0.429581245	0.772503696	0.830377565	0.870202870	0.455386729
NA	0.037631948	0.035229736	0.033625072	0.027572480	0.023535419	0.039253873
NA	0.453313904	0.585376742	0.523205908	0.424153813	0.483835842	0.492132819
NA	0.071961792	0.071509304	0.071549720	0.078007192	0.098627469	0.104587285
NA	0.063953394	0.064681160	0.059425380	0.063096530	0.056165235	0.084952137
NA	0.425777997	0.529486198	0.439462911	0.393918014	0.461606075	0.442918288
NA	0.553380576	0.606787675	0.532833786	0.542933296	0.545964958	0.541982619
NA	0.856059125	0.874087509	0.792830742	0.864584347	0.866250165	0.841448218
NA	0.314652177	0.350696490	0.263575617	0.240206923	0.226157277	0.223961386
NA	0.450856608	0.499940996	0.334173521	0.398765712	0.549463423	0.447385366
NA	0.644710052	0.655934379	0.464809922	0.574146385	0.593807573	0.577102158
NA	0.024088909	0.020894201	0.020594097	0.028502321	0.019718533	0.022210375
NA	0.032533372	0.032401748	0.026610134	0.028094952	0.026236343	0.029145905
NA	0.054188278	0.038781477	0.041586949	0.067645701	0.053086590	0.064772955
NA	0.062102039	0.046022977	0.063872493	0.043423372	0.037582006	0.057830037
NA	0.805451105	0.752153182	0.758431189	0.692510845	0.727763669	0.699892405
NA	0.021377586	0.025196679	0.019312287	0.023014976	0.020827823	0.032883265
NA	0.858703853	0.861409110	0.878566690	0.843820138	0.858640814	0.828162799
NA	0.033998376	0.035360389	0.029044737	0.021590534	0.023184831	0.035889804
NA	0.027577233	0.024951159	0.025854261	0.023316194	0.021092529	0.030287985
NA	0.045802143	0.043032077	0.034160997	0.030752216	0.031809604	0.039261334
NA	0.040741802	0.041925908	0.035299128	0.069823073	0.119420560	0.065996519
NA	0.031830349	0.016941997	0.020537871	0.023931558	0.023193667	0.026787164
NA	0.064245638	0.053952851	0.048283489	0.071955613	0.075512479	0.055662123
NA	0.055810869	0.050363702	0.037079935	0.032489631	0.027876263	0.064413634
NA	0.474300583	0.496362772	0.425220359	0.454792330	0.603949739	0.542164774
NA	0.898344593	0.896601002	0.869679756	0.878194258	0.888033446	0.813246162
NA	0.501693370	0.586360385	0.553165693	0.566107914	0.492301693	0.502818159
NA	0.460029051	0.457671388	0.388453249	0.446098968	0.476103553	0.443355916
NA	0.785084304	0.755285591	0.615953385	0.661664243	0.799803794	0.839775099
NA	0.808066096	0.907477081	0.832873048	0.783153361	0.852128731	0.841958856
NA	0.897462396	0.933810578	0.899928970	0.896311338	0.909206880	0.894999930
NA	0.385182154	0.419777554	0.350930973	0.389465465	0.470780007	0.457656026
NA	0.040901099	0.056428330	0.047318027	0.038755991	0.039469573	0.100049484
TRUE	0.363033887	0.352869676	0.295959158	0.358887061	0.527525465	0.422519970
NA	0.407831016	0.508962129	0.337627164	0.362201006	0.342016442	0.347161046
NA	0.226805505	0.278953820	0.236538172	0.327965494	0.289760191	0.252474977
NA	0.127748862	0.144358036	0.105504819	0.124291987	0.130849821	0.141804743
NA	0.835725427	0.860443868	0.843767478	0.824991717	0.814409166	0.803680167
NA	0.846297651	0.838025940	0.816516045	0.799974869	0.834601587	0.796373368

NA		0.018745666	0.022246927	0.018368848	0.016314377	0.015431128	0.019348673
NA		0.042741241	0.040438742	0.031575168	0.038444308	0.029940756	0.050191080
NA		0.251928546	0.248685853	0.221051623	0.201146720	0.209207893	0.176202413
NA		0.077549044	0.067827697	0.062996096	0.056987202	0.048989832	0.120551547
NA		0.050790499	0.040916177	0.037359158	0.049975928	0.041079523	0.048120508
NA		0.339072380	0.351820566	0.310994335	0.308034745	0.332943416	0.311302209
NA		0.066875959	0.040876702	0.060446865	0.052265024	0.045952385	0.076520397
NA		0.023831777	0.021750094	0.020588005	0.021154952	0.020033007	0.029282846
NA		0.034984080	0.024797527	0.025662633	0.032143870	0.039917367	0.035871885
NA		0.051343901	0.075858229	0.057300395	0.049774084	0.041515970	0.081953775
NA		0.017582181	0.016650925	0.017996433	0.017239523	0.018148152	0.021600185
NA		0.031530572	0.031592313	0.030991431	0.029239497	0.045565407	0.031522259
NA		0.080811351	0.094524718	0.064236886	0.059622080	0.054401047	0.110851479
NA		0.079808757	0.107491180	0.080367148	0.077515344	0.116081943	0.071430815
NA		0.033748754	0.037629129	0.351204071	0.036864435	0.021299837	0.048788677
NA		0.033897552	0.027303519	0.027664901	0.024143608	0.032447800	0.034817496
NA		0.043525657	0.054963723	0.032629938	0.050129412	0.042132752	0.042878211
	TRUE	0.624390833	0.680192749	0.619267738	0.647971823	0.764023129	0.675282284
NA		0.030062745	0.034017454	0.023759091	0.020989366	0.028948008	0.031629739
NA		0.017662770	0.021435333	0.016489965	0.020220192	0.023494926	0.027309765
NA		0.064386651	0.069163460	0.070463510	0.056115572	0.064909845	0.052573230
NA		0.816038059	0.767299146	0.667542868	0.845174837	0.882391566	0.802830716
NA		0.952134545	0.964630721	0.950773834	0.947843617	0.961044362	0.941991311
	TRUE	0.654278938	0.688767786	0.604136657	0.611636652	0.665031626	0.663831296
NA		0.059963082	0.051337358	0.047108488	0.049118678	0.052721998	0.072148984
NA		0.020894091	0.027838059	0.023332382	0.025359802	0.022899883	0.035411796
NA		0.900339247	0.887477826	0.907962734	0.792468115	0.889678801	0.733912465
NA		0.045283075	0.048464922	0.037373672	0.051486660	0.045888375	0.040986437
NA		0.336804328	0.510699747	0.573853689	0.571982219	0.504583189	0.531821163
NA		0.706967042	0.743275899	0.633492097	0.562085823	0.603220867	0.614572071
NA		0.838562392	0.837535504	0.789655413	0.817766463	0.856641008	0.795585682
NA		0.024421466	0.025014538	0.019480746	0.018922156	0.015888227	0.020505250
NA		0.731778571	0.779750006	0.663187088	0.627586858	0.739666046	0.642737158
NA		0.018164625	0.020576547	0.017455969	0.025762034	0.019688195	0.027724498
	TRUE	0.686715045	0.675267055	0.704916722	0.655500757	0.681227279	0.604862173
NA		0.022172465	0.018330992	0.019430171	0.020587634	0.022428294	0.023368453
NA		0.831722359	0.854713347	0.822017858	0.803103569	0.781775880	0.833898092
NA		0.039967807	0.036857817	0.036456518	0.035716595	0.045802119	0.038166894
	TRUE	0.080612743	0.100091538	0.060744130	0.054629389	0.050190566	0.052792332
NA		0.739621201	0.793013576	0.734309161	0.717485612	0.705539426	0.804748313
NA		0.741289530	0.795422726	0.703670863	0.694979384	0.733257295	0.727748324
NA		0.796109268	0.835940777	0.764126637	0.785217330	0.799589172	0.702779212
NA		0.477095964	0.334857415	0.071440856	0.409790836	0.379551383	0.339618977
NA		0.031385625	0.043246220	0.031441109	0.037081418	0.040313316	0.049200794

NA	0.024043981	0.027811472	0.024422513	0.027493528	0.024598147	0.029331833
NA	0.038533351	0.085397631	0.036520136	0.057194526	0.112769481	0.064326007
NA	0.107174050	0.145052024	0.105944036	0.114812581	0.115175792	0.103147068
NA	0.024990819	0.031342245	0.022505729	0.018761651	0.022499706	0.040179071
NA	0.095528807	0.104975132	0.083565874	0.082604947	0.068860704	0.082783830
NA	0.060071395	0.077472285	0.052126533	0.084213622	0.049638286	0.108661354
NA	0.029605287	0.036820453	0.036783205	0.035420253	0.032867431	0.044941548
NA	0.133702818	0.136736719	0.122187118	0.120626498	0.116039107	0.139508566
NA	0.036745991	0.029431752	0.027417888	0.033779199	0.032236661	0.044782672
NA	0.442786427	0.458296441	0.437002922	0.408094261	0.471031873	0.430963820
NA	0.024872252	0.021079014	0.022246474	0.022483114	0.027475694	0.036296406
NA	0.268787528	0.269702217	0.208301447	0.193764335	0.148980591	0.170341290
NA	0.741630219	0.651173910	0.733122854	0.707094708	0.766092740	0.614434953
NA	0.068091754	0.105917374	0.064901150	0.043744109	0.034081449	0.099280682
NA	0.378334011	0.488527350	0.406393273	0.475386760	0.525245287	0.501877633
NA	0.056197490	0.054465473	0.037268477	0.048897755	0.059310264	0.067528696
NA	0.028676386	0.026030510	0.029285754	0.023954365	0.035178722	0.034699333
NA	0.054841949	0.054781994	0.049892920	0.045991640	0.067721877	0.056959329
NA	0.150456583	0.159583421	0.105737382	0.122270931	0.151858957	0.137633239
NA	0.025970240	0.027536019	0.023539966	0.022710046	0.023106099	0.034264377
NA	0.850514872	0.895267483	0.873265284	0.874669662	0.847562472	0.843220741
NA	0.018333789	0.025440241	0.017771285	0.020766843	0.013077244	0.025374983
NA	0.035159941	0.038599703	0.019651419	0.030468139	0.030788856	0.029767296
NA	0.343768234	0.352438659	0.303858199	0.422548807	0.513406030	0.458355386
NA	0.041085484	0.047495823	0.030563288	0.038549310	0.023243863	0.031002618
NA	0.384305889	0.425860037	0.349594681	0.368891720	0.370067144	0.367839661
NA	0.048355590	0.032365373	0.044406245	0.051717889	0.045976760	0.038999901
TRUE	0.138570619	0.219149254	0.136139622	0.134518930	0.150589867	0.152611359
NA	0.911303272	0.920017734	0.868797614	0.890091774	0.891129948	0.878268241
NA	0.026074124	0.020403948	0.013869150	0.013403888	0.019509791	0.022972446
NA	0.731034332	0.755251436	0.697691949	0.693557447	0.674179888	0.727359337
NA	0.067629214	0.050742364	0.043973178	0.031130767	0.023672319	0.037401768
NA	0.027238698	0.040179838	0.028872420	0.024690036	0.024111031	0.037800713
NA	0.524372380	0.567634602	0.459676440	0.526521673	0.533024500	0.485639967
NA	0.951979169	0.955904085	0.931235096	0.957020346	0.962189566	0.897683038
NA	0.052668085	0.047519669	0.035178077	0.035384043	0.045004221	0.054589062
NA	0.133244460	0.115827859	0.114913272	0.109629590	0.162977506	0.148322921
NA	0.045175354	0.040476107	0.024958698	0.033263854	0.031851337	0.035712019
NA	0.049832221	0.054341080	0.048218802	0.041389346	0.040850867	0.075146920
NA	0.284181026	0.348839746	0.272885309	0.264469764	0.293292184	0.305138190
NA	0.014395185	0.015451782	0.015274260	0.014642169	0.018586913	0.021961806
NA	0.440691519	0.471316636	0.392522662	0.274037825	0.274000767	0.313824235
NA	0.298866931	0.291228566	0.249286510	0.245510878	0.271068655	0.265453337
TRUE	0.159940529	0.215335038	0.136153396	0.160111279	0.190655838	0.168565646

NA		0.559342530	0.575398654	0.511823134	0.542269405	0.473845568	0.533553247
NA		0.395909653	0.422459632	0.348366657	0.419207038	0.544788701	0.405926843
NA		0.289367920	0.298199555	0.327365764	0.296657397	0.346260688	0.307511146
NA		0.069968705	0.081286375	0.057675676	0.055470091	0.048637999	0.049560198
NA		0.217645208	0.219210309	0.190092054	0.185849106	0.181035745	0.228092092
	TRUE	0.471883997	0.468239956	0.471003294	0.499244523	0.532518507	0.412750795
NA		0.045777661	0.051340944	0.053073602	0.051297368	0.039699958	0.058794282
NA		0.060535726	0.083994217	0.078353232	0.077025106	0.086896890	0.081011438
NA		0.031667745	0.030362944	0.023035688	0.027968060	0.018863758	0.031961454
NA		0.052139754	0.056563965	0.059591696	0.049864261	0.053238747	0.058036121
NA		0.902423662	0.894459424	0.858632451	0.875252271	0.894140656	0.831892767
NA		0.905781086	0.901095856	0.874602161	0.847330226	0.831531512	0.849012910
NA		0.043095281	0.040055443	0.027462632	0.025954658	0.031507948	0.036526340
NA		0.774012077	0.743116569	0.715325024	0.710887428	0.731824731	0.712913155
NA		0.033161607	0.026017018	0.030151635	0.029532611	0.027152112	0.044362317
NA		0.019645036	0.028399135	0.021647619	0.017502652	0.013292422	0.035430233
NA		0.063646579	0.055124024	0.042585344	0.058724102	0.052203103	0.058449951
NA		0.056183444	0.085537731	0.059709419	0.047112551	0.056052605	0.081998178
NA		0.252070155	0.244285461	0.281475755	0.231967800	0.191344487	0.368485353
NA		0.115551898	0.085030392	0.061188898	0.088769280	0.051957669	0.064403174
NA		0.928429554	0.946275846	0.915819065	0.932702153	0.936359836	0.901469954
NA		0.858481902	0.872339369	0.849198992	0.845673831	0.820781953	0.813014938
NA		0.546092317	0.549338709	0.581231853	0.557124243	0.600285215	0.537679858
NA		0.040320436	0.028988427	0.024426040	0.023720309	0.022657458	0.033764760
NA		0.579122082	0.631623132	0.602497827	0.556122951	0.574242363	0.684852724
NA		0.028554610	0.019487224	0.021762434	0.025043527	0.023295107	0.029635209
NA		0.110368713	0.121871752	0.107092514	0.119901017	0.189608498	0.097158906
NA		0.024284323	0.013963178	0.020899423	0.014864257	0.016531448	0.019718909
	TRUE	0.048933422	0.031110193	0.036481078	0.032001749	0.034100007	0.044875582
NA		0.052762087	0.024351002	0.055493844	0.043726666	0.035982229	0.065323334
NA		0.830673711	0.819485638	0.840408152	0.836051561	0.852178138	0.849471223
NA		0.948150686	0.946198505	0.925469515	0.949014531	0.959121648	0.889433977
NA		0.052057446	0.047117976	0.039687644	0.040584268	0.040676037	0.064311597
NA		0.029905215	0.017983793	0.028824307	0.022601809	0.024165243	0.037636903
NA		0.034570836	0.033414873	0.028847820	0.030728711	0.032323356	0.036444090
	TRUE	0.775905143	0.804570647	0.755952413	0.776249768	0.828654289	0.808665566
	TRUE	0.922433740	0.929158882	0.890957999	0.906674635	0.912882429	0.872066504
	TRUE	0.446341352	0.450980773	0.448601297	0.451198201	0.544234671	0.432655950
NA		0.503664422	0.481009830	0.524848384	0.499801760	0.473129017	0.469427807
NA		0.052637260	0.034655290	0.036375647	0.047414244	0.044459289	0.044622721
NA		0.265824205	0.251791849	0.145648553	0.188448841	0.293004443	0.221356704
NA		0.049911033	0.027652592	0.019998941	0.033881987	0.031140657	0.034203577
NA		0.535400877	0.583682774	0.491418327	0.477869640	0.501305161	0.515496906
NA		0.029058662	0.025243551	0.028623160	0.025718038	0.030003570	0.032226472

NA		0.702272057	0.721878484	0.615978491	0.653832793	0.644625350	0.609406891
NA		0.856046299	0.862576663	0.822966988	0.867512338	0.873298485	0.862184365
NA		0.040026734	0.034990757	0.033836103	0.035328232	0.036800149	0.052377543
NA		0.463585477	0.507055470	0.411662531	0.497142116	0.508886827	0.534336725
	TRUE	0.728151444	0.737916162	0.761632432	0.762362337	0.779826285	0.745854184
NA		0.043107146	0.042512199	0.038356233	0.028246081	0.034161747	0.041035786
	TRUE	0.093636143	0.086323079	0.093655921	0.069051601	0.070950829	0.047388089
	TRUE	0.743114455	0.775566945	0.738284064	0.725495094	0.739072436	0.711873839
NA		0.014880665	0.014839704	0.016250490	0.018398574	0.017438404	0.021749498
NA		0.812377952	0.837525741	0.791796398	0.832447923	0.863214756	0.859908587
NA		0.066553562	0.071737681	0.054556503	0.061223522	0.064639251	0.070835845
	TRUE	0.780522799	0.765041336	0.771230733	0.752449614	0.804434982	0.693024476
NA		0.763987684	0.834492295	0.754655170	0.671376676	0.784273922	0.706148310
NA		0.556081934	0.592314327	0.481279686	0.641626128	0.797549094	0.706298874
NA		0.881005786	0.914691366	0.874127407	0.382418716	0.902203249	0.868944112
NA		0.030059883	0.029926200	0.035717519	0.032581370	0.030055797	0.050210015
NA		0.117652312	0.122563203	0.112193990	0.087818529	0.089143905	0.126585589
NA		0.828706623	0.876609704	0.864744101	0.877994883	0.867136514	0.844605891
NA		0.061117047	0.059811880	0.050912953	0.056153759	0.051603563	0.055263780
NA		0.018317913	0.018453112	0.017687148	0.013033560	0.015280960	0.026190919
	TRUE	0.867056902	0.892797429	0.871409626	0.856171295	0.859506110	0.862611903
NA		0.979105800	0.982017122	0.974426764	0.969636836	0.980388962	0.963121517
NA		0.051523916	0.050642471	0.037875245	0.043107379	0.036747574	0.042564870
NA		0.881885221	0.904690823	0.871738206	0.845718862	0.870457932	0.859468698
NA		0.020479171	0.019756491	0.017366925	0.017052147	0.021415707	0.025551164
	TRUE	0.050347108	0.188041587	0.069810593	0.088265730	0.121672050	0.128578683
NA		0.037072581	0.046418884	0.047791513	0.031528863	0.047147838	0.045542569
NA		0.969437737	0.967997943	0.967482456	0.958119848	0.961169808	0.955680246
NA		0.795467768	0.847906031	0.813162535	0.860331360	0.926851808	0.873541112
NA		0.362878675	0.387583103	0.326147067	0.362098770	0.379841720	0.328753429
NA		0.054439093	0.060555572	0.056828812	0.043000886	0.053676817	0.070433141
NA		0.633595775	0.713691875	0.577977863	0.693627085	0.664070365	0.724016300
	TRUE	0.682346277	0.653824259	0.597781709	0.627956810	0.632341554	0.550642286
	TRUE	0.445273467	0.448443279	0.426831268	0.494102354	0.522378893	0.447146174
NA		0.037927840	0.034649368	0.036700805	0.034024090	0.024452639	0.035080452
NA		0.079567390	0.095955466	0.065724494	0.056336352	0.077131173	0.065986393
NA		0.060936759	0.088385285	0.077695741	0.058283588	0.055044511	0.063530717
NA		0.027810078	0.025051952	0.021192955	0.024963207	0.019487400	0.037725254
NA		0.432798289	0.474855627	0.364367324	0.459256567	0.478300915	0.460973609
NA		0.884892887	0.901631069	0.866941956	0.862775750	0.853034462	0.879067557
NA		0.044431290	0.050372608	0.042959941	0.027524856	0.027112870	0.051907580
NA		0.630881297	0.686155956	0.559765512	0.610293158	0.628695065	0.579338508
NA		0.041351296	0.028912824	0.036837172	0.023578381	0.028100684	0.030108338
NA		0.252574703	0.335850030	0.233234152	0.214717433	0.274405778	0.271007617

NA		0.162003374	0.177835356	0.142434138	0.126975917	0.088074068	0.096095432
NA		0.029671339	0.024547360	0.022041138	0.022616491	0.018346936	0.024744869
NA		0.873909809	0.887807012	0.848101123	0.856279694	0.880351185	0.856419908
	TRUE	0.857785817	0.843919371	0.810307941	0.827964721	0.842514494	0.822181541
NA		0.134448154	0.122759485	0.107039066	0.112400516	0.208971415	0.114565463
NA		0.048156577	0.036265225	0.056786004	0.048085676	0.052476403	0.055833020
NA		0.029332389	0.032404699	0.024926451	0.029151575	0.025361344	0.041858073
NA		0.029097133	0.025588731	0.029076726	0.022512557	0.036705921	0.030926996
NA		0.821179475	0.840973850	0.847884803	0.807414733	0.803301055	0.829208618
NA		0.386581631	0.429550706	0.370320006	0.375193644	0.408798447	0.339174219
NA		0.036928989	0.030451137	0.028460260	0.031878307	0.030088600	0.052539582
NA		0.384096054	0.428108297	0.320410114	0.350884078	0.272756120	0.296611513
NA		0.052776628	0.055133226	0.064868909	0.067682843	0.063445033	0.059560487
NA		0.153432434	0.155131378	0.117372601	0.137410363	0.086457335	0.111762005
NA		0.624688511	0.608249370	0.595997443	0.590651510	0.582794302	0.503726747
NA		0.649097010	0.674217732	0.606659438	0.581834837	0.585685801	0.589194267
	TRUE	0.367568833	0.421982030	0.323207884	0.146330129	0.263195572	0.251157157
NA		0.131266752	0.124114547	0.110717057	0.119743532	0.116129031	0.102911548
	TRUE	0.064280241	0.061220157	0.074504770	0.048416082	0.147696099	0.050731349
NA		0.045250386	0.049160290	0.067204101	0.044495382	0.050853931	0.040095265
NA		0.021238834	0.018351745	0.020785866	0.020149893	0.014757553	0.021052194
	TRUE	0.434392935	0.482830952	0.338153286	0.435034295	0.305110802	0.428892348
NA		0.058088541	0.070134029	0.072063746	0.059677084	0.041673955	0.054733737
NA		0.040858122	0.043068687	0.036438648	0.037922509	0.029806516	0.045701474
NA		0.025927070	0.024688759	0.020821860	0.022256728	0.018589695	0.033076919
NA		0.063881688	0.081396254	0.053025300	0.070279998	0.059167368	0.076174827
	TRUE	0.347600716	0.358769100	0.287289353	0.314050121	0.342757710	0.297331287
NA		0.058267452	0.097086730	0.052157777	0.084073323	0.048135271	0.089419605
NA		0.056522417	0.046399352	0.041112205	0.048855780	0.032200884	0.052357283
NA		0.129991429	0.140275281	0.096218362	0.079466116	0.080358638	0.086311802
NA		0.024874680	0.025801116	0.022065851	0.018876045	0.024299830	0.037046194
NA		0.074194244	0.079689558	0.052399891	0.055620823	0.028869295	0.062419691
NA		0.394169419	0.414290761	0.334870344	0.352931550	0.340309620	0.321193562
NA		0.574620099	0.572171114	0.499913340	0.593411805	0.706637053	0.598427302
NA		0.460429046	0.467097474	0.407668484	0.353112692	0.465704183	0.342768552
NA		0.816855410	0.835446935	0.794378249	0.815176820	0.787273335	0.867270285
NA		0.060298168	0.060727925	0.058458038	0.061163449	0.049456929	0.057633444
	TRUE	0.047984765	0.058282068	0.041871321	0.077661589	0.084547053	0.078089158
NA		0.018292905	0.020137525	0.021419341	0.020420144	0.024912724	0.023157302
NA		0.169934038	0.178756584	0.148199517	0.145943320	0.150952017	0.167178287
NA		0.874717672	0.893588301	0.901739504	0.886039834	0.864795466	0.858695962
NA		0.018115592	0.015272486	0.020736410	0.018169521	0.017258059	0.022373624
NA		0.019116714	0.017196009	0.015750513	0.018411329	0.021869855	0.020856304
NA		0.036711239	0.042408158	0.033418932	0.038440519	0.026730113	0.061877920

NA	0.057265096	0.035439628	0.040244956	0.041530195	0.048723819	0.043078901
NA	0.022769084	0.017381091	0.024010180	0.025572719	0.025799125	0.038832896
NA	0.024703211	0.036192869	0.031094379	0.032403505	0.026765420	0.029585668
NA	0.786087211	0.818845982	0.820086520	0.770840976	0.787901778	0.741559364
NA	0.037443670	0.035202348	0.039509016	0.046066138	0.043006506	0.032233355
NA	0.821809515	0.798528690	0.750335983	0.819784976	0.830538620	0.780245063
TRUE	0.804175600	0.822564884	0.806658254	0.825238362	0.822455808	0.799676483
NA	0.070206077	0.085176758	0.061967759	0.066391723	0.049607125	0.083436199
NA	0.684046712	0.712682633	0.622973049	0.673732560	0.795805932	0.738279793
NA	0.037552763	0.025392106	0.021139199	0.026087042	0.019361955	0.032456562
NA	0.446134542	0.903975698	0.496616272	0.720982958	0.498366071	0.772718081
NA	0.021668344	0.021705900	0.023657929	0.019342566	0.025912759	0.025592702
NA	0.049238791	0.047396335	0.040943945	0.041870726	0.030688875	0.045432470
NA	0.070409654	0.059337592	0.050520468	0.062378138	0.036760477	0.058929763
NA	0.689114446	0.784800580	0.665354564	0.722620648	0.740578805	0.717619122
NA	0.024498687	0.018829573	0.013448508	0.021089799	0.018123685	0.019483673
NA	0.847663477	0.848865964	0.836954339	0.742409620	0.780541850	0.769382234
NA	0.819048199	0.897939582	0.845041934	0.852043927	0.857989454	0.861602393
NA	0.033869162	0.031704048	0.021327095	0.035979417	0.025604802	0.029366813
NA	0.290771003	0.318800555	0.256304045	0.359931219	0.391367441	0.265325373
NA	0.041190754	0.043577379	0.048738812	0.046114737	0.035371514	0.073329166
NA	0.032300259	0.042710634	0.040509217	0.045129234	0.032187690	0.052590286
NA	0.913516902	0.926711589	0.875613162	0.928087516	0.885521946	0.909181573
NA	0.026508248	0.021624570	0.024947900	0.017421237	0.028464139	0.027088329
NA	0.732111011	0.734285635	0.704426376	0.670274871	0.664395812	0.625747604
NA	0.148260465	0.214762235	0.178345315	0.195939188	0.173232794	0.244382542
NA	0.156864485	0.220750672	0.142775311	0.160133178	0.148764647	0.172884820
NA	0.092050382	0.107613543	0.085606971	0.096211687	0.068686040	0.101342531
NA	0.274246556	0.339308822	0.162898243	0.132695478	0.164550641	0.376619743
NA	0.585835929	0.605154908	0.680042334	0.563829226	0.629835387	0.679853425
NA	0.032299428	0.032530690	0.030248139	0.032448708	0.029817040	0.044377543
NA	0.407552298	0.425764172	0.388515507	0.393047241	0.376116295	0.358145433
NA	0.059353325	0.061862509	0.044452199	0.052484128	0.048060732	0.044254308
NA	0.050756403	0.031895196	0.027981208	0.038615950	0.031503945	0.039842713
NA	0.131215808	0.156721580	0.125203369	0.154211048	0.150172890	0.139390798
NA	0.244323612	0.239449740	0.199179213	0.194048448	0.223238680	0.220940417
NA	0.706232387	0.773433269	0.714010002	0.682755790	0.703041710	0.777872215
NA	0.917359639	0.909299191	0.905263530	0.899107067	0.905517349	0.902527654
NA	0.027082086	0.026563871	0.035796934	0.030336240	0.027012058	0.038776915
TRUE	0.427318341	0.483386132	0.357295313	0.408770568	0.551623222	0.486729759
NA	0.102558554	0.103362069	0.056514030	0.054039432	0.064053099	0.096673207
NA	0.814933576	0.865656516	0.752562821	0.806228235	0.788285280	0.782722722
NA	0.356281719	0.389231354	0.372884884	0.356988705	0.364804005	0.246205093
NA	0.568570080	0.664987268	0.586667849	0.647562731	0.693083539	0.764155088

NA		0.254299345	0.287526239	0.239576448	0.277452659	0.246445852	0.294083744
NA		0.891204876	0.909260607	0.889066180	0.871080187	0.887230182	0.874033867
NA		0.833378662	0.823776319	0.797740417	0.794135172	0.814153224	0.769909821
	TRUE	0.795956107	0.847130683	0.792316378	0.814357875	0.834962234	0.806221729
NA		0.031929870	0.031304603	0.029401674	0.028722378	0.029033758	0.038870811
NA		0.269081961	0.276035810	0.273587730	0.195677983	0.246424669	0.260148850
NA		0.077480091	0.086973763	0.069469462	0.073974596	0.069486475	0.077650093
NA		0.032304582	0.026033712	0.040121207	0.030510961	0.040073093	0.033433180
NA		0.901983382	0.917829060	0.882189000	0.883003003	0.910883172	0.871640242
NA		0.322706153	0.346175676	0.310031608	0.253329699	0.266696380	0.226525806
NA		0.050350476	0.054627511	0.046247420	0.041675696	0.047237262	0.058585723
	TRUE	0.129234465	0.131737773	0.114247542	0.159638365	0.311092288	0.163422647
NA		0.020709376	0.023504389	0.015983763	0.018521020	0.016706503	0.027181929
NA		0.697868033	0.733666828	0.646689434	0.548325224	0.686163203	0.632150593
	TRUE	0.137622507	0.217365303	0.158413518	0.151824033	0.137076565	0.159834509
NA		0.603656078	0.725780929	0.617060682	0.669073624	0.735888778	0.681972428
NA		0.034270039	0.032697334	0.025398270	0.031938576	0.025579448	0.029676181
NA		0.144852816	0.180840430	0.161817889	0.157213217	0.270386787	0.186223944
NA		0.076322019	0.065533922	0.067308662	0.051551553	0.030999746	0.074985052
NA		0.039609632	0.025535198	0.024191879	0.026099746	0.020335132	0.029271928
NA		0.972705726	0.980591417	0.973273693	0.976085268	0.977794768	0.959899366
NA		0.145692161	0.123763888	0.128131115	0.182809061	0.182466418	0.157558596
	TRUE	0.502717146	0.538516533	0.517162601	0.570392310	0.475463795	0.552807420
NA		0.735222521	0.728288405	0.754001383	0.768341240	0.767218525	0.689301216
NA		0.176604350	0.135170534	0.192644023	0.125105129	0.123517956	0.082920558
NA		0.275831410	0.236420099	0.202794701	0.218927225	0.244824580	0.225874994
NA		0.060093364	0.052904647	0.047716273	0.052719055	0.052666496	0.059775348
NA		0.023034524	0.025410636	0.025754849	0.028762591	0.025502536	0.036525168
NA		0.156884035	0.087271162	0.079110938	0.066863563	0.076966458	0.163260983
NA		0.874944263	0.914918803	0.842546183	0.889568032	0.874702466	0.864257914
NA		0.052203603	0.049135461	0.041638165	0.048058126	0.053727290	0.050700495
NA		0.055027566	0.048855396	0.037157869	0.049687079	0.049270219	0.055077084
NA		0.733720367	0.808776787	0.689094008	0.694459852	0.684394041	0.666182316
NA		0.075113907	0.079875226	0.059605665	0.067424445	0.062321150	0.071822964
NA		0.526793239	0.554190291	0.478977651	0.444523881	0.495355120	0.457367038
NA		0.507011936	0.416078305	0.488925758	0.426771119	0.425658772	0.379455996
NA		0.916490328	0.886326803	0.871516446	0.887985991	0.915762136	0.849154721
	TRUE	0.124436115	0.139032792	0.084042415	0.152483721	0.208384635	0.138108991
NA		0.663830773	0.690365074	0.601041882	0.502916256	0.537914438	0.513680941
NA		0.125325128	0.154908493	0.131028557	0.134469198	0.134017692	0.161479817
NA		0.360763773	0.368732843	0.324523479	0.363381782	0.370770217	0.383713694
NA		0.652427403	0.622424731	0.625029551	0.652737009	0.626626944	0.632181504
NA		0.762170086	0.804728358	0.744968368	0.767636813	0.756396040	0.720736405
NA		0.272946681	0.306028115	0.252966979	0.281837699	0.283431138	0.275827733

NA		0.021378837	0.017385451	0.020108157	0.015692800	0.019825658	0.024235467
NA		0.060439447	0.057574078	0.082585835	0.047403129	0.085428243	0.066325290
NA		0.089915920	0.108371414	0.124763111	0.088092327	0.077786338	0.163884233
NA		0.617532074	0.669853925	0.603376227	0.516156820	0.605068528	0.544453970
NA		0.427031373	0.505185321	0.446969827	0.446450187	0.489943143	0.497037918
	TRUE	0.216709447	0.262670546	0.212788433	0.208700497	0.177113223	0.198727797
NA		0.896939662	0.891463070	0.850327847	0.883242525	0.903659501	0.767119072
NA		0.300929062	0.305439546	0.275169641	0.354783377	0.474945396	0.471991247
NA		0.042921401	0.033235098	0.037330159	0.041339197	0.044376408	0.053608576
	TRUE	0.800499689	0.844072690	0.735395054	0.762786130	0.767625641	0.775687057
NA		0.830959300	0.812023246	0.761731170	0.811593228	0.789618231	0.835166757
	TRUE	0.177421815	0.193743599	0.148721199	0.218513828	0.251777438	0.241804298
NA		0.590681160	0.647031652	0.673379657	0.645221218	0.720094064	0.730557935
NA		0.782215843	0.745001713	0.924433585	0.750669837	0.828834826	0.755849460
NA		0.024043886	0.027250139	0.021074015	0.033218220	0.035756538	0.033825225
NA		0.489753435	0.524691464	0.473439294	0.472363486	0.527285929	0.545405674
NA		0.042879786	0.041789686	0.024830858	0.034111180	0.033218987	0.058158331
NA		0.067234508	0.057787819	0.055811693	0.065949406	0.061865496	0.070013659
NA		0.033392514	0.028135848	0.021546601	0.020025646	0.020424827	0.025097611
NA		0.571894244	0.611197221	0.551307274	0.540239157	0.622531092	0.637777205
NA		0.961183328	0.957990270	0.951800575	0.964214563	0.963743200	0.924112546
NA		0.068954663	0.064679483	0.087563039	0.081545295	0.070595284	0.115499744
NA		0.573049509	0.613366028	0.561912987	0.570649612	0.627255100	0.567654085
	TRUE	0.162492147	0.192131506	0.150409900	0.178357950	0.141137920	0.152144066
NA		0.445987497	0.494819074	0.438880308	0.422541825	0.407795129	0.448390754
NA		0.025128112	0.026304999	0.027398137	0.021072173	0.021868790	0.030181784
NA		0.779684508	0.851913561	0.735312180	0.856242615	0.926453189	0.867284352
NA		0.038070326	0.026092230	0.034772469	0.035032298	0.026815667	0.041717075
NA		0.571781530	0.688080456	0.553777207	0.511679599	0.572210845	0.613807921
NA		0.055126622	0.048619485	0.046676432	0.055905762	0.059711282	0.045638237
NA		0.328196947	0.353522274	0.277755746	0.248936843	0.353707386	0.276073583
NA		0.308609757	0.248775541	0.304344969	0.480274663	0.557188984	0.362958325
NA		0.037371177	0.046061557	0.032357670	0.038566987	0.026537115	0.082712582
NA		0.024509862	0.029132354	0.022378425	0.024861652	0.031968553	0.033534051
NA		0.263524936	0.361331713	0.262565515	0.259642014	0.336119551	0.273778979
NA		0.018485718	0.020216401	0.019182942	0.020138937	0.018806949	0.022321852
NA		0.731088404	0.736845431	0.714549550	0.741458244	0.762979172	0.697616212
NA		0.030205791	0.032332884	0.019645886	0.028060267	0.021161794	0.028239910
NA		0.657099514	0.707457614	0.568261354	0.682545397	0.795684262	0.684747092
NA		0.265538767	0.235859030	0.289165151	0.268527543	0.284920242	0.193555995
NA		0.027433351	0.024653114	0.032745859	0.031676874	0.027377858	0.037626030
NA		0.022554381	0.020297493	0.029066585	0.023467814	0.020394306	0.032406391
NA		0.079048942	0.076276272	0.064681468	0.066154461	0.069500104	0.071407804
	TRUE	0.734914345	0.724553797	0.683448529	0.635247985	0.760348714	0.710712181

NA		0.407291919	0.436694282	0.371083806	0.386832093	0.454077635	0.454892177
NA		0.868276462	0.895907216	0.878250933	0.822044331	0.841812912	0.852954615
NA		0.487878730	0.489799678	0.436799782	0.433477402	0.566739258	0.490993937
NA		0.482174541	0.518100540	0.446202161	0.507395344	0.538329232	0.451095541
NA		0.117733913	0.081432591	0.160186258	0.060352527	0.046113940	0.137407968
NA		0.545539075	0.560106546	0.465619832	0.470514391	0.480082556	0.509817309
NA		0.524968840	0.490985369	0.431758930	0.550287611	0.576849021	0.528243071
NA		0.742681478	0.766424273	0.743967230	0.766705804	0.871448739	0.808486231
NA		0.372206655	0.433782759	0.313723116	0.340091032	0.391009993	0.395706219
NA		0.025371361	0.029277266	0.024505169	0.024436318	0.025126546	0.035965150
NA		0.079494655	0.089791775	0.069208446	0.058116002	0.044455657	0.051662095
NA		0.033497686	0.047450898	0.037230555	0.045855020	0.040031920	0.051238985
NA		0.816321827	0.772115386	0.788782988	0.796085578	0.835732052	0.724479308
NA		0.135349113	0.117288040	0.091488654	0.168625707	0.194583318	0.139733365
NA		0.694723345	0.776493594	0.699249915	0.713727087	0.763964869	0.655205873
NA		0.176645163	0.189228005	0.144804156	0.149531257	0.096616752	0.088672837
NA		0.015391735	0.018632312	0.017794606	0.015715579	0.015413910	0.022609147
NA		0.369955049	0.376506906	0.238456456	0.295962623	0.386909365	0.315645977
	TRUE	0.306326557	0.351653730	0.279867493	0.260230099	0.325863488	0.321932382
NA		0.707349761	0.733667904	0.689139611	0.591606638	0.635487062	0.653034607
NA		0.868045895	0.914975861	0.858250794	0.884440903	0.911515526	0.814486127
NA		0.038455858	0.031024948	0.039870805	0.040528087	0.028882932	0.058853203
NA		0.035465610	0.031469238	0.022082291	0.027854577	0.032004992	0.045709593
NA		0.064864875	0.046357544	0.051814543	0.046070330	0.038490686	0.063858128
	TRUE	0.696162334	0.694018740	0.573383160	0.615672104	0.530349652	0.568351844
NA		0.478959358	0.461412034	0.463897814	0.480378742	0.494271919	0.473869490
NA		0.138836310	0.129273752	0.105262198	0.129310404	0.210491516	0.168289669
NA		0.104092354	0.085594933	0.094528487	0.083875029	0.113936851	0.111615310
NA		0.048651847	0.047654043	0.038850723	0.030659748	0.028124774	0.039571532
	TRUE	0.193411297	0.202271438	0.184372063	0.187748230	0.187155563	0.174290049
NA		0.070252287	0.075467749	0.060119961	0.068599747	0.055932953	0.085747199
NA		0.471494263	0.509133819	0.410016269	0.434459487	0.458512915	0.454796463
NA		0.032907695	0.028017034	0.036738532	0.037204420	0.028235130	0.042343003
NA		0.490433605	0.559700337	0.468327558	0.451855242	0.396097164	0.429549791
NA		0.918644144	0.924995714	0.883089993	0.907903358	0.905215201	0.861755177
NA		0.867422583	0.899327489	0.891940909	0.903429004	0.896980168	0.899719594
NA		0.396280964	0.422654394	0.360989646	0.342206743	0.354982359	0.316815220
NA		0.015453182	0.014297009	0.018852968	0.017195057	0.019112076	0.020974368
NA		0.851162232	0.879411354	0.806169147	0.877055504	0.801144420	0.848637655
	TRUE	0.030180857	0.046395302	0.032789119	0.041158184	0.022692955	0.048009469
NA		0.765980919	0.826197998	0.788873132	0.781549359	0.744502386	0.820930478
NA		0.652936158	0.643590122	0.581785965	0.583221671	0.670727291	0.594792194
NA		0.049637437	0.050955286	0.070196798	0.056934404	0.050506139	0.115950534
NA		0.035088565	0.028186620	0.029567324	0.028763035	0.032023452	0.041686984

NA		0.041111037	0.041613849	0.032631376	0.033875172	0.039611942	0.043265576
NA		0.628757607	0.670448567	0.576222109	0.517454949	0.589535667	0.529826040
NA		0.963375336	0.973169874	0.967987255	0.966209828	0.962059578	0.962218204
NA		0.021837052	0.020001194	0.020334614	0.022704322	0.023206316	0.024265365
NA		0.031185942	0.026068313	0.024520842	0.027180959	0.028244169	0.033325935
NA		0.891808234	0.893887713	0.849618121	0.881867813	0.915611782	0.761155589
NA		0.043374095	0.048374030	0.040000857	0.040067777	0.024205381	0.048489654
NA		0.570778081	0.662079643	0.595250142	0.606422030	0.662583830	0.672588297
NA		0.042016771	0.036712175	0.034448208	0.031299535	0.037654003	0.046873710
NA		0.038872162	0.032867062	0.025451476	0.031621405	0.037375649	0.040691658
NA		0.388677225	0.414830197	0.337740987	0.361484364	0.384080427	0.372500851
NA		0.044133359	0.034457527	0.036016892	0.035895257	0.035385962	0.050992558
NA		0.400207421	0.451723553	0.387678522	0.385646014	0.425880608	0.408950947
NA		0.095875705	0.138921235	0.150683201	0.079060121	0.084468545	0.197050747
	TRUE	0.117618092	0.137998478	0.092448276	0.118517994	0.123440823	0.127890952
NA		0.346129608	0.354080962	0.319454980	0.282322751	0.325071045	0.328364261
NA		0.562059684	0.783230054	0.615275206	0.663067440	0.655335559	0.733823077
NA		0.817826151	0.869065135	0.808101128	0.795424613	0.826876442	0.851699673
NA		0.319932549	0.313789978	0.247614325	0.241051500	0.183283706	0.209974044
NA		0.820502757	0.794404791	0.767221719	0.749022792	0.680033683	0.738125998
	TRUE	0.043945220	0.040792126	0.036149662	0.046053242	0.037709828	0.048504252
NA		0.025465858	0.023568312	0.030538719	0.027738057	0.022804010	0.031813066
NA		0.046065037	0.061268970	0.057799786	0.049955318	0.051509356	0.089655620
NA		0.041293083	0.028555899	0.032986662	0.036374595	0.039766687	0.050233269
NA		0.042076576	0.028825954	0.035523968	0.027262908	0.031675594	0.053088371
NA		0.263236908	0.357825180	0.211744473	0.220307317	0.183747570	0.183899647
NA		0.080373969	0.069224190	0.065723236	0.069127365	0.070902457	0.103025445
NA		0.025840935	0.020897375	0.023993847	0.023619582	0.028369031	0.041527825
NA		0.173751847	0.099195438	0.104301181	0.107316174	0.166064058	0.054854054
NA		0.572666243	0.609220125	0.575937462	0.629593457	0.562358972	0.519208678
NA		0.029151553	0.029921508	0.027303211	0.028807827	0.036203472	0.039900148
NA		0.904479393	0.933816348	0.913396767	0.914194002	0.881950281	0.894215966
NA		0.061651406	0.078345575	0.091084487	0.062045222	0.057917209	0.130991561
NA		0.044092287	0.035226294	0.040009442	0.033003449	0.019389947	0.040372297
NA		0.590162200	0.677531491	0.603425412	0.543384535	0.569173543	0.557845070
NA		0.209474186	0.205849627	0.161721084	0.155338119	0.182227153	0.130752981
NA		0.056744515	0.073509879	0.058439878	0.055184326	0.053531555	0.083775590
NA		0.307665681	0.331581594	0.345643387	0.393168761	0.280579297	0.328295948
NA		0.279958434	0.307280065	0.254165392	0.272680440	0.251763020	0.322850420
NA		0.769013972	0.777893027	0.686922777	0.741648638	0.695742315	0.684961059
NA		0.033347246	0.034081055	0.041689756	0.038391848	0.043792502	0.055210195
NA		0.056601374	0.056182378	0.037189599	0.043237201	0.044126587	0.052196037
NA		0.404272329	0.403717216	0.381660048	0.362541450	0.377745353	0.440639671
NA		0.769889698	0.739944000	0.798791740	0.769604966	0.824203736	0.771971075

NA	0.023547988	0.021871873	0.046891961	0.042359291	0.037178356	0.036217169
NA	0.033264358	0.039176578	0.025576814	0.023440274	0.039414513	0.033429080
NA	0.020585103	0.029026811	0.017928332	0.032548983	0.036463957	0.033417348
NA	0.030866820	0.029151253	0.034941383	0.036331956	0.024098175	0.031103730
NA	0.039909711	0.026165129	0.029765153	0.034842756	0.026045309	0.033034727
NA	0.058695078	0.056309990	0.037454748	0.045303987	0.040430915	0.055283056
NA	0.049662817	0.056149059	0.041663854	0.035551518	0.037077995	0.057305495
NA	0.022594357	0.027754480	0.032313138	0.037043134	0.040676338	0.034729948
NA	0.011649348	0.011609539	0.013499663	0.017331303	0.012560577	0.019477605
NA	0.331267059	0.339609546	0.306724240	0.392038239	0.278700993	0.351106080
NA	0.058578779	0.051809321	0.063303573	0.052416004	0.061996677	0.091739328
NA	0.073607411	0.057244656	0.045493884	0.050328785	0.043102678	0.047861155
NA	0.039209792	0.037945690	0.038353308	0.026329060	0.024633963	0.044413490
NA	0.560748556	0.536974520	0.510123070	0.489104588	0.473752631	0.528452086
NA	0.927275593	0.950431321	0.925382831	0.941129276	0.950662948	0.927318534
NA	0.793784133	0.813982326	0.662550486	0.641145153	0.573786630	0.503426414
NA	0.703835285	0.691313830	0.630404090	0.653931997	0.697145226	0.573458595
NA	0.670318751	0.717375039	0.625049478	0.613789030	0.695288131	0.631256106
NA	0.903961979	0.919868115	0.870995067	0.834791085	0.816908900	0.828120778
NA	0.862363366	0.859735638	0.820448059	0.881722201	0.905646980	0.723836506
NA	0.015192216	0.017745714	0.010651628	0.012494746	0.014062278	0.017172664
NA	0.949161910	0.957642787	0.932961832	0.956158976	0.966837393	0.909386331
NA	0.926875434	0.919482502	0.914230260	0.909238095	0.894624279	0.886039868
NA	0.046256768	0.052826099	0.042472428	0.033367491	0.040630313	0.064488974
NA	0.032791676	0.022130601	0.021309533	0.031540444	0.032283542	0.025569158
TRUE	0.420910825	0.454034286	0.425105053	0.483653871	0.518325448	0.433024596
NA	0.022736936	0.031613533	0.031717252	0.030067821	0.019188239	0.037636770
NA	0.071732992	0.060152248	0.065782491	0.053931760	0.059408309	0.073901980
NA	0.056544618	0.066097004	0.049764854	0.057423351	0.053007374	0.071996956
NA	0.025004649	0.029095395	0.024090604	0.023882808	0.022850120	0.032162187
NA	0.050439757	0.050683426	0.040902964	0.034514805	0.042685327	0.042320610
NA	0.028110716	0.025546675	0.018418390	0.020962987	0.023109328	0.027583728
NA	0.605425244	0.665166347	0.564785003	0.584836384	0.590124496	0.592195687
NA	0.808132705	0.836923925	0.806220707	0.809581804	0.825694099	0.800406565
NA	0.056436071	0.050605785	0.057687876	0.035566622	0.045026830	0.105685081
NA	0.900497632	0.906328137	0.886286499	0.898191188	0.928255141	0.825729584
NA	0.150477791	0.202593119	0.128279025	0.140670413	0.095758563	0.107370413
NA	0.677487220	0.757271506	0.724484104	0.770502538	0.795890497	0.646295640
NA	0.031341203	0.035809023	0.028147293	0.027217907	0.029894069	0.046854450
NA	0.024751646	0.038523905	0.033200756	0.033061606	0.033263817	0.051516062
NA	0.325206088	0.350210222	0.306912914	0.312926694	0.295286014	0.343185625
TRUE	0.627644793	0.602306592	0.597773909	0.602058292	0.670486754	0.536968294
NA	0.700223620	0.742246974	0.683047064	0.678506649	0.761490158	0.750041898
NA	0.033090915	0.021752410	0.032506512	0.031165515	0.024157349	0.034641692

NA		0.049009771	0.044301925	0.038617218	0.038161217	0.045964440	0.049853801
NA		0.426937989	0.433551082	0.383813759	0.411582634	0.412221102	0.385879490
NA		0.027882189	0.035549734	0.026377643	0.030416512	0.027331646	0.028281153
NA		0.017850894	0.019161568	0.015558431	0.019691896	0.013120389	0.028268543
NA		0.477731556	0.504292922	0.426819126	0.491236951	0.500369827	0.505357785
NA		0.599916270	0.662931519	0.558720929	0.624874302	0.612108160	0.586238097
NA		0.028515658	0.027691314	0.041655486	0.026304407	0.028361859	0.042828177
NA		0.718549031	0.750298275	0.619773962	0.671629637	0.691817087	0.650407707
NA		0.507219889	0.466953652	0.451689492	0.374715722	0.370502820	0.358194380
NA		0.627761606	0.612632184	0.592619162	0.475214842	0.566040057	0.483125929
NA		0.061320822	0.058595279	0.052488777	0.065673823	0.067189516	0.059562809
NA		0.573987058	0.592981748	0.568549468	0.555404653	0.618194858	0.489432034
	TRUE	0.700076987	0.729769179	0.648747049	0.690542778	0.759943184	0.677620506
NA		0.667193845	0.718334588	0.659212467	0.678827863	0.706645044	0.777705926
NA		0.110060632	0.128269342	0.095211540	0.128273826	0.098238747	0.121032142
	TRUE	0.166845584	0.202441159	0.159568712	0.141291763	0.151875792	0.206954775
NA		0.045709656	0.043451281	0.036666814	0.043874623	0.031585073	0.047208821
NA		0.241427370	0.268491291	0.197365089	0.273160273	0.254815476	0.244134016
NA		0.337160643	0.351639702	0.310804412	0.260805721	0.251253564	0.256364127
NA		0.910760169	0.925376903	0.887678314	0.886833742	0.888103400	0.876580530
NA		0.131590550	0.131297273	0.139372323	0.159965193	0.185467322	0.162874609
NA		0.036679338	0.051576157	0.028614429	0.038727249	0.036780865	0.077495829
	TRUE	0.112496029	0.121886900	0.084950054	0.083115654	0.069412141	0.083704821
NA		0.025393064	0.034896108	0.019371100	0.022027073	0.019457047	0.030096025
NA		0.492720190	0.545913438	0.482602661	0.544749848	0.612336013	0.629028700
NA		0.735632553	0.753857038	0.658795286	0.699508969	0.689624241	0.748481469
NA		0.802534834	0.886410743	0.851031677	0.786875071	0.786645764	0.785523634
NA		0.031152969	0.027950673	0.034232998	0.034138433	0.030306189	0.049284133
NA		0.028903018	0.039238836	0.039179308	0.037869483	0.055010981	0.039325417
NA		0.034115246	0.026975216	0.022581905	0.033442834	0.020721953	0.038601107
	TRUE	0.468246186	0.528771328	0.411593326	0.429221964	0.552455084	0.434326980
NA		0.057340949	0.049314142	0.044555594	0.040827422	0.033355367	0.055066683
NA		0.040696660	0.028482604	0.047080150	0.036907496	0.030673092	0.049734457
NA		0.062262576	0.067933842	0.048625667	0.057670248	0.057010108	0.074941841
NA		0.038200841	0.031230168	0.043496098	0.042283367	0.042962413	0.048108170
	TRUE	0.074888810	0.071146862	0.074038431	0.068621479	0.076260617	0.063170474
NA		0.034655032	0.028592306	0.024193154	0.030633805	0.016367948	0.035372800
NA		0.074208344	0.081262092	0.051046171	0.058513402	0.067275512	0.071207217
NA		0.586503673	0.649172609	0.578161103	0.572062282	0.621131106	0.577166283
NA		0.236285337	0.269784301	0.198747279	0.207793183	0.241870575	0.237715687
	TRUE	0.055911843	0.058953704	0.044154731	0.057280976	0.059306226	0.060037666
NA		0.035600239	0.043829296	0.033373312	0.036571306	0.031833352	0.044494355
NA		0.579759563	0.583918750	0.495896441	0.497759418	0.535610452	0.561827780
NA		0.033981628	0.034715457	0.026177577	0.031305531	0.042842670	0.040427568

NA		0.022991779	0.023045666	0.022389192	0.018788166	0.019785460	0.031614045
NA		0.530097497	0.564869891	0.497484814	0.562302222	0.640192682	0.567733514
	TRUE	0.881847857	0.904696957	0.889487774	0.876237142	0.904969231	0.854377683
NA		0.019079608	0.023057047	0.020165366	0.027514846	0.015429496	0.020574033
NA		0.383178727	0.347820806	0.332166793	0.240707499	0.308878386	0.205924667
NA		0.296136670	0.385575848	0.293642802	0.342538135	0.415454032	0.434158279
NA		0.169876715	0.180174504	0.131701555	0.172208434	0.179128955	0.158903339
NA		0.043284661	0.026080433	0.025533792	0.023095204	0.024887906	0.030834589
NA		0.055885736	0.063202361	0.050750176	0.050148513	0.052027986	0.060937171
NA		0.487927229	0.466395889	0.339683027	0.425877586	0.466997924	0.428828801
NA		0.276508195	0.352330060	0.310959467	0.242981366	0.361850844	0.373010670
NA		0.206260268	0.302216678	0.197063346	0.172890701	0.144872883	0.180181908
NA		0.876145750	0.919553252	0.894675098	0.866650766	0.876950102	0.843314345
NA		0.027278582	0.042413256	0.024030329	0.027253545	0.027668681	0.030624588
NA		0.414204337	0.439292765	0.372597986	0.386569982	0.408622488	0.379090312
NA		0.923199333	0.764941123	0.273793268	0.876131417	0.897900560	0.772917462
	TRUE	0.048421003	0.042721267	0.028237614	0.049699497	0.044014771	0.042995297
NA		0.032422692	0.030752553	0.032758330	0.040970339	0.024111271	0.048284752
NA		0.342311904	0.358587692	0.271724357	0.293390981	0.299496914	0.272806840
NA		0.040654252	0.037422161	0.030470791	0.036418795	0.049152772	0.038436932
NA		0.024605391	0.030808959	0.018177502	0.024455527	0.022380018	0.029133756
NA		0.014610734	0.014435054	0.018059817	0.016265140	0.013756765	0.022679108
NA		0.642311474	0.637580973	0.589113830	0.466804456	0.571524219	0.448422998
NA		0.044489582	0.045961791	0.054255718	0.048843447	0.042511548	0.056290675
NA		0.561308183	0.618894290	0.722071803	0.659579974	0.648533030	0.619515750
NA		0.094109878	0.055087048	0.110497180	0.059133780	0.083367260	0.059443112
	TRUE	0.042054451	0.045622813	0.024861736	0.031276511	0.033403483	0.033707652
	TRUE	0.482443220	0.482531897	0.454650772	0.449796656	0.470484529	0.429014912

CONT7	CONT8	CONT9	DA1	DA2	DA3	DA4
0.110776065	0.114396724	0.092843854	0.149947167	0.124632411	0.145546398	0.194714794
0.033500195	0.030804227	0.032908044	0.042635775	0.047657667	0.043116180	0.042919521
0.031322938	0.031740319	0.038422260	0.040982443	0.040873383	0.054121923	0.049377746
0.031549301	0.042192778	0.039319249	0.042874359	0.046324172	0.066013349	0.052509741
0.096155815	0.121693584	0.124016578	0.133243674	0.221593741	0.107406482	0.179703778
0.026372220	0.023508480	0.025136442	0.032346345	0.028107800	0.032921027	0.031091255
0.024399136	0.020218005	0.028518757	0.026693745	0.033090733	0.035219408	0.037673999
0.047024712	0.042247837	0.052241577	0.053917705	0.062270057	0.059922257	0.065343144
0.770877412	0.802116306	0.724714659	0.834809798	0.824608679	0.847545198	0.832491475
0.022266157	0.022670678	0.020860744	0.022927441	0.041932456	0.034583483	0.041151267
0.029331109	0.027780792	0.020291503	0.032077658	0.028192166	0.037243456	0.034079373
0.021085691	0.019260214	0.023459128	0.021115199	0.029410248	0.024889050	0.026671333
0.034128805	0.042656968	0.033332591	0.046215748	0.032052646	0.046386532	0.061376396
0.778433141	0.841787662	0.842551567	0.824935309	0.869665123	0.872488707	0.876162610
0.046266115	0.045540290	0.047943018	0.040693760	0.051815162	0.060753323	0.073413446
0.020699256	0.019381286	0.018310939	0.024081842	0.024289348	0.031479724	0.028207531
0.238742105	0.177894588	0.284862853	0.221836123	0.289421288	0.361610417	0.233778968
0.019824504	0.025565234	0.022241708	0.027428108	0.027045977	0.040267898	0.030954700
0.109638655	0.137423091	0.110922793	0.145647127	0.146735395	0.165222561	0.174227740
0.093206495	0.131404274	0.086711607	0.120532598	0.145324286	0.240916759	0.135001832
0.043931737	0.046625852	0.052959594	0.052189649	0.043576184	0.075837656	0.074377928
0.566484171	0.451845343	0.628102141	0.758579994	0.681177458	0.678595686	0.636398532
0.751885110	0.857749906	0.797732591	0.839813304	0.833157104	0.857676411	0.830753113
0.030251071	0.032845783	0.035777871	0.039254611	0.043027387	0.047622222	0.063353968
0.033702149	0.043203141	0.039953084	0.041116559	0.039336183	0.062817532	0.044953656
0.039852368	0.033658139	0.040017686	0.026675308	0.036590373	0.061634572	0.071620520
0.028340177	0.032376413	0.027389951	0.035198064	0.045849048	0.051813080	0.055439644
0.526162482	0.578961993	0.572449800	0.569204044	0.606596082	0.650220410	0.632432221
0.482655429	0.459641394	0.531523141	0.591404953	0.630479698	0.630820074	0.623991531
0.027143738	0.026387904	0.025644415	0.034752785	0.033587090	0.048708076	0.037299485
0.027512205	0.029008182	0.029744551	0.029495813	0.037043343	0.033730667	0.036058732
0.903294209	0.897466005	0.919993297	0.925540661	0.796332743	0.825121333	0.871517453
0.033780680	0.041459293	0.035351479	0.028519268	0.052170723	0.064292795	0.088805635
0.401015092	0.412123473	0.545196274	0.517786792	0.526733578	0.526171385	0.540226767
0.030647623	0.024402705	0.023781415	0.024188401	0.040885146	0.051213714	0.035366299
0.188175809	0.229384614	0.225722486	0.216275853	0.227670289	0.257092434	0.292404130
0.031370041	0.036041868	0.028807599	0.036386369	0.031879175	0.060046822	0.056901155
0.348879994	0.390475744	0.342277844	0.356339816	0.470573384	0.506801416	0.380808268
0.017830533	0.017742792	0.020414153	0.018961368	0.028182807	0.028063288	0.021725764
0.038963805	0.030583605	0.043711986	0.046836031	0.037396845	0.048056360	0.048212786
0.662676117	0.690126987	0.667893810	0.712383090	0.755037230	0.726360655	0.699773157
0.411208115	0.406902501	0.467985570	0.513899814	0.510835213	0.473877107	0.482198226
0.058610653	0.097302365	0.074830645	0.072824797	0.087298369	0.192810226	0.087991301

0.093102595	0.087620814	0.082650685	0.042869521	0.055923257	0.045071750	0.082214258
0.017696151	0.013527336	0.016763305	0.020462102	0.021934055	0.025082685	0.030969469
0.055772671	0.043390224	0.054491390	0.047734359	0.061664151	0.075475154	0.077969289
0.220413222	0.255788167	0.266963862	0.278726864	0.285196197	0.281144411	0.327356088
0.044207564	0.049316342	0.054509108	0.049375222	0.053053942	0.071332626	0.075329860
0.176857325	0.154167357	0.253285612	0.206467775	0.279720140	0.385651499	0.240722525
0.092951791	0.076733788	0.085427290	0.105121483	0.090150608	0.110102928	0.124200479
0.048177310	0.041573314	0.043690778	0.060664279	0.055069698	0.073558911	0.074632259
0.068828705	0.066388479	0.068277471	0.068579869	0.077386134	0.094636193	0.098854518
0.102137244	0.095775283	0.114323936	0.114415238	0.136064704	0.172094805	0.122772419
0.043388657	0.056029689	0.047896874	0.047134936	0.052905115	0.044923200	0.124749604
0.026645116	0.028366494	0.029830811	0.038082259	0.031044896	0.032590391	0.052710291
0.048926477	0.042643208	0.042824286	0.052756397	0.053304311	0.145838631	0.058394899
0.030801567	0.033327689	0.035432377	0.039718616	0.031427817	0.047619313	0.065160068
0.344796612	0.330069168	0.400599267	0.383406669	0.392175724	0.446497718	0.435708460
0.024787463	0.027368761	0.023333057	0.029205732	0.024744417	0.033915895	0.032284557
0.045063948	0.050249692	0.071380705	0.066624769	0.065232390	0.085926673	0.069998034
0.024409203	0.029758121	0.033264202	0.030526657	0.034973837	0.053483949	0.044961027
0.026746577	0.035492236	0.034199072	0.043038631	0.045858124	0.063803524	0.047590421
0.038108957	0.039824670	0.038990729	0.044480810	0.050491194	0.052969355	0.071335386
0.027745612	0.033395002	0.031777973	0.036117730	0.029633636	0.048077252	0.039590853
0.016465705	0.014907568	0.015797455	0.018672230	0.022732508	0.023515148	0.024846924
0.035876068	0.030723829	0.035371514	0.035375458	0.059471579	0.056679959	0.048312744
0.042152618	0.041124789	0.035125910	0.041385317	0.049300525	0.119499293	0.055581460
0.031439110	0.029320257	0.038328986	0.032243528	0.041798449	0.049887259	0.038847210
0.027478125	0.033246658	0.027418022	0.030693181	0.032117030	0.048643259	0.031833832
0.037184996	0.037097742	0.034793763	0.032751890	0.045826141	0.089393488	0.067070229
0.025290740	0.029720882	0.032739465	0.045823482	0.037106554	0.050936817	0.055282341
0.033837680	0.041118295	0.034162759	0.041143249	0.042404881	0.057065435	0.059679615
0.434970769	0.550013217	0.492225523	0.559459375	0.568310967	0.522233954	0.633714270
0.230273608	0.233847772	0.295549967	0.302926922	0.379067637	0.305201993	0.323797266
0.704443529	0.765316746	0.800281238	0.861689566	0.794917384	0.807070533	0.802871525
0.034812173	0.036294479	0.034880813	0.036689100	0.049105552	0.070544107	0.049852802
0.535838909	0.548473619	0.600650889	0.599825595	0.628082073	0.709315523	0.602326813
0.178572959	0.202110578	0.203656058	0.195341131	0.223449477	0.229123138	0.260650104
0.015713292	0.014743384	0.013303877	0.017203267	0.017888586	0.021519601	0.021887092
0.160471998	0.209307733	0.163384735	0.165755420	0.279552136	0.219283066	0.256147872
0.050710877	0.053779242	0.055990026	0.070447266	0.063022758	0.109556014	0.082068664
0.041919684	0.037554979	0.028869671	0.035041968	0.045385911	0.067543268	0.034349164
0.229775403	0.269778047	0.301586559	0.327225299	0.284791497	0.390525493	0.353229870
0.335568346	0.363794427	0.333657481	0.443951841	0.498897567	0.543296042	0.365278705
0.042810486	0.049082072	0.041013715	0.047115543	0.056092865	0.058664882	0.058189463
0.107862478	0.108889671	0.109191717	0.122142054	0.136659201	0.142302993	0.142507605
0.019443308	0.019220443	0.019488077	0.018478710	0.020818637	0.025459194	0.026429870

0.040061894	0.046211152	0.031264928	0.027221383	0.067990389	0.053099483	0.054960728
0.028080264	0.023106003	0.029403488	0.028189933	0.030179936	0.025047125	0.052174389
0.014105217	0.015936684	0.016796427	0.017639803	0.023133101	0.023500510	0.023381465
0.438311534	0.441210944	0.479122608	0.583125517	0.466855928	0.557710148	0.577821467
0.047280563	0.055711277	0.054686961	0.053664393	0.057756272	0.066566030	0.097865089
0.023864680	0.022142913	0.025444214	0.025070384	0.030497714	0.034830927	0.034480903
0.030077261	0.034705054	0.030360575	0.026294228	0.034282666	0.053555581	0.037620402
0.066664001	0.079859884	0.082503424	0.092217063	0.087039919	0.110951681	0.100481486
0.766345168	0.808944475	0.814042106	0.851502826	0.842772076	0.866828056	0.834786994
0.077550765	0.079300844	0.065034950	0.068855224	0.093950721	0.091973140	0.095371173
0.141136746	0.150470173	0.187593750	0.198245114	0.170227953	0.190181269	0.240926234
0.030838296	0.045139877	0.043040177	0.044100681	0.052108673	0.044345888	0.069642836
0.024013884	0.024487641	0.024708760	0.021969133	0.031104536	0.030883437	0.036079104
0.023382267	0.027438424	0.030398903	0.025679396	0.031507948	0.039814142	0.058333410
0.082380755	0.074327686	0.099538974	0.105602471	0.095591955	0.107711002	0.165395348
0.031168876	0.024350571	0.023884674	0.023657191	0.033507259	0.034872354	0.050564366
0.340115355	0.422713268	0.400806943	0.433650649	0.458615720	0.471735038	0.411609842
0.017083253	0.016014117	0.014501882	0.019026032	0.016464908	0.019111473	0.022542046
0.024519926	0.026683676	0.027318155	0.045037907	0.032966034	0.034678551	0.031811640
0.059393552	0.068988232	0.061959383	0.056389468	0.094662207	0.073761751	0.085643870
0.052287378	0.055579048	0.054744112	0.054626526	0.056462533	0.079783188	0.049960563
0.022293570	0.018874278	0.034318346	0.022078567	0.027430607	0.038625771	0.037511570
0.023457461	0.031189005	0.028078544	0.030131960	0.038731451	0.038826570	0.054431431
0.042020709	0.059719547	0.037931827	0.053565727	0.052631064	0.068906904	0.066397019
0.022991708	0.025909158	0.018649959	0.023234232	0.031255906	0.053625867	0.030778796
0.118854160	0.107803339	0.097150134	0.106247113	0.141583786	0.090219275	0.116863786
0.052052868	0.065506722	0.039822484	0.068795822	0.065195128	0.070619486	0.101633676
0.428090584	0.473718998	0.445381822	0.464556319	0.533001609	0.546389290	0.576361970
0.104204178	0.091140007	0.094283179	0.115605817	0.101907544	0.114467996	0.161441874
0.026222621	0.024050159	0.026357410	0.033504600	0.033523120	0.035252310	0.026951852
0.057543184	0.063374576	0.052097156	0.060432435	0.057622665	0.087362128	0.244881477
0.018359175	0.020699986	0.020545537	0.027490178	0.023210940	0.029402826	0.026679788
0.341015983	0.390059346	0.467249694	0.419002016	0.533229749	0.486404011	0.473421093
0.025214959	0.021681566	0.020829834	0.027659157	0.025667086	0.036819209	0.038728777
0.192921359	0.169188247	0.197915135	0.189894120	0.252179201	0.276101418	0.301572327
0.031823348	0.026057400	0.030603632	0.043535515	0.039079528	0.039814934	0.046306502
0.062399923	0.064877158	0.065255816	0.056168527	0.072705592	0.077435551	0.094320647
0.030803026	0.019736373	0.027772070	0.032624028	0.036217354	0.041582270	0.042757706
0.088225085	0.154360494	0.105064071	0.131988149	0.120622831	0.253491394	0.123102693
0.060049613	0.062193715	0.061334203	0.056312188	0.068042899	0.078911889	0.071232718
0.021518699	0.017897061	0.018813150	0.023429132	0.027193461	0.021087432	0.034844214
0.042304764	0.059011365	0.050782925	0.044074797	0.050699442	0.083246076	0.070572636
0.041241680	0.036066413	0.039329620	0.034595753	0.041283007	0.047713909	0.052564813
0.580137760	0.655065198	0.618314450	0.669880565	0.694205989	0.687090443	0.600297999

0.090562522	0.100993826	0.091045535	0.095709422	0.109825570	0.128882053	0.118890232
0.052105135	0.048833522	0.056592804	0.047411241	0.055484059	0.058314977	0.063194480
0.020115514	0.021296176	0.022380458	0.023774744	0.025383497	0.028922095	0.032668289
0.036402331	0.036445472	0.029704888	0.030402998	0.035648688	0.069059242	0.045290959
0.041717328	0.042419043	0.037511965	0.048089741	0.058381530	0.085199830	0.059173837
0.060520922	0.060619700	0.059090012	0.059904100	0.055017319	0.083794293	0.065581488
0.033743230	0.035341780	0.030466824	0.029678857	0.034969099	0.046421955	0.067696794
0.049602774	0.077960631	0.047951312	0.054141494	0.071598495	0.140446542	0.077223101
0.026291923	0.036959073	0.031394220	0.032680407	0.030042345	0.049634235	0.039673906
0.051615663	0.036954431	0.044136868	0.053238132	0.053750908	0.061239895	0.047544721
0.036186609	0.034850411	0.036746204	0.038389366	0.042211110	0.062628862	0.041324403
0.032295447	0.044583372	0.032900346	0.045545588	0.036054051	0.101051581	0.038618457
0.029273221	0.041346567	0.029503860	0.040578599	0.035862938	0.058245437	0.037939444
0.027038652	0.028103192	0.029670202	0.036356913	0.035447004	0.031864028	0.034875381
0.042654122	0.051040768	0.031910828	0.050351561	0.065345745	0.046085869	0.057715444
0.021520540	0.019698043	0.017762387	0.020677017	0.025078271	0.029067115	0.035033597
0.026998051	0.025216740	0.027578958	0.029161412	0.031041658	0.037042601	0.034280773
0.042076100	0.044446712	0.037338266	0.039925376	0.056581992	0.071372833	0.072008240
0.040983159	0.032134767	0.038214881	0.042024127	0.038481798	0.066555003	0.039750925
0.021878917	0.019769344	0.021324995	0.031804691	0.026732594	0.024431873	0.033736997
0.109405103	0.110685957	0.103053333	0.107622870	0.157293696	0.121898595	0.134023297
0.624956459	0.648182320	0.638367196	0.695978855	0.596792052	0.791014615	0.666273775
0.062750430	0.057677235	0.067033425	0.062282576	0.070930086	0.049766182	0.088895607
0.199091297	0.193175066	0.186000686	0.194718203	0.223686754	0.269170600	0.243791486
0.073407572	0.072474764	0.065014275	0.061781367	0.084302053	0.150239382	0.095731679
0.032830083	0.032247890	0.027190002	0.030589098	0.030243879	0.041010874	0.047188071
0.031478343	0.035945806	0.033215572	0.040545111	0.036899502	0.037385156	0.052134571
0.199844081	0.216737615	0.210283402	0.219642403	0.255328636	0.248254176	0.323335854
0.033972866	0.040155473	0.041274343	0.042619756	0.043121010	0.070275143	0.053311426
0.036495798	0.046227288	0.037702693	0.038811629	0.051460641	0.061707597	0.049761620
0.750905709	0.693995672	0.748268367	0.756811320	0.689841151	0.814845911	0.759746703
0.029134785	0.029662474	0.030599910	0.027417725	0.043945475	0.062618541	0.041845958
0.659458319	0.713354258	0.716113419	0.717665678	0.662918480	0.726957201	0.703181259
0.033203401	0.032042741	0.034867046	0.034640512	0.039068628	0.044886085	0.054303253
0.051313115	0.047678577	0.046076614	0.049609605	0.064757315	0.148547521	0.069224955
0.043269900	0.031575416	0.046287965	0.038372756	0.048829536	0.049881202	0.072022444
0.135904562	0.146468582	0.146624427	0.165549973	0.179017016	0.144860564	0.164854814
0.764002610	0.847789271	0.736784359	0.832202440	0.796423745	0.861068657	0.873979358
0.021263694	0.022703182	0.019502896	0.021258391	0.026284552	0.030394527	0.025534547
0.021777949	0.021579922	0.025446648	0.023923260	0.026895358	0.030373298	0.028253641
0.560163657	0.628370677	0.640117480	0.713195461	0.657387878	0.642749777	0.659628476
0.040273264	0.042416975	0.046082660	0.048275865	0.050350048	0.076672278	0.104258781
0.328298686	0.283258496	0.334683565	0.364384845	0.428253385	0.463154907	0.440613124
0.032233072	0.039256137	0.033492853	0.036357699	0.044969832	0.057977976	0.038597989

0.027674307	0.043512136	0.028872836	0.035209450	0.038313404	0.053780294	0.040848855
0.370581619	0.420683497	0.421352303	0.433217377	0.501418755	0.515128667	0.395648526
0.036881417	0.041073248	0.040540735	0.041563575	0.045907099	0.056820748	0.072869892
0.079418143	0.080781372	0.086436914	0.118046349	0.089799255	0.172652518	0.084857526
0.022761733	0.015748515	0.022579311	0.021194955	0.023419444	0.030469539	0.029638753
0.052722169	0.051281492	0.057740933	0.049821930	0.055896044	0.072357988	0.062514759
0.036353338	0.033321871	0.030265951	0.034252290	0.042027023	0.027320951	0.053816535
0.023173160	0.035903515	0.022899833	0.022685059	0.075155976	0.041192621	0.035454378
0.033584019	0.036814339	0.030636886	0.030111932	0.040055789	0.046465307	0.044632111
0.024039670	0.021967433	0.020706200	0.026823409	0.044622288	0.027318761	0.032260153
0.032476492	0.038414358	0.040899256	0.039732408	0.040371268	0.052002069	0.075603994
0.844535137	0.878389213	0.855448617	0.895690156	0.874176520	0.892004922	0.874425344
0.030820040	0.021688522	0.042285917	0.063370200	0.059250570	0.047229262	0.040601755
0.037535519	0.038206262	0.042278635	0.049206736	0.045683433	0.056760114	0.047827587
0.029991493	0.022459851	0.028218031	0.029374400	0.033937696	0.037801937	0.032476318
0.210396992	0.181220632	0.229684277	0.233174823	0.250724945	0.215005982	0.262617075
0.036059232	0.043340456	0.033887987	0.045927817	0.037028729	0.065041428	0.053427000
0.055012724	0.046735989	0.054227288	0.048027090	0.090443028	0.061426279	0.066930252
0.031655966	0.025212467	0.022603731	0.027061822	0.029896287	0.037301313	0.039593952
0.049016531	0.032784480	0.031837860	0.038100492	0.041631435	0.071382693	0.051991561
0.066941439	0.069866848	0.078742636	0.068932042	0.068725544	0.093522059	0.100882981
0.047524962	0.066880934	0.065810644	0.060913085	0.072882999	0.085293742	0.089284041
0.349753386	0.354969919	0.335984886	0.357789185	0.418547016	0.347269524	0.419594479
0.058742775	0.066293220	0.075380505	0.061059129	0.084171401	0.082195737	0.110349313
0.228729779	0.160003255	0.199447958	0.197383675	0.199100795	0.232563333	0.244399584
0.022792096	0.030491673	0.025222377	0.028650796	0.035759362	0.039559961	0.035504799
0.021730852	0.022407737	0.032129696	0.033238237	0.038027808	0.046258418	0.044854298
0.081015714	0.086689719	0.100264779	0.082548608	0.112751835	0.108248184	0.109100433
0.030956092	0.035673406	0.033567869	0.035363924	0.034105325	0.026634518	0.036391576
0.031238851	0.028185290	0.033392511	0.041434684	0.030690804	0.054195422	0.037960792
0.014809558	0.012924887	0.017021754	0.018225757	0.019695070	0.022332275	0.027760361
0.034265030	0.036690028	0.037105855	0.041889679	0.041492631	0.050357896	0.052361257
0.039339445	0.022059124	0.029791470	0.030849706	0.036794954	0.031119659	0.062239882
0.017922955	0.027281195	0.021087746	0.031174330	0.028802521	0.033963050	0.038546554
0.481146808	0.438227942	0.454409340	0.498617799	0.529962491	0.531676914	0.496304178
0.028965961	0.034406862	0.031051061	0.035800088	0.037447689	0.051829366	0.031363630
0.029834303	0.031197358	0.031668444	0.027826313	0.033223001	0.061030728	0.056063131
0.023159237	0.029530141	0.025158953	0.029434193	0.033184901	0.040853618	0.045546464
0.358912228	0.326185798	0.288243647	0.329175891	0.349051833	0.574218638	0.382372632
0.023596807	0.030564125	0.030321884	0.029262955	0.024324639	0.044543806	0.045842143
0.020532931	0.021047090	0.023553877	0.030447722	0.028681542	0.026813103	0.032432151
0.132960855	0.132957298	0.065871810	0.175324460	0.191159657	0.150194190	0.134997218
0.042538659	0.036975236	0.034406880	0.040924262	0.041711701	0.054561613	0.057565350
0.056229878	0.061546095	0.054835652	0.051592391	0.063534077	0.085995006	0.091608366

0.017978096	0.020463759	0.017798165	0.024666089	0.020000712	0.033335323	0.028405787
0.022137178	0.024235360	0.020669472	0.021776319	0.022085685	0.029431589	0.027329789
0.108634235	0.112915620	0.131059727	0.129879178	0.142254341	0.122653047	0.179062561
0.028710220	0.030958847	0.027430036	0.035748282	0.040899748	0.040233975	0.038720598
0.268113161	0.290837600	0.308317986	0.340963586	0.312542829	0.321505344	0.320617581
0.082878067	0.106307846	0.112178338	0.099122553	0.100428597	0.106929996	0.128656612
0.061677220	0.064777453	0.066325510	0.066770903	0.081821394	0.082561626	0.081258124
0.121606977	0.125371588	0.137419442	0.112643461	0.122621736	0.100708950	0.187846754
0.242302584	0.257321298	0.258040451	0.256372119	0.328128002	0.291614481	0.308750698
0.070763491	0.055253768	0.067481653	0.099517627	0.140774316	0.054715397	0.090664452
0.163953336	0.202328973	0.174834404	0.181934557	0.210517271	0.198095745	0.220917967
0.024965211	0.035172476	0.026496168	0.019735773	0.052081059	0.058055398	0.034862170
0.236219551	0.227135096	0.267435290	0.273018712	0.283030661	0.324009375	0.332778822
0.028830282	0.031210909	0.035801732	0.032424390	0.043736631	0.045277103	0.051014157
0.063680431	0.047529344	0.055565881	0.060193361	0.056236963	0.078903880	0.095934445
0.603945041	0.590468033	0.594726563	0.625063960	0.630082497	0.670997084	0.703966678
0.016242357	0.018527302	0.017303304	0.027076747	0.022789907	0.022109160	0.024367435
0.026437746	0.036432397	0.032654550	0.034307552	0.040448375	0.048691564	0.042242920
0.018889149	0.016812559	0.016466229	0.018825542	0.028910550	0.024116715	0.028766974
0.760064577	0.789248654	0.801084764	0.776471944	0.872191811	0.788934766	0.801441823
0.061920595	0.067713681	0.074862989	0.082798804	0.091271897	0.103370907	0.097870177
0.031359949	0.031824042	0.034376666	0.038775322	0.044610548	0.053909864	0.052764531
0.041713778	0.034486529	0.042639171	0.044759266	0.041016272	0.038951994	0.054422298
0.051352536	0.058670200	0.064825799	0.067098165	0.071204652	0.087848491	0.079402401
0.322974037	0.336087004	0.318368035	0.337447999	0.412701198	0.385719954	0.387275423
0.038957869	0.046708630	0.042451813	0.047987853	0.050178521	0.058185893	0.048098160
0.044634430	0.038622606	0.039724955	0.047289965	0.048182286	0.070829854	0.086799735
0.646462156	0.732534422	0.822325816	0.841032129	0.826288670	0.797902902	0.780320759
0.780517253	0.793921941	0.844076004	0.844246181	0.831328303	0.867636902	0.851785181
0.024600480	0.027306456	0.021787562	0.030276620	0.034969748	0.042517056	0.046799012
0.091486383	0.051845025	0.067856168	0.069333724	0.062491560	0.030711528	0.051544926
0.136087589	0.142686373	0.121924117	0.180162764	0.163584289	0.115147211	0.218389462
0.377509581	0.416326230	0.412953232	0.396211168	0.416053000	0.520797601	0.457683364
0.046457439	0.035042050	0.052935325	0.065674282	0.062945024	0.041580310	0.069749811
0.668756357	0.761553798	0.729482789	0.685256696	0.781477822	0.830850458	0.681038687
0.026971402	0.031318910	0.027525850	0.027189152	0.030069513	0.048559703	0.042345424
0.055924629	0.075442706	0.074729329	0.062196225	0.075067711	0.088175104	0.103260313
0.059813084	0.060112739	0.071101239	0.055336899	0.072423409	0.102123726	0.091641261
0.474393541	0.659094088	0.701047345	0.720463775	0.682918214	0.661948267	0.771334842
0.061208253	0.062729626	0.067429775	0.138595554	0.061207212	0.179221324	0.083784995
0.048294240	0.043430587	0.051082952	0.053644485	0.050461949	0.040429638	0.067992381
0.067725871	0.032527315	0.055070886	0.067121010	0.091016506	0.073101971	0.099076785
0.025328286	0.023613039	0.026502805	0.026772875	0.031215594	0.028451907	0.049626832
0.650615892	0.683326365	0.716059670	0.785829179	0.759651492	0.743669755	0.776420948

0.122519486	0.144811919	0.149225975	0.139120082	0.085238352	0.103835020	0.087300888
0.058694367	0.065379227	0.070061247	0.136191879	0.062107165	0.174837603	0.057714530
0.141978715	0.138970905	0.137111182	0.153573670	0.155094029	0.131372384	0.206783720
0.046884549	0.046526456	0.046353240	0.048356802	0.063861763	0.076197351	0.084853337
0.350971655	0.324564475	0.365907699	0.413394868	0.391942513	0.455479794	0.570126312
0.598411939	0.583008753	0.623042943	0.601324014	0.617132508	0.636984144	0.627162009
0.040416875	0.063387761	0.060084980	0.058803421	0.051720039	0.158719192	0.072902024
0.772018842	0.735410192	0.795457033	0.815046034	0.785898563	0.862243786	0.783898520
0.034311277	0.044505892	0.037742917	0.040062296	0.046565341	0.072670875	0.043061094
0.124386246	0.118710025	0.148785036	0.139376036	0.156151977	0.125747713	0.154100966
0.033478170	0.032120419	0.029125374	0.025810067	0.035039002	0.047297508	0.037926035
0.428943388	0.513904725	0.633571969	0.567262396	0.667420503	0.632465709	0.627002986
0.032333256	0.045991333	0.041440417	0.034841035	0.040240667	0.064401078	0.045425837
0.019169522	0.022045292	0.028713616	0.034932311	0.027827722	0.038693586	0.035261131
0.018942161	0.018171699	0.017788872	0.020265607	0.020510246	0.022880278	0.025670365
0.582105090	0.745053320	0.752816195	0.798572011	0.768050994	0.795043136	0.855941832
0.011542996	0.010698524	0.011708538	0.013694985	0.013914713	0.014669760	0.017959966
0.267406044	0.319111912	0.296864119	0.291092684	0.301335603	0.388668223	0.310633494
0.737091176	0.681868711	0.749488313	0.756059853	0.838264231	0.841485020	0.761110070
0.036768008	0.043702666	0.052860889	0.052912801	0.041187024	0.042976809	0.049360086
0.217662887	0.194500787	0.287578732	0.221810692	0.305618486	0.235545322	0.300401378
0.034411618	0.043286815	0.036081572	0.040506442	0.027944223	0.035527284	0.044047477
0.014266255	0.016030137	0.016956433	0.015749748	0.016135769	0.025285251	0.025302950
0.054634478	0.064514430	0.064836819	0.054767956	0.078445383	0.070416460	0.134004617
0.042811743	0.050206720	0.054579518	0.050063664	0.055041143	0.071373530	0.070474203
0.040966975	0.048261665	0.139853079	0.135821901	0.052299490	0.096507277	0.168289787
0.823488417	0.817464922	0.820574041	0.783708111	0.406022707	0.374336524	0.798675999
0.018035998	0.023422596	0.018040165	0.020314081	0.019248194	0.027460829	0.026939952
0.323316308	0.303611138	0.272718798	0.167824273	0.372146894	0.156447492	0.172967092
0.073446495	0.048537795	0.060612402	0.046181300	0.084909105	0.077235324	0.091743751
0.079255866	0.089613314	0.074478133	0.089835557	0.097381115	0.099941678	0.114155662
0.045668490	0.036432891	0.042492020	0.063247577	0.061221356	0.067458356	0.089568332
0.029911786	0.028193542	0.023150277	0.026832933	0.033253214	0.038915455	0.041370754
0.051467765	0.046118402	0.049442958	0.048595208	0.049366757	0.086646248	0.069741031
0.057916608	0.044641949	0.043347767	0.046187891	0.064511524	0.050941659	0.071995445
0.732244314	0.712616076	0.760144646	0.726619711	0.699106069	0.794164781	0.856828234
0.038148984	0.035901045	0.039055312	0.040259042	0.036817796	0.046960874	0.061214683
0.117847379	0.176337470	0.190777111	0.169751371	0.245775489	0.140326287	0.207443581
0.039318662	0.052293441	0.050264063	0.041451839	0.064528892	0.113213691	0.084275636
0.039928565	0.046263838	0.055776792	0.062506347	0.045426060	0.085748962	0.076940701
0.025972257	0.023451902	0.025854102	0.027138259	0.031009142	0.028031462	0.039898809
0.303194842	0.330387374	0.312425423	0.327499443	0.362495990	0.377561471	0.371287944
0.021906079	0.027771483	0.030925149	0.029660126	0.027138862	0.046451663	0.032622438
0.022023796	0.015736696	0.022344175	0.019432518	0.051275971	0.022267637	0.037520643

0.020641710	0.019800727	0.028975821	0.023684534	0.028298060	0.048992216	0.031880272
0.108928981	0.116531450	0.121031719	0.153052773	0.131284410	0.162919761	0.155755879
0.478414260	0.511610088	0.529920847	0.509863161	0.500485123	0.555337514	0.546240088
0.015823841	0.015600374	0.014721494	0.018872973	0.019058070	0.018523910	0.031679970
0.460205330	0.436331946	0.474405310	0.444574248	0.525240354	0.569071734	0.475268509
0.044260491	0.037627184	0.038627876	0.038976784	0.044267011	0.048868298	0.049575368
0.053376569	0.058570532	0.060523362	0.065131905	0.096240963	0.085724013	0.128985428
0.040987812	0.045154444	0.047719360	0.034888553	0.056256882	0.081700424	0.055026627
0.375954386	0.431549136	0.400729057	0.429751921	0.404678640	0.494430275	0.508423520
0.041198547	0.040036074	0.046348495	0.044130806	0.043435868	0.057811575	0.056912508
0.047599071	0.057053704	0.065492426	0.069529727	0.057567866	0.081963570	0.087633509
0.022906771	0.033012461	0.026415610	0.029983689	0.034474337	0.038090515	0.034057118
0.285832739	0.313728412	0.322539492	0.298698807	0.321341638	0.346553282	0.402132288
0.026239363	0.021798673	0.025209981	0.030395463	0.031482836	0.032192160	0.040851304
0.065370379	0.086505430	0.059747937	0.046348798	0.080968307	0.190811415	0.120202337
0.311252420	0.312138253	0.345774399	0.354113740	0.335282846	0.345759241	0.384666673
0.020296983	0.021448199	0.015506085	0.027781098	0.020772299	0.026386331	0.028755960
0.113453787	0.141841504	0.131220697	0.142081037	0.119881482	0.140006408	0.163833935
0.015722899	0.017482489	0.018357946	0.020991474	0.019654307	0.028773665	0.026955704
0.043611388	0.033090120	0.033897419	0.042557280	0.047251981	0.069801415	0.041970471
0.051874521	0.057927347	0.052489437	0.055100194	0.066006834	0.092675007	0.081524206
0.057400572	0.093318275	0.075291363	0.075304320	0.094599832	0.167627124	0.076824343
0.033457455	0.033292804	0.035592501	0.029243976	0.042455985	0.042340637	0.045676355
0.425786584	0.391536171	0.459370833	0.373063636	0.524560555	0.580727786	0.431800477
0.054702842	0.044983817	0.039627531	0.048815777	0.060265855	0.076488709	0.084365271
0.718274248	0.823494762	0.769736783	0.804815251	0.752392253	0.824014029	0.779148045
0.053838288	0.063675525	0.053166187	0.048704438	0.067603287	0.091732108	0.051240224
0.042280590	0.039314633	0.037916739	0.034907162	0.042298782	0.060620078	0.050166585
0.321518060	0.405665700	0.358652723	0.358840458	0.413003726	0.439809140	0.377691134
0.154769293	0.128223462	0.143737379	0.182319302	0.133907725	0.246661710	0.158990420
0.039659288	0.050791554	0.045708329	0.052207907	0.048563172	0.060330428	0.068969688
0.024437769	0.023595042	0.027160659	0.028536181	0.026597940	0.045910719	0.040338170
0.047866598	0.046475106	0.052819374	0.056697304	0.053772836	0.059083956	0.075635716
0.036464627	0.030323039	0.026285297	0.039825517	0.041091859	0.063100446	0.054432377
0.045016640	0.052151362	0.039548270	0.052082586	0.052794507	0.072657930	0.074718978
0.049048944	0.050214343	0.053773644	0.052954851	0.074284335	0.082983170	0.071081596
0.035301786	0.034275671	0.041461530	0.054020373	0.046642986	0.048381206	0.065827529
0.029808521	0.024951803	0.029917483	0.025952346	0.045303111	0.029060373	0.039214931
0.142630464	0.172517613	0.159118933	0.172336213	0.162127428	0.186040822	0.207589548
0.033158063	0.033253759	0.034242178	0.036068737	0.034154883	0.031609849	0.044858512
0.126237216	0.108825609	0.139124193	0.153763268	0.120578362	0.120691259	0.155790283
0.028484709	0.025122841	0.033462015	0.031645525	0.032091319	0.044091131	0.034187810
0.028157119	0.022890286	0.045456886	0.028475280	0.032290231	0.031532990	0.052763390
0.025288430	0.024729754	0.021869456	0.029515087	0.035493348	0.030246590	0.035400315

0.024176746	0.024882712	0.026458579	0.024418165	0.033610390	0.030353755	0.035719396
0.042575364	0.028010802	0.025789009	0.033649898	0.043846512	0.047480455	0.082283730
0.031062393	0.036916776	0.033192046	0.038005839	0.047572381	0.044510111	0.052501159
0.041774682	0.033552772	0.035456113	0.043733279	0.040971178	0.061374698	0.043825858
0.347151138	0.386269737	0.329842595	0.378021393	0.431431980	0.490881807	0.398447727
0.028237768	0.031643353	0.037028544	0.035706828	0.040801211	0.043945195	0.047979603
0.074746401	0.105756104	0.076277849	0.074070570	0.105447630	0.207198060	0.090458023
0.042665911	0.044024798	0.050310653	0.044957890	0.051809357	0.121556255	0.060194538
0.041894477	0.044710239	0.048322083	0.040318512	0.051116412	0.073672026	0.052532743
0.732375462	0.778018055	0.784492670	0.767899840	0.774894989	0.816965566	0.765739622
0.520314409	0.457114001	0.491755371	0.528286904	0.595188463	0.498192154	0.545053830
0.025292694	0.028177545	0.023259798	0.035888936	0.024075207	0.038002149	0.046854457
0.047886752	0.034345668	0.047650356	0.043041521	0.042425462	0.052444139	0.051968942
0.229670722	0.291289763	0.323704972	0.295854458	0.281623353	0.373103160	0.343851169
0.049065087	0.047162364	0.043850665	0.053533403	0.057632684	0.056289167	0.046640756
0.188047572	0.184571544	0.194998598	0.177828975	0.217825572	0.216294465	0.267127434
0.024892874	0.036124158	0.026769118	0.030756306	0.029487707	0.035581237	0.030152987
0.027097481	0.029610583	0.031150430	0.027351394	0.044383309	0.057063232	0.031914322
0.076059534	0.080741502	0.070578247	0.088449309	0.079399483	0.104368686	0.110837908
0.025119430	0.020311878	0.025217050	0.023701265	0.030279553	0.042939043	0.027392923
0.236251978	0.266551675	0.259657888	0.277180866	0.263403480	0.288636161	0.301058671
0.051551017	0.068504122	0.072302262	0.058505540	0.069919365	0.081542925	0.083690934
0.035853641	0.043954013	0.039676463	0.036510074	0.051670732	0.055855374	0.048364944
0.019849252	0.023012247	0.018169767	0.030019109	0.024395152	0.030512734	0.030729851
0.084760336	0.078331454	0.088097549	0.096745132	0.109810136	0.105817069	0.139509352
0.392342667	0.373190369	0.383990020	0.410996509	0.499324349	0.428827611	0.423494121
0.031329411	0.027442659	0.032944851	0.026609294	0.041320479	0.043002361	0.079235438
0.015840969	0.015300536	0.012592079	0.018405599	0.018167145	0.028855785	0.025309848
0.770913112	0.856589465	0.802723188	0.788595240	0.832036001	0.887331466	0.818111825
0.020553649	0.025497499	0.022051813	0.026393111	0.031677149	0.032353836	0.037514826
0.038020396	0.045477339	0.038864429	0.034638611	0.046840389	0.072836882	0.142062608
0.016098658	0.017085397	0.015100153	0.015828641	0.019021567	0.018288865	0.023729442
0.071677885	0.075433530	0.068677380	0.076950776	0.125337017	0.045137757	0.084807213
0.045275171	0.033395242	0.029287817	0.029202288	0.046890045	0.045709656	0.047820687
0.032356623	0.042212169	0.039380295	0.051081250	0.039845072	0.051325655	0.034578676
0.048206758	0.057174547	0.054682150	0.059126569	0.056872873	0.080607524	0.073008909
0.283472993	0.268910670	0.316016637	0.310926665	0.290781214	0.278986778	0.359036390
0.037766999	0.063974927	0.047163883	0.047591603	0.054570891	0.064580402	0.065138198
0.040836112	0.033686100	0.043721258	0.036819264	0.040376001	0.051623904	0.067613116
0.843571091	0.815166296	0.841241972	0.882570942	0.882330518	0.913724544	0.877356278
0.342881316	0.361140011	0.418881282	0.412242988	0.428697321	0.428440819	0.421492925
0.025457534	0.026874654	0.024464263	0.022500572	0.030826529	0.044814439	0.029452749
0.024650020	0.032599698	0.026124952	0.023106587	0.028959021	0.041707886	0.052399321
0.022828694	0.033039961	0.027716363	0.027905573	0.034409598	0.050906671	0.028867167

0.016981064	0.021964432	0.018341120	0.020529880	0.019230320	0.024889325	0.023660489
0.742914861	0.725739851	0.792810458	0.759495885	0.761293362	0.829206434	0.760908006
0.037902129	0.037699565	0.041322231	0.029931425	0.044671684	0.058213434	0.059644522
0.470166705	0.532743547	0.552478949	0.565047788	0.507021024	0.592433725	0.482528491
0.306946763	0.374351053	0.433032159	0.440067862	0.452483159	0.395854854	0.381225323
0.308415632	0.303258133	0.305227038	0.313131200	0.347946437	0.320547329	0.387378410
0.044472984	0.050904797	0.058949863	0.047049929	0.043247961	0.053991458	0.092755873
0.033891173	0.045893054	0.041089805	0.033910913	0.034696644	0.054830902	0.043360158
0.288719467	0.246878168	0.237404014	0.268903635	0.332324698	0.323574038	0.313290428
0.307112137	0.340846945	0.398807229	0.312107241	0.396831132	0.415617055	0.367151980
0.840757390	0.833933618	0.790309117	0.866898656	0.853878554	0.868425967	0.838092921
0.205774072	0.215621256	0.182021641	0.231955165	0.246770251	0.244770746	0.242502441
0.339429971	0.363397987	0.434082897	0.417495632	0.445990144	0.479573861	0.411781289
0.075519392	0.090093370	0.089505659	0.104034509	0.080750006	0.153513119	0.110310582
0.330367987	0.348721626	0.357951329	0.352684526	0.379556864	0.495696807	0.383317101
0.019319664	0.019957201	0.022945989	0.031764142	0.032671540	0.028386557	0.028655625
0.022145471	0.022654962	0.030585532	0.023660646	0.029465224	0.043128522	0.039675091
0.127514970	0.102418498	0.109542280	0.128738457	0.108569649	0.203058113	0.177891102
0.032902064	0.033582385	0.028656826	0.045918715	0.035634908	0.053751853	0.042013714
0.560818049	0.599506017	0.657507569	0.576009423	0.562183115	0.617527251	0.711078487
0.038469286	0.051637263	0.053935152	0.040597746	0.052174312	0.116435759	0.055513838
0.591187836	0.652687160	0.617112589	0.621079244	0.700815329	0.743252041	0.564195413
0.028457879	0.037663824	0.030404778	0.032830837	0.029007249	0.047156865	0.057656779
0.019838178	0.025214521	0.025083106	0.025521015	0.051176256	0.031693885	0.035064087
0.065737881	0.050544663	0.064654949	0.064188022	0.075578176	0.071619943	0.091219819
0.589495091	0.632835255	0.581252868	0.594258992	0.679149265	0.650809460	0.666488990
0.101944002	0.090821532	0.106304400	0.090592570	0.109210284	0.142239160	0.166168494
0.020539967	0.024127758	0.018672653	0.017478066	0.023575871	0.030814497	0.034784350
0.235157603	0.249932000	0.248772595	0.268112086	0.267353572	0.262272838	0.320824105
0.259606307	0.255226279	0.305694377	0.326691321	0.309289877	0.312816959	0.376550931
0.038950620	0.045687290	0.054347058	0.038980140	0.048908800	0.062967740	0.055402838
0.088902228	0.110843554	0.061536604	0.081067617	0.061065424	0.063960248	0.067365054
0.030385586	0.022348408	0.028168459	0.021778681	0.028315161	0.036338116	0.032254564
0.033774368	0.032528385	0.027967286	0.027270786	0.028397607	0.043656060	0.036869920
0.034829320	0.053281259	0.050489440	0.047965269	0.044463069	0.059072476	0.046300614
0.247055981	0.300711134	0.263484777	0.346150147	0.308329430	0.321218778	0.322387122
0.043395125	0.050980898	0.038672773	0.043989952	0.049745646	0.101486721	0.045786179
0.022428306	0.022709440	0.026093446	0.029747893	0.042178690	0.037071745	0.031369342
0.742705483	0.766456310	0.746312009	0.800679106	0.781512444	0.822082269	0.792482214
0.018694383	0.022319524	0.018444971	0.023102081	0.020714303	0.033921501	0.030166462
0.026772653	0.031190391	0.029720595	0.031913622	0.026953308	0.042115757	0.041673393
0.024959061	0.038677571	0.029398040	0.031697823	0.037984357	0.049794654	0.032907408
0.033291815	0.039491767	0.039047926	0.035832139	0.052102030	0.041512333	0.040125531
0.042398783	0.045610218	0.031830707	0.035802351	0.039264760	0.045566686	0.051167438

0.736069962	0.800144276	0.765750462	0.772079393	0.828587124	0.827838602	0.810471945
0.040661787	0.044191073	0.042248020	0.033434489	0.050978915	0.055791675	0.058794196
0.036316393	0.046034075	0.039079102	0.034474585	0.054497865	0.059552573	0.042108104
0.305305298	0.300894361	0.325568264	0.332313602	0.336964386	0.352176079	0.358613260
0.816378979	0.851530497	0.861153535	0.817205920	0.858439360	0.842397283	0.839692347
0.291900909	0.251393206	0.302645984	0.271242471	0.321674928	0.303295594	0.315356183
0.036604205	0.031264500	0.033328345	0.036202210	0.044428110	0.061326268	0.057500060
0.028771294	0.042067391	0.033356757	0.030608589	0.044592830	0.059689564	0.051580944
0.067414796	0.090340680	0.050032931	0.063736016	0.120660114	0.231676781	0.075964595
0.033100574	0.027604573	0.035245592	0.036740606	0.038031728	0.050545010	0.054774538
0.384197109	0.343106917	0.453827291	0.418116510	0.404134814	0.407166760	0.450456284
0.438516096	0.458570617	0.475995581	0.415676457	0.445539930	0.479481654	0.513419794
0.105399317	0.143107921	0.093733955	0.102750158	0.129330738	0.133572257	0.152916314
0.029057779	0.020817894	0.035589157	0.025112657	0.033944399	0.035235176	0.027980497
0.026843028	0.028950114	0.032397220	0.028130058	0.043144708	0.035219411	0.039787121
0.019544643	0.023614725	0.020581861	0.024704376	0.022378059	0.030932115	0.029497829
0.018506750	0.027602409	0.020629849	0.020845986	0.022298957	0.021889151	0.034794540
0.030625813	0.034562890	0.032288883	0.029615184	0.032598384	0.057388507	0.036651556
0.025679596	0.022871578	0.022898891	0.026500372	0.027298362	0.036102878	0.028355246
0.072016664	0.103089163	0.064826216	0.047068088	0.070266081	0.214657160	0.083777041
0.059429738	0.068305904	0.057761932	0.058116642	0.062754573	0.074037713	0.075470181
0.024884133	0.027970271	0.026323355	0.028820447	0.032993018	0.027184124	0.039506131
0.034476234	0.018009430	0.019261690	0.027721050	0.033328864	0.024866532	0.039831502
0.015476554	0.019017797	0.017008282	0.015658158	0.020105065	0.021719294	0.027304558
0.059855836	0.053694286	0.058498517	0.053950978	0.064086773	0.088064277	0.067221084
0.064309927	0.060585774	0.065273599	0.048316590	0.071571299	0.081821136	0.096381919
0.098832231	0.095091682	0.074035513	0.083087827	0.172547646	0.127031980	0.220905067
0.019789999	0.023298970	0.029354720	0.025374994	0.028757602	0.027403538	0.032438158
0.731571862	0.734637232	0.770800045	0.739159356	0.759605865	0.803741313	0.765063669
0.050312141	0.053182789	0.066521633	0.067231475	0.063628050	0.074824499	0.094025553
0.030876304	0.038053848	0.027912068	0.033462608	0.032239427	0.076155285	0.045053820
0.030139752	0.044740535	0.033156004	0.033195539	0.039165380	0.037717536	0.055663948
0.098879918	0.084037555	0.123563897	0.125344066	0.135359842	0.176376502	0.156899899
0.027852752	0.028937017	0.029235593	0.034960248	0.049351089	0.038433918	0.035168888
0.026066384	0.020574916	0.028229695	0.028271059	0.035872233	0.032251952	0.035871603
0.064299757	0.090695161	0.063006599	0.069628294	0.073783284	0.101090705	0.118222040
0.274497660	0.278051797	0.277020186	0.298733634	0.273891714	0.323306421	0.342260515
0.344496895	0.392997800	0.413541860	0.445339448	0.397662739	0.394727864	0.452537044
0.242063852	0.292902628	0.213739085	0.227057379	0.335581402	0.200212551	0.332734044
0.014914255	0.013056960	0.016197500	0.015733520	0.017494336	0.018039359	0.021286230
0.265916132	0.279268590	0.341012949	0.340360063	0.325279420	0.369472315	0.391468180
0.081815015	0.082736238	0.087363041	0.079366210	0.098509393	0.092546961	0.103425315
0.228424529	0.261229071	0.284828411	0.265421050	0.201698110	0.299550694	0.296730241
0.849771670	0.823582383	0.893084143	0.901714622	0.855129776	0.891751881	0.843824984

0.586179599	0.654073651	0.642611797	0.648985282	0.652709537	0.722668449	0.660995188
0.044547288	0.034614694	0.047387924	0.042411074	0.044301634	0.063073818	0.070256261
0.029561507	0.020935671	0.024586792	0.024377130	0.028341670	0.043843173	0.039581691
0.501779617	0.512384787	0.548146839	0.515716956	0.569441835	0.690759619	0.580027106
0.032206031	0.028129189	0.029961668	0.033727513	0.038378418	0.043638438	0.036162325
0.537496155	0.579552707	0.643556588	0.714107139	0.699963888	0.836009434	0.626530149
0.107722991	0.097442793	0.088104586	0.121426841	0.240612748	0.258095211	0.124504226
0.880413577	0.883492006	0.888792604	0.907392030	0.912687747	0.919776409	0.877257778
0.475044097	0.516255029	0.523421582	0.564477314	0.485946059	0.446094773	0.526378293
0.490535814	0.498214813	0.534672144	0.543745515	0.603348251	0.545078424	0.544601614
0.187520352	0.286817573	0.277182703	0.276716756	0.302638571	0.204914811	0.285947448
0.647656603	0.610094954	0.682409829	0.694830091	0.649333407	0.808531950	0.722230324
0.026447917	0.031356585	0.027232291	0.027689091	0.045053912	0.047051796	0.041379857
0.023310864	0.026410797	0.025878259	0.033631479	0.032926558	0.032866385	0.029479912
0.877857890	0.911615719	0.888968953	0.932661925	0.906900094	0.924422213	0.885886351
0.047246344	0.050649376	0.038129660	0.039092825	0.046288037	0.078403372	0.060985129
0.030469477	0.040459277	0.034162431	0.036822222	0.039371142	0.050269618	0.039539982
0.297513927	0.341963738	0.331717884	0.373265891	0.335308214	0.428167741	0.370672697
0.022906058	0.027126315	0.031142533	0.033099227	0.031183814	0.040103741	0.051094552
0.030668825	0.035787193	0.038400285	0.042523227	0.041397138	0.063395500	0.048150485
0.262287197	0.323768577	0.286249703	0.305105592	0.341818915	0.317321754	0.354345760
0.215242333	0.184576015	0.099110026	0.169445175	0.252828913	0.194478021	0.131297090
0.068829119	0.062339968	0.055524481	0.047366624	0.087778028	0.134557330	0.079316112
0.027281219	0.034651243	0.033015555	0.034371685	0.043958157	0.041775847	0.043354308
0.018130734	0.014334451	0.015553109	0.019243306	0.020500982	0.018976258	0.017361686
0.017647847	0.019374239	0.021694602	0.028245117	0.024473149	0.028119989	0.030272718
0.028106219	0.035749299	0.024736431	0.028420174	0.033098708	0.053506557	0.034325539
0.030873690	0.025337417	0.032391986	0.039473948	0.043865851	0.033418062	0.038298609
0.872082997	0.912385916	0.872959492	0.917581317	0.918774660	0.894057328	0.897006332
0.025975898	0.020717372	0.022247458	0.026935599	0.025714430	0.028233709	0.030022306
0.885445488	0.896338062	0.917363341	0.910130632	0.912513958	0.917370846	0.920051034
0.817281678	0.846115864	0.836386986	0.853289427	0.860767925	0.900752643	0.876617500
0.252053467	0.280268756	0.271536586	0.301427173	0.338855794	0.242816241	0.339974017
0.089393503	0.107280076	0.068184537	0.095165495	0.104607735	0.165159066	0.073098591
0.207512357	0.207214907	0.223901420	0.230376156	0.262043238	0.214433085	0.262442150
0.489482884	0.490615670	0.584402751	0.539657402	0.569647632	0.567947334	0.525652957
0.051288645	0.055012145	0.060466724	0.047513717	0.070539764	0.081262900	0.078242447
0.110401316	0.119952174	0.127069064	0.106749244	0.122123709	0.141999024	0.137491541
0.816229542	0.821675046	0.850888746	0.918027367	0.885056827	0.887101618	0.856040456
0.253814135	0.265049375	0.331729467	0.338725555	0.345592720	0.299127864	0.377320849
0.020349628	0.021330271	0.018304060	0.026561937	0.020466688	0.029176551	0.026455285
0.033298746	0.045197357	0.031033080	0.037575722	0.038684513	0.051692971	0.053981275
0.016119491	0.015761641	0.020371883	0.019303829	0.021761523	0.020278227	0.025887966
0.456726283	0.547717955	0.492834329	0.480253803	0.527341188	0.587801249	0.506226953

0.015905779	0.018470139	0.021713581	0.025164353	0.020009845	0.024240862	0.026213993
0.028194855	0.034702091	0.025875818	0.040039447	0.042015911	0.035440155	0.045340004
0.442695796	0.488954967	0.510988952	0.565507112	0.523709786	0.534609344	0.560852370
0.060660239	0.056442245	0.059121144	0.077282519	0.053829608	0.069923398	0.061343454
0.024678628	0.021446081	0.023264785	0.027470132	0.027665284	0.029032413	0.032799100
0.785183161	0.842585645	0.802534246	0.850688300	0.869265384	0.910662296	0.774810111
0.149503970	0.149595701	0.138378894	0.192210178	0.188860137	0.186128300	0.207901234
0.031611738	0.029467013	0.032311717	0.030890563	0.033219426	0.042054229	0.036319167
0.030408244	0.026056446	0.023777382	0.032531054	0.034816884	0.037707011	0.041168262
0.221076458	0.258147163	0.264466662	0.246604196	0.264824221	0.270510174	0.297231483
0.021145151	0.024446560	0.023957152	0.027329659	0.027923654	0.034631708	0.028865277
0.022766479	0.027287966	0.021967544	0.022088558	0.026392426	0.042926861	0.029487767
0.119277191	0.127911137	0.107847706	0.097290177	0.175444853	0.136396348	0.165518918
0.052402091	0.035832258	0.043090116	0.045734329	0.052113358	0.056624035	0.046892984
0.015720211	0.020445855	0.019812019	0.032086624	0.020273150	0.045496084	0.042323385
0.025776815	0.029923623	0.031619792	0.030134962	0.033529183	0.037331670	0.040601066
0.026821770	0.032416008	0.025777737	0.026379849	0.028101624	0.040437292	0.043560257
0.017252633	0.025046476	0.022881880	0.022120606	0.018799783	0.027779196	0.023289381
0.308327181	0.324711302	0.275294984	0.325232011	0.435119993	0.464028615	0.320387606
0.446577903	0.460798459	0.487083907	0.502056688	0.510341462	0.485173532	0.502567816
0.036265290	0.046548532	0.041283345	0.041764331	0.044811357	0.044532922	0.061346912
0.022060952	0.019710316	0.024228965	0.030942969	0.023565397	0.038902681	0.024792032
0.890829567	0.867332752	0.865651240	0.868986471	0.842853287	0.846495204	0.760191087
0.821313133	0.845785976	0.859079904	0.872935588	0.869178214	0.904931476	0.846005510
0.023485758	0.022906980	0.020279620	0.024349977	0.029989130	0.033895534	0.033067165
0.390072685	0.411114329	0.422974693	0.408572217	0.456741992	0.420246096	0.442059302
0.233343542	0.201204090	0.331229427	0.232280244	0.257540377	0.285304784	0.326545255
0.031466076	0.032803173	0.037919068	0.038398723	0.040734402	0.048799594	0.045675183
0.028622635	0.029123057	0.038897423	0.037985147	0.051646600	0.044320948	0.045625649
0.030439631	0.031099938	0.037632562	0.032309532	0.038152537	0.057608715	0.047701537
0.382157480	0.403181001	0.431184990	0.496259337	0.475471260	0.382318516	0.464739104
0.482455274	0.595914878	0.661234163	0.574951653	0.726459020	0.733722932	0.609433962
0.035627402	0.038915207	0.036143701	0.048326515	0.037996515	0.053907588	0.064530106
0.057499138	0.069488854	0.053003529	0.054325954	0.062162049	0.132643779	0.092027053
0.062391148	0.110726995	0.068150221	0.068703591	0.087598385	0.199760885	0.072158849
0.484605282	0.615176672	0.614316165	0.543417735	0.613167019	0.630285128	0.618592257
0.059099334	0.071297882	0.068066387	0.054833996	0.069839457	0.177206913	0.098660803
0.014821763	0.012257582	0.018043258	0.016292187	0.016201481	0.015699210	0.026409112
0.436874033	0.513497109	0.561903686	0.601442588	0.616203035	0.567853784	0.500178450
0.905295900	0.919326708	0.911150413	0.927693747	0.932249362	0.930142325	0.923434031
0.378164233	0.516977552	0.347572990	0.420536216	0.471996535	0.327404036	0.446332692
0.099887515	0.161618202	0.176794714	0.082924212	0.072599437	0.110330165	0.049870477
0.025137208	0.023537735	0.020064427	0.029857075	0.027386120	0.032016418	0.039466458
0.024545274	0.026508127	0.028277348	0.030582469	0.028921563	0.031305984	0.027611923

0.086658450	0.075735806	0.089840695	0.128416653	0.106514396	0.083632103	0.113745272
0.051781159	0.066368006	0.049249151	0.054054948	0.062980864	0.106528647	0.063341454
0.021541281	0.025087025	0.022379988	0.021121766	0.026529588	0.025704065	0.035723961
0.099963318	0.101399171	0.104681399	0.108805363	0.124313491	0.103219227	0.165141197
0.760915746	0.715444036	0.795363643	0.765393668	0.818121597	0.904914052	0.719962907
0.035899315	0.038845548	0.038395244	0.040845782	0.037144398	0.058214896	0.061731655
0.027830790	0.030293189	0.029262010	0.033312744	0.037024289	0.042124518	0.036311086
0.105335757	0.133342802	0.130964520	0.123746392	0.187035935	0.113503517	0.180521284
0.024320862	0.031238255	0.031305940	0.031007081	0.036752680	0.022542688	0.030258762
0.895043002	0.878605103	0.903058315	0.903095352	0.912339355	0.929491451	0.895639750
0.027542040	0.016803483	0.022826876	0.018950810	0.028564483	0.031083714	0.024855689
0.029576746	0.042674317	0.025283583	0.029109686	0.029279916	0.058832447	0.047777442
0.018553462	0.024042123	0.023506789	0.020187026	0.026362721	0.034951575	0.028219562
0.813717741	0.837657031	0.891150784	0.876378080	0.853611089	0.870154602	0.857102953
0.602309125	0.666642073	0.565461065	0.627351411	0.666365862	0.683926159	0.639309627
0.020470300	0.018439575	0.020293474	0.023608276	0.020718234	0.032264120	0.027590069
0.101497606	0.074449206	0.088359055	0.093636108	0.107712474	0.079662564	0.124719122
0.045129559	0.053103249	0.054481423	0.046217398	0.051920685	0.057978221	0.077223579
0.873573496	0.903833531	0.794678970	0.888986424	0.756290781	0.784742077	0.865061383
0.022476352	0.028599588	0.020250440	0.020470868	0.024243855	0.034595533	0.029666116
0.151084518	0.116449943	0.111716071	0.151183552	0.152534780	0.225091789	0.177390504
0.029831876	0.016875237	0.025707343	0.026228872	0.038280297	0.065596934	0.038531541
0.031639257	0.038247740	0.032340347	0.031918719	0.033465157	0.058729996	0.063277447
0.491257077	0.544402465	0.562248247	0.564641350	0.501033164	0.446928989	0.547790578
0.046698546	0.047586734	0.055429123	0.056001358	0.060960771	0.056848420	0.083196230
0.308624118	0.301215035	0.340444745	0.389795709	0.275030686	0.346924233	0.379194649
0.039878796	0.043582150	0.050379691	0.051382403	0.046617368	0.066960322	0.066072774
0.043397030	0.034937239	0.037247495	0.044045785	0.038119333	0.044178691	0.049916283
0.470769090	0.405491375	0.505148348	0.574083912	0.541185352	0.598113473	0.553416106
0.016058210	0.015847857	0.017807172	0.017666181	0.023296122	0.019281407	0.024092494
0.461303510	0.477309067	0.466231232	0.531537122	0.527406667	0.520347641	0.523678782
0.520109748	0.564697474	0.572753169	0.604462488	0.606498655	0.568633700	0.560973372
0.031188078	0.043655217	0.047053991	0.036474700	0.041167052	0.071734216	0.051129821
0.034099764	0.031923181	0.044954188	0.032913148	0.034379761	0.038664596	0.062538355
0.028170015	0.037020929	0.038693863	0.046041014	0.031288352	0.047983033	0.043388753
0.021269324	0.021151380	0.021075657	0.018705647	0.025972949	0.027067793	0.025978219
0.062551103	0.051018961	0.063024423	0.059678021	0.054038142	0.062966009	0.092608569
0.029671031	0.040777082	0.038800013	0.040215448	0.041620008	0.050867403	0.042464849
0.024835886	0.030841611	0.031079595	0.029897781	0.032971686	0.060639908	0.031200279
0.037017959	0.038298236	0.049754300	0.031525528	0.058829683	0.052000509	0.074930355
0.055105459	0.053046549	0.059766795	0.065762173	0.075364596	0.101061532	0.064271235
0.060165568	0.072354707	0.062833686	0.065149420	0.068882887	0.133891185	0.056110866
0.028649001	0.046094301	0.041245372	0.041341828	0.041163064	0.064487453	0.040454705
0.238635189	0.250309601	0.252890002	0.239261595	0.258599266	0.287440619	0.304546205

0.044419138	0.050747591	0.054905018	0.061234137	0.050877198	0.055568256	0.059472475
0.342865139	0.392275218	0.380624647	0.261191988	0.464623984	0.392100861	0.431596869
0.467634968	0.460303977	0.447336962	0.440138731	0.520244393	0.581687452	0.451031061
0.480021647	0.474402993	0.520580655	0.496730145	0.550788249	0.447833585	0.549640646
0.065847116	0.062819776	0.063862291	0.057717402	0.064783190	0.057108706	0.093374849
0.036286225	0.057788938	0.049311158	0.056114808	0.046000156	0.082513797	0.066907450
0.042792748	0.055717237	0.035959119	0.057612207	0.050753697	0.075655185	0.049535447
0.435443240	0.467602298	0.468835544	0.478914099	0.498499115	0.507096736	0.469845554
0.522363687	0.588186670	0.555563742	0.582326615	0.649046257	0.730756164	0.607279244
0.975266713	0.971639727	0.970566204	0.931458658	0.974203677	0.938576025	0.971610269
0.824113697	0.815271631	0.821289539	0.847557016	0.887363492	0.863604100	0.846776136
0.035380096	0.027859721	0.029836153	0.032563521	0.043507851	0.052240567	0.047810895
0.045249769	0.060456986	0.040667722	0.090846321	0.052810601	0.070686521	0.059071219
0.026293388	0.030553433	0.023970079	0.029155843	0.030946122	0.028841832	0.029731189
0.377250955	0.472622177	0.500947596	0.556184502	0.444647435	0.445451463	0.603651477
0.143277948	0.327586772	0.207590283	0.183943361	0.319770642	0.300673947	0.226953451
0.214062863	0.230016069	0.244710786	0.255500846	0.273930451	0.279603370	0.316715431
0.266944794	0.339973640	0.290189288	0.298850664	0.321925142	0.340430177	0.365216223
0.126113148	0.152875937	0.205535035	0.152833842	0.136562394	0.135454610	0.246534606
0.061902320	0.053409029	0.058375226	0.051277283	0.064512453	0.072143566	0.052729132
0.033184921	0.024021501	0.026953696	0.022201482	0.023239622	0.032585674	0.025697481
0.025428297	0.025170505	0.023502853	0.036083588	0.035355923	0.027564426	0.036999068
0.190319954	0.278673735	0.271777979	0.265369432	0.252392249	0.264006481	0.264918055
0.198940624	0.213060245	0.204126734	0.217072694	0.252531924	0.206996573	0.217239809
0.166835875	0.124660182	0.249412007	0.204849568	0.251695087	0.247490499	0.255441165
0.369692276	0.399196097	0.407428013	0.387671305	0.429773113	0.451788251	0.426126762
0.482920261	0.490654760	0.501892607	0.501151552	0.548637593	0.532537559	0.485744498
0.192745898	0.203073817	0.189658567	0.207199624	0.244456392	0.166305598	0.231892017
0.638450178	0.695825222	0.681779302	0.710500593	0.713854483	0.801831885	0.717260335
0.038866321	0.039479057	0.038772824	0.043442640	0.035155587	0.050625731	0.041679434
0.030469662	0.037021008	0.042195609	0.044753988	0.048050087	0.035416500	0.049441211
0.027310395	0.026884050	0.028780151	0.032204224	0.025973646	0.036829330	0.055536947
0.019574365	0.022257263	0.019411394	0.020829276	0.018326505	0.023424153	0.020401646
0.037804983	0.021805103	0.025568725	0.032761870	0.035723987	0.032337584	0.034491395
0.868780296	0.854542083	0.882467645	0.905004468	0.863942574	0.916888525	0.910689988
0.042220886	0.036853583	0.026473455	0.043710622	0.045226396	0.051007570	0.044742029
0.521434624	0.564802176	0.573013468	0.522136014	0.591975885	0.628524830	0.634983691
0.040650678	0.042811974	0.042249735	0.043552899	0.030998217	0.055689031	0.086583327
0.061268129	0.061454590	0.071745151	0.067404267	0.069481069	0.080607085	0.087664480
0.021198547	0.022589339	0.017016713	0.019593163	0.020250383	0.028446660	0.024219694
0.265735142	0.315861464	0.299510986	0.355444151	0.383444530	0.384807489	0.310568121
0.229754177	0.335899258	0.277560041	0.241772953	0.361552023	0.272430109	0.356132226
0.817011062	0.763136791	0.857841934	0.845344144	0.836204398	0.826768916	0.862904149
0.019598438	0.032415579	0.026565594	0.025305107	0.031072514	0.028632656	0.040573359

0.042906055	0.056132445	0.043919400	0.048830911	0.056775441	0.133530837	0.051701543
0.047708150	0.047902974	0.054277790	0.043512970	0.044402390	0.082064331	0.070753107
0.020383809	0.018670778	0.017508868	0.019443863	0.026686876	0.025893409	0.035725409
0.647677133	0.595098071	0.513916327	0.568790530	0.710458077	0.734642243	0.697624035
0.031474804	0.038992459	0.043505527	0.038730721	0.048791984	0.116116113	0.040417020
0.034668745	0.036990787	0.048241644	0.040417910	0.049777076	0.041985689	0.043658893
0.945699652	0.930278835	0.950057308	0.946402306	0.945592412	0.863341822	0.912629538
0.049478404	0.043352874	0.039951635	0.041509864	0.055874931	0.046907014	0.081061298
0.074506629	0.088480473	0.070133166	0.058623258	0.082267556	0.191243293	0.103565691
0.214508859	0.209425779	0.235370971	0.217251989	0.229079509	0.265916353	0.284996422
0.056857817	0.073970198	0.053978106	0.048180260	0.046515103	0.078717061	0.087033507
0.480624641	0.500719985	0.533354831	0.502941310	0.504655202	0.580106723	0.532991621
0.183034950	0.194189035	0.209333140	0.204082013	0.265696423	0.158679286	0.242475905
0.036929054	0.029873058	0.036199319	0.044732484	0.062894012	0.045832980	0.123183325
0.659252644	0.705649963	0.676209115	0.676155298	0.692648032	0.764145700	0.696909988
0.052499142	0.048485461	0.030105603	0.053790388	0.066232930	0.070267389	0.090090146
0.971521737	0.970390591	0.974034308	0.936675355	0.975853136	0.915677678	0.969402220
0.527637048	0.391861620	0.273191675	0.451858292	0.514886689	0.335012355	0.346629954
0.027662069	0.032937256	0.024010443	0.032133012	0.033035893	0.058967576	0.042814682
0.051392043	0.082208297	0.047094105	0.062970951	0.063430816	0.066642494	0.083539825
0.039516305	0.040976661	0.037266802	0.032701890	0.045364102	0.100350938	0.042641158
0.026407135	0.033393954	0.024745219	0.029266348	0.026200219	0.040485486	0.032150841
0.119870322	0.135451334	0.127096371	0.134942074	0.129701548	0.153152441	0.127659788
0.089597752	0.092308919	0.102168321	0.096225469	0.102779726	0.136228579	0.121216935
0.214446832	0.402154351	0.508395893	0.421767244	0.456280882	0.320520683	0.411075655
0.043536080	0.038343752	0.045327672	0.036224897	0.037450424	0.039566444	0.063698302
0.095971526	0.151305437	0.124678025	0.090795434	0.169782426	0.275386859	0.160183445
0.789390595	0.671372935	0.792083237	0.804115041	0.796613203	0.705113002	0.823500980
0.026693396	0.027868585	0.029316666	0.029033979	0.025223855	0.037784807	0.033782225
0.174149030	0.150325587	0.176213510	0.158797610	0.196371128	0.207020881	0.198726219
0.511756778	0.520895744	0.577053073	0.538540883	0.558783754	0.632712477	0.509668250
0.161871125	0.150216541	0.166991208	0.178587198	0.200863523	0.223194685	0.240136145
0.730211324	0.769011045	0.731403013	0.743729200	0.813435629	0.768025615	0.774055233
0.519612577	0.647253292	0.541263666	0.627648125	0.537980517	0.667007425	0.585651503
0.502158737	0.502951452	0.505974893	0.524891685	0.499350810	0.485435785	0.563377292
0.113355162	0.103978248	0.151030856	0.118091012	0.115566494	0.124043892	0.136349660
0.021267129	0.030592981	0.034657441	0.037747811	0.049956041	0.057870909	0.037274938
0.042821647	0.046819235	0.041776430	0.050204064	0.042406552	0.053181967	0.055220171
0.828848055	0.856736204	0.824330340	0.836982800	0.845662090	0.841884066	0.841270734
0.481466198	0.532930645	0.505446094	0.540275926	0.529066251	0.598040645	0.502177853
0.040243578	0.040866947	0.037786289	0.032347698	0.060836816	0.060505829	0.061510362
0.512573720	0.566876462	0.544437780	0.594816263	0.530524571	0.582375350	0.555907219
0.046753447	0.053550935	0.042626103	0.040132426	0.050521925	0.117111546	0.055668728
0.053572008	0.052901897	0.040917914	0.036022414	0.090132475	0.066027459	0.157781917

0.019957336	0.022124917	0.029831467	0.032265305	0.026092713	0.039494702	0.027622480
0.295885264	0.329900553	0.320474510	0.317451519	0.345890208	0.397954512	0.385467899
0.033869610	0.051450691	0.034062003	0.032667990	0.049667809	0.060394930	0.060025255
0.521939022	0.502720984	0.546908161	0.531501563	0.579892665	0.638080560	0.576372133
0.031059045	0.031369039	0.027997497	0.035860649	0.041801527	0.048575235	0.043235144
0.297940780	0.333832187	0.309353780	0.338580576	0.323122974	0.323317417	0.377707379
0.051441808	0.056835074	0.061382337	0.058014159	0.061014965	0.072243634	0.077668725
0.834666765	0.844945175	0.721427841	0.827409707	0.885382277	0.861091328	0.834209488
0.022508160	0.017942569	0.021451723	0.020348363	0.024726490	0.025961936	0.033666966
0.512517776	0.535147897	0.550231136	0.518883225	0.671448142	0.736163314	0.518817836
0.530126276	0.501934281	0.498767800	0.500343097	0.605480699	0.469112824	0.590081370
0.022948674	0.021792394	0.020785708	0.023784845	0.021479513	0.024859824	0.029959423
0.020629676	0.023909772	0.018407923	0.021870797	0.024360443	0.034741722	0.035968351
0.022467243	0.020545621	0.022218446	0.027973313	0.025943578	0.032743351	0.044602666
0.407142745	0.556577429	0.480862703	0.491953176	0.547551562	0.466816610	0.583491264
0.028724312	0.033288482	0.030056923	0.031980858	0.030028115	0.044983295	0.041719489
0.707103289	0.785230474	0.716747238	0.773078438	0.756607350	0.765553598	0.731447105
0.437048402	0.394984491	0.430413265	0.499787815	0.444812304	0.443359962	0.455043182
0.556651499	0.621615623	0.613040819	0.538552780	0.753762529	0.858229379	0.583151326
0.021199126	0.025631737	0.024694827	0.021707671	0.020844611	0.033621255	0.028292145
0.018781184	0.021702794	0.016893648	0.017284024	0.018837865	0.032374361	0.025474492
0.260921700	0.283001157	0.291718012	0.249075109	0.279282063	0.326830232	0.318349493
0.376846907	0.392510695	0.412387121	0.411432951	0.481598109	0.409284888	0.455680163
0.026256417	0.040645974	0.031691965	0.031803248	0.037327087	0.056941238	0.037396489
0.153947093	0.171430069	0.217972680	0.181552097	0.188860224	0.175252275	0.294344408
0.710899747	0.795111543	0.745304706	0.787211084	0.801298163	0.870986405	0.809212549
0.025092027	0.036432347	0.026536833	0.030291706	0.035487948	0.115026613	0.042375601
0.114916076	0.127088597	0.097640677	0.131565527	0.191619311	0.144638161	0.195126659
0.197440546	0.204928506	0.235652349	0.253106434	0.249684464	0.202020004	0.342811303
0.019383535	0.017717328	0.019296225	0.028436165	0.018566733	0.032383537	0.023015434
0.014672300	0.013705174	0.014600705	0.014776713	0.016368761	0.020307318	0.018540009
0.047242593	0.046479390	0.059096168	0.047709970	0.050855486	0.073729325	0.080498725
0.030631342	0.026520615	0.028232873	0.029311677	0.035118724	0.023363622	0.038436077
0.021492593	0.025123362	0.026952178	0.022398968	0.028869369	0.037683070	0.032560995
0.046158918	0.065854173	0.051604403	0.056908379	0.049586689	0.057885370	0.059670341
0.030350339	0.034671961	0.038538497	0.028313138	0.033266414	0.042367594	0.045483460
0.811116290	0.805743277	0.835190731	0.836146819	0.837889817	0.881769147	0.863007343
0.013821404	0.013893395	0.017719833	0.017698774	0.018191874	0.021990303	0.021710962
0.830862521	0.816602534	0.842567469	0.839032284	0.782337473	0.729434212	0.799887799
0.049519360	0.037002100	0.037569471	0.041080960	0.037893309	0.055727724	0.057079702
0.026479150	0.027115042	0.026396599	0.026180519	0.028734905	0.035074844	0.036570159
0.034972400	0.045301642	0.035130461	0.042329001	0.044509234	0.049304358	0.039117330
0.027578937	0.030796691	0.032106200	0.030648630	0.032671214	0.033671078	0.027814271
0.033576437	0.035074729	0.027785178	0.036856954	0.045948485	0.046503387	0.041938296

0.644075694	0.659676123	0.681325261	0.631790620	0.664768454	0.797868039	0.705863590
0.525364989	0.498367396	0.527705099	0.541583780	0.578695821	0.609347909	0.521011898
0.426031663	0.489318313	0.459952374	0.452732950	0.487290478	0.586970724	0.435352892
0.017650369	0.021138137	0.017638498	0.019471047	0.017446755	0.030975450	0.024125942
0.501640586	0.565476582	0.538183651	0.553563743	0.551276393	0.630621432	0.553573802
0.046176777	0.043137213	0.048091057	0.054339554	0.061453762	0.077528160	0.056946488
0.700287392	0.605922011	0.709849786	0.685994341	0.714376452	0.716501188	0.699103552
0.051879680	0.056264661	0.067026728	0.058218681	0.054580101	0.054086224	0.076975948
0.573704445	0.585106422	0.601880880	0.653420893	0.627809059	0.629206108	0.611132575
0.792386208	0.747096595	0.798222138	0.829641842	0.798569026	0.837618679	0.763456277
0.069369278	0.070332928	0.073694133	0.073592279	0.079068250	0.092402217	0.104130290
0.403019282	0.373835104	0.415989557	0.422483805	0.439645307	0.478217277	0.421953916
0.015219736	0.020167997	0.013649544	0.022251791	0.016699227	0.022411156	0.026120806
0.082673838	0.073513290	0.108045545	0.164683482	0.269810602	0.150471798	0.202601150
0.025551488	0.026983872	0.028353701	0.034412507	0.038170130	0.053770113	0.036925946
0.586920584	0.592082277	0.604428852	0.641599828	0.689155892	0.752892088	0.575915990
0.070604023	0.130612888	0.076752239	0.071971699	0.287914902	0.093100754	0.129617452
0.034647794	0.030190061	0.036192951	0.046677144	0.047190533	0.054237997	0.029401335
0.020943859	0.042194709	0.030902371	0.026129761	0.035728328	0.038740325	0.038313536
0.619768370	0.685849332	0.659604667	0.653707823	0.695675860	0.762782168	0.699178236
0.076977286	0.069436452	0.070276625	0.060819360	0.079503426	0.082928509	0.074856055
0.041473481	0.048637190	0.050061824	0.045519492	0.048346380	0.060342260	0.057853848
0.602661089	0.644546538	0.628847414	0.616143208	0.673795063	0.717070488	0.608560059
0.030601565	0.900453841	0.880540245	0.862912442	0.884948623	0.904137992	0.868910753
0.835559688	0.862295293	0.867264726	0.880228390	0.871765870	0.880050707	0.855761478
0.052478257	0.062228166	0.056092215	0.052758737	0.084882236	0.103461121	0.133806886
0.590479916	0.634882619	0.724177924	0.722660531	0.718981787	0.776394523	0.678811295
0.037058607	0.046521893	0.031111503	0.037465130	0.044594606	0.057501647	0.036095601
0.044064241	0.044574922	0.044077363	0.044434954	0.040975449	0.078847409	0.048320406
0.045164661	0.047013695	0.046404007	0.044850781	0.052517265	0.107292931	0.126843354
0.028102546	0.019303993	0.030177451	0.027745664	0.033867414	0.052198373	0.038857251
0.029469243	0.024567605	0.031545852	0.026980143	0.022080832	0.035621258	0.041729269
0.033963206	0.050828929	0.035961970	0.034541929	0.042507384	0.059529565	0.069661161
0.072554909	0.106479041	0.092076007	0.105656853	0.069920410	0.053501484	0.100107127
0.017133199	0.013287586	0.019520049	0.014731998	0.021793779	0.023596686	0.021400406
0.411584811	0.457466995	0.440403476	0.417674517	0.499658227	0.496472448	0.462599323
0.052239669	0.070479429	0.063423541	0.048844190	0.104040442	0.084184506	0.113509248
0.050540087	0.040567191	0.046163624	0.038906914	0.053853470	0.069922228	0.077438040
0.035098663	0.041411505	0.044873224	0.067714045	0.053897395	0.043400157	0.109002402
0.159046302	0.163343699	0.215451758	0.237975538	0.201627689	0.212998383	0.266782673
0.073603390	0.057952555	0.067488898	0.073501395	0.067664226	0.075464264	0.098237793
0.139530696	0.172334536	0.131612202	0.122145009	0.248424213	0.141939769	0.216262000
0.650896710	0.760188353	0.717397634	0.728641057	0.699040053	0.800681535	0.696253472
0.173318965	0.157818318	0.102023261	0.196292239	0.209553412	0.194893770	0.188634982

0.724177990	0.708824630	0.805444070	0.748108450	0.734341687	0.776171379	0.837484951
0.027246838	0.028913285	0.024406362	0.027813870	0.032701400	0.035359953	0.043147073
0.487361004	0.453493954	0.508841442	0.483777859	0.483964423	0.508378334	0.533710486
0.484278308	0.530479687	0.516935813	0.519797434	0.566188408	0.624671134	0.565862042
0.049223338	0.055312895	0.053656003	0.049827085	0.052359415	0.060860436	0.095970884
0.023331795	0.030677662	0.025957940	0.031635308	0.023038740	0.037922542	0.042949774
0.758957330	0.813346160	0.745333622	0.799516932	0.773782950	0.855578447	0.765241242
0.039335441	0.037135670	0.037299325	0.037120938	0.057169441	0.050998522	0.052941877
0.539292769	0.574615184	0.636829551	0.547157259	0.555014495	0.567658342	0.547540698
0.017054250	0.024429733	0.020625477	0.020442974	0.018951177	0.030283711	0.041698167
0.092258767	0.117592901	0.072394212	0.075136162	0.144656110	0.118117328	0.102266588
0.495189077	0.459445553	0.536564300	0.536306794	0.512892744	0.616905528	0.545032441
0.317224613	0.349131091	0.347862645	0.355838174	0.364890922	0.431953269	0.375147066
0.198607979	0.202811802	0.179910060	0.187899066	0.235206456	0.207243473	0.292449219
0.064005761	0.060436126	0.048431235	0.039823870	0.085057017	0.138526042	0.064634688
0.628069983	0.638732622	0.653041738	0.636444938	0.627342631	0.643421525	0.662602511
0.163906979	0.103968572	0.140802076	0.139119921	0.149894456	0.126391032	0.183246463
0.481276145	0.525892750	0.528169702	0.497526059	0.505740523	0.612615227	0.561483718
0.371524546	0.382669428	0.419482374	0.420504500	0.434192167	0.407089071	0.414269857
0.023060932	0.032910282	0.024543355	0.024231826	0.025693441	0.044103649	0.027443288
0.598681490	0.528182236	0.593366655	0.681646792	0.684931266	0.621952978	0.703036016
0.104839157	0.117237464	0.159303877	0.157305866	0.131908497	0.163510508	0.173668878
0.069791844	0.068036772	0.066211878	0.060557246	0.060103079	0.087121285	0.126834879
0.025194750	0.028906089	0.022772184	0.018994982	0.023719201	0.036092232	0.037624323
0.950080608	0.933565188	0.931882770	0.897903939	0.928946489	0.936034122	0.925625984
0.036092698	0.043590554	0.033975285	0.048322486	0.044006025	0.057631943	0.046151413
0.029840935	0.027681858	0.030659980	0.029414585	0.028050467	0.039145272	0.039641413
0.917703219	0.934951144	0.905844051	0.928829176	0.935604362	0.928986513	0.917838233
0.920130737	0.896780571	0.921464312	0.875361474	0.863698510	0.804850381	0.844651851
0.845409043	0.871826537	0.866482849	0.870571762	0.929912296	0.920668865	0.866295460
0.778604739	0.817814514	0.828605746	0.817048364	0.823232676	0.801834620	0.867451347
0.039043298	0.044652660	0.049705871	0.053848263	0.056590292	0.068822183	0.059113444
0.018395121	0.030869493	0.033564671	0.026160980	0.027071310	0.033491418	0.032814279
0.412188740	0.525573979	0.542271193	0.528126147	0.532030281	0.615266929	0.522644048
0.514424701	0.538795240	0.493167178	0.532010024	0.561859476	0.507726838	0.545899588
0.042686570	0.044375774	0.041103151	0.051805007	0.040574357	0.053860564	0.052869470
0.421177782	0.385761747	0.458407368	0.426082456	0.437106643	0.475554988	0.457801695
0.029397510	0.032549000	0.033783381	0.024634092	0.040084895	0.047338818	0.042835513
0.060635699	0.074905372	0.077056940	0.066658476	0.081326106	0.079482392	0.118432559
0.396127078	0.435135557	0.482658087	0.465755184	0.474268561	0.457851023	0.460311571
0.027173926	0.026387371	0.025836675	0.033107469	0.026000226	0.038303501	0.035650403
0.058259479	0.068047507	0.052232932	0.063457304	0.068820461	0.071297757	0.074065160
0.244719893	0.304301536	0.254922953	0.370863254	0.328644191	0.381703525	0.290774533
0.881738624	0.920382987	0.873723194	0.888820577	0.913941821	0.934833092	0.878406546

0.049956913	0.060919426	0.054553989	0.051307683	0.064184085	0.080706805	0.055756631
0.268470499	0.280720259	0.241528538	0.260742845	0.361823023	0.365791089	0.349399527
0.034707806	0.026122103	0.031937502	0.029991473	0.037378741	0.042662666	0.040406144
0.379876605	0.484599993	0.397577538	0.461389453	0.525578128	0.555135337	0.457516271
0.029497435	0.037848994	0.036785883	0.037865479	0.030830570	0.059899284	0.035580707
0.267509499	0.311099571	0.306995096	0.317962426	0.338238800	0.385283249	0.381044796
0.294327011	0.339793705	0.380659685	0.386007876	0.465736387	0.338021963	0.388757603
0.035650149	0.035079968	0.037027023	0.037704598	0.046282622	0.039880122	0.042662185
0.101246448	0.103027403	0.114463552	0.117477709	0.109117621	0.140232828	0.162775500
0.028887893	0.028587357	0.032760680	0.029238250	0.043049819	0.049237212	0.036058864
0.022212771	0.026460964	0.028215914	0.034574174	0.037914514	0.041212065	0.031626396
0.036409445	0.040667847	0.051341095	0.047579367	0.047860749	0.057632056	0.064142315
0.085568636	0.078578370	0.074301973	0.091989910	0.116045577	0.188298041	0.104210381
0.892223743	0.869738350	0.900169778	0.896012362	0.897152841	0.801873803	0.837774855
0.051429623	0.054597390	0.058591860	0.049116180	0.077336584	0.072255481	0.046612827
0.032916576	0.043516207	0.030615703	0.039712703	0.030087304	0.048395977	0.038079657
0.968763113	0.954241173	0.968545879	0.967449183	0.963178633	0.928093132	0.952884815
0.050873922	0.058063651	0.056156720	0.061791821	0.067966335	0.068235789	0.059395762
0.032987043	0.030720421	0.039116416	0.037066789	0.029994669	0.044359074	0.046886695
0.914440223	0.694615925	0.896434738	0.945470616	0.929150141	0.935125310	0.859520577
0.036015554	0.033182389	0.036576070	0.026850521	0.031023993	0.052473874	0.048118250
0.048884125	0.043936451	0.050487101	0.046431223	0.048389407	0.064639467	0.064930810
0.920366250	0.910043240	0.915703130	0.932643931	0.924360982	0.802856082	0.874650482
0.042913539	0.032533737	0.037166357	0.049091918	0.031569555	0.054624751	0.045045476
0.605564004	0.672703847	0.643546646	0.623594606	0.632297238	0.611506573	0.587521148
0.412928707	0.415332227	0.472024609	0.456081577	0.475893544	0.484056638	0.456625748
0.029088411	0.027032148	0.035758858	0.035672779	0.022657233	0.048293168	0.044813808
0.026001945	0.023869808	0.022337358	0.020613369	0.024185737	0.025403905	0.033549773
0.462512077	0.482315141	0.523006504	0.432375695	0.527437863	0.511404529	0.488333956
0.061152484	0.068184347	0.058156811	0.056059649	0.079277289	0.104962090	0.078352179
0.052307990	0.061438933	0.063382809	0.049914016	0.067531142	0.082617327	0.094419939
0.044970219	0.045982105	0.066528321	0.054185697	0.064136613	0.043882136	0.078157335
0.045485608	0.043845918	0.041882391	0.048202142	0.039899801	0.062970203	0.044571609
0.453840834	0.498339572	0.446082440	0.455712888	0.475820430	0.515085500	0.455349795
0.017180364	0.022416535	0.023910202	0.025485287	0.028286581	0.024098296	0.033891294
0.021805588	0.021494429	0.020465763	0.023440570	0.029589705	0.028784334	0.028898042
0.814420775	0.829897870	0.818089157	0.798709518	0.877896885	0.864951747	0.797944040
0.161463529	0.184260832	0.165064203	0.137381108	0.107257949	0.033438120	0.034902969
0.023397828	0.020940725	0.026674919	0.029269255	0.032362350	0.033620013	0.031385342
0.076748650	0.089386987	0.068365840	0.063126912	0.064278609	0.172130090	0.112208293
0.059407534	0.063410448	0.054723329	0.051630040	0.062147550	0.086102804	0.082584056
0.192991142	0.173483308	0.153892117	0.202236835	0.166471904	0.309314356	0.188782063
0.684866723	0.595053776	0.503813649	0.537457218	0.603121013	0.379506124	0.387649412
0.814434319	0.843101236	0.850659142	0.792328050	0.824948107	0.831250812	0.854162727

0.023993074	0.027390883	0.020107610	0.023909185	0.025277826	0.034815203	0.037806276
0.027780592	0.027522849	0.023199627	0.038846457	0.041673748	0.039455181	0.046724712
0.544853520	0.565193195	0.545763827	0.563531139	0.585300727	0.634330483	0.562310746
0.766334806	0.742047827	0.754580237	0.778647872	0.761745497	0.735988725	0.762072354
0.806015351	0.822093128	0.833717185	0.834229387	0.823191559	0.873769548	0.792190899
0.017779406	0.015399619	0.016551036	0.019012906	0.021109491	0.027477689	0.028764341
0.916706414	0.767979167	0.845664938	0.823519304	0.684695772	0.786445769	0.878173451
0.047666846	0.048827557	0.055126778	0.036078569	0.038924711	0.071831696	0.060222929
0.042557820	0.054040667	0.037888318	0.048949182	0.116389848	0.068136155	0.067874733
0.768348770	0.824860020	0.761488082	0.830395719	0.784261064	0.813296305	0.854996529
0.127057269	0.131701389	0.152014371	0.129375207	0.141140162	0.139165504	0.171963438
0.511777858	0.546573232	0.526228407	0.517072090	0.534682363	0.591582477	0.556847388
0.038505660	0.043281261	0.042310508	0.046583881	0.045298367	0.051894223	0.062692046
0.500296819	0.541743860	0.493328567	0.540432110	0.574846514	0.537425792	0.536671525
0.660457796	0.701353743	0.703065925	0.703720917	0.718646381	0.714245455	0.696192208
0.409139845	0.375820374	0.422773797	0.416605232	0.425966863	0.405248734	0.467819463
0.786391045	0.830731355	0.843457017	0.842367362	0.857960176	0.853915171	0.865364509
0.045931565	0.030978669	0.053382102	0.044412937	0.048100764	0.052247791	0.078045943
0.843931097	0.833221682	0.859428947	0.867338734	0.861239274	0.887961612	0.873925664
0.251274002	0.235887368	0.261128993	0.331040794	0.319835544	0.251512094	0.316380610
0.055898107	0.072780582	0.066253836	0.055432155	0.075999228	0.149150321	0.057014184
0.132847281	0.120379238	0.144159186	0.124744545	0.132302113	0.101242590	0.167324929
0.029611845	0.026954445	0.028761112	0.031617924	0.025133784	0.044011649	0.032197975
0.019982411	0.018266741	0.024465249	0.021674517	0.023420010	0.024127911	0.026695396
0.914499743	0.896388247	0.916926826	0.915915165	0.875909623	0.927669334	0.903339894
0.043766638	0.058089685	0.049927582	0.054336339	0.062300972	0.044174303	0.062020639
0.842612229	0.843271455	0.893939702	0.879863910	0.879630413	0.903413784	0.891189635
0.058701366	0.063892466	0.071553471	0.082072601	0.070742871	0.081471644	0.066825756
0.020974823	0.021065132	0.022731781	0.024492447	0.020330353	0.030478041	0.034979122
0.040456980	0.026049729	0.045078146	0.031530085	0.055335713	0.054784879	0.059047991
0.379530032	0.400661325	0.352999120	0.376421596	0.457869498	0.561647159	0.385457082
0.072193362	0.073067380	0.054849572	0.048442930	0.049700233	0.151540236	0.120487071
0.862987889	0.873867412	0.910324960	0.901759399	0.886842281	0.898613281	0.898437022
0.024586415	0.025837752	0.023250725	0.023433390	0.026012643	0.032721204	0.023170242
0.019706549	0.033350495	0.026128630	0.025519062	0.024566886	0.028427982	0.029315396
0.862489013	0.859628743	0.847420761	0.882505984	0.853284616	0.870520336	0.858535273
0.066357072	0.058392583	0.057219867	0.067222202	0.070388924	0.091278046	0.086140382
0.578769960	0.637549496	0.583512563	0.585634612	0.611082513	0.581850537	0.642501381
0.077140562	0.075781293	0.078687684	0.064349835	0.103929751	0.082345516	0.119741643
0.291967367	0.394612786	0.357725622	0.394041590	0.333953410	0.366915055	0.419220968
0.382111151	0.425248640	0.466822939	0.412799222	0.496462430	0.500411024	0.407258952
0.044005112	0.035143527	0.047895030	0.041762575	0.053881757	0.042393521	0.063268579
0.907050364	0.910302926	0.907971484	0.918917773	0.926830540	0.928056156	0.897552235
0.709509999	0.724302985	0.751558767	0.790818562	0.755922314	0.854558920	0.706014595

0.904714509	0.847691857	0.920233681	0.900996056	0.916638718	0.929194689	0.874462687
0.414700932	0.417331850	0.412395303	0.481897917	0.462443111	0.392642043	0.447681130
0.019269936	0.022607910	0.024688714	0.039661948	0.027571280	0.028895267	0.029131925
0.016548732	0.021048945	0.016730971	0.021498198	0.020257238	0.022904492	0.026208939
0.297265101	0.313571191	0.250116010	0.201463297	0.366940649	0.393067345	0.391234650
0.794394177	0.691906788	0.812570661	0.772604267	0.775350489	0.607231881	0.733255509
0.376300779	0.343452121	0.394204625	0.395941967	0.405409337	0.356856432	0.415979220
0.331266447	0.353839153	0.327414080	0.312379890	0.380961676	0.284185498	0.349772643
0.751252293	0.845936940	0.795042688	0.803746519	0.858000052	0.910349063	0.772196347
0.024964319	0.026904062	0.025970964	0.023095116	0.025940024	0.024222111	0.027185631
0.521318954	0.561830213	0.561096879	0.527803456	0.560717447	0.678884185	0.577594589
0.183568664	0.183595297	0.184985096	0.216255307	0.173380866	0.167942723	0.250540257
0.038526289	0.050963533	0.047564459	0.042799938	0.043118232	0.077300104	0.071370147
0.019463867	0.018712200	0.021153193	0.019127914	0.023662934	0.021696240	0.025692624
0.027713658	0.025677867	0.025271098	0.019651640	0.026254593	0.033409445	0.034337765
0.862428181	0.896873749	0.880990293	0.906355737	0.913482131	0.920169884	0.917088024
0.043183498	0.051468791	0.046329392	0.048071729	0.051304068	0.067360219	0.057225220
0.677198925	0.726662909	0.786219046	0.737559931	0.718191866	0.774200136	0.769647274
0.104210416	0.138824426	0.083703454	0.096194642	0.113416514	0.224682040	0.103027747
0.791506031	0.838829021	0.842846853	0.819696458	0.845539736	0.850684700	0.836998065
0.188666656	0.234360562	0.191817269	0.229788455	0.215883361	0.215385065	0.222501404
0.071033622	0.083130668	0.075118509	0.053902666	0.073191898	0.094845364	0.090982194
0.509061129	0.537557742	0.564133544	0.545939610	0.542422270	0.570819810	0.534967372
0.854795159	0.849066232	0.871097893	0.876601322	0.844862112	0.888379594	0.855189530
0.067598264	0.080154569	0.064376652	0.066825113	0.084177923	0.179058642	0.085245495
0.029794971	0.028365137	0.026827359	0.025907422	0.024955751	0.043253936	0.056927521
0.049060656	0.047867679	0.058694660	0.055022471	0.047601227	0.076744993	0.061713534
0.020414104	0.016178288	0.018767974	0.018183890	0.019765454	0.024919536	0.025810145
0.209980223	0.203750408	0.190533900	0.240292252	0.262213982	0.214981360	0.273404613
0.613631434	0.638865247	0.640161814	0.616928469	0.632596991	0.663624437	0.682261388
0.436827993	0.504730582	0.503808276	0.448278610	0.522763254	0.616998484	0.451815316
0.053903726	0.055605978	0.048610644	0.047570472	0.055930956	0.062888290	0.076634536
0.810977460	0.779549870	0.784443743	0.785636592	0.762073564	0.809504830	0.791180035
0.021140140	0.029788636	0.021668978	0.023752074	0.036235998	0.045393853	0.035765756
0.783029884	0.821672191	0.806836612	0.807899343	0.753010382	0.838383171	0.841587474
0.041193419	0.069890470	0.056641925	0.063143177	0.086636920	0.100929450	0.059935076
0.050443723	0.052848165	0.040512807	0.047711099	0.050904287	0.049186103	0.055688303
0.152669148	0.158416738	0.150916558	0.100132090	0.155467025	0.109001261	0.070049365
0.043703894	0.044332798	0.049816938	0.045839126	0.053563195	0.056979453	0.070703937
0.142592144	0.163026383	0.134279117	0.188828461	0.128178514	0.200141037	0.194083314
0.520156888	0.560273807	0.523959120	0.499364075	0.518510226	0.586858173	0.529790333
0.055148529	0.067398953	0.055750907	0.050777255	0.077131191	0.165527819	0.096017152
0.118162604	0.117901246	0.138596001	0.144101420	0.146301442	0.154932438	0.169328717
0.027550086	0.028708579	0.023382085	0.025140497	0.028445297	0.031647051	0.035165293

0.806721306	0.816938164	0.832750587	0.825742478	0.851984621	0.867521695	0.804055351
0.869779413	0.844945259	0.895789713	0.889278089	0.900545075	0.893370246	0.859865506
0.050931982	0.108151977	0.058390347	0.051780185	0.073292782	0.123372016	0.084890527
0.020307731	0.022824815	0.021521655	0.018779097	0.021820217	0.032487390	0.023927776
0.028318726	0.028544801	0.033393382	0.036457272	0.034477101	0.036085166	0.049417832
0.030415351	0.029568626	0.032334565	0.043271321	0.033373480	0.045336227	0.033248843
0.407158448	0.458881182	0.350637239	0.411441396	0.539710526	0.405184530	0.449414818
0.032715939	0.034958467	0.035571580	0.037323038	0.045766542	0.043294490	0.040892157
0.024426143	0.031665208	0.039077059	0.033470549	0.042851325	0.057478067	0.035538837
0.025772504	0.036776197	0.028117704	0.026990430	0.034641182	0.042015263	0.044906536
0.032411232	0.033083342	0.028964337	0.046370188	0.029463887	0.065218619	0.029951369
0.116415022	0.149716803	0.122051494	0.169992414	0.135466406	0.110768510	0.184248882
0.769553323	0.814333465	0.739095409	0.834391465	0.808745730	0.784691233	0.775679100
0.010798221	0.009652449	0.011540778	0.013723895	0.009894054	0.014213685	0.022266288
0.274462220	0.304019067	0.317840930	0.304127794	0.324140207	0.396512221	0.433633823
0.063997987	0.063465479	0.058874565	0.055529904	0.060818821	0.085850520	0.091627421
0.251555763	0.299155623	0.297817634	0.341650257	0.280337411	0.250474666	0.355542730
0.063530762	0.060388582	0.078904314	0.071022563	0.072588710	0.141479419	0.061757744
0.024237571	0.030072275	0.031450718	0.028753159	0.025109584	0.043235545	0.044505532
0.034908055	0.044339412	0.031938840	0.037337984	0.063149634	0.058624804	0.035498900
0.030115649	0.024791349	0.026571525	0.039188102	0.041166170	0.043975429	0.034776570
0.093251022	0.117091908	0.092762776	0.111386875	0.137503234	0.234351474	0.116272283
0.873196484	0.881483280	0.854917181	0.907565379	0.904517463	0.885510640	0.904310054
0.059536486	0.070911946	0.050930459	0.044279028	0.056716318	0.155508462	0.061119308
0.021405465	0.030836718	0.024939141	0.051326539	0.029148009	0.035077504	0.038228749
0.074406518	0.083529957	0.092495764	0.092705177	0.105501459	0.066270012	0.128135342
0.044627226	0.052534954	0.037243194	0.037224101	0.040350271	0.067223051	0.034270101
0.446524021	0.471499390	0.442787469	0.460597518	0.507208081	0.671128430	0.501100680
0.047693750	0.058698325	0.040118347	0.046992822	0.048017265	0.115964292	0.056128294
0.037935241	0.040814607	0.042797364	0.039599323	0.042828510	0.064121430	0.037372726
0.048049477	0.070809688	0.061823899	0.065230199	0.067778420	0.058384557	0.086317392
0.020915983	0.020344921	0.021505741	0.027120466	0.022897869	0.024016989	0.051778554
0.351353531	0.299903253	0.344532302	0.332354020	0.370000553	0.321160615	0.389301142
0.900879019	0.931414681	0.915085712	0.937294823	0.926021686	0.945556085	0.915072864
0.307861990	0.479438150	0.368828086	0.335077280	0.533285384	0.531461850	0.418780558
0.188330213	0.228464328	0.225228023	0.243424503	0.270151632	0.184578273	0.319452777
0.428144567	0.420861323	0.400309853	0.419543173	0.512400009	0.601547437	0.411453152
0.199412200	0.156800335	0.173477040	0.153409751	0.227469120	0.202232529	0.230920755
0.860043591	0.845440136	0.863885146	0.844755804	0.893509938	0.893486705	0.873744988
0.042352538	0.061508694	0.055652713	0.043807722	0.053104967	0.169255658	0.064685078
0.085254737	0.083991263	0.085801767	0.083044919	0.090899677	0.096214630	0.112807820
0.618797632	0.609423653	0.622085545	0.637904792	0.658671149	0.667988190	0.686172314
0.114311738	0.154676369	0.168490350	0.086552357	0.082966923	0.050777585	0.043559566
0.021592336	0.021221839	0.020342521	0.023629650	0.028329893	0.026834360	0.025325902

0.487444045	0.494613404	0.540186423	0.545750265	0.526549812	0.536018599	0.569580575
0.028296259	0.018332716	0.019511764	0.022504309	0.025849003	0.032279671	0.042674706
0.026264635	0.030478050	0.021949429	0.023353646	0.029423977	0.044296835	0.044262433
0.022883980	0.025212108	0.029701686	0.027471227	0.029591986	0.032743136	0.037435624
0.059388310	0.069029249	0.070879748	0.074726940	0.076662424	0.064765548	0.098317468
0.037768823	0.034794465	0.034163495	0.048306017	0.037181560	0.049755190	0.072457760
0.016163989	0.015143998	0.018425739	0.014987574	0.016479013	0.021573452	0.025416938
0.693236240	0.722762707	0.720129575	0.752062462	0.711338420	0.790136515	0.705675072
0.026556719	0.031670418	0.025306167	0.031995878	0.029775848	0.050225796	0.033969865
0.022575342	0.024537284	0.022780265	0.024184061	0.021854076	0.032984706	0.029101427
0.185817818	0.224670245	0.187761606	0.226246752	0.294883317	0.167999490	0.215024288
0.946567784	0.942594010	0.957431852	0.895817815	0.833737937	0.934922798	0.911557946
0.045438489	0.066173692	0.037841229	0.043568932	0.056265012	0.189596167	0.063194870
0.041966436	0.032244252	0.031806681	0.028284007	0.031897561	0.041212046	0.051298990
0.773606354	0.845398553	0.761633964	0.788592333	0.806595846	0.889165871	0.759587815
0.609097449	0.609013335	0.627393449	0.614677060	0.605083832	0.617025366	0.651347970
0.044416195	0.036037322	0.043886826	0.056440295	0.041405638	0.050618491	0.035115278
0.020663562	0.033252302	0.025207504	0.028107390	0.024238341	0.035556156	0.034245744
0.047726707	0.051987718	0.047970203	0.040329123	0.055610103	0.067995684	0.077347632
0.416596359	0.500056237	0.493219976	0.474964066	0.557112915	0.534704835	0.576013267
0.117037436	0.131274426	0.103974764	0.130920247	0.149519407	0.274147616	0.137263481
0.086875526	0.119466696	0.089180594	0.121769319	0.104753947	0.236360868	0.104084615
0.033993299	0.048432224	0.037294787	0.030264131	0.034543464	0.057341918	0.060165515
0.645999480	0.723932029	0.711704232	0.703111168	0.762400580	0.852393867	0.617824690
0.040357903	0.062636450	0.056200906	0.046533218	0.060268782	0.141617788	0.078184194
0.030720679	0.031388599	0.025296276	0.033720432	0.032146599	0.033224735	0.051084811
0.863080326	0.916532569	0.871415847	0.904201596	0.912879856	0.914914843	0.890674775
0.025516966	0.032340742	0.027388413	0.028403103	0.027588738	0.047993285	0.026451089
0.026668310	0.028715268	0.027989293	0.029765403	0.035787379	0.029390871	0.025698317
0.047678455	0.039450089	0.031711504	0.032597432	0.052418158	0.066241873	0.039521383
0.012570426	0.016075895	0.012204855	0.015024287	0.016081706	0.013025108	0.024623610
0.951051454	0.955430716	0.959318199	0.942690282	0.954946634	0.948908767	0.934530519
0.638403530	0.734815665	0.688621971	0.654978910	0.742399354	0.780717376	0.609045528
0.029890513	0.036201835	0.028265330	0.031344030	0.033975812	0.057658672	0.072904727
0.059620444	0.047316463	0.048655817	0.065320245	0.060872983	0.078032352	0.089488089
0.023783965	0.024072325	0.024649405	0.022996679	0.019382169	0.031343421	0.028976659
0.033476982	0.042040713	0.046199158	0.040527678	0.044146990	0.067865191	0.046221417
0.238727946	0.148027809	0.137729564	0.214430992	0.206434275	0.279845329	0.236752127
0.018582760	0.020245295	0.020649973	0.023206584	0.019308813	0.022913244	0.034374722
0.012465878	0.013811801	0.013208124	0.013638686	0.017725341	0.017764021	0.024309165
0.542715976	0.489493421	0.568949649	0.560387671	0.552971512	0.661787565	0.509227186
0.036225624	0.044769282	0.046280539	0.035895186	0.056391289	0.079837378	0.048215559
0.156221489	0.155447846	0.190819697	0.164254734	0.201386199	0.201820746	0.200918797
0.031343343	0.035875438	0.027698770	0.028014984	0.036709174	0.045989948	0.036036969

0.034775344	0.030793699	0.028222769	0.026373795	0.032625478	0.029091054	0.038837755
0.231867912	0.263553982	0.211441678	0.220137472	0.325899959	0.340318475	0.220392248
0.184265629	0.184193138	0.160508789	0.190565451	0.204143010	0.136481439	0.228487760
0.067449123	0.085383363	0.054030345	0.072712472	0.098674519	0.071251648	0.138917413
0.050041854	0.055924331	0.047331695	0.046171636	0.053508547	0.156156759	0.055130749
0.068797694	0.075184852	0.071909371	0.065262230	0.072446719	0.096484649	0.108308978
0.015744791	0.019295654	0.019086713	0.014640122	0.018049418	0.022682766	0.021076435
0.025845521	0.030718125	0.018887072	0.090555483	0.031512683	0.042278917	0.334190720
0.445444792	0.507156563	0.355078651	0.379625535	0.566648325	0.475851875	0.485359063
0.888990786	0.901639523	0.920455614	0.906110742	0.912000346	0.906929401	0.921986375
0.035739058	0.042489604	0.040279146	0.041475852	0.047895432	0.047072939	0.065706424
0.050617660	0.073757517	0.066718747	0.137166896	0.059421973	0.219360308	0.068728844
0.784654508	0.799935371	0.726960316	0.829789018	0.863305886	0.858460368	0.769885933
0.389569250	0.393303437	0.482739790	0.412368643	0.416385273	0.427843096	0.523632584
0.296688373	0.447657774	0.484355751	0.482309129	0.463844093	0.466489146	0.517099608
0.863046180	0.834129252	0.856159058	0.890621177	0.875227279	0.885514754	0.860204558
0.023610400	0.036048486	0.018901803	0.019225630	0.025952657	0.041069724	0.040559758
0.166444315	0.169620882	0.185883199	0.186992749	0.256730255	0.127421861	0.216433801
0.039660408	0.046709067	0.047310514	0.037647477	0.053208467	0.047153355	0.064816039
0.202713192	0.230180647	0.224928757	0.249558525	0.248267831	0.227325082	0.300577623
0.027923746	0.023787720	0.021996536	0.023160301	0.024829570	0.023700549	0.030645523
0.570131279	0.679949302	0.686170322	0.628895563	0.720386470	0.713676442	0.665962676
0.036603725	0.044399491	0.037034648	0.045401263	0.051629263	0.067150103	0.047257668
0.060024298	0.067992927	0.052900495	0.043300258	0.060285816	0.162081230	0.095167773
0.037483643	0.060109494	0.037797252	0.036728668	0.035241685	0.090163981	0.048480219
0.182920777	0.213023279	0.186757693	0.217926607	0.204081254	0.178413007	0.202317046
0.034095950	0.037522501	0.037257736	0.036167896	0.045126273	0.061223699	0.044167132
0.027349981	0.023953515	0.030207575	0.026668563	0.023953977	0.037812007	0.028444015
0.038349872	0.037280508	0.041559943	0.036224349	0.035764367	0.050620272	0.068400326
0.949191025	0.941120526	0.946998353	0.735151027	0.959858908	0.914990312	0.921174928
0.022818053	0.031371730	0.030333463	0.024277648	0.031944911	0.030026350	0.037313561
0.045060208	0.047817726	0.056981721	0.051715623	0.051949550	0.070195036	0.077214033
0.218594326	0.233181406	0.246819899	0.245645304	0.233394717	0.289634685	0.278767819
0.107128326	0.173110667	0.138400361	0.105697503	0.212538296	0.116501585	0.114677184
0.026118573	0.036910095	0.027114124	0.026628856	0.035019623	0.049914761	0.032248283
0.025049051	0.024459627	0.028207166	0.033845644	0.029227488	0.037597456	0.025987572
0.143767732	0.157937655	0.157894897	0.162541799	0.221823226	0.119114452	0.225603016
0.220894890	0.238194719	0.250956911	0.245284917	0.267243398	0.282161299	0.302108012
0.018149614	0.036069413	0.025226501	0.024942825	0.020799984	0.043621845	0.026841725
0.915173272	0.889768410	0.922591763	0.883883849	0.846072401	0.869365432	0.883945327
0.464684357	0.421771516	0.443713482	0.417885020	0.510875874	0.487410420	0.475634094
0.913538547	0.903577723	0.908907483	0.900509176	0.903371026	0.763372459	0.857820893
0.019326239	0.024509593	0.016244186	0.016867887	0.028412972	0.026358523	0.026997297
0.222479437	0.246000396	0.260966401	0.273654680	0.273504505	0.212484255	0.348508568

0.015633131	0.015360231	0.017729927	0.014875867	0.019026971	0.018592997	0.028716705
0.017353414	0.018389273	0.015286894	0.020734994	0.025309109	0.019702366	0.034676876
0.061040187	0.065752869	0.056426337	0.056291452	0.062318593	0.063967006	0.062700962
0.019571606	0.021095862	0.024677066	0.029975969	0.027326524	0.038860816	0.050198312
0.056127414	0.064706227	0.051974212	0.052004068	0.062055143	0.078193624	0.065400900
0.025899589	0.024951516	0.023750252	0.023350754	0.026761816	0.032426385	0.027424307
0.794019696	0.782076136	0.869630421	0.863331284	0.810762309	0.910258369	0.800433307
0.043156430	0.052507777	0.044079448	0.044163901	0.050569549	0.059039365	0.056411664
0.020072711	0.019738247	0.018862795	0.021697888	0.018405211	0.026412249	0.027076032
0.563662456	0.650181266	0.617245149	0.607034308	0.598618818	0.660132749	0.637303570
0.419547480	0.432673091	0.430990175	0.534875921	0.505740203	0.517236013	0.477483466
0.768770544	0.799086840	0.789611611	0.786553448	0.819738734	0.851933339	0.727292734
0.511980788	0.522848192	0.549809096	0.611335937	0.607699834	0.555208750	0.596388559
0.429629241	0.428563727	0.449238528	0.443359157	0.432984051	0.536846795	0.478945318
0.027019679	0.024349820	0.024096427	0.023056679	0.030903507	0.029651593	0.048443525
0.016246211	0.020564001	0.023660733	0.027855327	0.026323150	0.023446358	0.025645418
0.829840412	0.831084960	0.839753873	0.880997775	0.882404889	0.906650346	0.868432395
0.022671069	0.019563257	0.020319822	0.026007991	0.024621140	0.026185198	0.031491253
0.022276814	0.022623579	0.027324521	0.025319475	0.030207822	0.036383495	0.037289171
0.032470884	0.031421371	0.025969677	0.032557166	0.034871711	0.027235115	0.038002954
0.063864796	0.069995824	0.060784431	0.075226464	0.072912462	0.088486462	0.088444029
0.748898501	0.721746070	0.726844077	0.737806078	0.793366590	0.730991831	0.780139771
0.026014786	0.033493807	0.024881479	0.035145439	0.037486071	0.045262238	0.032036374
0.039031507	0.036288715	0.027121077	0.028748425	0.037858976	0.033756593	0.071504879
0.030090818	0.024950269	0.028683701	0.030386643	0.029009670	0.034882173	0.055371997
0.407106917	0.472797919	0.417964107	0.400321270	0.483643698	0.572515032	0.457892996
0.069072881	0.078067984	0.086644724	0.094126763	0.092351226	0.080705909	0.088412269
0.903928022	0.867787930	0.889492973	0.885007375	0.902665649	0.920722687	0.897071385
0.040788842	0.054412012	0.046379748	0.041775779	0.050577753	0.140594893	0.060498606
0.800354969	0.834279946	0.839075974	0.865539952	0.903602560	0.847613309	0.835465192
0.051547831	0.050263838	0.074716373	0.063632972	0.070002686	0.051452941	0.075152933
0.023296762	0.023838846	0.029824155	0.025951142	0.043819674	0.031766633	0.040543345
0.032606641	0.027377762	0.032843374	0.029892682	0.027908524	0.029823814	0.033656640
0.195963974	0.182089680	0.189402772	0.208957874	0.150524210	0.165604789	0.235100439
0.402932422	0.413737020	0.417050225	0.417479890	0.432556025	0.470591127	0.448357699
0.044300037	0.040762156	0.041814781	0.036422942	0.061783736	0.063930977	0.061112857
0.175828833	0.183262806	0.167392653	0.203876138	0.205751137	0.162944744	0.280567405
0.195990015	0.238613891	0.338771485	0.212655778	0.387638049	0.395867971	0.398375366
0.032622216	0.038986840	0.047798150	0.040241783	0.043526613	0.052835054	0.040721888
0.357873240	0.357400116	0.375636306	0.421683091	0.420103242	0.342853892	0.432028305
0.072997738	0.122333057	0.115925502	0.111843089	0.139506776	0.126655211	0.197909454
0.052699883	0.047052295	0.045766770	0.046096515	0.058412326	0.062787166	0.064281911
0.682776675	0.669460681	0.706062412	0.723581153	0.724345453	0.807315893	0.692498584
0.884888679	0.871241112	0.900913147	0.899058157	0.905270923	0.902877075	0.870550118

0.296070142	0.327169787	0.279720185	0.386992563	0.444797384	0.376891719	0.234283163
0.409130780	0.430915968	0.421725551	0.456266910	0.471322605	0.483039841	0.462575189
0.043697001	0.053333595	0.037924463	0.041500218	0.040328298	0.056768663	0.069023224
0.076729788	0.081392619	0.063825157	0.062849099	0.086690601	0.090096149	0.070930440
0.201333476	0.231992926	0.191001090	0.238816691	0.221403899	0.206949345	0.267414203
0.969598471	0.970428354	0.972867151	0.969300353	0.972798719	0.958146604	0.961194574
0.796960501	0.753671534	0.846016804	0.822583079	0.829133177	0.866869868	0.710473900
0.572592317	0.614178190	0.638721582	0.591973065	0.589652592	0.717070567	0.596146550
0.069072773	0.089689525	0.064622497	0.077221004	0.089429080	0.141474523	0.074012179
0.049858760	0.043548798	0.066424300	0.056456488	0.057104601	0.068301513	0.086936119
0.103635636	0.150779925	0.129062809	0.107992368	0.156848113	0.142353560	0.164998017
0.028853276	0.027849888	0.024561580	0.026284219	0.028147153	0.032975741	0.042801641
0.036115620	0.057776041	0.038926283	0.040950926	0.044520406	0.073416155	0.053621518
0.127660152	0.118837903	0.137515360	0.116592276	0.126370829	0.161630017	0.146559485
0.024708624	0.022204496	0.031813607	0.034967114	0.029387564	0.030308362	0.045627540
0.049290885	0.056368848	0.049730619	0.065115045	0.062603567	0.053091393	0.070975801
0.019316823	0.019930417	0.023330547	0.022739735	0.025840735	0.022427172	0.036443255
0.078764388	0.103928704	0.091586704	0.083650320	0.094960238	0.101252902	0.152959751
0.115661010	0.150882236	0.156180881	0.099618809	0.085881089	0.044068104	0.044399830
0.376107217	0.396007594	0.429887667	0.403304990	0.441486177	0.642352647	0.472503166
0.037231757	0.028722822	0.035058842	0.049375112	0.035284846	0.049894725	0.052881933
0.314530104	0.339510190	0.390577070	0.397372959	0.362947069	0.400319719	0.393091890
0.030072141	0.027107334	0.030981227	0.035447239	0.037484014	0.041937101	0.037812264
0.058883248	0.058909829	0.061179341	0.066255926	0.060925901	0.060453607	0.087982530
0.585401451	0.623269101	0.668875759	0.638221257	0.673098934	0.749334067	0.629417943
0.096567472	0.103217004	0.056953608	0.093865384	0.146265162	0.086208221	0.111886819
0.607236310	0.588468336	0.614799267	0.603686921	0.656648895	0.759256347	0.613342613
0.625728672	0.645274292	0.683886549	0.601969317	0.607060503	0.679190542	0.660832930
0.695691554	0.719997523	0.760743799	0.701349818	0.699827757	0.812227092	0.701692670
0.039958568	0.035313048	0.043141837	0.033023816	0.042865095	0.044036932	0.038062093
0.051222801	0.055821634	0.034826754	0.043490668	0.040637936	0.097625612	0.092606837
0.036345230	0.048922977	0.029699180	0.047958616	0.049261340	0.060443907	0.051398643
0.019926455	0.021563203	0.017718550	0.021699773	0.018578256	0.020285266	0.025474901
0.053050023	0.059711658	0.050350906	0.050073915	0.056754327	0.094946335	0.057603440
0.021896489	0.017066348	0.016793452	0.019147594	0.020649779	0.029810912	0.024652787
0.052288635	0.047316684	0.044512047	0.049948095	0.058084358	0.072371240	0.047932641
0.044793645	0.072616474	0.052973134	0.047713688	0.048549235	0.060331973	0.056467488
0.022029522	0.025919123	0.025360447	0.024346309	0.024561492	0.024558625	0.027567997
0.055206637	0.067884261	0.059223351	0.053788948	0.067363770	0.086229249	0.087550195
0.022972523	0.025701786	0.024839616	0.024664173	0.027729064	0.039659962	0.029245190
0.051911415	0.046051758	0.053717222	0.085760654	0.081880082	0.076870975	0.115350134
0.084076336	0.075962014	0.073370983	0.070600137	0.102234855	0.094595919	0.129766281
0.019645995	0.022096282	0.028206343	0.028120614	0.022849959	0.034104150	0.039677664
0.870687640	0.907898799	0.904639689	0.914033173	0.915216686	0.923686616	0.878893002

0.046835309	0.050064719	0.052724637	0.049790743	0.058645539	0.067744666	0.057467176
0.505082904	0.550904514	0.560253978	0.576765759	0.571129684	0.584824798	0.591284870
0.051366237	0.052605357	0.062921221	0.043824993	0.057203304	0.060032910	0.072164102
0.046402058	0.055093645	0.050521919	0.043716317	0.051210164	0.078228440	0.084650566
0.559914264	0.606514387	0.574989191	0.605196894	0.614908658	0.575358274	0.610828898
0.125738313	0.148560726	0.168689272	0.129074522	0.190772518	0.067779494	0.194031055
0.654063626	0.691121500	0.682209081	0.683229857	0.662136633	0.741353571	0.725253578
0.022787009	0.033368428	0.031161071	0.022965140	0.025029826	0.083306230	0.037173412
0.253660186	0.321459560	0.338581399	0.344621873	0.323944980	0.330811133	0.393252084
0.032183403	0.042364044	0.037805306	0.049462653	0.051099769	0.043971863	0.047060977
0.163578303	0.147348355	0.164400749	0.193227149	0.152416885	0.170700184	0.208731602
0.061594992	0.057351560	0.057027743	0.057587577	0.075642463	0.068606106	0.071635456
0.322869311	0.369354351	0.400381139	0.437701610	0.390447294	0.381771424	0.437585408
0.829861548	0.859423570	0.821170829	0.852979713	0.848213303	0.917379155	0.857853926
0.035592992	0.046974839	0.034547411	0.044067414	0.043432061	0.037150335	0.048484108
0.052340369	0.065520006	0.059884070	0.065846854	0.095873322	0.189257482	0.067304746
0.073462772	0.078383422	0.097777232	0.082234648	0.091478157	0.116010780	0.090477496
0.869495113	0.905268471	0.906434466	0.926835598	0.892779146	0.918662684	0.896212253
0.389297836	0.349827541	0.375033654	0.434275725	0.367565689	0.468158593	0.427865851
0.022278496	0.018733950	0.020316214	0.018554871	0.017493487	0.025306728	0.037626249
0.088617447	0.190748892	0.095122012	0.102086847	0.158253574	0.325360424	0.181505647
0.047267232	0.060063063	0.067139856	0.048833082	0.065388848	0.078262000	0.083472903
0.021612495	0.028925381	0.028728042	0.039275102	0.029110584	0.049134241	0.042316951
0.043284467	0.040684210	0.051271648	0.041642943	0.051965628	0.071582331	0.047907816
0.073016071	0.073525075	0.060643335	0.064246094	0.079530401	0.082088995	0.088557702
0.030295753	0.032524433	0.025917385	0.034905344	0.034499198	0.052815969	0.198341834
0.047722465	0.058812949	0.052113609	0.041721543	0.070874272	0.063577837	0.051413220
0.425235771	0.514741574	0.435575775	0.488287908	0.557682155	0.481773719	0.522282692
0.042253055	0.053577636	0.035900360	0.036504845	0.039022394	0.082835048	0.049289468
0.029668073	0.032154538	0.032418977	0.038122837	0.033436027	0.054948774	0.034063057
0.120297754	0.146514691	0.117895042	0.139867562	0.189535717	0.141777215	0.204653371
0.045732635	0.044406421	0.038700426	0.053193321	0.054380446	0.060688931	0.057654529
0.502595148	0.604775278	0.576816047	0.626327197	0.590189296	0.600388202	0.626290102
0.026942344	0.029929642	0.028212819	0.025481741	0.026963160	0.046576303	0.037377215
0.027267453	0.048241953	0.024880581	0.029667867	0.034493671	0.051244990	0.037894081
0.716598860	0.741711120	0.783702185	0.709938757	0.680094536	0.579380554	0.727045421
0.400181606	0.408825698	0.424205337	0.406782267	0.400401041	0.467126523	0.456711429
0.030582996	0.023066915	0.028008227	0.028632140	0.028644166	0.045704140	0.028139855
0.026625632	0.029396557	0.029567180	0.039255494	0.025332674	0.038989065	0.042299390
0.673883425	0.705908845	0.768985637	0.737485258	0.862812473	0.909332269	0.797024179
0.072769083	0.059628131	0.054396929	0.073210779	0.081639219	0.061447330	0.095932847
0.044861595	0.049709140	0.042269220	0.031552678	0.048866370	0.098332869	0.047973109
0.087591398	0.118753475	0.107773674	0.130192562	0.122604472	0.050297025	0.158942367
0.022966670	0.031248409	0.035268011	0.041598725	0.057134775	0.037448916	0.023738432

0.043158791	0.025027984	0.032477713	0.037626017	0.039851877	0.047525475	0.040514685
0.389897864	0.495721669	0.492402349	0.448489177	0.454004504	0.653999079	0.492944342
0.024079719	0.023531166	0.023931717	0.029777816	0.031584145	0.031940697	0.028968540
0.031878682	0.040933498	0.041266664	0.036100623	0.037515492	0.042367667	0.060261143
0.167502227	0.154994328	0.180766305	0.182554336	0.196435118	0.215578396	0.224149879
0.036712137	0.060785859	0.076131021	0.018876402	0.020162222	0.015739912	0.054503296
0.196391609	0.159164626	0.168771008	0.182815145	0.206929828	0.216380471	0.213108103
0.021471532	0.013948993	0.017711607	0.018213444	0.021531792	0.023388879	0.023472728
0.707235956	0.842235585	0.744032732	0.821518260	0.847760917	0.826471925	0.703792223
0.023693970	0.026471468	0.025059647	0.022360388	0.022153780	0.029557128	0.041376480
0.174096756	0.184955056	0.184425761	0.178704490	0.178371907	0.223445154	0.271774155
0.022599327	0.020029496	0.021505153	0.028849684	0.025170470	0.033165951	0.029045534
0.030255813	0.027184603	0.026126940	0.031901270	0.018675697	0.051112977	0.049263860
0.035940899	0.042536642	0.033123101	0.040220439	0.049381512	0.055938836	0.050012496
0.060197075	0.089398842	0.089279644	0.161127174	0.135917968	0.182567246	0.287985192
0.019761474	0.022484937	0.028186661	0.021116208	0.027125821	0.034028662	0.043745610
0.857563495	0.866991544	0.859991010	0.866960018	0.894736857	0.861074310	0.872217216
0.091180670	0.098959869	0.071695378	0.075277442	0.121233670	0.339488338	0.084398173
0.149338099	0.112035555	0.123203890	0.096560018	0.147865875	0.172201136	0.165991741
0.956449248	0.950575659	0.956903824	0.936186498	0.935837027	0.937650745	0.928849249
0.023455994	0.024639096	0.023645400	0.024748360	0.031810186	0.037899729	0.028429027
0.077825065	0.082150599	0.065037393	0.072185383	0.089778281	0.226621615	0.067983922
0.032918327	0.043632964	0.042711471	0.044132093	0.054602594	0.046726712	0.057910821
0.053392641	0.055886034	0.062027592	0.048458634	0.062013638	0.071373153	0.066695055
0.022700804	0.036991185	0.042149438	0.042222653	0.034951036	0.088870161	0.040807279
0.034300759	0.036441496	0.033564750	0.044835345	0.041049180	0.047772946	0.038114073
0.029980003	0.043165793	0.033963444	0.041472045	0.041522901	0.040458299	0.031721296
0.288766058	0.279742094	0.304848110	0.330666494	0.333402325	0.243885003	0.348156087
0.581281313	0.604405772	0.673231959	0.602235094	0.579604554	0.610055268	0.629418072
0.021762766	0.021895973	0.020468431	0.023766235	0.027845754	0.026774978	0.030879908
0.069960672	0.078078166	0.078520402	0.074524322	0.086060134	0.141697970	0.062847292
0.042084064	0.024677067	0.031544227	0.043449097	0.032146742	0.048293321	0.058174645
0.942095578	0.940791086	0.950012776	0.958531220	0.954418017	0.872095450	0.900369601
0.053780824	0.042147262	0.055484605	0.039938331	0.040198292	0.070570605	0.050814989
0.034445295	0.034189548	0.038910902	0.035598168	0.050278452	0.052577473	0.047885400
0.030838326	0.044680708	0.041771401	0.036420398	0.052704364	0.103446081	0.050010568
0.140844806	0.136956101	0.142952596	0.123688818	0.140824420	0.146847229	0.220446849
0.738713136	0.707561105	0.759328009	0.798890162	0.793847315	0.822419116	0.733455462
0.787631889	0.808176477	0.802210884	0.828803830	0.854343500	0.853995262	0.761919821
0.041960691	0.082995191	0.054842519	0.064894826	0.139429338	0.078551633	0.108961302
0.628024736	0.660440380	0.718524248	0.744219606	0.763958513	0.796019567	0.715119176
0.297731626	0.334647203	0.336235440	0.340491978	0.297647254	0.335101951	0.414451869
0.041531603	0.054072544	0.047538300	0.046077327	0.053921189	0.053990724	0.065458760
0.080664532	0.116954741	0.114012050	0.097426719	0.125562124	0.225210413	0.091352781

0.036441464	0.055632560	0.057971687	0.039857391	0.046536417	0.132457109	0.048612698
0.050815215	0.046314019	0.047743763	0.039607539	0.045533660	0.079990552	0.058874460
0.053666187	0.057759532	0.059934394	0.051042097	0.059472207	0.094003405	0.081374918
0.316386038	0.268347956	0.270806190	0.312278143	0.311351565	0.342022113	0.286303507
0.037811182	0.035617485	0.038283961	0.045213937	0.039784022	0.061373682	0.032331065
0.025147020	0.020488989	0.026633877	0.028021334	0.028925216	0.032658306	0.026512721
0.498025842	0.493901575	0.496161889	0.474903596	0.497439817	0.552458494	0.496010732
0.752220941	0.699141539	0.789537904	0.785643742	0.769999087	0.873355763	0.717887395
0.056457216	0.048987532	0.045494035	0.056054039	0.053498450	0.074972088	0.105950925
0.045616938	0.052615116	0.054547680	0.053159588	0.042903311	0.097223849	0.043171956
0.823856556	0.759831754	0.809425391	0.825575723	0.837800945	0.843022543	0.824476156
0.037964893	0.053533004	0.038702756	0.029892020	0.042300009	0.048893280	0.066115618
0.051848805	0.037309419	0.046728753	0.037554090	0.043671661	0.071664239	0.060338832
0.056131423	0.056597260	0.047689838	0.056050932	0.067682730	0.088235441	0.065370532
0.778307268	0.753496197	0.824493720	0.778406716	0.710450634	0.531371307	0.747469673
0.071803354	0.127727106	0.104524990	0.096528070	0.113849559	0.233130075	0.128277510
0.458229127	0.555915315	0.567476798	0.564038772	0.591736637	0.587700370	0.562053118
0.077745316	0.105074437	0.110629156	0.097528299	0.120633047	0.267542183	0.103403778
0.076632573	0.149624625	0.085565196	0.109376616	0.097659209	0.147259268	0.092836086
0.342653578	0.339925505	0.406969915	0.344755334	0.354930782	0.604191461	0.372178574
0.027365080	0.025847990	0.025243779	0.024438275	0.024851520	0.035479606	0.043176970
0.042667885	0.047467172	0.060935248	0.054858163	0.052964001	0.055775333	0.068200621
0.545911196	0.642049537	0.679035757	0.623469875	0.639298063	0.760976937	0.662118428
0.882863112	0.871689601	0.890782240	0.901484532	0.875507640	0.910047012	0.872659778
0.643744039	0.701001014	0.700960708	0.771961388	0.681879841	0.748407304	0.676390798
0.098966692	0.118780276	0.117470208	0.121052924	0.119440844	0.114285959	0.172291254
0.471653793	0.439388621	0.474244690	0.488978515	0.559916780	0.446131010	0.493130968
0.361548350	0.392069199	0.451299620	0.349550702	0.454727254	0.467057402	0.454618643
0.027606457	0.027827268	0.039967298	0.038033637	0.037338504	0.050877559	0.034748178
0.492712952	0.552312178	0.472157707	0.670878500	0.523967621	0.595354058	0.604757092
0.024622027	0.031919794	0.022399917	0.028044920	0.024445799	0.038871310	0.043584246
0.038869460	0.092652806	0.074892982	0.068189044	0.079481536	0.141190380	0.108742662
0.035838262	0.057263565	0.057387762	0.040363909	0.053522272	0.061074471	0.059302686
0.033732021	0.041200718	0.031281086	0.055029756	0.039598344	0.052037025	0.046251223
0.065010047	0.068948217	0.075402933	0.077484568	0.073675022	0.086706230	0.092195113
0.165903907	0.219719460	0.190480777	0.180691975	0.178691789	0.202088480	0.249237379
0.036350158	0.023601671	0.030810734	0.028584464	0.039669706	0.055237608	0.036748242
0.045177920	0.047942537	0.051664333	0.063550134	0.051712771	0.056816037	0.075736099
0.439413035	0.608854373	0.613870018	0.630588249	0.618915812	0.585918509	0.628730089
0.260298274	0.178248006	0.243935413	0.188359880	0.311823814	0.334955974	0.336614267
0.033633009	0.039803273	0.035022073	0.030704201	0.041206470	0.058964733	0.060839961
0.262599211	0.340818782	0.351193074	0.293003225	0.320625249	0.348520216	0.380906158
0.028443604	0.025547361	0.031249644	0.022848030	0.032745930	0.036506448	0.039026689
0.030916430	0.031919404	0.028999568	0.032891080	0.038267171	0.041362418	0.031457939

0.031059531	0.030216423	0.041343490	0.025315261	0.041787740	0.048852265	0.047310692
0.036254727	0.048911409	0.046391620	0.032541829	0.045975364	0.084058536	0.044480759
0.494882426	0.548118279	0.476033748	0.556233511	0.532269586	0.641811838	0.554661992
0.822370485	0.841999416	0.853818298	0.856294336	0.865234332	0.848608876	0.867310804
0.467720817	0.443888869	0.469801780	0.389516740	0.440318211	0.527193201	0.472576352
0.048371113	0.029268805	0.023679727	0.036732976	0.042039046	0.066970519	0.079227551
0.037336542	0.046394141	0.043850880	0.057250340	0.055472058	0.047219563	0.049440236
0.482258890	0.515436343	0.577433090	0.630964634	0.542986863	0.585154020	0.503557511
0.048192990	0.046256350	0.039819072	0.044282433	0.052931719	0.060315128	0.069608682
0.114967692	0.130012764	0.117191984	0.082009089	0.168054080	0.147444446	0.122547335
0.451527947	0.467734802	0.477942503	0.446953842	0.420649785	0.562294896	0.464533282
0.051228387	0.071935663	0.052611220	0.068306135	0.061781757	0.144308822	0.068395580
0.078745943	0.072409191	0.083386174	0.098976530	0.097093235	0.103986700	0.114789373
0.034886154	0.043767563	0.040036255	0.037631384	0.052204435	0.069712010	0.039583717
0.067544607	0.086724572	0.112932556	0.120716241	0.089758622	0.072793620	0.146709531
0.030896142	0.028916675	0.030498038	0.033365004	0.033800939	0.046244300	0.032385984
0.226772831	0.257615365	0.241041737	0.281448163	0.308962032	0.245989688	0.304259531
0.273883562	0.315754311	0.299656068	0.312155695	0.435771038	0.265504398	0.359722729
0.140941133	0.114078456	0.145621345	0.128912827	0.148857610	0.138299244	0.187759928
0.019513202	0.027710779	0.022474841	0.018078940	0.023209527	0.036860309	0.030789503
0.040239491	0.048897733	0.053611830	0.054856831	0.047236672	0.063823056	0.084061540
0.849889332	0.888981262	0.887909917	0.893743295	0.874377929	0.925705591	0.839438708
0.024660988	0.033594480	0.024350480	0.029091915	0.038068768	0.040077531	0.030559136
0.827864502	0.891602839	0.876568913	0.890099589	0.865506056	0.931050383	0.883492913
0.148025782	0.163536946	0.178886660	0.155976240	0.168643840	0.160234499	0.187153912
0.858654923	0.911198578	0.844436692	0.905373514	0.913854386	0.906033321	0.891875538
0.057320417	0.052526740	0.063938594	0.036301705	0.082766057	0.075970813	0.076736316
0.557239207	0.550718464	0.576087103	0.595880984	0.678975996	0.655268118	0.628557632
0.017193584	0.018797103	0.023787946	0.016356390	0.047289515	0.026183275	0.024042221
0.019271827	0.020215523	0.018742884	0.017946257	0.018746311	0.033600785	0.030782952
0.726144796	0.765703665	0.678779066	0.714397512	0.790725581	0.846977388	0.750617697
0.796538954	0.810400541	0.862832786	0.908082817	0.859705357	0.910095378	0.827504401
0.041249173	0.035675670	0.033742386	0.045795524	0.032522841	0.051082975	0.042480836
0.842496043	0.880421425	0.882913658	0.885605891	0.883480694	0.875071290	0.880778331
0.041997891	0.041286742	0.036102650	0.037969032	0.050147624	0.049827131	0.056990076
0.028091243	0.032378708	0.038183184	0.024314429	0.030895594	0.039207989	0.038565285
0.032328899	0.028692227	0.025093145	0.023992781	0.025554385	0.032970503	0.032835973
0.025152811	0.031226447	0.022965465	0.018612305	0.020568757	0.037671787	0.032800814
0.028486064	0.041229530	0.042127904	0.029931184	0.040814270	0.073283087	0.029333174
0.046674519	0.046935077	0.052014357	0.054039134	0.043005352	0.052741223	0.045862455
0.039493476	0.051067646	0.043526773	0.042585432	0.037960624	0.068552828	0.058639371
0.457966962	0.487097379	0.526773937	0.485877130	0.516642509	0.490131499	0.527148790
0.869977543	0.817188069	0.860218195	0.868814569	0.798430693	0.821700211	0.795771378
0.696943750	0.661588161	0.751497651	0.728308160	0.774293049	0.778237572	0.762237206

0.162846403	0.189741749	0.251265804	0.269277212	0.203267860	0.192534803	0.322698834
0.151043099	0.115433514	0.161372001	0.143390114	0.219122251	0.058869948	0.206548620
0.111983011	0.110862781	0.122018768	0.129088485	0.151229444	0.076109827	0.144097314
0.729067560	0.736277794	0.764547807	0.745383717	0.740146156	0.825702947	0.692615632
0.739646833	0.809076243	0.781976636	0.805753019	0.809426688	0.800902344	0.765684861
0.226744090	0.233642453	0.233151574	0.180988991	0.201580891	0.287954679	0.318811852
0.052404543	0.073425676	0.061056636	0.053169593	0.107318507	0.187633459	0.086234060
0.631898940	0.617726534	0.627341068	0.691592540	0.642026462	0.625550826	0.614622529
0.514678313	0.567453089	0.516249426	0.531371114	0.519901289	0.631815124	0.504719555
0.216736269	0.211376260	0.206315696	0.226265362	0.213092675	0.304468564	0.308558444
0.174446146	0.422996341	0.496198597	0.390348894	0.424279823	0.277726618	0.366022882
0.039050309	0.052980744	0.033873968	0.056036392	0.037335346	0.113304920	0.062506115
0.943320741	0.962334966	0.961336902	0.969623957	0.967944997	0.960816518	0.958963487
0.285637095	0.272310418	0.287281304	0.292436849	0.291277918	0.278050236	0.340767432
0.023770710	0.026925058	0.031804188	0.024055207	0.028834717	0.034317088	0.043050791
0.526792239	0.502711107	0.535467967	0.512125254	0.546805339	0.551448377	0.565409929
0.064292739	0.199384492	0.201276931	0.030725603	0.047585114	0.183667720	0.045607994
0.032024579	0.032896930	0.030472451	0.033929253	0.036501580	0.046404807	0.035758736
0.075406120	0.110392280	0.057486351	0.057349026	0.068922309	0.148278710	0.076543148
0.658194422	0.613659565	0.724233904	0.621386532	0.700272359	0.731002336	0.590907424
0.923633844	0.912705837	0.918760781	0.924636476	0.928834903	0.744395192	0.809290120
0.063564452	0.049601906	0.071940352	0.074560987	0.071200404	0.095916959	0.100386138
0.030695803	0.034217884	0.033473224	0.031225697	0.030230597	0.039389213	0.036651496
0.044308473	0.049268875	0.049996884	0.052014923	0.051545208	0.066252154	0.078676634
0.139300920	0.163739729	0.145617712	0.187735950	0.214572287	0.134171015	0.219997946
0.670575087	0.690701321	0.676305927	0.661081533	0.746481757	0.790829333	0.676740583
0.204913698	0.204205053	0.213872101	0.242966667	0.214514588	0.176605601	0.257034425
0.582312459	0.566097908	0.578826011	0.571263415	0.642115173	0.623057301	0.613674882
0.109120567	0.068921255	0.111014569	0.080842923	0.153838212	0.154623727	0.180900255
0.038706362	0.046252764	0.050977619	0.048823144	0.050702711	0.056133209	0.067722153
0.033143799	0.031643292	0.042436952	0.038055694	0.065790298	0.044398696	0.046697810
0.232193813	0.246002890	0.277568296	0.278927570	0.275462794	0.264313900	0.325508085
0.779545149	0.797284766	0.859368622	0.809786992	0.839593590	0.907243371	0.810168907
0.100175067	0.090823504	0.104795623	0.107349192	0.101740587	0.102633858	0.146049485
0.695240508	0.692325117	0.673803593	0.682139728	0.710682372	0.750152489	0.641222697
0.021115870	0.019267683	0.022785497	0.028062786	0.022688815	0.030850476	0.031687567
0.071143820	0.087577631	0.080224928	0.069635462	0.084382839	0.101890950	0.099063311
0.044714964	0.067484466	0.059197303	0.064776576	0.065134777	0.053173135	0.075413566
0.546349617	0.557164306	0.607449894	0.571057947	0.554109789	0.570562258	0.590096080
0.027633097	0.025874209	0.021706393	0.021283085	0.027849842	0.028481805	0.032383490
0.033874124	0.053365246	0.102366019	0.082355725	0.104781517	0.072883138	0.091401016
0.109914620	0.147903065	0.094075467	0.089856035	0.139254406	0.212435335	0.120701583
0.660984718	0.632956282	0.666895059	0.668476031	0.648486816	0.715952992	0.647085769
0.063992267	0.078053347	0.061700292	0.068772002	0.072238920	0.079707538	0.116065500

0.691271160	0.674573861	0.676818868	0.624173956	0.651181927	0.699545057	0.742596135
0.456623616	0.444103251	0.458904820	0.435146234	0.578032063	0.599112564	0.461480093
0.701047658	0.726213127	0.714707944	0.760425196	0.817259539	0.871932624	0.686647401
0.038758645	0.051808124	0.039971347	0.039676357	0.045807162	0.052336145	0.058770012
0.033963584	0.032838308	0.031277682	0.035154153	0.046449491	0.036374364	0.053126446
0.689491201	0.684844011	0.753198733	0.715343881	0.736476035	0.836749669	0.646601984
0.152582161	0.117136130	0.160261454	0.143637003	0.137536799	0.189213302	0.157390986
0.300962987	0.291590374	0.337139443	0.322253292	0.363867832	0.460392583	0.320988389
0.265050240	0.216643550	0.122488448	0.226630566	0.160925050	0.155027598	0.164799830
0.052716246	0.067148247	0.062079396	0.043737300	0.057053674	0.068915369	0.073585023
0.036179862	0.034423797	0.031506164	0.050590297	0.037653829	0.042758144	0.044733221
0.025440771	0.024416603	0.020741650	0.022980611	0.023837789	0.038659831	0.031320382
0.085030527	0.084592875	0.067497237	0.071117171	0.085477646	0.124783231	0.070996533
0.025233172	0.042737555	0.022978361	0.031938013	0.042231832	0.045670340	0.042914168
0.122567410	0.108105569	0.139615825	0.108260015	0.123639258	0.160279457	0.193756165
0.133189504	0.144031060	0.135266313	0.128240788	0.176111155	0.054595833	0.164793326
0.027867379	0.027817040	0.029932287	0.032446359	0.045364238	0.028932494	0.044314074
0.029139922	0.025734771	0.022913057	0.026768332	0.024930359	0.035745150	0.036898214
0.816736500	0.805428679	0.867055857	0.827516097	0.842920954	0.812914694	0.836937342
0.884166285	0.896646419	0.919750985	0.900869475	0.919385411	0.902426399	0.877824735
0.056165017	0.037023851	0.055082807	0.056381726	0.058154559	0.087029378	0.095872227
0.136764412	0.154855219	0.195871331	0.172101334	0.208916362	0.245670585	0.245752530
0.033989473	0.044232858	0.041581984	0.036660657	0.053632864	0.048692804	0.056910800
0.039645330	0.054867441	0.037713770	0.030060771	0.062767447	0.082770497	0.038490861
0.062282399	0.068107730	0.065207540	0.062064066	0.066273546	0.078063631	0.085647123
0.052564140	0.053995197	0.054500757	0.050705131	0.057239350	0.148989476	0.055173870
0.017176556	0.020040564	0.021109249	0.024614483	0.023218474	0.026843141	0.026590431
0.071745523	0.063279503	0.057743227	0.078083843	0.053072926	0.071778081	0.103061670
0.024546002	0.031340350	0.029636648	0.029818814	0.029908198	0.053996116	0.036588434
0.025734511	0.028928414	0.028285771	0.022231235	0.037047150	0.037100428	0.036171896
0.641158982	0.687423320	0.713439857	0.690931120	0.706840795	0.738096222	0.668411404
0.040664716	0.032081228	0.041985261	0.038724363	0.039910868	0.058086581	0.049706376
0.027864641	0.034182426	0.030095002	0.036971806	0.031931442	0.040934002	0.037345381
0.088243932	0.078949216	0.046357875	0.104238256	0.126210355	0.111211717	0.103329323
0.019083010	0.019975146	0.017238903	0.024327146	0.024314797	0.029084390	0.026759042
0.027609217	0.033561898	0.033818803	0.028999322	0.037487081	0.038405294	0.037726755
0.104719340	0.107321648	0.117177603	0.130213025	0.100318838	0.105829539	0.150726300
0.276780025	0.299687272	0.310234305	0.297213662	0.300003830	0.358718271	0.357608517
0.031873473	0.044017810	0.038216186	0.046962958	0.036002639	0.073905168	0.051056611
0.101086541	0.081856898	0.101285373	0.133302904	0.107648776	0.156253294	0.117252516
0.041643706	0.026742867	0.046066527	0.032334060	0.052578712	0.050229417	0.060278687
0.821236886	0.857328753	0.831336165	0.852281610	0.840385211	0.844959981	0.816490654
0.045132242	0.044624414	0.056770160	0.046010347	0.042656444	0.049728326	0.077805065
0.470967743	0.167116082	0.554859889	0.263911096	0.230676753	0.061218654	0.257972560

0.792524074	0.850073938	0.809289782	0.838479135	0.851843906	0.906969889	0.808744288
0.797577251	0.816176049	0.837318437	0.847669294	0.835493929	0.851830735	0.836243732
0.766105700	0.822044283	0.821683084	0.826336722	0.823052867	0.844080547	0.770302459
0.054042138	0.061101543	0.062433314	0.057928973	0.057936238	0.089726650	0.097549674
0.035182982	0.036605368	0.035187614	0.032732022	0.033234383	0.065355071	0.044181323
0.344926495	0.368747852	0.332562520	0.377961919	0.406713750	0.378715601	0.408858438
0.670709233	0.725820689	0.722541059	0.686782229	0.757401151	0.754926397	0.663959571
0.074351469	0.076011164	0.070673449	0.077220101	0.081532965	0.098309683	0.102289726
0.026291980	0.024726932	0.024787447	0.024079016	0.030758748	0.041650202	0.046723398
0.737032331	0.790564759	0.758989225	0.783987636	0.815459135	0.830633915	0.721559722
0.568715989	0.615476397	0.601520305	0.579520837	0.620485623	0.682128171	0.562873798
0.030241180	0.040047175	0.031684007	0.030636887	0.037177652	0.038834350	0.060303878
0.061053463	0.070270333	0.046523741	0.043429473	0.073201350	0.187689659	0.054432269
0.054388406	0.059489591	0.060165183	0.062033233	0.057961779	0.080180086	0.076023601
0.811287993	0.818976250	0.842594486	0.772339324	0.896657353	0.887126876	0.839384529
0.017917401	0.016961283	0.022501399	0.021674010	0.027552714	0.023952838	0.033052987
0.126690849	0.091295964	0.153828318	0.106051134	0.091022980	0.088931904	0.114457062
0.865938820	0.887037682	0.874852058	0.894195942	0.892505833	0.901259210	0.827604774
0.052106549	0.059496105	0.043273053	0.049293063	0.052089877	0.086865046	0.068678404
0.782731050	0.785022750	0.806075104	0.825202660	0.808792415	0.808589307	0.800184777
0.022735524	0.027848896	0.021908565	0.024470790	0.028301177	0.041760880	0.028889758
0.051616235	0.061071110	0.067078616	0.053398500	0.055651658	0.090376159	0.066769813
0.082806656	0.151336228	0.097750225	0.074525934	0.117504898	0.277860956	0.092294325
0.034201243	0.030751318	0.036639856	0.031044689	0.041168544	0.055329403	0.045999187
0.019715644	0.024358580	0.024761617	0.020440845	0.022316091	0.030189715	0.023774126
0.860095741	0.866769233	0.864750599	0.890680098	0.879074129	0.883897105	0.848495750
0.799923438	0.824446470	0.801166286	0.856005729	0.781508496	0.889129250	0.810244267
0.408841307	0.415734088	0.422861521	0.447843698	0.508608401	0.416551783	0.484102919
0.072296807	0.072853259	0.077832814	0.077603196	0.074592899	0.068104379	0.139518019
0.049050526	0.041461539	0.045413644	0.042955249	0.047914507	0.068182731	0.051940052
0.151654689	0.152866548	0.162777046	0.166573183	0.153998164	0.113602032	0.236732229
0.024179868	0.032675046	0.019730302	0.025402133	0.021278301	0.037267334	0.026161995
0.430157156	0.476357794	0.501007624	0.495491571	0.523668763	0.474120919	0.525142475
0.082504460	0.086799589	0.078550083	0.110204149	0.077247666	0.112698952	0.134848994
0.831617510	0.739922280	0.756268432	0.726612650	0.689708483	0.774398665	0.795911536
0.559416887	0.497606345	0.736648045	0.275386558	0.305913964	0.428900043	0.413049860
0.027666149	0.036150951	0.039171471	0.074810194	0.039433063	0.060132015	0.061780477
0.038311248	0.052657847	0.040227056	0.043665730	0.049282542	0.099050051	0.050920803
0.194602984	0.206072640	0.194402385	0.191973772	0.256008358	0.265533102	0.272087226
0.546748098	0.585126108	0.590649585	0.539490213	0.667926809	0.692641969	0.572160460
0.024426300	0.024264046	0.023841531	0.018405836	0.023058136	0.032643611	0.038046487
0.038042324	0.043200678	0.047125562	0.041988138	0.034636576	0.057928950	0.044856083
0.037160091	0.044486475	0.035523224	0.033294819	0.052145981	0.063453366	0.045293802
0.029579106	0.032372127	0.029943740	0.037403973	0.027330356	0.041895799	0.032697452

0.023709388	0.024057052	0.033391104	0.027109726	0.028023339	0.037863428	0.033625879
0.049353723	0.034093713	0.031483989	0.045691331	0.035565242	0.036310576	0.049700271
0.016427683	0.020698109	0.016786791	0.020809505	0.019601095	0.020962367	0.023312431
0.090489082	0.076494897	0.088487519	0.140200738	0.105636289	0.112028949	0.104937178
0.786774201	0.804232263	0.809085203	0.791162257	0.873487405	0.903850593	0.773104599
0.030321172	0.041800681	0.031618815	0.044393246	0.055265186	0.031676307	0.045768972
0.431316267	0.500437514	0.528398875	0.495190747	0.510672310	0.516069186	0.555542412
0.079775303	0.073956290	0.088291066	0.066621928	0.087947775	0.109377344	0.136414821
0.076021263	0.083604153	0.079609868	0.072472656	0.072454641	0.089438053	0.091212130
0.448884878	0.472100926	0.444748039	0.485961702	0.528991102	0.434765023	0.464575687
0.542579945	0.588072051	0.608828976	0.592437362	0.579611920	0.585830851	0.558856609
0.826940868	0.882202296	0.886382348	0.857036241	0.898675889	0.928168531	0.815156098
0.266645463	0.308997783	0.261267740	0.290675830	0.341789277	0.237684391	0.352777858
0.438659979	0.479308627	0.349197485	0.404371411	0.513252830	0.536182187	0.486794708
0.553381830	0.525825343	0.670103770	0.623837511	0.643173799	0.665387440	0.613126913
0.024371828	0.020432025	0.026171018	0.020135719	0.031751811	0.023078877	0.037079381
0.025852290	0.030659136	0.028447611	0.029772632	0.032226913	0.040593566	0.039310089
0.064875104	0.061567579	0.057277149	0.058923954	0.075782501	0.074512405	0.063407193
0.050415457	0.060163406	0.075013856	0.065478952	0.068226233	0.076018752	0.059349476
0.687515620	0.773814306	0.830193947	0.768275156	0.835005024	0.823063855	0.746876176
0.026914463	0.023090371	0.023809790	0.027370640	0.025836477	0.032358542	0.035709083
0.771515757	0.832356680	0.803379549	0.878064765	0.852042993	0.891760060	0.856663977
0.025110359	0.039480298	0.031669022	0.038147449	0.028187186	0.047643792	0.030934499
0.025049538	0.023978844	0.026062592	0.022915297	0.031107667	0.037571841	0.032768266
0.031355962	0.034220024	0.046480810	0.034982156	0.043605993	0.052151447	0.077412025
0.081730758	0.052214103	0.076326696	0.058018231	0.090529365	0.099687436	0.078498780
0.029917838	0.029626540	0.031820338	0.023939344	0.030917400	0.032795427	0.035981855
0.071212512	0.054313593	0.049411548	0.054132156	0.067085925	0.071741123	0.094482376
0.033511321	0.046574822	0.040546162	0.032495822	0.077527325	0.397368129	0.061189988
0.525955828	0.482931276	0.487506974	0.459025857	0.551783869	0.660921028	0.530245866
0.863753320	0.889718546	0.861808071	0.911055209	0.879741886	0.914776796	0.905546765
0.482562013	0.588112098	0.600788737	0.618672842	0.555446589	0.529442474	0.537049087
0.442655774	0.465685289	0.486107059	0.441368492	0.459532912	0.506735782	0.488394885
0.742474094	0.796013070	0.725023171	0.769499644	0.807720114	0.844197424	0.749752849
0.837982040	0.830466063	0.860954620	0.882582636	0.847338242	0.918172158	0.783523114
0.892435149	0.903248463	0.914663186	0.918576522	0.911185123	0.926307532	0.878913348
0.422680520	0.404883427	0.438640129	0.375569953	0.458764105	0.487399152	0.410953352
0.053763189	0.068190532	0.054404550	0.053196933	0.067823733	0.166281633	0.056763175
0.380117272	0.378167057	0.380437349	0.415188165	0.420436122	0.450742395	0.439511841
0.332325547	0.458208153	0.350946047	0.404554639	0.459288008	0.432337118	0.443381195
0.308347747	0.318681241	0.331504423	0.241798815	0.325850461	0.431875193	0.276884162
0.139118792	0.121766383	0.120801793	0.121247273	0.156646990	0.151706100	0.174963045
0.807905368	0.848417064	0.855802307	0.841786961	0.862018079	0.932312550	0.820673336
0.779317954	0.831075145	0.804781813	0.843402854	0.835568386	0.900828753	0.790823207

0.019244446	0.018439365	0.025361652	0.021576546	0.023166304	0.023315110	0.026168214
0.038023093	0.040873113	0.052580466	0.053761781	0.038445860	0.054022486	0.059312750
0.242016059	0.227272055	0.278549790	0.248614531	0.283713109	0.176281378	0.322969387
0.092073456	0.073856992	0.080823128	0.075829761	0.074646030	0.161477361	0.064597879
0.054498851	0.046643400	0.052412910	0.044285614	0.068624178	0.072618862	0.070456671
0.304505979	0.317876626	0.280016953	0.344931114	0.317587946	0.317666302	0.339964897
0.060155359	0.075928717	0.051589792	0.054987109	0.063846253	0.090608872	0.076729779
0.022855296	0.019477567	0.021126895	0.023180449	0.018701530	0.028835683	0.037222687
0.027698219	0.033949107	0.031488201	0.027646551	0.039726165	0.045167371	0.044266752
0.051373547	0.070428260	0.049512148	0.047847015	0.069821462	0.175754293	0.053196800
0.016906273	0.015504951	0.012858141	0.016774507	0.025016840	0.020660727	0.028680585
0.027142271	0.040681781	0.030014529	0.036643224	0.045359384	0.032447997	0.052159600
0.065562495	0.088790589	0.067878465	0.064142823	0.093397040	0.136954959	0.082589928
0.101235804	0.104713221	0.092018810	0.108494938	0.105186949	0.095932993	0.141180425
0.035127791	0.023166607	0.034658841	0.033060061	0.348776244	0.028003712	0.330509519
0.027779963	0.031846238	0.029387911	0.023828493	0.032891857	0.040593156	0.039076749
0.043973133	0.054665372	0.042220101	0.044549447	0.060194509	0.069929399	0.067414835
0.602103622	0.681985267	0.703499619	0.648964829	0.687649221	0.822783384	0.662337324
0.026692344	0.042347112	0.031188365	0.027390216	0.036678150	0.034043976	0.033646169
0.021084239	0.029489887	0.019840335	0.026554559	0.021978378	0.027740357	0.031670804
0.063374771	0.063411435	0.069961853	0.070182589	0.069845171	0.082998770	0.087916200
0.856228532	0.848494212	0.872618617	0.504278008	0.590891324	0.573966932	0.812998253
0.949960333	0.944598201	0.953136955	0.943509195	0.952570998	0.938983711	0.944320263
0.650743351	0.697444846	0.619404505	0.654582977	0.689676767	0.679242722	0.667771328
0.053200246	0.050798017	0.062539384	0.069934361	0.061603658	0.059214429	0.064909632
0.031200415	0.037856517	0.041069587	0.032213000	0.050101464	0.047076267	0.028330350
0.688369325	0.803353294	0.890463270	0.795164693	0.920918522	0.848564548	0.888494309
0.049303500	0.045443328	0.045687501	0.057296878	0.048628863	0.066347966	0.085195448
0.584026351	0.602629432	0.684757244	0.179811757	0.292381565	0.382400701	0.404060065
0.641595523	0.653407457	0.661245105	0.694610716	0.701506847	0.677960852	0.680417763
0.823720556	0.839956387	0.855033548	0.856747579	0.865365659	0.909636607	0.819339625
0.019716306	0.027156138	0.018421973	0.025031831	0.019585968	0.021437012	0.045190387
0.659627700	0.767095497	0.745715444	0.752611409	0.733700762	0.774260690	0.709111385
0.021622401	0.027107338	0.025069069	0.026952076	0.027179190	0.026311293	0.024316563
0.653304721	0.595183046	0.714466470	0.690496175	0.680971769	0.687944544	0.712226093
0.029747512	0.028498932	0.038423730	0.028613958	0.025663025	0.029292124	0.036621419
0.820478769	0.866610523	0.856790974	0.815880452	0.851039722	0.872325709	0.847397535
0.045688587	0.033665804	0.047407280	0.050354876	0.036961711	0.060319182	0.075460052
0.064391644	0.066212678	0.072618100	0.081747546	0.080465973	0.072008705	0.130036139
0.814948153	0.793456114	0.797423358	0.777002138	0.759813689	0.813830321	0.826641200
0.776400578	0.790312598	0.803110531	0.767557972	0.817767875	0.787229583	0.731661402
0.775814371	0.775589713	0.793680224	0.808214955	0.790609135	0.832030318	0.807621802
0.363049123	0.342424818	0.338941304	0.055255036	0.078025465	0.067686442	0.471674735
0.035785018	0.039114822	0.047907119	0.043524218	0.033111687	0.036309661	0.132121789

0.021402225	0.035510180	0.030890430	0.038348753	0.028570984	0.039298033	0.028650077
0.057064772	0.049481346	0.040586482	0.054087810	0.085439339	0.115630198	0.062872176
0.096851435	0.099212781	0.112172767	0.114613788	0.127733174	0.135108612	0.135212444
0.024827299	0.032927164	0.026798535	0.030179965	0.034368578	0.032808038	0.035912287
0.087199033	0.109510960	0.104850126	0.104310075	0.097588451	0.114423910	0.114534703
0.063527979	0.095959709	0.076208936	0.069042702	0.072773083	0.119148574	0.096540584
0.041891937	0.039496712	0.041819297	0.042730221	0.038841785	0.048190627	0.051963768
0.122345459	0.145040490	0.115738820	0.124813356	0.136517674	0.157224078	0.128409883
0.036749122	0.055393447	0.042897569	0.038463992	0.045237809	0.044426478	0.053461645
0.401318410	0.437055310	0.506696771	0.482429730	0.452888248	0.514394464	0.460910900
0.026027207	0.023097421	0.025630202	0.025580779	0.027434095	0.037027748	0.032334528
0.193143404	0.221182795	0.187818424	0.230391807	0.271285014	0.186564630	0.273435166
0.708625403	0.765805457	0.620954060	0.692444470	0.771619041	0.799747485	0.673178055
0.060158448	0.085525287	0.063816099	0.069873913	0.084882402	0.122715010	0.100847249
0.493677972	0.438137180	0.481692646	0.502433115	0.523092136	0.609132846	0.463682861
0.073304916	0.068131012	0.078334297	0.060893987	0.058841786	0.088192286	0.080277031
0.026493488	0.029542633	0.026983783	0.029582061	0.029117399	0.043149944	0.032537947
0.050924317	0.064245812	0.071795197	0.063816797	0.072932057	0.066852009	0.059935456
0.131318546	0.141484648	0.149720739	0.154930326	0.168630476	0.136009452	0.209390375
0.031087214	0.026691296	0.027323347	0.023125368	0.027085999	0.034035684	0.034685891
0.831619881	0.882752757	0.883072127	0.899761642	0.926182120	0.939228450	0.824676001
0.020905438	0.017875447	0.017632120	0.019034008	0.021142625	0.032009491	0.026411305
0.030660085	0.036562405	0.032003321	0.039155336	0.036052747	0.039541929	0.049455298
0.456950901	0.390522624	0.394771581	0.380342201	0.491836410	0.586096671	0.410049125
0.027120674	0.041244028	0.036296101	0.032301907	0.044986993	0.037234128	0.039961960
0.400516081	0.429265552	0.463474510	0.399364461	0.416282685	0.515357606	0.501570660
0.047071759	0.050554875	0.046923273	0.051577888	0.050567505	0.085323150	0.067541953
0.149956253	0.173186192	0.132958288	0.197085683	0.188106780	0.139648998	0.261671526
0.888609407	0.932209418	0.915617862	0.906842533	0.905729172	0.937038261	0.905782869
0.026318828	0.026115843	0.020666416	0.022048980	0.023421528	0.026096306	0.039471974
0.709554678	0.755178345	0.725273222	0.731912660	0.704513274	0.772553877	0.725685200
0.028205432	0.030321026	0.045743075	0.065816730	0.074019920	0.028439220	0.096407370
0.037236234	0.029535500	0.036668346	0.029156778	0.039084864	0.042448444	0.035941953
0.468869674	0.542324876	0.495360241	0.505569855	0.564343599	0.533316419	0.513442696
0.940653463	0.927845243	0.940347645	0.951911578	0.944308673	0.860282359	0.924096042
0.038416566	0.058949213	0.032943095	0.031073532	0.139453807	0.070807140	0.047634242
0.190322553	0.158368557	0.132624089	0.127629283	0.296346791	0.284421151	0.169964723
0.032079953	0.033679622	0.042000612	0.030985374	0.037995260	0.046560977	0.063875521
0.057299583	0.098842612	0.058907511	0.054509886	0.065540022	0.203296434	0.059541909
0.250639841	0.315291791	0.303754817	0.307269468	0.344759136	0.332817316	0.328239406
0.015153789	0.015886379	0.013623229	0.020285292	0.017470970	0.018435446	0.022721007
0.269354617	0.338808644	0.424201798	0.423874897	0.526216092	0.439507486	0.309097159
0.250295353	0.273304697	0.274897391	0.282131150	0.277304280	0.301180042	0.344256805
0.182261102	0.140786992	0.149426757	0.167939782	0.179931519	0.177680775	0.211914940

0.567455321	0.636146100	0.644583706	0.549318117	0.518673954	0.675000262	0.596595728
0.441400958	0.454121275	0.406034460	0.392430404	0.520564441	0.539819239	0.441427589
0.289734123	0.287458359	0.300735306	0.304297607	0.300661873	0.337799478	0.346050032
0.067843984	0.072726414	0.075628417	0.074888860	0.067225747	0.066604085	0.080009519
0.225368770	0.259989442	0.121410949	0.230518150	0.237828127	0.193995580	0.251717774
0.507993922	0.452662352	0.514132055	0.516065463	0.518117527	0.553211070	0.518270144
0.044640233	0.045622748	0.035519948	0.049811562	0.060056795	0.049232785	0.058817203
0.093775801	0.087062798	0.078971273	0.101554311	0.108613014	0.087120001	0.123219436
0.027839817	0.026801397	0.029798650	0.027614841	0.025556392	0.036058904	0.032189960
0.052731951	0.046223189	0.054507885	0.055831487	0.069917001	0.062042448	0.069372322
0.881951791	0.866020486	0.890576628	0.901609874	0.888711496	0.862071284	0.900808817
0.889307160	0.893217669	0.856191309	0.887740419	0.886427594	0.900792723	0.878338888
0.035607804	0.035344444	0.034953195	0.033081739	0.041095993	0.042168263	0.061805500
0.751813228	0.756279009	0.750578544	0.764207320	0.712689920	0.832557502	0.733783667
0.028893274	0.029781695	0.024286411	0.032119636	0.034773490	0.032906590	0.042919550
0.020772140	0.023181250	0.021482306	0.021701535	0.020346969	0.026331945	0.035180759
0.061192773	0.061309784	0.079738839	0.079354397	0.068592992	0.082876420	0.164638888
0.059363496	0.067123154	0.057226445	0.053548865	0.076197058	0.092175646	0.071338592
0.312107739	0.291872632	0.278203557	0.245806507	0.337038517	0.395368316	0.246441620
0.080939118	0.076408948	0.099016208	0.079432884	0.108662277	0.091562225	0.128735855
0.928288561	0.929470157	0.938169948	0.935448802	0.939798708	0.942085857	0.938679924
0.865106373	0.873745249	0.894519601	0.866723426	0.890539269	0.873717263	0.841607920
0.547360959	0.510201366	0.585917324	0.633709425	0.572875596	0.558460815	0.565926787
0.026203583	0.029126506	0.034057288	0.024928615	0.028773693	0.037068895	0.047298687
0.573535279	0.644209738	0.642418315	0.596366624	0.606262014	0.630118874	0.665042058
0.027473780	0.028761934	0.029996966	0.031186479	0.034341792	0.038934189	0.035843966
0.113423124	0.096077780	0.123191738	0.106323229	0.109570694	0.180423088	0.153440402
0.017507061	0.015679139	0.017328840	0.022535520	0.020900235	0.017371533	0.025295976
0.032757206	0.039504035	0.030646896	0.034644262	0.038129573	0.044484932	0.050341110
0.045340515	0.066778263	0.045131720	0.049288774	0.063995684	0.086574049	0.063847872
0.864509590	0.845544747	0.869322239	0.846813400	0.848798490	0.896074330	0.799271686
0.926141545	0.903226291	0.935750321	0.947810996	0.929787224	0.819037221	0.921408089
0.047637130	0.050736418	0.054188183	0.044680508	0.048491958	0.069701549	0.069214324
0.030442693	0.035040958	0.027995118	0.035687975	0.038448604	0.051565584	0.043725316
0.030642623	0.036905388	0.036819637	0.037383582	0.039037785	0.044518920	0.052954521
0.763314160	0.810884870	0.796985257	0.775458318	0.836613763	0.924824462	0.777135222
0.885788178	0.907394838	0.915096813	0.922553506	0.927755148	0.927352235	0.908375816
0.479968325	0.436361348	0.447715546	0.446615440	0.462248504	0.511270057	0.507533929
0.423538287	0.401162935	0.381575900	0.466622315	0.426155042	0.373266968	0.439584243
0.040151624	0.041972050	0.034658978	0.045629188	0.049540629	0.047361079	0.062350024
0.214424656	0.232644185	0.207650211	0.246760601	0.263936595	0.204804136	0.277272091
0.033367535	0.033930073	0.039538063	0.037526167	0.035266233	0.066786195	0.032518434
0.474291637	0.553505215	0.560721600	0.533623885	0.545299050	0.615329905	0.552504565
0.029654304	0.025314665	0.030714746	0.025778181	0.029767494	0.034296575	0.045865987

0.645014033	0.738083846	0.656502456	0.679806770	0.689589682	0.708678615	0.690084221
0.819878082	0.847450045	0.859231713	0.882858076	0.840799732	0.904171884	0.869547881
0.031517935	0.039991902	0.042358494	0.029964930	0.041921581	0.091988307	0.041531417
0.464422586	0.499021834	0.482397934	0.486085006	0.525842686	0.522126310	0.467717150
0.730949432	0.728970415	0.801039693	0.791189349	0.760571520	0.807154572	0.732126012
0.050959221	0.036110109	0.040203183	0.045082214	0.037730258	0.059927120	0.047455960
0.070655366	0.066592076	0.088076299	0.103439783	0.106162951	0.100319845	0.122492237
0.745233876	0.801866688	0.782962587	0.794971138	0.759781977	0.804563374	0.779110865
0.017257028	0.016190809	0.015018365	0.015692794	0.019667921	0.022014677	0.023438615
0.847253662	0.866417233	0.836306982	0.817538389	0.874414851	0.910663432	0.817127502
0.065654873	0.069193956	0.061996537	0.057933770	0.078470293	0.095627032	0.100642068
0.748811501	0.760472099	0.779501043	0.794258697	0.790282257	0.802037250	0.789406818
0.773380678	0.783239800	0.747421916	0.791244624	0.828018690	0.727185912	0.819228462
0.657864141	0.629638290	0.610264055	0.583564326	0.741208151	0.811481270	0.625723491
0.401533057	0.438553405	0.888633849	0.865680421	0.905869290	0.476742778	0.880985435
0.043948821	0.037269715	0.037599253	0.041243044	0.041873559	0.049512737	0.050504473
0.099854591	0.089743267	0.108340959	0.099628868	0.111462199	0.128417852	0.164633872
0.864494172	0.869499524	0.855694449	0.869742475	0.868155871	0.868192568	0.850372012
0.057516873	0.060073999	0.063280855	0.061400748	0.067954976	0.077273824	0.066763663
0.023300135	0.018374484	0.021576353	0.023191407	0.021092811	0.031796313	0.035081175
0.864488376	0.899440945	0.883263287	0.890231091	0.898730503	0.898805127	0.871138190
0.975514939	0.974706019	0.974587851	0.976914319	0.965958048	0.970079201	0.961402938
0.049113513	0.043159155	0.039208311	0.043230788	0.047784036	0.062698170	0.039532740
0.892197602	0.892906219	0.874353707	0.911228387	0.911691839	0.909344250	0.879732363
0.023111604	0.026761260	0.024425151	0.022722400	0.027286699	0.030661921	0.035167582
0.076201332	0.092753052	0.044940423	0.094885482	0.153627335	0.127891230	0.154239718
0.048665816	0.059557493	0.055168979	0.056480538	0.044446577	0.065043324	0.059368128
0.965250535	0.956774447	0.968283290	0.967209163	0.936891048	0.955899377	0.945541177
0.804321213	0.803247652	0.813564898	0.838959906	0.694326976	0.869662103	0.741736779
0.336901199	0.389162950	0.376559587	0.422655482	0.383695334	0.369042325	0.392695502
0.061014358	0.054987713	0.055506242	0.049095616	0.064335973	0.085009042	0.067893049
0.638521141	0.689240433	0.656789115	0.700220394	0.751075475	0.724708962	0.670776613
0.626661520	0.620397208	0.646269572	0.664085356	0.698502292	0.633723906	0.622860090
0.457344320	0.400386044	0.466534423	0.446291676	0.501288740	0.547619136	0.455144012
0.034227195	0.025204194	0.031247715	0.035263326	0.040664427	0.040219201	0.033241201
0.074521620	0.081436998	0.074484787	0.064653014	0.088913447	0.097133903	0.169493382
0.043953081	0.076696206	0.068712663	0.062752583	0.035848245	0.059980807	0.074963489
0.026894654	0.036044114	0.028686812	0.022718166	0.028172750	0.036819491	0.050275404
0.426237767	0.436583993	0.416341492	0.451184111	0.482145813	0.533556306	0.443635358
0.866581101	0.897682509	0.914206137	0.908441055	0.898805317	0.891318473	0.887043474
0.046574649	0.051091922	0.040049636	0.048556677	0.053365537	0.053344482	0.056272239
0.611205799	0.664035764	0.676076268	0.670281685	0.638291882	0.738493034	0.645856114
0.027354400	0.032426187	0.040734656	0.030003913	0.036650896	0.048737715	0.047440956
0.283087643	0.311884709	0.275920926	0.263646240	0.318785865	0.343737147	0.316352762

0.131646044	0.184841069	0.108830498	0.137921197	0.209970228	0.151047800	0.194418599
0.019987625	0.022304173	0.026910792	0.024075579	0.027532787	0.031420907	0.030784196
0.845074304	0.874786940	0.902355760	0.896623911	0.884399089	0.888775551	0.893623019
0.824813365	0.818738981	0.839366497	0.856273250	0.828449066	0.902594750	0.828463451
0.091491872	0.167335107	0.144988260	0.135486786	0.146694220	0.257091268	0.151541721
0.059198071	0.053761835	0.055928437	0.053719425	0.058585330	0.073716115	0.056862941
0.038896110	0.037450486	0.033567071	0.025341766	0.034115856	0.043257907	0.041410436
0.027659629	0.017229553	0.033371161	0.029528569	0.024469259	0.038768777	0.040800696
0.839029278	0.869817146	0.864456995	0.852199127	0.846617112	0.882197424	0.861875317
0.374609386	0.397534478	0.396868487	0.394702450	0.434149447	0.386889284	0.440650100
0.041999656	0.034754418	0.035591757	0.036728307	0.037542785	0.087265513	0.036897943
0.311680849	0.373332065	0.339095460	0.393819973	0.426611535	0.263136655	0.415466774
0.052752951	0.054991133	0.048819716	0.073367562	0.082905502	0.071743461	0.075951855
0.136766773	0.139551881	0.151982863	0.165425038	0.164736823	0.125699264	0.219873251
0.561523594	0.583730071	0.577864777	0.609037609	0.591544408	0.601707303	0.623412946
0.650267897	0.689579697	0.737059834	0.641322755	0.638142857	0.682157058	0.666131011
0.191347551	0.360878439	0.295551724	0.350321988	0.313653323	0.318645113	0.436110428
0.116196612	0.123161253	0.121048870	0.143432903	0.161161661	0.155502547	0.232985938
0.053374201	0.061200815	0.084423196	0.080288497	0.145207222	0.084781997	0.128686097
0.034152602	0.044517878	0.050996297	0.064166632	0.077413184	0.050270513	0.053745770
0.015883670	0.017232258	0.020778967	0.023812698	0.019640254	0.026136149	0.025474885
0.399057758	0.452768481	0.397753887	0.429298289	0.490951015	0.317475473	0.452843040
0.053119954	0.056862969	0.045728020	0.060270903	0.082713325	0.083058456	0.061982560
0.033255049	0.045997057	0.046641463	0.039503720	0.058092803	0.073519583	0.057146575
0.025098325	0.033590319	0.035136839	0.022811849	0.026242750	0.047315023	0.040511476
0.059804901	0.053706665	0.065363025	0.082193531	0.083112895	0.084204298	0.108401461
0.366332313	0.322016880	0.352829245	0.346328330	0.364629458	0.384594727	0.377186057
0.065909939	0.072560750	0.087809625	0.112543620	0.088085388	0.107990281	0.070954821
0.045263342	0.056721023	0.045674021	0.062592309	0.058254951	0.056077890	0.049077995
0.094769071	0.096404656	0.095303882	0.108613177	0.149838848	0.077443760	0.127366993
0.028975960	0.030727889	0.028954452	0.027497303	0.028953951	0.052765921	0.034966016
0.056466297	0.073288365	0.063030320	0.105490135	0.089293630	0.054573674	0.091328526
0.365802695	0.359763359	0.368958893	0.353773306	0.316814434	0.411602170	0.448830655
0.577413674	0.574278743	0.579271256	0.555244523	0.643017772	0.728020484	0.540838911
0.407209905	0.411787263	0.445114685	0.463233821	0.417547790	0.468263573	0.472695418
0.831257609	0.888726312	0.845503451	0.816084746	0.839307091	0.903587782	0.842926754
0.055988100	0.052796541	0.064137377	0.050736313	0.052236568	0.064565144	0.105856850
0.055209785	0.056574363	0.053989448	0.051510866	0.057658227	0.086377001	0.078757614
0.021458065	0.022859877	0.016842660	0.022017731	0.025677818	0.034804098	0.024836544
0.172697115	0.179149331	0.166286186	0.181590161	0.155597651	0.195658144	0.237940280
0.882802893	0.907898636	0.895551691	0.900441512	0.900943843	0.908179388	0.891333991
0.018639978	0.018421429	0.017565187	0.020505884	0.018166448	0.025780853	0.025866707
0.015657066	0.017060071	0.023028492	0.022311721	0.019779125	0.028091981	0.031728233
0.044701269	0.058631339	0.051946574	0.036540491	0.071626092	0.070191591	0.049268223

0.047942830	0.046520775	0.047010603	0.048330685	0.046981024	0.070159357	0.055350719
0.028947098	0.024717478	0.027679063	0.030211607	0.025987017	0.034693940	0.052085914
0.028366468	0.020857572	0.024393874	0.028888953	0.041618595	0.038996785	0.045035948
0.798774166	0.761885855	0.762353260	0.812841661	0.848078291	0.863148385	0.796067773
0.040659294	0.041926614	0.047118057	0.037741441	0.047095963	0.054323044	0.043563256
0.807439487	0.822959486	0.836528606	0.783460730	0.847930242	0.860446835	0.776274031
0.799762939	0.805290462	0.823924844	0.844518067	0.854065936	0.888186924	0.780397102
0.089430336	0.088448599	0.071901444	0.068610632	0.066421516	0.129866353	0.112907206
0.680773355	0.703049997	0.725340174	0.708764588	0.712077665	0.851851676	0.688880852
0.027217838	0.049186840	0.024160941	0.027989992	0.030419660	0.031831255	0.049377368
0.725881586	0.636321566	0.585257431	0.454820735	0.654622143	0.458424282	0.552283642
0.036620213	0.022027773	0.024074682	0.030633007	0.032826301	0.034762880	0.042120645
0.057642643	0.053834085	0.047852969	0.049657466	0.046612861	0.056208934	0.075966294
0.060198894	0.058058605	0.066268145	0.063109573	0.079002609	0.045315992	0.103344300
0.695741138	0.727179699	0.744586638	0.725601810	0.758360126	0.796108384	0.712917535
0.019386334	0.016688397	0.022073741	0.021294136	0.022060321	0.031298406	0.026122780
0.774688168	0.815709585	0.823484779	0.853614426	0.782357580	0.860142671	0.858858152
0.867709441	0.895987004	0.838900258	0.889794642	0.873570151	0.899297179	0.849587061
0.034266049	0.039855076	0.035565522	0.032756905	0.043477334	0.053749617	0.051596840
0.395505357	0.352431263	0.335640150	0.384404736	0.341327985	0.504398529	0.375053456
0.037981132	0.045066071	0.039553220	0.051144860	0.050347057	0.069974060	0.048953305
0.043079626	0.060163617	0.045287731	0.032044485	0.044503324	0.072556429	0.063899562
0.897698577	0.935399197	0.920413611	0.936559567	0.930577359	0.945931784	0.921698928
0.027629460	0.051073836	0.024610106	0.034579523	0.031214928	0.053269493	0.038531185
0.665718411	0.727431430	0.691928716	0.772499125	0.840778690	0.726551436	0.761035787
0.196092019	0.183026454	0.183081895	0.207306429	0.188626920	0.219746078	0.289332561
0.161088991	0.178897473	0.164050813	0.175426633	0.195066162	0.198222594	0.209722986
0.104586356	0.115196406	0.105185614	0.112347992	0.088330556	0.085145885	0.179804966
0.228403145	0.297582185	0.344135128	0.251953204	0.417605472	0.423287192	0.232678721
0.665090385	0.717918742	0.739543861	0.631905658	0.588429100	0.694618133	0.679383475
0.033305107	0.034429775	0.045598688	0.049616609	0.032047716	0.058906050	0.043006509
0.385109075	0.491775795	0.457212045	0.455409260	0.400705299	0.420107488	0.461285983
0.049153128	0.048349950	0.055477900	0.056366662	0.055468130	0.078814701	0.076322351
0.044926839	0.040726888	0.039645803	0.043665659	0.052608982	0.048218629	0.056604234
0.176916929	0.130071054	0.150170580	0.150234342	0.150681921	0.190414395	0.202669221
0.272953809	0.229813129	0.236746559	0.221385716	0.302360883	0.162425464	0.253744177
0.729823436	0.782707928	0.751704373	0.735947147	0.767381569	0.863085689	0.726339182
0.897351608	0.920328902	0.899954200	0.895821006	0.902523422	0.882193954	0.867903701
0.027589724	0.031667468	0.026222734	0.026492441	0.027713936	0.041004704	0.043365042
0.440276100	0.514320077	0.447488424	0.469977571	0.532856252	0.648774980	0.459729529
0.082930010	0.079199208	0.073677198	0.074152107	0.080977084	0.094840812	0.132212211
0.779282051	0.796905800	0.810093510	0.838193890	0.807865597	0.821123451	0.825087482
0.194717345	0.170239263	0.212348764	0.266074568	0.313406567	0.163973942	0.237353461
0.673803940	0.710917638	0.700223454	0.597610237	0.709672765	0.786137825	0.677864585

0.267490538	0.285673662	0.286969329	0.280030172	0.277297900	0.293188696	0.281017196
0.885631384	0.886131901	0.886794150	0.885098086	0.918577422	0.905867527	0.875859623
0.803231257	0.833227677	0.795537002	0.843950895	0.829135362	0.863492350	0.792580795
0.850661866	0.851796838	0.855459534	0.833981116	0.825860430	0.854391612	0.847051416
0.032038559	0.030153194	0.031167795	0.028049094	0.042073626	0.038424591	0.050115374
0.145704514	0.191185856	0.233515762	0.174114775	0.211659435	0.208590264	0.152976779
0.071697725	0.077866211	0.075683919	0.086234272	0.084041604	0.088571084	0.105301289
0.038986820	0.031278178	0.050690198	0.028554292	0.059716991	0.038263008	0.050761043
0.881321393	0.899787954	0.903036110	0.914738237	0.917040694	0.910168132	0.894508641
0.288465817	0.346415110	0.325366543	0.469569080	0.317875040	0.319413590	0.364630645
0.054247774	0.055104674	0.055833400	0.056906974	0.055719635	0.076186808	0.064083187
0.210856158	0.142779670	0.169769224	0.145409233	0.230426771	0.211307547	0.205806592
0.025024750	0.023316804	0.022051065	0.025019619	0.023970328	0.033037605	0.040440190
0.719548284	0.670810822	0.670988557	0.689232419	0.669917608	0.746888046	0.683299053
0.147124502	0.134836918	0.139475384	0.189795308	0.210482546	0.115095340	0.191500627
0.673139566	0.645210719	0.608520857	0.726724727	0.658022133	0.815278433	0.677921927
0.032611924	0.023264713	0.031466788	0.035773628	0.029082288	0.050595142	0.058203544
0.192611039	0.178715750	0.161708455	0.170307773	0.171767019	0.265694574	0.192765341
0.069451919	0.096433568	0.080581401	0.059895981	0.072355201	0.074020042	0.142856997
0.027192518	0.023482723	0.021681312	0.026822053	0.038778419	0.075588533	0.030291188
0.969440343	0.969814101	0.972496410	0.969797136	0.971026472	0.954089289	0.957993693
0.164631125	0.151954042	0.148985742	0.150164565	0.173861271	0.184074789	0.199921557
0.532757761	0.509787871	0.584982896	0.555822859	0.539593852	0.577857666	0.567997354
0.765726375	0.794150410	0.742717005	0.790591481	0.785328293	0.888605622	0.762671001
0.161977866	0.147041206	0.149061364	0.206859615	0.281500293	0.096300131	0.193080145
0.227647121	0.260986327	0.254041162	0.235721747	0.317039326	0.209327637	0.315541200
0.064353984	0.056138511	0.065316996	0.056541129	0.060001257	0.083808445	0.072207774
0.021772078	0.020486869	0.023066511	0.032299910	0.026815115	0.081528023	0.038546869
0.086319854	0.125765700	0.083731223	0.069612930	0.221336865	0.208441916	0.076852859
0.874918060	0.907570199	0.886197548	0.900010405	0.900726267	0.907421532	0.867116567
0.049240139	0.064962173	0.053105858	0.055650527	0.055806619	0.077323275	0.056918962
0.054348092	0.056877007	0.069887212	0.053635203	0.054165992	0.057805274	0.068554450
0.647753926	0.738191842	0.745402714	0.787607435	0.746758646	0.756787102	0.757079967
0.072060338	0.075511371	0.070576191	0.069410855	0.079198615	0.099325214	0.103322587
0.522062621	0.563194921	0.508358728	0.526852124	0.505031522	0.653607079	0.486134936
0.444161945	0.401427362	0.594075033	0.476001046	0.537946020	0.520017365	0.584109613
0.894486710	0.893972264	0.897429984	0.899579430	0.854129466	0.805928869	0.866788346
0.148155870	0.130157680	0.145470166	0.139377773	0.181271857	0.181020481	0.159148405
0.617494140	0.636439225	0.687064639	0.634209385	0.653903567	0.673806401	0.652431059
0.179104112	0.148595011	0.194573228	0.151813685	0.149560021	0.191290525	0.188222443
0.356065343	0.378252861	0.378815953	0.354519313	0.428069712	0.444851580	0.410499740
0.641986561	0.620787224	0.635619613	0.614833119	0.630563565	0.577669394	0.592592263
0.774926026	0.787560804	0.807918423	0.792280402	0.769864215	0.829138346	0.796390639
0.277796566	0.244236286	0.289220128	0.290008251	0.281524915	0.283037790	0.348691490

0.019206641	0.019118670	0.019433598	0.021418172	0.023278746	0.021583023	0.029513644
0.073198114	0.070560859	0.075886215	0.095227586	0.162484355	0.062747968	0.052525857
0.114227845	0.129628943	0.097841449	0.083821316	0.119766897	0.296690307	0.128175377
0.556915825	0.679573534	0.614053566	0.653425995	0.649366232	0.662350159	0.605053720
0.414968984	0.491562669	0.435368496	0.481372925	0.543812269	0.442353482	0.477339762
0.216444209	0.194499754	0.183155629	0.249237849	0.215127595	0.217671828	0.257959853
0.849034927	0.827040909	0.839476752	0.873575693	0.869903456	0.665918029	0.825618724
0.400458389	0.368793455	0.336956914	0.325566184	0.405351396	0.528491196	0.350894108
0.048583487	0.048037948	0.054335050	0.034508012	0.050831251	0.090440279	0.061035257
0.768090442	0.787217321	0.806632866	0.810495108	0.821335395	0.854936055	0.754593018
0.784963616	0.783578383	0.802966568	0.827321078	0.805077673	0.844439503	0.829625875
0.227072654	0.195798079	0.206808972	0.207712248	0.217902320	0.222443226	0.292690021
0.595965330	0.762750123	0.672110864	0.621483920	0.760307913	0.692522170	0.631193857
0.816493865	0.724603091	0.870077213	0.910090114	0.782621832	0.879388163	0.790536374
0.030304824	0.031933376	0.030887702	0.035090438	0.026704391	0.048351228	0.046629049
0.554886580	0.597849861	0.623291941	0.540322128	0.517160352	0.569329772	0.588363252
0.042769417	0.042994593	0.034949929	0.032172496	0.059809832	0.046994675	0.060479707
0.064444685	0.065092460	0.063500635	0.068969346	0.065310280	0.073477326	0.074639394
0.026311771	0.024655798	0.027671439	0.023848567	0.029091135	0.029066422	0.028462478
0.629018167	0.698319545	0.692373969	0.582425224	0.633581113	0.705553159	0.645583720
0.951500288	0.944723103	0.952776525	0.961818459	0.960001879	0.904575426	0.935956219
0.098372248	0.074204723	0.079430671	0.064388017	0.096221839	0.134475096	0.117796881
0.587204767	0.558110451	0.612507910	0.716505936	0.568628093	0.623900428	0.668225236
0.176773212	0.178676352	0.166905339	0.217070176	0.195085248	0.141351588	0.198457112
0.492906327	0.471800459	0.502057951	0.461473409	0.465242516	0.572685483	0.511268622
0.025868836	0.027749752	0.024330930	0.022551420	0.027829758	0.034433276	0.035207559
0.777049339	0.817126325	0.798323547	0.833116130	0.915968236	0.902657454	0.797869700
0.030036317	0.031645466	0.024044142	0.034348541	0.039668394	0.048344424	0.037439635
0.547196040	0.617895510	0.601307222	0.595236311	0.584779684	0.677652547	0.641442953
0.060674090	0.042016920	0.039284299	0.040546195	0.083098429	0.040187177	0.073514433
0.273768312	0.263732571	0.317058651	0.335871165	0.339654971	0.273138838	0.380658103
0.443895208	0.265994808	0.366146957	0.359822481	0.268842787	0.256607721	0.298560259
0.046710866	0.051224548	0.037916592	0.043353615	0.042523947	0.097372119	0.063694050
0.029728969	0.030538897	0.027938034	0.025503377	0.026149074	0.048835041	0.036050759
0.267758019	0.277899676	0.270772044	0.270283410	0.348347789	0.343474162	0.320022141
0.019381429	0.017082664	0.018198173	0.017763340	0.019272695	0.022935740	0.032109809
0.678487385	0.724814574	0.714848838	0.762329650	0.743792153	0.767796778	0.727504141
0.028242105	0.028285982	0.027738591	0.026846831	0.023990100	0.036038872	0.043392946
0.678226710	0.633559070	0.652395471	0.772438360	0.784202785	0.774468715	0.715850877
0.203818110	0.134610375	0.176721348	0.196324042	0.260038259	0.133847572	0.194223586
0.029153199	0.031620432	0.028367236	0.027788501	0.032505874	0.038040216	0.034530438
0.021850152	0.022599096	0.024721978	0.023009764	0.026609772	0.027456785	0.031820545
0.073559015	0.076194841	0.080622985	0.080259958	0.077790062	0.095782886	0.098173891
0.775888001	0.736235918	0.784604293	0.741788394	0.777729240	0.799925892	0.749618392

0.457068513	0.466889169	0.476442122	0.404102612	0.442049492	0.419329512	0.474180152
0.864848815	0.890673737	0.840813623	0.882840347	0.885305360	0.898287198	0.872555582
0.499088208	0.468685391	0.505628748	0.469560627	0.517762318	0.602567302	0.512589628
0.485612789	0.495079077	0.486577911	0.494423887	0.499211801	0.528272348	0.509157385
0.071279751	0.037157876	0.045563055	0.043108989	0.054733763	0.046666608	0.056763489
0.508163301	0.544920956	0.551459916	0.501725108	0.482052586	0.586164197	0.667873665
0.542137129	0.520457335	0.607889591	0.509444988	0.586409549	0.728160335	0.544389690
0.743347628	0.784335532	0.763489023	0.734622685	0.854812276	0.903974169	0.736088732
0.329009496	0.379878816	0.381093101	0.395886576	0.429299759	0.461996536	0.415792653
0.026461933	0.030017440	0.026835505	0.025831454	0.038444127	0.034018118	0.036960272
0.070688864	0.065778631	0.075493955	0.119832170	0.065293230	0.084460088	0.139656539
0.055632523	0.042149376	0.056927367	0.044758113	0.059579125	0.036923450	0.090951992
0.770903237	0.748713316	0.785066669	0.774857100	0.740903248	0.679346661	0.685829278
0.160243468	0.140611283	0.145481211	0.174267829	0.192475378	0.165855160	0.194563424
0.711863693	0.748410957	0.775426886	0.770032492	0.757636297	0.739527541	0.762617894
0.143841488	0.151162745	0.136698421	0.183203785	0.242129246	0.118560566	0.173419553
0.016817228	0.022462789	0.022266932	0.018935666	0.019642627	0.021491020	0.028743258
0.304720151	0.330086343	0.308811534	0.339804623	0.363616904	0.333589191	0.371667185
0.363844765	0.345143198	0.326100008	0.342066695	0.365372420	0.399430604	0.289264950
0.588872050	0.631128241	0.777723809	0.714033213	0.852854935	0.789040931	0.743102336
0.880328514	0.811597156	0.888923054	0.860010639	0.853689198	0.693975826	0.822856009
0.038633970	0.031805282	0.031051200	0.039801069	0.034539483	0.057010711	0.050601729
0.030949009	0.046904734	0.040525850	0.038390967	0.036991389	0.040008684	0.048388441
0.064459697	0.069703752	0.069914782	0.056893008	0.057975765	0.073633142	0.106160727
0.619254705	0.752631267	0.703531699	0.748336393	0.670940888	0.564219069	0.708473214
0.449263668	0.497539260	0.440985556	0.469866071	0.494898884	0.382061026	0.441744480
0.149527930	0.178218526	0.118076716	0.128451392	0.197168930	0.195871354	0.157475142
0.136090702	0.095243690	0.104811003	0.081810167	0.104608833	0.117765570	0.150477735
0.035306226	0.035233046	0.043029482	0.038303426	0.046968349	0.062469450	0.045440974
0.176676201	0.195412737	0.211700023	0.181852982	0.193840322	0.200513297	0.231454123
0.071043604	0.087945069	0.084262385	0.070254541	0.081055849	0.101650097	0.102501521
0.457207954	0.490799015	0.476840932	0.525326512	0.514532679	0.491758016	0.486519410
0.033443675	0.035765580	0.032994502	0.037263720	0.034321097	0.042666218	0.037294655
0.488781442	0.362352074	0.474389852	0.492959215	0.512441506	0.480033218	0.579271914
0.886939558	0.901217491	0.916635111	0.922457994	0.922674604	0.920424912	0.900900555
0.890186195	0.908458125	0.897503214	0.890226093	0.921813773	0.925151626	0.888687021
0.362596329	0.373457171	0.394519404	0.417102670	0.356401905	0.396661671	0.448099706
0.022264778	0.016839006	0.016573600	0.015031268	0.020922321	0.019018097	0.043867777
0.866736338	0.878042568	0.892087423	0.880866515	0.873738850	0.875145450	0.838128141
0.031385292	0.040333974	0.036675749	0.042745347	0.042897771	0.040199966	0.048091736
0.814836378	0.819613914	0.869063744	0.824796841	0.793286780	0.874569598	0.831880664
0.657496549	0.640868384	0.638853009	0.654749272	0.616673712	0.767039698	0.636125478
0.061499444	0.077342518	0.051649801	0.052329586	0.081978820	0.123340419	0.081415188
0.038938883	0.038953953	0.032421771	0.033031247	0.037803735	0.059716518	0.036392788

0.043592167	0.050657571	0.043141038	0.042217446	0.038207081	0.057186942	0.053288473
0.517748181	0.682558677	0.581090019	0.536700752	0.693062066	0.559687348	0.608184818
0.964103399	0.967616891	0.965701387	0.968552322	0.871760699	0.961226010	0.953139473
0.025039439	0.032868263	0.027842687	0.023236165	0.024093352	0.021860633	0.039218866
0.037448304	0.030446851	0.030183892	0.028344618	0.037401769	0.047392214	0.063367542
0.883993442	0.797236505	0.876866345	0.890477295	0.834096824	0.616264338	0.836315869
0.037052941	0.035161935	0.052627080	0.051477671	0.062976812	0.043664013	0.056461171
0.617577978	0.570839588	0.673568898	0.635964320	0.643314279	0.826375982	0.654394414
0.029661890	0.041095526	0.050945048	0.048136885	0.046646949	0.074105929	0.040480281
0.033484824	0.026347335	0.036468499	0.032378410	0.046634009	0.036042268	0.039175309
0.360700004	0.380531356	0.378670304	0.386759474	0.394379954	0.455787662	0.408836177
0.047713538	0.049076612	0.050003241	0.038873164	0.048869323	0.045786361	0.071436496
0.412046082	0.417138274	0.414888651	0.437790714	0.432007604	0.411602944	0.442165622
0.112057812	0.136418258	0.118465044	0.105598285	0.141398329	0.209090364	0.100935355
0.130915293	0.125558529	0.117122816	0.129337850	0.129988140	0.162249046	0.153242874
0.311725592	0.372251722	0.376652499	0.365866656	0.401690947	0.413643045	0.403330333
0.642841000	0.758060625	0.657772569	0.710056069	0.624101535	0.488878911	0.535251701
0.791825196	0.837063403	0.840868480	0.852027880	0.833359313	0.908080342	0.837820362
0.253407889	0.303135656	0.254914704	0.279848200	0.325885868	0.207658184	0.332017731
0.738874642	0.766136401	0.812022703	0.817138354	0.751263904	0.786272476	0.823893677
0.051321874	0.045436656	0.052321274	0.047029463	0.059903192	0.075452001	0.039448436
0.025100424	0.023659879	0.022684756	0.024452024	0.031930839	0.031189793	0.035076025
0.059358833	0.079663319	0.057553416	0.047019284	0.121124532	0.125043635	0.078260887
0.043083399	0.049313262	0.038713238	0.041353015	0.041701937	0.054882512	0.053777259
0.036513476	0.052634880	0.034940483	0.034055478	0.037007376	0.091784131	0.041167230
0.192099400	0.277921035	0.256801350	0.269033401	0.322184548	0.238034420	0.317132041
0.064727237	0.080502334	0.073538123	0.063682953	0.081265371	0.111781774	0.088996105
0.026012288	0.033341822	0.032812956	0.021633155	0.046965381	0.042122872	0.035716792
0.096647953	0.169636559	0.174903386	0.153524941	0.168022074	0.126110274	0.249078980
0.689490682	0.715520765	0.615624556	0.598546302	0.606464121	0.640732683	0.663023820
0.042322542	0.038086927	0.036769964	0.037251976	0.035335327	0.035616961	0.049598351
0.912974077	0.929380736	0.914988115	0.915118933	0.920440597	0.937921310	0.921142388
0.082680642	0.091900732	0.079254551	0.063119302	0.087994590	0.181225240	0.092864690
0.040527557	0.035476336	0.042394179	0.043986761	0.031692116	0.031685461	0.052245504
0.596925010	0.604934648	0.653772352	0.609486641	0.631051652	0.608729715	0.640743722
0.195431426	0.203418216	0.201911450	0.205024318	0.193923731	0.156871446	0.280626186
0.070164335	0.085166514	0.065774087	0.064924409	0.073251367	0.136604299	0.067108499
0.376692363	0.335100977	0.318571423	0.315960197	0.465170812	0.332520578	0.358182858
0.258112707	0.447557200	0.304870298	0.262372107	0.285783156	0.516251541	0.291458841
0.726822567	0.744447312	0.742406891	0.756101880	0.744264824	0.771697248	0.702690983
0.034457438	0.053404286	0.042786227	0.031172339	0.042713707	0.087063018	0.055769825
0.050653374	0.048548640	0.047126458	0.049621740	0.041678283	0.071816638	0.069123784
0.387124757	0.369313875	0.433891436	0.402822188	0.377291957	0.414172823	0.446885745
0.801009816	0.731500906	0.799693693	0.794899848	0.804003399	0.860484382	0.789039632

0.032487393	0.022727784	0.042244475	0.032378301	0.034074138	0.039646287	0.041789299
0.024764965	0.025991958	0.026345921	0.040083370	0.032633850	0.042781561	0.031762608
0.028900775	0.023216415	0.024571256	0.025993311	0.028420367	0.038414745	0.046696830
0.034901360	0.031184377	0.028145944	0.037416395	0.032986526	0.041339721	0.035449490
0.060167900	0.031381887	0.045387375	0.030062977	0.033294075	0.056840878	0.047289099
0.050827573	0.037246030	0.051054681	0.045981445	0.053590861	0.079827697	0.061454010
0.041300625	0.053927177	0.040996800	0.037004507	0.046194085	0.107460158	0.049368702
0.035538954	0.028908066	0.035585189	0.030129798	0.039886170	0.103755621	0.031906407
0.017143501	0.015837085	0.018101626	0.012897563	0.018095184	0.032302237	0.020227264
0.323876884	0.407570772	0.466873313	0.480527667	0.462261458	0.235531738	0.470701962
0.046669894	0.057995437	0.054377893	0.068452928	0.074698401	0.067325049	0.087591388
0.061537933	0.054007622	0.062060712	0.068367255	0.069446123	0.088968371	0.111002352
0.034557534	0.047204789	0.036428171	0.037374871	0.042134609	0.058938342	0.045066623
0.508687271	0.567732502	0.612650783	0.610865046	0.602090379	0.511618412	0.562155576
0.935793934	0.943546581	0.956282926	0.940207539	0.885236759	0.939109438	0.904317170
0.653448042	0.736804098	0.707438763	0.801387528	0.728169428	0.716410530	0.779709562
0.678612729	0.691740411	0.700699715	0.691068265	0.669310635	0.693718412	0.686609101
0.707172137	0.655934205	0.704091148	0.712564847	0.748203477	0.686003543	0.634061761
0.867012209	0.904162562	0.861182801	0.925000176	0.899807737	0.914033365	0.868429395
0.812188324	0.778386733	0.844718082	0.868828883	0.869526721	0.687515752	0.686233262
0.016062635	0.012789483	0.018245832	0.013865804	0.016299364	0.021977138	0.022147037
0.958341905	0.950996735	0.952665210	0.947035371	0.925953804	0.921313462	0.929277074
0.890685921	0.918161176	0.914443925	0.897089627	0.935784411	0.935715684	0.907983571
0.046420775	0.041786959	0.047918974	0.040624959	0.062826057	0.072289028	0.049537733
0.045077258	0.022843621	0.030706162	0.028892694	0.048727812	0.030994596	0.036391440
0.456406610	0.420315046	0.451237116	0.438848339	0.502533747	0.595715792	0.489434415
0.025151196	0.031398826	0.021157891	0.030910433	0.036494963	0.029862142	0.048425167
0.068828451	0.068023187	0.071121862	0.060695743	0.070633047	0.087788609	0.112308642
0.065656424	0.064183890	0.054585026	0.072347975	0.062350822	0.071783355	0.102119772
0.027678713	0.026637700	0.029701649	0.029732591	0.027200321	0.036038723	0.028614835
0.043321428	0.051256715	0.053819891	0.047341331	0.051728895	0.049778821	0.078917184
0.023821762	0.027416921	0.024098608	0.023945927	0.027240028	0.035071417	0.029654710
0.617880518	0.665701282	0.650229878	0.639618306	0.621471481	0.640290976	0.658476744
0.832175597	0.859556018	0.842675647	0.801583378	0.847845866	0.854716886	0.850607276
0.064956592	0.073549599	0.076228380	0.051024032	0.071825456	0.163526765	0.060809986
0.882118112	0.876909390	0.924828677	0.903914689	0.872924098	0.790491917	0.809907309
0.153799157	0.173659527	0.155246015	0.151688367	0.189438838	0.116673298	0.191703022
0.777911609	0.715172563	0.758909498	0.731130066	0.741436833	0.531493104	0.754022271
0.036545260	0.032294069	0.028486615	0.034300359	0.031141837	0.039440586	0.066736048
0.034738230	0.034909380	0.047869766	0.030810036	0.047982477	0.049156555	0.103508055
0.306757092	0.353945662	0.355475346	0.350530481	0.331915339	0.347738595	0.375407564
0.658962908	0.522770750	0.658486114	0.574876862	0.601371676	0.504127815	0.518142926
0.712730467	0.774667177	0.701450687	0.765179338	0.788856177	0.823049876	0.696982170
0.020672994	0.027788620	0.025415164	0.028770637	0.024764254	0.047865494	0.039482547

0.043244436	0.070570675	0.046422768	0.048928991	0.051664117	0.101324035	0.054073804
0.417856558	0.455760797	0.444870817	0.427566791	0.436540632	0.452245281	0.395900693
0.043135348	0.030694096	0.024534462	0.032702194	0.035346422	0.033462984	0.037093416
0.022383849	0.020280235	0.019344405	0.019299242	0.026315261	0.025157487	0.024985078
0.513212727	0.527146302	0.493165572	0.484908205	0.520220424	0.532396866	0.502286856
0.614946863	0.653742548	0.707357646	0.658966428	0.649600626	0.677840435	0.631767894
0.033908796	0.031379757	0.028479360	0.029180035	0.030848147	0.038478225	0.044865236
0.635208660	0.733995306	0.697336110	0.732556444	0.721523073	0.712041907	0.657247947
0.390815121	0.427751031	0.465882616	0.521562709	0.442683900	0.400965708	0.464027630
0.608909059	0.629251854	0.611565837	0.586781069	0.602358479	0.649051759	0.616131088
0.069846055	0.061607523	0.062377112	0.057734271	0.067534422	0.073232461	0.083028619
0.571243632	0.564719753	0.561112327	0.636160056	0.575393468	0.497939845	0.631382083
0.677756564	0.637222606	0.675426466	0.720889584	0.747276018	0.785870774	0.668898527
0.701238754	0.761066905	0.720897884	0.674537329	0.747202416	0.809269200	0.700672330
0.130368373	0.114399101	0.116284013	0.107181413	0.124377696	0.137334738	0.170017424
0.143562625	0.142439807	0.158342910	0.172874391	0.142086611	0.062379648	0.150153922
0.040941290	0.045783192	0.046615508	0.042130406	0.037608706	0.037108533	0.062968802
0.229884165	0.246378618	0.272019390	0.252994448	0.265620146	0.273838353	0.324333403
0.302138209	0.323408137	0.273886502	0.347049624	0.302367827	0.300940059	0.384361152
0.895438493	0.926349456	0.906800385	0.910514977	0.913713952	0.917438643	0.890721532
0.165938022	0.130599546	0.147946436	0.141277939	0.161490011	0.193300119	0.188405992
0.038517238	0.055046137	0.054592654	0.040215168	0.047016491	0.072216897	0.074803304
0.102701294	0.104917392	0.101393752	0.103964210	0.096983829	0.085858684	0.141145140
0.023231129	0.019561018	0.026277879	0.027798690	0.025690792	0.033518954	0.027637219
0.594433582	0.476808476	0.595983439	0.566881721	0.535964285	0.774127489	0.576172511
0.740600168	0.766640734	0.807460332	0.712262649	0.735780006	0.800869015	0.754668083
0.842131851	0.842803818	0.837952314	0.868006733	0.833439762	0.841278221	0.842446085
0.036341487	0.037181688	0.033225339	0.029119518	0.042350056	0.044328373	0.045207044
0.041941806	0.045151019	0.036054339	0.061764228	0.082173889	0.038058512	0.043032028
0.037594150	0.034210333	0.044338787	0.038071901	0.029898320	0.046151621	0.034707172
0.498871194	0.469241103	0.471608331	0.471416205	0.528816157	0.616250451	0.487667044
0.038264862	0.044981434	0.043416519	0.044050692	0.051511332	0.144929000	0.039748957
0.033951096	0.033093226	0.034127087	0.035601910	0.045448448	0.043469899	0.049743666
0.059723124	0.089941090	0.070575008	0.069912911	0.070320406	0.129704117	0.049835522
0.053749285	0.051515061	0.056183815	0.039298224	0.041628897	0.068957676	0.046722356
0.067201615	0.074550358	0.072227662	0.082892673	0.084664539	0.076304221	0.106582522
0.037272656	0.026374006	0.023583864	0.033062815	0.027931728	0.036967846	0.031760748
0.058514983	0.069242955	0.083344786	0.078384485	0.079204552	0.090427087	0.125658477
0.595292897	0.590553852	0.634475993	0.629892914	0.609233878	0.665111296	0.637397377
0.244421186	0.271443999	0.260266619	0.215707570	0.290809948	0.241377574	0.232937585
0.058145561	0.078975935	0.058642527	0.064645704	0.096601061	0.058788665	0.074960973
0.038952351	0.042685496	0.041119235	0.038240681	0.040466803	0.067499394	0.041568490
0.590171294	0.601098735	0.596133006	0.623989237	0.598072148	0.593023618	0.615525866
0.038873062	0.036031917	0.031211703	0.033770782	0.062591016	0.051264021	0.043935849

0.027347437	0.020630801	0.023884855	0.031325829	0.029812528	0.018075929	0.063505777
0.570401610	0.552979564	0.647738338	0.588852602	0.580370999	0.668376514	0.594949186
0.845020712	0.890430313	0.887203514	0.909398934	0.886112082	0.905302346	0.868228350
0.023349971	0.022656742	0.030181811	0.022274119	0.024030131	0.052390809	0.036005382
0.342526764	0.322992282	0.355832694	0.373238840	0.311376167	0.325983858	0.353352315
0.341961580	0.350119112	0.336926543	0.314982570	0.399213415	0.429234965	0.367463203
0.182557512	0.159157749	0.163684160	0.176776411	0.191611574	0.136895841	0.202437314
0.027417441	0.033326733	0.039921243	0.030725142	0.023468627	0.050490107	0.042315105
0.056428711	0.075044938	0.057765980	0.056374754	0.051248687	0.072053997	0.087756622
0.489011560	0.520646910	0.503645857	0.440955734	0.487390684	0.466521257	0.524133008
0.264061742	0.388843121	0.317728345	0.265693733	0.435832190	0.367456947	0.278616420
0.179101315	0.206236004	0.176910131	0.232201840	0.322633382	0.118299137	0.259336538
0.838895138	0.884258359	0.888582946	0.879766478	0.914439420	0.912567230	0.883123270
0.031762934	0.020123971	0.025276718	0.036673326	0.034236043	0.033718588	0.029035021
0.384609073	0.376430510	0.403589530	0.425030636	0.425740937	0.381606198	0.442435903
0.854933587	0.800386398	0.881386811	0.297463793	0.297595875	0.332951570	0.877987789
0.041764811	0.043675165	0.050994171	0.045956559	0.037282581	0.064107484	0.084574610
0.034828916	0.023585416	0.033583195	0.030340570	0.031399340	0.051584775	0.049362436
0.322509594	0.341156741	0.314437153	0.336905497	0.383343503	0.248246031	0.372066935
0.047752449	0.041494091	0.047617882	0.052689519	0.042300878	0.055379505	0.049344913
0.023585621	0.036256884	0.026877863	0.033641498	0.026689656	0.040202863	0.053403167
0.017070968	0.016726906	0.014016684	0.017816851	0.018361370	0.022906877	0.027996728
0.604856720	0.546158082	0.596949737	0.561684790	0.637292177	0.553467891	0.625054666
0.054046396	0.048617429	0.048087083	0.038589816	0.046982001	0.072505659	0.065524109
0.644904618	0.563885466	0.676787744	0.802537811	0.704572106	0.763994679	0.669171471
0.048822380	0.087675210	0.075495634	0.167042127	0.129104938	0.080482060	0.224842916
0.037667241	0.052462523	0.032169355	0.052106484	0.035403517	0.045767844	0.052974187
0.465303456	0.387963562	0.476139758	0.475847880	0.522407298	0.432570274	0.483493431

DA5	DA6	DA7	DA8	DA9	DA10	mean.CONT
0.139743185	0.142828257	0.151139428	0.138602387	0.123219249	0.139130076	0.107468765
0.042544300	0.043949607	0.034922162	0.057060386	0.073453261	0.043305074	0.031504051
0.041332591	0.043288578	0.049978425	0.050367011	0.058927241	0.055918651	0.034441660
0.037522355	0.046955349	0.043925904	0.057853974	0.068118692	0.040971335	0.033618734
0.153230326	0.201792081	0.139611788	0.140038189	0.165942995	0.170307756	0.108832253
0.029358089	0.044343168	0.024201196	0.033687543	0.037484775	0.029049423	0.022706988
0.031736411	0.029413173	0.025665129	0.028843403	0.033536236	0.032770968	0.023438820
0.064889221	0.052067385	0.051047165	0.056134833	0.056423928	0.063468601	0.045212359
0.750981133	0.846202686	0.843772556	0.853716547	0.894105031	0.880481248	0.770797240
0.028056172	0.030076260	0.024069106	0.028437759	0.033329163	0.029079862	0.021801935
0.035813452	0.029674806	0.028572159	0.042322288	0.026463061	0.039100798	0.023556869
0.021571639	0.022325745	0.024709981	0.029978470	0.031035333	0.024030509	0.018844179
0.041637263	0.040635059	0.045958869	0.038642273	0.060002314	0.059762139	0.032661068
0.855480675	0.867969460	0.842113922	0.878089043	0.891291274	0.870957893	0.814731153
0.040513781	0.065100815	0.073397447	0.047584566	0.055702010	0.064756641	0.038969448
0.019992892	0.023126254	0.030059221	0.024394838	0.027792305	0.024825661	0.019584321
0.245577647	0.289354657	0.240710869	0.293910983	0.311231742	0.249607515	0.199680493
0.026271408	0.043483223	0.027347383	0.029205863	0.041490305	0.030447151	0.022679010
0.149069374	0.174904653	0.114932390	0.135339692	0.170883665	0.175216427	0.118277475
0.104577866	0.197144018	0.204228388	0.228168630	0.205287739	0.191864682	0.109211238
0.038569830	0.051242048	0.058537717	0.062330925	0.050152144	0.056460784	0.037416291
0.559803375	0.633016690	0.676982825	0.655452732	0.649836418	0.790885264	0.559677178
0.822821009	0.854857972	0.820095839	0.866099962	0.842893412	0.825889454	0.781962985
0.040260993	0.051146355	0.033712933	0.043735970	0.046364062	0.052386223	0.032888362
0.039604442	0.057996589	0.046698822	0.046826345	0.063651220	0.048592188	0.034419358
0.046647977	0.049859431	0.054754054	0.051867998	0.042749393	0.057919668	0.031907170
0.040871678	0.043845906	0.042564111	0.049197263	0.048180631	0.041927567	0.034044981
0.607484229	0.629728957	0.597172897	0.628650688	0.608406591	0.604125567	0.549841247
0.609958881	0.624137165	0.653688842	0.637945174	0.682636753	0.726955172	0.537226754
0.032901839	0.038039748	0.029945914	0.035175418	0.052921326	0.034958356	0.027661522
0.030700681	0.028169815	0.030745969	0.034202292	0.039892061	0.038493871	0.026211470
0.846946796	0.816550071	0.866454751	0.840840784	0.858095527	0.851492503	0.901253501
0.043523447	0.055209809	0.056047632	0.060630344	0.061040133	0.059045744	0.036666169
0.509265724	0.500901563	0.535159320	0.508468717	0.572439415	0.521852449	0.445000442
0.029903940	0.042979389	0.028049925	0.029119614	0.029361050	0.031232465	0.023004325
0.260283700	0.249532251	0.240396908	0.283717969	0.273987415	0.276084665	0.208099185
0.043322067	0.039690825	0.039186053	0.052233074	0.045638690	0.047174170	0.032474929
0.403396368	0.482105272	0.400079431	0.481337221	0.541284309	0.461340396	0.360233722
0.021138323	0.023820412	0.021894738	0.019533152	0.027224607	0.026216909	0.017771668
0.046705724	0.046163346	0.038995327	0.042045427	0.054762688	0.040787098	0.034052734
0.724467912	0.819925075	0.712367949	0.783349544	0.796777746	0.763451157	0.678775674
0.448843256	0.502600761	0.463417342	0.478879530	0.554577224	0.526376614	0.420298524
0.085007058	0.130431386	0.129281856	0.121422455	0.143300277	0.101186212	0.070544953

0.054940925	0.038880840	0.055804899	0.090208330	0.046917252	0.081105467	0.094823786
0.018613472	0.022554924	0.022370773	0.018664549	0.024495120	0.019237799	0.016967779
0.055738805	0.070722682	0.065732918	0.060490776	0.075551589	0.069939333	0.050173060
0.280459597	0.281347252	0.259872917	0.292167095	0.300694653	0.286334890	0.241137514
0.054734967	0.061939613	0.058903578	0.068516039	0.068484746	0.065813494	0.048037815
0.250362896	0.282678988	0.383995797	0.293131466	0.235780436	0.191998933	0.184189104
0.084750443	0.105835686	0.096954838	0.137527237	0.113295642	0.133280190	0.083484237
0.056507763	0.055634937	0.047010479	0.082116026	0.056348044	0.079460916	0.046811859
0.078690785	0.092578809	0.074498566	0.096309655	0.087373316	0.088344226	0.065209630
0.122364876	0.123190690	0.108923186	0.154090599	0.219500172	0.158589085	0.098851987
0.057135908	0.073876795	0.054565166	0.066027604	0.067059174	0.051479402	0.040488337
0.040898645	0.034262934	0.033921400	0.040106901	0.032865988	0.032198226	0.026401434
0.041580329	0.098876106	0.063857706	0.075600772	0.089198482	0.065062989	0.043915145
0.032350257	0.030820839	0.043062510	0.038226349	0.037107818	0.038419405	0.027440460
0.339262512	0.434299859	0.386722340	0.393577912	0.485594689	0.393623191	0.339191190
0.026756758	0.035735868	0.026764654	0.029809588	0.033850721	0.035457913	0.024045390
0.070886409	0.071281054	0.067395017	0.066053212	0.078283703	0.066851392	0.054775025
0.029534419	0.059399433	0.038173109	0.039866352	0.062314118	0.037802791	0.029000311
0.035749430	0.041904539	0.035463911	0.046377260	0.050442298	0.042482636	0.033847177
0.048961145	0.041749253	0.063591303	0.055293059	0.051136536	0.073105514	0.039816841
0.070128204	0.051888654	0.031351975	0.047098886	0.044342148	0.054092521	0.030539331
0.018095643	0.019087041	0.018075022	0.022459546	0.022103340	0.017487400	0.015879812
0.032698634	0.048796738	0.036665545	0.055339958	0.046071964	0.035571199	0.032492364
0.049013294	0.066210726	0.041939672	0.059583144	0.058722973	0.054657941	0.037866894
0.031675014	0.049141650	0.036316089	0.040072367	0.047672823	0.041522225	0.030818870
0.033763903	0.044985665	0.035397733	0.048232054	0.051405185	0.053355773	0.029040042
0.048421568	0.076085717	0.043343938	0.049318665	0.060987543	0.046592921	0.035590165
0.038162126	0.031151323	0.041063783	0.042029683	0.049424429	0.041230517	0.031998804
0.040924323	0.042125949	0.048431199	0.049939522	0.047969399	0.045713828	0.036610341
0.459199117	0.655835965	0.627399901	0.573628563	0.497734831	0.594973109	0.476111094
0.337200977	0.380044503	0.292227331	0.340222903	0.397314671	0.336112365	0.269356414
0.779277232	0.844945079	0.776289706	0.797125513	0.825678278	0.794043943	0.747968518
0.058743642	0.043326772	0.033599421	0.048070783	0.045303180	0.045561592	0.033991803
0.641740866	0.760093447	0.645170811	0.674800200	0.741894301	0.674848833	0.583102101
0.202172935	0.204966844	0.180805706	0.259416228	0.256974232	0.216513636	0.175303699
0.017175144	0.021529418	0.019273799	0.024569600	0.025340662	0.018749547	0.015618652
0.198333394	0.285936597	0.190577477	0.266888764	0.335231852	0.297171362	0.179722313
0.042981684	0.062366989	0.072953257	0.076407535	0.075538447	0.066374404	0.051380163
0.058626052	0.045224417	0.052012000	0.061524299	0.057394293	0.034959248	0.033929970
0.285978286	0.302575474	0.306075806	0.282766109	0.414830497	0.385608084	0.261497666
0.338693723	0.500174786	0.447948456	0.463053884	0.485763410	0.478776442	0.369947527
0.046129122	0.045601885	0.052939536	0.053986536	0.049805918	0.054084811	0.041664299
0.132937352	0.152140364	0.114059798	0.145296794	0.154669188	0.137605143	0.114410764
0.021170222	0.022716717	0.021702107	0.025857971	0.028265425	0.020851132	0.017944900

0.060294891	0.062449460	0.046733083	0.053220803	0.089894904	0.054720048	0.036969959
0.041982125	0.034428536	0.034014542	0.037055380	0.031633452	0.040036600	0.025341174
0.017499454	0.020045568	0.019598953	0.017221274	0.026314032	0.020014713	0.016269643
0.475760148	0.529055178	0.463779891	0.541334951	0.571409787	0.498135974	0.447220721
0.062329929	0.063751742	0.045688443	0.049351463	0.083412739	0.068224228	0.045213084
0.025107509	0.029224466	0.026797189	0.023053710	0.028493611	0.034822329	0.022174934
0.038825239	0.054349471	0.031618513	0.051743258	0.061128727	0.052736120	0.029430513
0.081126061	0.068438414	0.078897693	0.100605571	0.073228888	0.092281657	0.068298373
0.872458866	0.829864494	0.831376062	0.867941419	0.864707728	0.865426491	0.806779850
0.077314464	0.079332652	0.066614680	0.095862731	0.093013411	0.084881593	0.065549910
0.192300425	0.201537740	0.162859241	0.183002928	0.194628683	0.184706885	0.152845034
0.058688866	0.044845569	0.031310531	0.043795465	0.055413464	0.050645364	0.035615566
0.025890115	0.034589395	0.025558962	0.028959976	0.036874274	0.029982118	0.023075339
0.036470021	0.039902085	0.038766646	0.040547027	0.037812395	0.035368004	0.027635353
0.105327563	0.096058308	0.095513438	0.113231905	0.096311538	0.119476556	0.082762172
0.035457072	0.028364527	0.028225792	0.028518037	0.029679631	0.036576136	0.023700191
0.458437203	0.460407094	0.377766015	0.549698853	0.585226415	0.566010502	0.391349315
0.020129621	0.019381509	0.017306000	0.019389456	0.016036788	0.020980203	0.015276061
0.030484538	0.038767515	0.028377452	0.041354373	0.053108234	0.035997879	0.027029115
0.063645547	0.063447623	0.054458471	0.067224447	0.088000877	0.068865972	0.052483260
0.066857483	0.075882432	0.059599469	0.065301972	0.086708049	0.055260424	0.045703739
0.040323931	0.065139584	0.035973456	0.030238622	0.035427938	0.024803069	0.023029457
0.037257486	0.034600676	0.025577961	0.037811415	0.028485185	0.035236893	0.025933916
0.044282837	0.050247818	0.047360534	0.047944469	0.055450043	0.053734369	0.040411362
0.023741627	0.037470601	0.025505244	0.030976745	0.050909533	0.028794662	0.021931068
0.125942860	0.160177331	0.100182940	0.132005935	0.143430452	0.154014289	0.091576097
0.086735111	0.082636988	0.057822870	0.083074561	0.069383464	0.069631727	0.053498265
0.482683010	0.421639970	0.484877320	0.565553184	0.594208660	0.531574160	0.441521624
0.092095404	0.109371690	0.106313487	0.122190508	0.118694178	0.135726686	0.091165610
0.029801703	0.025333436	0.031937586	0.039683778	0.038992849	0.033946812	0.025124920
0.078094604	0.077918462	0.071293717	0.114192297	0.098281478	0.071521112	0.052819469
0.017001499	0.021998348	0.019148939	0.026213369	0.029513893	0.032336084	0.018664142
0.412597001	0.439084608	0.438787267	0.465041416	0.491425282	0.466682707	0.394910725
0.024578156	0.022878643	0.029698641	0.026708351	0.027055729	0.027190786	0.022037853
0.226889464	0.209747692	0.207260357	0.219658652	0.258709372	0.249366536	0.189035761
0.026404120	0.034527689	0.031060093	0.031513166	0.034318554	0.031169471	0.027205600
0.062505653	0.076302026	0.069472532	0.076551595	0.072195873	0.067907593	0.055616714
0.030877875	0.024704612	0.040922600	0.033803417	0.039296599	0.041064926	0.027231238
0.097577860	0.199050653	0.146264795	0.189633430	0.215570944	0.153236193	0.101812948
0.068334208	0.078946381	0.067127634	0.070509506	0.086473605	0.073282795	0.057326009
0.024539967	0.025799945	0.024209822	0.022372053	0.022858084	0.019862412	0.019262359
0.053729054	0.053335161	0.063751829	0.054009930	0.057483145	0.055752823	0.043265554
0.042880005	0.049987238	0.044940619	0.051926844	0.046804571	0.045106363	0.035812191
0.630477449	0.693546437	0.648216374	0.661264762	0.698758849	0.684020549	0.608000265

0.096356844	0.107454236	0.117861154	0.114259577	0.133789336	0.120370249	0.094173406
0.053609862	0.064841252	0.082774004	0.059183619	0.074302395	0.051752151	0.046352480
0.027284574	0.034345567	0.024487841	0.030962337	0.036146233	0.033568132	0.023003971
0.029258349	0.056323354	0.038233877	0.060342896	0.056674698	0.038838375	0.030156407
0.042214087	0.092737746	0.051502569	0.057110358	0.073305794	0.057631633	0.043264850
0.057467119	0.071533308	0.061833965	0.066789066	0.079327668	0.064025379	0.050035339
0.039299634	0.045406850	0.031393912	0.043315975	0.053496763	0.051688206	0.030272144
0.066638249	0.105133733	0.083256853	0.084905409	0.106737224	0.072312132	0.057142529
0.036285052	0.031234839	0.034774315	0.040188295	0.037266300	0.045097857	0.028577298
0.043286088	0.049582572	0.051456604	0.059784880	0.057361925	0.070714579	0.043009610
0.056517627	0.035099924	0.038926924	0.047883444	0.057702305	0.059373405	0.035596260
0.038842158	0.063584506	0.053893073	0.054116306	0.071861519	0.061477349	0.035991800
0.036666362	0.053038490	0.042265976	0.039228738	0.075327886	0.055099630	0.033324892
0.028224193	0.037964802	0.033827986	0.034631033	0.039560945	0.033375422	0.028177204
0.049339174	0.044432110	0.033058126	0.048223196	0.054389950	0.083692376	0.036891102
0.024867868	0.029600552	0.021708646	0.024521170	0.030446207	0.021086709	0.019933495
0.038154621	0.034248976	0.032522636	0.035312370	0.041297962	0.034845852	0.028100633
0.050651626	0.061752687	0.049784359	0.065806824	0.050956941	0.054960129	0.042887854
0.043472535	0.039851512	0.066166064	0.048316899	0.056347905	0.035380301	0.033569490
0.029260826	0.025918291	0.026914303	0.028325017	0.049470007	0.029866270	0.022869596
0.088960313	0.133195464	0.130135972	0.106150239	0.150984046	0.158308441	0.096665593
0.637813430	0.648077547	0.675221788	0.709244002	0.687178959	0.621683661	0.582786899
0.097072598	0.088116629	0.066632674	0.074357061	0.075176835	0.090611556	0.056572253
0.219172345	0.276064335	0.212021035	0.266220144	0.290506614	0.237172732	0.195740529
0.058923915	0.108352528	0.087906118	0.102956476	0.132019239	0.075031709	0.063994600
0.038238792	0.029197702	0.033857919	0.031128171	0.036178120	0.035507001	0.026734909
0.032703893	0.049639672	0.035569575	0.036259390	0.046549462	0.038365437	0.031222976
0.239151557	0.287510206	0.250308997	0.221557503	0.244803749	0.255373216	0.207884988
0.039137782	0.049423575	0.047806157	0.046539721	0.057643083	0.049275101	0.038490381
0.066866638	0.060182813	0.042919535	0.038368190	0.054144238	0.045216251	0.037207459
0.762084536	0.787752237	0.742673196	0.760636764	0.761880725	0.768101167	0.707002782
0.040591428	0.040965069	0.028268544	0.040039535	0.037919612	0.035308246	0.028580056
0.747608972	0.758402641	0.699094280	0.723407033	0.796733736	0.728542673	0.667399971
0.037377609	0.047822855	0.051875768	0.064154719	0.046265326	0.042770874	0.035616160
0.055253318	0.083681627	0.059138203	0.067913137	0.073910564	0.079136490	0.049839422
0.042811992	0.061679242	0.051180690	0.052617324	0.048316737	0.061274049	0.039828837
0.183815284	0.167603564	0.127568316	0.150870318	0.161949101	0.202462737	0.130816528
0.775027930	0.815499327	0.880019248	0.859886892	0.853863418	0.852669056	0.787962794
0.021606468	0.025267789	0.023140946	0.025936130	0.031813235	0.031319432	0.020868052
0.019807169	0.038424629	0.028200519	0.031132905	0.030644675	0.027342971	0.021585462
0.615204029	0.688160450	0.706525628	0.706317185	0.722453432	0.661493249	0.594443296
0.055616799	0.062581571	0.049806464	0.052361879	0.050054054	0.049423758	0.041833374
0.344052365	0.363141458	0.356196596	0.430073324	0.468465174	0.396995975	0.339404230
0.033018485	0.049153074	0.039506460	0.040020148	0.061253113	0.038418639	0.032820468

0.030187891	0.053731961	0.038220373	0.047983969	0.057099389	0.043335489	0.031933440
0.385782531	0.494299695	0.435254518	0.520833725	0.529866520	0.522861534	0.399902917
0.071278054	0.038966664	0.035878909	0.050263797	0.058206852	0.058278710	0.038358965
0.075086286	0.128818928	0.110972460	0.108103723	0.128901675	0.128487601	0.079969662
0.026756242	0.024273845	0.024772721	0.023625825	0.027188904	0.030278214	0.020268320
0.047005438	0.074226193	0.065992720	0.077757180	0.087768924	0.061519081	0.047753371
0.037716860	0.028626077	0.040122063	0.040077803	0.048890879	0.040183235	0.028360401
0.025589541	0.040709580	0.028760520	0.034037556	0.038440341	0.036545918	0.024082185
0.032954514	0.054269046	0.043193970	0.035312061	0.059674895	0.041447177	0.030910510
0.032295958	0.029116748	0.022637720	0.033027496	0.041598337	0.023994803	0.022852226
0.043090578	0.032772053	0.046564514	0.049471954	0.049170305	0.067710831	0.035361108
0.875084819	0.888721509	0.893289623	0.902122264	0.904440386	0.901679732	0.863399485
0.026327844	0.082777557	0.045987829	0.044253457	0.078583284	0.047988873	0.033546799
0.048093464	0.048143688	0.033354155	0.038215621	0.041042910	0.048798942	0.035945956
0.029472078	0.025609062	0.031247955	0.035703399	0.037334929	0.032254285	0.025743346
0.207973956	0.261382184	0.215359295	0.240336562	0.274556613	0.255845790	0.201746200
0.044197393	0.052180017	0.043905444	0.048564947	0.031499006	0.060489224	0.035367507
0.067700830	0.067333020	0.053416003	0.063132632	0.072807625	0.084604111	0.050143484
0.035053079	0.037302306	0.031855892	0.038145806	0.038887924	0.039432152	0.027703162
0.045327165	0.042153096	0.044926434	0.036015100	0.053599876	0.048425603	0.034281172
0.076857029	0.071750181	0.083989772	0.076021180	0.081175090	0.113152061	0.064540934
0.068252708	0.065420561	0.065524871	0.071140167	0.072425468	0.088822305	0.057380027
0.383510593	0.466181940	0.348693708	0.408904587	0.429174470	0.428580199	0.339054774
0.073177911	0.076081212	0.073083577	0.083542198	0.077152213	0.086512950	0.063557285
0.238036580	0.178161259	0.230530629	0.229792780	0.263518463	0.249171181	0.182081825
0.026230040	0.038457106	0.030000682	0.030735736	0.043126982	0.039473288	0.026317582
0.037741052	0.022413197	0.031557746	0.040038868	0.028863331	0.036564268	0.026362050
0.105639295	0.128413742	0.101713412	0.102891121	0.111330708	0.145210024	0.085176412
0.054502571	0.036467904	0.039827599	0.045257684	0.039530171	0.031885723	0.028130350
0.033419871	0.041617797	0.037558518	0.030427623	0.031677304	0.037049330	0.029164548
0.021841834	0.013299352	0.015213212	0.019708991	0.018395928	0.020492870	0.014713876
0.040999683	0.037607609	0.033971892	0.045645017	0.035013629	0.040910835	0.032769712
0.034870068	0.037956244	0.034019249	0.059244709	0.044691462	0.038544503	0.029264882
0.031902925	0.024355939	0.024143750	0.038618213	0.025387752	0.030587065	0.022561602
0.424144398	0.525119206	0.519222254	0.521080305	0.600198368	0.550008898	0.451187472
0.032921068	0.041089627	0.038951478	0.036167233	0.053115556	0.037787913	0.030683852
0.032808713	0.050128920	0.029763045	0.040523431	0.038026330	0.033821565	0.027953829
0.023705208	0.042722102	0.032078734	0.040680265	0.039328369	0.026887965	0.025710004
0.436899722	0.551545056	0.389374778	0.387488414	0.531584530	0.396579038	0.333657394
0.031852540	0.037029614	0.032400940	0.027478851	0.038568799	0.094050444	0.024264480
0.025196872	0.026602777	0.019945674	0.031866246	0.032979304	0.023320366	0.021687535
0.135331451	0.180561347	0.140713573	0.189356816	0.222120293	0.207900971	0.125548064
0.038724609	0.054727309	0.038209742	0.041503488	0.046987025	0.061175648	0.036881070
0.049811294	0.116074190	0.070749351	0.082057346	0.100853153	0.066582201	0.054031172

0.026920096	0.029862464	0.020413897	0.021923276	0.020285596	0.021740425	0.018912893
0.022090157	0.021501998	0.032462932	0.032303137	0.033784297	0.024166483	0.019662919
0.164847223	0.171026955	0.118755620	0.136439971	0.136842770	0.155070471	0.112938934
0.030834559	0.037153362	0.038813961	0.035732579	0.040227635	0.041533754	0.030817443
0.291904290	0.381018012	0.361744856	0.318735154	0.354909909	0.318521776	0.280237268
0.123784908	0.107698315	0.114604574	0.126589816	0.126058229	0.134736041	0.094449052
0.068354699	0.068875855	0.075220930	0.063954883	0.078258850	0.078414140	0.059671070
0.130352973	0.193728474	0.118049224	0.152640406	0.198011800	0.185794585	0.106154183
0.269075519	0.325915791	0.253081186	0.285725714	0.364607944	0.282293569	0.247287473
0.084229608	0.104535196	0.052923579	0.105108031	0.108140061	0.105975020	0.060127796
0.249724711	0.270100849	0.175105830	0.192663005	0.308074416	0.188183548	0.167920582
0.027933715	0.049374627	0.075237423	0.055980894	0.086059912	0.036447556	0.027712761
0.299677111	0.264740641	0.279168956	0.261174025	0.301581322	0.305639652	0.247380082
0.029928444	0.036308279	0.034079890	0.039517098	0.041602700	0.034762162	0.029274879
0.076513916	0.066117485	0.066513920	0.071657172	0.072762945	0.064399003	0.056366056
0.625670061	0.642021849	0.613835379	0.738447095	0.677373233	0.691553446	0.601977822
0.020313244	0.020079940	0.017580337	0.028245657	0.023577642	0.023462786	0.018234678
0.034517882	0.051748023	0.040491883	0.037039657	0.051989234	0.052775410	0.033825063
0.019013871	0.021990307	0.017457640	0.019886793	0.022540231	0.020956436	0.017320894
0.860695472	0.818712489	0.781078965	0.801202874	0.896168091	0.781565124	0.722187979
0.072515338	0.073269716	0.066859472	0.067909386	0.097648440	0.079622570	0.065717822
0.037740115	0.055985002	0.032798163	0.045087466	0.047309783	0.038942609	0.035011246
0.055756986	0.050600610	0.047300923	0.065655610	0.042194807	0.059050685	0.039376874
0.064613498	0.064166826	0.057014167	0.072480988	0.067935386	0.069375316	0.056215857
0.360340174	0.509026049	0.351824487	0.431388769	0.418083628	0.386092438	0.334179734
0.044519537	0.041932902	0.049632392	0.050383772	0.050275355	0.056064414	0.039904962
0.047012199	0.053262362	0.048435418	0.056551645	0.046226712	0.043254122	0.040670867
0.749323029	0.816439851	0.794936812	0.804110967	0.812852835	0.804624936	0.662525651
0.839569699	0.841769381	0.909486727	0.877429765	0.887675914	0.885734555	0.818004862
0.026264611	0.036688032	0.030433744	0.031542069	0.035196892	0.030581550	0.026659272
0.055508777	0.042455085	0.049096363	0.040158636	0.046343632	0.029292696	0.071474095
0.148869307	0.147477530	0.132337466	0.190627442	0.200839242	0.247577370	0.128552829
0.407564609	0.429914935	0.399001320	0.464964987	0.471141049	0.474866595	0.386799876
0.059209519	0.066571962	0.050477742	0.067663252	0.079070226	0.072245246	0.047543612
0.764330438	0.800334255	0.830765667	0.822015823	0.810220578	0.826180795	0.710579785
0.036705467	0.065308752	0.038482538	0.043501071	0.054297897	0.033374226	0.029518355
0.075976505	0.068837178	0.069522272	0.081972090	0.065260031	0.066352543	0.059277959
0.078000037	0.079244773	0.075868748	0.068786185	0.075875139	0.067154161	0.060239950
0.716989575	0.725499717	0.698568923	0.712892586	0.768021326	0.773598321	0.640657314
0.066426076	0.144277627	0.074805064	0.081403444	0.073336113	0.155930321	0.064545022
0.054575212	0.055980962	0.039226228	0.045488930	0.073783845	0.058959946	0.039682016
0.063978166	0.065250143	0.045321856	0.071484792	0.087629806	0.068177625	0.052634340
0.029844071	0.031777269	0.029243003	0.027041760	0.037177999	0.028909961	0.024430602
0.760706897	0.727852735	0.674628894	0.728409419	0.750751973	0.784772045	0.695712404

0.130899781	0.092190163	0.123679384	0.116218559	0.205397498	0.099087456	0.190159105
0.091166912	0.104186712	0.061623007	0.070438936	0.085995795	0.154842081	0.060477650
0.177043895	0.175742038	0.121882188	0.144866747	0.173024440	0.157846472	0.126957335
0.065673469	0.060823217	0.043092084	0.054508573	0.059736480	0.046676036	0.045487151
0.410873178	0.501830705	0.384857785	0.580857279	0.340273220	0.361771366	0.342674924
0.565300996	0.666441711	0.640828127	0.615679075	0.697897971	0.657320123	0.578597573
0.057099568	0.089442154	0.068398711	0.074410856	0.112629165	0.078071061	0.052279058
0.753224464	0.876879458	0.839004978	0.827272172	0.854615308	0.784111505	0.764698443
0.035118253	0.066129671	0.043038557	0.041791612	0.063293994	0.051600763	0.036470867
0.138223883	0.146244430	0.152642359	0.161920580	0.157802448	0.162668083	0.126101930
0.035920985	0.048731941	0.042466492	0.048428612	0.049296087	0.045922581	0.030780716
0.565034505	0.616069990	0.666153357	0.645159487	0.756179856	0.726133992	0.543669841
0.040909028	0.056348107	0.051952188	0.044474179	0.070066738	0.051765505	0.036921989
0.032713651	0.020807775	0.027514376	0.037315651	0.043265627	0.036805294	0.024894427
0.023654781	0.019798111	0.016740295	0.023578958	0.026895706	0.022734089	0.017729783
0.764498188	0.865180409	0.792052761	0.860663923	0.837459508	0.818524647	0.742834737
0.012651809	0.016334522	0.013865654	0.014020821	0.015584993	0.014979671	0.011975771
0.338100575	0.354748906	0.280991538	0.324525878	0.353483081	0.348562177	0.279169320
0.762835980	0.775031124	0.755583564	0.773772811	0.790598361	0.802392162	0.730370889
0.045132269	0.068832781	0.050878352	0.046420866	0.062697331	0.068355021	0.038999808
0.221960527	0.269293251	0.274794875	0.368356059	0.352205986	0.290245971	0.214316346
0.048417909	0.039027691	0.045834911	0.066654684	0.053258309	0.037750214	0.031384124
0.022800391	0.019539822	0.017152317	0.019112894	0.019417545	0.026611995	0.015752910
0.084085199	0.078879859	0.071300519	0.084402862	0.095992324	0.097766026	0.062203592
0.050754459	0.066738338	0.043516750	0.073012472	0.063329137	0.061806870	0.047264740
0.090882992	0.128506700	0.185628735	0.078490046	0.148167072	0.162549450	0.073042812
0.810846360	0.820848166	0.370596606	0.841226019	0.471913862	0.452905404	0.817922361
0.027654537	0.026473421	0.019507223	0.019682273	0.029210287	0.030775086	0.018955484
0.300251141	0.175779146	0.154428310	0.199226083	0.190733226	0.250940519	0.305291208
0.066865040	0.083222737	0.065058504	0.068836073	0.126802867	0.077718656	0.056273360
0.081290146	0.079962078	0.084817904	0.097213760	0.102669203	0.105424986	0.078034398
0.048788569	0.056692400	0.061563091	0.069219296	0.111162065	0.092532561	0.051385701
0.032590595	0.032833820	0.036336566	0.034974398	0.047999510	0.030563678	0.027307244
0.047120052	0.064399523	0.045968319	0.056074149	0.062677410	0.062950720	0.045123885
0.050944709	0.051065182	0.057699815	0.050648599	0.055073411	0.066940705	0.045165787
0.831681903	0.785762836	0.730659517	0.771342963	0.783836472	0.831958893	0.708173274
0.038342542	0.044380801	0.038098062	0.056941713	0.059435362	0.047974199	0.036125792
0.182397939	0.222926854	0.179837179	0.192349839	0.218188220	0.256634100	0.150123245
0.038598896	0.094179728	0.071143055	0.063707984	0.074899903	0.076761852	0.047726276
0.047246211	0.048379686	0.056786919	0.064375543	0.066604200	0.072683090	0.047214841
0.019444039	0.029945030	0.058916133	0.030392256	0.030758547	0.042630165	0.022563846
0.330923020	0.388563742	0.335113885	0.399893518	0.386930729	0.375892474	0.317282144
0.029242400	0.040843807	0.037329064	0.035046858	0.034067059	0.036428723	0.027556437
0.041930226	0.039495113	0.025777268	0.030759647	0.063796500	0.049943831	0.023605566

0.023696146	0.033849693	0.031150663	0.034556219	0.042935311	0.030209594	0.023990276
0.141478151	0.118714404	0.129401769	0.138236665	0.170344181	0.119392130	0.116731030
0.500336854	0.609547785	0.564116384	0.598580728	0.627314990	0.586419355	0.500848420
0.020026332	0.020771706	0.019319499	0.015437702	0.022091910	0.025327059	0.016252834
0.476868913	0.533086164	0.459540218	0.561119618	0.610569281	0.533082796	0.450401671
0.032141294	0.044388894	0.034621357	0.049100857	0.045255095	0.035770923	0.031721807
0.053369972	0.062868814	0.070840571	0.068597353	0.112529730	0.065903658	0.056997137
0.042016421	0.088422680	0.046855656	0.052792562	0.104003470	0.059445203	0.040675408
0.405276021	0.532896771	0.418560841	0.457975043	0.464933364	0.465615947	0.391630698
0.043405565	0.058143939	0.055938786	0.054172143	0.054349664	0.059852206	0.042970561
0.060833894	0.069462047	0.062892951	0.067744990	0.078456483	0.057150474	0.055898817
0.024489088	0.023582602	0.030567953	0.043340078	0.030412411	0.041167775	0.024537753
0.311237293	0.386228436	0.310476980	0.391023417	0.430489181	0.369775211	0.297883359
0.025896324	0.056877010	0.027579459	0.031705360	0.046200063	0.029915239	0.025391340
0.096212299	0.114716776	0.075957744	0.073174259	0.126918361	0.108595003	0.064547050
0.365940435	0.334633267	0.325622042	0.341320298	0.407834084	0.386388789	0.314942344
0.035350489	0.022395508	0.021201924	0.023684376	0.022395269	0.023695669	0.019744829
0.194076836	0.149700850	0.180314500	0.190984829	0.136887485	0.159788896	0.125989502
0.023341916	0.018924732	0.035567483	0.025708804	0.021093533	0.025882161	0.018398503
0.036950612	0.043747383	0.039922344	0.053029121	0.044689050	0.043314973	0.035842204
0.061379463	0.053660943	0.056250303	0.062133818	0.058328781	0.066952886	0.050990703
0.059500315	0.112511090	0.075768096	0.097092082	0.124228760	0.095452089	0.067257333
0.034896521	0.058869539	0.035525701	0.039865862	0.059121886	0.056436590	0.032278761
0.420012245	0.524900843	0.420405312	0.615049729	0.619630766	0.555052142	0.399620278
0.055217379	0.056799938	0.046458959	0.061622863	0.069936052	0.054569257	0.047433940
0.775319889	0.820891613	0.811913752	0.850213667	0.847606237	0.809589504	0.757087536
0.062189336	0.110459045	0.063232383	0.119931427	0.083959941	0.072405364	0.052733421
0.042643956	0.091019361	0.052790695	0.048389363	0.089317772	0.057072975	0.038336983
0.397772844	0.429086943	0.440254190	0.395119768	0.420780323	0.364650638	0.349718216
0.125373013	0.289644736	0.124081507	0.218230916	0.205584199	0.149957232	0.125645851
0.046253702	0.045943789	0.052685783	0.056268265	0.064310241	0.054812930	0.044492838
0.025857102	0.026730329	0.022636427	0.053287442	0.040023857	0.032897520	0.023919265
0.051692361	0.056325649	0.046658535	0.047058527	0.055588629	0.056163438	0.045532810
0.047487899	0.032995889	0.033114765	0.033214586	0.049904429	0.038586763	0.031700905
0.064927386	0.046688305	0.046638416	0.055416310	0.058408250	0.056375401	0.045574743
0.069284379	0.073899249	0.062833098	0.067823309	0.063415963	0.057078081	0.054380882
0.039839916	0.040835977	0.047650995	0.046613729	0.039922985	0.036218646	0.036621346
0.032269521	0.040998442	0.023297841	0.030204894	0.031132162	0.033857334	0.024604199
0.148452840	0.222545528	0.209258848	0.198375474	0.202768682	0.170457390	0.155202839
0.035869317	0.044977132	0.024285726	0.049198878	0.049225400	0.043490422	0.029315360
0.122685992	0.154772378	0.140594429	0.157671450	0.156213456	0.147720641	0.117819194
0.036909638	0.035073987	0.030200356	0.043885383	0.030001962	0.029797882	0.026945644
0.050262670	0.051291867	0.035175910	0.037624186	0.046428074	0.064708348	0.030036156
0.017752445	0.026173590	0.027250371	0.026763629	0.028069152	0.032164952	0.022185790

0.024066075	0.026283324	0.023542846	0.035148157	0.029418340	0.035261252	0.023068238
0.043523216	0.032716088	0.035037585	0.034351312	0.038361684	0.033171325	0.029577445
0.032069904	0.042437842	0.038571054	0.041329162	0.038217280	0.036726074	0.032714133
0.031763643	0.057848535	0.057844430	0.037699913	0.074799836	0.046165343	0.035840301
0.360288159	0.431044992	0.396833731	0.489487371	0.456354918	0.447834285	0.365062231
0.041749333	0.045036352	0.028750200	0.042186768	0.052594688	0.040052808	0.033077128
0.075891602	0.149266435	0.097413278	0.117652609	0.141450456	0.113157181	0.076496031
0.043802950	0.102291049	0.063029040	0.054855984	0.122215391	0.054947408	0.044702196
0.063639393	0.038159271	0.045980412	0.049344262	0.054931596	0.053138584	0.039820664
0.722724215	0.783100496	0.787624728	0.790345148	0.814096770	0.801327621	0.737238331
0.451455491	0.569987003	0.544253713	0.583913943	0.599408835	0.552882268	0.479957475
0.028917446	0.026330354	0.030008350	0.032489851	0.033482886	0.038137935	0.025200451
0.046587730	0.059831109	0.046670015	0.052025101	0.058593919	0.046682993	0.040795005
0.271524214	0.426221807	0.308970633	0.370116747	0.398225014	0.318287493	0.277690293
0.061130570	0.057937252	0.059586024	0.065736107	0.078537838	0.054401060	0.047861581
0.204973236	0.270061747	0.202547072	0.263093053	0.239757973	0.228846897	0.186829670
0.041907576	0.037811658	0.047864631	0.053010452	0.034014468	0.071403716	0.028782953
0.024745278	0.045294325	0.033723440	0.037781648	0.038549571	0.039243888	0.027425501
0.084808234	0.088129226	0.079899374	0.103170852	0.091669350	0.102273306	0.075123742
0.020871258	0.025665185	0.026320620	0.030570719	0.031037429	0.033466566	0.022294408
0.297223250	0.290980001	0.277672558	0.306744161	0.312959609	0.322450001	0.252631391
0.082968148	0.066508594	0.066807087	0.075346971	0.076462198	0.068750604	0.059097849
0.053515798	0.044641234	0.041585276	0.041062910	0.041074620	0.057130839	0.035671832
0.032685021	0.047714535	0.045483190	0.019068841	0.026316083	0.020476227	0.021397867
0.105084881	0.081350038	0.086564864	0.097327060	0.124588178	0.117588847	0.085162850
0.416738523	0.524255609	0.333491240	0.563007725	0.523736672	0.519584692	0.381233713
0.043123576	0.043048313	0.040000008	0.045080103	0.044918258	0.036392648	0.031707034
0.019401952	0.018667998	0.015909354	0.018970130	0.022578087	0.019758046	0.015999960
0.804215059	0.828442806	0.794708655	0.848311494	0.861622384	0.881335987	0.780314342
0.022805623	0.027205580	0.021614659	0.026942470	0.030329331	0.032145646	0.022646297
0.037226609	0.069175021	0.064987464	0.045535794	0.085505769	0.062572567	0.039814699
0.016842483	0.014678307	0.022447155	0.017217587	0.018182269	0.017891216	0.014366851
0.086630956	0.124385788	0.059938963	0.077375305	0.080486220	0.108024428	0.057440032
0.050703340	0.052415203	0.067690500	0.059410288	0.040682038	0.040275321	0.034782463
0.032801237	0.041929218	0.050577309	0.044531071	0.059107083	0.048572695	0.035237638
0.062805535	0.050394176	0.059343028	0.066458051	0.058389193	0.059291376	0.051258035
0.326078469	0.294529118	0.330939360	0.296432862	0.326227247	0.313486993	0.271699639
0.046044740	0.040905624	0.042806097	0.072257184	0.064849340	0.056334109	0.041416278
0.057790979	0.052760690	0.046136778	0.051533520	0.065372783	0.052922875	0.040764530
0.838729097	0.905724352	0.876234328	0.859053727	0.905079670	0.868616906	0.843055466
0.330772180	0.401826504	0.446129764	0.397288984	0.483793116	0.434791351	0.361102256
0.026230134	0.039611947	0.033343157	0.033377072	0.038973587	0.032304207	0.025282583
0.029966755	0.046293295	0.030769183	0.034994752	0.033867861	0.033806819	0.026040600
0.025645749	0.041305125	0.027304499	0.035595734	0.035772171	0.034996797	0.025686756

0.024432656	0.017313251	0.021481275	0.022837918	0.022869865	0.025954311	0.017911302
0.710820446	0.834569116	0.793492510	0.820492148	0.835607693	0.797110343	0.740320669
0.044668555	0.044161777	0.041431688	0.047678330	0.049184122	0.042288439	0.035698484
0.536599741	0.543676054	0.598436850	0.499134778	0.606188596	0.564070042	0.481350785
0.428884744	0.531342820	0.408575353	0.418004991	0.482603097	0.392150794	0.366169145
0.334817626	0.375843057	0.330298396	0.341380901	0.405264851	0.361540978	0.309977046
0.038894486	0.056235251	0.046870643	0.108469715	0.057822593	0.087514290	0.040951789
0.040993099	0.043773617	0.048729037	0.043074659	0.041869743	0.049256655	0.034334762
0.270605609	0.371374285	0.255448122	0.456072706	0.392029644	0.371786741	0.260898470
0.341652568	0.460023824	0.308214208	0.331363901	0.454423551	0.383398463	0.304767089
0.794715384	0.868332983	0.867488260	0.891276177	0.914403600	0.911979747	0.826004576
0.215487257	0.263396018	0.187493261	0.257600068	0.268827956	0.261931173	0.203192513
0.379865309	0.386845753	0.422595472	0.566233579	0.460257491	0.428461592	0.368881740
0.065802936	0.155141257	0.114146653	0.128533106	0.133126058	0.104829616	0.081517762
0.369875377	0.377882360	0.375150086	0.425590649	0.426623626	0.404478309	0.346861910
0.025289180	0.040901755	0.024004619	0.022699438	0.033050728	0.032042828	0.023352168
0.031331801	0.026149164	0.026532203	0.023482623	0.025511897	0.035889020	0.022870526
0.114300266	0.142408452	0.147996345	0.130921060	0.235766968	0.151642009	0.109969145
0.034738949	0.036769020	0.037200544	0.035920111	0.037275291	0.033868072	0.031130622
0.677036791	0.657170398	0.609528216	0.614468820	0.700362569	0.685072118	0.572800844
0.035278006	0.079959024	0.075839584	0.088428821	0.086021840	0.070040082	0.044757182
0.612031046	0.761653353	0.709424619	0.727297247	0.795650223	0.738621904	0.620977913
0.046072817	0.035345825	0.044965986	0.042280627	0.040096546	0.039817498	0.032059494
0.035698108	0.053155083	0.024518862	0.023803158	0.028481390	0.032911172	0.024128122
0.060229129	0.072147207	0.066053141	0.062330997	0.070971194	0.070677277	0.056280595
0.549681684	0.718757589	0.612605892	0.716375880	0.731892731	0.665524633	0.579297381
0.123944396	0.123845903	0.102800580	0.139476805	0.134111470	0.128337303	0.100865335
0.018872913	0.019902608	0.027917399	0.022161023	0.027679623	0.038052393	0.018417267
0.264722620	0.278842717	0.254674191	0.274523825	0.286291594	0.289995994	0.241653671
0.299067887	0.259234692	0.292956488	0.285492153	0.338080179	0.326710992	0.266104003
0.051538837	0.053776274	0.053021130	0.059242433	0.057139596	0.044626124	0.040862688
0.075549511	0.058672439	0.065054358	0.052875770	0.067783220	0.060871161	0.082650137
0.029116791	0.025992919	0.027844705	0.034323018	0.031921692	0.029870761	0.023118447
0.025035338	0.040349949	0.032128000	0.039120873	0.045021840	0.044083647	0.027545083
0.050218394	0.057407037	0.046684926	0.058569004	0.047852632	0.054783511	0.041961767
0.261914280	0.297818170	0.268645263	0.250439764	0.381303001	0.302429484	0.256882369
0.039549561	0.068208217	0.049770787	0.058024433	0.069472625	0.046560370	0.040231418
0.028528135	0.024002220	0.033269606	0.036530851	0.036256708	0.029652545	0.026069137
0.788535030	0.787907020	0.778167979	0.809629464	0.846849029	0.806771821	0.754849061
0.021738605	0.020256409	0.019964297	0.020738482	0.031994684	0.029311408	0.019171801
0.026097067	0.033020415	0.027373168	0.032544059	0.040898128	0.034113407	0.026239834
0.032439051	0.047337500	0.038164296	0.050916773	0.050656616	0.036169883	0.031115936
0.039915216	0.052918665	0.038651288	0.041402607	0.044874226	0.052768028	0.035733209
0.031562426	0.060057383	0.059032075	0.045007758	0.061996570	0.048767701	0.035322354

0.770656848	0.827736757	0.757114476	0.852228515	0.833706612	0.841240322	0.755380935
0.040256254	0.049824377	0.043690538	0.052041019	0.043834467	0.066179684	0.037705468
0.030822692	0.055742151	0.035671867	0.044326539	0.081907991	0.046506312	0.033634826
0.335792505	0.341768976	0.342727107	0.374054664	0.366979733	0.348155618	0.310860705
0.865832029	0.846909725	0.859832211	0.863168487	0.848395320	0.874838330	0.815955408
0.267030871	0.296977507	0.277189486	0.321792589	0.292624410	0.306178077	0.259208399
0.034328562	0.050715499	0.041971363	0.056792275	0.084940634	0.048794343	0.037419444
0.029237313	0.100358369	0.054136262	0.042769756	0.076313159	0.043934315	0.033798825
0.080519766	0.100101074	0.082502889	0.126017082	0.112227701	0.098485768	0.068675229
0.039565033	0.049218510	0.031282489	0.053246108	0.051546200	0.055538562	0.035396505
0.401860674	0.426708904	0.428994764	0.412420555	0.489634537	0.419715877	0.372560040
0.485273662	0.448596786	0.451415305	0.510623155	0.528137994	0.541193609	0.424541730
0.184764014	0.179883885	0.133288701	0.126550677	0.209711714	0.202578151	0.115840972
0.028276640	0.033750034	0.027479722	0.033714141	0.059038246	0.045476858	0.025367830
0.034859246	0.029587848	0.023914789	0.034353884	0.035906148	0.034192294	0.026343590
0.022659716	0.024788956	0.020606847	0.024769693	0.031792108	0.025982934	0.020816262
0.022801761	0.027828029	0.032470559	0.025231907	0.032028462	0.025163453	0.020620330
0.033556615	0.034052415	0.025491568	0.038826226	0.041096217	0.043186659	0.027716139
0.026879820	0.026699734	0.031561212	0.027810694	0.031659977	0.027744362	0.024042922
0.074510727	0.132053457	0.109999163	0.139689375	0.127790462	0.121287160	0.065599804
0.076611245	0.056404882	0.095054491	0.162710431	0.090550931	0.083409443	0.057547188
0.024473883	0.030661023	0.023504146	0.027249785	0.034652086	0.041298189	0.024084199
0.036059507	0.038139561	0.042090711	0.035596019	0.055908251	0.034631050	0.026970015
0.028561324	0.022726110	0.020139849	0.046169078	0.025578684	0.024310790	0.017825343
0.055976900	0.132779302	0.060430364	0.055356207	0.118692802	0.072986980	0.052633399
0.060121142	0.085518295	0.056857466	0.081007762	0.082940379	0.086400598	0.056459086
0.073903720	0.091144131	0.640388342	0.034184886	0.671287398	0.460832482	0.066197145
0.037414326	0.028493044	0.024825276	0.031959329	0.036296240	0.030630182	0.024075488
0.728902514	0.820585679	0.760433315	0.797062068	0.857112706	0.783807217	0.734485006
0.063520508	0.051179484	0.063325854	0.066489265	0.059424959	0.067471456	0.054077792
0.035527973	0.062771269	0.035054876	0.040794915	0.053877726	0.034614207	0.030870162
0.041411798	0.030086111	0.039153933	0.045949743	0.030921552	0.042162564	0.029956164
0.157389396	0.087766769	0.096695500	0.110348938	0.113197028	0.102667776	0.094768515
0.031027653	0.030528175	0.029634975	0.033526191	0.046323789	0.044204304	0.029484239
0.027107987	0.030330678	0.027113916	0.032442495	0.027876057	0.036483299	0.025966371
0.080027144	0.076354806	0.084074084	0.103734858	0.110513773	0.102579035	0.071899236
0.293186131	0.309252076	0.279813764	0.324008102	0.385535483	0.318629652	0.272320963
0.401530727	0.402844845	0.399583638	0.440934841	0.496208387	0.452751013	0.377918564
0.330260478	0.407136184	0.261808037	0.307088692	0.338488172	0.313515455	0.239631410
0.018356654	0.016907151	0.013947129	0.021166340	0.019564855	0.019230965	0.014869474
0.368467069	0.353755447	0.289494345	0.243348658	0.369277843	0.355872905	0.272952001
0.102923398	0.098887269	0.092738219	0.103134425	0.100324706	0.094423388	0.083199398
0.280434279	0.336604741	0.283993250	0.371935586	0.374914894	0.272866822	0.239383074
0.835340426	0.892800941	0.896826453	0.882006877	0.903202424	0.886454528	0.840951433

0.630175481	0.671932906	0.651502478	0.649708619	0.717561800	0.695513552	0.621329088
0.029318257	0.047543863	0.040861689	0.043180097	0.053625842	0.045918657	0.035289245
0.025472189	0.045823657	0.028722490	0.024028823	0.037527145	0.035620848	0.024399400
0.526877154	0.637259026	0.507014977	0.657081391	0.686964936	0.575348408	0.513383897
0.027695910	0.033871413	0.025555582	0.033906229	0.040249867	0.043134911	0.028492196
0.504157560	0.761047812	0.683190220	0.701727396	0.818831774	0.775611775	0.611340259
0.122916794	0.248311049	0.143720971	0.179739400	0.128978054	0.105883006	0.111723511
0.894312960	0.903948437	0.894574591	0.914743254	0.930379214	0.928711235	0.884886049
0.531365978	0.636159630	0.503267092	0.526800547	0.542563031	0.525132526	0.453178504
0.560696049	0.602042781	0.522404118	0.613175264	0.588630379	0.583440644	0.519304878
0.264350699	0.288245425	0.250157539	0.278859111	0.341459699	0.391786382	0.231425464
0.614333732	0.686039020	0.731283427	0.697962150	0.675219493	0.691035485	0.632436760
0.040506324	0.038319282	0.035319491	0.041420303	0.025244717	0.047012763	0.029472070
0.024428850	0.030120132	0.024451753	0.039868147	0.040497078	0.028925986	0.025153008
0.896346792	0.922526053	0.898191242	0.912822937	0.923529288	0.926207073	0.891687131
0.035609175	0.067168412	0.054277925	0.067090824	0.084299830	0.070303233	0.043356365
0.031208619	0.048176775	0.037772996	0.046370199	0.054140667	0.049713234	0.034280351
0.327872774	0.356796439	0.372893691	0.377285002	0.414930749	0.417241338	0.327993576
0.030997844	0.032623047	0.031699516	0.035887533	0.036819812	0.040420747	0.028908848
0.051584008	0.035809202	0.035616483	0.054842724	0.050298411	0.048673338	0.037318861
0.306633109	0.362397875	0.296126182	0.328632310	0.346314598	0.361229512	0.287271505
0.121632243	0.062972018	0.181185677	0.058409885	0.064201398	0.111552417	0.219243044
0.045569205	0.090015529	0.078276384	0.078477289	0.081900367	0.067544819	0.054041902
0.034934544	0.032916994	0.035934166	0.032548396	0.037115956	0.038814098	0.030193543
0.017380087	0.017057103	0.016077016	0.017314787	0.019536804	0.020917330	0.015411685
0.022052730	0.019721963	0.021631709	0.023572535	0.020404073	0.026748244	0.019857777
0.030744661	0.030671573	0.047124536	0.032198940	0.039210021	0.029252037	0.026478105
0.037780605	0.035551074	0.027358437	0.028300675	0.043261553	0.039699209	0.029048530
0.906060329	0.900733007	0.874761860	0.923698208	0.926951495	0.920910268	0.879335660
0.028612863	0.032566965	0.026216146	0.031907122	0.026881330	0.027258544	0.023956625
0.900405350	0.904190325	0.920889523	0.919313625	0.926390700	0.904368418	0.895663545
0.802149163	0.868775637	0.859594816	0.866061858	0.884364677	0.870147348	0.828844680
0.306715771	0.322523026	0.259264988	0.273238274	0.335181332	0.365742395	0.258374377
0.077346537	0.196491885	0.091048107	0.105950753	0.158671779	0.104492351	0.079187629
0.251516769	0.286654500	0.233721347	0.252653409	0.273625260	0.281120890	0.216041517
0.547610010	0.565300885	0.558132976	0.479489620	0.548692773	0.556545351	0.485693762
0.082341081	0.050055293	0.053910735	0.059544336	0.059730146	0.062622610	0.050321063
0.132325964	0.125521175	0.125118039	0.138730920	0.128235105	0.148414174	0.111766825
0.828213201	0.898523276	0.886346928	0.903523164	0.903872778	0.904164877	0.843228766
0.303508668	0.364236320	0.320326010	0.354800919	0.350709414	0.354899210	0.289523110
0.019053987	0.028671574	0.023850950	0.027562423	0.029748016	0.020838047	0.019940837
0.044775276	0.040378322	0.035881809	0.039087764	0.043967249	0.049625669	0.034876133
0.019523779	0.019923556	0.017886335	0.020298050	0.027430753	0.024861294	0.017453385
0.485287682	0.576764363	0.513460068	0.566777457	0.650641001	0.607039677	0.481845293

0.019311798	0.019212444	0.024350138	0.024302605	0.023631985	0.020117006	0.018452013
0.035213119	0.027363019	0.032728343	0.042698250	0.033676738	0.043896430	0.030300954
0.564113398	0.521625918	0.501568586	0.514248152	0.608897193	0.625496477	0.492352118
0.056599262	0.093246878	0.077757272	0.067085014	0.087777698	0.087571676	0.056798780
0.028008596	0.023695378	0.021080507	0.028642348	0.026994367	0.034669280	0.022886298
0.834422396	0.885197513	0.856593152	0.884914387	0.905319214	0.871223185	0.821478591
0.200527800	0.170510519	0.146201504	0.223665808	0.217303936	0.178443266	0.155419837
0.027177409	0.047110208	0.030530792	0.044602714	0.046037269	0.036859278	0.029759387
0.024764161	0.035150599	0.034455409	0.036910904	0.034587749	0.031015354	0.027658811
0.285146504	0.271937525	0.268322659	0.288124969	0.290071767	0.285727913	0.241342158
0.031101876	0.024749890	0.026225735	0.027377525	0.034465715	0.028066772	0.024105815
0.019640145	0.041826715	0.025805494	0.033152835	0.031720887	0.036827480	0.022679653
0.129675069	0.230206088	0.114055765	0.184221436	0.223015423	0.189400963	0.120206145
0.048740886	0.054734750	0.042916251	0.052118143	0.055354099	0.049431473	0.041265306
0.021536416	0.019960747	0.018339189	0.027450292	0.030973508	0.021412243	0.019324641
0.029758218	0.033586194	0.026761772	0.028417034	0.047326345	0.040218892	0.027459697
0.032201517	0.042639226	0.031089692	0.036239415	0.037363006	0.041046319	0.028205300
0.023575006	0.024340491	0.025037051	0.026468440	0.028845717	0.025832465	0.020095351
0.330847498	0.418407086	0.281093492	0.460176063	0.478649251	0.437242003	0.314665324
0.487390550	0.531274626	0.482850316	0.487041886	0.577669310	0.534671133	0.462841492
0.039382152	0.054950323	0.041375642	0.053567611	0.047148773	0.060197646	0.038502103
0.023166774	0.020836650	0.020461584	0.022100896	0.026882949	0.027412431	0.020037789
0.832442036	0.856177829	0.779916571	0.843831134	0.884595188	0.877060253	0.880893039
0.813456529	0.875243458	0.883403408	0.890804650	0.908755495	0.877135525	0.837112118
0.025746752	0.023354873	0.033494354	0.025840273	0.026278300	0.024346538	0.022432368
0.424722817	0.490200277	0.426394013	0.503944957	0.443604890	0.457325671	0.389029575
0.253522942	0.225535206	0.296247865	0.246286807	0.304906171	0.332394903	0.223542099
0.036200097	0.043912996	0.040363743	0.043097245	0.040034334	0.039940130	0.034643600
0.031083244	0.027823730	0.038014210	0.037082762	0.050271352	0.032962238	0.030997672
0.051246813	0.042236913	0.038168788	0.036583312	0.042616243	0.041050450	0.034302492
0.423938799	0.555611158	0.499236120	0.426791425	0.510933271	0.465969229	0.401376435
0.560180446	0.652951124	0.618462535	0.681363588	0.720722926	0.667626655	0.581324545
0.053778030	0.036598650	0.043143166	0.054990542	0.046695773	0.041483214	0.037239657
0.058164367	0.078704844	0.111408715	0.069287430	0.088387797	0.083806303	0.058729654
0.067992432	0.114846719	0.105513384	0.107496537	0.110905133	0.086848338	0.067920922
0.580621762	0.685572488	0.556263175	0.699884812	0.719947930	0.669990612	0.558631260
0.070844682	0.078987103	0.072202598	0.089889194	0.130498288	0.122511591	0.064130005
0.023433308	0.016140238	0.017084966	0.017550494	0.016561722	0.020495565	0.014622038
0.511388569	0.535399245	0.526783474	0.618434879	0.588811362	0.584700993	0.499371180
0.921520438	0.912947195	0.911409789	0.928385364	0.935603357	0.935484892	0.911258330
0.452698074	0.547467050	0.434856830	0.375063354	0.482657170	0.471134443	0.344321104
0.179485012	0.132623559	0.085958062	0.093243631	0.100647002	0.123756814	0.149603536
0.027307927	0.027923836	0.027165785	0.025920104	0.027996985	0.030258065	0.023703264
0.022369896	0.028558941	0.027637153	0.033818658	0.046136064	0.028870838	0.023910576

0.071103970	0.146787807	0.119026176	0.115820204	0.119273847	0.140994844	0.088665262
0.051217826	0.067468478	0.058020844	0.068117201	0.087350036	0.070186492	0.051458589
0.031618580	0.025428048	0.037699040	0.027543124	0.032493906	0.036305885	0.023730164
0.131913088	0.117241133	0.100636843	0.135750876	0.116708604	0.136103647	0.101794902
0.744706110	0.899096368	0.835923391	0.877945480	0.898062742	0.785606029	0.755518428
0.035308407	0.030763003	0.046191231	0.061089124	0.043668074	0.051109462	0.034753629
0.031426927	0.032109748	0.027126134	0.039007619	0.043785906	0.044769807	0.029577677
0.173615924	0.184450940	0.107006421	0.098666381	0.160186395	0.154179370	0.108393659
0.028533009	0.029525663	0.031443958	0.031630397	0.026097465	0.033607274	0.024036297
0.885747576	0.918172662	0.908465255	0.903826206	0.917520806	0.928948621	0.890617489
0.032344177	0.019195008	0.042399963	0.036224730	0.022590909	0.043484625	0.021021336
0.033175036	0.051965511	0.043981277	0.040444084	0.056009443	0.064822298	0.032065137
0.019135038	0.029978276	0.021391879	0.028520650	0.029226249	0.026108953	0.020683999
0.852659136	0.899025328	0.891932967	0.874985645	0.877021378	0.896654407	0.836111804
0.647782775	0.728637865	0.676678728	0.736689216	0.806146839	0.749865116	0.619925937
0.025061678	0.019201711	0.024141591	0.020110018	0.026291176	0.021439074	0.019093399
0.092698074	0.102340919	0.103017930	0.143850852	0.180681687	0.097960115	0.083582564
0.051781505	0.061933604	0.046210629	0.050159402	0.058514069	0.057969007	0.043939588
0.693178963	0.695014943	0.808619544	0.752058366	0.797946940	0.866826043	0.861427571
0.025897882	0.045802550	0.027990775	0.028223111	0.041813471	0.028342550	0.022997996
0.139436068	0.162918195	0.131411772	0.188891631	0.178085407	0.123837172	0.126796598
0.035825755	0.032149893	0.024229605	0.033703031	0.033580880	0.026853104	0.025195711
0.033881308	0.052972353	0.037922764	0.053616216	0.050639326	0.040557103	0.033534696
0.545368293	0.561234558	0.511171770	0.598012918	0.547667764	0.567908780	0.475666553
0.057553122	0.066094900	0.053736457	0.059773942	0.061166031	0.073296483	0.052213666
0.329097654	0.353995346	0.372451774	0.357527266	0.412159133	0.347543610	0.309954178
0.034831190	0.043558043	0.047213643	0.052938714	0.055863870	0.056967051	0.040945962
0.038346856	0.032758791	0.048433127	0.063650547	0.057307613	0.043315163	0.036014327
0.456164129	0.563867355	0.559924559	0.536876166	0.670796144	0.558816843	0.492540644
0.017663567	0.019432934	0.017572723	0.030942443	0.022186350	0.018992543	0.016450996
0.438317145	0.458742839	0.487798864	0.494347971	0.561023366	0.548411198	0.448784383
0.546386702	0.620343233	0.551193366	0.622872269	0.634054605	0.636692061	0.542742881
0.030884306	0.050243667	0.047512249	0.047058856	0.054535655	0.050695847	0.036478128
0.039922977	0.051416062	0.041465279	0.041013000	0.059714084	0.043218893	0.033829559
0.033352045	0.043052663	0.047307574	0.038116540	0.040196011	0.038149853	0.032867342
0.020819407	0.029414727	0.020581034	0.029953350	0.033907424	0.027631551	0.020435524
0.067466261	0.102547659	0.078142759	0.065555015	0.113575275	0.089452375	0.058660479
0.042520242	0.038516206	0.042860464	0.037667911	0.073882944	0.057837823	0.035333413
0.026187267	0.057531495	0.035162370	0.038820949	0.060301176	0.034240304	0.028430929
0.053795345	0.059283949	0.036381367	0.038335566	0.061746947	0.040564681	0.036254336
0.052761576	0.057189462	0.062669612	0.065137118	0.067873667	0.065760422	0.054962337
0.041907902	0.117504408	0.073662209	0.076720271	0.098918213	0.080228976	0.054126262
0.039764449	0.048873115	0.040715903	0.039777343	0.052417505	0.035977607	0.035034312
0.256233488	0.247131315	0.258723580	0.284789520	0.289268155	0.298791972	0.238253813

0.052861535	0.058122966	0.050384783	0.063778492	0.064304517	0.078141547	0.048195604
0.319838239	0.493888965	0.395151044	0.506019467	0.415944281	0.430977289	0.330170461
0.443829389	0.625984151	0.525285217	0.658139784	0.558352935	0.513306138	0.449287793
0.509213879	0.559712364	0.518200312	0.444626108	0.531621579	0.509737628	0.454080358
0.072925945	0.067725817	0.058491815	0.075472775	0.086911466	0.063199230	0.053689743
0.051486863	0.056523774	0.056254339	0.058927103	0.067750614	0.060220921	0.048113201
0.034576868	0.053822728	0.056175598	0.091030464	0.075666759	0.075817968	0.044933996
0.469449026	0.528593386	0.489706529	0.550545997	0.596558351	0.535595231	0.461034910
0.524426793	0.656156222	0.596664747	0.663101275	0.687814348	0.640051383	0.564688278
0.925202686	0.956056076	0.970169577	0.974280666	0.978378664	0.971407586	0.975526546
0.822047402	0.826357430	0.843527218	0.859517149	0.883110589	0.890228171	0.824272686
0.027116599	0.029423958	0.055357214	0.038519726	0.050425503	0.050314439	0.031285430
0.046094956	0.081170469	0.061977947	0.054891219	0.076984921	0.054454121	0.050144365
0.026188573	0.041805828	0.037819938	0.031685093	0.034325528	0.029124672	0.025852596
0.520005828	0.508473456	0.493174743	0.495963642	0.572493144	0.557943603	0.444895797
0.171674761	0.280340934	0.161823393	0.252583599	0.266978765	0.370032462	0.171782441
0.243613058	0.252783645	0.246144486	0.301806294	0.368747524	0.287469841	0.231795187
0.351576844	0.330862254	0.327140121	0.341802957	0.364876398	0.370294086	0.300955562
0.209924984	0.167137846	0.205612845	0.229196764	0.180482846	0.134798282	0.129109726
0.055653095	0.060602585	0.069333266	0.050782114	0.077674482	0.068371457	0.047828785
0.025072187	0.039433587	0.056478427	0.028988427	0.037906223	0.034394951	0.023104809
0.030048609	0.022280084	0.027312605	0.035804665	0.025040029	0.031868387	0.023562918
0.237982757	0.393578761	0.284611671	0.284217893	0.280332795	0.327971149	0.232627971
0.238890917	0.287521945	0.225280115	0.281147303	0.346350264	0.256087931	0.207055727
0.182835804	0.292320617	0.140605782	0.262822480	0.235614616	0.186441270	0.168916370
0.373644590	0.424550055	0.402560815	0.451362856	0.468959383	0.465154612	0.380268770
0.524683828	0.538865300	0.531784206	0.558635768	0.610706338	0.591423507	0.489183501
0.236091403	0.281519511	0.189855430	0.222089862	0.240697385	0.254594298	0.181388360
0.645174314	0.799353489	0.688219526	0.789909688	0.781064077	0.745104144	0.678930198
0.041953717	0.039993054	0.040364390	0.042486804	0.041802750	0.056088944	0.035949033
0.042123457	0.040920872	0.037262419	0.040359143	0.062624456	0.045471683	0.035627172
0.035779411	0.028737256	0.027221076	0.036449491	0.046823947	0.022428887	0.025509278
0.018310856	0.025709210	0.026361130	0.021106454	0.024029420	0.020488363	0.018085416
0.033092592	0.034173364	0.031962452	0.033539480	0.041183820	0.042298637	0.028528353
0.811845664	0.899120432	0.867979782	0.909102817	0.927319547	0.926265376	0.864004288
0.045106610	0.038563135	0.045189602	0.039935164	0.048760155	0.040246876	0.036583758
0.552132422	0.676968364	0.660711783	0.645881470	0.649665247	0.679866906	0.559891011
0.054231786	0.041859203	0.042535399	0.054210974	0.051041508	0.061445933	0.037993410
0.049787874	0.091839422	0.069673237	0.109313685	0.080126679	0.093264157	0.061071641
0.027551519	0.021921239	0.025264294	0.025474723	0.027998930	0.028778442	0.020413678
0.285516153	0.401303711	0.319787575	0.342516978	0.382176314	0.356945357	0.295104462
0.318972532	0.336003051	0.327159365	0.335516647	0.383683380	0.385465366	0.273127010
0.827016643	0.857199253	0.834718232	0.844424977	0.862802091	0.829530352	0.806664012
0.025363813	0.045362502	0.032412005	0.039923886	0.055115560	0.037333231	0.026850068

0.042289792	0.106307113	0.062913926	0.087891540	0.114686577	0.066159503	0.049474379
0.038475762	0.076961097	0.064257468	0.044699690	0.058886566	0.066381332	0.043455867
0.018825189	0.019785312	0.029522932	0.023823700	0.025656409	0.024996573	0.019606422
0.602720724	0.650987952	0.707762404	0.797920769	0.729333439	0.636861039	0.604212803
0.031327750	0.068048278	0.050616879	0.060136309	0.115673513	0.065375323	0.038459650
0.058314051	0.053158709	0.039702673	0.048443735	0.051696515	0.043456004	0.038363228
0.929601720	0.883811004	0.926147925	0.923556679	0.920949240	0.945029578	0.944688082
0.053546493	0.054385955	0.042744322	0.044357059	0.058214647	0.054661889	0.042286591
0.059526518	0.139426170	0.110815685	0.146228671	0.145906861	0.114842881	0.075031800
0.261426724	0.231599029	0.216169996	0.170714763	0.294215843	0.265665420	0.200259274
0.077015372	0.071473838	0.069191625	0.077339042	0.078470955	0.081853465	0.054411058
0.501097788	0.538072842	0.505813398	0.546785336	0.584984394	0.598995852	0.493117009
0.217714948	0.261098698	0.179792551	0.218246310	0.226169442	0.249003486	0.180201681
0.038860961	0.075214166	0.052644887	0.040503761	0.082728048	0.064276922	0.041810324
0.622110247	0.820975182	0.730594060	0.738795409	0.785215646	0.752489654	0.668493793
0.061267243	0.049497690	0.059906157	0.057240131	0.056335987	0.064948086	0.049618338
0.949529339	0.967303507	0.912849426	0.913497333	0.945006433	0.962072323	0.967455724
0.514352262	0.549970440	0.414581520	0.510135872	0.521263513	0.555271337	0.357291879
0.025602862	0.053038083	0.032037075	0.033212307	0.042259984	0.031040356	0.028593950
0.069269257	0.054705238	0.066206531	0.076406468	0.083211243	0.078117442	0.056705761
0.034616862	0.067486656	0.045996880	0.062405114	0.059259324	0.069646740	0.037951495
0.026795468	0.033665884	0.037091069	0.032149246	0.036535333	0.047077423	0.026597274
0.137412220	0.118714590	0.127410471	0.151553013	0.164422783	0.152572936	0.117793873
0.096806188	0.093494661	0.087854101	0.116878081	0.113309392	0.113922751	0.089874244
0.487934863	0.525104224	0.432882820	0.501385285	0.536724828	0.405114628	0.348136024
0.042293556	0.054675714	0.049987458	0.045271312	0.057661381	0.054822547	0.037433541
0.091860938	0.199970952	0.158617620	0.182158023	0.198102672	0.186360062	0.115389934
0.793254154	0.799736145	0.800215922	0.813739312	0.849705349	0.848059563	0.752264836
0.024191463	0.042740329	0.034159412	0.032288617	0.040298892	0.037531076	0.026846857
0.176759303	0.167263706	0.195762592	0.222376426	0.203652216	0.191827631	0.159924047
0.536718432	0.619001013	0.549907004	0.606672712	0.649451880	0.570902567	0.521567257
0.152508543	0.200378284	0.172080623	0.255007874	0.217552153	0.172046134	0.160168486
0.714992089	0.857802420	0.786134663	0.801127790	0.851891230	0.814775367	0.741977938
0.519304642	0.705604595	0.587482017	0.656347766	0.748728839	0.639011750	0.552669250
0.553128162	0.622105968	0.614230289	0.573478402	0.525703235	0.577806709	0.491919655
0.096855151	0.118539642	0.200682134	0.199645126	0.167336873	0.134830876	0.107187873
0.026704602	0.042494817	0.033937266	0.047907579	0.040016671	0.046234759	0.032152169
0.038562123	0.051528874	0.040517378	0.046418409	0.052732957	0.056926923	0.037792312
0.850935928	0.845007459	0.851892664	0.827456541	0.885154836	0.890099782	0.819558714
0.517711262	0.590891700	0.528624271	0.591765068	0.593315276	0.563074731	0.501172807
0.042267939	0.061754430	0.048594739	0.053259567	0.057406841	0.065315911	0.042176367
0.537048110	0.581143787	0.526693265	0.588828267	0.559357071	0.587744761	0.521736258
0.049257757	0.114828481	0.076025211	0.066494540	0.077290502	0.053616584	0.046952544
0.033748170	0.030674385	0.368930721	0.027741037	0.435589459	0.277826672	0.037350847

0.025919512	0.026266120	0.027234779	0.040674778	0.028262190	0.035082599	0.024366753
0.336570812	0.336038667	0.329730130	0.398245209	0.391133098	0.371560577	0.319918326
0.051184729	0.061004144	0.050499726	0.044792273	0.044418924	0.050315488	0.039666143
0.540144356	0.582751937	0.602970199	0.642652272	0.649768322	0.609963253	0.534735268
0.032939356	0.045340488	0.037982144	0.038471830	0.039381848	0.036588030	0.033248686
0.316052168	0.314775737	0.329008239	0.349992210	0.361773731	0.379671802	0.304185737
0.051062298	0.067468848	0.069168347	0.074757521	0.077395102	0.064217064	0.056106142
0.831306176	0.775756501	0.834452234	0.878843891	0.874364725	0.844087934	0.784556385
0.025183917	0.021004055	0.020231271	0.032341207	0.033666595	0.025517564	0.020598836
0.475987424	0.565474997	0.591608451	0.601928630	0.642691842	0.589069140	0.511796092
0.489045726	0.691776000	0.519227296	0.667417075	0.606764081	0.643437113	0.496076726
0.019109808	0.026793531	0.019535416	0.022659096	0.027179256	0.026522290	0.019696690
0.029677230	0.036064293	0.024718083	0.027585369	0.030972757	0.021434068	0.022396148
0.027916742	0.024365604	0.024413821	0.028171763	0.033347869	0.031034448	0.023729615
0.488448272	0.549705679	0.512295775	0.519248400	0.580414482	0.581206188	0.460151716
0.021448376	0.035652290	0.038459513	0.032756555	0.044042866	0.037362200	0.027975133
0.732181498	0.845318596	0.754824294	0.792096819	0.798742069	0.809225367	0.726656965
0.463097868	0.577716519	0.457467905	0.499498662	0.562385148	0.528547432	0.436956922
0.542031417	0.719240077	0.690170908	0.796001406	0.720198301	0.651102746	0.579171870
0.023261618	0.027825530	0.024858669	0.031748870	0.031062965	0.028760953	0.021702468
0.019707882	0.022562465	0.019912560	0.019913553	0.026170112	0.018446268	0.017319290
0.286054228	0.295975535	0.294875816	0.310440193	0.312677319	0.319724186	0.262407449
0.398053020	0.512094533	0.419051332	0.515902839	0.500076370	0.496866426	0.397500614
0.027212603	0.048266517	0.033249713	0.038413644	0.049891400	0.047127245	0.030756190
0.214341880	0.162976381	0.234474386	0.198342697	0.202822463	0.222248868	0.158128961
0.781980236	0.802665841	0.805058842	0.815674793	0.854732041	0.822948589	0.769953249
0.031888171	0.048549100	0.030397482	0.046198862	0.045181367	0.036106394	0.029415460
0.182554049	0.151917720	0.115687617	0.130854153	0.169207178	0.174154565	0.126133674
0.292356297	0.241019314	0.250995576	0.252098258	0.316148721	0.253988086	0.216797602
0.021243619	0.020433565	0.019144813	0.020828885	0.022478520	0.031515075	0.018850711
0.018974331	0.017043830	0.015463581	0.016168660	0.018522607	0.014925620	0.014444283
0.060346224	0.052760443	0.038310144	0.050960905	0.067161289	0.061352023	0.044071659
0.032478443	0.026629876	0.026378173	0.028644633	0.036659032	0.031707772	0.024836330
0.022746584	0.035474246	0.027929866	0.026723536	0.031631344	0.029310260	0.023568375
0.046110657	0.050799261	0.055922003	0.068915737	0.063185278	0.078149611	0.047371617
0.040309707	0.032133848	0.033583300	0.034510700	0.040322987	0.035656570	0.029622172
0.799895628	0.866410274	0.862424446	0.857120597	0.858444993	0.837601084	0.819311224
0.016569068	0.013722080	0.017618366	0.019087171	0.020168476	0.034052692	0.015194776
0.793390561	0.741130874	0.815629277	0.830457888	0.736210803	0.827170341	0.833331718
0.031911774	0.047249149	0.044172030	0.053305891	0.065881363	0.043812995	0.036827296
0.028325235	0.034445013	0.027583668	0.026973219	0.035130828	0.032435617	0.025853010
0.029089821	0.040195359	0.036821831	0.066398067	0.034803748	0.052188300	0.033063911
0.029472362	0.032862431	0.030702938	0.032152931	0.033316663	0.024625791	0.025622809
0.030060366	0.046381542	0.037225043	0.040548115	0.052537731	0.040351276	0.033779686

0.634702375	0.817140678	0.721121420	0.754356203	0.813179391	0.733262140	0.652991007
0.503099234	0.561751152	0.570778185	0.598776297	0.642246917	0.598625209	0.519602727
0.449573375	0.595363899	0.512654296	0.583227105	0.605297514	0.518017379	0.450874299
0.020992963	0.022402812	0.024523293	0.029456727	0.025758738	0.025014934	0.019202703
0.511064203	0.580821316	0.589186506	0.597784895	0.613231233	0.598831421	0.522987907
0.073067006	0.059690196	0.048725241	0.073016643	0.059860010	0.061628450	0.050713067
0.649284432	0.715956276	0.700414555	0.687066432	0.785909360	0.770194504	0.657761299
0.059191121	0.058062825	0.074247925	0.075471474	0.069097720	0.101201096	0.053253279
0.578395830	0.621671506	0.603310809	0.647053476	0.660450812	0.681710851	0.585878274
0.770972267	0.787097084	0.814209193	0.827011409	0.861109561	0.822979510	0.767532351
0.084715020	0.081835721	0.070485341	0.089785308	0.091613277	0.091783816	0.070916677
0.386099844	0.448491409	0.410751782	0.503251801	0.506296382	0.474877918	0.397575672
0.017935878	0.014624552	0.016058101	0.018391266	0.016922371	0.018962363	0.014976713
0.140431416	0.149549961	0.179779256	0.207304628	0.087326794	0.229436015	0.123515260
0.037730248	0.045384915	0.031377222	0.031742778	0.041456413	0.029688891	0.030208952
0.625318218	0.669718398	0.621530900	0.766867535	0.766890057	0.686905343	0.610586321
0.140031760	0.240309114	0.116434693	0.170312679	0.234080196	0.197264756	0.103757305
0.032737970	0.041629185	0.054632068	0.054827696	0.041795936	0.054958379	0.035627454
0.034727218	0.037101691	0.041265108	0.033344760	0.049945127	0.038435982	0.028876395
0.569963510	0.731834066	0.644840625	0.747180956	0.792816536	0.742276403	0.638407373
0.063187189	0.089909891	0.068779460	0.084586903	0.077743815	0.075889371	0.060412066
0.046691835	0.046461766	0.049712412	0.045769586	0.054126144	0.064274303	0.043391503
0.566369540	0.713408547	0.727169883	0.687060021	0.667199619	0.719301989	0.603784334
0.852195407	0.893016927	0.451322091	0.898415182	0.548036148	0.755348915	0.452373038
0.855055577	0.879760164	0.871726512	0.890422765	0.901006645	0.884929926	0.857019583
0.067919176	0.068454303	0.059818385	0.069712329	0.077110041	0.107763040	0.059037746
0.600919338	0.710951477	0.664199209	0.685265524	0.734660074	0.712676498	0.642264308
0.035130997	0.046662012	0.041070969	0.055514482	0.053698964	0.047072936	0.035571411
0.034842537	0.098608450	0.046477833	0.057458817	0.050206904	0.050498188	0.038872187
0.035908399	0.072161605	0.054995526	0.050885009	0.075107643	0.065353129	0.045169170
0.031080276	0.031740819	0.028576956	0.034949275	0.033341737	0.029745440	0.027035101
0.028464109	0.031408585	0.032209679	0.037477190	0.036786116	0.038604848	0.026284907
0.053074945	0.043356468	0.039287749	0.039988304	0.073268427	0.047029292	0.037518595
0.063646920	0.059459277	0.065093950	0.043295304	0.044054670	0.061703522	0.089317957
0.022887579	0.016936866	0.016673553	0.028239765	0.017499403	0.018984853	0.015870203
0.411532703	0.498745462	0.470789995	0.591892830	0.537851625	0.528204450	0.403751793
0.042376195	0.069188955	0.475837602	0.102796709	0.559706778	0.311509984	0.058833150
0.052511165	0.061811804	0.049763701	0.068510556	0.057490895	0.071536552	0.047508955
0.058469582	0.041111045	0.042067134	0.033575869	0.048782910	0.065196845	0.038512288
0.221215556	0.197243098	0.185710069	0.253505884	0.225161717	0.244261764	0.186899792
0.066428632	0.082120311	0.066003892	0.075756491	0.102723074	0.077101405	0.065236567
0.151285242	0.223931578	0.149998084	0.229879792	0.277162811	0.243054390	0.148601652
0.729085874	0.812700985	0.747460951	0.782642896	0.801676147	0.765326785	0.699973782
0.164849598	0.244859625	0.193987175	0.318628675	0.317410365	0.224318716	0.172191040

0.751104940	0.800130405	0.749637021	0.801656510	0.772421350	0.831829159	0.734324620
0.041731321	0.034436004	0.029557923	0.031600308	0.031153325	0.023940382	0.026816135
0.472063659	0.577633158	0.519360485	0.492923898	0.576471666	0.569261908	0.471717772
0.521871180	0.638390395	0.516642934	0.616196763	0.634661739	0.580831821	0.519844115
0.062967052	0.057999390	0.053601215	0.075520056	0.059223497	0.069384544	0.050670878
0.027941865	0.041497450	0.038460801	0.026427412	0.036505179	0.026367928	0.025887748
0.753436775	0.861163696	0.830052666	0.845484526	0.840777917	0.835206659	0.754456585
0.037847209	0.045668827	0.039294639	0.048691906	0.077648800	0.057949804	0.038562721
0.607731034	0.573401224	0.592226444	0.634078227	0.639253827	0.642725546	0.527844477
0.018926970	0.025240472	0.020878042	0.020648229	0.022326044	0.030733093	0.018241211
0.125873916	0.083748600	0.110584629	0.079674530	0.095354152	0.147678613	0.081323192
0.497296204	0.532266364	0.569809583	0.526302534	0.549080453	0.496740141	0.487076559
0.321057643	0.347309130	0.347591595	0.378296224	0.394062248	0.394611158	0.331953072
0.242226230	0.231295958	0.207181466	0.224911049	0.240196356	0.239130865	0.196042382
0.056054287	0.112373773	0.079223555	0.078998656	0.142441995	0.079089478	0.057443575
0.599084200	0.655710882	0.652001061	0.658168319	0.714257707	0.704981948	0.607397257
0.170550021	0.143489177	0.160068192	0.212232624	0.198457897	0.180825058	0.133316152
0.491507855	0.546303611	0.525370068	0.554245870	0.597213567	0.599930908	0.497692012
0.384916551	0.407188178	0.431612185	0.449184158	0.473596092	0.444927552	0.385177098
0.023664510	0.038414986	0.027997670	0.034126789	0.038563203	0.030099855	0.024254994
0.613947546	0.644043986	0.634937098	0.639170198	0.682599035	0.718632489	0.607633960
0.163899160	0.149404511	0.106591918	0.133839460	0.186636307	0.156751870	0.122528544
0.067950296	0.091203934	0.073470454	0.079355874	0.084486703	0.077549834	0.063036019
0.031584296	0.046626950	0.030521399	0.030099942	0.058399728	0.028627324	0.024178806
0.870102922	0.927562130	0.945373562	0.918227684	0.925201529	0.920516825	0.941814550
0.033810047	0.061182203	0.040875329	0.050706875	0.065092650	0.056711505	0.039679194
0.027571519	0.037175656	0.039957622	0.024795780	0.032366542	0.029460413	0.025603194
0.912543332	0.926331799	0.929135415	0.936732423	0.922809794	0.935396025	0.903117049
0.883961241	0.851624022	0.847453589	0.877707593	0.881384650	0.887257215	0.891997360
0.888801406	0.921597688	0.886239024	0.930007276	0.934281093	0.906848860	0.874341815
0.796110931	0.821999174	0.824253481	0.870392051	0.848958572	0.824612958	0.795420518
0.059937960	0.044858810	0.046445653	0.047481088	0.048948420	0.049267170	0.044005463
0.026784180	0.031052514	0.028136694	0.026160995	0.039865528	0.030007230	0.024410437
0.485257846	0.583441320	0.510556191	0.564399293	0.584284542	0.542607561	0.490251030
0.481952514	0.543490194	0.575655008	0.553040381	0.655377571	0.617913211	0.499469613
0.040530909	0.058770546	0.045916190	0.055171120	0.054133369	0.037345171	0.040159547
0.408660218	0.497722588	0.396884965	0.457556974	0.463396356	0.480049969	0.399259923
0.030085724	0.031416756	0.037981170	0.039377509	0.039787664	0.044261819	0.030163894
0.077346762	0.079937914	0.062614654	0.085446502	0.087920755	0.082547160	0.066884002
0.433942655	0.454847363	0.419748822	0.535376286	0.583097739	0.550084464	0.423785463
0.026445534	0.043906108	0.025459340	0.039588771	0.035490706	0.035995960	0.026833493
0.049672191	0.066327223	0.083015955	0.076315961	0.065259137	0.075604810	0.057654765
0.290834643	0.311998363	0.287711499	0.374248494	0.325659667	0.376634222	0.284077055
0.875493622	0.909640253	0.895225444	0.921170739	0.931973817	0.921222027	0.882789065

0.039206387	0.088486527	0.090412868	0.078638211	0.083160962	0.066683412	0.051492129
0.322172254	0.259231842	0.287270856	0.267367825	0.378852457	0.375571534	0.271053959
0.034199136	0.032485484	0.034403192	0.032902346	0.038008316	0.030988710	0.028874238
0.434518006	0.480915244	0.463992888	0.497428987	0.533669381	0.541469133	0.434669923
0.032173864	0.047597838	0.038871370	0.041104630	0.050243081	0.040392751	0.032524547
0.329947821	0.339571881	0.330572346	0.342604619	0.361480322	0.356163629	0.308066459
0.342174765	0.357571976	0.273915563	0.327495556	0.363785537	0.486839193	0.303713577
0.024974710	0.045363206	0.037626873	0.053061540	0.051992168	0.036925475	0.032444922
0.125692657	0.115319572	0.138374792	0.152836296	0.143847182	0.108336120	0.110192188
0.027548457	0.056613900	0.038636813	0.038098274	0.069358664	0.041219237	0.031458568
0.032913356	0.031407309	0.029128581	0.030370738	0.041420970	0.037275459	0.028953685
0.033396875	0.036464550	0.044021729	0.049615125	0.045038768	0.046958685	0.037441578
0.088950496	0.159727330	0.110529564	0.102005378	0.122096534	0.075647489	0.086378674
0.859448368	0.844892709	0.860896784	0.849646956	0.870441603	0.848439121	0.886971484
0.065160268	0.061125449	0.040563625	0.052652550	0.074370997	0.064386179	0.047125769
0.031143786	0.037215911	0.049545888	0.040721478	0.038922582	0.049666196	0.032029059
0.963342074	0.952982390	0.960526135	0.955872423	0.953259122	0.950483397	0.965261227
0.050438723	0.055103685	0.069400183	0.075951599	0.055037593	0.059920474	0.052262544
0.042680718	0.057066987	0.033345944	0.061416305	0.058922635	0.047665013	0.034631329
0.945924829	0.951883705	0.940577213	0.919992350	0.927988088	0.822935962	0.859333741
0.037125099	0.036778519	0.041868793	0.039370738	0.034754107	0.038761237	0.029959868
0.058920491	0.053865455	0.055331599	0.056730406	0.055354266	0.057858785	0.047985026
0.905770909	0.871650780	0.908855456	0.910478071	0.867009449	0.911896760	0.923582374
0.052876945	0.042268151	0.032701541	0.042412889	0.058523160	0.052786943	0.034943116
0.674887291	0.669982431	0.636138035	0.704649753	0.682331676	0.677713687	0.591550891
0.426580348	0.425460821	0.491855837	0.460410056	0.479721689	0.503785438	0.422843643
0.034546857	0.026352373	0.031132044	0.035687627	0.029058657	0.040203914	0.026474252
0.027175266	0.028803898	0.022913759	0.022052759	0.030396736	0.030483421	0.021521997
0.417610846	0.452079803	0.446208223	0.556501265	0.491919236	0.545004488	0.419495846
0.050254210	0.114824937	0.072818827	0.095130937	0.129039621	0.101853359	0.063483904
0.064333700	0.057675375	0.054948712	0.061670084	0.071796271	0.072930311	0.054329253
0.055036402	0.069981980	0.050057173	0.056602370	0.051389117	0.068980293	0.046488285
0.047481558	0.057941969	0.061939174	0.052977399	0.088523701	0.050871977	0.043462701
0.424245331	0.540409060	0.482672766	0.560882832	0.543305185	0.525280473	0.444111196
0.024745837	0.033286080	0.024828849	0.024541467	0.024753340	0.025318965	0.021435567
0.022781976	0.022307260	0.020123516	0.030344552	0.030201305	0.031985098	0.021728646
0.814457578	0.884259469	0.879251605	0.882468337	0.889935875	0.880683896	0.822742458
0.408130283	0.195988499	0.042227027	0.053354641	0.069281128	0.062947575	0.206712654
0.019817599	0.037307488	0.032002894	0.030981602	0.028110044	0.038749421	0.025193116
0.057610889	0.128015438	0.090775918	0.102433710	0.092392461	0.102920717	0.067569307
0.057664307	0.064118353	0.060078843	0.070479488	0.092462668	0.081705014	0.056661042
0.226408863	0.203665464	0.138777059	0.254163355	0.235066157	0.247070725	0.164075404
0.519443675	0.427937723	0.647741018	0.325518101	0.574667164	0.446254395	0.613524724
0.841140533	0.847600406	0.854315677	0.852811007	0.879837317	0.891714293	0.813339667

0.032644500	0.018525596	0.029335986	0.030304744	0.037379254	0.033914967	0.023270687
0.032849155	0.030180308	0.022415347	0.036675200	0.032596247	0.041204967	0.028132260
0.545427719	0.612531406	0.534890708	0.654627699	0.628426609	0.610558760	0.544410514
0.790745372	0.790270986	0.800644290	0.790397830	0.818861773	0.819379496	0.741626481
0.771279099	0.873660823	0.859915559	0.865222761	0.867505667	0.856990883	0.802918669
0.026276955	0.022987980	0.015707178	0.024144717	0.022153729	0.023809839	0.018494723
0.779789469	0.789249591	0.404366312	0.390581141	0.767073134	0.687647421	0.833568713
0.041990617	0.075934843	0.051438199	0.094323860	0.066480167	0.079319654	0.043251030
0.048816585	0.072934425	0.047634111	0.055490486	0.101911617	0.066668430	0.049915213
0.792927376	0.821593524	0.853725695	0.768166626	0.877383073	0.815196318	0.777542526
0.139599097	0.146182691	0.133940032	0.151640379	0.159487511	0.170060522	0.128689180
0.546574630	0.633119701	0.541356545	0.568406157	0.560753662	0.523997836	0.503823416
0.045943980	0.036061149	0.032116898	0.043162679	0.049851251	0.056983417	0.037307589
0.476435352	0.576709855	0.508772874	0.604449376	0.561434857	0.542626996	0.490574945
0.647526678	0.845739708	0.722756640	0.753784607	0.757260436	0.747414644	0.670140868
0.427261903	0.462320421	0.416088634	0.538298955	0.486105439	0.492799677	0.405699028
0.820769960	0.858277381	0.838510698	0.881367711	0.868863321	0.872879668	0.829888738
0.044379610	0.046898113	0.057859174	0.048173833	0.047126836	0.064569548	0.042162779
0.828670784	0.877743271	0.836546657	0.879979948	0.903019473	0.893333232	0.843612842
0.252270751	0.303779107	0.312255022	0.299728671	0.414081516	0.292172093	0.259667607
0.036830390	0.104425681	0.094016058	0.096088940	0.152123412	0.100622881	0.057880340
0.149352685	0.129691089	0.125860615	0.146689862	0.160860850	0.189013828	0.114127417
0.029250732	0.038439593	0.032544449	0.031053437	0.041809910	0.032699681	0.027168691
0.023702207	0.024819068	0.020248283	0.024025094	0.028067493	0.024269914	0.020512888
0.875374443	0.877167916	0.905256145	0.918156670	0.908883511	0.911628888	0.922588053
0.054454242	0.065217930	0.056694477	0.039498234	0.061894347	0.069954292	0.044348547
0.852573984	0.901355522	0.847005946	0.861928878	0.913430842	0.889568111	0.853379121
0.084069993	0.072954946	0.068212880	0.061636073	0.075810276	0.087417557	0.064014563
0.022756274	0.022962767	0.020225133	0.024469175	0.021751104	0.021520790	0.019411100
0.042489201	0.048046942	0.025244624	0.052960443	0.041220274	0.052765070	0.034186610
0.358376911	0.562433273	0.418504325	0.615249904	0.484434843	0.420826059	0.382756831
0.056915318	0.132820664	0.113903994	0.117275949	0.098639709	0.085761028	0.063447951
0.863597368	0.859538631	0.864987088	0.886507870	0.891569623	0.896995482	0.857299229
0.025016019	0.026659339	0.026400674	0.025288646	0.029205357	0.030465860	0.022544353
0.024173284	0.031338775	0.031406634	0.031224503	0.034201399	0.028459642	0.023547182
0.820135415	0.891429245	0.900665017	0.899538514	0.897835092	0.898351685	0.852268581
0.068085904	0.063934177	0.061453297	0.075233469	0.066833378	0.078515000	0.061157493
0.597388653	0.766698182	0.612038362	0.632653335	0.668829284	0.670649499	0.568237276
0.086290373	0.092001090	0.070550064	0.276522085	0.137780063	0.083786986	0.069928491
0.350880074	0.438434827	0.406557017	0.348096836	0.414524691	0.379977857	0.311548811
0.398396101	0.478642109	0.422263969	0.538267051	0.597513742	0.516400809	0.405776144
0.041661449	0.047881571	0.046818323	0.051669073	0.041326132	0.050817367	0.039537840
0.914968806	0.928416996	0.918119895	0.930758235	0.928970599	0.933746801	0.905472122
0.732637774	0.772564389	0.798356484	0.829988841	0.849362182	0.794702559	0.740720427

0.863101202	0.924535650	0.919352894	0.930135149	0.944278291	0.886101244	0.880837372
0.420165859	0.400124800	0.420618639	0.426035314	0.501606627	0.470084203	0.395736803
0.029678789	0.023191783	0.020860623	0.029148000	0.030526458	0.030769653	0.023644003
0.019691851	0.025836016	0.024424654	0.021209062	0.029991597	0.022977728	0.019332277
0.293499224	0.389278670	0.355754406	0.384429601	0.356935958	0.328222849	0.280560376
0.692799817	0.733412180	0.724429455	0.716150957	0.708550131	0.747276139	0.780991178
0.402922012	0.452066257	0.381995792	0.398710830	0.462796836	0.437565599	0.369150225
0.333356212	0.463139307	0.431018324	0.436854596	0.367142242	0.364486168	0.315509739
0.775646341	0.859710872	0.830939802	0.873373062	0.911766548	0.847462389	0.796150583
0.025821000	0.031878215	0.024535312	0.037572986	0.037393768	0.030666940	0.023320423
0.513208564	0.603719861	0.605757148	0.611742362	0.649720087	0.620910558	0.540622650
0.223688254	0.185902033	0.172706018	0.195818174	0.226239968	0.215423077	0.172854207
0.046950090	0.051390787	0.064686486	0.061425485	0.090601771	0.053595135	0.045322031
0.020915097	0.021952293	0.020398900	0.027036631	0.021521316	0.027236051	0.019530758
0.033531344	0.033974165	0.025374351	0.029497385	0.037868689	0.030201298	0.024220929
0.885724065	0.910257088	0.868202318	0.894377636	0.876776029	0.918889114	0.877473903
0.054831177	0.051956155	0.056930144	0.054192447	0.060114117	0.054957227	0.047984521
0.755033183	0.750516642	0.754602639	0.757939938	0.783783441	0.788706623	0.718800367
0.068226166	0.177971353	0.143835850	0.165077726	0.166794969	0.183088638	0.098622807
0.808970510	0.867588178	0.822489937	0.870980152	0.870548792	0.883882644	0.816527339
0.225044257	0.194497651	0.186788251	0.205711246	0.298109905	0.255366636	0.190071473
0.073084654	0.078187944	0.073621811	0.077810109	0.095622298	0.111718307	0.064974391
0.511969511	0.634917709	0.549006743	0.634800849	0.653510113	0.598314088	0.523289479
0.875028011	0.893593712	0.899847980	0.901751456	0.889959121	0.896928289	0.852384764
0.074420706	0.141352328	0.108481754	0.101032641	0.111725297	0.086914125	0.075657040
0.027544908	0.046671914	0.036768739	0.039798594	0.059313026	0.036002568	0.028692271
0.085279497	0.063140258	0.056090199	0.067406137	0.077405020	0.063337808	0.052626934
0.029456481	0.021946149	0.021822983	0.017958309	0.023777238	0.018443814	0.018051878
0.240346723	0.216937456	0.204635136	0.217165691	0.252617604	0.254615911	0.207830192
0.589654174	0.656340916	0.650555323	0.679156460	0.687592807	0.712242847	0.612471577
0.430443665	0.533520036	0.532793644	0.574182089	0.577080437	0.510209169	0.458082614
0.067934206	0.052487608	0.054412380	0.056521386	0.066423291	0.057902791	0.049350578
0.776566309	0.836111627	0.821226999	0.830570520	0.852826070	0.847374972	0.776024162
0.021699917	0.037170413	0.028181798	0.033726589	0.032833741	0.027083501	0.025123501
0.823140740	0.820876381	0.797858498	0.819569331	0.863328524	0.834638115	0.783148039
0.046821445	0.063082843	0.047970268	0.062806238	0.076060001	0.089159371	0.052851648
0.038633512	0.046363037	0.046391859	0.049417432	0.066988077	0.062537336	0.042071591
0.099841789	0.141668293	0.162819909	0.138436375	0.119853544	0.115816828	0.150692690
0.060745307	0.049763337	0.050660630	0.057791145	0.048947107	0.057396538	0.046550067
0.166731203	0.118858475	0.133605780	0.099929716	0.176843683	0.191154760	0.122973607
0.540832875	0.546167426	0.550611451	0.600937243	0.580454238	0.602005764	0.510636046
0.044326375	0.113935634	0.073267820	0.086461605	0.114624529	0.082578046	0.060325836
0.134998007	0.137496785	0.116967427	0.129458180	0.194263119	0.151310128	0.124616932
0.023193652	0.048239565	0.027698915	0.031309967	0.053005898	0.035445907	0.025449528

0.775833520	0.902610023	0.869909767	0.859823222	0.896246715	0.801278209	0.805660844
0.881412037	0.903537715	0.884042688	0.911024214	0.919703822	0.924956879	0.875640407
0.054237195	0.094331140	0.094155377	0.086543316	0.083764182	0.072012220	0.060324272
0.024522236	0.024070021	0.018551300	0.060105581	0.028459486	0.023680184	0.019392984
0.046818609	0.033219921	0.032700569	0.039851871	0.037686680	0.041533935	0.032344308
0.039981032	0.036527833	0.030357411	0.033331656	0.035705568	0.040753977	0.030404787
0.400492546	0.623980033	0.428479095	0.473418727	0.532184053	0.460695021	0.401672507
0.033323471	0.040989251	0.036855183	0.042081974	0.043311562	0.044072683	0.034722262
0.029462820	0.050351567	0.039333258	0.044451770	0.038242865	0.034705072	0.032264631
0.029728160	0.046941497	0.032381100	0.035612228	0.048219029	0.044644645	0.029889012
0.027371602	0.046013397	0.042140586	0.043386164	0.052721338	0.045681737	0.031578955
0.193446796	0.159320531	0.111811083	0.188718730	0.181646305	0.149165836	0.123881804
0.756335532	0.868777503	0.800675861	0.870036109	0.853020456	0.890792877	0.778691071
0.015991301	0.011692769	0.013546919	0.012938693	0.014199816	0.017687290	0.011391101
0.302307458	0.311975929	0.297298357	0.298831229	0.347488435	0.369982585	0.289601917
0.061148325	0.084680261	0.058017695	0.070217443	0.074209197	0.063609292	0.056404291
0.296201624	0.299314827	0.311605555	0.273699629	0.330547850	0.330577036	0.262835577
0.056812354	0.101925477	0.085921363	0.108328610	0.096764669	0.096400513	0.064829774
0.031633351	0.035663297	0.034562782	0.041888900	0.045424338	0.041618973	0.029856545
0.039856781	0.045319647	0.037869613	0.052315457	0.074619422	0.046343771	0.037298862
0.028432825	0.029580321	0.029074119	0.033855146	0.038275464	0.039326257	0.029508762
0.112035399	0.166432141	0.223175266	0.148671992	0.230131669	0.139445759	0.117940533
0.891780982	0.877531175	0.904833810	0.904268838	0.921450768	0.918433332	0.876469479
0.043418459	0.118406299	0.062691449	0.080157009	0.101875605	0.083820496	0.051798522
0.026516891	0.024763804	0.030434725	0.035919354	0.032551295	0.032916935	0.026527158
0.104124323	0.108537279	0.081134304	0.079820281	0.103471483	0.103289849	0.073908453
0.032510414	0.059747117	0.059243125	0.064181959	0.064174753	0.070281371	0.038311062
0.476595322	0.568529930	0.464160171	0.642179531	0.611536459	0.526134826	0.457997671
0.033015463	0.089866744	0.067656459	0.082266156	0.099555514	0.069331230	0.046843379
0.043587700	0.048894324	0.039662309	0.037990405	0.039483578	0.041816296	0.035499881
0.068332631	0.069450761	0.045120226	0.080571550	0.070383787	0.076794106	0.055815011
0.035783178	0.026509042	0.029812345	0.022939638	0.025990754	0.020822705	0.021636024
0.301525596	0.403372138	0.430280656	0.469228831	0.460044929	0.364036061	0.325170267
0.909239266	0.934951616	0.918693292	0.924363953	0.938789255	0.946887526	0.914575290
0.365838546	0.467836584	0.437746435	0.504880819	0.504745907	0.500822250	0.387000541
0.283264658	0.319341465	0.218889292	0.237730622	0.252606750	0.291397089	0.209468933
0.443759542	0.551469748	0.463673588	0.611522547	0.679344499	0.526405307	0.439182616
0.187825203	0.207279997	0.181491695	0.221261756	0.182615996	0.224648182	0.171851704
0.882648142	0.892877057	0.877971716	0.906260477	0.895765174	0.894321068	0.859782197
0.038857315	0.102003942	0.060310254	0.068743739	0.084265285	0.076307641	0.048356512
0.106309776	0.097932716	0.090192492	0.091711797	0.109545097	0.083538603	0.082172275
0.620489242	0.624601954	0.632884711	0.645542834	0.673575370	0.689537106	0.619559196
0.215116715	0.176036167	0.056366039	0.067088052	0.078645952	0.078534897	0.159780757
0.023566999	0.021607085	0.029213579	0.020627079	0.022169329	0.030217526	0.021229107

0.593191568	0.581137756	0.554422083	0.554792708	0.548839862	0.571405515	0.510892809
0.038607007	0.035908080	0.023007000	0.020397729	0.030768878	0.028072131	0.022925944
0.038510892	0.026649615	0.032303330	0.042851540	0.038669467	0.044056095	0.028243558
0.027624423	0.033172796	0.023381611	0.055063892	0.032271080	0.040135203	0.026306238
0.065031803	0.072441512	0.064395005	0.077081216	0.088013621	0.077105978	0.063312402
0.064456241	0.033512662	0.035061748	0.042115339	0.063280797	0.044777954	0.037400648
0.016816533	0.018649502	0.016064686	0.019766749	0.019491327	0.021693683	0.015720100
0.709214655	0.732187843	0.734715526	0.746166606	0.771058403	0.762706611	0.707007308
0.027673807	0.033184069	0.055634256	0.032376570	0.037145288	0.038593064	0.028709325
0.021423669	0.028177449	0.030283098	0.022840071	0.034032288	0.026816810	0.022354847
0.209434539	0.253496643	0.172254963	0.159715571	0.276627330	0.295634214	0.179900105
0.932999992	0.922452452	0.861791099	0.942137205	0.936767891	0.914572351	0.938396289
0.039858148	0.094434820	0.058331820	0.062657565	0.099525776	0.054429367	0.046207492
0.037069256	0.032113253	0.036207180	0.045758058	0.036165624	0.045578143	0.030247647
0.783827050	0.886301844	0.855652937	0.864028321	0.860999785	0.850631529	0.790386516
0.615429637	0.605952322	0.634533890	0.651086885	0.682406096	0.682183652	0.589639709
0.039177825	0.040170586	0.049408004	0.065926104	0.057884574	0.061919759	0.039592089
0.031031579	0.031164422	0.026211292	0.042401309	0.037365889	0.046778789	0.026054489
0.044034690	0.054620080	0.055960395	0.055392472	0.070002481	0.070061671	0.046412252
0.486159453	0.577865975	0.532238828	0.472739813	0.565684686	0.590542744	0.471633144
0.104250349	0.188731264	0.151587397	0.235899267	0.270912482	0.167315018	0.130667398
0.066959318	0.200675671	0.158295239	0.180189283	0.222932286	0.124521725	0.100292531
0.035429381	0.035097318	0.064896839	0.107926760	0.051602844	0.087576086	0.036426615
0.749884936	0.696667551	0.675951846	0.813657862	0.819913918	0.786556977	0.669939833
0.040055989	0.129237086	0.081827662	0.071801214	0.085382004	0.078920339	0.054366111
0.037685601	0.036931485	0.033284954	0.046633331	0.038294242	0.047992256	0.031694732
0.885780584	0.908574130	0.880552942	0.909735605	0.927904953	0.934180544	0.878300878
0.030405388	0.032500105	0.027393824	0.041669410	0.031228855	0.034915554	0.026552292
0.024768970	0.032252152	0.032360534	0.035885078	0.031466342	0.031679845	0.026225802
0.034547836	0.052561203	0.037323383	0.054919071	0.060398212	0.079839765	0.037009576
0.016739432	0.014210870	0.012819871	0.012170567	0.016551020	0.017374166	0.012618919
0.943674410	0.947891190	0.950527193	0.951375222	0.958450627	0.961164780	0.958102571
0.631738839	0.784682626	0.698317882	0.795495612	0.845351649	0.705927540	0.650708983
0.029876932	0.045767251	0.032879585	0.035234659	0.040568663	0.040597678	0.031065972
0.068289135	0.095478845	0.067570304	0.061233149	0.083352327	0.048481167	0.057453032
0.022679665	0.036488694	0.025838355	0.027222785	0.038412278	0.035152692	0.022694076
0.039195460	0.037504421	0.052490270	0.053419364	0.049223600	0.049135806	0.038760425
0.287481453	0.258299060	0.195274283	0.217374659	0.252009880	0.166932822	0.184671228
0.022747484	0.026846167	0.021364365	0.023283463	0.031830861	0.021307742	0.020056905
0.018694037	0.015988413	0.019158793	0.018174907	0.016564613	0.016865437	0.014648965
0.488375368	0.663136540	0.592117172	0.631096010	0.647797452	0.570075182	0.529367825
0.038629590	0.067916292	0.045761506	0.049926051	0.062309123	0.046631071	0.040880589
0.187645188	0.189900222	0.149942239	0.209202904	0.213659870	0.219185374	0.168162319
0.032065644	0.035251944	0.037531808	0.041838588	0.036022523	0.048036164	0.030980676

0.032865716	0.031909494	0.043433176	0.041369973	0.056748354	0.032455789	0.028769617
0.208023412	0.268185620	0.275108922	0.365873725	0.290233375	0.310512272	0.231284261
0.171447775	0.257936528	0.189519687	0.201926646	0.213360038	0.217104139	0.163955187
0.167082052	0.068793013	0.092141580	0.093836968	0.095487093	0.086469665	0.071755264
0.036577863	0.098529110	0.056343076	0.071290045	0.111733679	0.068487412	0.047428573
0.081437507	0.068955649	0.073747278	0.088277919	0.091197126	0.088850338	0.069346198
0.018987904	0.018784927	0.016826155	0.021051375	0.022794444	0.026748303	0.016384678
0.031598042	0.029042712	0.027708466	0.035007206	0.026671713	0.029052025	0.023123431
0.412019848	0.618125751	0.448648226	0.555144502	0.519680696	0.547264282	0.421526780
0.904699700	0.893084185	0.913443005	0.924427967	0.939755775	0.933404746	0.898067956
0.049652051	0.039069499	0.036930975	0.046172192	0.045985693	0.041472385	0.037810840
0.050394915	0.155417262	0.075826704	0.090582266	0.075702820	0.210442801	0.066244139
0.726573949	0.848315754	0.853592230	0.838431696	0.809784652	0.865770649	0.770873262
0.377209970	0.423750847	0.508324113	0.421458772	0.485752366	0.476402657	0.395366540
0.454791461	0.442826385	0.474668436	0.509643411	0.520802554	0.518181386	0.416176009
0.838552586	0.883803600	0.884638935	0.904431827	0.896851389	0.898427350	0.858465818
0.027928020	0.036565939	0.026860788	0.031003777	0.029243994	0.036171827	0.024263898
0.225845560	0.237447201	0.153526273	0.203230974	0.226136728	0.209148337	0.160253365
0.055044273	0.043506763	0.051135442	0.049376948	0.058746379	0.049523000	0.042529368
0.268444838	0.275901291	0.225365950	0.228200573	0.334280909	0.274702256	0.220980698
0.022482480	0.022979438	0.023962093	0.027360652	0.028797213	0.025429202	0.020982188
0.669149001	0.671708491	0.683947202	0.737709683	0.706191057	0.733149428	0.642149031
0.036067905	0.054293221	0.041428146	0.047250964	0.057886259	0.050101576	0.040458518
0.054711354	0.110750757	0.078494704	0.069712449	0.123042567	0.077107502	0.058066631
0.034814567	0.047751695	0.055959314	0.051419190	0.081846126	0.049122785	0.037512668
0.195001802	0.294794923	0.197995246	0.227814196	0.282055232	0.235038119	0.185813969
0.033492273	0.037601359	0.034363666	0.036637183	0.035378170	0.045788946	0.032628243
0.026398211	0.038299266	0.031140057	0.031859548	0.057601940	0.032365003	0.025541012
0.048420662	0.038561152	0.040963475	0.045073440	0.038304780	0.051517754	0.036241296
0.943672895	0.926488678	0.938271359	0.950843097	0.942149191	0.884298060	0.951121736
0.020299774	0.035410093	0.034554831	0.031962914	0.028793748	0.045919338	0.025081909
0.042728148	0.057381769	0.046465311	0.052850259	0.059793776	0.054938948	0.045797476
0.215113175	0.310944185	0.258607103	0.298529452	0.279750342	0.260043734	0.232796846
0.135516849	0.213189823	0.128203159	0.147202279	0.158523439	0.217222039	0.116333531
0.029045172	0.053578684	0.036195526	0.055630371	0.052848602	0.036115575	0.030824856
0.030111528	0.023310137	0.026796404	0.028133258	0.027031911	0.044809181	0.025026774
0.190605599	0.223266305	0.126751947	0.164138992	0.197356148	0.203021446	0.145559621
0.289710379	0.289595944	0.217255257	0.289261118	0.304653544	0.327382443	0.240615853
0.019935517	0.035790553	0.030627833	0.032015612	0.037211952	0.026565801	0.022548303
0.859760284	0.830640392	0.906199197	0.890147226	0.933937031	0.917588715	0.913282978
0.467581644	0.447195893	0.425301219	0.512405090	0.602916269	0.525067371	0.430364831
0.905219964	0.839851252	0.899190876	0.902729957	0.827152846	0.929357396	0.912562198
0.029574340	0.015832783	0.020272894	0.019556687	0.018873248	0.021290927	0.017222556
0.259107278	0.316668512	0.246259944	0.252397494	0.368353629	0.299409599	0.235735407

0.026285556	0.017521610	0.016295667	0.018506735	0.023364194	0.019346350	0.016242784
0.021196558	0.025094063	0.016459626	0.017063807	0.028218135	0.020279091	0.018086975
0.049696734	0.127547119	0.065231274	0.076587935	0.095920780	0.070332489	0.055104478
0.026844159	0.035239866	0.030071758	0.027492082	0.032579222	0.030664784	0.026123469
0.066939761	0.088554974	0.067020296	0.063524595	0.068530175	0.066304262	0.056859528
0.025726687	0.027758830	0.019134358	0.028752755	0.029154539	0.026295182	0.022252541
0.812592162	0.908997416	0.863793157	0.856714573	0.880230904	0.802731811	0.810566543
0.061782261	0.056188840	0.039833572	0.052911220	0.054506382	0.068858981	0.045056843
0.028623747	0.019342093	0.018089603	0.022771060	0.021816507	0.020634508	0.018522130
0.662212556	0.654484566	0.643210151	0.640990872	0.638513866	0.639588436	0.602229159
0.382027391	0.435157158	0.433359257	0.443120721	0.568749990	0.486067866	0.425947246
0.745493202	0.838896963	0.815973939	0.811868316	0.838789494	0.833365991	0.766569768
0.567930190	0.648699547	0.624753672	0.558644113	0.631392399	0.652589173	0.551147302
0.417519696	0.506612648	0.473396095	0.484017377	0.552008034	0.513392684	0.437716974
0.035322314	0.028395374	0.024240779	0.032508039	0.038518868	0.029794011	0.025508546
0.026872968	0.027194765	0.017502934	0.025147273	0.031862754	0.028715077	0.021394435
0.811946421	0.904307564	0.881870785	0.862890860	0.919907103	0.851135027	0.841452548
0.028206693	0.026662990	0.025322360	0.028737417	0.025527399	0.027319629	0.023213976
0.022979375	0.027853830	0.021549542	0.027117178	0.024204765	0.027691878	0.023004136
0.038549070	0.026963313	0.024513568	0.027603383	0.028698189	0.031174117	0.040330069
0.078933641	0.070719467	0.061318294	0.077457084	0.085388294	0.092275726	0.066629072
0.632065154	0.786246364	0.810225430	0.828594734	0.808046977	0.772844142	0.720533048
0.033146030	0.045792992	0.028514965	0.035478432	0.034918741	0.026698351	0.028897227
0.034058019	0.066871537	0.035938091	0.081923540	0.061098748	0.048430465	0.034077380
0.025704116	0.031766845	0.034247271	0.034467203	0.031808506	0.028070602	0.026131891
0.417992190	0.480520763	0.473411989	0.510753736	0.560472257	0.547155259	0.431223022
0.094764274	0.076172844	0.069179227	0.080738857	0.084153045	0.084382970	0.072842810
0.828686096	0.922705277	0.909971032	0.921906885	0.929160676	0.929840327	0.880611745
0.038614035	0.078265455	0.049599327	0.050288926	0.069636897	0.051900167	0.042740477
0.856602743	0.874225279	0.866467503	0.849029017	0.916928897	0.906702423	0.840523145
0.056752249	0.058320547	0.076431004	0.073750007	0.084124197	0.065666331	0.055907493
0.028201874	0.028250304	0.043291677	0.046784564	0.035050442	0.044096832	0.028408639
0.021636511	0.028569577	0.037439538	0.033471134	0.038165381	0.030067006	0.025051144
0.173331538	0.123184935	0.134254492	0.192108093	0.106890042	0.143836991	0.196183137
0.415042248	0.456874265	0.430937871	0.463529958	0.481065417	0.473558249	0.414471895
0.041163442	0.050950625	0.045389062	0.051508950	0.081509221	0.046733570	0.041990290
0.230942774	0.188870189	0.163025783	0.192600407	0.234357437	0.251577754	0.174727230
0.280694669	0.392163937	0.260534684	0.269236085	0.319105140	0.339375536	0.258607377
0.038399260	0.028052204	0.039456846	0.049717324	0.042087833	0.043713784	0.033594307
0.397646329	0.406154957	0.393171039	0.379593390	0.436505960	0.427661406	0.366454798
0.119878198	0.124204753	0.123371291	0.134595423	0.164495850	0.154122726	0.112662421
0.060634810	0.050560103	0.041777126	0.056843751	0.080159800	0.054108552	0.046941367
0.697082553	0.712463917	0.694744127	0.777574432	0.802740002	0.771151353	0.687733337
0.866303104	0.891435708	0.903029155	0.870914919	0.888288066	0.910274929	0.866956445

0.337885628	0.392212115	0.295839895	0.541472125	0.576841223	0.486593205	0.316073049
0.466793541	0.467488105	0.443888944	0.503856812	0.572052508	0.539658232	0.438478660
0.040292655	0.056744075	0.047086751	0.054932570	0.058899835	0.061241861	0.042266615
0.081436701	0.086134299	0.066222330	0.104797124	0.140312114	0.081301591	0.068013571
0.229279483	0.270122892	0.212458562	0.262147943	0.284282809	0.285242240	0.214040499
0.958010391	0.950743281	0.962990348	0.964541428	0.971901295	0.967531445	0.971211823
0.738901827	0.907544449	0.893598892	0.864406594	0.903621396	0.839020102	0.788547294
0.566336096	0.651897857	0.641677218	0.650978456	0.682021305	0.734679387	0.586935705
0.050582676	0.145226054	0.110785228	0.116301595	0.121629202	0.113227637	0.072586739
0.048315355	0.051908916	0.056664040	0.061728162	0.050634924	0.068061805	0.049642216
0.120841129	0.159000450	0.144241613	0.130485976	0.167661753	0.153700340	0.119002043
0.028422344	0.046990580	0.030139413	0.033323356	0.047164340	0.032358037	0.027862695
0.035780029	0.055652150	0.051379728	0.044013000	0.081149441	0.064159824	0.040977189
0.147615711	0.128489156	0.135382920	0.173464591	0.165621976	0.137793124	0.121615486
0.024466447	0.049421558	0.034812136	0.031629882	0.037909588	0.040891028	0.027740157
0.047654391	0.067118747	0.044892619	0.061498040	0.081665423	0.102369311	0.050109151
0.019495587	0.020243901	0.021920938	0.021150310	0.028505390	0.038973790	0.019924533
0.096265567	0.118255081	0.089436739	0.095184146	0.129048577	0.098957190	0.086321255
0.241517936	0.154875969	0.063999336	0.055900930	0.071685084	0.072659078	0.147192792
0.432889959	0.453718379	0.414856823	0.401710210	0.468503984	0.429290199	0.393579955
0.041010978	0.039983976	0.040973903	0.061186321	0.050021174	0.068882526	0.039020342
0.339975685	0.378709478	0.377629670	0.390307586	0.409146005	0.436755128	0.350254551
0.037354726	0.024806042	0.022706709	0.030495191	0.035979065	0.027692906	0.026485151
0.070369086	0.076742258	0.056417866	0.067807559	0.083285952	0.063306680	0.058095681
0.589835487	0.730556358	0.645561285	0.699627595	0.746791441	0.619778653	0.618206234
0.102149863	0.139353931	0.085000736	0.099885301	0.101497897	0.110196935	0.084600238
0.565236952	0.647815388	0.631674286	0.696768179	0.736324440	0.709203437	0.601155980
0.629755916	0.713897453	0.682880017	0.750550460	0.711093120	0.729216995	0.620089801
0.708343062	0.765527487	0.748527187	0.795740193	0.774006723	0.793075806	0.703148206
0.033106340	0.063673859	0.056376606	0.048410438	0.070324786	0.050020389	0.036977815
0.050588548	0.043114919	0.049916457	0.066052318	0.072066558	0.055487433	0.044218831
0.036854931	0.046448823	0.054641844	0.046421205	0.037407105	0.036437434	0.038211810
0.023029685	0.021147393	0.023817964	0.023868454	0.024003447	0.030278326	0.019427316
0.073868574	0.052829569	0.047351286	0.075732263	0.074672925	0.061430211	0.051635720
0.020443492	0.018243843	0.021089767	0.020900792	0.026380957	0.024484673	0.018932005
0.053868467	0.076453361	0.063122828	0.063228332	0.108395713	0.065433539	0.050969172
0.041147308	0.048592106	0.074231311	0.067619466	0.060599690	0.080101154	0.045713924
0.022930363	0.030413727	0.029536014	0.028065472	0.040887822	0.031819291	0.023328684
0.061315029	0.067006972	0.057299227	0.082312999	0.074382194	0.073692264	0.058695732
0.032310355	0.020584097	0.032070320	0.030595330	0.034212191	0.028355092	0.024130172
0.056446593	0.044633630	0.039766519	0.049597720	0.036124909	0.069956669	0.045535375
0.099179062	0.102949218	0.055777144	0.084468904	0.094359310	0.057188421	0.066738229
0.026339766	0.027074875	0.020186305	0.027999684	0.028473025	0.026611167	0.022880244
0.892341155	0.903200132	0.912709087	0.923638841	0.931211610	0.922584791	0.889653146

0.052106812	0.056332529	0.051154414	0.056989223	0.053839585	0.058796500	0.048923871
0.551013300	0.573633595	0.526901897	0.545582348	0.607500526	0.615472777	0.532787890
0.058212173	0.063897659	0.048016159	0.062394671	0.067123676	0.063631586	0.049650926
0.051105051	0.047758916	0.053550123	0.058104450	0.060262426	0.062121073	0.047019322
0.589005881	0.672076399	0.591465062	0.594763403	0.627193334	0.652055009	0.562648223
0.173911477	0.246614329	0.113451350	0.174648634	0.184912179	0.231469785	0.113804478
0.659931937	0.706630951	0.683516930	0.699960617	0.752951042	0.751632015	0.669103059
0.024727084	0.042000600	0.030799508	0.038622320	0.036536335	0.046752703	0.026256566
0.347362232	0.319462629	0.363275701	0.303496704	0.344302222	0.356247144	0.295351361
0.035627495	0.041391537	0.051327890	0.047003857	0.055733883	0.069692161	0.040441534
0.150041257	0.178219397	0.138771144	0.152353289	0.192207670	0.169219113	0.146744944
0.068716297	0.122936141	0.090957820	0.064393938	0.099250449	0.071429207	0.062246085
0.382281550	0.519319624	0.459182573	0.434814930	0.464862060	0.415791637	0.376588882
0.837943804	0.869255504	0.845359113	0.860718330	0.892760634	0.861655857	0.833131408
0.042689529	0.047170275	0.037220204	0.039607792	0.036837104	0.034709716	0.034016709
0.045570530	0.107348082	0.068366471	0.114888799	0.094416921	0.087355578	0.063823312
0.087975931	0.110386764	0.087924560	0.118497231	0.113575727	0.107517239	0.085343570
0.872620472	0.901725204	0.899405088	0.906601201	0.918117239	0.924758985	0.886008322
0.370255291	0.393000425	0.404409316	0.423637294	0.380197029	0.363579620	0.357624396
0.022155824	0.023903992	0.025517447	0.027981124	0.035530526	0.021761146	0.019916608
0.098308838	0.210939044	0.128203240	0.159181054	0.232382087	0.158942107	0.120268114
0.060715341	0.047212498	0.063031145	0.066808007	0.053083110	0.067689873	0.051026465
0.043365961	0.022882210	0.027576633	0.049551276	0.033899335	0.035207786	0.028626225
0.048650249	0.053091351	0.042418478	0.056163011	0.039172989	0.050746355	0.040319659
0.091648508	0.071818931	0.073005875	0.083905385	0.076709475	0.076495640	0.066876419
0.036494309	0.028754988	0.040320694	0.043947086	0.028938392	0.035329959	0.027931629
0.067880289	0.070499627	0.048884208	0.084860841	0.066094374	0.070283661	0.050604943
0.442548371	0.551883180	0.481515732	0.520610012	0.597781005	0.551362613	0.457818235
0.034978576	0.056752100	0.048961838	0.042250354	0.083694017	0.066081843	0.039290665
0.034321054	0.034089255	0.029354932	0.046210822	0.041697864	0.029525342	0.030171126
0.169038957	0.135443170	0.154892241	0.170508897	0.226296976	0.272252245	0.136299224
0.040916608	0.051140443	0.042615175	0.075295289	0.055890108	0.068284525	0.045618357
0.573943820	0.575839032	0.641921000	0.540650159	0.603279823	0.607782706	0.550082048
0.036451942	0.047322491	0.030509065	0.034388298	0.039305449	0.028172028	0.028047170
0.038906704	0.039730311	0.031270264	0.041244648	0.046192082	0.038662543	0.031271633
0.808842161	0.585065582	0.763550756	0.535554458	0.610571390	0.718206695	0.755712358
0.402867039	0.460032866	0.416996494	0.451516101	0.498151873	0.485287527	0.404217130
0.024261288	0.037791091	0.027915436	0.033110955	0.037367097	0.036384467	0.026462384
0.036185067	0.025877865	0.025999473	0.037524369	0.037448992	0.034827131	0.027893168
0.666064212	0.777686737	0.758525714	0.830233152	0.795964937	0.770585569	0.724088437
0.081988396	0.084301119	0.051460975	0.066171463	0.060626973	0.079316052	0.060252306
0.053524271	0.076098298	0.052780600	0.057735933	0.071402230	0.054895713	0.043524013
0.183015013	0.161549758	0.098642470	0.091656265	0.110238240	0.127888892	0.083218292
0.029467173	0.031206828	0.032610037	0.040083303	0.039298914	0.042473217	0.029128686

0.034540887	0.041370972	0.031759488	0.043024250	0.058773118	0.044344957	0.034345614
0.423107659	0.635819284	0.543350965	0.584828296	0.519883386	0.479699739	0.449094487
0.021248997	0.020441472	0.027987960	0.029612280	0.026101393	0.023782324	0.022672075
0.032855544	0.041515631	0.046778669	0.048585264	0.043002670	0.045036602	0.035278777
0.187080083	0.187502053	0.155845264	0.174383954	0.219384391	0.215783110	0.170100359
0.056098578	0.024871247	0.028597596	0.024372314	0.161111513	0.023382038	0.122287406
0.185570569	0.245834128	0.173519897	0.218619680	0.304187075	0.187560135	0.176966492
0.030185992	0.023771261	0.023413759	0.023580071	0.025405770	0.017614392	0.018933424
0.710715694	0.860632825	0.779222094	0.905588206	0.880079133	0.837535864	0.759748260
0.032388768	0.025862181	0.026496956	0.023710190	0.033555016	0.029285656	0.022805447
0.217221697	0.175027169	0.179036624	0.192838687	0.256615486	0.227131573	0.177108270
0.018660872	0.026647405	0.021393418	0.022637448	0.025661498	0.025955635	0.021114749
0.028662994	0.038320139	0.030156050	0.036011323	0.036211179	0.036984153	0.027193482
0.048320426	0.061134448	0.041611008	0.045481694	0.062720149	0.044983968	0.041140650
0.084101250	0.283295286	0.118219495	0.088311174	0.289266414	0.207458090	0.115094601
0.022126105	0.031481653	0.023983740	0.029055729	0.034641512	0.021863943	0.022645193
0.864400575	0.887961195	0.850824995	0.890233804	0.914085254	0.913833925	0.858547373
0.068704549	0.127065187	0.126965040	0.126987692	0.137661944	0.138633177	0.084201386
0.157875500	0.132094456	0.134666725	0.224835256	0.133712134	0.150258835	0.120631698
0.918664429	0.935462832	0.933054339	0.946507520	0.948800932	0.964001858	0.951723524
0.023084674	0.024580009	0.021204211	0.037717782	0.024450722	0.027174424	0.022335805
0.063530639	0.166115472	0.132402770	0.124976978	0.123830540	0.090561136	0.077032272
0.041960605	0.069412924	0.052180693	0.053059141	0.062968244	0.067575030	0.045150952
0.066748841	0.064234852	0.061136907	0.061004313	0.071016205	0.062461692	0.054987628
0.036911629	0.051104500	0.039968702	0.030824654	0.044834515	0.044596871	0.033740124
0.028593972	0.040552439	0.036382794	0.052062301	0.046535282	0.047445172	0.034825717
0.036718368	0.036180427	0.055610221	0.044020187	0.052565445	0.048030592	0.035309796
0.306524383	0.333471017	0.268907911	0.272828604	0.379014683	0.329881878	0.260827058
0.586886583	0.661335081	0.651254585	0.670835640	0.678000048	0.652453416	0.588399069
0.030740778	0.031607110	0.025999349	0.025388217	0.024841077	0.020118308	0.022617206
0.065545351	0.134086795	0.104820532	0.121972370	0.131365296	0.119277183	0.077499883
0.031624190	0.033631821	0.033377835	0.030066075	0.031111103	0.038667604	0.029850794
0.928693514	0.881414469	0.934747226	0.927895255	0.923818217	0.947574661	0.947593929
0.052354532	0.057852233	0.044047211	0.058680381	0.063806274	0.054155868	0.043098131
0.045276583	0.045633160	0.038013664	0.045337303	0.045639268	0.060007601	0.038472689
0.033310898	0.051943349	0.049425032	0.056823587	0.051503278	0.054794184	0.039570601
0.148780973	0.145474235	0.137796589	0.151476266	0.157825606	0.165355288	0.130503993
0.720148921	0.756356809	0.789081050	0.815462224	0.822088874	0.803129418	0.750819553
0.789574773	0.844247732	0.825096386	0.848074874	0.849725264	0.875332233	0.802382588
0.063112213	0.115726293	0.059079635	0.088571783	0.160682798	0.104368243	0.069589617
0.626654152	0.818702504	0.693691436	0.742573227	0.803098123	0.840398772	0.694074897
0.339802937	0.318237576	0.333136559	0.364963577	0.400505756	0.368478415	0.309158646
0.054331841	0.048050921	0.055806725	0.060437757	0.055592122	0.060241585	0.047862080
0.087186376	0.182404223	0.136325029	0.186714865	0.200937299	0.143050591	0.105312012

0.034277610	0.076733605	0.062061414	0.051485048	0.066127438	0.071566288	0.042821022
0.041311018	0.066107699	0.067039531	0.062515148	0.065580624	0.061545451	0.046091418
0.052931006	0.054622540	0.058937890	0.071327626	0.061235246	0.058003004	0.051675222
0.273037267	0.298132597	0.281350871	0.317551503	0.349867572	0.344783054	0.276830989
0.034983283	0.045039387	0.042866658	0.034716593	0.051426946	0.045724760	0.035096441
0.027064568	0.018167929	0.023035496	0.022853265	0.027735104	0.030973459	0.021763948
0.504186675	0.583903214	0.514174279	0.591678149	0.596169374	0.567872176	0.491595726
0.755806665	0.814438279	0.820787045	0.799741312	0.830088735	0.783809467	0.745433999
0.078497574	0.068786518	0.055022532	0.045013212	0.066199262	0.047874643	0.050482631
0.047572836	0.075459273	0.044550056	0.051214908	0.145660456	0.059754704	0.044080230
0.813924497	0.826984628	0.799155758	0.835110909	0.905362985	0.831874461	0.797710986
0.046683305	0.066520708	0.041367256	0.058230505	0.057101906	0.056667152	0.039743213
0.053757304	0.040487873	0.044447709	0.060387972	0.055597785	0.044343245	0.040609374
0.058077565	0.082762903	0.071248412	0.063438751	0.067403757	0.063551046	0.057379275
0.672748828	0.638822654	0.633275967	0.795254868	0.742897491	0.766899860	0.767389069
0.065960328	0.145688713	0.107316653	0.101547453	0.122471757	0.115784340	0.087012435
0.494635106	0.597686053	0.576334467	0.549030685	0.644610426	0.611974676	0.528929207
0.099215034	0.150296655	0.136494159	0.121239369	0.146909981	0.111472119	0.099812266
0.096393142	0.127117524	0.101135770	0.102351448	0.096097237	0.144028893	0.086509576
0.340046240	0.360311775	0.397176943	0.304357447	0.537250503	0.446389758	0.328302296
0.032324466	0.027789311	0.028830828	0.025809330	0.024349541	0.047479175	0.024612274
0.064331562	0.059019215	0.063013175	0.084804294	0.061871352	0.071439016	0.052840709
0.633878935	0.669273230	0.603137021	0.632517963	0.720926458	0.708769786	0.604080895
0.888311635	0.897201497	0.892004165	0.894153013	0.914573780	0.915225140	0.875530552
0.692206364	0.762753051	0.739952809	0.765896896	0.782290633	0.729210164	0.691282044
0.156182022	0.099872883	0.118494152	0.107898106	0.131277642	0.145307819	0.105384619
0.420143331	0.537171096	0.483553989	0.567915287	0.572105683	0.526972788	0.459184355
0.359492238	0.476863899	0.421679093	0.540821817	0.579552100	0.458874512	0.391007710
0.034390219	0.021349602	0.050899019	0.039666107	0.036591567	0.036840726	0.029807165
0.448788292	0.558757001	0.493132842	0.529743769	0.590690125	0.577398435	0.501773273
0.035673331	0.025131653	0.030983465	0.040142090	0.032865096	0.037235387	0.026316820
0.062409072	0.078832245	0.069864952	0.104490795	0.087615987	0.106808382	0.068545338
0.064860047	0.058866907	0.051001753	0.054733640	0.067214713	0.052063863	0.046603598
0.037733015	0.056517195	0.034820959	0.042307403	0.062269645	0.057211898	0.039114765
0.078446187	0.073961626	0.059026525	0.086662667	0.095693177	0.086397023	0.068663138
0.185996221	0.196054034	0.174297918	0.231921370	0.243950050	0.231404324	0.175148291
0.030384595	0.032898039	0.031206630	0.036371417	0.044630756	0.036114704	0.030012818
0.054714195	0.045532851	0.060962007	0.067216352	0.051619473	0.071498867	0.050330668
0.578421287	0.638500774	0.595420488	0.666727291	0.659827370	0.680165438	0.571723572
0.215803065	0.347262893	0.291226503	0.266184170	0.366781849	0.304281706	0.238892521
0.030183698	0.037754293	0.043162693	0.040258119	0.049273751	0.042581145	0.034201018
0.349823453	0.424915352	0.341223406	0.352260113	0.413276617	0.385577926	0.315970070
0.034742633	0.044826513	0.031261674	0.040605506	0.054748981	0.032909653	0.029021169
0.023934368	0.036383242	0.038106523	0.033114294	0.053146045	0.038834628	0.029398102

0.046831638	0.036768320	0.033960275	0.040869523	0.041571206	0.041035777	0.033135509
0.037333584	0.053371911	0.040055934	0.055272827	0.108883398	0.061146434	0.039224317
0.529925686	0.546763928	0.590919772	0.490627991	0.601713657	0.549058158	0.502971885
0.842205174	0.828968015	0.845057963	0.860712827	0.872659647	0.869177630	0.834639219
0.451112454	0.522485084	0.484437152	0.533021486	0.551838368	0.538170644	0.441956620
0.020274041	0.061610766	0.037704596	0.055219780	0.070187072	0.039722327	0.034089082
0.053423511	0.040220549	0.047220843	0.059821413	0.057429892	0.051447966	0.044461164
0.539511051	0.511915766	0.572917743	0.639689754	0.661724599	0.591315155	0.526227214
0.050514519	0.099624510	0.063260819	0.070682693	0.092703631	0.052611758	0.050535216
0.108764707	0.253654572	0.123127881	0.155165262	0.229521860	0.172118537	0.112602064
0.431308230	0.527246201	0.575865412	0.455231421	0.602384711	0.515744451	0.433458822
0.059855018	0.085375507	0.071114628	0.072635744	0.068709685	0.059244295	0.058500108
0.083086909	0.074071320	0.079372864	0.075765814	0.070044440	0.093426485	0.070775702
0.033836045	0.051306326	0.050883426	0.043336336	0.049874734	0.048887541	0.038533293
0.066220842	0.100358057	0.086371484	0.108798504	0.115324475	0.109411485	0.078443813
0.035469613	0.026892292	0.034381398	0.032882021	0.031691033	0.041116354	0.028912891
0.265923509	0.260736889	0.233836949	0.260125863	0.313078964	0.334845672	0.243603616
0.341144715	0.406204338	0.246577386	0.335929079	0.367363510	0.394166936	0.285648976
0.132726833	0.146968903	0.125293695	0.151791014	0.184802806	0.190261716	0.128968570
0.024340618	0.042983092	0.024971280	0.031691231	0.031632144	0.026601041	0.022656165
0.046324163	0.050409302	0.046009320	0.057264615	0.057501440	0.054904356	0.046349811
0.828815413	0.888229438	0.869307113	0.936674395	0.903425483	0.893856751	0.855786519
0.028667002	0.049825110	0.032633504	0.029919107	0.037541848	0.026476682	0.027641279
0.866784382	0.910646169	0.896950265	0.910805342	0.875636698	0.881222045	0.867205404
0.173452494	0.165674502	0.139094280	0.171600562	0.181647933	0.208257543	0.148897180
0.884502695	0.897529650	0.864050182	0.922830588	0.933133489	0.923265816	0.868066594
0.075827585	0.045855791	0.144225429	0.074300460	0.051128975	0.061914829	0.050398396
0.543305906	0.651881002	0.603635780	0.652157856	0.648807671	0.632560261	0.578759655
0.020778681	0.016612273	0.018196144	0.023255324	0.018231314	0.020562401	0.017006749
0.023340386	0.033334494	0.021857856	0.024855898	0.027805851	0.021147158	0.020036471
0.736681357	0.800801180	0.762483703	0.845980964	0.841169326	0.740397259	0.722044363
0.826938795	0.829633066	0.836037683	0.845834569	0.865457990	0.891308719	0.828284058
0.043495054	0.035016639	0.052838361	0.040534495	0.054672121	0.035861105	0.035760100
0.880073513	0.872217268	0.876500681	0.902808425	0.901324486	0.908531628	0.868399398
0.049495166	0.057879704	0.044018362	0.041724247	0.046791204	0.039535057	0.039897270
0.030968981	0.069595448	0.047067134	0.034968436	0.040564627	0.037397272	0.029992424
0.026766706	0.028926118	0.036443434	0.045974606	0.035510093	0.036002204	0.026101653
0.029313227	0.025505316	0.029128123	0.027580309	0.035545760	0.029969311	0.022533287
0.042272918	0.054261114	0.037581897	0.058776325	0.059282201	0.035240291	0.034008548
0.036571809	0.047469115	0.052654748	0.058243437	0.070747386	0.060838052	0.042810930
0.042132146	0.051065419	0.045170417	0.047377744	0.050212456	0.062731906	0.041087346
0.479787824	0.566505327	0.500403361	0.519740482	0.608358567	0.573066748	0.483192710
0.803821471	0.793000575	0.854267873	0.872175564	0.876725144	0.867787011	0.867504216
0.728301271	0.729034172	0.760291932	0.736557832	0.804260321	0.819538056	0.720058614

0.243343132	0.26299438	0.23752233	0.22064485	0.29873042	0.28801008	0.21022727
0.19090485	0.22254829	0.16196040	0.19354905	0.18339134	0.21085342	0.12601817
0.18402349	0.18173495	0.11005502	0.13675007	0.13450039	0.12402961	0.10613187
0.65961537	0.84439848	0.79293644	0.80106457	0.848808650	0.75276871	0.71857406
0.75174243	0.82431936	0.75545748	0.78582446	0.82866066	0.84090139	0.75453731
0.28168830	0.22657159	0.25832576	0.31835853	0.38325955	0.30568194	0.22185162
0.08996075	0.09961537	0.07506511	0.06255314	0.096407982	0.09376061	0.06853675
0.61054386	0.59808349	0.67054156	0.65105814	0.66802544	0.63574591	0.60302417
0.46083380	0.61811369	0.50739186	0.57093588	0.645059931	0.57782889	0.50154958
0.20284635	0.27088219	0.24026878	0.26156127	0.279692941	0.25933233	0.22131813
0.53099791	0.47482108	0.40517574	0.47366250	0.53051072	0.31734328	0.31715459
0.04764118	0.04974093	0.03917014	0.049919805	0.05407475	0.04710276	0.04079864
0.94338464	0.97233423	0.96494768	0.96353014	0.97461894	0.97166450	0.95424872
0.32728395	0.326921078	0.26603784	0.31861014	0.34539702	0.34481761	0.27689441
0.02945513	0.02528133	0.02593106	0.03556321	0.03360392	0.02495204	0.02473393
0.46445811	0.70065485	0.62992753	0.56810681	0.59448185	0.58154695	0.50961550
0.03081021	0.04818551	0.07132485	0.07677716	0.051977051	0.04988436	0.11382011
0.03491452	0.04687375	0.05428347	0.039007867	0.04352797	0.04741482	0.03433581
0.05329339	0.15970030	0.10304382	0.124847489	0.11283392	0.08792838	0.06802386
0.64318165	0.73224590	0.77013905	0.70877144	0.793887908	0.74606507	0.64278892
0.87714417	0.87183909	0.89164696	0.88711096	0.89914664	0.90080627	0.91344152
0.07781719	0.07012742	0.06846269	0.079080798	0.11718853	0.08175037	0.06812539
0.03355449	0.04130287	0.043227642	0.03681040	0.04165228	0.04381316	0.03232988
0.04802127	0.05496363	0.04784025	0.06171326	0.05959931	0.06213657	0.04913437
0.17507568	0.19759156	0.13724794	0.12805740	0.18770596	0.20004859	0.14570278
0.59062358	0.72986581	0.73074356	0.726327257	0.74572532	0.69746279	0.65709188
0.22401324	0.306996500	0.22663738	0.18784662	0.25442844	0.24082000	0.19854543
0.57048944	0.66022806	0.60848350	0.59629764	0.68015620	0.66005419	0.58319254
0.09282226	0.09425403	0.070279565	0.10653500	0.10035269	0.07320553	0.07995762
0.04743609	0.04834036	0.05486583	0.04931412	0.04536049	0.05430068	0.04533063
0.05419892	0.036581960	0.03167101	0.038145272	0.05013684	0.04306709	0.03645149
0.29187988	0.26717824	0.28448201	0.25234218	0.29338918	0.30773936	0.25439579
0.78440852	0.87222576	0.88078338	0.86585525	0.89925311	0.83368896	0.81271359
0.12565604	0.102567053	0.10886623	0.10471427	0.11763566	0.11706033	0.09889797
0.63536851	0.78062770	0.71515094	0.76128858	0.749185820	0.72803531	0.66626464
0.01822986	0.02635762	0.02317981	0.03300275	0.02893492	0.02809775	0.02239756
0.06801212	0.09348938	0.09145504	0.09611162	0.08823256	0.09691765	0.07471740
0.05276003	0.06323797	0.05709238	0.05265874	0.07700209	0.06645331	0.05015217
0.53654634	0.58626102	0.62730074	0.58536635	0.62659692	0.63394748	0.54716845
0.02967596	0.02534296	0.02218504	0.03897928	0.02969156	0.02385940	0.02257762
0.05248713	0.06462222	0.041294231	0.09139614	0.13443356	0.13900292	0.05998708
0.10662074	0.22740131	0.17570853	0.15983211	0.25088055	0.12652788	0.11680858
0.64704311	0.71611361	0.67843816	0.70005688	0.65399406	0.66629808	0.62313947
0.07868181	0.07366868	0.06825621	0.06645505	0.07392855	0.08905622	0.06513164

0.658689217	0.754919875	0.701641456	0.739923778	0.724528409	0.670946275	0.644771626
0.429765110	0.629699749	0.478874317	0.627574394	0.634041315	0.577477460	0.467974320
0.692716120	0.811374916	0.742823095	0.820876380	0.855750662	0.816102976	0.735458444
0.045182213	0.034902879	0.039448848	0.052108544	0.057477517	0.049386679	0.039449326
0.042684044	0.048126657	0.040933358	0.035264123	0.044383216	0.029552098	0.033805602
0.661971835	0.779005274	0.748213266	0.765441513	0.833863542	0.743293195	0.695498034
0.125314608	0.248915503	0.154064169	0.296925507	0.146571518	0.158002223	0.132242757
0.312467842	0.370621812	0.283036701	0.407082958	0.364534641	0.339197568	0.308037958
0.178147698	0.108687974	0.244349197	0.103826031	0.095579688	0.169972248	0.216095715
0.060555386	0.055686003	0.059653681	0.075820163	0.086492628	0.089926071	0.053843433
0.050223089	0.036309457	0.028443672	0.048774063	0.049294675	0.046966971	0.035757930
0.024981630	0.023830705	0.030622575	0.030489337	0.027624112	0.026418857	0.023471385
0.078057178	0.124730620	0.110708970	0.110769833	0.091868500	0.098380346	0.077467417
0.022978356	0.041814248	0.040241070	0.032456538	0.042819767	0.035043406	0.030048674
0.128189845	0.131107466	0.121697134	0.146024516	0.157109738	0.117338973	0.115578842
0.218458964	0.270374450	0.127832780	0.157624852	0.184287973	0.236335339	0.112274375
0.032797145	0.024099644	0.027731851	0.026637932	0.038233477	0.031224185	0.026738739
0.026468959	0.022773805	0.062770978	0.026663125	0.032460725	0.047212110	0.024910889
0.824201808	0.838725462	0.830182582	0.847351398	0.878004430	0.863914718	0.807786509
0.901608720	0.906337023	0.911924111	0.915148801	0.926859081	0.927093231	0.890318029
0.085633492	0.058483128	0.056637975	0.065583445	0.064480805	0.059466591	0.055176185
0.162782256	0.172518704	0.207674147	0.220958482	0.339770499	0.250054532	0.177658790
0.055891867	0.026867644	0.037995734	0.044130216	0.055459467	0.053349840	0.037164115
0.042227246	0.056314055	0.059610951	0.071729972	0.060122680	0.054537777	0.041634888
0.054144080	0.078125643	0.070216210	0.075565724	0.089711770	0.077671793	0.062668021
0.045257807	0.082079717	0.064363308	0.069413109	0.084676183	0.076942368	0.052903819
0.018649130	0.027698091	0.020197055	0.025728776	0.031229238	0.022923873	0.020732971
0.074525197	0.067749516	0.053958974	0.058252490	0.062644802	0.085031074	0.056684838
0.031690619	0.039396153	0.034898057	0.035521547	0.036028022	0.027196406	0.029261739
0.034574586	0.037292380	0.043801413	0.038865448	0.030877795	0.033920430	0.028647434
0.655616852	0.783597001	0.701303465	0.690441309	0.750107189	0.717358364	0.672322298
0.035787184	0.035511593	0.050397073	0.044066371	0.043683966	0.045837963	0.037090900
0.031880328	0.031281779	0.032608258	0.031303664	0.033172968	0.030895933	0.029157615
0.073843271	0.155448940	0.078812482	0.076199965	0.124454798	0.116934345	0.082063088
0.023755679	0.028376109	0.021847140	0.022299845	0.021156841	0.018288883	0.020447315
0.028575234	0.032665783	0.032671475	0.038926990	0.034178957	0.040175458	0.029690331
0.122386489	0.112203441	0.100899105	0.122581159	0.116142304	0.137349087	0.102625105
0.280311936	0.302449036	0.270199230	0.300386114	0.361295444	0.340783831	0.280352896
0.027364463	0.039615174	0.044774604	0.041889159	0.049306701	0.043216693	0.035180364
0.077903290	0.117516042	0.088938588	0.108176773	0.132831452	0.116732976	0.094840099
0.051183734	0.042962278	0.030652103	0.062548537	0.042479413	0.047699351	0.036378625
0.847801881	0.847197534	0.864630381	0.879962658	0.887540132	0.902833352	0.832714903
0.054835559	0.040957699	0.049361727	0.054650832	0.048276916	0.043328058	0.040520246
0.264761287	0.150475450	0.437167452	0.162406546	0.237067828	0.475339980	0.407293896

0.813286150	0.884300190	0.809444616	0.854335069	0.910226171	0.875813725	0.819499511
0.798819358	0.864399389	0.814562239	0.843405449	0.871207339	0.867075375	0.814402684
0.803284577	0.892264428	0.880768607	0.822375585	0.891648919	0.862593459	0.802440757
0.068488426	0.061038037	0.057521055	0.066586948	0.069855167	0.065757170	0.057787392
0.030291927	0.041383571	0.039556313	0.055256907	0.055409699	0.042074736	0.034514412
0.350715634	0.387908541	0.344124641	0.346964388	0.422447679	0.407670396	0.349058724
0.699911114	0.709486302	0.725161800	0.712068779	0.719687067	0.794690296	0.679769388
0.080120694	0.078409873	0.058773377	0.082389527	0.085940235	0.084277008	0.070967434
0.029526217	0.020109375	0.021780736	0.023527687	0.025704511	0.028484542	0.022600660
0.722762679	0.819740945	0.784715549	0.786287701	0.856637212	0.828943269	0.746820775
0.555845235	0.687766455	0.610525851	0.612402190	0.686825380	0.671990254	0.574706513
0.033125514	0.030331786	0.038202616	0.036508091	0.043998361	0.051621407	0.032072829
0.043817567	0.143421575	0.070334562	0.161851741	0.162496203	0.071326888	0.058663524
0.052103054	0.057797774	0.054003420	0.058976774	0.065792395	0.068077611	0.054231988
0.831859339	0.860457061	0.862010250	0.883334163	0.887129125	0.878971956	0.816970326
0.024951412	0.020549588	0.020677540	0.023225464	0.033410233	0.023800861	0.020706132
0.091458194	0.055207696	0.096576718	0.118011258	0.094886376	0.104399058	0.121023755
0.871485610	0.914449235	0.902120164	0.888338407	0.897003400	0.906899956	0.866790150
0.060924677	0.117637460	0.078123265	0.062452578	0.073174664	0.045725254	0.052265482
0.776725431	0.807952355	0.843905960	0.792821575	0.826739940	0.849325354	0.786718902
0.027205708	0.031802460	0.026728495	0.031587304	0.046171296	0.027743320	0.025597093
0.048922942	0.080426226	0.094825099	0.108900375	0.086986723	0.093732610	0.059870344
0.072935979	0.181613553	0.129140109	0.154874656	0.224304531	0.147032735	0.096621229
0.043238693	0.036306972	0.033046169	0.040933099	0.057924075	0.045809240	0.035405144
0.022364336	0.027006804	0.020944444	0.021562672	0.028134914	0.025696979	0.020468623
0.862943566	0.854568419	0.887118309	0.884553320	0.903973641	0.872035267	0.857212174
0.763476169	0.846455123	0.831100345	0.743263164	0.836263220	0.835593152	0.778391337
0.436693419	0.497378834	0.440594145	0.407192324	0.526444666	0.490771305	0.424133043
0.093836730	0.087471159	0.066655763	0.075487841	0.072583608	0.081556175	0.067567585
0.049421496	0.047893678	0.041475916	0.054707128	0.051877836	0.060424236	0.043187406
0.198008450	0.151393807	0.159843802	0.156894658	0.226097384	0.185008288	0.141033801
0.030334693	0.027473929	0.027095972	0.024091520	0.027469950	0.032038438	0.022758475
0.451808485	0.595547692	0.598139696	0.667195706	0.500602775	0.557556852	0.477317591
0.090862891	0.097821379	0.094017322	0.104390263	0.094892435	0.110889477	0.085966722
0.707021008	0.536098682	0.777133737	0.834885192	0.710593406	0.778596244	0.804887317
0.409241419	0.443118877	0.310200034	0.508507080	0.485075468	0.463927798	0.499146213
0.037077334	0.029082329	0.029676023	0.040597615	0.041000376	0.055163362	0.034795114
0.050411655	0.088015230	0.062729492	0.046008445	0.063986170	0.041061726	0.044680556
0.203992198	0.253582581	0.175842923	0.280003326	0.266291323	0.255957807	0.203197060
0.519918309	0.748991257	0.549789400	0.670416047	0.698450279	0.661306824	0.566565828
0.025233160	0.033169430	0.026793473	0.034459072	0.050512430	0.032943133	0.024306078
0.046988151	0.046968899	0.039170560	0.049468448	0.040987332	0.048556475	0.038449890
0.049356439	0.050404211	0.045453055	0.050336500	0.044577307	0.046513672	0.038866695
0.030684475	0.039231157	0.034186008	0.035766663	0.043567651	0.037273417	0.030588311

0.023470977	0.031071155	0.022965320	0.027871853	0.031434961	0.034745516	0.025020333
0.048279274	0.036251554	0.038908871	0.036868716	0.040945650	0.035288905	0.033859565
0.016739094	0.021560423	0.019390253	0.024009649	0.027500172	0.019381036	0.017773749
0.067738515	0.116365114	0.090301405	0.110125789	0.303226942	0.083840978	0.083392900
0.716275858	0.833102675	0.825734394	0.900313530	0.857525725	0.843357971	0.724639532
0.036455999	0.053318156	0.046642466	0.030346120	0.040424400	0.032242833	0.033398799
0.506084463	0.646439086	0.513047969	0.532686869	0.604721870	0.573763130	0.491352409
0.089986465	0.102874240	0.090849306	0.114418400	0.098360570	0.096870796	0.082029491
0.083044839	0.080950034	0.072396121	0.076414124	0.090738209	0.083024286	0.070167680
0.482333977	0.553824309	0.445394527	0.506915551	0.549532934	0.506630630	0.450989258
0.543250342	0.628207725	0.613656264	0.632681606	0.638304676	0.660351320	0.562595987
0.829028946	0.903051919	0.899270675	0.897548257	0.927136736	0.904683805	0.854531735
0.330954502	0.382265953	0.282700835	0.279016030	0.348252035	0.349887289	0.272906762
0.434292366	0.540942047	0.478128209	0.650768003	0.524897451	0.506377732	0.438639080
0.577866459	0.621739415	0.646613453	0.706129754	0.617170817	0.693882485	0.584424601
0.022569855	0.028201001	0.029985192	0.029233666	0.024997901	0.031168590	0.022998145
0.026146196	0.036233742	0.031988790	0.029668671	0.040896654	0.032365337	0.028886833
0.047966165	0.077606727	0.060360579	0.063650992	0.091621473	0.070067039	0.055975753
0.055691006	0.070921107	0.054438969	0.071688194	0.064269104	0.069522603	0.055158405
0.736775744	0.784083097	0.786722672	0.800773153	0.804716205	0.861069115	0.747525141
0.026357304	0.024029023	0.022813127	0.030819984	0.028445545	0.031221241	0.024047471
0.800530479	0.866764971	0.852942641	0.887082672	0.899257403	0.896653145	0.837395043
0.041774122	0.032442734	0.027137114	0.046330529	0.046346817	0.046229023	0.030592039
0.025357132	0.039004436	0.023810723	0.032190119	0.032647971	0.027384729	0.025352259
0.043188212	0.036049580	0.032782191	0.053433368	0.048420052	0.061654350	0.037430574
0.048804781	0.098779707	0.086982032	0.180590845	0.109810490	0.076271010	0.064830950
0.020260427	0.034206268	0.033230598	0.037749834	0.041235298	0.036828768	0.026065258
0.089890416	0.058700997	0.078410197	0.077607248	0.062772787	0.078650585	0.060505538
0.048291943	0.072134611	0.057325218	0.067611955	0.057182323	0.063063967	0.043185149
0.466346122	0.592604562	0.573412373	0.626373712	0.596706708	0.546926867	0.499242737
0.871021577	0.880151124	0.883026067	0.910267967	0.887908705	0.912577343	0.873264350
0.534844868	0.664090477	0.626671818	0.585794959	0.679896856	0.626176988	0.541545562
0.447593191	0.486802275	0.470226356	0.535898879	0.542839961	0.562070621	0.451795583
0.757651520	0.821846671	0.744243677	0.885272117	0.873812389	0.815405191	0.746786306
0.856211390	0.911988434	0.884921429	0.883647764	0.909843227	0.874270328	0.839451099
0.910975160	0.923792629	0.935319024	0.932386996	0.941887389	0.929504218	0.904674099
0.443716036	0.504766692	0.446514635	0.656358036	0.500128768	0.496924758	0.415555140
0.042729197	0.127418017	0.065253910	0.074797508	0.105343871	0.069552312	0.055475642
0.335798766	0.569610194	0.393952616	0.583217792	0.518861635	0.429881757	0.384390766
0.380423277	0.493920562	0.391094824	0.396723158	0.490726770	0.486447658	0.383030950
0.280393908	0.427940592	0.299969637	0.386106195	0.398903611	0.373806231	0.285670174
0.126394460	0.145109428	0.126829336	0.159712827	0.170059405	0.147254882	0.128471693
0.815494290	0.851595234	0.855312558	0.878756879	0.882447205	0.898681679	0.832793618
0.775250183	0.883098235	0.844748086	0.856087526	0.894481591	0.863901913	0.816329375

0.026672554	0.016541661	0.025783990	0.020278938	0.021327620	0.023288057	0.019277898
0.033140250	0.048013202	0.046344546	0.051637285	0.059425833	0.050544465	0.040534218
0.253086683	0.298495012	0.253086075	0.272636745	0.313439062	0.274624324	0.228451217
0.072327971	0.182002053	0.102020905	0.087374295	0.139630219	0.109981891	0.075739444
0.047813465	0.047542374	0.043319406	0.059532409	0.055552128	0.059221291	0.046866328
0.323138391	0.361035179	0.346679117	0.380641795	0.372222227	0.386982653	0.317396357
0.062330542	0.128838374	0.067278102	0.073668875	0.071102205	0.063890753	0.058956800
0.024356378	0.028809808	0.022164622	0.026237537	0.031145299	0.029460411	0.022233382
0.036227818	0.044292234	0.024828070	0.049489142	0.045222685	0.039766673	0.031834765
0.050654877	0.098092170	0.102213855	0.080491088	0.137269548	0.069355518	0.058784479
0.020237322	0.016782731	0.019851634	0.015965286	0.026479133	0.019629216	0.017165196
0.043072383	0.027083970	0.032988858	0.039338955	0.051162463	0.059296697	0.033142229
0.078997377	0.105875868	0.094500570	0.083370103	0.125942111	0.097473457	0.076297679
0.108269012	0.082327979	0.097411231	0.113597282	0.120537361	0.126534404	0.092295891
0.324633565	0.027709204	0.347752210	0.039646420	0.355472601	0.082011334	0.069165349
0.031397251	0.035840282	0.036621689	0.039145748	0.034424485	0.042132703	0.029920999
0.046002413	0.045860323	0.052341614	0.046042872	0.050058271	0.063497183	0.045235367
0.671296925	0.723623128	0.693374765	0.764466451	0.782950237	0.772985540	0.666524118
0.036044447	0.032657951	0.051041742	0.038022699	0.032422466	0.042028271	0.029959358
0.022921289	0.020061681	0.022064394	0.027136529	0.035511590	0.033790279	0.021891935
0.080540504	0.068064533	0.070550034	0.072978083	0.063412069	0.065106169	0.063817814
0.672609878	0.828450490	0.838510092	0.645463708	0.870314264	0.813054068	0.817624284
0.934369032	0.925326366	0.937224572	0.952867232	0.944357027	0.952359663	0.951790431
0.620252022	0.744484446	0.678407379	0.752416081	0.760145735	0.692620189	0.650586184
0.084191461	0.065721373	0.050499798	0.051442034	0.074241394	0.084220836	0.055437360
0.025303072	0.036237108	0.028865276	0.039597075	0.055402749	0.040738032	0.029540281
0.849021066	0.916928004	0.930281935	0.903644897	0.943872562	0.925440329	0.832669453
0.053206084	0.045229538	0.039158926	0.047692386	0.059589713	0.057044124	0.045546386
0.399888715	0.684265851	0.346237868	0.515121996	0.464613692	0.574750184	0.544573040
0.654909386	0.695685537	0.654574273	0.760823178	0.743569927	0.690271251	0.646651321
0.826995889	0.854849826	0.817024306	0.852994662	0.908062945	0.870745725	0.828272995
0.023353075	0.026903417	0.019804874	0.024071737	0.031965782	0.057099829	0.021058533
0.719462251	0.750342569	0.743306768	0.774323749	0.806106144	0.776506200	0.706349374
0.024239559	0.026035250	0.022226291	0.029145196	0.029971540	0.026280844	0.022574520
0.658429183	0.715693218	0.762926401	0.707016342	0.778666798	0.672882712	0.663493696
0.033087821	0.020905242	0.026626292	0.029492439	0.038425344	0.039170972	0.024776465
0.807901357	0.894362513	0.854714455	0.865471403	0.897858233	0.887905908	0.830123486
0.053331224	0.043132617	0.037447065	0.044276349	0.045202859	0.046247995	0.039969936
0.071428240	0.075626051	0.061134397	0.100331217	0.083696913	0.077694901	0.066920347
0.752049895	0.863008064	0.807986373	0.837685726	0.808912105	0.814271923	0.766727213
0.748808257	0.777206863	0.809048176	0.799105189	0.834445460	0.849706451	0.751799092
0.787984894	0.811574290	0.796360773	0.807472728	0.838118141	0.842035568	0.780982967
0.084138522	0.360961435	0.287548923	0.064869519	0.363378069	0.269807415	0.339641186
0.054390531	0.069666550	0.058258992	0.044107489	0.051213555	0.049183408	0.039497271

0.028889352	0.035866509	0.027377616	0.030460852	0.032428336	0.030847163	0.027278256
0.069867033	0.059435292	0.129959616	0.068973986	0.097027784	0.071091865	0.060208192
0.112888434	0.123984642	0.107792709	0.132469415	0.137579421	0.137539602	0.111060282
0.024192845	0.041903122	0.024632515	0.029234977	0.047879591	0.044080984	0.027203580
0.099643217	0.101951715	0.085447930	0.104730970	0.119105909	0.108502536	0.091097712
0.069560825	0.094171737	0.091504199	0.080708320	0.127516380	0.145065626	0.074208900
0.034571817	0.041324920	0.047727838	0.051608150	0.038025924	0.048064997	0.037738458
0.137042639	0.128161417	0.155664628	0.160154734	0.170033371	0.160899650	0.127991733
0.038136606	0.093361713	0.050898912	0.037364755	0.043092687	0.047437838	0.037714922
0.413944915	0.469091598	0.515730533	0.495410195	0.545163009	0.498929926	0.443694026
0.028886903	0.031762145	0.023955570	0.026747344	0.039327949	0.031271714	0.025467532
0.260444683	0.292140170	0.194461851	0.218950933	0.249949337	0.297793457	0.206891337
0.731034854	0.748086330	0.771530766	0.726794822	0.824922706	0.806648478	0.700992700
0.065270300	0.124210510	0.078556388	0.073934557	0.086695888	0.094592810	0.069501817
0.390165389	0.596707989	0.452899167	0.625732904	0.659984501	0.537351900	0.465474679
0.063578757	0.081280903	0.057484362	0.090688229	0.083851112	0.074270848	0.060382042
0.030945072	0.032825988	0.030476444	0.038804770	0.037812155	0.033324844	0.028982775
0.057229342	0.059848242	0.062417098	0.084767028	0.071035129	0.070815718	0.057461670
0.165757158	0.155696575	0.145360692	0.160130515	0.146879312	0.158072905	0.138896049
0.024030786	0.039531980	0.035521969	0.037669373	0.041853188	0.031453327	0.026914289
0.837041317	0.910080288	0.870126419	0.897009781	0.935553896	0.892124874	0.864660586
0.019894639	0.020914319	0.020983430	0.022282061	0.033176588	0.028205638	0.019686377
0.034092115	0.037022673	0.031772783	0.031725932	0.038400833	0.037111992	0.031517907
0.367752212	0.512839172	0.395411444	0.578379388	0.580237826	0.496514254	0.404068936
0.044078984	0.046847028	0.043058626	0.044919363	0.041733571	0.043249703	0.035177910
0.443417353	0.376336288	0.387160104	0.437919438	0.473702185	0.474369949	0.395535031
0.061182898	0.043204651	0.041647887	0.048999825	0.046888144	0.064134272	0.045152407
0.189253035	0.164411432	0.147663634	0.178651022	0.175827079	0.209342960	0.154186709
0.887861506	0.912675549	0.929887247	0.924398813	0.945800094	0.948290243	0.899560586
0.026169234	0.021638457	0.024850469	0.026332762	0.022935594	0.026579208	0.021037159
0.743660813	0.778633025	0.734781629	0.750900882	0.776260954	0.791274594	0.718786737
0.067743886	0.077335134	0.034126644	0.036672201	0.047496010	0.051935075	0.039868794
0.038999798	0.033776400	0.046992451	0.038094281	0.037022713	0.035908678	0.031814757
0.538479692	0.659541444	0.502473880	0.571462314	0.611199439	0.600886122	0.511491595
0.940266409	0.880664696	0.923449062	0.913017448	0.911558903	0.931391400	0.940539739
0.035934372	0.074509984	0.037153604	0.073015458	0.153716241	0.090669867	0.044516892
0.075482551	0.216254200	0.169821955	0.217088839	0.214076457	0.212078252	0.140692312
0.041578679	0.045420691	0.038298018	0.044904751	0.037514721	0.046089325	0.035466395
0.050230538	0.092650032	0.068275551	0.085825802	0.097285805	0.083663313	0.058314327
0.329770111	0.353904512	0.247836822	0.325715027	0.366793070	0.374050858	0.293165852
0.015978631	0.018297443	0.016107787	0.016986762	0.019839937	0.022473414	0.016108390
0.483689013	0.478870765	0.398445232	0.349067464	0.511734385	0.364857529	0.355417634
0.291967914	0.294680405	0.262380475	0.289046872	0.304889804	0.322103408	0.268879146
0.194328235	0.159835182	0.185866611	0.211416926	0.225278275	0.215348697	0.167026286

0.548317200	0.649081283	0.643290696	0.664383308	0.663696844	0.677295911	0.560490852
0.392753205	0.520745883	0.454423125	0.542747882	0.530225153	0.508806566	0.426468357
0.327652749	0.357979112	0.301649806	0.377907412	0.413316237	0.329583620	0.304810029
0.070549810	0.089094422	0.069180828	0.072997090	0.090575206	0.072689206	0.064310873
0.252544432	0.265308697	0.215765677	0.209837022	0.247583744	0.286237879	0.203188186
0.475787844	0.505539177	0.467741076	0.521713609	0.646940769	0.576639735	0.481158822
0.059392153	0.070338209	0.054469507	0.052021090	0.068587476	0.041135965	0.047307416
0.091717838	0.077392592	0.078953379	0.071299841	0.120396940	0.117961768	0.080847387
0.033519686	0.039890628	0.027538732	0.031535538	0.039993184	0.035579527	0.027588835
0.055138261	0.068781905	0.071304406	0.060817291	0.059869728	0.046830178	0.053655285
0.888632871	0.905352245	0.908191691	0.895816442	0.908238707	0.905611924	0.877261126
0.866128356	0.895823351	0.899940575	0.911381354	0.909922921	0.911691140	0.872007765
0.038034380	0.032737705	0.032374482	0.090097404	0.045641815	0.048004281	0.034500860
0.723135895	0.828648524	0.807137437	0.769606344	0.806841924	0.766973907	0.738527752
0.036174796	0.047994385	0.034359396	0.031587358	0.042354575	0.028488113	0.030370965
0.021750813	0.031264398	0.031301089	0.025149647	0.033557931	0.035798075	0.022372533
0.066173877	0.040243195	0.076986210	0.065137920	0.076462311	0.097803864	0.059219389
0.060016211	0.123815245	0.071267304	0.074059536	0.093697007	0.081157418	0.063367447
0.236624449	0.409275353	0.354852554	0.371450396	0.406129829	0.327746106	0.272423660
0.154480422	0.088073787	0.087129231	0.080183352	0.086577646	0.106315201	0.080362843
0.926112521	0.937166291	0.934800868	0.942939891	0.953355190	0.951029632	0.928553897
0.873468059	0.868877221	0.887547103	0.877937953	0.900082837	0.901812204	0.854762468
0.538699987	0.634533859	0.594784227	0.605259035	0.655922926	0.602258203	0.557247983
0.036126432	0.039194773	0.033302133	0.030974119	0.040383386	0.038001253	0.029251645
0.594774986	0.718809730	0.665883361	0.703355509	0.677013901	0.726127706	0.609847157
0.029621492	0.021211148	0.029136460	0.043500595	0.027843909	0.026751136	0.026001199
0.146200343	0.181470539	0.131613242	0.175945996	0.239612647	0.112116050	0.119854893
0.020236953	0.021292094	0.023373585	0.020316774	0.020837800	0.017376425	0.017864064
0.035822370	0.044166945	0.042444083	0.044732163	0.051754368	0.045786246	0.036712241
0.038173606	0.070337376	0.050575577	0.079787201	0.057441741	0.064567749	0.048321073
0.845556082	0.867062072	0.889873483	0.919263866	0.907972827	0.899915644	0.845293889
0.930453353	0.819289667	0.904157348	0.901877415	0.886891547	0.928077165	0.931389669
0.056202803	0.054630470	0.053918428	0.063446767	0.054547953	0.057832122	0.048555189
0.030807027	0.025137334	0.031899968	0.027395318	0.036980082	0.031999898	0.028288449
0.037346303	0.028677919	0.039615484	0.040771624	0.034967355	0.037269367	0.033410815
0.771862871	0.849344555	0.814446530	0.844058288	0.874408268	0.837398287	0.791242457
0.880748153	0.926348565	0.915984762	0.924306693	0.947364696	0.947451234	0.904717113
0.457349979	0.531117680	0.473018002	0.536237648	0.580285878	0.526572724	0.459784163
0.398488741	0.435893380	0.426078545	0.431869302	0.394565538	0.386741916	0.462017594
0.035451585	0.042560054	0.048193903	0.059315759	0.060246309	0.050072985	0.041883011
0.282410910	0.265680414	0.232945328	0.275752459	0.270443454	0.298624877	0.224532628
0.038491507	0.039719594	0.044740639	0.038142757	0.038000947	0.052185390	0.033736051
0.536978208	0.547843729	0.538767335	0.553041465	0.592162413	0.598900870	0.521521349
0.028951174	0.038247506	0.029883342	0.034462721	0.037316121	0.032061310	0.028506352

0.693822988	0.728452596	0.672115278	0.718589140	0.715332453	0.782542284	0.665288267
0.880372697	0.852706461	0.857406725	0.868729292	0.892717378	0.880901731	0.852349442
0.036869139	0.063930081	0.040286183	0.051220071	0.068073063	0.055821810	0.038580872
0.500894153	0.589378379	0.478791216	0.571319567	0.613129778	0.573272980	0.485390167
0.710748594	0.850335608	0.788308095	0.813296781	0.859701439	0.796828828	0.752966932
0.046997820	0.036677998	0.046527705	0.054616386	0.046286111	0.045437950	0.039410190
0.068825261	0.088592663	0.076246196	0.071593938	0.173594455	0.084238801	0.076258823
0.703574228	0.816761807	0.761185575	0.793438018	0.822187778	0.823128388	0.751496665
0.020560951	0.019929915	0.022199856	0.021060976	0.019289541	0.014731895	0.016891504
0.840328987	0.875784164	0.873536373	0.898606313	0.928440370	0.855325787	0.838583248
0.093963355	0.074538806	0.065948482	0.061016679	0.076169359	0.073572433	0.065154637
0.756131273	0.761344541	0.763257252	0.838378192	0.836702918	0.803912064	0.761720954
0.732868171	0.844567652	0.773063488	0.833205905	0.854721324	0.814850181	0.757664050
0.575387431	0.796843461	0.627435027	0.839181446	0.823595680	0.780177432	0.630324059
0.022168503	0.427097574	0.373790317	0.022473654	0.477219233	0.213601351	0.728012327
0.043795262	0.044402364	0.038482707	0.037853202	0.038905323	0.037993601	0.036374286
0.131792376	0.129963930	0.102508253	0.114772879	0.125694399	0.135570982	0.105988483
0.843637851	0.904772272	0.891443004	0.899291131	0.927740277	0.918432113	0.861053985
0.053786122	0.059025913	0.062422339	0.068824497	0.070603801	0.062336190	0.057303857
0.014964294	0.022749258	0.021848269	0.022429048	0.024655251	0.022823167	0.019134954
0.841167255	0.911688701	0.873116915	0.916613444	0.928026832	0.908575089	0.872971764
0.962747086	0.965032243	0.971858252	0.974325767	0.966609414	0.976619571	0.974833979
0.045258074	0.060848230	0.058019043	0.052256871	0.049271315	0.056402449	0.043771382
0.854547066	0.897751472	0.873540715	0.899239024	0.914659207	0.913615721	0.877046363
0.018287799	0.029419802	0.019974418	0.027675866	0.032503760	0.023976342	0.021768847
0.104697059	0.131817697	0.097938536	0.125391030	0.104865303	0.131069162	0.095623395
0.039889880	0.058470316	0.059429966	0.054174847	0.057395915	0.060101918	0.046543837
0.934626155	0.966411940	0.964653804	0.958505460	0.946504539	0.960403354	0.963355146
0.768838332	0.677469258	0.824428682	0.786408504	0.831143805	0.806796559	0.837599375
0.360496964	0.434037701	0.355707268	0.369079209	0.440070135	0.450298089	0.361102944
0.058632338	0.085324188	0.064791539	0.064800834	0.065104596	0.065663805	0.056715848
0.618231113	0.751575365	0.652783708	0.720548653	0.785415827	0.775266666	0.665725550
0.589379671	0.681010718	0.669719112	0.699551719	0.734789579	0.696435450	0.626469022
0.426945696	0.503789828	0.495227305	0.524200673	0.644231537	0.555320310	0.456493358
0.041823606	0.041135914	0.025592004	0.039860261	0.044261574	0.054995313	0.032612700
0.069965410	0.103770366	0.069667220	0.101240813	0.112651533	0.086976227	0.074571630
0.037793981	0.044048334	0.060604800	0.040236009	0.047823917	0.063007418	0.065915395
0.025995043	0.037036297	0.035747442	0.037484117	0.042604308	0.030714375	0.027539603
0.424017769	0.497695002	0.403862974	0.529914628	0.558152265	0.571005453	0.438857287
0.889888740	0.872071117	0.900350469	0.918065720	0.913489758	0.923430588	0.880757048
0.043320959	0.056254456	0.039731728	0.063733740	0.061131402	0.041515354	0.042447261
0.599504674	0.695088570	0.634061822	0.699872131	0.712854124	0.684481488	0.627383037
0.027673830	0.037614068	0.032848982	0.054336468	0.038057642	0.046813782	0.032155993
0.329912042	0.234219706	0.276142236	0.323813700	0.432508749	0.371871491	0.272520332

0.196090103	0.236656430	0.114637945	0.126548439	0.146479990	0.218229712	0.135415100
0.025444693	0.027277520	0.021257022	0.023707076	0.031096539	0.035512169	0.023463414
0.863952029	0.882343975	0.872584079	0.898054257	0.895494755	0.900914719	0.869453971
0.819422112	0.844500742	0.879161205	0.862364937	0.884056327	0.850494940	0.831954748
0.134937658	0.218416957	0.148278469	0.182486257	0.184261205	0.128343003	0.133777704
0.058648496	0.053028780	0.055151060	0.061080341	0.062570185	0.063596305	0.051832361
0.031442605	0.077410862	0.035368014	0.044654415	0.062032832	0.042005449	0.032549800
0.031524437	0.034394916	0.032398557	0.037004288	0.039631228	0.030125120	0.028018712
0.807306242	0.849827139	0.846169957	0.891790928	0.885483789	0.887806231	0.835918439
0.395595631	0.477907035	0.398736793	0.385186685	0.495943521	0.441473028	0.386514556
0.028820752	0.055070094	0.039104703	0.046793752	0.062065800	0.050235960	0.035854745
0.446009381	0.522324299	0.318201007	0.346708795	0.476139367	0.475648903	0.341886061
0.047800892	0.065887101	0.052613695	0.070525757	0.072724996	0.068672394	0.057781214
0.176593757	0.154696076	0.122721721	0.141551116	0.153684225	0.158584720	0.132207515
0.552900399	0.675899521	0.601760061	0.628811040	0.651258972	0.682109608	0.581025147
0.696967352	0.649603003	0.743851434	0.716629936	0.761635224	0.705838437	0.640399612
0.339084734	0.334780026	0.361137153	0.318037889	0.374225640	0.386863962	0.291246591
0.114197330	0.118234419	0.112984178	0.121999397	0.135467402	0.142571512	0.118365467
0.064240552	0.073797590	0.088424616	0.131215251	0.058459669	0.114405462	0.071760768
0.043467025	0.066608778	0.042486304	0.046076294	0.085154447	0.068439438	0.047414015
0.025350205	0.019814423	0.015380919	0.024107529	0.025248680	0.020291569	0.018914553
0.480282341	0.514250385	0.443569953	0.524493744	0.509401824	0.526380039	0.408221638
0.043714350	0.087880376	0.065740149	0.066232276	0.093833128	0.061580829	0.056898004
0.072820063	0.041612406	0.030399073	0.050516708	0.044029362	0.045677475	0.039965503
0.043522854	0.026864548	0.030900769	0.037855285	0.033562172	0.027509027	0.026576279
0.079137730	0.049546051	0.055968197	0.071761929	0.109480783	0.088862269	0.064755559
0.336229272	0.349999546	0.353743633	0.401628164	0.379740244	0.341566063	0.332108525
0.081382323	0.140087450	0.065310897	0.070832258	0.094434618	0.092651504	0.072824497
0.044733032	0.068349296	0.045132253	0.053930981	0.069465882	0.054224228	0.047234034
0.154884812	0.165303912	0.092165239	0.107120236	0.123688283	0.149992015	0.099899915
0.021784029	0.036891679	0.029643523	0.031645401	0.038200524	0.034534095	0.026846891
0.086275793	0.062861180	0.046402907	0.070092008	0.088636179	0.099089027	0.060664276
0.378596364	0.544118788	0.423653993	0.369932917	0.427781481	0.451237632	0.361365578
0.588899104	0.673929289	0.623804265	0.699995303	0.716040918	0.687287055	0.586238265
0.420097356	0.492407509	0.442265145	0.496762771	0.462873761	0.455915400	0.417876921
0.810671987	0.890631496	0.874760888	0.871000811	0.899869381	0.889740838	0.831320934
0.061919249	0.075630402	0.066664720	0.076383949	0.068888502	0.078444629	0.057851108
0.065814301	0.072401703	0.078894917	0.105178712	0.082094453	0.084908459	0.061578839
0.020921166	0.028276793	0.017408127	0.027334913	0.027332379	0.024222368	0.021055616
0.185813292	0.171457299	0.165575372	0.172443558	0.220994614	0.188439004	0.164344044
0.889976547	0.899566750	0.893031134	0.888059321	0.931070211	0.916204506	0.885092218
0.023294952	0.020629978	0.020327090	0.024878426	0.018329831	0.018125163	0.018505810
0.018940515	0.018424620	0.020126916	0.024277418	0.020424313	0.020771793	0.018771817
0.040361081	0.067780750	0.047936200	0.054005684	0.065104495	0.053883297	0.043874007

0.046975231	0.054619148	0.050169226	0.051438426	0.048103619	0.052811171	0.045306311
0.028246015	0.035038028	0.033087832	0.035651910	0.028909520	0.023057001	0.026189859
0.026715689	0.025706659	0.027822479	0.033287285	0.041916799	0.036198922	0.028262552
0.731767488	0.835792845	0.820381716	0.825575776	0.866512966	0.769377681	0.783148346
0.048732891	0.043146688	0.042546239	0.049443335	0.045641960	0.050509247	0.040351667
0.788327927	0.872144267	0.864019723	0.874715531	0.904227994	0.841927217	0.807574492
0.749108253	0.865677121	0.839275742	0.855437599	0.865608206	0.858969788	0.812194182
0.061765026	0.088442105	0.130437909	0.091765579	0.094059221	0.102742168	0.074062891
0.658989856	0.812497858	0.755118410	0.799331409	0.831397775	0.766137931	0.704076023
0.030830237	0.029900972	0.062341074	0.033758452	0.149816524	0.042030922	0.029172805
0.503814261	0.437139600	0.675144279	0.310951491	0.671568001	0.491970726	0.642917134
0.028342897	0.026910207	0.025631893	0.027754845	0.023377409	0.023841719	0.024511430
0.053455657	0.044175066	0.051783766	0.054480044	0.052475099	0.065596985	0.046100093
0.066421767	0.063549099	0.057012927	0.079028303	0.078125697	0.097678331	0.058095748
0.718719432	0.762140890	0.721295756	0.807020553	0.811005578	0.774011891	0.720843960
0.022987413	0.020852365	0.016770260	0.018086403	0.025570955	0.033296573	0.019291377
0.802447489	0.848000148	0.832504245	0.843674036	0.864040862	0.826282858	0.804411113
0.872914015	0.879891546	0.880018674	0.864668465	0.908614541	0.919977002	0.859584688
0.021787316	0.041205034	0.034462952	0.042644945	0.047068141	0.037977104	0.031948665
0.229216972	0.344394586	0.470327417	0.464531869	0.504194224	0.394007908	0.329564045
0.057466550	0.038867678	0.048165940	0.053327504	0.064150013	0.093277200	0.045658087
0.043839281	0.052599104	0.061667491	0.060925157	0.052904654	0.065906012	0.043773144
0.893402246	0.931375956	0.907304480	0.929960959	0.936153899	0.943336926	0.910238230
0.023215055	0.035388259	0.023272078	0.037141070	0.039931922	0.037972250	0.027707536
0.682562127	0.695090301	0.695434515	0.711623959	0.771696462	0.700689938	0.690702207
0.197444229	0.225841996	0.189582968	0.203880786	0.233316670	0.256171516	0.190791434
0.215505235	0.179524324	0.170203306	0.156435003	0.208484512	0.195518357	0.167356710
0.139104325	0.122618907	0.115942981	0.100647482	0.128470363	0.122073903	0.097386614
0.323716854	0.241283495	0.273990414	0.432575459	0.398636931	0.351755847	0.257826660
0.687700345	0.776891000	0.719673486	0.795069012	0.781976263	0.768314044	0.651900466
0.032708502	0.035793673	0.038758151	0.048663925	0.035935740	0.054132754	0.035006124
0.368995210	0.497930724	0.469724639	0.484120242	0.502460916	0.491302031	0.409248651
0.052228895	0.048268154	0.050727414	0.060762908	0.077428824	0.058274519	0.051494242
0.038497465	0.038528771	0.038021705	0.043792457	0.046056931	0.047478142	0.038432772
0.166686237	0.134491738	0.157026701	0.167051246	0.191178436	0.163682587	0.146008229
0.253691764	0.361026531	0.267731165	0.330924246	0.343681650	0.283723635	0.228965956
0.680720983	0.762075697	0.799550757	0.810678030	0.839502912	0.822660504	0.735731235
0.889328961	0.916462034	0.900965842	0.891085463	0.891818468	0.892057648	0.906301015
0.036324511	0.053189358	0.034893147	0.030865178	0.041749095	0.033164658	0.030116448
0.415888717	0.525429143	0.480661086	0.606304587	0.530858626	0.528091449	0.457467548
0.115058956	0.088556170	0.080301142	0.085653024	0.105563603	0.115937291	0.079222979
0.810897070	0.793102314	0.846354010	0.843353217	0.835275769	0.872400981	0.799630057
0.181541589	0.234742120	0.203958910	0.213141479	0.193707241	0.262291108	0.295966793
0.662614916	0.789820678	0.667772977	0.836186844	0.825905348	0.774801724	0.667774621

0.266554829	0.314056966	0.318277534	0.311972295	0.318617288	0.317632531	0.271057535
0.873511892	0.901141629	0.899351125	0.921934516	0.932671135	0.920209702	0.886714815
0.793205993	0.847247540	0.807094774	0.836429790	0.870574925	0.844166176	0.807232172
0.823056184	0.860082436	0.845719234	0.868441716	0.879236549	0.887539047	0.827651472
0.027526088	0.034217983	0.034274919	0.039342138	0.041983139	0.037479536	0.031402516
0.273044113	0.139216565	0.159126989	0.252255716	0.150720729	0.162240518	0.232373682
0.075960374	0.082490728	0.068444827	0.086239336	0.096107659	0.086445383	0.075586926
0.036893072	0.055489560	0.040298397	0.045975815	0.052992775	0.038179592	0.035936881
0.870281134	0.900676375	0.914342606	0.927374084	0.936105961	0.933978150	0.894630369
0.296790132	0.416239815	0.335769647	0.278010292	0.346861775	0.334027682	0.298412532
0.055477452	0.054820899	0.060931240	0.062941556	0.050386017	0.054387978	0.051545548
0.178684285	0.198750760	0.209268570	0.324726445	0.236274691	0.197448022	0.170308681
0.020521385	0.021212585	0.020357790	0.023249310	0.027117555	0.032618220	0.021444400
0.665841962	0.757932526	0.736518397	0.759445947	0.734068786	0.690693075	0.667356775
0.222635003	0.186878310	0.154092651	0.149925496	0.219386211	0.225927323	0.153730360
0.614442650	0.726546493	0.707481699	0.757293529	0.755164483	0.698706527	0.662255962
0.033200801	0.041819421	0.028217550	0.031227657	0.025897779	0.042365642	0.029655919
0.191483134	0.202981211	0.236917714	0.275282874	0.280812592	0.200225929	0.181596703
0.079096426	0.092461996	0.072975452	0.082874541	0.100625872	0.105231418	0.068129760
0.028231899	0.024899068	0.038259388	0.022498828	0.058146595	0.030676103	0.026377785
0.967041149	0.965497684	0.967760801	0.966663334	0.973702851	0.972838522	0.972455677
0.153336333	0.173873724	0.143947688	0.204744990	0.206760613	0.187103524	0.153999128
0.513144547	0.554673193	0.568911826	0.609469364	0.629709838	0.584754389	0.531620926
0.746297031	0.805811181	0.676510804	0.822298980	0.838526399	0.804198751	0.749440787
0.225373228	0.252947051	0.158935559	0.147028937	0.138275226	0.206097885	0.143782554
0.275895117	0.230081018	0.258716838	0.279479390	0.293541532	0.328506317	0.238594180
0.054072764	0.062720282	0.061565781	0.080956713	0.058326416	0.074404241	0.056853853
0.030039619	0.022057101	0.027407711	0.023790788	0.069977014	0.031101315	0.025590640
0.079729223	0.159627640	0.176691436	0.142328537	0.193553728	0.185973816	0.102908213
0.882042454	0.913788625	0.903892352	0.894111231	0.912197719	0.941678073	0.881069274
0.051438218	0.056483674	0.047522909	0.063027747	0.066382460	0.068627195	0.051419034
0.046472788	0.073404674	0.054147147	0.072453507	0.075046392	0.084498944	0.052909725
0.664010653	0.757755658	0.769709492	0.735503705	0.787219603	0.799161991	0.711997317
0.072785600	0.075987568	0.068589169	0.085364661	0.076714325	0.083710105	0.070479029
0.438485794	0.584993149	0.614639280	0.649913743	0.591922368	0.579854443	0.505647054
0.592223419	0.455863248	0.533454793	0.421096278	0.491959431	0.509587001	0.453729581
0.893603090	0.867950193	0.869710207	0.869855267	0.814391888	0.899634085	0.890347265
0.132981958	0.144920515	0.149128335	0.218676499	0.229314377	0.184883327	0.141141376
0.660054103	0.675618526	0.700822257	0.682443409	0.658577551	0.599425665	0.605638596
0.169877094	0.162543106	0.155039827	0.197045011	0.215552633	0.168530540	0.151500137
0.336670687	0.368255711	0.373800208	0.417506439	0.435862333	0.445144315	0.365002216
0.585286928	0.606315079	0.588500039	0.644104938	0.611331937	0.617073648	0.634424504
0.798866350	0.770573572	0.795188731	0.790216282	0.822928361	0.800822104	0.769671258
0.281342811	0.320475483	0.302047471	0.279400195	0.336226640	0.318314831	0.276032369

0.025552876	0.019754097	0.020832831	0.018625010	0.022863757	0.024096227	0.019598364
0.057058497	0.079878380	0.097575118	0.135898755	0.085083991	0.141296189	0.068822357
0.107589610	0.199342225	0.159168351	0.159976086	0.170799380	0.106659653	0.110501287
0.563016292	0.720083948	0.694887239	0.631894396	0.674965011	0.646559094	0.600776052
0.463640432	0.603657274	0.439386785	0.561748454	0.580780656	0.517734007	0.461613102
0.244223800	0.220498783	0.258132672	0.237361410	0.197307044	0.236438399	0.207867726
0.829545313	0.786096222	0.830021390	0.772768095	0.839999309	0.823889883	0.856478252
0.375225179	0.533015712	0.372903836	0.586979311	0.582498679	0.401175337	0.365496336
0.052101512	0.052513207	0.060549858	0.049328217	0.062315307	0.047843188	0.044863036
0.750774838	0.837895260	0.805557648	0.824517596	0.882389013	0.842614408	0.783111877
0.826550633	0.812823216	0.813629541	0.815261984	0.814945736	0.863415647	0.801400055
0.212269709	0.211335428	0.208941832	0.325280319	0.283949351	0.261327328	0.206851320
0.703829782	0.745329280	0.748277317	0.789078166	0.812221312	0.778551135	0.670865778
0.861545870	0.882310802	0.923693648	0.845195593	0.913473073	0.805690812	0.799797715
0.034315569	0.034311881	0.027934145	0.029213198	0.037314629	0.047079639	0.029810436
0.555095327	0.565948568	0.572822339	0.618636891	0.677306212	0.759010884	0.534329740
0.038545721	0.054723557	0.043996032	0.053884982	0.077821965	0.038191934	0.039522530
0.069162139	0.076392285	0.071125448	0.081230589	0.058097862	0.076902779	0.063522262
0.026681776	0.029562620	0.030939671	0.043636984	0.034548016	0.025578204	0.025251339
0.631542986	0.691671643	0.627282169	0.771742596	0.734811139	0.698144468	0.617184208
0.952874141	0.919633895	0.944705763	0.931912416	0.912978750	0.944560827	0.952449378
0.108053056	0.130825860	0.078084534	0.092289783	0.123808720	0.081916957	0.082316128
0.513357298	0.710764917	0.637686844	0.655657820	0.646745391	0.593831089	0.585745605
0.198363515	0.202362609	0.154953726	0.179714843	0.197499645	0.245394112	0.166558710
0.449464314	0.536587893	0.507393265	0.454999711	0.512677740	0.518537685	0.458353258
0.023648402	0.030835649	0.029665650	0.027667842	0.032538900	0.035501151	0.025544835
0.848547846	0.860461821	0.815174260	0.922285866	0.927401454	0.916862663	0.823265513
0.039726880	0.045998516	0.033874923	0.031661781	0.041661933	0.029003226	0.032025110
0.658228785	0.599300125	0.606233211	0.609012745	0.655237118	0.714720873	0.586415148
0.062671491	0.060647766	0.061399220	0.060029799	0.086081533	0.064559716	0.050405903
0.306980774	0.360646964	0.309038243	0.415322807	0.329140387	0.346690776	0.299194702
0.260088563	0.249603648	0.363450735	0.289004925	0.302720758	0.331486698	0.370909913
0.040530713	0.061661715	0.045195772	0.059435925	0.066253473	0.057709082	0.044384344
0.023502992	0.037086843	0.037444939	0.044694261	0.042137000	0.031828778	0.028287866
0.307686939	0.318404711	0.295600157	0.330346020	0.356429903	0.304549851	0.285932494
0.022144048	0.027727983	0.019369562	0.022869733	0.022454184	0.021240786	0.019312785
0.707500221	0.745557313	0.728754221	0.758218132	0.794885346	0.781140764	0.722520868
0.029430316	0.033959327	0.031289686	0.031574837	0.033648066	0.031502168	0.027101468
0.595846781	0.682846616	0.681909884	0.780962618	0.800147901	0.727157616	0.673330721
0.193789392	0.200963007	0.155060698	0.165592310	0.124246709	0.195342050	0.228079618
0.034851960	0.041522831	0.037664656	0.031807759	0.034619357	0.032006896	0.030072662
0.030241495	0.033254204	0.029960337	0.024593312	0.034096137	0.022861695	0.024150911
0.072374529	0.070758041	0.079172313	0.077999610	0.084428021	0.092417090	0.073049544
0.657083091	0.840483829	0.816177133	0.852814756	0.756286149	0.755839903	0.727328196

0.454605593	0.535096063	0.483234232	0.555504729	0.542504007	0.506051435	0.434585746
0.843037368	0.891634091	0.884619162	0.868895631	0.909114673	0.900472775	0.861731405
0.436674651	0.544808319	0.541940636	0.581451536	0.599506858	0.540987501	0.486565682
0.459375619	0.557245671	0.529970515	0.564115923	0.597654669	0.556577077	0.490063015
0.040352710	0.037855921	0.050099775	0.045974731	0.089106287	0.069368229	0.084136431
0.561812092	0.551453722	0.526596231	0.553062629	0.589213669	0.603911420	0.515135987
0.503134215	0.521514548	0.591179230	0.608244507	0.672069968	0.653042776	0.530397433
0.739033671	0.882996906	0.825878465	0.870282256	0.867487714	0.834389807	0.776765104
0.335270413	0.396614260	0.372541611	0.422052644	0.441822921	0.434947375	0.370722354
0.024566389	0.041371196	0.027042827	0.031516754	0.034622343	0.032272385	0.027555188
0.088865201	0.050840647	0.080088908	0.061282385	0.086642233	0.109561537	0.067187787
0.051999953	0.060014119	0.042268497	0.046396647	0.085749404	0.091848277	0.045557148
0.770026216	0.701652411	0.766375438	0.760378538	0.719085682	0.821769509	0.782022262
0.144177307	0.128504744	0.139761205	0.199974465	0.206276554	0.170778526	0.143711573
0.730912913	0.755663819	0.740227071	0.775641635	0.782108060	0.795203054	0.726562913
0.185160853	0.245578592	0.130219765	0.132729600	0.165912321	0.231530747	0.141911203
0.015338550	0.028374376	0.022491416	0.025276963	0.030469286	0.018190692	0.018567138
0.359524367	0.356872676	0.326563166	0.406347960	0.392960702	0.391821190	0.325228267
0.324364666	0.491191346	0.326918366	0.382185975	0.375423623	0.360666570	0.320106858
0.529838921	0.739203396	0.708749406	0.718910071	0.813059594	0.737342524	0.667556631
0.844858288	0.829888697	0.860530773	0.860991029	0.835537098	0.873850609	0.870284870
0.046182210	0.053535275	0.042121150	0.037463941	0.053090656	0.039389599	0.037678476
0.026408499	0.046425755	0.038738631	0.056900596	0.051435250	0.048033727	0.034773988
0.062143594	0.062707129	0.063067195	0.075182971	0.059904263	0.082229363	0.057281593
0.719090015	0.733268285	0.633491128	0.716661005	0.769959672	0.771752940	0.639261723
0.408966287	0.374610669	0.423385031	0.502132719	0.419585666	0.412883437	0.471175316
0.148472654	0.195462645	0.143485318	0.216199003	0.216709177	0.179380087	0.147476336
0.081189655	0.180329274	0.116939925	0.144830609	0.196687504	0.177652360	0.103309818
0.036954293	0.042405878	0.033127722	0.051723401	0.056720791	0.057876173	0.038564602
0.211857496	0.202450653	0.201603601	0.219668385	0.244174018	0.223460088	0.190337511
0.095840898	0.070154504	0.078503823	0.087868552	0.081705851	0.084135611	0.073263440
0.464655319	0.516008406	0.482069185	0.430764793	0.574126522	0.528968960	0.462584569
0.040716247	0.063534688	0.040496438	0.035752833	0.041700429	0.033939576	0.034183285
0.438690553	0.510095156	0.462378756	0.511362344	0.555760682	0.534398414	0.457943007
0.904245049	0.909426364	0.905769575	0.921322258	0.936960444	0.926602178	0.900710639
0.868352579	0.910927641	0.917134272	0.935037297	0.938093029	0.916712742	0.894996365
0.402227622	0.412177180	0.382592959	0.384776074	0.421524630	0.406434671	0.369389137
0.028196999	0.019635922	0.020054382	0.021521247	0.021113191	0.022063107	0.017951338
0.897243328	0.866148902	0.873923541	0.889540349	0.918569237	0.905736854	0.855605182
0.038132034	0.039531575	0.033427816	0.049169578	0.047514038	0.059154015	0.036624545
0.773525491	0.855049431	0.834431882	0.857306519	0.850818355	0.878450998	0.803505368
0.583423441	0.718060155	0.674532701	0.728365200	0.708650151	0.661494060	0.629363483
0.054096075	0.154136909	0.078330100	0.088656006	0.110419099	0.062600826	0.064963596
0.039585036	0.038886016	0.034494360	0.040359439	0.044944783	0.037106716	0.033958954

0.051043674	0.036022435	0.043881084	0.069955777	0.051034466	0.050978363	0.041055525
0.649138426	0.645924662	0.679727473	0.658327194	0.714105504	0.721924181	0.588182424
0.950868107	0.918072029	0.966283921	0.965174629	0.968580893	0.956097375	0.965826861
0.030086546	0.033788031	0.027559099	0.030158870	0.026630135	0.034230325	0.024233250
0.032606388	0.024897435	0.033326088	0.033239948	0.036562095	0.038855114	0.029845023
0.874943692	0.705278433	0.850260033	0.817133174	0.726201128	0.853766119	0.861338394
0.037350277	0.059292870	0.033173757	0.053717759	0.067834875	0.046069263	0.041039305
0.559472772	0.671450860	0.688278452	0.668837747	0.759529923	0.723047329	0.625743165
0.038031150	0.041135660	0.040695340	0.042022691	0.049857359	0.050447546	0.038967429
0.032144783	0.032005006	0.042926384	0.047745817	0.041393292	0.051902320	0.033686674
0.375581444	0.396528978	0.365873672	0.434803526	0.427988510	0.424655633	0.375468413
0.046319943	0.041062470	0.050805580	0.056629130	0.054448003	0.054623543	0.042630549
0.408244892	0.476711887	0.417478351	0.456141387	0.509878180	0.451960153	0.411573341
0.142623312	0.213843775	0.131712634	0.231990772	0.276585565	0.131616849	0.123666741
0.155602516	0.153984801	0.095687131	0.124926544	0.140533888	0.179623682	0.121279028
0.288883751	0.337903858	0.335220797	0.397834054	0.385246398	0.416706217	0.335117047
0.587588647	0.743673564	0.581457549	0.543989189	0.545627904	0.666254229	0.674607246
0.831151580	0.837293900	0.845269914	0.845442307	0.873540154	0.862494606	0.826527803
0.326632231	0.367377179	0.263552470	0.278967904	0.347214804	0.314642857	0.258567150
0.788166189	0.830147384	0.800133362	0.756054424	0.849851340	0.793711158	0.762927276
0.054705725	0.049534562	0.045782563	0.051054342	0.053306789	0.052526567	0.044692681
0.035560256	0.032965313	0.028655574	0.027367809	0.030225701	0.023720924	0.025930342
0.048496107	0.093670232	0.069555228	0.062563915	0.095231392	0.083191836	0.061425517
0.033334183	0.049955982	0.042748435	0.050985591	0.052740675	0.054553807	0.040035566
0.026914547	0.069302800	0.045986039	0.061965674	0.074856400	0.051015434	0.038060245
0.268720557	0.293207338	0.263876578	0.231156826	0.270393561	0.322474355	0.238620320
0.077891281	0.087817759	0.077695080	0.108629721	0.148279373	0.084003206	0.075238262
0.032613405	0.026764484	0.031619848	0.048297395	0.031908097	0.041815375	0.028490629
0.151987339	0.137239305	0.128865106	0.162819753	0.140807476	0.210823362	0.127407850
0.690312851	0.646148407	0.673940437	0.675579711	0.677848865	0.754778620	0.609957882
0.047804275	0.034833569	0.036139007	0.043472825	0.036311401	0.044184497	0.034274128
0.911693769	0.915724492	0.925081687	0.933938607	0.936661129	0.929359283	0.911043965
0.074035310	0.143026698	0.093955276	0.111587918	0.132894015	0.102308915	0.081763487
0.041205529	0.035687322	0.039903030	0.071724747	0.122715823	0.086034627	0.036721310
0.605138935	0.672736211	0.621185614	0.641877285	0.713047229	0.662051107	0.599683807
0.219552432	0.222366964	0.194444833	0.227087885	0.211105873	0.219037900	0.182902694
0.049451830	0.103386270	0.074985986	0.108135319	0.097595858	0.090501515	0.066921187
0.392094078	0.388696245	0.322544133	0.403347429	0.436695534	0.366106650	0.335255492
0.315293211	0.384559938	0.371647211	0.337138195	0.480295443	0.425870338	0.299915331
0.691539021	0.831070429	0.762928026	0.799398779	0.834699835	0.803068255	0.729984284
0.044533883	0.062002962	0.036021862	0.063781438	0.069317984	0.057865222	0.041906728
0.058842892	0.050352865	0.045608466	0.073350331	0.070678758	0.053718275	0.048429072
0.426677848	0.391857327	0.421491507	0.469256600	0.495442976	0.493926190	0.395656237
0.746700459	0.846347370	0.801641737	0.816231055	0.869307968	0.787439076	0.778512181

0.039400217	0.050936828	0.039084398	0.068645473	0.090248505	0.030256905	0.033947366
0.030277724	0.033477699	0.027425343	0.044226987	0.036212165	0.040265377	0.030156051
0.029418120	0.035383102	0.028994272	0.032563156	0.032732101	0.032459552	0.027406553
0.034839463	0.035888496	0.041813734	0.036616388	0.032782195	0.028517844	0.031191667
0.042110844	0.035730737	0.052651102	0.049561355	0.069759609	0.045040155	0.036299994
0.049928006	0.049857664	0.047300402	0.062333290	0.059276305	0.062688043	0.048067340
0.042131601	0.091645526	0.050524355	0.054223213	0.085722974	0.062840076	0.045959482
0.035068778	0.045533087	0.037578067	0.049768509	0.046593874	0.033024795	0.032793734
0.021257852	0.013520131	0.017929439	0.018747924	0.021736697	0.016472847	0.015245583
0.380808620	0.525564528	0.378719981	0.375238291	0.490789460	0.462611342	0.355307458
0.073518901	0.087894004	0.062134232	0.081093870	0.067675808	0.047417077	0.059876323
0.033569879	0.061938555	0.064422387	0.070825341	0.067377399	0.076687208	0.055027204
0.038486314	0.039483478	0.029156262	0.046884072	0.066378611	0.054357884	0.036563977
0.544955089	0.569119316	0.602887516	0.497276026	0.636919237	0.659585181	0.532025112
0.910695009	0.886787121	0.941526526	0.935390577	0.950666684	0.924616690	0.939758216
0.807155988	0.756611189	0.751998651	0.614560221	0.756038056	0.787537121	0.676262894
0.670651704	0.749299115	0.728888474	0.739249189	0.764743638	0.685216816	0.669015764
0.624695562	0.743693840	0.691999394	0.778986907	0.739533167	0.775326713	0.668919336
0.905653516	0.872922393	0.898739773	0.868454377	0.903118484	0.903851199	0.867444833
0.787119026	0.647201356	0.727636936	0.821687817	0.774318307	0.829382311	0.832116210
0.018553791	0.019380915	0.013410286	0.015336187	0.019370619	0.018531099	0.014935244
0.934999928	0.899590306	0.946480033	0.907310247	0.953176241	0.958902346	0.948239231
0.916099369	0.917794895	0.916506161	0.929220830	0.924393075	0.939180957	0.908197940
0.042461121	0.052386797	0.048139815	0.055512135	0.067813086	0.072053462	0.046240976
0.028808230	0.037891921	0.029434741	0.067617851	0.036876899	0.032252675	0.029361333
0.426226761	0.503577969	0.434233588	0.503727676	0.572232381	0.512826714	0.451445872
0.037268506	0.029685307	0.033016132	0.115688939	0.032788097	0.024573194	0.027852052
0.084151382	0.071097237	0.064181252	0.083970588	0.059530612	0.101906337	0.065875920
0.064120856	0.057983574	0.052556099	0.073832244	0.069620020	0.130754421	0.059917722
0.022280929	0.043511481	0.032546851	0.029620760	0.049109587	0.029634913	0.026789314
0.053922060	0.046555717	0.047340468	0.048355436	0.061528838	0.051321094	0.045549436
0.023453959	0.030843988	0.023704391	0.030161798	0.032796721	0.026630366	0.024341013
0.630817177	0.612498860	0.627169652	0.694088929	0.690175539	0.733028052	0.615149427
0.793397552	0.857998929	0.862901618	0.888986594	0.867950010	0.861220681	0.824596341
0.047026287	0.113765549	0.080721788	0.101575681	0.095167417	0.096033471	0.062860315
0.828905171	0.851492059	0.878327800	0.882800314	0.889940703	0.903578493	0.892127151
0.204776123	0.195865664	0.161808595	0.143944975	0.203583071	0.193262342	0.145317114
0.624118354	0.594479213	0.689751784	0.748699419	0.616366872	0.730136414	0.735991686
0.029081837	0.034152446	0.034001254	0.046579316	0.045130628	0.063526452	0.032954432
0.031011018	0.049802563	0.030106780	0.061139102	0.053589481	0.056593445	0.036870574
0.332315981	0.373032092	0.312563203	0.350028913	0.426227700	0.426291717	0.327767295
0.498520177	0.650826242	0.604078883	0.604289160	0.444817441	0.533655140	0.608606490
0.638468795	0.756650781	0.780423334	0.791634189	0.809942396	0.790394145	0.722711633
0.027216045	0.038115099	0.036399096	0.022900877	0.042936399	0.042629523	0.027910130

0.041310386	0.057374495	0.052597697	0.057190403	0.064083648	0.058372205	0.047349584
0.481378099	0.459183667	0.437013609	0.431062366	0.529946494	0.518188734	0.419163803
0.043340192	0.031853041	0.029239301	0.038229582	0.043688730	0.031029611	0.030466976
0.017520170	0.023279391	0.021442414	0.023096375	0.021582925	0.029385956	0.019517801
0.478138734	0.539035092	0.503623464	0.578408631	0.584973100	0.606839162	0.493259196
0.630841847	0.623106057	0.666522431	0.681361604	0.707460664	0.718487046	0.624537370
0.036718339	0.046469325	0.041483074	0.035257926	0.043575239	0.033277234	0.032124979
0.669149196	0.790216269	0.703545984	0.735336398	0.776616092	0.796023708	0.685446197
0.455849756	0.547546988	0.471314804	0.421055851	0.488607440	0.497569312	0.423747192
0.555309309	0.742495887	0.668369366	0.647390218	0.617618265	0.623204502	0.578568948
0.061636529	0.062088436	0.072156579	0.074177273	0.070692855	0.086977555	0.062073524
0.618850016	0.609402653	0.610827471	0.659630310	0.604182515	0.623753989	0.566180615
0.658594179	0.756979943	0.696655048	0.749904956	0.764789727	0.728286395	0.688567258
0.658028150	0.795912435	0.726847036	0.826911794	0.819527330	0.821199808	0.710124809
0.134238213	0.121738787	0.111801331	0.121496884	0.146770402	0.198983194	0.115793079
0.119015272	0.100550279	0.150256364	0.128368421	0.154325972	0.167545303	0.163702570
0.049495345	0.068469071	0.044266283	0.057686600	0.063337086	0.053406916	0.042426251
0.270365575	0.251190651	0.243767970	0.282713594	0.300545076	0.284292013	0.247519521
0.310820112	0.302503658	0.324949756	0.328134159	0.341858795	0.358978898	0.296384557
0.912812496	0.918336081	0.906106706	0.928548384	0.934281532	0.929273588	0.900435710
0.144069630	0.160775622	0.145237811	0.210680263	0.207186603	0.194212187	0.150561253
0.054745876	0.046408408	0.055529821	0.063654148	0.070679127	0.051857431	0.046447766
0.112856714	0.111828333	0.119106092	0.103246685	0.119356840	0.129314427	0.096064226
0.022983118	0.028928642	0.026177526	0.027453470	0.037023541	0.031103589	0.024478938
0.493220253	0.598712876	0.611732270	0.671054964	0.685237638	0.670506025	0.552730705
0.731590547	0.778295904	0.789690450	0.774885703	0.820126681	0.845793278	0.733400088
0.823222339	0.862420558	0.862302338	0.859793786	0.862235493	0.874030053	0.824656634
0.037810649	0.057167619	0.038088066	0.038223946	0.044461973	0.037318457	0.034868212
0.038624001	0.061407302	0.041442508	0.049908797	0.037603703	0.053521207	0.040297134
0.039126203	0.033622261	0.045578053	0.043790448	0.037738976	0.038799424	0.032509059
0.425278131	0.536583756	0.528645607	0.597227409	0.530714715	0.514381192	0.473815055
0.045376086	0.064142771	0.045388217	0.061830422	0.074732342	0.059707830	0.045235886
0.039905565	0.060557227	0.042185416	0.043476014	0.055441626	0.032021746	0.037193985
0.066209567	0.085782348	0.075082622	0.087166092	0.112407380	0.081247427	0.065409278
0.051964923	0.051774410	0.053101347	0.054902997	0.074610469	0.072650115	0.045303247
0.084130152	0.063873343	0.070601267	0.079889502	0.074688174	0.094855304	0.071345145
0.026459555	0.038581593	0.030911674	0.036387012	0.040503978	0.039655524	0.028560619
0.068662676	0.059509743	0.070642973	0.083794104	0.083231532	0.081823923	0.068290607
0.559468031	0.664728952	0.596975204	0.686041801	0.665713566	0.650142733	0.600502200
0.261264558	0.268844226	0.291862120	0.346439293	0.280312203	0.327152701	0.240925352
0.058142749	0.063369090	0.052974853	0.073067540	0.080767105	0.078335946	0.059045463
0.029602504	0.058193799	0.046186290	0.044393871	0.062093919	0.050514078	0.038717660
0.593900896	0.568424024	0.547678847	0.573356778	0.620282204	0.643191575	0.560241715
0.046755277	0.031795352	0.041283908	0.042491620	0.034804606	0.036007807	0.035063013

0.021234873	0.027112724	0.025744509	0.033493496	0.035285770	0.027358413	0.023386378
0.532601296	0.623542835	0.605029029	0.652097141	0.719845018	0.622555361	0.570422237
0.879909934	0.901168743	0.884187910	0.919056042	0.929526140	0.925019095	0.881585687
0.023347917	0.023165785	0.020278341	0.025845849	0.029939978	0.031959410	0.022445436
0.279893386	0.462336557	0.382303476	0.392061099	0.430013125	0.365947590	0.315558735
0.352225082	0.441256447	0.365503868	0.463811053	0.510153089	0.414126847	0.355168111
0.210403039	0.207678018	0.148726553	0.220524488	0.213252487	0.216304770	0.166376991
0.028956133	0.043646353	0.037372921	0.035266099	0.052543266	0.037549491	0.030486889
0.064284642	0.063292820	0.070368117	0.070286912	0.061712373	0.080045056	0.058021286
0.538178241	0.438878928	0.503770489	0.571965469	0.563860012	0.548949587	0.458779420
0.297292153	0.473854790	0.406714905	0.382822418	0.490619989	0.456007486	0.320919312
0.283497662	0.398495420	0.165063714	0.218671120	0.315012981	0.286542950	0.196192582
0.878370931	0.918800561	0.877736054	0.876335376	0.923195413	0.920404792	0.876558417
0.032494086	0.030539049	0.026510185	0.034251098	0.033869266	0.042754254	0.028492512
0.405654025	0.413524475	0.422051647	0.421392888	0.464179271	0.457728995	0.396111887
0.350257337	0.833087783	0.826169634	0.283252595	0.885909226	0.776875530	0.782843329
0.057824272	0.053692959	0.041389473	0.054070461	0.044158822	0.056400062	0.043613733
0.050414620	0.046775363	0.027295006	0.053278191	0.038866219	0.039529962	0.033477496
0.359550512	0.384977970	0.302616834	0.361999163	0.387041192	0.413482501	0.312935797
0.048672938	0.049875894	0.040021326	0.035943639	0.059655034	0.046780145	0.041046681
0.034922420	0.029131063	0.023129607	0.034632171	0.023186990	0.030923654	0.026253502
0.020891508	0.021120600	0.017100140	0.017604420	0.018113766	0.014127226	0.016402353
0.574360277	0.701277101	0.676827474	0.675677551	0.620250592	0.599585336	0.567080277
0.056953150	0.075995415	0.053058080	0.065852955	0.069641826	0.048658124	0.049233741
0.560578132	0.612389063	0.690105460	0.725238009	0.763125719	0.633891174	0.635053429
0.077847496	0.385220228	0.084406765	0.084661015	0.041516040	0.087389473	0.074847942
0.047739411	0.033638212	0.041783974	0.040357050	0.038047258	0.059555499	0.037025085
0.487440296	0.525575469	0.493912870	0.426650588	0.586233703	0.535553632	0.455369862

mean.DA	mean.diff	mean.quot.log2	max.CONT	min.CONT	sd.CONT
0.144950335	-0.037481570092	-0.3995286784	0.125082016	0.086928838	0.011718061
0.047156393	-0.015652342332	-0.4616627206	0.035836828	0.023696639	0.004059744
0.048516799	-0.014075139677	-0.3969381624	0.044419514	0.025323966	0.005632323
0.050306923	-0.016688188598	-0.4673757131	0.042192778	0.024653864	0.005653600
0.161287081	-0.052454828431	-0.5274898855	0.125222288	0.076028929	0.016537006
0.032259062	-0.009552074271	-0.3696618435	0.027865381	0.018285199	0.003654844
0.031464320	-0.008025500825	-0.3103465226	0.028518757	0.018497334	0.003089807
0.058548430	-0.013336070950	-0.3121323793	0.052241577	0.038146925	0.005356260
0.840871435	-0.070074195161	-0.1239932070	0.803978279	0.709398503	0.035595576
0.031364297	-0.009562362177	-0.3792715188	0.026455307	0.017423157	0.002670798
0.033353922	-0.009797052417	-0.3695543732	0.029955970	0.017635097	0.004484138
0.025573751	-0.006729571254	-0.3025328654	0.023459128	0.014060141	0.002690413
0.047266924	-0.014605856158	-0.4247820314	0.042656968	0.027584617	0.004580762
0.864915402	-0.050184248491	-0.0852196203	0.867958485	0.778433141	0.031317660
0.057373095	-0.018403647282	-0.4602906460	0.047943018	0.028972409	0.006530478
0.025824962	-0.006240640257	-0.2761323527	0.023697125	0.016108906	0.002137092
0.273704021	-0.074023528159	-0.4361939476	0.284862853	0.150983462	0.039652915
0.032394202	-0.009715191185	-0.3755026551	0.032597118	0.018820734	0.004166470
0.155217902	-0.036940427794	-0.3651021621	0.141144288	0.097357489	0.015013926
0.177304680	-0.068093441381	-0.6518666941	0.166549395	0.081032238	0.028915287
0.056327487	-0.018911195990	-0.4842240494	0.052959594	0.025075433	0.008991102
0.672072897	-0.112395719200	-0.2597813254	0.628102141	0.451845343	0.056661881
0.839405758	-0.057442772949	-0.1010208856	0.857749906	0.720065216	0.040115700
0.046086472	-0.013198109862	-0.3870666079	0.047789021	0.025741081	0.006275667
0.049159353	-0.014739995948	-0.4134177557	0.043203141	0.024021189	0.006558691
0.050031930	-0.018124760005	-0.5185329507	0.040017686	0.023977375	0.005776082
0.045488699	-0.011443718314	-0.3332163517	0.046678667	0.027389951	0.005774104
0.613402169	-0.063560921826	-0.1551453908	0.593548862	0.493697646	0.033881671
0.641201824	-0.103975070480	-0.2509659759	0.663545311	0.459641394	0.072555223
0.037829004	-0.010167481153	-0.3447944112	0.036303320	0.024021304	0.003805818
0.033853324	-0.007641854328	-0.2762394637	0.029744551	0.020574125	0.002872439
0.849889262	0.0513642392002	0.08370157341	0.924479998	0.874072240	0.016561030
0.056928553	-0.020262384026	-0.5202447838	0.043921766	0.029813467	0.005274422
0.525900571	-0.080900128876	-0.2360974058	0.545196274	0.373145776	0.057992851
0.034229994	-0.011225669580	-0.4223699700	0.030647623	0.015921642	0.004465557
0.257744561	-0.049645375889	-0.2958728874	0.257661824	0.182680347	0.024496127
0.045245840	-0.012770911063	-0.3792542960	0.038173357	0.025979129	0.004109242
0.448406588	-0.088172866525	-0.3081914733	0.411048959	0.323477751	0.032208148
0.023676137	-0.005904469303	-0.2781128388	0.020493173	0.012955149	0.002255267
0.044996163	-0.010943428782	-0.3200993968	0.043711986	0.025456862	0.005891852
0.749389352	-0.070613677214	-0.1408055773	0.731636524	0.621051791	0.033332967
0.495550509	-0.075251984996	-0.2325173457	0.478684606	0.344268175	0.045427557
0.115155394	-0.044610440738	-0.6358543683	0.097302365	0.043366693	0.017958156

0.059393650	0.035430135674	0.595090557	0.141638695	0.061588771	0.024999718
0.022438495	-0.005470715615	-0.266470153	0.018846786	0.013527336	0.001659459
0.066101906	-0.015928845441	-0.338814847	0.059505312	0.042864534	0.005774953
0.287329996	-0.046192482348	-0.243587468	0.266963862	0.208784082	0.023512663
0.062748409	-0.014710593888	-0.325922486	0.059206050	0.039351890	0.006253404
0.275051046	-0.090861941097	-0.553758035	0.253285612	0.114889997	0.044138518
0.110121954	-0.026637716963	-0.361704819	0.098707279	0.070776906	0.010320938
0.064100331	-0.017288472306	-0.383287879	0.059852144	0.038354156	0.006673296
0.085725207	-0.020515576938	-0.347981472	0.083291261	0.043929448	0.010456419
0.143200577	-0.044348589854	-0.493053982	0.126722170	0.061128922	0.018532588
0.063985690	-0.023497353400	-0.551296101	0.056029689	0.030995412	0.007876985
0.036868193	-0.010466759187	-0.364613890	0.034037338	0.016574953	0.005388368
0.074447062	-0.030531917557	-0.647356649	0.057230271	0.036707855	0.006444104
0.040391299	-0.012950839039	-0.428576481	0.035432377	0.019496234	0.005846851
0.409086907	-0.069895717595	-0.263232291	0.400599267	0.296090872	0.032568564
0.030852610	-0.006807220412	-0.262968805	0.027368761	0.019155225	0.002410043
0.070853265	-0.016078240086	-0.319868364	0.071380705	0.040329196	0.009937761
0.043103569	-0.014103258035	-0.445323189	0.039754960	0.024409203	0.004814069
0.045271077	-0.011423899834	-0.334040753	0.039851622	0.026746577	0.004194382
0.055311355	-0.015494514290	-0.390700301	0.056395639	0.029984771	0.007602211
0.045232186	-0.014692855331	-0.446186954	0.036499864	0.023635108	0.004398232
0.020707480	-0.004827667980	-0.246762979	0.020516913	0.012012635	0.002332309
0.045498378	-0.013006014054	-0.385241999	0.037630432	0.027479393	0.003940664
0.059589434	-0.021722540708	-0.539840090	0.045834070	0.025725865	0.006089407
0.040917661	-0.010098791482	-0.318929913	0.038328986	0.024480376	0.004190308
0.041042761	-0.012002719596	-0.386751778	0.039325831	0.023671953	0.004612967
0.055979210	-0.020389044778	-0.533288862	0.056018957	0.025389655	0.008584486
0.043221106	-0.011222301769	-0.341650238	0.040950498	0.025290740	0.005248344
0.047539740	-0.010929398777	-0.303908625	0.043290914	0.029074997	0.005004615
0.569249005	-0.093137911531	-0.252897602	0.550013217	0.393658823	0.043992092
0.339411657	-0.070055243074	-0.322820795	0.364757985	0.224556572	0.046104134
0.808390876	-0.060422357622	-0.110652132	0.803604744	0.681447192	0.041237801
0.048079695	-0.014087892374	-0.400799153	0.040433308	0.021302738	0.005323559
0.667809846	-0.084707744731	-0.192600110	0.644710551	0.535838909	0.040579242
0.222941343	-0.047637643813	-0.330075032	0.203656058	0.128681265	0.023364550
0.020513672	-0.004895019296	-0.252261197	0.021016728	0.013004644	0.002579457
0.249487794	-0.069765481074	-0.451777314	0.214609005	0.142640314	0.024803309
0.072171702	-0.020791538716	-0.420869163	0.058374631	0.045152283	0.005012700
0.049206062	-0.015276092096	-0.430539383	0.041919684	0.027061222	0.005103540
0.333360641	-0.071862975501	-0.338784880	0.309681320	0.214792429	0.034884483
0.456583486	-0.086635958926	-0.296335058	0.431448678	0.333657481	0.034653019
0.052261056	-0.010596757515	-0.269162362	0.050529385	0.034333763	0.005681026
0.138032049	-0.023621284743	-0.250798236	0.129201151	0.107862478	0.007425459
0.023174998	-0.005230098721	-0.247511395	0.021985290	0.014471830	0.002405360

0.057058517	-0.020088558461	-0.5136822512	0.046381559	0.027665348	0.006871125
0.035474202	-0.010133027688	-0.3636983402	0.031952326	0.017855057	0.004164888
0.020834887	-0.004565244034	-0.2311669603	0.018750643	0.014105217	0.001423899
0.526498899	-0.079278177939	-0.2306844227	0.509419790	0.373671120	0.045113393
0.064861033	-0.019647948741	-0.4392047611	0.055711277	0.031361934	0.008340142
0.029237874	-0.007062940204	-0.2863096902	0.026775816	0.015591973	0.003321544
0.044215420	-0.014784907162	-0.4593907587	0.037872135	0.022161353	0.005227050
0.088526843	-0.020228470294	-0.3315345039	0.082503424	0.053348051	0.008681858
0.852766501	-0.045986651360	-0.0790228867	0.872657134	0.766345168	0.033722331
0.084716979	-0.019167068777	-0.3261930247	0.079300844	0.051891113	0.010022626
0.191861647	-0.039016613039	-0.3098671037	0.187593750	0.114193769	0.022934908
0.049489734	-0.013874167101	-0.3831144802	0.045139877	0.028848051	0.005925022
0.030189105	-0.007113765957	-0.2810485000	0.028554906	0.018994527	0.002775202
0.038420107	-0.010784754492	-0.3635177747	0.037746194	0.021996425	0.005071452
0.110022008	-0.027259836277	-0.3716904672	0.103560754	0.067677994	0.013512423
0.032942237	-0.009242045643	-0.3496405641	0.031168876	0.017459763	0.004207789
0.477315733	-0.085966417890	-0.2799983647	0.422713268	0.340115355	0.024856726
0.019036804	-0.003760742602	-0.2001109926	0.018329639	0.013174560	0.001669408
0.037258412	-0.010229296950	-0.3519110737	0.042751669	0.021010854	0.006185550
0.071610023	-0.019126763689	-0.3852766338	0.068988232	0.041395606	0.009843623
0.065044264	-0.019340525282	-0.4299676384	0.067216868	0.031945072	0.012745789
0.035755312	-0.012725854217	-0.4701859569	0.034318346	0.017224424	0.005365174
0.036109103	-0.010175186906	-0.3597054265	0.031189005	0.018195786	0.004477491
0.054052078	-0.013640716264	-0.3454964442	0.059719547	0.028055689	0.009179972
0.033629321	-0.011698253236	-0.4503372164	0.026671684	0.013419342	0.004183306
0.127066777	-0.035490679754	-0.4323179752	0.118854160	0.056517258	0.019081436
0.075552883	-0.022054618032	-0.4300992946	0.074787795	0.028666251	0.014129901
0.520084549	-0.078562924853	-0.2314274065	0.504251985	0.386386251	0.036314781
0.117781518	-0.026615908232	-0.3369602377	0.104919353	0.067834009	0.011059301
0.032892805	-0.007767884548	-0.2882407061	0.030158899	0.017270300	0.003873235
0.096160037	-0.043340568360	-0.7569571241	0.063958849	0.036138348	0.009751314
0.025299586	-0.006635444135	-0.3004041669	0.021550791	0.014841390	0.002332679
0.462567515	-0.067656789786	-0.2229166020	0.467249694	0.341015983	0.041133778
0.028698453	-0.006660600552	-0.2724984424	0.025214959	0.017887288	0.002266466
0.239137914	-0.050102152964	-0.3239169227	0.242704381	0.157574968	0.024902865
0.035772957	-0.008567356886	-0.2989757017	0.031823348	0.021477580	0.003277186
0.072556559	-0.016939845086	-0.3313194932	0.065381120	0.040398416	0.009542933
0.036385139	-0.009153900571	-0.3171490571	0.034410782	0.019736373	0.004534283
0.163053894	-0.061240946132	-0.6301341409	0.158120225	0.057460504	0.034834128
0.071917382	-0.014591373729	-0.2830056751	0.067518378	0.045908421	0.007186823
0.024619652	-0.005357293197	-0.2425451488	0.022242093	0.017334064	0.001604526
0.058665489	-0.015399935738	-0.3663823375	0.059011365	0.030886189	0.008330864
0.045780312	-0.009968121510	-0.2840244539	0.045542865	0.028879298	0.005679928
0.666775942	-0.058775676977	-0.1310708276	0.655065198	0.558540560	0.033708684

0.114339867	-0.020166461201	-0.25530191661	0.102429347	0.083083256	0.005786035
0.061086804	-0.014734324315	-0.3351026764	0.056592804	0.037646691	0.007094906
0.029754331	-0.006750359887	-0.2684724149	0.028817873	0.018296996	0.003225139
0.046007344	-0.015850936464	-0.4799858041	0.039478028	0.021345460	0.006207977
0.062534713	-0.0192698624041	-0.4454877777	0.063905558	0.031715204	0.009717972
0.066527371	-0.016492031998	-0.3501638637	0.060619700	0.035645104	0.010832113
0.044336804	-0.014064660026	-0.4321474226	0.039735742	0.019850583	0.006491038
0.086239323	-0.029096794708	-0.5193996252	0.082829233	0.040455922	0.015523252
0.037687755	-0.0091104570631	-0.30586676611	0.036959073	0.023189594	0.004956852
0.054796030	-0.011786420594	-0.2896515079	0.051615663	0.034939511	0.005346398
0.048005737	-0.012409477430	-0.3472801119	0.043339326	0.030142962	0.003827842
0.056504459	-0.020512658541	-0.5320743941	0.055160631	0.027812165	0.008808485
0.047425350	-0.014100458220	-0.4064915948	0.045280985	0.028750621	0.005866372
0.034612771	-0.0064355671891	-0.2247453186	0.033012624	0.024437091	0.003094431
0.053263355	-0.016372253527	-0.4320558937	0.051040768	0.026819261	0.007797912
0.026208715	-0.006275220513	-0.2745762604	0.024965157	0.017537858	0.002673255
0.034790886	-0.006690252957	-0.23339023231	0.031917832	0.022371222	0.003236002
0.057380101	-0.0144922467971	-0.3493861472	0.055270424	0.034624401	0.007409808
0.047634707	-0.014065216367	-0.4036195992	0.045746549	0.023899305	0.007075399
0.030646087	-0.007776491039	-0.30636274401	0.027526565	0.019769344	0.002623426
0.128857293	-0.032191700463	-0.3805080727	0.113577671	0.075320302	0.014986184
0.672927868	-0.090140968918	-0.2042196427	0.653411026	0.502423134	0.060852871
0.076384180	-0.019811927053	-0.3758461303	0.067033425	0.044587429	0.008055893
0.243252425	-0.047511895808	-0.2997500623	0.257361196	0.160113597	0.025695373
0.095724447	-0.031729846339	-0.5148171066	0.078917508	0.052984052	0.009561923
0.035313963	-0.0085790534621	-0.3028039472	0.032830083	0.018644274	0.004760979
0.040605177	-0.009382200252	-0.2958362943	0.037263005	0.024111424	0.004489621
0.254526630	-0.046641641836	-0.2798461649	0.256302642	0.177623946	0.024461650
0.049915275	-0.011424894423	-0.3052252734	0.047805600	0.033351001	0.004632882
0.050943915	-0.013736456696	-0.3684673662	0.046227288	0.023451930	0.006769000
0.760437371	-0.053434588998	-0.1036989678	0.750905709	0.667326967	0.031383796
0.039892013	-0.011311956827	-0.3709536283	0.039020841	0.022137217	0.004755839
0.726451195	-0.059051224039	-0.1205819951	0.716113419	0.623401756	0.032799223
0.046316563	-0.010700403051	-0.3040142833	0.039133284	0.032042741	0.002433286
0.075117274	-0.025277851835	-0.5083557022	0.065549353	0.042481604	0.007548318
0.052698597	-0.012869759896	-0.3314522519	0.046564609	0.031575416	0.005342345
0.164855169	-0.034038640876	-0.31234376661	0.159241150	0.105265392	0.018874186
0.840064007	-0.0521012129551	-0.0912499947	0.847789271	0.736784359	0.032646820
0.026255602	-0.0053875496891	-0.2320894718	0.023779489	0.018566978	0.001644105
0.028499842	-0.006914380736	-0.2855918773	0.025446648	0.016123732	0.003167318
0.677311556	-0.082868259958	-0.1853572119	0.693784680	0.493172478	0.069015671
0.059940150	-0.018106775257	-0.4322395617	0.052074486	0.030480493	0.006930552
0.405533115	-0.066128884883	-0.2500663788	0.395306390	0.283258496	0.031283297
0.043927341	-0.011106872973	-0.3327163295	0.039256137	0.027104976	0.004078955

0.043871108	-0.0119376674683	-0.3614105328	0.043512136	0.024416908	0.006422316
0.473431185	-0.0735282676913	-0.2380282829	0.435437609	0.354901396	0.030225313
0.053003430	-0.0146444649662	-0.3816470005	0.045521624	0.032169375	0.003703114
0.114572632	-0.0346029703828	-0.4694766448	0.105732555	0.050801524	0.016573518
0.026161844	-0.0058935238146	-0.2566596314	0.025777480	0.015272562	0.003704795
0.065486026	-0.0177326544531	-0.3863044249	0.064852234	0.035292845	0.009989344
0.039303372	-0.0109429707231	-0.3620685071	0.036353338	0.020578176	0.005731786
0.037857149	-0.0137749634915	-0.4897165967	0.035903515	0.016860826	0.005761785
0.042811680	-0.0119011704831	-0.3683855252	0.043482208	0.022257590	0.006440987
0.031369567	-0.0085173415956	-0.3325787494	0.028908285	0.016828302	0.003567174
0.049648997	-0.0142878893905	-0.3950420043	0.041986627	0.028758577	0.004827651
0.890163527	-0.0267640423076	-0.0435454305	0.885830896	0.841191859	0.018618570
0.053637063	-0.0200902640672	-0.5473005741	0.043430593	0.021688522	0.008922605
0.045712665	-0.0097667087610	-0.2780674328	0.042278635	0.030108804	0.004050080
0.032521206	-0.0067778603991	-0.2505078351	0.031961663	0.021900097	0.003946221
0.241697722	-0.0399515229158	-0.2493560854	0.229684277	0.178700188	0.021102082
0.048226101	-0.0128585931184	-0.3600066025	0.045775377	0.028232555	0.005761655
0.067582087	-0.0174386034336	-0.3673151506	0.067607162	0.033892220	0.009261851
0.035453053	-0.0077498917719	-0.2696917034	0.035829952	0.022206303	0.004668029
0.047355346	-0.0130741738260	-0.3732345510	0.049016531	0.022989183	0.007918815
0.083500794	-0.0189598599035	-0.3269457167	0.078742636	0.053634512	0.008569430
0.073995995	-0.0166159674359	-0.3179995213	0.074511412	0.044882439	0.010539067
0.400824570	-0.0617697965283	-0.2350690253	0.389244844	0.290598403	0.033759621
0.080732564	-0.0171752789739	-0.3027521943	0.076257475	0.055073770	0.007786500
0.226265828	-0.0441840023856	-0.2986899609	0.228729779	0.148419124	0.027256978
0.034749875	-0.0084322927413	-0.3012154849	0.034050546	0.018652602	0.004827771
0.035955722	-0.0095936723653	-0.3378109737	0.032129696	0.021097640	0.004238021
0.110784736	-0.0256083240164	-0.3437621718	0.100453581	0.056473897	0.014721384
0.037996700	-0.0098663497869	-0.3319954350	0.035673406	0.020677831	0.005089182
0.037603214	-0.0084386666569	-0.2815106924	0.033392511	0.026348078	0.002615563
0.019696565	-0.0049826886711	-0.2649747473	0.017623631	0.011953992	0.001987738
0.042025013	-0.0092553013824	-0.2826159379	0.039965341	0.024390670	0.005026252
0.041033044	-0.0117681612285	-0.3781921068	0.039339445	0.022059124	0.004505472
0.030748210	-0.0081866074634	-0.3235649780	0.034018917	0.015902364	0.005989643
0.519633481	-0.0684460093390	-0.1996410031	0.513157114	0.378425231	0.039013024
0.039647365	-0.0089635127927	-0.2872608589	0.037141451	0.025851498	0.003480806
0.040321518	-0.0123676885196	-0.4069299825	0.036227618	0.021470258	0.004738648
0.035442182	-0.0097321782263	-0.3477038204	0.039176878	0.020116049	0.005685821
0.432829053	-0.0991716588816	-0.3657788758	0.396307172	0.288243647	0.039478157
0.040535473	-0.0162709929449	-0.5605826307	0.030564125	0.016928192	0.004989708
0.027828576	-0.0061410408709	-0.2555610259	0.027513338	0.018275248	0.002847324
0.172765998	-0.0472179338562	-0.4311931853	0.157068008	0.065871810	0.029105336
0.047609075	-0.0107280049810	-0.2972905931	0.042538659	0.030845058	0.003760191
0.077885737	-0.0238545656453	-0.4568546413	0.074056682	0.043962294	0.009258374

0.024755366	-0.005842473139	-0.2655227659	0.023238602	0.015306715	0.002465691
0.026693239	-0.007030319280	-0.3068536503	0.028112054	0.014453642	0.004547395
0.145683214	-0.032744280254	-0.3406715208	0.146505678	0.083817582	0.021942023
0.037989845	-0.007172402212	-0.2335433480	0.039709955	0.023915408	0.004551649
0.332246334	-0.052009065272	-0.23780230331	0.344000241	0.227333207	0.035215037
0.116860964	-0.022411911703	-0.2804488064	0.112178338	0.074956563	0.013320253
0.074549140	-0.014878070561	-0.27923036831	0.077900497	0.045917714	0.010292212
0.150239836	-0.044085653443	-0.4641917428	0.137419442	0.081286186	0.019901142
0.296556502	-0.049269029071	-0.2527717880	0.283354931	0.203257575	0.021757075
0.094658329	-0.0345305332091	-0.5776288398	0.086194516	0.028839687	0.019172657
0.219531790	-0.051611207751	-0.3674605645	0.202328973	0.122809505	0.024569620
0.049576853	-0.021864092036	-0.6596991415	0.045087717	0.019823322	0.008180421
0.292481928	-0.045101845263	-0.2329485344	0.315890772	0.224574594	0.029010528
0.038865085	-0.009590206097	-0.3151971685	0.035801732	0.020228994	0.005679941
0.070923309	-0.014557253361	-0.2861097796	0.065433971	0.047529344	0.005805195
0.661901128	-0.059923305941	-0.13476958121	0.673830032	0.581830164	0.029060882
0.022960285	-0.004725607987	-0.22326058211	0.021813112	0.015139583	0.002244269
0.043425250	-0.009600186545	-0.2857655710	0.038117400	0.026437746	0.003816332
0.022246506	-0.004925612296	-0.2391381695	0.019823577	0.014612273	0.001823872
0.817846336	-0.095658356558	-0.1771489122	0.801084764	0.516763673	0.097012860
0.083313671	-0.017595848368	-0.3014555344	0.075111769	0.053364194	0.007143701
0.044792340	-0.009781094208	-0.2836887179	0.040503077	0.031359949	0.003137409
0.049970945	-0.010594070622	-0.2804281888	0.042639171	0.034486529	0.002667190
0.070113989	-0.013898132522	-0.2748774412	0.064825799	0.042100646	0.007725876
0.397990012	-0.063810278457	-0.2453716844	0.390092796	0.298330853	0.029773239
0.049725880	-0.009820918133	-0.25917293961	0.046708630	0.028923743	0.006182792
0.054784430	-0.014113562430	-0.3544905924	0.047838252	0.033711829	0.005029221
0.802783289	-0.1402576382451	-0.2732814456	0.822325816	0.379877583	0.157303636
0.863666261	-0.045661399306	-0.0774430392	0.865131970	0.765260532	0.034801591
0.034526933	-0.007867661345	-0.2805001148	0.034887776	0.021787562	0.003790744
0.047693693	0.0237804026082	0.49792781746	0.110684315	0.044116310	0.020687617
0.174501208	-0.045948378989	-0.4131940919	0.161717152	0.103603119	0.017147260
0.443819863	-0.057019987322	-0.1937081810	0.416326230	0.357847315	0.023647939
0.063518737	-0.015975125677	-0.3534562126	0.059930608	0.035042050	0.009113026
0.783247122	-0.072667337476	-0.1386122034	0.761553798	0.631868212	0.048257527
0.041983374	-0.012465019660	-0.3955273974	0.039026081	0.024425596	0.004889107
0.075661997	-0.016384038409	-0.3062588883	0.075442706	0.052843733	0.009031636
0.076645434	-0.016405484006	-0.3028319052	0.071101239	0.049491852	0.006292936
0.723223555	-0.082566240623	-0.1723552230	0.720727591	0.474393541	0.076252589
0.105898773	-0.041353750772	-0.6366813644	0.080330002	0.052911305	0.007634645
0.054054358	-0.014372341391	-0.3665729933	0.051082952	0.027755128	0.008178034
0.073215866	-0.020581526531	-0.4099047802	0.067725871	0.032527315	0.011411063
0.032006127	-0.007575525337	-0.2869083775	0.031181261	0.019704313	0.003419732
0.749269334	-0.053556929925	-0.1055313725	0.739964505	0.640411323	0.035812918

0.118296718	0.0718623871527	0.64166297726	0.347678533	0.122519486	0.077160581
0.099910462	-0.039432812209	-0.6410909943	0.070061247	0.042583141	0.008092615
0.159722958	-0.032765623560	-0.3094551978	0.146271556	0.103566413	0.015626496
0.060377911	-0.014890760137	-0.3429689620	0.054824055	0.037097450	0.004844679
0.441140702	-0.098465777809	-0.3552384505	0.423247068	0.263324512	0.050477491
0.632607068	-0.054009494852	-0.1266552550	0.623042943	0.540493873	0.023815848
0.082219619	-0.029940561535	-0.5663265893	0.076852229	0.033512900	0.013161581
0.818219479	-0.053521036291	-0.0963782944	0.822594584	0.731057063	0.029105970
0.050333246	-0.013862378922	-0.3766266380	0.044641141	0.028542350	0.005719802
0.149487848	-0.023385917474	-0.2287589724	0.148785036	0.107864821	0.012421475
0.041683931	-0.010903215403	-0.3418287069	0.038789818	0.021015215	0.005890983
0.646888278	-0.103218437168	-0.2466220825	0.640438309	0.428943388	0.085287871
0.050042436	-0.013120446713	-0.3557183274	0.045991333	0.029040620	0.006250464
0.033513712	-0.008619285127	-0.3184734491	0.030150711	0.019169522	0.004081434
0.022272844	-0.004543060484	-0.2188843652	0.022403792	0.014009081	0.002321049
0.815598741	-0.072764004432	-0.1331075736	0.813133682	0.582105090	0.069645608
0.014767689	-0.002791918574	-0.1725455103	0.015262892	0.010112724	0.001790931
0.329214216	-0.050044895956	-0.2302821373	0.319111912	0.236099078	0.026082746
0.785713317	-0.055342428632	-0.1040005748	0.796005660	0.681868711	0.039624560
0.052875334	-0.013875526139	-0.3597180632	0.052860889	0.023982146	0.008640616
0.284023255	-0.069706909092	-0.3903955120	0.312352360	0.162437730	0.051958291
0.043896914	-0.012512790049	-0.3811252461	0.043286815	0.021874029	0.006691575
0.020710868	-0.004957958344	-0.2540138396	0.019533271	0.013179219	0.002325223
0.085006120	-0.022802528543	-0.3959498461	0.072722914	0.045339488	0.008456057
0.060611057	-0.013346316474	-0.3022470067	0.054579518	0.038507779	0.005421629
0.124714345	-0.051671533190	-0.6979762812	0.139853079	0.040966975	0.031114861
0.613107976	0.2048143855770	0.41001330108	0.874656867	0.780345639	0.029900219
0.024726588	-0.005771104610	-0.2622040805	0.023422596	0.015315675	0.002623534
0.214074418	0.0912167903735	0.49270699274	0.411314600	0.197038581	0.067172172
0.078857336	-0.022583975376	-0.4230618095	0.073446495	0.045292362	0.008870042
0.095269209	-0.017234810655	-0.2579442577	0.089613314	0.061658678	0.008205272
0.072145360	-0.020759659723	-0.4202764613	0.061611034	0.036432891	0.009151329
0.035567092	-0.008259848078	-0.2885365120	0.036514480	0.020447404	0.005081991
0.059353942	-0.014230056487	-0.3313003026	0.051467765	0.036373089	0.005199430
0.056600894	-0.011435106996	-0.2717677345	0.057916608	0.036945248	0.005725258
0.781196138	-0.073022863507	-0.1397034171	0.794153461	0.642649910	0.051503478
0.047042507	-0.010916715088	-0.3064637038	0.045469504	0.031073111	0.004607668
0.201563086	-0.051439841314	-0.4019051685	0.195530767	0.104743403	0.036447880
0.072276148	-0.024549872045	-0.5112460938	0.066356783	0.038681883	0.009283270
0.062669772	-0.015454930548	-0.3449659498	0.055776792	0.039928565	0.005651479
0.033816384	-0.011252538000	-0.4281993199	0.025972257	0.011761268	0.004346916
0.365616222	-0.048334077439	-0.1987244751	0.373120160	0.279099764	0.030074613
0.034883100	-0.007326663004	-0.2571121214	0.030925149	0.021906079	0.002857344
0.038219936	-0.014614369212	-0.5209294994	0.031070525	0.015736696	0.005105005

0.032925271	-0.008934994674	-0.3367051491	0.030328329	0.019214234	0.004519533
0.142058012	-0.025326709855	-0.2628489241	0.145833604	0.104095937	0.012175558
0.559824198	-0.058975777893	-0.1576216123	0.529920847	0.463730553	0.020250318
0.021110913	-0.004858078680	-0.2449475400	0.018811708	0.014721494	0.001555928
0.518842184	-0.068440512628	-0.1999441896	0.507110114	0.396096322	0.032546717
0.042296588	-0.010574781167	-0.3259151820	0.044260491	0.022490028	0.007990278
0.081019241	-0.024022104300	-0.4420721141	0.067673771	0.048336315	0.007259957
0.062140848	-0.021465439976	-0.5095305795	0.053375819	0.032647574	0.006867940
0.458254234	-0.066623535767	-0.2214224947	0.447479596	0.331346349	0.043559475
0.052815306	-0.009844744921	-0.2459253464	0.048536072	0.034904398	0.004201976
0.069323551	-0.013424734176	-0.2674966979	0.065492426	0.047599071	0.005023446
0.033016557	-0.008478804145	-0.3167178371	0.033012461	0.018458398	0.004895393
0.356795653	-0.058912294603	-0.2525926512	0.346852793	0.259781521	0.029272404
0.035309522	-0.009918182168	-0.3564178868	0.036048291	0.019918102	0.004773627
0.103390530	-0.038843480004	-0.6050769860	0.086505430	0.049169092	0.012676353
0.358156141	-0.043213797689	-0.1801340121	0.345774399	0.293063046	0.017455668
0.025241892	-0.005497063394	-0.2446525075	0.023222690	0.015506085	0.002605591
0.157755626	-0.031766123752	-0.3028658634	0.141841504	0.096217527	0.014806248
0.024689378	-0.006290874285	-0.2886790521	0.028244858	0.013198154	0.004251758
0.046323463	-0.010481259431	-0.2970596425	0.043611388	0.028247227	0.005148572
0.065401243	-0.014410540684	-0.3059989740	0.062373346	0.040179725	0.006461499
0.097890805	-0.030633472187	-0.4818281355	0.093318275	0.054343363	0.012916947
0.044443305	-0.012164543783	-0.3648215380	0.041507936	0.022705286	0.005730817
0.506520349	-0.106900070812	-0.3345380491	0.520570950	0.323023196	0.064568664
0.061454006	-0.014020065716	-0.3151113522	0.057094936	0.039627531	0.005998169
0.807590424	-0.050502887825	-0.0919870785	0.823494762	0.692305810	0.037555233
0.077145755	-0.024412334370	-0.4741961560	0.064795051	0.036215053	0.008772912
0.056922673	-0.018585690132	-0.4693676430	0.059734362	0.031229681	0.008907293
0.403700916	-0.053982700381	-0.2017209341	0.405665700	0.296782676	0.036913796
0.183475076	-0.057829224976	-0.5123028040	0.168540886	0.088399671	0.026855791
0.055034591	-0.010541752959	-0.2551406419	0.050791554	0.037566763	0.005021016
0.034281569	-0.010362303653	-0.3846014164	0.029024868	0.018829514	0.003853282
0.055867695	-0.010334885266	-0.2462306705	0.052819374	0.040163661	0.003969459
0.043375453	-0.011674548513	-0.3560977288	0.039274983	0.020717938	0.005846453
0.058070807	-0.012496063525	-0.2926068276	0.053242924	0.035701271	0.006269576
0.067563803	-0.013182921280	-0.2687512041	0.069515222	0.046712668	0.007757285
0.046595434	-0.009974088362	-0.2796950146	0.041461530	0.030556037	0.003895895
0.033129095	-0.008524896011	-0.3177143259	0.030613124	0.017645539	0.004841093
0.187995277	-0.032792438754	-0.2612275424	0.175228456	0.132659598	0.014783140
0.039373886	-0.010058525533	-0.3286551145	0.034242178	0.022900824	0.004211878
0.143048152	-0.025228957880	-0.2598811278	0.139124193	0.095510968	0.014418750
0.034788499	-0.007842855648	-0.2777240613	0.035575812	0.019859154	0.005410859
0.043055295	-0.013019138972	-0.4061932763	0.045456886	0.022890286	0.007313364
0.028882948	-0.006697158186	-0.2727137289	0.025288430	0.018253191	0.002717449

0.029782170	-0.006713932261	-0.2666758055	0.027639393	0.017242831	0.003673116
0.042442180	-0.012864735176	-0.4060491772	0.042575364	0.022681706	0.005502450
0.041194081	-0.008479947662	-0.2612635057	0.040873148	0.023190861	0.004807495
0.049602671	-0.013762370299	-0.3787604702	0.043324777	0.029127683	0.005373203
0.428062636	-0.063000405010	-0.2240071765	0.451919277	0.321446881	0.039253883
0.041880299	-0.008803170811	-0.2682647206	0.039201048	0.028237768	0.004120552
0.117200584	-0.040704553920	-0.5563994652	0.111195981	0.046030140	0.022039793
0.071965986	-0.027263790252	-0.5834265974	0.055745604	0.035448600	0.006100174
0.052283321	-0.012462656529	-0.3221016061	0.048322083	0.028419304	0.006171280
0.782471900	-0.045233568770	-0.0847913169	0.784492670	0.690173396	0.031798372
0.546862260	-0.066904784979	-0.1846639819	0.524886901	0.397346423	0.043798374
0.033418757	-0.008218305719	-0.3027244954	0.033892164	0.017966191	0.004659944
0.050027093	-0.009232087848	-0.2409271610	0.047886752	0.034345668	0.005140444
0.338777805	-0.061087511614	-0.2777916890	0.323704972	0.229670722	0.027305165
0.059142486	-0.011280905383	-0.2569667400	0.056601723	0.037702419	0.005331241
0.228835642	-0.042005972656	-0.2790704449	0.207977116	0.134893253	0.021851436
0.041199074	-0.012416120956	-0.4006950566	0.037859638	0.023028966	0.005025851
0.038005041	-0.010579540128	-0.3591642835	0.035635355	0.020180255	0.005180236
0.093300573	-0.018176830806	-0.2792147750	0.098541013	0.056364215	0.012594434
0.029224456	-0.006930048247	-0.2804690704	0.025217050	0.016902435	0.003028162
0.293830876	-0.041199485006	-0.2102291225	0.314069061	0.214080766	0.030353335
0.073050237	-0.013952387833	-0.2653434801	0.072302262	0.047741436	0.009321729
0.047141180	-0.011469348678	-0.3232261852	0.051111085	0.022468738	0.008970909
0.030740074	-0.009342207028	-0.3757820401	0.025286066	0.017066501	0.002734949
0.106438556	-0.021275705305	-0.2910984577	0.095245802	0.071646052	0.008605088
0.464345705	-0.083111991844	-0.2779081891	0.436478172	0.325736770	0.041509342
0.044273048	-0.012566013991	-0.3799452124	0.040464020	0.024361677	0.004560126
0.020602394	-0.004602434937	-0.2351351582	0.020814334	0.012592079	0.002436048
0.834471092	-0.054156749578	-0.0956214473	0.856589465	0.720330825	0.041296579
0.028898223	-0.006251925903	-0.2527848814	0.028603946	0.017492774	0.003202974
0.066138171	-0.026323472817	-0.6120484226	0.051779750	0.033002564	0.005804793
0.018412753	-0.004045902259	-0.2216188147	0.018152241	0.010389390	0.002671722
0.086907442	-0.029467409901	-0.5230022333	0.079742525	0.038050873	0.016807487
0.048079937	-0.013297473191	-0.3751059835	0.045800077	0.024722755	0.008336819
0.045434926	-0.010197288236	-0.2932716109	0.043398867	0.030755965	0.004961363
0.062629723	-0.011371688437	-0.2456609952	0.057174547	0.042425106	0.004571925
0.312742510	-0.041042871049	-0.1962259000	0.316016637	0.239141535	0.023848226
0.055507819	-0.014091540616	-0.3494419252	0.063974927	0.028829225	0.010020899
0.052294991	-0.011530461111	-0.2952953508	0.049770005	0.033686100	0.004403982
0.880942036	-0.037886570062	-0.0626920250	0.906258276	0.815166296	0.027613996
0.418547595	-0.057445339211	-0.2076386695	0.418881282	0.326293476	0.034865514
0.033143439	-0.007860856129	-0.2901849965	0.035451093	0.021152869	0.004160592
0.035587148	-0.009546548280	-0.3390041314	0.032599698	0.019355080	0.004464158
0.034270908	-0.008584152493	-0.3109702131	0.033039961	0.019350897	0.004507762

0.022319929	-0.004408627081	-0.2115746043	0.021964432	0.013456099	0.002742207
0.790299594	-0.049978925061	-0.0930328767	0.792810458	0.711748134	0.026195353
0.046187398	-0.010488913240	-0.2981002610	0.042568168	0.028172303	0.004851986
0.549513709	-0.068162924125	-0.1874201194	0.552478949	0.397124998	0.050798160
0.433119300	-0.066950154454	-0.2363136446	0.433032159	0.281895721	0.047068024
0.351814919	-0.041837872834	-0.1772834810	0.335811537	0.294533699	0.011348860
0.063285220	-0.022333431115	-0.5243894665	0.058949863	0.029669967	0.009521210
0.043449543	-0.009114780286	-0.2697392584	0.045893054	0.025500996	0.005949183
0.335540991	-0.074642520721	-0.3511046185	0.341376778	0.201397905	0.047101258
0.377078392	-0.072311303046	-0.2983410680	0.398807229	0.222638937	0.049990320
0.867549225	-0.041544648783	-0.0699692142	0.858473965	0.790309117	0.024855305
0.242073434	-0.038880920389	-0.2416873027	0.240226768	0.165196796	0.023222564
0.439910012	-0.071028272425	-0.2478888615	0.434082897	0.287303978	0.046933957
0.115018784	-0.033501021974	-0.4500211946	0.103429560	0.053129417	0.016900485
0.399085570	-0.052223660079	-0.1970367264	0.381804637	0.314672339	0.021487717
0.029946641	-0.006594473255	-0.2602936582	0.032654924	0.019319664	0.003845205
0.030482619	-0.007612092937	-0.3005080758	0.030585532	0.016714941	0.003888460
0.154129242	-0.044160097683	-0.4521688990	0.183589118	0.078743298	0.031561786
0.039309118	-0.008178495507	-0.2616415400	0.038528273	0.023488232	0.005144040
0.641043719	-0.068242875094	-0.1597514602	0.657507569	0.522476897	0.042491938
0.070028901	-0.025271719738	-0.5474729081	0.064185399	0.032845117	0.010362135
0.697402042	-0.076424128560	-0.1649408770	0.667561522	0.591187836	0.028652746
0.041523103	-0.009463608848	-0.2927879810	0.038251407	0.026638019	0.004203783
0.034202302	-0.010074180073	-0.3731604791	0.031596180	0.017903593	0.004163614
0.070501490	-0.014220895535	-0.2804289406	0.070880526	0.040495636	0.010422376
0.658554512	-0.079257131028	-0.1820493438	0.676478809	0.483533242	0.058010862
0.126072696	-0.025207360897	-0.2955692659	0.112743618	0.089034325	0.008839405
0.026123874	-0.007706607808	-0.3461848388	0.024127758	0.011333777	0.004024400
0.276761354	-0.035107683595	-0.1884109689	0.265936350	0.203468851	0.019215448
0.312689148	-0.046585144939	-0.2249332558	0.310310694	0.232811097	0.028182842
0.052560391	-0.011697703729	-0.2986418383	0.054347058	0.028859832	0.009148415
0.065426480	0.0172236565902	0.2967220013	0.110843554	0.061536604	0.014630060
0.029775641	-0.006657194137	-0.2642501614	0.030385586	0.015881417	0.004855779
0.036193402	-0.008648318579	-0.2990627990	0.033774368	0.023668748	0.003548313
0.051331693	-0.009369925858	-0.2391822769	0.053281259	0.034829320	0.006490102
0.306063544	-0.049181174632	-0.2440106363	0.300711134	0.218375703	0.023479344
0.057259449	-0.017028031538	-0.4211469693	0.052203966	0.029157876	0.007547975
0.032860774	-0.006791636347	-0.2488929708	0.034911654	0.022428306	0.003803676
0.801461637	-0.046612576052	-0.0853478234	0.819763523	0.697188542	0.039677240
0.025190823	-0.006019022520	-0.2706248159	0.025110420	0.016382624	0.002834500
0.033670232	-0.007430398251	-0.2690738526	0.031190391	0.020925767	0.003785828
0.040806836	-0.009690900267	-0.3053249569	0.038905938	0.023997495	0.005684622
0.044010206	-0.008276997054	-0.2399898957	0.042661487	0.030905170	0.004019513
0.047822515	-0.012500160989	-0.3514085619	0.045610218	0.027115619	0.007385126

0.8121660599	-0.0567851242448	-0.1032518504	0.8191923424	0.6295862741	0.0541381167
0.0494825619	-0.0117770934334	-0.3183121333	0.0458507961	0.0263559120	0.0064460554
0.0485610684	-0.0149262422030	-0.4244618231	0.0460340759	0.0235033945	0.0071160529
0.3489545935	-0.0380938877362	-0.1618542390	0.3503278501	0.2644511359	0.0240774218
0.8516711017	-0.0357156933045	-0.0610734046	0.8611535351	0.7621196218	0.0314297458
0.2973362119	-0.0381278122991	-0.1910943381	0.3026459844	0.2372480531	0.0243031244
0.0516999329	-0.0142804884095	-0.3797901580	0.0491419908	0.0312645007	0.0054051057
0.0533221105	-0.0195232852803	-0.5318171656	0.0513540251	0.0247167170	0.0083380590
0.1091891790	-0.0405139494765	-0.5992718738	0.1109569781	0.0443189730	0.0220819965
0.0460488787	-0.0106523733489	-0.3041042684	0.0451701291	0.0276045737	0.0052272743
0.4259209683	-0.0533609275968	-0.1883804119	0.4538272918	0.3207812431	0.0426434589
0.4819358351	-0.0573941049965	-0.1789754228	0.4759955819	0.3730902255	0.0341093514
0.1555346614	-0.0396936889549	-0.3955316108	0.1454028894	0.0904032087	0.0203403072
0.0350008373	-0.0096330071378	-0.3475141339	0.0355891571	0.0208178949	0.0046174477
0.0339095511	-0.0075659601900	-0.2728338139	0.0323972207	0.0171867391	0.0043500367
0.0258112630	-0.0049950014785	-0.2167215449	0.0262299747	0.0170344319	0.0027250443
0.0265352810	-0.0059149509116	-0.2548004681	0.0276024094	0.0162523997	0.0033137038
0.0372463337	-0.0095301945494	-0.3250203801	0.0345628909	0.0192665740	0.0049774359
0.0290612661	-0.0050183433092	-0.1983838054	0.0287237831	0.0182684415	0.0029589161
0.1121098720	-0.0465100671173	-0.6917254238	0.1030891637	0.0292168601	0.0224176948
0.0835120538	-0.0259648648653	-0.4692566104	0.0683059040	0.0425622988	0.0092292374
0.0310342837	-0.0069500843968	-0.2677266773	0.0283111184	0.0194531468	0.0033543829
0.0368173051	-0.0098472896834	-0.3406862461	0.0344762349	0.0180094309	0.0062082987
0.0252272915	-0.0074019479718	-0.3402940563	0.0228250234	0.0142433624	0.0029927021
0.0769545671	-0.0243211680548	-0.4733296291	0.0598558367	0.0415241310	0.0073079854
0.0750936590	-0.0186345726077	-0.3565851686	0.0701009785	0.0405042754	0.0097739798
0.2575313484	-0.1913342028908	############	0.0988322311	0.0333165590	0.0212550534
0.0303592694	-0.0062837814185	-0.2441657407	0.0318725931	0.0174701331	0.0045322268
0.7815473707	-0.0470623638621	-0.0884328973	0.7708000458	0.6990151827	0.0209235014
0.0671121108	-0.0130343187435	-0.2671330221	0.0665216339	0.0469072321	0.0062090327
0.0449552110	-0.0140850483157	-0.4272083012	0.0451221970	0.0188795891	0.0073375228
0.0395428107	-0.0095866461444	-0.3102576153	0.0447405351	0.0193151371	0.0070282057
0.1262045721	-0.0314360565491	-0.3785699008	0.1235638979	0.0739698049	0.0155286193
0.0373159234	-0.0078316840700	-0.2610488834	0.0345316124	0.0254157808	0.0031995010
0.0313621284	-0.0053957573363	-0.2016618235	0.0286939250	0.0205749160	0.0024229627
0.0920008027	-0.0201015660147	-0.3166585945	0.0906951611	0.0616671849	0.0108783161
0.3148617490	-0.0425407861411	-0.2024896268	0.2951086787	0.2322668941	0.0183797397
0.4284120550	-0.0504934900258	-0.1765336466	0.4137135350	0.3375040190	0.0301326030
0.3053882400	-0.0657568297357	-0.3373293917	0.2929026280	0.1957628764	0.0283973109
0.0181726541	-0.0033031801491	-0.1799194910	0.0181741860	0.0128096021	0.0017493151
0.3406796250	-0.0677276231285	-0.3095962657	0.3410129499	0.1644174271	0.0526146534
0.0966279288	-0.0134285303108	-0.1941928204	0.0887350280	0.0722281258	0.0049101891
0.2984149671	-0.0590318926609	-0.3065092263	0.2848284117	0.2044210901	0.0301277990
0.8789052917	-0.0379538585765	-0.0629529210	0.8930841430	0.7960946329	0.0320832268

0.670175329	-0.048846240849	-0.1075144540	0.667080313	0.584153999	0.031423014
0.048049119	-0.012759874215	-0.3581056857	0.047387924	0.027519108	0.007218935
0.033333882	-0.008934481647	-0.3331120668	0.029847376	0.017299217	0.004710499
0.594649141	-0.081265243963	-0.2082286965	0.579571509	0.397376093	0.051169307
0.035632061	-0.007139865085	-0.2454818377	0.032206031	0.023702227	0.002391119
0.712117715	-0.100777455896	-0.2168504998	0.693278120	0.524990007	0.059991442
0.167418830	-0.055695318855	-0.5435512791	0.156618278	0.083749758	0.025557381
0.908378365	-0.023492316511	-0.0373846690	0.918057250	0.860742694	0.016065333
0.528818524	-0.075640019668	-0.2182311488	0.523421582	0.358861929	0.062568120
0.570716304	-0.051411426447	-0.1337345906	0.583111489	0.484234553	0.034354535
0.288507644	-0.057082179674	-0.3061900228	0.286817573	0.187520352	0.032387541
0.697079908	-0.064643147947	-0.1383188191	0.682409829	0.564075190	0.039708225
0.038899753	-0.009427683539	-0.3089950114	0.040974484	0.024107696	0.005774618
0.031719628	-0.006566619558	-0.2470781289	0.030491313	0.020756086	0.003199723
0.912949397	-0.021262265879	-0.0336246190	0.913142643	0.868559120	0.014250713
0.060351876	-0.016995511297	-0.3989285149	0.050649376	0.037535669	0.005208153
0.043338546	-0.009058194538	-0.2685118242	0.042387424	0.029068532	0.004869147
0.377443454	-0.049449877901	-0.1969899425	0.381519693	0.290914413	0.031636813
0.036392983	-0.007484135119	-0.2538083466	0.035791759	0.022906058	0.004472589
0.047229051	-0.009910190263	-0.2743323390	0.043383760	0.030668825	0.004243291
0.331992561	-0.044721055965	-0.2021837635	0.350597715	0.257875854	0.034123055
0.134800284	0.0844427598929	0.66281352489	0.368779357	0.099110026	0.072953024
0.079080169	-0.025038267164	-0.4760881417	0.079000896	0.037309707	0.013674719
0.037572415	-0.007378871829	-0.2431615012	0.039044671	0.022038715	0.005893985
0.018436536	-0.003024850514	-0.1622536627	0.018742433	0.012792059	0.001989349
0.024524223	-0.004666445496	-0.2095021622	0.023850225	0.017051968	0.002370574
0.035855275	-0.009377169505	-0.3300568993	0.036159571	0.016721175	0.006671954
0.036700802	-0.007652272688	-0.2581791075	0.035875213	0.022535443	0.004760653
0.908053480	-0.028717820376	-0.0458501639	0.918404005	0.835464111	0.025513928
0.028434901	-0.004478276650	-0.1787239011	0.028874672	0.020717372	0.002804375
0.913562441	-0.017898896468	-0.0282343171	0.917363341	0.877976236	0.013860352
0.864252099	-0.035407419161	-0.0596456481	0.881697790	0.786642103	0.026441531
0.308573901	-0.050199524542	-0.2473811449	0.309795000	0.207005223	0.029080019
0.117203230	-0.038015600637	-0.5122197800	0.107280076	0.062118586	0.017505071
0.254858680	-0.038817163357	-0.2286350146	0.278039889	0.183072201	0.029718317
0.545867694	-0.060173932302	-0.1652924349	0.584402751	0.392233849	0.053978602
0.064576303	-0.014255240041	-0.3060554256	0.060466724	0.040000226	0.006445932
0.130670889	-0.018904064540	-0.2082026785	0.127069064	0.096490764	0.008806285
0.887087049	-0.043858282886	-0.0723153779	0.912817431	0.800864948	0.039084841
0.340924753	-0.051401643443	-0.2284943959	0.336965674	0.213995574	0.044089330
0.025238546	-0.005297708961	-0.2350398401	0.026314094	0.015433374	0.003449310
0.043565057	-0.008688924164	-0.2553438093	0.045197357	0.027866892	0.006003724
0.021715531	-0.004262146120	-0.2082054528	0.022055599	0.012992369	0.002681530
0.550159344	-0.068314050770	-0.1876326807	0.547717955	0.437824038	0.041579832

0.022655503	-0.004203490005	-0.1987954010	0.023813561	0.014725371	0.002949564
0.037841142	-0.007540187950	-0.2474378347	0.034702091	0.023250420	0.003913284
0.552062834	-0.059710715475	-0.1620324599	0.567369745	0.442695796	0.042524333
0.073241678	-0.016442897795	-0.3174842869	0.079267816	0.042497266	0.010304265
0.028005740	-0.005119442613	-0.2087307323	0.025364232	0.018203343	0.002296235
0.864309594	-0.042831002626	-0.0724651121	0.870557819	0.785183161	0.027852096
0.191175268	-0.035755431544	-0.2823207067	0.217018258	0.127683566	0.030021406
0.037480105	-0.007720717895	-0.2560276026	0.032311717	0.026506560	0.001762386
0.034310739	-0.006651927674	-0.2346689244	0.038340406	0.023042166	0.004899905
0.276850141	-0.035507983292	-0.1906445514	0.269616388	0.203589174	0.026325387
0.029073781	-0.004967966054	-0.1961831267	0.030127682	0.020404886	0.003069929
0.030986917	-0.008307264088	-0.3267708139	0.030898443	0.018167486	0.004142980
0.164522504	-0.044316358965	-0.4226155395	0.139686335	0.105875480	0.012489200
0.050466031	-0.009200725219	-0.2381420817	0.052402091	0.029223360	0.007604356
0.027985164	-0.008660522442	-0.3733225733	0.022897365	0.015720211	0.002486483
0.034766534	-0.007306836309	-0.2570813658	0.031619792	0.021831140	0.003345216
0.035905820	-0.007700519622	-0.2649042670	0.036167911	0.021705107	0.004557344
0.024608814	-0.004513462072	-0.2015988152	0.025046476	0.015787408	0.002950389
0.395118362	-0.080453038246	-0.3193901722	0.410236929	0.264526312	0.048653677
0.510103732	-0.047262239958	-0.1374427516	0.516308366	0.424286173	0.027640147
0.048907767	-0.010405663968	-0.2804105598	0.046548532	0.027252634	0.006816056
0.025906436	-0.005868647034	-0.2574638315	0.024228965	0.013681786	0.002829575
0.839254906	0.041638132565	0.06905458348	0.922727402	0.851207824	0.023703927
0.874184985	-0.037072867099	-0.0617953076	0.887051014	0.801396183	0.028928312
0.028036290	-0.005603921383	-0.2299421428	0.028990466	0.016483049	0.003847639
0.447381223	-0.058351648326	-0.1969014614	0.452948792	0.306226976	0.050717576
0.276056455	-0.052514356092	-0.2926172614	0.331229427	0.175342154	0.044605629
0.041715645	-0.007072044257	-0.2121473965	0.044907680	0.026959670	0.005466018
0.039681588	-0.008683916262	-0.2771693038	0.038897423	0.027765612	0.003359619
0.042767484	-0.008464992314	-0.2522613517	0.043277875	0.030439631	0.003972300
0.470126822	-0.068750386965	-0.2229563793	0.469406304	0.310287863	0.052908669
0.654587484	-0.073262939425	-0.1685089618	0.661234163	0.482455274	0.048679075
0.048145010	-0.010905352791	-0.2996568876	0.054441696	0.025686507	0.007982953
0.083091829	-0.024362175035	-0.4377218428	0.076092622	0.040368753	0.012654194
0.102182425	-0.034261503602	-0.5257640277	0.110726995	0.045733584	0.023749945
0.631774292	-0.073143031673	-0.1745725867	0.615176672	0.484605282	0.050067006
0.096547462	-0.032417457495	-0.5233667182	0.079138570	0.043078690	0.011639620
0.018586828	-0.003964790646	-0.2154003966	0.018043258	0.011040277	0.002078512
0.565119638	-0.065748457371	-0.1751447639	0.561903686	0.436874033	0.053624353
0.925887050	-0.014628719496	-0.0227286649	0.926573005	0.896237392	0.010333978
0.443014640	-0.098693536163	-0.3545002770	0.516977552	0.180963774	0.094705848
0.103143837	0.0464596991905	0.49633461195	0.209007889	0.096402738	0.039733587
0.029529877	-0.005826613432	-0.2300551634	0.032409566	0.017228014	0.004818124
0.030581349	-0.006670773205	-0.2590815402	0.029666066	0.019292974	0.003852022

0.114531527	-0.025866265132	-0.3358968919	0.108625161	0.075735806	0.010306282
0.068926679	-0.017468090285	-0.3608984045	0.066368006	0.031535911	0.010617345
0.030016796	-0.006286632134	-0.2465663284	0.028094931	0.019457672	0.002663327
0.123983347	-0.022188444711	-0.2611992913	0.117467404	0.085045242	0.010402506
0.824973234	-0.069454806617	-0.1252928446	0.821971995	0.705489162	0.040077017
0.046606603	-0.011852974644	-0.3389656914	0.042386542	0.026003530	0.005904442
0.036699878	-0.007122201011	-0.2387318429	0.034951523	0.023183909	0.003533220
0.148291256	-0.039897597284	-0.4189897514	0.148708341	0.072416758	0.026721039
0.030139898	-0.006103601018	-0.2379628643	0.031305940	0.017756768	0.004940168
0.910324703	-0.019707214350	-0.0312284603	0.906495777	0.870045367	0.013626783
0.029969411	-0.008948074514	-0.3656354679	0.028842873	0.015975083	0.004686789
0.045539714	-0.013474577051	-0.4008947020	0.052838515	0.024525814	0.009440412
0.026408193	-0.005724194192	-0.2467766259	0.026026778	0.017053052	0.003066484
0.874952559	-0.038840754323	-0.0647518037	0.891150784	0.755624260	0.040165526
0.696275360	-0.076349422249	-0.1650485490	0.697788893	0.524456681	0.061363367
0.024042595	-0.004949195552	-0.2266491348	0.022528269	0.012987759	0.002937221
0.112627984	-0.029045420493	-0.3899765854	0.117027667	0.058028261	0.017267250
0.055990810	-0.012051221487	-0.2909206083	0.054481423	0.029031952	0.008743427
0.790872546	0.0705550245229	0.1218080935	0.903833531	0.776578938	0.045244604
0.030704671	-0.007706674906	-0.3028159380	0.028599588	0.019998528	0.003602541
0.163078087	-0.036281488794	-0.3393907250	0.177752093	0.093436214	0.027953075
0.035497991	-0.010302279793	-0.3704031975	0.034542736	0.016875237	0.005494043
0.045698039	-0.012163342877	-0.3554608785	0.041463182	0.022387432	0.006318213
0.539175817	-0.063509263063	-0.1773019609	0.562248247	0.393049873	0.051125290
0.062862771	-0.010649104974	-0.2279503376	0.056830998	0.046698546	0.004371721
0.356372006	-0.046417827904	-0.1954439671	0.340444745	0.285684876	0.020922558
0.052240538	-0.011294575838	-0.2888867221	0.050379691	0.031118553	0.005524977
0.046007219	-0.009992892054	-0.2835296657	0.043397030	0.026444716	0.004575166
0.561324404	-0.068783759865	-0.1850698731	0.560565110	0.405491375	0.050077901
0.021112676	-0.004661680887	-0.2341804796	0.023995349	0.012698854	0.003325529
0.509161159	-0.060376776201	-0.1783661696	0.523109920	0.385157433	0.048798973
0.595211045	-0.052468164288	-0.1308297782	0.593410768	0.496873587	0.037209910
0.048143637	-0.011665508804	-0.3230693465	0.047053991	0.028940808	0.006097529
0.044524616	-0.010695056571	-0.3150035269	0.044954188	0.025232877	0.006573751
0.040887584	-0.008020242298	-0.2474347408	0.040011858	0.026244717	0.005665165
0.026003210	-0.005567685964	-0.2423693313	0.025138354	0.016661539	0.002924769
0.078603008	-0.019942529830	-0.3678757691	0.067901335	0.041578645	0.008461384
0.046845330	-0.011511916591	-0.3264670364	0.042838921	0.021334183	0.006701152
0.040695322	-0.012264392249	-0.3995847448	0.038083883	0.022173648	0.005459566
0.050739393	-0.014485057313	-0.3930438830	0.049754300	0.026451036	0.008616272
0.067785140	-0.012822802680	-0.2598910293	0.059766795	0.049765281	0.003133853
0.081297634	-0.027171372087	-0.5096621602	0.083749383	0.038637729	0.016333903
0.044497297	-0.009462985364	-0.2751600622	0.046094301	0.028649001	0.006510700
0.272478572	-0.034224758918	-0.1863255558	0.262847470	0.207240524	0.017844922

0.059474591	-0.011278986787	-0.2555752522	0.059450050	0.034177424	0.007896377
0.411133299	-0.080962837568	-0.3080190831	0.392275218	0.256666084	0.043922842
0.531799925	-0.082512132016	-0.2383617536	0.560989614	0.382026646	0.051484424
0.511810450	-0.057730091841	-0.1691511985	0.520580655	0.380551508	0.042867701
0.069771120	-0.016081376694	-0.3248054762	0.065847116	0.036671108	0.012668038
0.060269982	-0.012156781282	-0.2740426082	0.059900848	0.036286225	0.007909003
0.062064692	-0.017130695902	-0.3915933400	0.055717237	0.035959119	0.007862321
0.512480402	-0.051445491940	-0.1495429361	0.503212084	0.429170679	0.027315643
0.633762305	-0.069074026574	-0.1637484796	0.644808716	0.478700973	0.047615982
0.959134388	0.0163921579535	0.02419799667	0.980001670	0.970566204	0.003016580
0.857008870	-0.032736184491	-0.0555277414	0.873817217	0.796798646	0.022446650
0.042728027	-0.011442597371	-0.35293729091	0.041491763	0.021117942	0.006747759
0.064898829	-0.014754463883	-0.3165135807	0.060456986	0.040667722	0.006927668
0.031962462	-0.006109866146	-0.2270217442	0.031031571	0.019654965	0.004190137
0.519798929	-0.074903132598	-0.2199088237	0.554195709	0.372708115	0.064884381
0.253477531	-0.081695090550	-0.5354670867	0.327586772	0.076346696	0.075246984
0.282631495	-0.050836307496	-0.2752995195	0.279861447	0.150826773	0.038309012
0.341297487	-0.040341924734	-0.1759848324	0.354258051	0.266944794	0.029288309
0.179853902	-0.050744176286	-0.4486663611	0.205535035	0.074156985	0.046858950
0.062307943	-0.014479158143	-0.3223663434	0.063784471	0.028315492	0.012899848
0.032599806	-0.009494996891	-0.3638060428	0.033184921	0.016063763	0.005402631
0.030835738	-0.007272820833	-0.2829641782	0.031953396	0.012710278	0.005552943
0.285538124	-0.052910153394	-0.2845983705	0.278673735	0.169850052	0.038799078
0.252911947	-0.045856220805	-0.2765142168	0.253294171	0.168630843	0.026200082
0.226011689	-0.057095318388	-0.3995729153	0.249412007	0.121997502	0.049750719
0.428159174	-0.047890404082	-0.1669870454	0.420493249	0.314040532	0.031699571
0.542417015	-0.0532335135041	-0.14618750411	0.547299757	0.430328825	0.036817512
0.227470152	-0.046081792361	-0.3112431025	0.241062689	0.129569450	0.038865253
0.739227253	-0.060297055536	-0.1210455619	0.750580725	0.622490933	0.041010088
0.043359305	-0.0074102720621	-0.2157053739	0.040572369	0.030227254	0.003970151
0.044642382	-0.009015209254	-0.2601271140	0.042811482	0.026056649	0.005745876
0.034798422	-0.0092891439391	-0.3352518849	0.028958173	0.018875460	0.003304115
0.021898701	-0.003813285245	-0.1836765112	0.022257263	0.015257385	0.002254214
0.035156518	-0.006628165056	-0.2290137283	0.041348995	0.021805103	0.006701135
0.893815917	-0.029811629758	-0.0483886077	0.882467645	0.849362185	0.010413155
0.044248816	-0.007665058263	-0.2197646276	0.048017519	0.026473455	0.006560545
0.624284661	-0.064393650369	-0.15444441751	0.620103330	0.469496784	0.046770067
0.052214828	-0.014221417703	-0.37442213381	0.045183864	0.027458472	0.006694735
0.079916196	-0.018844554992	-0.3393069912	0.078715214	0.046555425	0.011396593
0.024949905	-0.0045362263471	-0.2005682064	0.025484063	0.017016713	0.002725215
0.352251038	-0.057146576259	-0.2476865457	0.378809670	0.208350981	0.049621205
0.331868765	-0.0587417552341	-0.2719932295	0.350866324	0.211220062	0.045116227
0.842691315	-0.036027303405	-0.0622809091	0.857841934	0.763136791	0.035300336
0.036109463	-0.009259395293	-0.32339558871	0.032415579	0.019598438	0.004352867

0.077108718	-0.027634338870	-0.5505488011	0.068942301	0.033486000	0.010803645
0.059039471	-0.0155836047131	-0.3690731259	0.054277790	0.032949391	0.007106998
0.025035967	-0.0054295453011	-0.2429265765	0.025201854	0.015599930	0.002835603
0.683710121	-0.079497318137	-0.1755943483	0.706571488	0.513916327	0.059764004
0.063523389	-0.0250637385521	-0.6014192651	0.062930324	0.021912400	0.011551175
0.047061126	-0.008697897382	-0.2385976566	0.048561875	0.028209382	0.006588422
0.919706222	0.0249818600262	0.03825453850	0.961806752	0.907434494	0.016822001
0.053326347	-0.011039756343	-0.2763648532	0.049478404	0.036836532	0.004466060
0.115244658	-0.0402128588301	-0.5586746959	0.107291452	0.050251454	0.016947283
0.243703605	-0.043444330858	-0.2709745828	0.235370971	0.149404835	0.023502620
0.071579023	-0.017167964972	-0.3408898376	0.073970198	0.035368989	0.011511502
0.539644447	-0.046527437586	-0.1276047072	0.533354831	0.468776429	0.019758283
0.222295906	-0.042094225548	-0.28843373471	0.218117109	0.128337082	0.027424289
0.063087154	-0.0212768300611	-0.4963782444	0.057271607	0.029873058	0.008781249
0.728003922	-0.059510128576	-0.12129286471	0.705649963	0.619246823	0.031619646
0.062957615	-0.013339276958	-0.2913024086	0.060237115	0.030105603	0.008802559
0.944786675	0.0226690489954	0.03385292153	0.981501988	0.931528746	0.015242400
0.471396223	-0.114104344722	-0.39029782681	0.527637048	0.211852029	0.114136546
0.038414183	-0.009820233386	-0.3270550509	0.036643187	0.024010443	0.004171828
0.070450026	-0.013744265006	-0.2702815200	0.082208297	0.046089898	0.012736715
0.056046966	-0.018095470854	-0.4619164832	0.056480085	0.026834313	0.008526130
0.034141732	-0.007544457334	-0.2704070184	0.039364365	0.019363511	0.006084149
0.139754186	-0.021960313788	-0.2287776692	0.135451334	0.092638828	0.015115199
0.107871588	-0.017997343798	-0.2390314228	0.102168321	0.074170584	0.009115528
0.449879111	-0.101743086793	-0.3607470235	0.508395893	0.214446832	0.104439892
0.048165203	-0.010731662819	-0.2942487751	0.045327672	0.024837587	0.006556928
0.171321843	-0.055931908938	-0.5321311906	0.187524620	0.068469927	0.037411361
0.803405267	-0.051140430897	-0.0936820076	0.792083237	0.671372935	0.045723569
0.033703466	-0.006856608537	-0.2462061175	0.030265615	0.021451114	0.002864113
0.191855771	-0.031931723769	-0.2484347991	0.199540107	0.109531094	0.026419162
0.577235897	-0.0556686405041	-0.1436879206	0.577053073	0.472001352	0.032848667
0.201235516	-0.041067030354	-0.3118885389	0.241317279	0.111944408	0.035159285
0.792596924	-0.050618985994	-0.0939852910	0.784630750	0.664382058	0.035714846
0.627476718	-0.074807467991	-0.1800855305	0.647253292	0.499025821	0.042774305
0.553950834	-0.062031178777	-0.1681129486	0.579472022	0.418666254	0.049350924
0.141194086	-0.034006212719	-0.3675784218	0.151030856	0.086619329	0.018013948
0.042014539	-0.009862370371	-0.3033080775	0.042174059	0.021267129	0.005650169
0.048769942	-0.010977629933	-0.2982999094	0.051452573	0.020376385	0.010017658
0.851634690	-0.032075976448	-0.0547322406	0.856736204	0.766312602	0.027109488
0.555494298	-0.054321491501	-0.1457013876	0.580066345	0.433504357	0.042696189
0.054380013	-0.012203645822	-0.3032163662	0.054690858	0.035638019	0.006543862
0.564443866	-0.042707608181	-0.1114550773	0.566876462	0.486414315	0.026524824
0.070094770	-0.023142226282	-0.4919477578	0.072568302	0.034877260	0.011151115
0.152447471	-0.1150966241351	###########	0.053572008	0.019968700	0.011726140

0.030891518	-0.006524764557	-0.2507880502	0.029831467	0.018066713	0.004533285
0.361004263	-0.041085937202	-0.1693268471	0.347019789	0.283523457	0.020568174
0.050497127	-0.010830983688	-0.2846039060	0.051450691	0.031483546	0.007530592
0.595409726	-0.060674457873	-0.1523565768	0.623626334	0.471092479	0.051255274
0.040017625	-0.006768939573	-0.2097802707	0.044828146	0.027997497	0.005028310
0.341400223	-0.037214486778	-0.1614974215	0.341515077	0.281396770	0.020877898
0.067301066	-0.011194924363	-0.2257039942	0.065273704	0.048445424	0.005479011
0.844690426	-0.060134040988	-0.1052523589	0.872282324	0.676666171	0.064263889
0.026264836	-0.005666000394	-0.2450945639	0.023690228	0.015124855	0.002908831
0.591207300	-0.079411208195	-0.2043763880	0.571732309	0.446593738	0.043829475
0.578268528	-0.082191802191	-0.2171187276	0.554894355	0.377398762	0.051687737
0.024188300	-0.0044916103201	-0.2032005616	0.022948674	0.015667878	0.002723130
0.028739311	-0.006343163048	-0.2579760091	0.033620208	0.018407923	0.004625022
0.030051315	-0.006321699946	-0.2478337526	0.029535240	0.016779923	0.003818780
0.532113141	-0.071961425232	-0.2054675957	0.556577429	0.344654404	0.070614470
0.035843356	-0.007868222917	-0.2716576395	0.033288482	0.022869349	0.003006832
0.775907514	-0.049250548549	-0.0933665806	0.785230474	0.683879713	0.038828828
0.493171680	-0.056214757409	-0.1709149331	0.488650018	0.394984491	0.031445542
0.685244087	-0.106072216438	-0.2388310175	0.687956442	0.467060259	0.065445625
0.027198429	-0.005495960629	-0.2306465167	0.029584783	0.017972169	0.004069857
0.022068358	-0.004749068257	-0.2312305039	0.021702794	0.012897689	0.002597393
0.299328417	-0.036920967809	-0.1833732192	0.291718012	0.222528765	0.024397442
0.460004063	-0.062503449169	-0.2058709955	0.511677025	0.322149451	0.050863421
0.040762918	-0.010006728136	-0.3167558104	0.043188115	0.022919566	0.006884368
0.207521568	-0.049392606464	-0.3715901938	0.217972680	0.078611702	0.044282200
0.815176854	-0.045223605656	-0.0813157053	0.831022022	0.709764868	0.041385171
0.046150325	-0.016734864804	-0.5105327532	0.041046193	0.022616734	0.006267467
0.158732494	-0.032598820097	-0.3097138545	0.172111365	0.097640677	0.023912508
0.265422846	-0.048625243767	-0.28024284611	0.260935270	0.162140811	0.036756721
0.023804634	-0.004953923044	-0.2286141489	0.022245831	0.015988649	0.001789144
0.017109143	-0.002664859735	-0.1492823990	0.017372404	0.012585299	0.001424518
0.058368453	-0.0142967945261	-0.3384581698	0.059096168	0.026377662	0.009378270
0.030872803	-0.006036473382	-0.2305485532	0.030631342	0.017580110	0.003849715
0.029532824	-0.005964448873	-0.2359483101	0.029413852	0.019378749	0.003981842
0.058713333	-0.011341716167	-0.2602528867	0.065854173	0.036217346	0.008223414
0.036594772	-0.006972599525	-0.2338601054	0.038538497	0.022961500	0.005210520
0.850071015	-0.030759790682	-0.0525421676	0.868399890	0.794756407	0.022309321
0.020080977	-0.004886200300	-0.2557267545	0.021886061	0.013177199	0.002859956
0.789468151	0.0438635668352	0.07705965422	0.879813469	0.785312440	0.028189990
0.047811490	-0.010984193766	-0.3040065132	0.049519360	0.026319439	0.006650749
0.031145401	-0.005292391134	-0.1986369441	0.030414527	0.020414221	0.003150091
0.043475705	-0.010411793869	-0.3124042425	0.045301642	0.023510199	0.006034341
0.030793831	-0.005171022295	-0.1955497257	0.032106200	0.019244574	0.004174229
0.041835120	-0.008055434000	-0.2436682943	0.043643584	0.026826785	0.005654732

0.727405291	-0.074414283717	-0.1534684660	0.750068882	0.598060309	0.052307573
0.572591640	-0.052988912886	-0.1375744480	0.587762094	0.471776652	0.034024879
0.522648061	-0.071773762522	-0.2088092936	0.519030833	0.396416373	0.047373027
0.024016866	-0.004814163171	-0.2201483228	0.023937871	0.015456373	0.002889343
0.577995494	-0.055007587184	-0.1417022995	0.591368877	0.458138476	0.046241571
0.062625551	-0.0119124836561	-0.25847013911	0.072132617	0.041406929	0.009811598
0.712480109	-0.054718810765	-0.1136253875	0.709849786	0.587752687	0.044919510
0.068113312	-0.014860032562	-0.3044281522	0.067026728	0.035510295	0.008374475
0.631416192	-0.045537918462	-0.1062431277	0.646572161	0.541578254	0.032075525
0.811266485	-0.043734133424	-0.0789477202	0.810947802	0.709282207	0.038742542
0.085941152	-0.015024474846	-0.2457126847	0.099880392	0.058163558	0.013156866
0.449206944	-0.051631272716	-0.1720765201	0.455529489	0.349768447	0.034398803
0.019037751	-0.004061038191	-0.2173460999	0.020167997	0.010111241	0.002857770
0.178139510	-0.054624250111	-0.49479820801	0.197208470	0.073513290	0.040591851
0.038065916	-0.007856964314	-0.2574974995	0.039626672	0.025292624	0.005222620
0.679679425	-0.069093103947	-0.1522940304	0.673855493	0.552858509	0.043005757
0.168103800	-0.064346495745	-0.6467591110	0.154260346	0.070604023	0.029787275
0.045808824	-0.010181370506	-0.2905911043	0.041181795	0.028020459	0.004432448
0.037373184	-0.008496788455	-0.2851761750	0.043038153	0.019825827	0.008627351
0.704025618	-0.0656182452131	-0.1390753411	0.685849332	0.565919017	0.039279081
0.075820398	-0.015408332034	-0.28549791691	0.076977286	0.035322922	0.013395277
0.051909803	-0.008518299509	-0.2135576927	0.050061824	0.036265164	0.004823817
0.669607842	-0.065823507599	-0.1469706754	0.653676359	0.469707740	0.054391144
0.791924448	-0.339551410037	-0.7944090477	0.900453841	0.025042569	0.423143687
0.877070803	-0.020051220872	-0.0329846822	0.873646410	0.835559688	0.011458592
0.082568625	-0.023530879828	-0.4231379420	0.099779300	0.039364653	0.016919267
0.700552026	-0.058287718048	-0.1234836001	0.724177924	0.590479916	0.045381641
0.045480735	-0.009909323581	-0.2838578464	0.051919353	0.026211270	0.008511399
0.055067095	-0.016194907662	-0.4129144663	0.054834430	0.029478853	0.008511863
0.068591564	-0.023422394092	-0.5105121834	0.059252950	0.035136361	0.007610491
0.034210320	-0.007175219575	-0.2554899219	0.033272135	0.019303993	0.004388674
0.033136203	-0.006851296381	-0.24952963101	0.031802755	0.021701289	0.003772741
0.050224522	-0.012705927142	-0.3418588568	0.050828929	0.025608876	0.006953317
0.066643952	0.0226740051078	0.37388263357	0.111639231	0.057380061	0.018008133
0.020274489	-0.004404285113	-0.2268111842	0.019520049	0.011903865	0.002676391
0.491542158	-0.087790364669	-0.2776054088	0.541306875	0.239843392	0.089910231
0.191199461	-0.132366310648	############	0.074597279	0.043765897	0.009674731
0.060174533	-0.012665577826	-0.2871609302	0.060515966	0.040567191	0.006125205
0.056321738	-0.017809450875	-0.4511316093	0.054404002	0.024867614	0.009196743
0.224648237	-0.037748445083	-0.2530380335	0.238512964	0.136194500	0.032517872
0.078500148	-0.013263581274	-0.2342458495	0.073603390	0.057952555	0.004630217
0.200408289	-0.051806636647	-0.4077837381	0.178753354	0.116912982	0.021286635
0.756350976	-0.056377193275	-0.1102395227	0.760188353	0.623108524	0.049517079
0.225342856	-0.053151815412	-0.3693120440	0.227599494	0.102023261	0.035132583

0.780288585	-0.045963965033	-0.08644761317	0.805444070	0.687182916	0.033832492
0.033144156	-0.006328021430	-0.2288269902	0.031562761	0.021729183	0.002840792
0.521754587	-0.050036814965	-0.1425724225	0.527985896	0.444564723	0.029657115
0.578511385	-0.058667269939	-0.1515023567	0.598061661	0.445182960	0.041800503
0.063771357	-0.013100478987	-0.2820565805	0.062306668	0.041443013	0.006353039
0.033274700	-0.007386951452	-0.2700323986	0.033319430	0.021509910	0.003894772
0.816024181	-0.061567595901	-0.1117494449	0.846252752	0.633801644	0.063984530
0.050533196	-0.011970475478	-0.3178772747	0.047124703	0.026776841	0.006928121
0.590678710	-0.062834232882	-0.1594044669	0.636829551	0.442007598	0.059566296
0.025012888	-0.006771677073	-0.3100841124	0.026198024	0.013561979	0.004918199
0.108309063	-0.026985871238	-0.3735074053	0.117592901	0.055183744	0.018972038
0.538263279	-0.051186719590	-0.1414007776	0.536564300	0.428771766	0.038815908
0.371075743	-0.039122671466	-0.1562794293	0.353829150	0.308389571	0.015748811
0.230774014	-0.034731631234	-0.2247385655	0.226530677	0.161150247	0.022826456
0.087622336	-0.030178761570	-0.5335302704	0.087147879	0.040686784	0.013196897
0.655401572	-0.048004315288	-0.1080262018	0.653041738	0.555384716	0.036078834
0.166427484	-0.033111332346	-0.2998741116	0.177081089	0.096962782	0.026368941
0.549193741	-0.051501728810	-0.1393946513	0.532401172	0.452057886	0.029188928
0.426748031	-0.041570933636	-0.1443018613	0.437672868	0.350499234	0.028862806
0.031433922	-0.007178927582	-0.2744980359	0.032910282	0.019025638	0.004699567
0.662489740	-0.054855780806	-0.1227601787	0.704591770	0.528182236	0.047740939
0.152351697	-0.029823153666	-0.2928193492	0.168530002	0.091749912	0.025370036
0.080863358	-0.017827339478	-0.3150904995	0.071883944	0.045931323	0.007946635
0.034229038	-0.010050232008	-0.3718918613	0.032245440	0.017255270	0.004425057
0.919549518	0.022265031111	0.03414878622	0.967700934	0.914582647	0.018707453
0.050449048	-0.010769853934	-0.2830778488	0.051650471	0.029743691	0.006518390
0.032757927	-0.007154732850	-0.2641852276	0.032840822	0.016341156	0.005569638
0.927420707	-0.024303658748	-0.0378968588	0.934951144	0.836319741	0.029705983
0.861795053	0.030202307715	0.04913419494	0.927094314	0.855575620	0.026006036
0.905522373	-0.031180558217	-0.0499910359	0.930195933	0.845409043	0.024134645
0.829589417	-0.034168899408	-0.0599417570	0.828605746	0.748278699	0.026657130
0.053531328	-0.009525864967	-0.2343628206	0.054276386	0.032886937	0.006578741
0.030154513	-0.005744075458	-0.2227159041	0.033564671	0.018395121	0.005075050
0.546861416	-0.056610386039	-0.1546661015	0.542271193	0.412188740	0.047762730
0.557492480	-0.058022867817	-0.1556051767	0.538795240	0.409803706	0.038131567
0.049097670	-0.008938123735	-0.2365769503	0.046155168	0.029614274	0.004874047
0.450081685	-0.050821762505	-0.1688726310	0.468185631	0.336691473	0.043335970
0.037780396	-0.007616502504	-0.2505196761	0.033783381	0.024778597	0.002900163
0.082171328	-0.015287326288	-0.2616346068	0.077056940	0.056098045	0.007924385
0.483528367	-0.059742904070	-0.1861513040	0.482658087	0.351134191	0.038607164
0.033994802	-0.007161308450	-0.2563148373	0.036226589	0.020800823	0.005018271
0.069383596	-0.011728830556	-0.2306493572	0.068047507	0.047013600	0.006845607
0.333907239	-0.049830184537	-0.2258252619	0.362809588	0.230819862	0.040361931
0.907072794	-0.024283729083	-0.0387168975	0.920382987	0.863235498	0.016863424

0.069854357	-0.018362227737	-0.3769693589	0.062458983	0.033724805	0.010250371
0.322822325	-0.051768365984	-0.2439050717	0.312543818	0.230771266	0.027257032
0.035342621	-0.006468382880	-0.2220533846	0.042325709	0.022405038	0.006449701
0.495161283	-0.060491359463	-0.1840092435	0.501114535	0.344763521	0.054538547
0.041455957	-0.0089314100491	-0.2750422355	0.039206672	0.024462825	0.005195975
0.348286989	-0.040220529619	-0.1717874082	0.355627881	0.267509499	0.033541522
0.373030642	-0.069317064618	-0.2880118405	0.380659685	0.214685411	0.052445825
0.041647350	-0.009202428037	-0.2831023614	0.038559955	0.025092554	0.005260452
0.131401028	-0.021208839929	-0.23444947781	0.132604209	0.099877638	0.010658484
0.042905949	-0.011447380775	-0.3517596577	0.047793872	0.024461738	0.006790919
0.034784356	-0.005830670919	-0.2012350411	0.033823045	0.022212771	0.003834804
0.047271022	-0.009829443925	-0.2716533458	0.051341095	0.028553978	0.006669702
0.115950070	-0.029571396112	-0.3860660665	0.123825434	0.074301973	0.015321147
0.856657940	0.0303135439905	0.04959942997	0.922517959	0.855898984	0.020341517
0.060358014	-0.0132322448491	-0.3005730733	0.058591860	0.037884248	0.007278877
0.040349148	-0.008320089023	-0.2605802063	0.043516207	0.021949174	0.006239864
0.954807130	0.0104540962540	0.01554813132	0.971499277	0.950158774	0.007722989
0.062324196	-0.010061652393	-0.2161138610	0.059111402	0.043184300	0.006002234
0.045940483	-0.011309154147	-0.3258359456	0.056825991	0.026620174	0.009173204
0.917856879	-0.058523138142	-0.0939921481	0.930200365	0.694615925	0.078058669
0.038712513	-0.0087526454301	-0.2857406065	0.036576070	0.019485161	0.006509734
0.056245191	-0.008260164731	-0.1921355313	0.054153860	0.040825022	0.004379274
0.891017290	0.0325650833478	0.05122253144	0.946615861	0.886072041	0.019029250
0.046190133	-0.011247016590	-0.3222166459	0.042913539	0.017987393	0.008151007
0.650062244	-0.058511353048	-0.1339152832	0.672703847	0.497512016	0.056261439
0.466047170	-0.043203527016	-0.1372585613	0.472024609	0.365820035	0.032309414
0.034841846	-0.008367593807	-0.2979672675	0.035758858	0.017772771	0.005436056
0.026557862	-0.005035865218	-0.2138227697	0.026001945	0.015149128	0.003237980
0.486887590	-0.067391744039	-0.21027533211	0.523006504	0.317265312	0.063119750
0.088257310	-0.024773405824	-0.4191364627	0.101469408	0.048280063	0.015761493
0.067783688	-0.0134544351341	-0.2739927059	0.063382809	0.041994771	0.006679472
0.059240912	-0.012752626456	-0.2936730124	0.066528321	0.029306484	0.010635436
0.055537953	-0.012075251844	-0.29379787831	0.055228747	0.038608969	0.005018685
0.497876426	-0.053765229955	-0.1614319034	0.498339572	0.376857113	0.036042068
0.026923600	-0.0054880325841	-0.2321454162	0.030334216	0.014367370	0.005554129
0.026845636	-0.0051169898071	-0.2157077899	0.030819013	0.017754756	0.003897154
0.857055895	-0.034313436435	-0.0582546164	0.855417188	0.786805999	0.020115331
0.114490930	0.0922217244513	0.79974276017	0.381471222	0.088032196	0.108765752
0.031360601	-0.006167484517	-0.2329638711	0.033705024	0.020940725	0.003835419
0.098589304	-0.031019996610	-0.4853241787	0.096798099	0.038675668	0.017988498
0.070897312	-0.014236269813	-0.2792478910	0.063961337	0.042408316	0.006954599
0.217195678	-0.053120274348	-0.3842230202	0.236990470	0.104647245	0.043440904
0.484929584	0.1285951397995	0.33322348536	0.840566453	0.481336008	0.122710875
0.847010893	-0.033671226206	-0.0578258079	0.850659142	0.767499494	0.026470687

0.030391354	-0.007120666138	-0.2797948143	0.029113356	0.016266833	0.004633720
0.036262132	-0.008129872014	-0.2788197106	0.036661653	0.019169004	0.005743931
0.593193600	-0.048783085898	-0.1216665036	0.591324155	0.478736448	0.030532829
0.784875420	-0.043248938637	-0.0807128692	0.826498838	0.702089696	0.038955776
0.841795618	-0.038876949417	-0.0673962881	0.833717185	0.723189275	0.033653142
0.023144483	-0.004649759852	-0.2180739736	0.023266732	0.014269006	0.003142037
0.699154137	0.134414576197	0.2504063524	0.927757563	0.656728715	0.099664194
0.061654525	-0.018403494592	-0.4282483710	0.060572465	0.031804161	0.010349194
0.069480557	-0.019565343751	-0.4076796113	0.066377796	0.037888318	0.010091567
0.821194223	-0.043651696414	-0.0778277973	0.824860020	0.709939968	0.038578508
0.148255454	-0.019566274403	-0.1903999852	0.152014371	0.117485519	0.010400076
0.557439285	-0.053615869460	-0.1431933976	0.568130992	0.425580485	0.048177435
0.047058789	-0.009751199970	-0.2703774871	0.043281261	0.027582861	0.005629302
0.545980525	-0.055405580542	-0.1514482677	0.550357907	0.404448176	0.050370264
0.730728767	-0.060587899360	-0.1231117851	0.747065452	0.597233440	0.046415265
0.453851532	-0.048152503750	-0.1581237306	0.441853764	0.372105410	0.025487864
0.856027596	-0.026138857834	-0.0442147735	0.859076274	0.786391045	0.021846745
0.053181455	-0.011018676125	-0.2764804312	0.053382102	0.030978669	0.007866801
0.870975865	-0.027363022836	-0.0455206150	0.877628401	0.808594450	0.020423207
0.309305620	-0.049638013507	-0.2437557150	0.310472155	0.201549917	0.035316595
0.092170325	-0.034289984550	-0.5899105226	0.080687805	0.039568148	0.013776240
0.142708311	-0.028580893556	-0.2989567660	0.144159186	0.078574573	0.021035593
0.033875914	-0.006707222674	-0.2393412858	0.037311101	0.020445284	0.005144508
0.024104989	-0.003592101631	-0.1605640876	0.024465249	0.016896468	0.002594027
0.901930159	0.020657894276	0.0323166099	0.942417492	0.896388247	0.015475265
0.057054578	-0.012706030267	-0.3030943471	0.058089685	0.028274284	0.011624136
0.881996102	-0.028616981400	-0.0470432015	0.893939702	0.823257365	0.023740817
0.075121460	-0.011106896608	-0.2017137270	0.073701581	0.055087265	0.006332267
0.024396521	-0.004985420951	-0.2259019054	0.022731781	0.012852963	0.003286837
0.046342522	-0.012155912105	-0.3506148895	0.045308203	0.026049729	0.008071903
0.464122065	-0.081365234446	-0.2716221693	0.469461000	0.327125045	0.041749869
0.097548713	-0.034100761802	-0.5501961033	0.082313468	0.046989724	0.012936803
0.884884805	-0.027585557605	-0.0451721541	0.910324960	0.824426896	0.025586369
0.026837337	-0.004292984902	-0.1787615739	0.025837752	0.016656580	0.002937134
0.028863356	-0.005316174273	-0.2122189121	0.033350495	0.018798983	0.005663064
0.877280118	-0.025011536831	-0.0412523283	0.873407196	0.832155898	0.012966930
0.072908478	-0.011750985379	-0.2205039589	0.075457773	0.052468375	0.007716589
0.636932636	-0.068695359491	-0.1619538782	0.660874088	0.505260097	0.058554640
0.111729741	-0.041801249264	-0.6068999194	0.108233497	0.050270062	0.018681838
0.385260232	-0.073711421733	-0.2977650574	0.439385595	0.189154256	0.087114925
0.476841541	-0.071065396753	-0.2276452908	0.509547849	0.325315614	0.054688226
0.048148035	-0.008610194236	-0.2311994526	0.048471189	0.029695737	0.006675723
0.922633804	-0.017161681421	-0.0267947675	0.934478327	0.881987869	0.015747484
0.788492662	-0.047772234682	-0.0890034098	0.785881445	0.699846168	0.029710510

0.908879658	-0.028042286477	-0.0447138479	0.920233681	0.847691857	0.022333705
0.442329964	-0.046593161244	-0.1568313986	0.454514477	0.347444633	0.032743341
0.028943573	-0.005299570104	-0.2110358891	0.027909238	0.019269936	0.002688652
0.023499977	-0.004167700340	-0.1916710471	0.028902292	0.015982017	0.003935617
0.346082665	-0.065522289205	-0.2933742292	0.328902425	0.217510540	0.041966708
0.721106082	0.0598850954160	0.11358084959	0.880004824	0.691906788	0.060357589
0.411024428	-0.041874203394	-0.1511343636	0.418723112	0.341252562	0.025899526
0.372329656	-0.056819917149	-0.2321164015	0.356354924	0.268608594	0.031021150
0.844319100	-0.048168516568	-0.0837256875	0.845936940	0.750734754	0.034255818
0.028831110	-0.005510686858	-0.2208062213	0.027760354	0.019319556	0.003247590
0.595005826	-0.054383175114	-0.1358850760	0.597416681	0.498849930	0.031799221
0.202789668	-0.029935460712	-0.2187342803	0.194836674	0.153995311	0.014188482
0.060323818	-0.015001786651	-0.3461592667	0.064493862	0.030802412	0.009662370
0.022924000	-0.003393242217	-0.1569212423	0.021766306	0.018186692	0.001234697
0.030410067	-0.006189138636	-0.2398358391	0.032832549	0.019509058	0.004197009
0.901132202	-0.023658299268	-0.0379557031	0.896873749	0.826871386	0.022494527
0.055694250	-0.007709728791	-0.1800992709	0.054733172	0.041645595	0.004498528
0.759018167	-0.040217800719	-0.0774939959	0.786219046	0.657084178	0.038552771
0.144231564	-0.045608757537	-0.5057710007	0.145970005	0.068833544	0.027200263
0.847737917	-0.031210578126	-0.0534743575	0.845354254	0.771317583	0.025202272
0.224907623	-0.034836150026	-0.2315780539	0.234360562	0.161966542	0.023262766
0.082296725	-0.017322333407	-0.2998815470	0.083130668	0.050550761	0.011756738
0.577666807	-0.054377328456	-0.1400795482	0.591882428	0.484024087	0.034674469
0.882214113	-0.029829348922	-0.0490582788	0.913411683	0.825093399	0.026511280
0.103923403	-0.028266362624	-0.4114204114	0.113020890	0.060160347	0.015673121
0.039714438	-0.011022166583	-0.3616194696	0.036987326	0.019338670	0.005178473
0.065374114	-0.012747180597	-0.2672858977	0.075921334	0.042914240	0.009967807
0.022208400	-0.004156521530	-0.1993396088	0.020414104	0.013487093	0.002499868
0.237721073	-0.029890880737	-0.1855126678	0.232384309	0.177632758	0.017492092
0.657095381	-0.044623803817	-0.0998850884	0.653423298	0.562488988	0.030498361
0.519808470	-0.061725856008	-0.1787077278	0.504730582	0.402608618	0.036422831
0.059870592	-0.010520014177	-0.2354232871	0.063187456	0.037195469	0.008069034
0.811307152	-0.035282990073	-0.0633482004	0.812860045	0.739822823	0.024373694
0.032184364	-0.007060863402	-0.2642717077	0.032641047	0.021140140	0.004013952
0.820029196	-0.036881156841	-0.0655719170	0.821672191	0.721915432	0.032171939
0.069654479	-0.016802830898	-0.3418049098	0.069890470	0.041193419	0.009141509
0.051382105	-0.009310513855	-0.2373216291	0.052848165	0.033056443	0.006465264
0.121308648	0.0293840422916	0.29134236768	0.170199116	0.120221885	0.017940419
0.055238978	-0.008688910361	-0.2062054314	0.056126077	0.038518422	0.005516159
0.159835494	-0.036861887372	-0.3529980782	0.163026383	0.091681051	0.022848742
0.555553180	-0.044917134624	-0.1193874918	0.560273807	0.468430114	0.028921415
0.090464743	-0.030138906676	-0.5145625818	0.098898196	0.044914622	0.017264707
0.147915766	-0.023298833760	-0.2302953285	0.144151064	0.103312708	0.012864837
0.033929204	-0.008479676590	-0.3094139571	0.033442274	0.018638256	0.004545851

0.845500560	-0.039839716286	-0.0687994027	0.837435457	0.753079401	0.026367076
0.896773627	-0.021133219686	-0.0340213851	0.895789713	0.844945259	0.014262392
0.081837894	-0.021513622125	-0.3850668470	0.108151977	0.038201341	0.020875467
0.027640329	-0.008247344898	-0.3568074012	0.022824815	0.016771401	0.002257880
0.038824896	-0.006480587930	-0.2054489155	0.039418965	0.028318726	0.004230561
0.037188735	-0.006783948360	-0.2239162802	0.047263182	0.022634237	0.007050032
0.472500075	-0.070827567979	-0.2290320631	0.473630655	0.342952740	0.046699204
0.040791035	-0.006068772708	-0.1835807012	0.046446561	0.029604638	0.004955302
0.040588613	-0.008323981480	-0.2593618105	0.039077059	0.024426143	0.004881668
0.038608007	-0.008718995050	-0.2852025895	0.043349309	0.022023906	0.007783624
0.042831889	-0.011252933691	-0.3455554832	0.040284907	0.019507697	0.006559997
0.158458549	-0.034576745490	-0.3314337525	0.161074362	0.081653853	0.027215823
0.824314587	-0.045623515696	-0.0811311592	0.832538442	0.739095409	0.032105310
0.014615471	-0.003224370035	-0.2025546028	0.015153530	0.009200418	0.002010928
0.338629804	-0.049027886950	-0.2186490538	0.348706408	0.247198495	0.032014863
0.070570888	-0.014166597355	-0.2789821918	0.068952234	0.038582139	0.010287885
0.306995158	-0.044159581162	-0.2164290239	0.301080922	0.209735114	0.037974746
0.089300142	-0.024470367950	-0.4081833584	0.098430257	0.036626532	0.018311400
0.037239546	-0.007383001371	-0.2451784554	0.037125000	0.024237571	0.003472375
0.049093601	-0.011794739127	-0.3211964421	0.051845130	0.028595324	0.007410737
0.035765040	-0.006256278072	-0.2120732994	0.036067598	0.024791349	0.003973464
0.161940609	-0.044000076351	-0.4264369250	0.177226944	0.091055739	0.029137192
0.902020244	-0.025550765362	-0.0409948884	0.919073006	0.848594628	0.025309441
0.080799243	-0.029000721508	-0.5551079408	0.085754174	0.035139784	0.017181327
0.033688381	-0.007161222586	-0.2582801179	0.034548507	0.019680702	0.004733758
0.097298951	-0.023390497384	-0.3547478985	0.094830677	0.031189157	0.019470476
0.052920626	-0.014609564261	-0.3811794582	0.052534954	0.025021749	0.008291467
0.542917095	-0.084919423612	-0.2405618258	0.627559337	0.385508733	0.074634459
0.070879424	-0.024036044294	-0.5087803866	0.095552361	0.027276965	0.020457320
0.043535660	-0.008035779028	-0.2346374141	0.042797364	0.026746279	0.005485602
0.068836363	-0.013021351697	-0.2604445439	0.070809688	0.047010679	0.008450652
0.028767154	-0.007131129973	-0.2932665280	0.025607910	0.015199027	0.002984157
0.384130454	-0.058960187563	-0.2337790581	0.393326068	0.278735386	0.040421899
0.929687037	-0.015111746576	-0.0233895402	0.931414681	0.900419443	0.011128973
0.460047561	-0.073047020600	-0.2436657693	0.479438150	0.307861990	0.056714564
0.262083706	-0.052614773100	-0.3100338289	0.307689830	0.142997104	0.050974422
0.522111900	-0.082929284467	-0.2444275745	0.561194163	0.389738748	0.050084034
0.201915498	-0.030063794223	-0.2207266565	0.201307378	0.144142586	0.019947097
0.885534107	-0.025751909625	-0.0420941990	0.907326710	0.831478950	0.023775670
0.076134160	-0.027777647633	-0.5616918418	0.068943331	0.029938492	0.012229537
0.096219753	-0.014047477699	-0.2046473096	0.096304405	0.064206724	0.009525929
0.653736766	-0.034177570171	-0.0762691535	0.635265999	0.589657659	0.014297089
0.093564425	0.0662163318019	0.71314443318	0.264834258	0.068527017	0.077060798
0.025152140	-0.003923032987	-0.1707212002	0.024292883	0.017326836	0.001867742

0.558168874	-0.047276065618	-0.1253332794	0.606053759	0.456634746	0.047012558
0.030006851	-0.007080907119	-0.2810222711	0.030268397	0.018332716	0.004397520
0.036437783	-0.008194225461	-0.2800823594	0.037454484	0.021949429	0.004852532
0.033889098	-0.007582860198	-0.2736451786	0.030885995	0.022007145	0.003347402
0.075854151	-0.012541749753	-0.2278306268	0.071502822	0.048367925	0.008414154
0.049090527	-0.011689878848	-0.3180200767	0.045304695	0.033546232	0.003877874
0.019093946	-0.003373845308	-0.1778226901	0.018425739	0.012990851	0.001834050
0.741526211	-0.034518903295	-0.0678355989	0.734036070	0.665894984	0.020280571
0.037057444	-0.008348119093	-0.2817418087	0.043456450	0.021589504	0.006376024
0.027169766	-0.004814918590	-0.2001477325	0.025435040	0.017526708	0.002386824
0.227131711	-0.047231605951	-0.3204478997	0.224670245	0.153120251	0.022146181
0.908675749	0.0297205398834	0.04593427225	0.969544840	0.847895824	0.035133803
0.076186248	-0.029978756175	-0.6166952494	0.074095656	0.032609177	0.014651834
0.038558412	-0.008310764356	-0.2708167759	0.041966436	0.020524130	0.007114957
0.834538333	-0.044151816485	-0.0774660438	0.845398553	0.745470577	0.032044171
0.635972671	-0.046332962066	-0.1073772067	0.662860846	0.539848708	0.041131112
0.049806655	-0.010214566284	-0.2701960348	0.044493690	0.032250344	0.004496948
0.033710091	-0.007655602597	-0.2777875055	0.034977134	0.016496770	0.006407633
0.059135433	-0.012723181673	-0.2934167869	0.055122444	0.031810408	0.008369768
0.536802658	-0.065169514720	-0.1830855819	0.555587965	0.379290072	0.066777933
0.181054653	-0.050387255057	-0.4416973953	0.177987794	0.103974764	0.023110556
0.152054227	-0.051761695794	-0.5551415526	0.206401156	0.067725996	0.042825315
0.056484426	-0.020057810519	-0.5180643144	0.048432224	0.026461369	0.007069109
0.747836339	-0.077896506144	-0.1564792304	0.778667538	0.542054574	0.070396209
0.081382828	-0.027016717016	-0.5056217862	0.076404314	0.040357903	0.012181710
0.039099845	-0.007405112874	-0.2358533502	0.045744165	0.025296276	0.005955794
0.906939983	-0.028639105034	-0.0457788902	0.916532569	0.801752110	0.033686732
0.032854935	-0.006302643082	-0.2294994753	0.032340742	0.023003531	0.002849520
0.030905489	-0.004679687557	-0.1752768372	0.029451982	0.021315335	0.002592513
0.051036832	-0.014027255395	-0.3767253960	0.055019390	0.024109362	0.009431920
0.015862064	-0.003243145195	-0.1933074467	0.016075895	0.010032547	0.001879244
0.949415962	0.0086866088363	0.01300346752	0.966579966	0.951051454	0.005231266
0.724865532	-0.074156548590	-0.1534653246	0.734815665	0.571396900	0.054066945
0.042080801	-0.011014829247	-0.3428081967	0.040823923	0.020974268	0.005549535
0.071811860	-0.014358827812	-0.2784267034	0.072828258	0.047316463	0.008157048
0.028849340	-0.006155263771	-0.2488608267	0.025414555	0.018538692	0.002805937
0.047973020	-0.009212594554	-0.2496709273	0.048039254	0.029324192	0.006546635
0.231483488	-0.046812259582	-0.3108848684	0.246705750	0.134956328	0.046582589
0.024718345	-0.004661439274	-0.2080016812	0.024370007	0.017102079	0.002679065
0.017888341	-0.003239376138	-0.1781350484	0.020541628	0.011571780	0.002880127
0.587697166	-0.058329340924	-0.1481452391	0.568949649	0.489493421	0.028806363
0.053151304	-0.012270715502	-0.3116971619	0.049510104	0.028994125	0.006041421
0.193791627	-0.025629307932	-0.1939025348	0.190819697	0.155447846	0.011607862
0.037749774	-0.006769098730	-0.2205501431	0.038313797	0.024655584	0.004698027

0.036571058	-0.007801441553	-0.26450718971	0.034775344	0.023606684	0.003799310
0.282468548	-0.051184286492	-0.2775476709	0.271049487	0.197147518	0.026981868
0.201097247	-0.037142060397	-0.2791920684	0.225099699	0.130260263	0.030775359
0.098536642	-0.0267813782021	-0.4087986484	0.106532506	0.049146532	0.020067099
0.075392888	-0.027964314816	-0.57234719991	0.076935890	0.029901001	0.013970183
0.083496839	-0.0141506414231	-0.2367564998	0.083810421	0.057745627	0.007692468
0.020164185	-0.003779507231	-0.1931362358	0.019295654	0.012088471	0.002415893
0.067761797	-0.044638365809	############	0.030718125	0.014753108	0.004559499
0.500836810	-0.0793100302931	-0.2434123934	0.507156563	0.326612453	0.068157274
0.915584224	-0.017516268500	-0.0275640087	0.920455614	0.880763990	0.013316479
0.046143344	-0.008332504068	-0.2317772425	0.048465587	0.027531896	0.005818697
0.114304479	-0.048060340033	-0.7051799330	0.079441653	0.050617660	0.009474872
0.826391013	-0.055517751751	-0.09908914781	0.864278039	0.694510939	0.055812426
0.447312832	-0.0519462923671	-0.1739543921	0.482739790	0.359673196	0.035922673
0.485065561	-0.068889551611	-0.2161702101	0.506635780	0.268467714	0.081717192
0.881827346	-0.023361527662	-0.0382953700	0.883742296	0.822467076	0.019801071
0.031458211	-0.007194313933	-0.2749686072	0.036048486	0.018901803	0.005409267
0.204291374	-0.044038008695	-0.3318904612	0.201928572	0.099293309	0.034773854
0.051015814	-0.008486446525	-0.2160589915	0.047463997	0.036301661	0.004751439
0.263262488	-0.0422817894571	-0.2425151270	0.305667692	0.162046207	0.038999908
0.025334702	-0.004352514095	-0.1896467103	0.027923746	0.014801296	0.004361604
0.693077601	-0.050928570481	-0.1084822430	0.698841615	0.570131279	0.050485670
0.049846637	-0.009388118583	-0.2461723320	0.047341652	0.028973119	0.006412250
0.087465441	-0.029398810542	-0.5179430665	0.090011984	0.041235870	0.015245783
0.053152823	-0.015640155414	-0.41053501261	0.060109494	0.026293554	0.011440121
0.223543743	-0.037729774567	-0.2542091092	0.213023279	0.139714461	0.024312569
0.040994660	-0.0083664163221	-0.2585365633	0.046529035	0.024691393	0.006954636
0.033454259	-0.007913246340	-0.2900127933	0.031129689	0.017923148	0.004081661
0.045385058	-0.009143762032	-0.2603149802	0.044839575	0.027139490	0.005220206
0.911689846	0.0394318902778	0.0604378181	0.964884754	0.926418216	0.012137023
0.032050317	-0.006968407456	-0.2613893979	0.031371730	0.019690385	0.004126784
0.056523245	-0.010725768895	-0.2536586758	0.056981721	0.033945433	0.007476283
0.267043051	-0.0342462056671	-0.1903605032	0.260998142	0.195537666	0.019302079
0.154927216	-0.038593684331	-0.3845918765	0.173110667	0.090404298	0.025409629
0.040722545	-0.0098976895741	-0.3131793529	0.039172565	0.026118573	0.004668133
0.030685058	-0.0056582842501	-0.2160409340	0.028207166	0.023419591	0.001515253
0.183422293	-0.0378626719561	-0.3142864568	0.182068020	0.118273197	0.019704652
0.281465631	-0.040849777780	-0.2178480951	0.316817244	0.182372790	0.035945594
0.029835365	-0.007287062068	-0.2914674688	0.036069413	0.017380030	0.006085592
0.882153985	0.0311289929118	0.04948014907	0.951015063	0.886196593	0.019685026
0.487227289	-0.056862458686	-0.1752062239	0.494867426	0.369486176	0.039241651
0.872857584	0.0397046132380	0.06346544606	0.936035526	0.876323171	0.018931184
0.022403756	-0.005181199875	-0.2513585098	0.024509593	0.010994097	0.004257344
0.285034846	-0.049299439610	-0.2637796165	0.305418863	0.179322932	0.036851528

0.020253265	-0.004010481091	-0.2051700714	0.018304435	0.013352125	0.001606247
0.022873462	-0.004786487577	-0.2270221688	0.019773607	0.015286894	0.001394428
0.073059434	-0.017954956031	-0.3513872598	0.066227993	0.042795585	0.008135919
0.032925349	-0.006801880324	-0.2488934288	0.035858695	0.019571606	0.005473542
0.067852780	-0.010993251707	-0.2196153796	0.069511242	0.045391663	0.007422998
0.026678561	-0.004426020756	-0.1855242415	0.025899589	0.016659074	0.002830412
0.850984529	-0.040417986196	-0.0693669820	0.869630421	0.782076136	0.026790709
0.054426573	-0.009369730323	-0.2267339740	0.057474765	0.037394808	0.006316598
0.022486890	-0.003964759540	-0.1877758888	0.020345301	0.013192449	0.002175823
0.638208989	-0.035979829737	-0.0823872679	0.650181266	0.563662456	0.026547980
0.478381799	-0.052434552519	-0.1638558684	0.457685686	0.396463169	0.019351588
0.806990616	-0.040420848398	-0.0732039643	0.807176149	0.712486147	0.030647506
0.605464217	-0.054316915160	-0.1332954511	0.651967735	0.468801142	0.053782245
0.483908185	-0.046191211598	-0.1416558635	0.484451483	0.412852525	0.022655907
0.032083469	-0.006574922906	-0.2450873405	0.029851884	0.020781050	0.003065484
0.026056602	-0.004662167457	-0.1997546106	0.028472985	0.016246211	0.003558754
0.877054316	-0.035601768478	-0.0590963170	0.909942363	0.804943232	0.030287107
0.027008207	-0.003794231218	-0.1560548113	0.029303339	0.019563257	0.003383113
0.028059653	-0.005055516745	-0.2056155632	0.027324521	0.019945677	0.002334411
0.031016859	0.0093132100343	0.29520355873	0.055113802	0.025969677	0.010444560
0.079116193	-0.012487120800	-0.2177957644	0.079457513	0.052325973	0.009792682
0.768032707	-0.047499659368	-0.0908812658	0.766180304	0.680465364	0.026909645
0.035447963	-0.006550736201	-0.2245483288	0.038196517	0.023894497	0.004705432
0.050018928	-0.015941547770	-0.4453790715	0.049337697	0.021107849	0.008264149
0.033571503	-0.007439611101	-0.2701120818	0.033120731	0.016713350	0.005517266
0.490467919	-0.059244897443	-0.1817695242	0.511671530	0.388202704	0.041731747
0.084498738	-0.011655928856	-0.1899185853	0.086644724	0.063326458	0.008298351
0.904773739	-0.024161993548	-0.0386183116	0.903928022	0.854042667	0.017154985
0.063175184	-0.020434706915	-0.4724438438	0.062046249	0.032105903	0.009972514
0.872217687	-0.031694542355	-0.0527841889	0.874641820	0.800354969	0.026818962
0.067528587	-0.011621093918	-0.2342858752	0.074716373	0.045283577	0.008879739
0.036775649	-0.008367009823	-0.2843268193	0.038914790	0.019476454	0.007092579
0.031063081	-0.006011936687	-0.2283803325	0.032843374	0.017174066	0.005376164
0.163379340	0.0328037963591	0.24999434261	0.207243853	0.182089680	0.007963278
0.448999275	-0.034527379545	-0.1128228395	0.446829547	0.393619323	0.016102416
0.054050538	-0.012060248336	-0.3009684917	0.058439233	0.033003924	0.007635048
0.211451377	-0.036724146899	-0.2615934221	0.233949603	0.153071067	0.023975983
0.325564721	-0.066957344841	-0.3210921247	0.338771485	0.192664168	0.051429472
0.041875259	-0.008280951695	-0.2509068773	0.047798150	0.024694720	0.007925767
0.405740161	-0.039285362919	-0.1432054734	0.409907526	0.320864847	0.027623083
0.139658277	-0.026995855643	-0.2869787344	0.146817081	0.072997738	0.022206342
0.057566206	-0.010624838975	-0.2468247161	0.053114356	0.037211684	0.005928969
0.740349747	-0.052616409473	-0.1048874428	0.776455735	0.629389586	0.045399874
0.890800215	-0.023843770351	-0.0387019808	0.900913147	0.831921873	0.023499685

0.407380902	-0.091307853473	-0.3561693879	0.404251158	0.268665790	0.042713551
0.486694269	-0.048215608531	-0.1473187602	0.500233280	0.371820906	0.036632301
0.052681815	-0.010415199972	-0.2621572238	0.057463307	0.032552665	0.008094877
0.087077045	-0.019063473966	-0.3154050794	0.081392619	0.053140238	0.010638358
0.247811807	-0.033771307359	-0.2025587852	0.260103400	0.191001090	0.023629251
0.963715844	0.007495979556	0.0110638011	0.979510473	0.956750136	0.006793381
0.837615328	-0.049068034024	-0.0860318232	0.846016804	0.753671534	0.025166760
0.642243309	-0.055307603956	-0.1278346895	0.638721582	0.538822174	0.034323960
0.103988918	-0.031402178902	-0.4649115180	0.164674980	0.035690052	0.037502750
0.060611192	-0.010968975941	-0.2435630049	0.066424300	0.043193191	0.007340115
0.144812332	-0.025810288735	-0.2631264803	0.159224947	0.084685600	0.025161273
0.034860683	-0.006997987148	-0.2446744493	0.032592516	0.024208791	0.003068595
0.054464318	-0.013487128745	-0.3386489968	0.060782992	0.028921376	0.010968441
0.143952009	-0.022336522135	-0.2261514365	0.154356171	0.099306089	0.018143589
0.035942122	-0.008201965077	-0.2837170697	0.042475852	0.018416480	0.007968161
0.065698434	-0.015589282748	-0.3326788044	0.065717503	0.031744322	0.010851534
0.025774081	-0.005849547741	-0.2575859555	0.023784749	0.015200927	0.003232869
0.105997051	-0.019675796237	-0.2681620358	0.103928704	0.068964641	0.012088109
0.093460616	0.053732175414	0.6034533687	0.241920649	0.070811968	0.060125377
0.456061653	-0.062481698824	-0.2076662965	0.434535952	0.337604517	0.032092601
0.048949549	-0.009929207227	-0.2661002281	0.051487514	0.028722822	0.006476454
0.388625519	-0.038370968043	-0.1460174151	0.390577070	0.314530104	0.029780733
0.033171526	-0.006686375172	-0.2427706697	0.033059847	0.021093953	0.004360009
0.069354736	-0.011259055004	-0.2207530274	0.073096393	0.047179872	0.007643161
0.672222302	-0.054016067705	-0.1190036573	0.668875759	0.578976117	0.033131463
0.107631025	-0.023030786696	-0.3143528944	0.127840138	0.054871675	0.023530087
0.661995746	-0.060839766243	-0.1369114672	0.685579341	0.519765388	0.043604962
0.676644725	-0.056554924536	-0.1240063746	0.683886549	0.529210321	0.049153428
0.750031779	-0.046883573881	-0.0918578167	0.760743799	0.658912859	0.037100728
0.047990035	-0.011012220534	-0.3038254069	0.048601157	0.032168612	0.005704626
0.061158729	-0.016939897462	-0.3922467192	0.055821634	0.030892241	0.009504125
0.046727385	-0.008515574849	-0.2346587613	0.048922977	0.029699180	0.005969938
0.023218347	-0.003791030989	-0.1748243195	0.022626272	0.013889578	0.002631000
0.064526284	-0.012890564649	-0.2739826537	0.059711658	0.045964870	0.004650732
0.022580459	-0.003648454353	-0.1713406355	0.021896489	0.016793452	0.001642599
0.065883857	-0.014914685658	-0.3157130672	0.079428633	0.040116577	0.011597657
0.058535342	-0.012821417526	-0.2988101975	0.072616474	0.033474670	0.012022552
0.028468711	-0.005140027455	-0.2069211519	0.027172182	0.016823852	0.003098827
0.071094085	-0.012398352985	-0.2393762191	0.071692540	0.045805265	0.007860577
0.029942577	-0.005812405842	-0.2268797600	0.029413353	0.017758749	0.004455570
0.065638788	-0.020103413849	-0.4457192281	0.053717222	0.038972002	0.005015612
0.089111925	-0.022373696314	-0.3691131839	0.087138386	0.040527661	0.015605552
0.028143721	-0.005263476773	-0.2142245708	0.028206343	0.019048153	0.003222676
0.911751509	-0.022098363362	-0.0350089844	0.922925785	0.856151255	0.022745631

0.056286719	-0.0073628471619	-0.1698676132	0.0552179621	0.040553633	0.004662039
0.574410955	-0.0416230657874	-0.1065946906	0.5922884081	0.495212858	0.034524314
0.059650123	-0.0099991973657	-0.2235813731	0.062921221	0.037655772	0.007167524
0.059070752	-0.0120514309259	-0.2766240652	0.055093645	0.033551107	0.007155433
0.613285181	-0.0506369578404	-0.1222432461	0.643546181	0.502236186	0.052310610
0.170666534	-0.0568620559760	-0.5452657913	0.168689272	0.039724777	0.041669855
0.706659713	-0.0375566544715	-0.0776577257	0.700742697	0.637548547	0.020761605
0.038791316	-0.0125347501283	-0.4283821102	0.033368428	0.018473923	0.006009617
0.342677670	-0.0473263094884	-0.2078799598	0.376141194	0.226353199	0.054600531
0.049237208	-0.0087956746098	-0.2318915068	0.046333639	0.032183403	0.004709409
0.170588769	-0.0238438248224	-0.2042892625	0.164400749	0.129054601	0.012244021
0.079115546	-0.0168694605390	-0.3027577087	0.082453475	0.050713772	0.010741747
0.432375811	-0.0557869293717	-0.1944723553	0.469082233	0.322869311	0.056223927
0.864411934	-0.0312805252917	-0.0525555869	0.879518480	0.780872201	0.028465850
0.041136854	-0.0071201442837	-0.2163120992	0.046974839	0.023465650	0.007581311
0.093622878	-0.0297995662649	-0.4891941890	0.082251910	0.052340369	0.010416643
0.100607853	-0.0152642834457	-0.2142462723	0.097777232	0.073462772	0.009612375
0.905771787	-0.0197634653517	-0.0314759879	0.906434466	0.855574981	0.017632423
0.403294483	-0.0456700875387	-0.1689375984	0.422258013	0.286608311	0.040646663
0.025583140	-0.0056665319790	-0.2502472142	0.025547506	0.016646287	0.002638608
0.175516286	-0.0552481720565	-0.5100618452	0.190748892	0.083681973	0.039229706
0.063449681	-0.0124232158763	-0.2673211974	0.067139856	0.037050788	0.009867889
0.037232008	-0.0086057830391	-0.2901841814	0.034024455	0.020814767	0.004602723
0.050334115	-0.0100144558079	-0.2618518279	0.051501674	0.027093685	0.008987426
0.078800700	-0.0119242818308	-0.2080299272	0.089359590	0.053721922	0.011294606
0.053434778	-0.0255031488822	-0.7418726770	0.032524433	0.023250242	0.002713605
0.063608987	-0.0130040440838	-0.2804464498	0.065299160	0.035766811	0.008537804
0.519572739	-0.0617545041146	-0.1788807608	0.588421189	0.396134799	0.063277118
0.054037048	-0.0147463834419	-0.3775923653	0.053577636	0.029791353	0.007463019
0.037576996	-0.0074058704197	-0.2441052923	0.036103001	0.023052859	0.004716045
0.180426635	-0.0441274113299	-0.3803131674	0.252347298	0.073456513	0.050328466
0.056005938	-0.0103875800717	-0.2470346658	0.059495793	0.037084255	0.006737337
0.598661134	-0.0485790858756	-0.1200010581	0.628434580	0.481668633	0.049021639
0.035254769	-0.0072075987735	-0.2502806726	0.038044593	0.023039149	0.004493788
0.038930716	-0.0076590829372	-0.2455898663	0.048241953	0.022339899	0.008290862
0.671825031	0.0838873272790	0.1674009775	0.852797530	0.660064219	0.068300036
0.444587316	-0.0403701863852	-0.1341702139	0.436654471	0.365245378	0.022417146
0.032795064	-0.0063326792291	-0.2310354830	0.033801639	0.020415448	0.004385054
0.034373952	-0.0064807841949	-0.2277752921	0.033384247	0.022379595	0.003717968
0.790571450	-0.0664830130508	-0.1250762895	0.836355248	0.673883425	0.051262581
0.073609515	-0.0133572097269	-0.2511215645	0.072769083	0.047213145	0.007102898
0.059316207	-0.0157921941337	-0.3730064245	0.058065503	0.032731202	0.009113166
0.123502706	-0.0402844147917	-0.5181840078	0.118753475	0.030829117	0.031217851
0.037506032	-0.0083773462388	-0.2798840508	0.035597605	0.022966670	0.005234655

0.041933173	-0.0075875590471	-0.2278649524	0.043158791	0.025027984	0.005308866
0.523612643	-0.074518155672	-0.2170017400	0.509002412	0.366234282	0.055388668
0.027144562	-0.004472487282	-0.1850929385	0.025721376	0.017660376	0.002274028
0.043401931	-0.008123153396	-0.2380568987	0.042747909	0.023741532	0.006359231
0.195869658	-0.025769299104	-0.1929301576	0.186051944	0.137993922	0.015168457
0.042771512	0.0795158936699	############	0.382704446	0.023835555	0.136384002
0.213452503	-0.036486010849	-0.2571884686	0.230267537	0.145518944	0.027767438
0.023057809	-0.004124384367	-0.19225401431	0.023958655	0.013948993	0.003493831
0.817331714	-0.0575834541351	-0.1040791849	0.842235585	0.707235956	0.044890839
0.028674654	-0.005869206881	-0.2374529948	0.029260451	0.015034109	0.004464478
0.210016694	-0.032908423789	-0.2337396657	0.221012929	0.155111944	0.019050826
0.025718791	-0.004604042904	-0.1990846771	0.028185607	0.015843068	0.003555225
0.035729964	-0.008536482534	-0.2980899816	0.030637198	0.023234878	0.002604787
0.049980498	-0.008839847432	-0.2300229867	0.056521120	0.033123101	0.008446695
0.183824929	-0.068730327669	-0.6317346050	0.191072002	0.060197075	0.043727713
0.028916898	-0.006271705394	-0.25352616241	0.028186661	0.019109831	0.003367834
0.881632815	-0.023085441512	-0.0378451729	0.878946143	0.820547489	0.017286550
0.134641521	-0.050440134914	-0.61866155781	0.149111290	0.046114886	0.028899967
0.151606167	-0.030974469898	-0.3069772495	0.153707741	0.089659240	0.021290575
0.938501543	0.013221980967	0.0199720874	0.961692569	0.921677909	0.012407046
0.028109913	-0.0057741080901	-0.2370337907	0.026821609	0.013451370	0.003911644
0.115798674	-0.038766401780	-0.53149434741	0.123910500	0.062630006	0.019128628
0.055052886	-0.009901933380	-0.2382272559	0.054966068	0.032918327	0.007003164
0.063514329	-0.008526701290	-0.1778603926	0.062027592	0.048912947	0.004839807
0.045509200	-0.011769076139	-0.3437695923	0.042149438	0.022700804	0.006714642
0.042334351	-0.007508633779	-0.2234315407	0.041277459	0.025928055	0.004773665
0.042829978	-0.007520182472	-0.2215338265	0.043165793	0.028653179	0.004757652
0.314673838	-0.053846780630	-0.2616192564	0.314948732	0.149935000	0.054348055
0.632207834	-0.043808765177	-0.10193233361	0.673231959	0.556638873	0.034481354
0.026796171	-0.004178964938	-0.1739224348	0.028686449	0.019006593	0.002854699
0.104219725	-0.026719841404	-0.3844588132	0.102312258	0.061188983	0.013046837
0.038054243	-0.0082034489221	-0.2700553440	0.042084064	0.022437068	0.006270059
0.922955763	0.024638166157	0.0376053301	0.967840138	0.907683744	0.017847441
0.053241872	-0.010143740401	-0.2522189761	0.055484605	0.033933313	0.007606110
0.046624707	-0.008152017877	-0.2242595553	0.053005252	0.030072521	0.007681848
0.054038174	-0.014467573188	-0.3694474239	0.060017085	0.028422059	0.009821429
0.153851627	-0.0233476343221	-0.22177886931	0.142952596	0.111821988	0.010964058
0.785487935	-0.034668382404	-0.0642857265	0.780975955	0.707561105	0.022026552
0.833111367	-0.030728779927	-0.0535638933	0.831927832	0.772692131	0.020177497
0.098337806	-0.028748188848	-0.44488463381	0.088409753	0.041960691	0.016882375
0.754443508	-0.060368610632	-0.1186809844	0.755624594	0.602682401	0.052538754
0.351281787	-0.042123141313	-0.1788507973	0.353378811	0.239915542	0.033287697
0.055390895	-0.0075288147141	-0.1764715752	0.055028535	0.041531603	0.005084421
0.147617042	-0.042305030344	-0.4508807283	0.163846303	0.069162125	0.026976176

0.062971502	-0.020150479942	-0.4662209297	0.057971687	0.032031808	0.010038587
0.058810568	-0.012719150555	-0.2948501087	0.066229631	0.034123126	0.009446049
0.064294994	-0.012619771922	-0.2685739983	0.069532716	0.040415198	0.009453749
0.311667819	-0.034836830434	-0.1653707115	0.316386038	0.228604802	0.026452613
0.043346033	-0.008249592806	-0.2423674314	0.039942295	0.027869926	0.005106929
0.026594740	-0.004830791220	-0.2042460200	0.026962439	0.016285949	0.003767466
0.537879651	-0.046283924606	-0.1273339645	0.526795650	0.424959305	0.027437426
0.795155749	-0.049721749793	-0.0919621690	0.815161654	0.646964121	0.049920116
0.065186924	-0.014704293087	-0.3139608797	0.058311196	0.037964103	0.006191530
0.066067094	-0.021986863673	-0.4921711938	0.054547680	0.034505183	0.007573715
0.834328860	-0.036617874615	-0.0639658665	0.841549204	0.736289393	0.034741326
0.051377176	-0.011633962997	-0.3032025685	0.053533004	0.028535794	0.008428150
0.051225071	-0.010615697203	-0.2747179157	0.051848805	0.025660039	0.007845838
0.068382207	-0.011002932056	-0.2182212902	0.076941478	0.047689838	0.009087272
0.701759800	0.065629268919	0.1272463695	0.824493720	0.729438887	0.027882660
0.123055446	-0.036043011155	-0.4557859742	0.127727106	0.042903898	0.029101806
0.577980031	-0.049050823866	-0.1256713835	0.572200211	0.458229127	0.043626739
0.135473462	-0.035661196417	-0.4057167838	0.140675177	0.076119329	0.020609289
0.111425519	-0.024915943764	-0.3313276580	0.149624625	0.045352545	0.032266326
0.406158882	-0.077856585218	-0.2988214567	0.406969915	0.255246454	0.043354884
0.031452902	-0.006840628015	-0.2601893712	0.029155778	0.019119007	0.003529657
0.063627673	-0.010786964004	-0.2285486462	0.065796566	0.037156757	0.009337655
0.665436670	-0.061355774548	-0.1373917861	0.679035757	0.473926693	0.063651225
0.896116819	-0.020586267777	-0.0331549768	0.912837546	0.850943324	0.019202685
0.735094925	-0.043812881070	-0.0874294476	0.759280289	0.637439098	0.038345090
0.128610361	-0.023225741656	-0.2645841746	0.126206027	0.069669307	0.017971359
0.509601945	-0.050417589254	-0.1472519214	0.507969031	0.414857177	0.030445917
0.456323766	-0.065316056350	-0.2177019839	0.451299620	0.314251662	0.045852979
0.038073512	-0.008266346350	-0.2722140501	0.039967298	0.024017644	0.005755952
0.559346774	-0.057573500660	-0.1538028208	0.552891985	0.450644185	0.034591436
0.033697730	-0.007380909648	-0.2669204400	0.034738135	0.014758931	0.007028512
0.090762505	-0.022217167945	-0.3593613539	0.092652806	0.038869460	0.017309922
0.056300426	-0.009696828225	-0.2281243826	0.057387762	0.035724801	0.008863214
0.048377646	-0.009262881793	-0.2492592605	0.047978274	0.031281086	0.005944054
0.081024814	-0.012361675652	-0.2105721417	0.087038179	0.056342064	0.008742778
0.207433354	-0.032285063429	-0.2318920347	0.219719460	0.129596982	0.028076972
0.037184616	-0.007171798412	-0.2378543361	0.039889632	0.023601671	0.005203240
0.059935878	-0.009605210429	-0.2131412174	0.062275284	0.044654179	0.005316883
0.628321531	-0.056597958460	-0.1339495455	0.635955515	0.439413035	0.065421497
0.296329412	-0.057436890914	-0.2995610123	0.295812637	0.177300362	0.044274434
0.043492906	-0.009291888546	-0.2752679963	0.039803273	0.023069564	0.005029873
0.361013171	-0.045043101915	-0.1867309022	0.376112100	0.262599211	0.036883514
0.037022206	-0.008001036280	-0.2690851970	0.037751599	0.020265974	0.005545079
0.036749771	-0.007351668567	-0.2468331509	0.036339012	0.020362573	0.004963759

0.040430270	-0.007294760184	-0.2254139419	0.041343490	0.028947048	0.003894353
0.056312058	-0.017087740596	-0.4299002816	0.048911409	0.031751511	0.006442949
0.559398612	-0.056426727299	-0.1505592203	0.624725224	0.431692696	0.057398223
0.855622960	-0.020983741147	-0.0354035291	0.856893410	0.802812432	0.017972655
0.491066969	-0.049110349532	-0.1488191324	0.469801780	0.409729908	0.024061470
0.050968867	-0.016879785587	-0.4676513098	0.054009175	0.020810074	0.011946876
0.051894637	-0.007433473048	-0.1845865912	0.053919362	0.037336542	0.005234745
0.577973710	-0.051746495640	-0.1329072092	0.577433090	0.482258890	0.032761554
0.065653589	-0.015118372922	-0.3216338552	0.074671675	0.039819072	0.009921653
0.156240777	-0.043638712920	-0.4392910337	0.159914826	0.074761328	0.024189342
0.500221223	-0.066762400828	-0.2023227620	0.477942503	0.293689313	0.058417646
0.075972717	-0.017472609197	-0.3277726394	0.073799743	0.051228387	0.008720966
0.089061367	-0.018285665158	-0.2944011464	0.083386174	0.032137618	0.015850092
0.047725595	-0.009192302518	-0.2502363989	0.045202912	0.028208153	0.005294998
0.101646286	-0.023202473308	-0.3361021332	0.112932556	0.052472188	0.020079868
0.034822894	-0.005910002642	-0.2039876169	0.042316645	0.021894229	0.005711626
0.280920726	-0.037317109699	-0.1980507633	0.312629237	0.208280845	0.031987661
0.346453982	-0.060805006012	-0.2698305566	0.364725107	0.208962158	0.053025613
0.153567457	-0.024598887808	-0.2351271175	0.149158561	0.106979147	0.016791283
0.029115768	-0.006459603574	-0.2603949334	0.028182761	0.016841252	0.003951419
0.056239130	-0.009889318768	-0.2332729467	0.053611830	0.037698879	0.005560246
0.885357412	-0.029570892113	-0.0484523592	0.888981262	0.824396535	0.020842541
0.034286060	-0.006644781324	-0.2345370087	0.034866815	0.021857583	0.005262581
0.891219384	-0.024013980627	-0.0389636455	0.892127150	0.827864502	0.021882848
0.171173581	-0.022276400224	-0.1892790668	0.178886660	0.123156758	0.016328930
0.904244918	-0.036178323549	-0.0582503410	0.918725758	0.759779353	0.048698786
0.072502796	-0.022104399816	-0.4499327692	0.063938594	0.039458394	0.008801799
0.629103121	-0.050343466049	-0.1183699192	0.674483593	0.529257196	0.049059921
0.023150754	-0.006144004906	-0.2957216938	0.023787946	0.013728276	0.003085312
0.025341795	-0.005305323488	-0.2346599693	0.026342583	0.014451721	0.003448533
0.783023197	-0.060978833463	-0.1154319855	0.839400528	0.621411380	0.064130149
0.860059877	-0.031775819630	-0.0536754974	0.862832786	0.796538954	0.023992651
0.043429995	-0.007669895601	-0.2235596927	0.047841443	0.029979541	0.005493761
0.886639221	-0.018239822607	-0.0296505412	0.892234621	0.842496043	0.015788249
0.047437760	-0.007540490086	-0.2030386020	0.054652607	0.034246967	0.006292185
0.039354520	-0.009362095505	-0.3034554702	0.038183184	0.024350667	0.004159690
0.032497680	-0.006396026980	-0.2353191836	0.035632929	0.019510340	0.005307375
0.028669571	-0.006136284237	-0.2492821754	0.031226447	0.016257979	0.004987096
0.046077646	-0.012069097816	-0.3496420066	0.042127904	0.025689754	0.006727819
0.052217271	-0.009406340743	-0.2364785592	0.052014357	0.032579566	0.005806880
0.050642834	-0.009555488730	-0.2473712039	0.051067646	0.031308994	0.007269918
0.526766224	-0.043573514104	-0.1221424180	0.526773937	0.454325189	0.025082294
0.835249449	0.0322547671720	0.05402888650	0.893278143	0.817188069	0.021909950
0.762105957	-0.042047343483	-0.0807865468	0.779233799	0.661588161	0.042008605

0.253902392	-0.043675116313	-0.2610112654	0.274946483	0.154225572	0.039122056
0.179113830	-0.053095651806	-0.4754553970	0.161372001	0.081314419	0.029514546
0.137161864	-0.031029984789	-0.3416397961	0.149052837	0.071063022	0.023837843
0.770344069	-0.051769662777	-0.0990340605	0.764547807	0.661853178	0.034079774
0.796867272	-0.042329952266	-0.0777444463	0.829817088	0.701998988	0.043810260
0.276322211	-0.054470582646	-0.3044377299	0.294410516	0.174405085	0.036312243
0.095171861	-0.026635108875	-0.4213089172	0.092168247	0.052404543	0.013654020
0.640779080	-0.037754908856	-0.0862239151	0.635207096	0.545735250	0.028973936
0.556797114	-0.0552475261801	-0.1479583156	0.567453089	0.449851383	0.032765736
0.256696893	-0.035378755812	-0.2053226283	0.249522200	0.192334176	0.021601293
0.419088948	-0.101934355060	-0.3913042168	0.496198597	0.174446146	0.126154272
0.055683237	-0.014884589677	-0.3707351436	0.052980744	0.028556503	0.008935191
0.964782912	-0.0105341898011	-0.0156756317	0.966163643	0.925301094	0.012931301
0.313160009	-0.036265598496	-0.1717288149	0.338465110	0.226900492	0.031010640
0.030504451	-0.005770513727	-0.2217344927	0.031804188	0.018227038	0.004378147
0.571496504	-0.061881001904	-0.1623260455	0.584691644	0.423336336	0.049783254
0.063654558	0.0501655605264	0.74939902591	0.201276931	0.039750422	0.065108331
0.041861679	-0.007525860649	-0.22619620401	0.052027945	0.026852651	0.007958524
0.099274052	-0.0312501847211	-0.48596344631	0.110392280	0.038161957	0.022911222
0.703785969	-0.060997042852	-0.1288749584	0.724233904	0.524682174	0.053275848
0.873485080	0.0399564405289	0.06381483018	0.939484681	0.865544812	0.021605782
0.083649151	-0.015523755351	-0.2614743236	0.097106609	0.049601906	0.014116066
0.037785787	-0.005455907213	-0.1749051838	0.036729217	0.028483240	0.003111877
0.058276324	-0.009141951101	-0.2073884189	0.055429310	0.041736050	0.005133433
0.178220435	-0.032517651376	-0.2736285315	0.195022674	0.119992130	0.023784533
0.709588156	-0.052496274641	-0.1092859572	0.710612977	0.562316097	0.044623339
0.233186348	-0.034640916754	-0.2217005343	0.229645049	0.169367642	0.020120551
0.622581982	-0.039389436352	-0.0927519954	0.636200601	0.555223807	0.022506556
0.110765421	-0.030807795168	-0.4248899290	0.111014569	0.050624172	0.020867345
0.052299881	-0.006969246574	-0.17115094121	0.050977619	0.038706362	0.003640308
0.044874360	-0.008422868400	-0.2404072836	0.042436952	0.031643292	0.003334601
0.284122324	-0.029726524643	-0.1537170335	0.290606882	0.232162446	0.023701012
0.850300786	-0.037587188185	-0.0644508672	0.859368622	0.779526850	0.030459328
0.113427273	-0.0145293008071	-0.1806841227	0.114772148	0.090823504	0.007430966
0.715385417	-0.049120775689	-0.1011598178	0.702676653	0.574102106	0.043851881
0.027109236	-0.004711675454	-0.1958931091	0.027968080	0.018997640	0.002906707
0.088919096	-0.014201694091	-0.2235907077	0.101437704	0.057920276	0.013228433
0.062770260	-0.012618080967	-0.2747319641	0.067484466	0.021981517	0.012802352
0.588184496	-0.041016043158	-0.1024769443	0.607449894	0.483572784	0.033797040
0.027973244	-0.005395618590	-0.2211017969	0.028173846	0.014608160	0.004610808
0.087465762	-0.027478676756	-0.4778067848	0.102366019	0.033874124	0.020180262
0.160921850	-0.044113269136	-0.4306844643	0.172322679	0.094075467	0.026180851
0.674194553	-0.051055074934	-0.1118832639	0.707273041	0.516856179	0.061034670
0.078683051	-0.013551409602	-0.2392377818	0.081268835	0.046302641	0.010382821

0.696814608	-0.052042982214	-0.1103400520	0.691271160	0.515684427	0.055204370
0.545120330	-0.077146010116	-0.21586742061	0.580327458	0.383790778	0.064855243
0.787590891	-0.052132446705	-0.0975210018	0.788995649	0.675266049	0.038120016
0.047509636	-0.008060309275	-0.2178528279	0.051808124	0.032795897	0.005809546
0.041204795	-0.007399193358	-0.2251635466	0.044224678	0.023685535	0.005726261
0.746696019	-0.051197985270	-0.10107179301	0.753198733	0.658957920	0.028769294
0.175757162	-0.043514404819	-0.3850626410	0.182348987	0.084137155	0.033273507
0.354444362	-0.046406403950	-0.1964996194	0.346480888	0.261559595	0.027453639
0.160794588	0.0553011272262	0.40467138996	0.354482914	0.122488448	0.063518106
0.067142530	-0.013299096842	-0.2729882270	0.067148247	0.041338654	0.008228883
0.043574742	-0.007816812037	-0.2275312078	0.043182988	0.025334455	0.005157238
0.028076583	-0.004605197623	-0.1859757496	0.027896332	0.020102917	0.002952670
0.096689003	-0.019221585896	-0.2865938796	0.107594212	0.061926525	0.015047363
0.037810774	-0.007762100032	-0.2555812852	0.042737555	0.022222034	0.007497632
0.138740257	-0.023161414891	-0.2442017543	0.139615825	0.091793539	0.014551577
0.171865546	-0.059591170960	-0.5727501611	0.149904334	0.040338379	0.036528568
0.033178140	-0.006439401275	-0.2329989968	0.032580705	0.020288225	0.003824788
0.034269176	-0.009358286556	-0.3426254046	0.040548306	0.015499273	0.006962662
0.840266948	-0.032480439356	-0.0561915954	0.867055857	0.754385886	0.035858297
0.908947699	-0.018629670057	-0.0295480442	0.919750985	0.868110155	0.017948509
0.068772333	-0.013596147477	-0.2733440845	0.069294570	0.037023851	0.010676032
0.222619943	-0.044961153461	-0.3098629218	0.256654402	0.136764412	0.036074850
0.046959189	-0.0097950747601	-0.2722390261	0.044232858	0.031149899	0.004967831
0.055863226	-0.014228337458	-0.35112698371	0.062131720	0.029985688	0.010505817
0.073748359	-0.011080337937	-0.2047403033	0.074168461	0.055174369	0.006046559
0.073484032	-0.020580213176	-0.4083526733	0.067510830	0.037035646	0.009384728
0.024769269	-0.004036298776	-0.1780255263	0.025782145	0.016775058	0.003168006
0.070815857	-0.014131018996	-0.2772796153	0.071745523	0.041484160	0.010884872
0.035504237	-0.006242497556	-0.2128767913	0.034502176	0.024546002	0.002998848
0.035188276	-0.006540842124	-0.2255758885	0.037310654	0.018755137	0.005506186
0.710270372	-0.037948074179	-0.0780851974	0.713439857	0.641158982	0.022829655
0.044171234	-0.007080333753	-0.2020786501	0.041985261	0.030062910	0.004519871
0.033832556	-0.004674941002	-0.1627099107	0.034182426	0.022754226	0.004110929
0.107068345	-0.025005257310	-0.3466562900	0.120195861	0.046357875	0.022746440
0.024020987	-0.003573671889	-0.1601099782	0.024163292	0.017238903	0.002106047
0.034981235	-0.005290903386	-0.18053565461	0.037052874	0.022261186	0.004656397
0.119864929	-0.017239823842	-0.2054834190	0.117177603	0.081155699	0.012185434
0.316896987	-0.036544091540	-0.1710286585	0.314740775	0.242755561	0.026058460
0.045409417	-0.010229053578	-0.29443530851	0.045337609	0.026507166	0.007146311
0.115655661	-0.020815561824	-0.2612850436	0.116850870	0.079612865	0.012905336
0.047294629	-0.010916004194	-0.3049398544	0.057256409	0.022982676	0.011483465
0.858408339	-0.025693436109	-0.0433289410	0.869048137	0.814487835	0.018738978
0.050761097	-0.010240851621	-0.26628626641	0.056770160	0.023063216	0.009916906
0.254099761	0.1531941353532	0.65998082250	0.554859889	0.120349250	0.155635394

0.855344314	-0.0358448024757	-0.06103315285	0.856988056	0.754468637	0.032046164
0.843070684	-0.0286679996697	-0.04931608331	0.858483832	0.772814643	0.028581833
0.841670817	-0.0392300605724	-0.06803330094	0.856623486	0.741797404	0.037505199
0.069238834	-0.0114514414131	-0.22519067130	0.062433314	0.051647921	0.004317507
0.043947595	-0.0094331836340	-0.27728616197	0.044330767	0.028165712	0.004866762
0.383208099	-0.0341493750661	-0.13107322181	0.376635247	0.308213695	0.021094038
0.722407471	-0.0426380823970	-0.08653240295	0.725820689	0.615556903	0.039031006
0.082926319	-0.0119588843782	-0.19874549226	0.076785445	0.062047325	0.004993162
0.029234443	-0.0066337835342	-0.26721956488	0.026291980	0.018326777	0.002782205
0.795072776	-0.0482520008599	-0.08916751205	0.815990844	0.673232993	0.049755040
0.627036379	-0.0523298663072	-0.12366310139	0.633999661	0.510975381	0.044952963
0.040074054	-0.0080012256735	-0.25117445074	0.040047175	0.024449000	0.005050605
0.101200129	-0.0425366041722	-0.69554263590	0.135606440	0.027742944	0.031302362
0.063294973	-0.0090629849106	-0.19042230103	0.060172776	0.049296566	0.004568951
0.859926998	-0.0429566715427	-0.07305877562	0.910890184	0.762399444	0.043411866
0.025284765	-0.0045786323384	-0.20051857947	0.027159597	0.015929970	0.004090866
0.096100238	0.0249235173733	0.30440051085	0.156201244	0.078147601	0.026088623
0.889586253	-0.0227961033783	-0.03703002445	0.895601809	0.825146939	0.021398266
0.069496429	-0.0172309468406	-0.35245744969	0.085658570	0.034803427	0.014540103
0.814023977	-0.0273050752941	-0.04861551426	0.814629162	0.734556511	0.024031329
0.031466119	-0.0058690253685	-0.22017356915	0.030810391	0.021311765	0.003618219
0.077999010	-0.0181286665016	-0.33280705666	0.081478665	0.045798622	0.011036014
0.147208767	-0.0505875379807	-0.56018695259	0.151336228	0.063856584	0.028815855
0.043080007	-0.0076748628611	-0.22531280296	0.041695145	0.030751318	0.003367667
0.024243093	-0.0037744701726	-0.16848872506	0.024761617	0.015600137	0.003163800
0.876733960	-0.0195217862639	-0.03211631959	0.879567455	0.821285784	0.016395169
0.819303892	-0.0409125544047	-0.07298893810	0.824446470	0.705541756	0.036798044
0.465618150	-0.0414851064325	-0.13166653465	0.480806407	0.398783501	0.025214608
0.083740977	-0.0161733914325	-0.27322593439	0.081078366	0.053008862	0.009298367
0.051679283	-0.0084918772496	-0.21370132215	0.052519091	0.032446322	0.007339303
0.174815200	-0.0337813986767	-0.29121195400	0.167543495	0.092242589	0.023382664
0.027861427	-0.0051029516459	-0.20886057453	0.033491774	0.016352320	0.006227831
0.538927493	-0.0616099023927	-0.17175329794	0.562371489	0.413898112	0.050221132
0.102787353	-0.0168206303991	-0.23299917559	0.107075734	0.056115480	0.013912125
0.733095960	0.0717913568045	0.13305205030	0.908154335	0.703686216	0.071042593
0.404332110	0.0948141025585	0.29729238610	0.736648045	0.369096321	0.111415998
0.046875279	-0.0120801647316	-0.34446032689	0.044179800	0.027666149	0.005489058
0.059513184	-0.0148326285870	-0.34625872586	0.065206257	0.034101801	0.009832441
0.242127262	-0.0389302015401	-0.24196457542	0.279183620	0.162767028	0.035836875
0.632109157	-0.0655433289163	-0.15533323913	0.617349756	0.495825425	0.044738015
0.031526477	-0.0072203982367	-0.27556724783	0.027929170	0.019221810	0.002265258
0.045154961	-0.0067050715604	-0.18699728077	0.047125562	0.032845499	0.004445352
0.048082915	-0.0092162195514	-0.24926230956	0.065961521	0.025803757	0.012082903
0.036003695	-0.0054153838141	-0.18068542656	0.039516620	0.025939419	0.003978790

0.0298182159	-0.0047978820067	-0.18523574166	0.0333911040	0.0221763021	0.0033831690
0.0403810390	-0.0065214744694	-0.19998938398	0.0493537230	0.0267233340	0.0071466071
0.0213266030	-0.0035528536791	-0.17366638761	0.0231827760	0.0131729058	0.0036799937
0.1234401901	-0.0400472897865	-0.51480846216	0.1061122369	0.0604528650	0.0131940604
0.8317915011	-0.1071519691525	-0.19642641240	0.8702028701	0.4295812450	0.1631955518
0.0416533688	-0.0082545689207	-0.25120729554	0.0418006811	0.0235354194	0.0058102169
0.5454218040	-0.0540693949124	-0.14775875871	0.5853767420	0.4241538130	0.0511544545
0.0993721650	-0.0173426732698	-0.24907745599	0.1045872851	0.0715093041	0.0124089160
0.0812145098	-0.0110468295379	-0.18624260798	0.0849521370	0.0561652351	0.0109128605
0.4958925447	-0.0449032858487	-0.13409784245	0.5294861981	0.3939180145	0.0368345997
0.6033188679	-0.0407228805918	-0.09911977403	0.6088289761	0.5328337860	0.0299977130
0.8859757102	-0.0314439745070	-0.05154069745	0.8863823489	0.7928307421	0.0298803990
0.3196004005	-0.0466936383170	-0.22039133434	0.3506964901	0.2239613869	0.0433137791
0.5076006949	-0.0689616147951	-0.20628425785	0.5494634235	0.3341735210	0.0693023045
0.6408928050	-0.0564682032162	-0.13092613993	0.6701037708	0.4648099221	0.0661828925
0.0278201997	-0.0048220539271	-0.19677202187	0.0285023215	0.0197185334	0.0029918071
0.0339202594	-0.0050334263654	-0.17560482937	0.0325333729	0.0258522905	0.0025272750
0.0683899031	-0.0124141493984	-0.24873191435	0.0676457011	0.0387814779	0.0102467198
0.0655604401	-0.0104020349517	-0.21367981244	0.0750138560	0.0375820060	0.0117429140
0.7947360200	-0.0472108787409	-0.08722183866	0.8301939471	0.6875156204	0.0503837004
0.0284960970	-0.0044486255592	-0.17716452327	0.0328832655	0.0193122874	0.0040244028
0.8681763111	-0.0307812672679	-0.05147592730	0.8785666907	0.7715157578	0.0332157031
0.0385173269	-0.0079252874870	-0.25730324046	0.0394802980	0.0215905348	0.0062340238
0.0304758180	-0.0051235588164	-0.19525782026	0.0302879851	0.0210925294	0.0026100318
0.0483679379	-0.0109373634349	-0.29935875758	0.0464808100	0.0307522169	0.0063325831
0.0927972681	-0.0279663180451	-0.45809492648	0.1194205608	0.0352991280	0.0263635690
0.0327145224	-0.0066492639862	-0.24411689031	0.0318303491	0.0169419971	0.0052558071
0.0733473815	-0.0128418427177	-0.24140027755	0.0755124791	0.0482834898	0.0103810630
0.0934191285	-0.0502339791876	-0.95940768337	0.0644136345	0.0278762630	0.0120398080
0.5604346968	-0.0611919591201	-0.16370823559	0.6039497391	0.4252203595	0.0524509231
0.8956073444	-0.0223429935089	-0.03604037377	0.8983445931	0.8132461625	0.0262762411
0.5958086964	-0.0542631335628	-0.13538221732	0.6007887374	0.4825620131	0.0466595750
0.4941463359	-0.0423507524270	-0.12658818247	0.4861070598	0.3884532491	0.0279795921
0.8069401600	-0.0601538537873	-0.11034444251	0.8397750991	0.6159533857	0.0709535641
0.8752498725	-0.0357987726465	-0.05955383229	0.9074770810	0.7831533615	0.0345281771
0.9208847944	-0.0162106949777	-0.02534483273	0.9338105780	0.8924351499	0.0130342670
0.4781095491	-0.0625544091798	-0.19785889950	0.4707800078	0.3509309731	0.0376117419
0.0829160294	-0.0274403871928	-0.50496919926	0.1000494844	0.0387559919	0.0192619700
0.4557201289	-0.0713293625445	-0.23983746313	0.5275254651	0.2959591584	0.0631805831
0.4378897211	-0.0548587706246	-0.18850063061	0.5089621299	0.3323255478	0.0624399971
0.3443528808	-0.0586827059175	-0.26119805588	0.3315044231	0.2268055059	0.0395860405
0.1479923750	-0.0195206814844	-0.19026384329	0.1443580368	0.1055048198	0.0122393861
0.8639078770	-0.0311142591278	-0.05230181673	0.8604438685	0.8036801671	0.0210146528
0.8488190738	-0.0324896988569	-0.05563727822	0.8462976510	0.7793179541	0.0226083179

0.0228118999	-0.0035340014988	-0.1644071326	0.0253616529	0.0154311281	0.0029838358
0.0494648461	-0.0089306272606	-0.2347764120	0.0525804661	0.0299407561	0.0074709560
0.2696946311	-0.0412434139178	-0.2301584135	0.2785497904	0.1762024139	0.0307335735
0.1069888369	-0.0312493927983	-0.4483399053	0.1205515478	0.0489898321	0.0212125071
0.0568966403	-0.0100303117146	-0.2343590932	0.0544988511	0.0373591584	0.0058629030
0.3490849625	-0.0316886054275	-0.1332869690	0.3518205665	0.2800169534	0.0212895620
0.0753280869	-0.0163712865771	-0.3073278690	0.0765203970	0.0408767029	0.0125397029
0.0270114408	-0.0047780581254	-0.1994157007	0.0292828467	0.0194775677	0.0029664985
0.0396633465	-0.0078285805636	-0.2474791152	0.0399173674	0.0247975274	0.0050119857
0.0884696631	-0.0296851837326	-0.5175962521	0.0819537751	0.0415159704	0.0138823548
0.0210077980	-0.0038426019787	-0.1908716160	0.0216001857	0.0128581418	0.0023222924
0.0419553535	-0.0088131241216	-0.2681716827	0.0455654078	0.0271422719	0.0059628371
0.0963244240	-0.0200267445926	-0.3010793691	0.1108514797	0.0544010479	0.0186807528
0.1099472580	-0.0176513661490	-0.2296519694	0.1160819435	0.0714308154	0.0157436549
0.1917574874	-0.1225921379676	############	0.3512040711	0.0212998371	0.1060716224
0.0355952417	-0.0056742426923	-0.1917354489	0.0348174961	0.0241436080	0.0035367281
0.0545890871	-0.0093537202555	-0.2256981176	0.0549637239	0.0326299388	0.0070285577
0.7230431809	-0.0565190623329	-0.1157568165	0.7640231291	0.6021036227	0.0498477631
0.0363976091	-0.0064382504113	-0.2155170344	0.0423471129	0.0209893660	0.0061728861
0.0269429864	-0.0050510512799	-0.2121088562	0.0294898874	0.0164899658	0.0042512210
0.0731594127	-0.0093415982136	-0.1719105446	0.0704635101	0.0525732300	0.0061046411
0.7150537020	0.1025705823431	0.1908881236	0.8823915667	0.6675428680	0.0668018221
0.9425888061	0.0092016251675	0.0138689774	0.9646307211	0.9419913110	0.0072469891
0.6939599650	-0.0433737804691	-0.0917465860	0.6974448460	0.6041366575	0.0329739583
0.0665978981	-0.0111605380331	-0.2271902568	0.0721489849	0.0471084889	0.0079859761
0.0383864398	-0.0088461580936	-0.2912796415	0.0410695877	0.0208940914	0.0072204471
0.8922330868	-0.0595636333701	-0.0985333649	0.9079627344	0.6883693250	0.0813122703
0.0559389931	-0.0103926070514	-0.2474388065	0.0514866609	0.0373736729	0.0042679593
0.4243532399	0.1202198009074	0.3525086862	0.6847572440	0.3368043288	0.0950423425
0.6954329730	-0.0487816524900	-0.1033815029	0.7432758997	0.5620858237	0.0541966921
0.8581762828	-0.0299032874936	-0.0505678567	0.8566410081	0.7896554139	0.0238220711
0.0294443910	-0.0083858578853	-0.3448304631	0.0271561385	0.0158882271	0.0036569250
0.7539731931	-0.0476238183826	-0.0928586327	0.7797500065	0.6275868585	0.0577494827
0.0262657807	-0.0036912606081	-0.1548649818	0.0277244989	0.0174559697	0.0039103671
0.7067253240	-0.0432316270902	-0.0897558861	0.7144664704	0.5951830460	0.0411978708
0.0307898641	-0.0060133989688	-0.2300994027	0.0384237301	0.0183309921	0.0063910720
0.8594857291	-0.0293622432825	-0.0495609502	0.8666105230	0.7817758809	0.0271344170
0.0492733935	-0.0093034572298	-0.2463242783	0.0474072800	0.0336658049	0.0050639010
0.0834170087	-0.0164966615530	-0.2803199756	0.1000915380	0.0501905668	0.0157950498
0.8061201438	-0.0393929305454	-0.0713735390	0.8149481537	0.7055394261	0.0419417301
0.7922537231	-0.0404546308404	-0.0746480109	0.8031105311	0.6949793845	0.0406231194
0.8122022607	-0.0312192932611	-0.0558467098	0.8359407771	0.7027792121	0.0357600889
0.2103345564	0.1293066301441	0.6661793598	0.4770959640	0.0714408568	0.1106334514
0.0571887885	-0.0176915168093	-0.4408715113	0.0492007941	0.0313856251	0.0064232821

0.032073767	-0.004795510919	-0.1745866183	0.035510180	0.021402225	0.004267416
0.081438510	-0.021230317411	-0.3811625047	0.112769481	0.036520136	0.024910128
0.126492224	-0.015431942392	-0.1730931499	0.145052024	0.096851435	0.014290930
0.034519290	-0.007315710242	-0.2589891349	0.040179071	0.018761651	0.006561448
0.105023942	-0.013926229225	-0.1861838280	0.109510960	0.068860704	0.013453877
0.096603203	-0.022394303194	-0.3402061630	0.108661354	0.049638286	0.019937749
0.044305005	-0.006566546731	-0.1859331939	0.044941548	0.029605287	0.004820338
0.145892143	-0.017900410208	-0.1759663805	0.145040490	0.115738820	0.010869732
0.049188243	-0.011473321016	-0.3108701119	0.055393447	0.027417888	0.008741823
0.484889352	-0.041195325814	-0.1253863377	0.506696771	0.401318410	0.032102756
0.030432877	-0.004965345796	-0.1890299458	0.036296406	0.021079014	0.004557271
0.247541705	-0.040650367762	-0.2478336416	0.269702217	0.148980591	0.040922548
0.754600701	-0.053608000326	-0.1048717741	0.766092740	0.614434953	0.058734886
0.090157903	-0.020656085536	-0.3332165149	0.105917374	0.034081449	0.023797236
0.536118281	-0.070643601570	-0.1998449360	0.525245287	0.378334011	0.047980818
0.073935930	-0.013553887817	-0.2540811325	0.078334297	0.037268477	0.012846186
0.033857662	-0.004874887530	-0.1699921288	0.035178722	0.023954365	0.003803964
0.066964888	-0.009503217134	-0.1901323758	0.071795197	0.045991640	0.008679191
0.160085779	-0.021189729172	-0.1919570409	0.159583421	0.105737382	0.016913123
0.032899356	-0.005985066976	-0.2167766132	0.034264377	0.022710046	0.003816017
0.893178479	-0.028517892361	-0.0462878243	0.895267483	0.831619881	0.021854970
0.024405410	-0.004719033455	-0.2128344193	0.025440241	0.013077244	0.003949649
0.037433164	-0.005915256703	-0.1921623773	0.038599703	0.019651419	0.005423863
0.479945870	-0.075876934551	-0.2427513944	0.513406030	0.303858199	0.065815672
0.041837226	-0.006659316424	-0.1983709961	0.047495823	0.023243863	0.007762998
0.442548073	-0.047013042443	-0.1582445124	0.463474510	0.349594681	0.037182085
0.056106818	-0.010954410482	-0.2613752092	0.051717889	0.032365373	0.006041216
0.185166215	-0.030979505730	-0.2493659831	0.219149254	0.132958288	0.027435049
0.920430629	-0.020870042922	-0.0327288505	0.932209418	0.868797614	0.021096666
0.025954451	-0.004917291729	-0.2121738584	0.026318828	0.013403888	0.004941202
0.751017691	-0.032230953674	-0.0624332869	0.755251436	0.674179888	0.027559254
0.057999219	-0.018130425292	-0.4473808667	0.067629214	0.023672319	0.013748579
0.037742636	-0.005927878606	-0.1912660291	0.040179838	0.024111031	0.006160942
0.560071546	-0.048579951111	-0.1284989934	0.567634602	0.459676440	0.036035014
0.918094657	0.0224450821490	0.03447498847	0.962189566	0.897683038	0.019974115
0.075396825	-0.030879932469	-0.6474790987	0.058949213	0.032943095	0.009534318
0.198316420	-0.057624108500	-0.4671707474	0.190322553	0.109629590	0.026689712
0.043322332	-0.007855936636	-0.2299392454	0.045175354	0.024958698	0.006175652
0.086081929	-0.027767602369	-0.4920769444	0.098842612	0.040850867	0.018399522
0.331115573	-0.037949720501	-0.1701533572	0.348839746	0.250639841	0.029513923
0.018859669	-0.002751278454	-0.1445412264	0.021961806	0.013623229	0.002592149
0.428536002	-0.073118368700	-0.2631490331	0.471316636	0.269354617	0.078641870
0.296994116	-0.028114969040	-0.1385709428	0.298866931	0.245510878	0.018528368
0.192954094	-0.025927807746	-0.1971898428	0.215335038	0.136153396	0.025436108

0.618565330	-0.058074478613	-0.1398589478	0.644583706	0.473845568	0.054773393
0.484394349	-0.057925991143	-0.1797851848	0.544788701	0.348366657	0.053536640
0.339689793	-0.034879763857	-0.1515941835	0.346260688	0.287458359	0.019773099
0.075381477	-0.0110706040981	-0.2003498013	0.081286375	0.048637999	0.011816177
0.239133708	-0.035945522148	-0.22479274081	0.259989442	0.121410949	0.039456746
0.530002641	-0.048843818881	-0.1367768501	0.532518507	0.412750795	0.036434921
0.056386274	-0.009078858499	-0.2121631441	0.058794282	0.035519948	0.007128253
0.097822912	-0.0169755253011	-0.2471468571	0.093775801	0.060535726	0.009281804
0.032947739	-0.005358904433	-0.1922779930	0.031961454	0.018863758	0.004272312
0.061990503	-0.008335217103	-0.1775262786	0.059591696	0.046223189	0.004131447
0.896504535	-0.019243408861	-0.0309554767	0.902423662	0.831892767	0.022217282
0.894818732	-0.0228109667251	-0.0368374395	0.905781086	0.831531512	0.026853411
0.046504156	-0.012003295794	-0.3445237561	0.043095281	0.025954658	0.005505603
0.774558244	-0.036030492409	-0.06782474881	0.774012077	0.710887428	0.022137127
0.036367789	-0.005996824266	-0.1998048706	0.044362317	0.024286411	0.005843447
0.028238316	-0.0058657836491	-0.2402487587	0.035430233	0.013292422	0.006373383
0.081827007	-0.022607618724	-0.4077423276	0.079738839	0.042585344	0.009952643
0.079727288	-0.016359841037	-0.2904067213	0.085537731	0.047112551	0.012694458
0.333073365	-0.060649704860	-0.2806561683	0.368485353	0.191344487	0.050883221
0.101115288	-0.020752445139	-0.2982557603	0.115551898	0.051957669	0.019760574
0.940141768	-0.011587871328	-0.0177031954	0.946275846	0.901469954	0.013140195
0.878231325	-0.0234688578911	-0.0386315434	0.894519601	0.813014938	0.025937041
0.596243086	-0.038995103358	-0.0959168156	0.600285215	0.510201366	0.027682192
0.035605198	-0.006353553408	-0.2164451624	0.040320436	0.022657458	0.005838681
0.658375476	-0.048528319381	-0.1087462827	0.684852724	0.556122951	0.043029470
0.031837117	-0.005835917760	-0.2167384728	0.029996966	0.019487224	0.003776925
0.153671623	-0.033816729610	-0.3339038302	0.189608498	0.096077780	0.027963061
0.020953690	-0.003089625302	-0.1517056757	0.024284323	0.013963178	0.003260617
0.043230605	-0.006518364479	-0.1884553159	0.048933422	0.030646896	0.006487209
0.062458963	-0.014137889662	-0.3131468855	0.066778263	0.024351002	0.013545717
0.872060188	-0.026766299078	-0.0444568704	0.869322239	0.819485638	0.015813827
0.898879003	0.0325106662084	0.05070377652	0.959121648	0.889433977	0.022953741
0.057266688	-0.008711499485	-0.2000952117	0.064311597	0.039687644	0.007938647
0.035364711	-0.007076261882	-0.2446612424	0.037636903	0.017983793	0.006084226
0.039254286	-0.005843471156	-0.1821947650	0.036905388	0.028847820	0.002984095
0.830555057	-0.0393125997701	-0.0691034496	0.828654289	0.755952413	0.024456710
0.922824081	-0.018106967837	-0.0282793901	0.929158882	0.872066504	0.018406424
0.503224984	-0.043440821636	-0.1275933257	0.544234671	0.432655950	0.034326542
0.417926599	0.0440909947169	0.14147727806	0.524848384	0.381575900	0.049121917
0.050072151	-0.008189139916	-0.2114341095	0.052637260	0.034655290	0.006104863
0.261863087	-0.037330459086	-0.21309163561	0.293004443	0.145648553	0.043480249
0.042337786	-0.008601735527	-0.2590299459	0.049911033	0.019998941	0.008125264
0.561445142	-0.039923793887	-0.1044873704	0.583682774	0.474291637	0.038896672
0.033663041	-0.0051566889681	-0.1813161575	0.032226472	0.025243551	0.002529868

0.707901403	-0.042613135911	-0.0882822256	0.738083846	0.609406891	0.045373520
0.873021186	-0.020671743599	-0.0341754537	0.873298485	0.819878082	0.018950105
0.052160658	-0.013579786042	-0.3556133866	0.052377543	0.031517935	0.006202192
0.532855723	-0.047465555931	-0.1320035849	0.534336725	0.411662531	0.035533393
0.791026080	-0.038059148018	-0.0702286849	0.801039693	0.728151444	0.025370524
0.046673952	-0.007263762781	-0.1978772245	0.050959221	0.028246081	0.006365201
0.099550613	-0.023291790239	-0.3448536238	0.093655921	0.047388089	0.015346233
0.785870315	-0.034373649833	-0.0636956477	0.801866688	0.711873839	0.029135652
0.019858714	-0.002967210108	-0.1510015877	0.021749498	0.014839704	0.002207032
0.869176617	-0.030593368768	-0.0510968173	0.866417233	0.791796398	0.024574012
0.077788228	-0.012633590961	-0.2241653723	0.071737681	0.054556503	0.005386896
0.793571126	-0.031850172523	-0.0583464480	0.804434982	0.693024476	0.030759078
0.801895441	-0.044231390735	-0.0808188553	0.834492295	0.671376676	0.047123668
0.720459772	-0.090135712606	-0.1900026043	0.797549094	0.481279686	0.089579789
0.466562856	0.2614494717985	0.63097840500	0.914691366	0.382418716	0.241199949
0.042456627	-0.006082341443	-0.1777999853	0.050210015	0.029926200	0.006957852
0.124444561	-0.018456078169	-0.2130298319	0.126585589	0.087818529	0.014976933
0.884177957	-0.023123972793	-0.0377998477	0.877994883	0.828706623	0.015844426
0.065039207	-0.007735350552	-0.1569554080	0.063280855	0.050912953	0.004229299
0.024063099	-0.004928145588	-0.2254586850	0.026190919	0.013033560	0.004015330
0.893809315	-0.020837550938	-0.0336511223	0.899440945	0.856171295	0.015329261
0.969154684	0.0056792948538	0.00834374811	0.982017122	0.963121517	0.005761969
0.051530172	-0.007758790150	-0.1944554772	0.051523916	0.036747574	0.005511843
0.896535004	-0.019488641173	-0.0313532165	0.904690823	0.845718862	0.018158836
0.026767659	-0.004998812227	-0.2108245998	0.026761260	0.017052147	0.003455987
0.122642255	-0.027018859624	-0.3286110130	0.188041587	0.044940423	0.044763688
0.055480141	-0.008936303548	-0.2116876291	0.059557493	0.031528863	0.008413864
0.953664602	0.0096905438885	0.01443518194	0.969437737	0.955680246	0.005452763
0.783977090	0.0536222850962	0.09428514458	0.926851808	0.795467768	0.043471456
0.397777801	-0.036674856301	-0.1359637897	0.389162950	0.326147067	0.024841714
0.067065098	-0.010349249715	-0.2080481118	0.070433141	0.043000886	0.007317501
0.715060278	-0.049334727561	-0.1016635316	0.724016300	0.577977863	0.045531147
0.669005789	-0.042536767497	-0.0933335780	0.682346277	0.550642286	0.036927121
0.510005891	-0.053512533194	-0.1566714276	0.522378893	0.400386044	0.035642000
0.039705683	-0.007092982715	-0.2221273330	0.037927840	0.024452639	0.004784551
0.096446532	-0.021874901183	-0.3318832437	0.095955466	0.056336352	0.011293937
0.052705958	0.0132094362708	0.27578993742	0.088385285	0.043953081	0.013495186
0.034756739	-0.007217136376	-0.2536915407	0.037725254	0.019487400	0.006080948
0.489516968	-0.050659680676	-0.1542768676	0.478300915	0.364367324	0.035379707
0.900290471	-0.019533423340	-0.0312949854	0.914206137	0.853034462	0.020447127
0.051722657	-0.009275395829	-0.2349327279	0.051907580	0.027112870	0.009432946
0.671878552	-0.044495515813	-0.0973541839	0.686155956	0.559765512	0.042744651
0.040017825	-0.007861832190	-0.2467045836	0.041351296	0.023578381	0.006204039
0.321098994	-0.048578661530	-0.2289079276	0.335850030	0.214717433	0.037033240

0.173200044	-0.037784944738	-0.3332427682	0.184841069	0.088074068	0.034601271
0.027810849	-0.004347435031	-0.1762156044	0.029671339	0.018346936	0.003456574
0.887676538	-0.018222567771	-0.0295876183	0.902355760	0.845074304	0.019300799
0.855578078	-0.023623330197	-0.0399212658	0.857785817	0.810307941	0.014926966
0.168753754	-0.034976050292	-0.3141335707	0.208971415	0.091491872	0.035980657
0.059695898	-0.007863536837	-0.1727116492	0.059198071	0.036265225	0.006942601
0.043704014	-0.011154214373	-0.3358775812	0.041858073	0.024926451	0.005954842
0.033864585	-0.005845872663	-0.2063469488	0.036705921	0.017229553	0.005783751
0.861127327	-0.025208887295	-0.0423650327	0.869817146	0.803301055	0.023131724
0.425123397	-0.038608841426	-0.1340507672	0.429550706	0.339174219	0.025861641
0.048052561	-0.012197815650	-0.3402886700	0.052539582	0.028460260	0.007512618
0.408406669	-0.066520607817	-0.2497974790	0.428108297	0.272756120	0.048083085
0.068219321	-0.010438107409	-0.2066395420	0.067682843	0.048819716	0.006412453
0.158356599	-0.026149084183	-0.2435225652	0.155131378	0.086457335	0.022912970
0.621844187	-0.040819039564	-0.0963493164	0.624688511	0.503726747	0.034169196
0.690227907	-0.049828294265	-0.1064981618	0.737059834	0.581834837	0.053924012
0.353286026	-0.062039434771	-0.2701609560	0.421982030	0.146330129	0.088161863
0.143853729	-0.025488261742	-0.2613022685	0.131266752	0.102911548	0.008205346
0.096950695	-0.025189927356	-0.3874652081	0.147696099	0.048416082	0.030695873
0.059782839	-0.012368823808	-0.2814693398	0.067204101	0.034152602	0.009154092
0.022525731	-0.003611177759	-0.1697856664	0.021238834	0.014757553	0.002451771
0.468894610	-0.060672972026	-0.1954404915	0.482830952	0.305110802	0.056032438
0.070700635	-0.013802631461	-0.2706168658	0.072063746	0.041673955	0.009936246
0.051331777	-0.011366273894	-0.2957023697	0.046641463	0.029806516	0.006011693
0.033709575	-0.007133295925	-0.2570410294	0.035136839	0.018589695	0.005983980
0.081266914	-0.016511355860	-0.2879110983	0.081396254	0.053025300	0.009710679
0.363564549	-0.031456024306	-0.1269034967	0.366332313	0.287289353	0.028086867
0.092427316	-0.019602819277	-0.3064710750	0.097086730	0.048135271	0.017718645
0.056183882	-0.008949847742	-0.2096066121	0.056721023	0.032200884	0.007710715
0.125641727	-0.025741812232	-0.3036107884	0.140275281	0.079466116	0.021192356
0.033688244	-0.006841353506	-0.2457022397	0.037046194	0.018876045	0.005314204
0.079404306	-0.018740029987	-0.3393632642	0.079689558	0.028869295	0.015146571
0.412634174	-0.051268595899	-0.1865693294	0.414290761	0.321193562	0.029127639
0.645707762	-0.059469497329	-0.1371640069	0.706637053	0.499913340	0.053327059
0.459206254	-0.041329333826	-0.1330263811	0.467097474	0.342768552	0.046574460
0.863858177	-0.032537243340	-0.0547429144	0.888726312	0.787273335	0.032750461
0.070132632	-0.012281524508	-0.2400174963	0.064137377	0.049456929	0.004536661
0.076359625	-0.014780786581	-0.2708238439	0.084547053	0.041871321	0.014857854
0.025283194	-0.004227577652	-0.1841269651	0.024912724	0.016842660	0.002486771
0.187550937	-0.023206893563	-0.1802876138	0.179149331	0.145943320	0.012837071
0.901880720	-0.016788502374	-0.0268087978	0.907898636	0.858695962	0.016588171
0.021590533	-0.003084723703	-0.1482363136	0.022373624	0.015272486	0.002034762
0.022487663	-0.003715846462	-0.1752356515	0.023028492	0.015657066	0.002658813
0.055669790	-0.011795783269	-0.2856404724	0.061877920	0.026730113	0.011693849

0.052493861	-0.007187549111	-0.1762703364	0.057265096	0.035439628	0.006206969
0.032696879	-0.006507019062	-0.2385450930	0.038832896	0.017381091	0.005770777
0.034618811	-0.006356259625	-0.2217189875	0.036192869	0.020857572	0.004670771
0.816954458	-0.033806112336	-0.0602171553	0.820086520	0.741559364	0.026645337
0.046274406	-0.005922739779	-0.1604394008	0.047118057	0.032233355	0.004868925
0.841347450	-0.033772957780	-0.0583978556	0.836528606	0.750335983	0.027520699
0.840124474	-0.027930292032	-0.0481949309	0.825238362	0.799676483	0.011036561
0.094701771	-0.020638880311	-0.3167448695	0.089430336	0.049607125	0.013588847
0.758504802	-0.054428779309	-0.1059765984	0.795805932	0.622973049	0.048158813
0.048829646	-0.019656840607	-0.5866909014	0.049186840	0.019361955	0.009321455
0.521073916	0.1218432176865	0.29798722814	0.903975698	0.446134542	0.150983325
0.029620180	-0.005108750376	-0.1991611921	0.036620213	0.019342566	0.004993928
0.055041217	-0.008941123995	-0.2133510872	0.057642643	0.030688875	0.007826829
0.073258860	-0.015163111244	-0.2900390711	0.070409654	0.036760477	0.009721112
0.758718195	-0.037874235429	-0.0728914030	0.784800580	0.665354564	0.035023764
0.023833961	-0.004542583699	-0.2079960501	0.024498687	0.013448508	0.003174691
0.837192247	-0.032781133751	-0.0569321384	0.848865964	0.742409620	0.038603635
0.883833328	-0.024248639733	-0.0396793014	0.897939582	0.819048199	0.025515341
0.040672619	-0.008723953958	-0.2725814944	0.039855076	0.021327095	0.005708400
0.401185768	-0.071621722888	-0.2761066236	0.395505357	0.256304045	0.050946415
0.057567417	-0.011909329619	-0.2797363696	0.073329166	0.035371514	0.011191757
0.055084550	-0.011311406030	-0.2754292857	0.060163617	0.032187690	0.008864504
0.927630210	-0.017391979957	-0.0270116609	0.935399197	0.875613162	0.020326953
0.035451576	-0.007744040289	-0.2694774454	0.051073836	0.017421237	0.009410129
0.735796234	-0.045094026815	-0.0899800713	0.734285635	0.625747604	0.037258707
0.221125015	-0.030333581318	-0.2029756911	0.244382542	0.148260465	0.027140902
0.190410911	-0.023054201517	-0.1763071456	0.220750672	0.142775311	0.022850201
0.119448736	-0.022062121446	-0.2695667006	0.115196406	0.068686040	0.013885367
0.334748359	-0.076921698779	-0.3642441063	0.376619743	0.132695478	0.089642192
0.712396052	-0.060495585367	-0.1261757203	0.739543861	0.563829226	0.059770567
0.042956963	-0.007950838577	-0.2346990500	0.045598688	0.029817040	0.005839637
0.455204179	-0.045955528173	-0.1500579154	0.491775795	0.358145433	0.042383983
0.061466256	-0.009972013823	-0.2168108739	0.061862509	0.044254308	0.006277727
0.045347298	-0.006914525872	-0.1925293071	0.050756403	0.027981208	0.007095636
0.167411682	-0.021403453867	-0.1854788874	0.176916929	0.125203369	0.016284660
0.278069523	-0.049103566638	-0.2696119315	0.272953809	0.194048448	0.023837070
0.780794247	-0.045063012359	-0.0846466060	0.782707928	0.682755790	0.036930941
0.893016050	0.0132849655135	0.02106998817	0.920328902	0.897351608	0.008032118
0.036876207	-0.006759759478	-0.2246619803	0.038776915	0.026222734	0.004510215
0.519857194	-0.062389645389	-0.1807373625	0.551623222	0.357295313	0.058454605
0.097325240	-0.018102261388	-0.2665021809	0.103362069	0.054039432	0.018895390
0.829365378	-0.029735321443	-0.0520361070	0.865656516	0.752562821	0.031221107
0.227019098	0.0689476940739	0.36837176794	0.389231354	0.170239263	0.088205626
0.732838790	-0.065064169293	-0.1322435365	0.764155088	0.568570080	0.060947184

0.297864541	-0.026807005402	-0.1314302148	0.294083744	0.239576448	0.019998414
0.903422266	-0.016707450844	-0.0266327243	0.909260607	0.871080187	0.010845712
0.832787860	-0.025555687596	-0.0444235323	0.833378662	0.769909821	0.020881756
0.852535974	-0.0248845023551	-0.0422345337	0.855459534	0.792316378	0.025514139
0.037348649	-0.005946133199	-0.1936048255	0.038870811	0.028722378	0.003059434
0.188394588	0.0439790934296	0.28886037762	0.276035810	0.145704514	0.045383404
0.085983656	-0.010396729404	-0.1653983242	0.086973763	0.069469462	0.005420674
0.044712454	-0.008775573030	-0.2522163582	0.050690198	0.026033712	0.007333641
0.911921401	-0.017291032800	-0.02731533031	0.917829060	0.871640242	0.015609752
0.347918770	-0.049506237594	-0.2147708127	0.346415110	0.226525806	0.042341294
0.059184175	-0.007638626148	-0.1687875565	0.058585723	0.041675696	0.005510119
0.213810292	-0.043501610422	-0.3118075185	0.311092288	0.114247542	0.059896984
0.026754459	-0.005310058588	-0.2251161587	0.027181929	0.015983763	0.003794869
0.713383782	-0.0460270066441	-0.0948453275	0.733666828	0.548325224	0.055074767
0.186571881	-0.032841521447	-0.2637351049	0.217365303	0.134836918	0.025624325
0.713758260	-0.051502297798	-0.1064972627	0.735888778	0.603656078	0.048303011
0.037638345	-0.007982425944	-0.26458706941	0.034270039	0.023264713	0.003926849
0.218823816	-0.037227112992	-0.2561644795	0.270386787	0.144852816	0.036685639
0.088239393	-0.020109632233	-0.3304294496	0.096433568	0.030999746	0.018482737
0.037419207	-0.011041421920	-0.3824138240	0.039609632	0.020335132	0.005663909
0.966641093	0.0058145838506	0.00856384027	0.980591417	0.959899366	0.005940691
0.177778905	-0.023779777881	-0.1953468631	0.182809061	0.123763888	0.020759798
0.570193489	-0.038572562848	-0.0992506253	0.584982896	0.475463795	0.034502185
0.792083954	-0.042643167651	-0.0788157690	0.794150410	0.689301216	0.030145074
0.190639807	-0.046857253154	-0.3837160213	0.192644023	0.082920558	0.032349878
0.274385012	-0.035790832433	-0.1940529133	0.275831410	0.202794701	0.022694109
0.066460480	-0.009606627542	-0.1937035568	0.065316996	0.047716273	0.005928112
0.038356347	-0.012765706461	-0.44220736331	0.036525168	0.020486869	0.004785749
0.151414895	-0.048506682287	-0.5156232823	0.163260983	0.066863563	0.036235791
0.902298522	-0.021229247956	-0.0339683847	0.914918803	0.842546183	0.021877949
0.059918159	-0.008499124368	-0.1869813604	0.064962173	0.041638165	0.006221387
0.064018437	-0.011108712164	-0.23460161811	0.069887212	0.037157869	0.008684871
0.756159425	-0.044162108095	-0.0856511483	0.808776787	0.647753926	0.049321044
0.081440870	-0.010961841461	-0.1842262383	0.079875226	0.059605665	0.006456805
0.563143444	-0.057496389423	-0.1525123353	0.563194921	0.444523881	0.040737530
0.512225821	-0.058496240656	-0.1713900357	0.594075033	0.379455996	0.066191578
0.864157084	0.0261901807185	0.04258900549	0.916490328	0.849154721	0.020865585
0.172072353	-0.030930976924	-0.2686132075	0.208384635	0.084042415	0.032388050
0.659129192	-0.053490596068	-0.1202011050	0.690365074	0.502916256	0.072370677
0.174947488	-0.023447351320	-0.1955803185	0.194573228	0.125325128	0.023550017
0.401518004	-0.036515787992	-0.1340564291	0.383713694	0.324523479	0.017687184
0.606827091	0.0275974133677	0.06314522408	0.652737009	0.620787224	0.012258330
0.796626900	-0.026955641889	-0.0490355667	0.807918423	0.720736405	0.028020048
0.304106988	-0.028074618421	-0.1350776166	0.306028115	0.244236286	0.018431577

0.022751838	-0.003153474287	-0.1460584460	0.024235467	0.015692800	0.002386039
0.096977670	-0.028155313158	-0.4406328926	0.085428243	0.047403129	0.012242712
0.153198920	-0.042697633610	-0.4375829599	0.163884233	0.077786338	0.026443069
0.650160209	-0.049384156610	-0.1121726856	0.679573534	0.516156820	0.054451066
0.511182605	-0.049569502712	-0.1441851336	0.505185321	0.414968984	0.034201082
0.233395923	-0.025528197196	-0.1598525056	0.262670546	0.177113223	0.024996890
0.811733611	0.0447446401672	0.07649275985	0.903659501	0.767119072	0.043355163
0.446210094	-0.080713757419	-0.2808995349	0.474945396	0.275169641	0.071952595
0.056146609	-0.011283572816	-0.2698327324	0.054335050	0.033235098	0.007057821
0.818510834	-0.035398957322	-0.0629961754	0.844072690	0.735395054	0.031223537
0.825309088	-0.023909032998	-0.0418967339	0.835166757	0.761731170	0.023804795
0.244385178	-0.037533857805	-0.2303083861	0.251777438	0.148721199	0.032257127
0.728279485	-0.057413706859	-0.1167966480	0.762750123	0.590681160	0.058845708
0.859454628	-0.059656913324	-0.1025491715	0.924433585	0.724603091	0.066055068
0.036694417	-0.006883980365	-0.2301033697	0.035756538	0.021074015	0.004810570
0.596399573	-0.062069832232	-0.1557878742	0.623291941	0.472363486	0.052644278
0.050662090	-0.011139560429	-0.2927101933	0.058158331	0.024830858	0.009301028
0.071530745	-0.008008482414	-0.1491630323	0.070013659	0.055811693	0.004475048
0.030141587	-0.004890247976	-0.1874195912	0.033392514	0.020025646	0.004276198
0.672233822	-0.055049613612	-0.1213770439	0.698319545	0.540239157	0.056098666
0.936901778	0.0155476000179	0.02349587832	0.964214563	0.924112546	0.012435098
0.102786074	-0.020469946354	-0.2889343290	0.115499744	0.064679483	0.016156092
0.633530305	-0.047784699958	-0.1113116856	0.627255100	0.558110451	0.025623120
0.193025257	-0.026466547099	-0.2015112205	0.192131506	0.141137920	0.016491675
0.499033064	-0.040679805808	-0.1201622672	0.502057951	0.407795129	0.033698182
0.029987961	-0.004443125784	-0.1699257723	0.030181784	0.021072173	0.002864562
0.874034543	-0.050769030007	-0.0853264715	0.926453189	0.735312180	0.058013331
0.038172825	-0.006147715192	-0.1969679380	0.041717075	0.024044142	0.005886452
0.634184435	-0.047769287498	-0.1111569030	0.688080456	0.511679599	0.051117104
0.063273576	-0.012867672773	-0.2786034786	0.060674090	0.039284299	0.007733493
0.339714303	-0.040519601342	-0.1776612044	0.353707386	0.248936843	0.039540327
0.298018857	0.0728910551638	0.30643115407	0.557188984	0.248775541	0.103868602
0.057773041	-0.013388697343	-0.3175201203	0.082712582	0.026537115	0.016248424
0.035323306	-0.007035440152	-0.2433658468	0.033534051	0.022378425	0.003707815
0.319514508	-0.033582014129	-0.1550738703	0.361331713	0.259642014	0.036598277
0.022788788	-0.003476003067	-0.1616725309	0.022321852	0.017082664	0.001488741
0.751747872	-0.029227004130	-0.0564437053	0.762979172	0.678487385	0.024902175
0.032167315	-0.005065847073	-0.1846488909	0.032332884	0.019645886	0.004080370
0.731583215	-0.058252494600	-0.1180246029	0.795684262	0.568261354	0.060927560
0.181942763	0.0461368554103	0.31076795331	0.289165151	0.134610375	0.053871041
0.034533849	-0.004461187296	-0.1522839665	0.037626030	0.024653114	0.003816440
0.028390405	-0.004239493910	-0.1688217113	0.032406391	0.020297493	0.004066955
0.082915640	-0.009866096656	-0.1619492216	0.080622985	0.064681468	0.005553993
0.774774678	-0.047446481875	-0.0899715633	0.784604293	0.635247985	0.046842605

0.481665783	-0.047080036479	-0.1452162595	0.476442122	0.371083806	0.037342224
0.883676219	-0.021944813778	-0.0358685488	0.895907216	0.822044331	0.024539486
0.534784937	-0.048219255677	-0.1337022565	0.566739258	0.433477402	0.039677644
0.529600490	-0.039537474515	-0.1097817506	0.538329232	0.446202161	0.029533282
0.053403050	0.0307333808487	0.57020090680	0.160186258	0.037157876	0.044199975
0.562386532	-0.047250544910	-0.1242986607	0.560106546	0.465619832	0.036915202
0.591758980	-0.061361547481	-0.15516494551	0.607889591	0.431758930	0.050139811
0.824956668	-0.048191563860	-0.0857683571	0.871448739	0.742681478	0.041483476
0.410622475	-0.039900120706	-0.1437866582	0.433782759	0.313723116	0.037304735
0.032664587	-0.005109398975	-0.1840268611	0.035965150	0.024436318	0.003727703
0.088652294	-0.021464507255	-0.3539800059	0.089791775	0.044455657	0.014097039
0.061048958	-0.015491810049	-0.3548409494	0.056927367	0.033497686	0.008090559
0.742022408	0.0399998539298	0.07476533114	0.835732052	0.724479308	0.033459697
0.171663459	-0.027951885732	-0.2410424620	0.194583318	0.091488654	0.029592838
0.760957078	-0.034394164334	-0.0658417836	0.776493594	0.655205873	0.041817183
0.180844503	-0.038933299978	-0.3291693635	0.189228005	0.088672837	0.032623157
0.022895385	-0.004328247603	-0.20352871621	0.022609147	0.015391735	0.003102883
0.364276796	-0.039048529375	-0.1589618049	0.386909365	0.238456456	0.046938630
0.365688521	-0.045581663530	-0.1866039265	0.363844765	0.260230099	0.033544274
0.734613533	-0.067056901364	-0.1361503016	0.777723809	0.588872050	0.064306749
0.833618817	0.0366660536179	0.06137919757	0.914975861	0.811597156	0.037171025
0.045373582	-0.007695105886	-0.2158597213	0.058853203	0.028882932	0.009102603
0.043172194	-0.008398205717	-0.2480111607	0.046904734	0.022082291	0.008234692
0.069989716	-0.012708122695	-0.2496026587	0.069914782	0.038490686	0.011695104
0.703619261	-0.064357538234	-0.1363543971	0.752631267	0.530349652	0.075337639
0.433013427	0.0381618887421	0.11921220906	0.497539260	0.440985556	0.019107881
0.177867570	-0.030391234514	-0.25458100091	0.210491516	0.105262198	0.033044960
0.135229163	-0.031919345637	-0.3580583189	0.136090702	0.083875029	0.016175095
0.047199046	-0.008634443443	-0.2360859291	0.048651847	0.028124774	0.007058632
0.211087497	-0.020749985498	-0.1421848608	0.211700023	0.174290049	0.011887755
0.085367125	-0.012103685170	-0.1958088638	0.087945069	0.055932953	0.011214231
0.501472980	-0.038888411706	-0.1140855068	0.509133819	0.410016269	0.029381332
0.040768590	-0.006585304516	-0.2004354884	0.042343003	0.028017034	0.004496272
0.507739176	-0.049796168692	-0.14589265851	0.559700337	0.362352074	0.057603504
0.917078393	-0.016367754924	-0.0256986006	0.924995714	0.861755177	0.020170849
0.911213607	-0.016217242875	-0.0256237248	0.908458125	0.867422583	0.011706850
0.402799909	-0.033410772390	-0.1217642832	0.422654394	0.316815220	0.031693030
0.023142431	-0.005191092790	-0.2457620680	0.022264778	0.014297009	0.002578929
0.881904117	-0.026298934187	-0.0431794860	0.892087423	0.801144420	0.032525615
0.044086388	-0.007461843091	-0.2141759101	0.048009469	0.022692955	0.008194290
0.837411656	-0.033906288262	-0.0589111075	0.869063744	0.744502386	0.037232331
0.674911387	-0.045547904377	-0.0992809993	0.670727291	0.581785965	0.033672646
0.088730303	-0.023766707226	-0.3973027967	0.115950534	0.049637437	0.021430583
0.040232064	-0.006273109511	-0.1924514807	0.041686984	0.028186620	0.004955566

0.049381574	-0.0083260485905	-0.2179482296	0.050657571	0.032631376	0.005394326
0.646678242	-0.058495818428	-0.1346011341	0.682558677	0.517454949	0.062133876
0.947975546	0.0178513155371	0.02663636767	0.973169874	0.962059578	0.003502657
0.029086202	-0.0048529519385	-0.1912611352	0.032868263	0.020001194	0.004038658
0.037599321	-0.007754298074	-0.2565414632	0.037448304	0.024520842	0.003944491
0.800473691	0.060864703380	0.1044677602	0.915611782	0.761155589	0.050488002
0.051201847	-0.010162541509	-0.2619664922	0.052627080	0.024205381	0.008550376
0.683066608	-0.057323442775	-0.1245499640	0.673568898	0.570778081	0.042668351
0.047155979	-0.008188549593	-0.2230819601	0.050945048	0.029661890	0.007003786
0.040234760	-0.006548085246	-0.2014926855	0.040691658	0.025451476	0.005291129
0.407119503	-0.031651090139	-0.1138481252	0.414830197	0.337740987	0.021446735
0.050885401	-0.008254851873	-0.2101958919	0.050992558	0.034457527	0.007091488
0.444398173	-0.032824832266	-0.1081733966	0.451723553	0.385646014	0.020118928
0.168539524	-0.044872783083	-0.4176029463	0.197050747	0.079060121	0.037031773
0.142517647	-0.021238619154	-0.2163397173	0.137998478	0.092448276	0.012789716
0.374632606	-0.039515558917	-0.1563953301	0.376652499	0.282322751	0.030230995
0.602687930	0.0719193160950	0.1601241318	0.783230054	0.562059684	0.070935010
0.852648036	-0.026120233140	-0.0443585633	0.869065135	0.791825196	0.025852377
0.304379743	-0.045812592650	-0.2272254075	0.319932549	0.183283706	0.046498968
0.799663227	-0.036735950499	-0.0669892755	0.820502757	0.680033683	0.043305869
0.052874364	-0.008181682562	-0.2011241042	0.052321274	0.036149662	0.005663377
0.030114426	-0.004184083466	-0.1589184634	0.031813066	0.022684756	0.003373757
0.082415705	-0.020990187664	-0.3716984603	0.089655620	0.046065037	0.014264602
0.047603339	-0.007567773348	-0.2031984988	0.050233269	0.028555899	0.007050113
0.053405511	-0.015345265477	-0.3997642192	0.053088371	0.027262908	0.009457615
0.279621363	-0.041001042556	-0.2202238053	0.357825180	0.183747570	0.056918927
0.093004262	-0.017766000604	-0.2731309585	0.103025445	0.064727237	0.011845002
0.035945680	-0.007455051278	-0.2554219771	0.041527825	0.020897375	0.006393048
0.162927861	-0.035520010952	-0.3317058972	0.174903386	0.054854054	0.044194055
0.662737582	-0.052779699412	-0.1178736474	0.715520765	0.519208678	0.062185538
0.040054819	-0.005780690667	-0.1770450664	0.042322542	0.027303211	0.005529221
0.924708220	-0.013664254418	-0.0212460570	0.933816348	0.881950281	0.016055669
0.108301195	-0.026537707933	-0.3664725237	0.130991561	0.057917209	0.022343390
0.055688092	-0.018966782154	-0.4915511349	0.044092287	0.019389947	0.007442491
0.640604811	-0.040921004362	-0.0937202624	0.677531491	0.543384535	0.043267539
0.213004157	-0.030101463256	-0.2091973135	0.209474186	0.130752981	0.027614103
0.086594535	-0.019673348461	-0.3285605491	0.085166514	0.053531555	0.012066176
0.378131851	-0.042876359021	-0.1688824648	0.393168761	0.280579297	0.034040175
0.367066998	-0.067151667549	-0.2829467647	0.447557200	0.251763020	0.060932451
0.769745928	-0.039761643849	-0.0755094825	0.777893027	0.684961059	0.034245464
0.055024224	-0.013117496096	-0.3250557270	0.055210195	0.033347246	0.008030449
0.058479203	-0.010050130794	-0.2289795282	0.056601374	0.037189599	0.006320023
0.433982516	-0.038326278589	-0.1302451927	0.440639671	0.362541450	0.027396725
0.811609493	-0.033097311152	-0.0593198072	0.824203736	0.731500906	0.030306383

0.046646035	-0.012698669604	-0.3661982914	0.046891961	0.021871873	0.009385703
0.035914668	-0.005758617232	-0.1933377184	0.039414513	0.023440274	0.006269519
0.033107556	-0.005701002067	-0.2046497063	0.036463957	0.017928332	0.006239328
0.035765025	-0.004573358599	-0.1518929747	0.036331956	0.024098175	0.003842450
0.046234083	-0.009934088853	-0.2804327867	0.060167900	0.026045309	0.010913517
0.057223772	-0.009156432580	-0.2112445606	0.058695078	0.037246030	0.008255140
0.062711520	-0.016752037422	-0.3778013313	0.057305495	0.035551518	0.008379666
0.045324511	-0.012530776997	-0.3705192250	0.040676338	0.022594357	0.005522917
0.019318714	-0.004073130748	-0.21579081361	0.019477605	0.011609539	0.002974483
0.426275505	-0.070968046369	-0.2561282645	0.466873313	0.278700993	0.057647985
0.071780166	-0.011903842904	-0.2269472946	0.091739328	0.046669894	0.013048418
0.071260487	-0.016233282910	-0.3215106136	0.073607411	0.043102678	0.009679530
0.045826106	-0.009262129115	-0.2617256436	0.047204789	0.024633963	0.007399965
0.579747178	-0.047722065725	-0.1217369161	0.612650783	0.473752631	0.043156725
0.921855351	0.017902864780	0.0274542595	0.956282926	0.925382831	0.011483981
0.749957827	-0.073694933584	-0.1471580103	0.813982326	0.503426414	0.099831540
0.707875535	-0.038859770657	-0.0802886626	0.703835285	0.573458595	0.043247554
0.713506921	-0.044587585123	-0.0917666430	0.717375039	0.613789030	0.039205560
0.896001042	-0.028556208913	-0.0462042867	0.919868115	0.816908900	0.036483605
0.769945037	0.062171172513	0.1106468741	0.905646980	0.723836506	0.055732257
0.017887224	-0.0029519798751	-0.1614180006	0.018245832	0.010651628	0.002619006
0.932403881	0.015835349888	0.0240403989	0.966837393	0.909386331	0.017212263
0.921976858	-0.013778917971	-0.0214889362	0.926875434	0.886039868	0.014290882
0.056364419	-0.010123443658	-0.2387883367	0.064488974	0.033367491	0.008735330
0.037788886	-0.008427552978	-0.27989605531	0.045077258	0.021309533	0.007481895
0.497935738	-0.046489865953	-0.1384845557	0.518325448	0.420315046	0.032550508
0.041871288	-0.014019236493	-0.4545646946	0.037636770	0.019188239	0.006079174
0.079626345	-0.013750424811	-0.2402807553	0.073901980	0.053931760	0.006678633
0.075746914	-0.015829191906	-0.29442656601	0.071996956	0.049764854	0.007349279
0.032829099	-0.006039785185	-0.2193045559	0.032162187	0.022850120	0.003120788
0.053678984	-0.008129548606	-0.1970450670	0.053819891	0.034514805	0.006306661
0.028350330	-0.004009317604	-0.1593064165	0.028110716	0.018418390	0.003239881
0.654763572	-0.039614144970	-0.0886402540	0.665701282	0.564785003	0.037078322
0.848720879	-0.0241245382781	-0.0411106748	0.859556018	0.800406565	0.019934131
0.088147643	-0.025287328025	-0.42982038661	0.105685081	0.035566622	0.020653384
0.861228255	0.030898895743	0.0502800459	0.928255141	0.825729584	0.030532028
0.175274429	-0.029957315843	-0.25444697981	0.202593119	0.095758563	0.032574691
0.676163433	0.059828253050	0.1206073080	0.795890497	0.646295640	0.049432045
0.042409076	-0.009454644291	-0.2870096842	0.046854450	0.027217907	0.006150562
0.051369951	-0.014499376935	-0.3888499612	0.051516062	0.024751646	0.008173525
0.362605158	-0.034837863309	-0.1416180036	0.355475346	0.295286014	0.023325478
0.553470632	0.055135857534	0.1346815487	0.670486754	0.522770750	0.052339143
0.764158120	-0.0414464873351	-0.0793827431	0.774667177	0.678506649	0.035207325
0.035107997	-0.007197866847	-0.2507998119	0.034641692	0.020672994	0.005178022

0.058691978	-0.011342394552	-0.260358618	0.070570675	0.038161217	0.009603676
0.456902637	-0.037738833417	-0.121593348	0.455760797	0.383813759	0.024322000
0.035598547	-0.005131571490	-0.172242823	0.043135348	0.024534462	0.005696301
0.023206430	-0.003688628910	-0.169877372	0.028268543	0.013120389	0.004256471
0.533083053	-0.039823857116	-0.109871214	0.527146302	0.426819126	0.028558379
0.664595503	-0.040058132595	-0.088317568	0.707357646	0.558720929	0.044493910
0.038015278	-0.005890298650	-0.188817549	0.042828177	0.026304407	0.006155704
0.729425702	-0.043979504623	-0.088466285	0.750298275	0.619773962	0.044855057
0.471118410	-0.047371218372	-0.149537588	0.507219889	0.358194380	0.052473120
0.630870994	-0.052302046297	-0.122822545	0.629251854	0.475214842	0.059481214
0.070925900	-0.008852376158	-0.167132119	0.069846055	0.052488777	0.005131920
0.606752241	-0.040571626041	-0.098169929	0.618194858	0.489432034	0.034551150
0.727814515	-0.039247257445	-0.078859147	0.759943184	0.637222606	0.038012831
0.758010783	-0.047885974143	-0.092879596	0.777705926	0.659212467	0.040080338
0.137394008	-0.021600928680	-0.228625322	0.130368373	0.095211540	0.012836045
0.134755618	0.028946951384	0.2629997524	0.206954775	0.141291763	0.024816738
0.051647775	-0.009221523906	-0.233759439	0.047208821	0.031585073	0.005226476
0.274966123	-0.027446601886	-0.146108621	0.273160273	0.197365089	0.024049657
0.330196404	-0.033811846641	-0.151024307	0.351639702	0.251253564	0.037245114
0.916174789	-0.015739078843	-0.024727340	0.926349456	0.876580530	0.017799512
0.174663618	-0.024102365086	-0.201775877	0.185467322	0.130599546	0.019223745
0.057712667	-0.011264901131	-0.262509258	0.077495829	0.028614429	0.014832196
0.112366096	-0.016301869193	-0.206265737	0.121886900	0.069412141	0.016740394
0.028831554	-0.004352616297	-0.171514114	0.034896108	0.019371100	0.005329222
0.618361003	-0.065630297710	-0.159148961	0.629028700	0.476808476	0.058499641
0.774396232	-0.040996143743	-0.077443745	0.807460332	0.658795286	0.044614220
0.852917537	-0.028260902663	-0.048039881	0.886410743	0.785523634	0.035731166
0.041407570	-0.006539358045	-0.196287124	0.049284133	0.027950673	0.006138444
0.050753617	-0.010456483310	-0.272494112	0.055010981	0.028903018	0.007069272
0.038748438	-0.006239378987	-0.197585667	0.044338787	0.020721953	0.007724696
0.523698067	-0.049883011665	-0.141568122	0.552455084	0.411593326	0.046514049
0.063141765	-0.017905879144	-0.405089570	0.057340949	0.033355367	0.007687842
0.044785152	-0.007591166449	-0.215181929	0.049734457	0.028482604	0.007267213
0.082766839	-0.017357560901	-0.298867142	0.089941090	0.048625667	0.012145472
0.055561141	-0.010257894698	-0.245476790	0.056183815	0.031230168	0.007925727
0.081848170	-0.010503024559	-0.175194726	0.076260617	0.063170474	0.004278977
0.034222247	-0.005661628357	-0.197644130	0.037272656	0.016367948	0.006717809
0.082133955	-0.013843348571	-0.234893723	0.083344786	0.051046171	0.010775426
0.636470575	-0.035968375554	-0.082588212	0.649172609	0.572062282	0.027665779
0.275670778	-0.034745426147	-0.187095228	0.271443999	0.198747279	0.025149522
0.070165369	-0.011119905391	-0.215432504	0.078975935	0.044154731	0.008901710
0.047875983	-0.009158322612	-0.248519939	0.044494355	0.031833352	0.004627989
0.597744519	-0.037502803771	-0.091891395	0.601098735	0.495896441	0.041000572
0.042470024	-0.007407011099	-0.219549673	0.042842670	0.026177577	0.005171426

0.031294985	-0.007908607051	-0.3067069975	0.031614045	0.018788166	0.003978104
0.618821998	-0.048399761379	-0.1155488962	0.647738338	0.497484814	0.047696811
0.900790958	-0.019205270662	-0.0307465118	0.904969231	0.845020712	0.020491156
0.028923772	-0.006478336366	-0.2626359835	0.030181811	0.015429496	0.004416452
0.367650641	-0.052091906060	-0.2141343876	0.383178727	0.205924667	0.056965581
0.405797054	-0.050628942616	-0.1873187500	0.434158279	0.293642802	0.048426129
0.192461050	-0.026084058150	-0.1989820096	0.182557512	0.131701555	0.015726797
0.038233324	-0.007746435112	-0.2525754457	0.043284661	0.023095204	0.007074008
0.067742398	-0.009721112437	-0.1927153349	0.075044938	0.050148513	0.007770794
0.508460341	-0.049680920600	-0.1453243996	0.520646910	0.339683027	0.054650292
0.385491103	-0.064571790843	-0.2571657511	0.388843121	0.242981366	0.051693826
0.259975474	-0.063782892798	-0.3888359242	0.302216678	0.144872883	0.044075292
0.898473952	-0.0219155350431	-0.03522945361	0.919553252	0.838895138	0.025007060
0.033408091	-0.004915579743	-0.1733861771	0.042413256	0.020123971	0.006255005
0.425934498	-0.029822610599	-0.1022341195	0.439292765	0.372597986	0.021959371
0.576155113	0.2066882156021	0.43575331448	0.923199333	0.273793268	0.198874080
0.053945728	-0.010331995345	-0.2542453962	0.050994171	0.028237614	0.006668998
0.041884648	-0.008407152480	-0.2550388843	0.048284752	0.023585416	0.007680084
0.355023014	-0.042087216382	-0.17674005251	0.358587692	0.271724357	0.031041713
0.048066379	-0.007019698548	-0.1858859227	0.049152772	0.030470791	0.006199048
0.032986309	-0.006732806528	-0.2457568557	0.036256884	0.018177502	0.005277283
0.019603948	-0.003201595531	-0.1651231013	0.022679108	0.013756765	0.002794789
0.622547786	-0.055467508780	-0.1324024476	0.642311474	0.448422998	0.068944796
0.059376113	-0.010142372439	-0.2280198143	0.056290675	0.042511548	0.004722681
0.692560362	-0.057506933772	-0.1232035223	0.722071803	0.561308183	0.051478180
0.136251306	-0.061403363339	-0.7854979220	0.110497180	0.048822380	0.020710788
0.044737344	-0.007712258553	-0.2190948674	0.052462523	0.024861736	0.008416018
0.496968544	-0.041598681650	-0.1235184506	0.482531897	0.387963562	0.030616644

max.DA	min.DA	sd.DA	diffmeth.p.val (Welch's t-test)
0.194714794	0.123219249	0.019807343	0.000113039148242169
0.073453261	0.034922162	0.010752983	0.000121653408648056
0.058927241	0.040873383	0.006625023	0.000138982836204649
0.068118692	0.037522355	0.010537844	0.0001859742928629
0.221593741	0.107406482	0.033986222	0.000243104600534497
0.044343168	0.024201196	0.005558411	0.000258411873712882
0.037673999	0.025665129	0.003783673	0.000267142044972077
0.065343144	0.051047165	0.005337240	0.000285136533999872
0.894105031	0.750981133	0.038152964	0.000296325684961978
0.041932456	0.022927441	0.006433255	0.000309332915541311
0.042322288	0.026463061	0.005242135	0.000330495830695972
0.031035333	0.021115199	0.003577638	0.000379806831804773
0.061376396	0.032052646	0.010014003	0.000391994431953747
0.891291274	0.824935309	0.019231959	0.000408080356572393
0.073413446	0.040513781	0.012164009	0.000455182455996829
0.031479724	0.019992892	0.003483115	0.000490669785530798
0.361610417	0.221836123	0.043180154	0.000492794652828944
0.043483223	0.026271408	0.006666281	0.000507871624212232
0.175216427	0.114932390	0.020313693	0.000522261063830755
0.240916759	0.104577866	0.047224880	0.000527443259687501
0.075837656	0.038569830	0.012048317	0.000541740208278194
0.790885264	0.559803375	0.064912409	0.000544194274203337
0.866099962	0.820095839	0.015783357	0.000544306602132412
0.063353968	0.033712933	0.008262963	0.000553813628802719
0.063651220	0.039336183	0.009169036	0.000557090186623979
0.071620520	0.026675308	0.012820547	0.000587559037741119
0.055439644	0.035198064	0.005881199	0.000606103895035033
0.650220410	0.569204044	0.022671633	0.000608274962484556
0.726955172	0.591404953	0.038734154	0.000625845124633777
0.052921326	0.029945914	0.007269543	0.000675561376767598
0.039892061	0.028169815	0.004000873	0.00068750647627362
0.925540661	0.796332743	0.035306636	0.000709048617889804
0.088805635	0.028519268	0.015342832	0.000713533022559394
0.572439415	0.500901563	0.020399766	0.000749842656988005
0.051213714	0.024188401	0.008347523	0.000758723515160631
0.292404130	0.216275853	0.024665460	0.000766253770651394
0.060046822	0.031879175	0.009043400	0.000773913816738574
0.541284309	0.356339816	0.059927884	0.000788973748059555
0.028182807	0.018961368	0.003518780	0.000803413463679023
0.054762688	0.037396845	0.005195609	0.000812319848539232
0.819925075	0.699773157	0.040766392	0.000829616694047564
0.554577224	0.448843256	0.031962610	0.000830965277828941
0.192810226	0.072824797	0.036062349	0.000842797775650268

0.090208330	0.038880840	0.018381986	0.000843684483359882
0.030969469	0.018613472	0.003760845	0.000855492456776674
0.077969289	0.047734359	0.009719063	0.000868625444884805
0.327356088	0.259872917	0.017508109	0.000925398490107632
0.075329860	0.049375222	0.008563647	0.000927316431401766
0.385651499	0.191998933	0.066198151	0.000969376281948015
0.137527237	0.084750443	0.017495869	0.00096939083576613
0.082116026	0.047010479	0.012182541	0.000974295860128855
0.098854518	0.068579869	0.010324650	0.000977327810837946
0.219500172	0.108923186	0.033859498	0.00098178806052818
0.124749604	0.044923200	0.023251957	0.000984448060730175
0.052710291	0.031044896	0.006537816	0.000987206792171805
0.145838631	0.041580329	0.030492366	0.00101306568348523
0.065160068	0.030820839	0.010161346	0.00102058993253071
0.485594689	0.339262512	0.041363090	0.00102096895367487
0.035735868	0.024744417	0.003945115	0.00104069175450583
0.085926673	0.065232390	0.006530475	0.00105420540447655
0.062314118	0.029534419	0.011628906	0.00105541874024962
0.063803524	0.035463911	0.008083424	0.00106323276640853
0.073105514	0.041749253	0.010696126	0.00107496677697312
0.070128204	0.029633636	0.012045607	0.00112445792703832
0.024846924	0.017487400	0.002687944	0.00113562487103266
0.059471579	0.032698634	0.009883422	0.00115312884844364
0.119499293	0.041385317	0.022446943	0.00115804258730953
0.049887259	0.031675014	0.006512415	0.00117656448797155
0.053355773	0.030693181	0.009070251	0.00118363713104641
0.089393488	0.032751890	0.017194011	0.00118451267548521
0.055282341	0.031151323	0.007218462	0.00118953750025623
0.059679615	0.040924323	0.006570492	0.0011924599436537
0.655835965	0.459199117	0.062581409	0.00119720097261188
0.397314671	0.292227331	0.035845999	0.0012337894833502
0.861689566	0.776289706	0.027689525	0.00126966211837448
0.070544107	0.033599421	0.010533702	0.00128769561369495
0.760093447	0.599825595	0.055283313	0.00128840711645545
0.260650104	0.180805706	0.028444852	0.00129297604085156
0.025340662	0.017175144	0.002931160	0.00133008812067979
0.335231852	0.165755420	0.053971114	0.00133841688587573
0.109556014	0.042981684	0.016976780	0.00134993660044481
0.067543268	0.034349164	0.012048127	0.00136567305577178
0.414830497	0.282766109	0.049355205	0.00141362596853781
0.543296042	0.338693723	0.062418206	0.00143358106389391
0.058664882	0.045601885	0.004853986	0.0014892106276354
0.154669188	0.114059798	0.012570117	0.00149903843208819
0.028265425	0.018478710	0.003132783	0.00150273713853608

0.089894904	0.027221383	0.015896927	0.00151086225826175
0.052174389	0.025047125	0.007834071	0.00151767355655841
0.026314032	0.017221274	0.003093156	0.00153182740537096
0.583125517	0.463779891	0.047055743	0.00154684878553392
0.097865089	0.045688443	0.015773627	0.00154989667077514
0.034830927	0.023053710	0.004355427	0.00156263448726686
0.061128727	0.026294228	0.011814902	0.00157587747225527
0.110951681	0.068438414	0.013361114	0.00158670511017001
0.872458866	0.829864494	0.016751331	0.001610819262709
0.095862731	0.066614680	0.011093482	0.00164169454319419
0.240926234	0.162859241	0.021058662	0.00169218417666731
0.069642836	0.031310531	0.010403171	0.00169294600199711
0.036874274	0.021969133	0.004820181	0.00169383939603919
0.058333410	0.025679396	0.008359387	0.00169642275990433
0.165395348	0.095513438	0.021120018	0.00170594259078683
0.050564366	0.023657191	0.007406784	0.00171827777076177
0.585226415	0.377766015	0.068238127	0.00172514614188101
0.022542046	0.016036788	0.002003249	0.00175922081220627
0.053108234	0.028377452	0.007541625	0.00177172025810803
0.094662207	0.054458471	0.013680090	0.0017725775108131
0.086708049	0.049960563	0.012213099	0.00180441082449474
0.065139584	0.022078567	0.012030175	0.00180968989963743
0.054431431	0.025577961	0.007902018	0.00182480656486792
0.068906904	0.044282837	0.007953787	0.00183981121596407
0.053625867	0.023234232	0.010693802	0.00184046533575476
0.160177331	0.090219275	0.023439490	0.00185619344740518
0.101633676	0.057822870	0.012809565	0.00187034186660992
0.594208660	0.421639970	0.054906863	0.00190814525564818
0.161441874	0.092095404	0.019374213	0.0019116459804831
0.039683778	0.025333436	0.004639013	0.0019131856681027
0.244881477	0.057622665	0.054945679	0.00191345500942755
0.032336084	0.017001499	0.004871655	0.00193383845838344
0.533229749	0.412597001	0.036586135	0.00193603149936682
0.038728777	0.022878643	0.005138124	0.00194096165539767
0.301572327	0.189894120	0.034571149	0.00195477958028191
0.046306502	0.026404120	0.006249213	0.00198007364991104
0.094320647	0.056168527	0.010156189	0.00201103006539155
0.042757706	0.024704612	0.005832756	0.00201487901596428
0.253491394	0.097577860	0.049403291	0.00201713836369913
0.086473605	0.056312188	0.008224916	0.00204797759358084
0.034844214	0.019862412	0.004182153	0.0020669515059148
0.083246076	0.044074797	0.011217739	0.00206810141768003
0.052564813	0.034595753	0.005387089	0.00211735119198062
0.698758849	0.600297999	0.032185910	0.00212503789003225

0.133789336	0.095709422	0.012461084	0.00212894002090623
0.082774004	0.047411241	0.010730444	0.00214991943971281
0.036146233	0.023774744	0.004419895	0.0021654151135924
0.069059242	0.029258349	0.013746801	0.0021774019676957
0.092737746	0.042214087	0.016218125	0.00218165881781463
0.083794293	0.055017319	0.009278062	0.00218336979597717
0.067696794	0.029678857	0.011477867	0.0021871068919103
0.140446542	0.054141494	0.025011875	0.00219890806716959
0.049634235	0.030042345	0.006179039	0.00220826133500541
0.070714579	0.043286088	0.007840829	0.00221334149575934
0.062628862	0.035099924	0.010161078	0.00221983324204917
0.101051581	0.036054051	0.019657757	0.00222010319458213
0.075327886	0.035862938	0.012763389	0.00223253058693142
0.039560945	0.028224193	0.003163054	0.00223309075280581
0.083692376	0.033058126	0.013662760	0.00224350264698155
0.035033597	0.020677017	0.004696555	0.00224723641856131
0.041297962	0.029161412	0.003503148	0.00224866469677349
0.072008240	0.039925376	0.010302607	0.00225410750682669
0.066555003	0.035380301	0.011466156	0.00226592406854671
0.049470007	0.024431873	0.007175270	0.0022680094773206
0.158308441	0.088960313	0.023140724	0.00227387660528469
0.791014615	0.596792052	0.054078662	0.00231552210910848
0.097072598	0.049766182	0.014766305	0.00231742577180607
0.290506614	0.194718203	0.031339958	0.0023343968261777
0.150239382	0.058923915	0.029111203	0.0023365613586663
0.047188071	0.029197702	0.005655553	0.00234896269743094
0.052134571	0.032703893	0.006546225	0.00236458494949065
0.323335854	0.219642403	0.030760576	0.00236978546467638
0.070275143	0.039137782	0.008930673	0.00237484363721179
0.066866638	0.038368190	0.009827354	0.0023795997325079
0.814845911	0.689841151	0.031768220	0.00238736051826148
0.062618541	0.027417725	0.009741207	0.00240325447415339
0.796733736	0.662918480	0.036248465	0.00240811418968543
0.064154719	0.034640512	0.008810026	0.00241032794101831
0.148547521	0.049609605	0.027843722	0.00241244516237452
0.072022444	0.038372756	0.009860103	0.00241265981562073
0.202462737	0.127568316	0.021019896	0.00243139308918182
0.880019248	0.775027930	0.034527793	0.00245004834628624
0.031813235	0.021258391	0.003823125	0.00246408516613807
0.038424629	0.019807169	0.004877558	0.00246845875986259
0.722453432	0.615204029	0.035197309	0.0024705952601324
0.104258781	0.048275865	0.017845791	0.00248379311972846
0.468465174	0.344052365	0.046436150	0.00250227548793421
0.061253113	0.033018485	0.009403655	0.00256267967317984

0.057099389	0.030187891	0.008955606	0.00257409596428539
0.529866520	0.385782531	0.055417428	0.0025796308843903
0.072869892	0.035878909	0.012811141	0.00259407133157125
0.172652518	0.075086286	0.028083075	0.00259697885491222
0.030469539	0.021194955	0.003216522	0.00262039504932314
0.087768924	0.047005438	0.012799343	0.0026287156385196
0.053816535	0.027320951	0.008148811	0.00263695891880557
0.075155976	0.022685059	0.014515591	0.00265030035494283
0.059674895	0.030111932	0.009168767	0.00266146255930512
0.044622288	0.022637720	0.007129332	0.00268813265324264
0.075603994	0.032772053	0.013027587	0.0026949288230359
0.904440386	0.874176520	0.011812853	0.00270230363483098
0.082777557	0.026327844	0.017425923	0.00273885222601302
0.056760114	0.033354155	0.006587670	0.00274924194078208
0.037801937	0.025609062	0.003827714	0.0027502568444486
0.274556613	0.207973956	0.023104615	0.00277122899886265
0.065041428	0.031499006	0.010117611	0.00278044034424552
0.090443028	0.048027090	0.012827689	0.00278538646727073
0.039593952	0.027061822	0.004387549	0.00279159676704469
0.071382693	0.036015100	0.010127194	0.00280572760224556
0.113152061	0.068725544	0.014742717	0.00281547610459957
0.089284041	0.060913085	0.010233332	0.00281821605678084
0.466181940	0.347269524	0.039881695	0.00284306099622626
0.110349313	0.061059129	0.012778032	0.00287191925559513
0.263518463	0.178161259	0.026533525	0.00288255035592751
0.043126982	0.026230040	0.005571858	0.00289587208366267
0.046258418	0.022413197	0.007228665	0.0029065750127275
0.145210024	0.082548608	0.016592077	0.00292408029622293
0.054502571	0.026634518	0.007632536	0.00294416615470123
0.054195422	0.030427623	0.007136933	0.00294562555501283
0.027760361	0.013299352	0.003973354	0.00294865903720879
0.052361257	0.033971892	0.006013867	0.0029513618519065
0.062239882	0.030849706	0.011143263	0.00296968121273305
0.038618213	0.024143750	0.005287589	0.00300552946493553
0.600198368	0.424144398	0.044474131	0.00302816354873669
0.053115556	0.031363630	0.007313837	0.0030328128205544
0.061030728	0.027826313	0.011522228	0.00304934961872634
0.045546464	0.023705208	0.007381302	0.00309556907307194
0.574218638	0.329175891	0.087825762	0.00310453744471941
0.094050444	0.024324639	0.020072476	0.00312969321262142
0.032979304	0.019945674	0.004262718	0.00313073373276736
0.222120293	0.134997218	0.031119622	0.00316387634229068
0.061175648	0.038209742	0.008606189	0.00316714302432326
0.116074190	0.049811294	0.021399677	0.00317616495823966

0.033335323	0.020000712	0.004669787	0.00318612157961868
0.033784297	0.021501998	0.004976329	0.00318954288837845
0.179062561	0.118755620	0.020796541	0.00319103447082353
0.041533754	0.030834559	0.003236965	0.00322057807206055
0.381018012	0.291904290	0.026823887	0.00323510866011218
0.134736041	0.099122553	0.012728709	0.00323833874955956
0.082561626	0.063954883	0.006948573	0.00325258610757589
0.198011800	0.100708950	0.037887089	0.00325492034246817
0.364607944	0.253081186	0.035345403	0.00325635311760933
0.140774316	0.052923579	0.026095565	0.00329084892590186
0.308074416	0.175105830	0.043388666	0.00330832710830921
0.086059912	0.019735773	0.020700251	0.00331894456315596
0.332778822	0.261174025	0.024314278	0.00333507975565938
0.051014157	0.029928444	0.006565744	0.00333730802638546
0.095934445	0.056236963	0.011256355	0.00340492430345237
0.738447095	0.613835379	0.041120111	0.00342640364098964
0.028245657	0.017580337	0.003205804	0.00343257381734118
0.052775410	0.034307552	0.007302082	0.0034595118595006
0.028910550	0.017457640	0.003984612	0.00348197718459
0.896168091	0.776471944	0.043058777	0.00349166293366654
0.103370907	0.066859472	0.013426385	0.00349513633883917
0.055985002	0.032798163	0.007763421	0.00349736304710893
0.065655610	0.038951994	0.008682690	0.00350194740911007
0.087848491	0.057014167	0.008558271	0.00350618543895467
0.509026049	0.337447999	0.049086840	0.00351230835712199
0.058185893	0.041932902	0.004786600	0.00351413083489978
0.086799735	0.043254122	0.013721558	0.00351777015543056
0.841032129	0.749323029	0.025290486	0.00352905097553154
0.909486727	0.831328303	0.025803421	0.00353105787157745
0.046799012	0.026264611	0.006212192	0.0035354836092213
0.069333724	0.029292696	0.012815685	0.00354713366497667
0.247577370	0.115147211	0.040905359	0.00356606388029113
0.520797601	0.396211168	0.040591885	0.00357473651488945
0.079070226	0.041580310	0.010843499	0.00357658346095944
0.830850458	0.681038687	0.057057085	0.00357954916282163
0.065308752	0.027189152	0.011625842	0.00358038828993157
0.103260313	0.062196225	0.012556461	0.00360058301432309
0.102123726	0.055336899	0.012942339	0.00362176512351882
0.773598321	0.661948267	0.038012533	0.0036234438823891
0.179221324	0.061207212	0.043580045	0.00363266046489384
0.073783845	0.039226228	0.011073067	0.00364937323810705
0.099076785	0.045321856	0.015584236	0.00366769729556217
0.049626832	0.026772875	0.006867594	0.00371532828635992
0.785829179	0.674628894	0.033452184	0.00374301991937087

0.205397498	0.085238352	0.035785740	0.00375055394818059
0.174837603	0.057714530	0.041873274	0.00376537170869193
0.206783720	0.121882188	0.024647331	0.00377076314342481
0.084853337	0.043092084	0.013146983	0.00379488632536217
0.580857279	0.340273220	0.084192120	0.00380934850334658
0.697897971	0.565300996	0.036771923	0.00382347517924885
0.158719192	0.051720039	0.032136293	0.00384482308752761
0.876879458	0.753224464	0.040603945	0.00388042624768213
0.072670875	0.035118253	0.012685626	0.00388296356655818
0.162668083	0.125747713	0.011908474	0.00389193522720608
0.049296087	0.025810067	0.007793650	0.00389980395064121
0.756179856	0.565034505	0.061113519	0.00390180490083364
0.070066738	0.034841035	0.011137602	0.00391125748012812
0.043265627	0.020807775	0.006520817	0.00391690208557112
0.026895706	0.016740295	0.002999640	0.0039263013050613
0.865180409	0.764498188	0.037627032	0.00392656087478859
0.017959966	0.012651809	0.001530969	0.00392889630145345
0.388668223	0.280991538	0.033586053	0.00394591472166495
0.841485020	0.755583564	0.032226000	0.00396731733406187
0.068832781	0.041187024	0.010237682	0.00396816777265341
0.368356059	0.221810692	0.050452019	0.00397241159245836
0.066654684	0.027944223	0.010683816	0.00398542821149386
0.026611995	0.015749748	0.004013957	0.00401954616173316
0.134004617	0.054767956	0.021294754	0.00402086889564885
0.073012472	0.043516750	0.010246454	0.00402910171665316
0.185628735	0.052299490	0.043524531	0.00403612029446587
0.841226019	0.370596606	0.211382411	0.00404585246722504
0.030775086	0.019248194	0.004507489	0.00404655225059499
0.372146894	0.154428310	0.072042939	0.00406307090349173
0.126802867	0.046181300	0.021152393	0.00407477368164635
0.114155662	0.079962078	0.011121290	0.00407490154469958
0.111162065	0.048788569	0.019341531	0.00407772267257447
0.047999510	0.026832933	0.005994501	0.00408826001033943
0.086646248	0.045968319	0.012724848	0.00413403180479398
0.071995445	0.046187891	0.008488859	0.00415035944327746
0.856828234	0.699106069	0.051001412	0.0041550186913791
0.061214683	0.036817796	0.009229419	0.00420622620652818
0.256634100	0.140326287	0.035654314	0.00422099204004627
0.113213691	0.038598896	0.022473824	0.00423070902559191
0.085748962	0.045426060	0.013453778	0.00424168505460603
0.058916133	0.019444039	0.010920144	0.00424938386331631
0.399893518	0.327499443	0.025890633	0.00424940857530124
0.046451663	0.027138862	0.005798533	0.00427232511402829
0.063796500	0.019432518	0.014125904	0.00428281049462324

0.048992216	0.023684534	0.007926449	0.00428443086992252
0.170344181	0.118714404	0.017931017	0.00429379363837408
0.627314990	0.500336854	0.045966013	0.00429556436140157
0.031679970	0.015437702	0.004509794	0.00430336887310071
0.610569281	0.444574248	0.053607360	0.00433369882194643
0.049575368	0.032141294	0.006456037	0.00433650301011608
0.128985428	0.053369972	0.024449991	0.00433743272126697
0.104003470	0.034888553	0.022078139	0.00436176006084984
0.532896771	0.404678640	0.044172688	0.00437074950432081
0.059852206	0.043405565	0.006545151	0.00438675377732306
0.087633509	0.057150474	0.010439217	0.00438728975970797
0.043340078	0.023582602	0.006546102	0.00439005511764653
0.430489181	0.298698807	0.045522356	0.00440847162959311
0.056877010	0.025896324	0.009737850	0.00441314402720725
0.190811415	0.046348798	0.039591806	0.00445838854454751
0.407834084	0.325622042	0.027114642	0.00446518505303296
0.035350489	0.020772299	0.004467290	0.00451681085367011
0.194076836	0.119881482	0.024650738	0.00455716949207176
0.035567483	0.018924732	0.005051029	0.00456092463860636
0.069801415	0.036950612	0.009293713	0.00458304421326124
0.092675007	0.053660943	0.012521709	0.00459152880408001
0.167627124	0.059500315	0.031100553	0.00460891083216263
0.059121886	0.029243976	0.010540631	0.00460917435799224
0.619630766	0.373063636	0.089063752	0.00462717425348471
0.084365271	0.046458959	0.012096306	0.00463381199326409
0.850213667	0.752392253	0.031255659	0.0046459925878622
0.119931427	0.048704438	0.024061022	0.00468365145594982
0.091019361	0.034907162	0.019042869	0.00468386764533187
0.440254190	0.358840458	0.029786865	0.00469728191952805
0.289644736	0.124081507	0.055823110	0.00470510980679738
0.068969688	0.045943789	0.007637441	0.00471159512923866
0.053287442	0.022636427	0.010130712	0.00471613907363878
0.075635716	0.046658535	0.008071997	0.0047212137837253
0.063100446	0.032995889	0.010153703	0.00472146677480461
0.074718978	0.046638416	0.009837299	0.00473987576252708
0.082983170	0.052954851	0.008828104	0.00475192757879273
0.065827529	0.036218646	0.008565542	0.00475388541911821
0.045303111	0.023297841	0.006877437	0.00477049418265
0.222545528	0.148452840	0.023909900	0.00478126053789934
0.049225400	0.024285726	0.008235310	0.00478143406239886
0.157671450	0.120578362	0.015807845	0.00479368071217885
0.044091131	0.029797882	0.005377475	0.00480944041788994
0.064708348	0.028475280	0.011771309	0.00486399776189585
0.035493348	0.017752445	0.005145137	0.00486504552906888

0.035719396	0.023542846	0.004964278	0.00487880158059995
0.082283730	0.032716088	0.014946811	0.00487927570547844
0.052501159	0.032069904	0.005870966	0.00491054363781752
0.074799836	0.031763643	0.013010442	0.00493227005158406
0.490881807	0.360288159	0.044572858	0.0049360791314476
0.052594688	0.028750200	0.006509399	0.00494216257464459
0.207198060	0.074070570	0.040199278	0.00498363564234946
0.122215391	0.043802950	0.030972228	0.00502572315636458
0.073672026	0.038159271	0.010470147	0.00502574355684366
0.816965566	0.722724215	0.027394094	0.00502770283089389
0.599408835	0.451455491	0.045785411	0.00503026769149639
0.046854457	0.024075207	0.006668374	0.00503080851913635
0.059831109	0.042425462	0.005999809	0.00506114617254402
0.426221807	0.271524214	0.051968936	0.00506238845985886
0.078537838	0.046640756	0.008471615	0.00508374498260781
0.270061747	0.177828975	0.030910995	0.00509641933960355
0.071403716	0.029487707	0.013164081	0.00509757419418474
0.057063232	0.024745278	0.009449645	0.00511375171174573
0.110837908	0.079399483	0.011084946	0.00512383899648954
0.042939043	0.020871258	0.006119501	0.00513591933519513
0.322450001	0.263403480	0.017996042	0.0051383069663515
0.083690934	0.058505540	0.008314906	0.005154263032324
0.057130839	0.036510074	0.007166801	0.00518686002111374
0.047714535	0.019068841	0.009494092	0.00519962197526463
0.139509352	0.081350038	0.017519451	0.00525157245542939
0.563007725	0.333491240	0.071718111	0.0052558445203256
0.079235438	0.026609294	0.013453449	0.0052717981671616
0.028855785	0.015909354	0.003880479	0.00529576482162504
0.887331466	0.788595240	0.034689503	0.00532885390521587
0.037514826	0.021614659	0.004826305	0.00533354277603353
0.142062608	0.034638611	0.031327516	0.00534993127455842
0.023729442	0.014678307	0.002784333	0.00535207927519788
0.125337017	0.045137757	0.025895318	0.00536564742610911
0.067690500	0.029202288	0.010633989	0.00536722327413683
0.059107083	0.032801237	0.008243656	0.00537199154748008
0.080607524	0.050394176	0.008696531	0.0054148351941628
0.359036390	0.278986778	0.023704820	0.00541711098048267
0.072257184	0.040905624	0.010909710	0.00541953437710872
0.067613116	0.036819264	0.009758214	0.00543903417079848
0.913724544	0.838729097	0.022910506	0.00544366712547056
0.483793116	0.330772180	0.039393987	0.00547442588627545
0.044814439	0.022500572	0.006607208	0.0054777554418694
0.052399321	0.023106587	0.008792096	0.00553963874965274
0.050906671	0.025645749	0.007626168	0.00556127372824254

0.025954311	0.017313251	0.002690768	0.00557974862519997
0.835607693	0.710820446	0.041453532	0.00562451111575936
0.059644522	0.029931425	0.008483354	0.0056347764749564
0.606188596	0.482528491	0.043374816	0.00563901644996064
0.531342820	0.381225323	0.046102023	0.0056446529240323
0.405264851	0.313131200	0.030080262	0.00564533533043482
0.108469715	0.038894486	0.024019771	0.00569452951221751
0.054830902	0.033910913	0.006390210	0.00570957568313116
0.456072706	0.255448122	0.063278300	0.00571612908183751
0.460023824	0.308214208	0.054958999	0.00573489269931867
0.914403600	0.794715384	0.035108579	0.0057412785369628
0.268827956	0.187493261	0.024996849	0.00575516427443028
0.566233579	0.379865309	0.053880034	0.00577269772686169
0.155141257	0.065802936	0.028763570	0.00577504957988314
0.495696807	0.352684526	0.041476187	0.00578505303186341
0.040901755	0.022699438	0.005358216	0.00579750586591848
0.043128522	0.023482623	0.006912450	0.0058753256073927
0.235766968	0.108569649	0.040345576	0.00589082474378987
0.053751853	0.033868072	0.006237034	0.00589103808608477
0.711078487	0.562183115	0.052301048	0.00589844998699919
0.116435759	0.035278006	0.024683755	0.00590395633689798
0.795650223	0.564195413	0.074154144	0.00590870346965047
0.057656779	0.029007249	0.008168142	0.00592521358072568
0.053155083	0.023803158	0.010372330	0.00592525111328579
0.091219819	0.060229129	0.008763299	0.00592899647755205
0.731892731	0.549681684	0.058727587	0.00593773437039676
0.166168494	0.090592570	0.021635125	0.00593881856746527
0.038052393	0.017478066	0.006937495	0.00595737006093477
0.320824105	0.254674191	0.018923392	0.00595786172023527
0.376550931	0.259234692	0.032144203	0.00595864729209861
0.062967740	0.038980140	0.007035270	0.00596635861097705
0.081067617	0.052875770	0.008176040	0.00598528894286953
0.036338116	0.021778681	0.004203970	0.00599935139036633
0.045021840	0.025035338	0.007480423	0.00601331394405867
0.059072476	0.044463069	0.005576553	0.00603681531605514
0.381303001	0.250439764	0.039852044	0.00611012216575426
0.101486721	0.039549561	0.018481333	0.00611303057960649
0.042178690	0.024002220	0.005261787	0.00613168083165697
0.846849029	0.778167979	0.020957017	0.00615827239519192
0.033921501	0.019964297	0.005498097	0.0061590562532082
0.042115757	0.026097067	0.006098410	0.00617078320235787
0.050916773	0.031697823	0.007989611	0.00617842763903714
0.052918665	0.035832139	0.006348942	0.00621722863917897
0.061996570	0.031562426	0.010453354	0.00627191469606662

0.852228515	0.757114476	0.033392509	0.00630718373644656
0.066179684	0.033434489	0.009558712	0.00634404464808058
0.081907991	0.030822692	0.015138796	0.00634891766266824
0.374054664	0.332313602	0.013918685	0.0063540645369776
0.874838330	0.817205920	0.016440463	0.00637108813104732
0.321792589	0.267030871	0.020156104	0.00638085917275261
0.084940634	0.034328562	0.014742470	0.00638649744209054
0.100358369	0.029237313	0.021491346	0.00638917045128378
0.231676781	0.063736016	0.047525804	0.00639560439979331
0.055538562	0.031282489	0.008752642	0.00640731803244366
0.489634537	0.401860674	0.026579854	0.00643328875837078
0.541193609	0.415676457	0.041143990	0.00643905504549668
0.209711714	0.102750158	0.036350896	0.00646367931059652
0.059038246	0.025112657	0.010224037	0.00646442712792225
0.043144708	0.023914789	0.005564673	0.00647619468491854
0.031792108	0.020606847	0.003767907	0.00650107448716736
0.034794540	0.020845986	0.004996777	0.00651827503322916
0.057388507	0.025491568	0.008822279	0.00652751094663376
0.036102878	0.026500372	0.003098126	0.00655319803831692
0.214657160	0.047068088	0.047383379	0.00658983464765955
0.162710431	0.056404882	0.030673589	0.0065952145598912
0.041298189	0.023504146	0.006036679	0.00660479605986581
0.055908251	0.024866532	0.008483214	0.00662310302431072
0.046169078	0.015658158	0.008281184	0.00665672129904411
0.132779302	0.053950978	0.027822324	0.00668205068300251
0.096381919	0.048316590	0.015339382	0.00669077877654853
0.671287398	0.034184886	0.241848596	0.00672939385887575
0.037414326	0.024825276	0.004245643	0.00675675973693046
0.857112706	0.728902514	0.039072103	0.00675972519338566
0.094025553	0.051179484	0.011238132	0.0067640229870752
0.076155285	0.032239427	0.014815091	0.00677194447324025
0.055663948	0.030086111	0.007593597	0.00682134612589047
0.176376502	0.087766769	0.029513286	0.00684979052658751
0.049351089	0.029634975	0.007022348	0.00689190796177299
0.036483299	0.027107987	0.003767965	0.00690694149487769
0.118222040	0.069628294	0.017146112	0.00691063713680937
0.385535483	0.273891714	0.032630858	0.00694798355414541
0.496208387	0.394727864	0.034159121	0.00697316685831498
0.407136184	0.200212551	0.060453953	0.00699541865067027
0.021286230	0.013947129	0.002300541	0.00706138761203326
0.391468180	0.243348658	0.044385718	0.00706578441865508
0.103425315	0.079366210	0.007341613	0.00708523437253316
0.374914894	0.201698110	0.052011596	0.00708987232979229
0.903202424	0.835340426	0.024833489	0.00711304886952598

0.722668449	0.630175481	0.031361718	0.0071934840622722
0.070256261	0.029318257	0.011675404	0.00719958471815058
0.045823657	0.024028823	0.008185958	0.00720394675286071
0.690759619	0.507014977	0.069280129	0.00720744674972207
0.043638438	0.025555582	0.005997834	0.0072169235998884
0.836009434	0.504157560	0.097086323	0.00726554600400863
0.258095211	0.105883006	0.059664059	0.0072798972270034
0.930379214	0.877257778	0.016545992	0.00728229148787424
0.636159630	0.446094773	0.049829509	0.00730248710294393
0.613175264	0.522404118	0.031327929	0.00730440546707238
0.391786382	0.204914811	0.050498507	0.00731162749209317
0.808531950	0.614333732	0.051632024	0.00731593373735447
0.047051796	0.025244717	0.007531635	0.00732094155290743
0.040497078	0.024428850	0.005489641	0.00732169858122512
0.932661925	0.885886351	0.015499425	0.00733335280626413
0.084299830	0.035609175	0.016294181	0.00739710184853364
0.054140667	0.031208619	0.007368883	0.00740167919634014
0.428167741	0.327872774	0.033837967	0.00740643932305006
0.051094552	0.030997844	0.006235197	0.00741669138049169
0.063395500	0.035616483	0.008641401	0.00744420734664348
0.362397875	0.296126182	0.024629606	0.00745891809228133
0.252828913	0.058409885	0.064592045	0.00746894291536695
0.134557330	0.045569205	0.024818825	0.00747320478774213
0.043958157	0.032548396	0.004222000	0.00750284930530078
0.020917330	0.016077016	0.001618261	0.00751686016463359
0.030272718	0.019721963	0.003654321	0.00753365117294037
0.053506557	0.028420174	0.008333323	0.0075372411510572
0.043865851	0.027358437	0.005624562	0.00757738243657659
0.926951495	0.874761860	0.016521832	0.00758595968341191
0.032566965	0.025714430	0.002359176	0.0075869790996839
0.926390700	0.900405350	0.008604162	0.00761641500860462
0.900752643	0.802149163	0.025731487	0.00762437081703307
0.365742395	0.242816241	0.039626456	0.00763372809427691
0.196491885	0.073098591	0.041441326	0.00764163144408528
0.286654500	0.214433085	0.023234147	0.00766418455044351
0.569647632	0.479489620	0.026996631	0.0076760477540698
0.082341081	0.047513717	0.012844042	0.00767879359319268
0.148414174	0.106749244	0.011860118	0.00768029251049111
0.918027367	0.828213201	0.026596449	0.00769025907220953
0.377320849	0.299127864	0.025711121	0.00769107513746288
0.029748016	0.019053987	0.003923863	0.00771682843199748
0.053981275	0.035881809	0.006340728	0.00773204037188243
0.027430753	0.017886335	0.003206590	0.00774008123521178
0.650641001	0.480253803	0.056396921	0.00776401194586484

0.026213993	0.019212444	0.002676799	0.00777803311686698
0.045340004	0.027363019	0.005818528	0.00778003108302333
0.625496477	0.501568586	0.040879782	0.00778711450128389
0.093246878	0.053829608	0.013735005	0.0078078531862844
0.034669280	0.021080507	0.003898748	0.00780839523559986
0.910662296	0.774810111	0.039316475	0.00781105510948452
0.223665808	0.146201504	0.022971596	0.00783774433176877
0.047110208	0.027177409	0.007113096	0.00783775794044591
0.041168262	0.024764161	0.004365831	0.00787564380951395
0.297231483	0.246604196	0.015134447	0.00791021254700059
0.034631708	0.024749890	0.003319699	0.00792828063430401
0.042926861	0.019640145	0.007870758	0.00793705798319936
0.230206088	0.097290177	0.044641202	0.00794616134068033
0.056624035	0.042916251	0.004492965	0.0079680224479981
0.045496084	0.018339189	0.009660665	0.00798789904680549
0.047326345	0.026761772	0.006514514	0.00799276486190803
0.043560257	0.026379849	0.006161613	0.00801634895670681
0.028845717	0.018799783	0.002905153	0.00804045344388725
0.478649251	0.281093492	0.072630446	0.00807169607650994
0.577669310	0.482850316	0.030093324	0.0081321476415879
0.061346912	0.039382152	0.008013413	0.00814026232286995
0.038902681	0.020461584	0.005593150	0.00814288037531251
0.884595188	0.760191087	0.040221870	0.00815589621260557
0.908755495	0.813456529	0.027928763	0.0081574986840875
0.033895534	0.023354873	0.004156899	0.00824215034764197
0.503944957	0.408572217	0.030656424	0.00825125337483995
0.332394903	0.225535206	0.038375715	0.00825368326407769
0.048799594	0.036200097	0.003693694	0.00825444832851302
0.051646600	0.027823730	0.008057171	0.00829789752833716
0.057608715	0.032309532	0.007526376	0.00830548496372762
0.555611158	0.382318516	0.049776681	0.0083236184517498
0.733722932	0.560180446	0.062618685	0.00834649531697581
0.064530106	0.036598650	0.008743966	0.00835722548695918
0.132643779	0.054325954	0.024635914	0.00837423640453083
0.199760885	0.067992432	0.038638484	0.0084009866235627
0.719947930	0.543417735	0.060933453	0.00840982985683819
0.177206913	0.054833996	0.037113254	0.00846102385292077
0.026409112	0.015699210	0.003667118	0.00847154622810933
0.618434879	0.500178450	0.043692024	0.00847394838441707
0.935603357	0.911409789	0.008542266	0.00849282062825132
0.547467050	0.327404036	0.060343743	0.00849343401414623
0.179485012	0.049870477	0.036137101	0.00853299204579539
0.039466458	0.025920104	0.003924808	0.00856192324298214
0.046136064	0.022369896	0.006215027	0.00856551198518446

0.146787807	0.071103970	0.023294117	0.00858755955467105
0.106528647	0.051217826	0.016593015	0.00859596403699643
0.037699040	0.021121766	0.005546776	0.00863173126980747
0.165141197	0.100636843	0.019285265	0.00864507312924574
0.904914052	0.719962907	0.068935751	0.00865643141317885
0.061731655	0.030763003	0.011074171	0.0086736811825521
0.044769807	0.027126134	0.005796041	0.00868712635456753
0.187035935	0.098666381	0.034390976	0.00869821925771047
0.036752680	0.022542688	0.003908084	0.00871045304652305
0.929491451	0.885747576	0.013941473	0.00874117854851126
0.043484625	0.018950810	0.008839641	0.00876503013753322
0.064822298	0.029109686	0.012561115	0.00877252461353002
0.034951575	0.019135038	0.004924728	0.00880554967321773
0.899025328	0.852659136	0.017124380	0.00880818434089963
0.806146839	0.627351411	0.057157152	0.00882757784994567
0.032264120	0.019201711	0.003988875	0.00882939521213272
0.180681687	0.079662564	0.029878395	0.00885771746653689
0.077223579	0.046210629	0.009188387	0.00886523276007248
0.888986424	0.693178963	0.068909756	0.00890698502829456
0.045802550	0.020470868	0.007861650	0.00895486028115682
0.225091789	0.123837172	0.030361379	0.00902401298564053
0.065596934	0.024229605	0.011684422	0.0090287496248237
0.063277447	0.031918719	0.011474267	0.009033421494817
0.598012918	0.446928989	0.042629813	0.00904296660370833
0.083196230	0.053736457	0.009089244	0.00904423551738708
0.412159133	0.275030686	0.037332238	0.00904623953168382
0.066960322	0.034831190	0.009888578	0.00904869056585919
0.063650547	0.032758791	0.009255691	0.0090591632410982
0.670796144	0.456164129	0.053374503	0.0090648862801481
0.030942443	0.017572723	0.004195233	0.00907037413124955
0.561023366	0.438317145	0.038924991	0.00907885878580859
0.636692061	0.546386702	0.035022552	0.00909191497026047
0.071734216	0.030884306	0.011063007	0.00911729940381979
0.062538355	0.032913148	0.010106993	0.0091182634891224
0.047983033	0.031288352	0.005712021	0.0091244371800793
0.033907424	0.018705647	0.004749634	0.00915313016833712
0.113575275	0.054038142	0.020030152	0.00916356584398572
0.073882944	0.037667911	0.011287541	0.00917201037537096
0.060639908	0.026187267	0.013409398	0.00917213598226311
0.074930355	0.031525528	0.013696980	0.00919764740282904
0.101061532	0.052761576	0.013150708	0.00920904848936451
0.133891185	0.041907902	0.028025198	0.00923453652022695
0.064487453	0.035977607	0.008501387	0.00923623441269899
0.304546205	0.239261595	0.023020834	0.00929440361217539

0.078141547	0.050384783	0.008237864	0.00936561000251583
0.506019467	0.261191988	0.075265217	0.00937060054392684
0.658139784	0.440138731	0.075430944	0.00937480125872731
0.559712364	0.444626108	0.040171198	0.00943792955161709
0.093374849	0.057108706	0.012465477	0.0094865528230115
0.082513797	0.046000156	0.010136868	0.00954817035041145
0.091030464	0.034576868	0.016868192	0.00957663576440626
0.596558351	0.469449026	0.040621902	0.00959778178362673
0.730756164	0.524426793	0.058349818	0.00963242931300076
0.978378664	0.925202686	0.020011992	0.00963706729829031
0.890228171	0.822047402	0.024252444	0.00965412804894876
0.055357214	0.027116599	0.010192454	0.00967131064308786
0.090846321	0.046094956	0.014410864	0.00967719366729945
0.041805828	0.026188573	0.004736157	0.00972360358276461
0.603651477	0.444647435	0.052880816	0.0097324672797686
0.370032462	0.161823393	0.068103480	0.00974901475546113
0.368747524	0.243613058	0.038779077	0.00975545979725061
0.370294086	0.298850664	0.022513927	0.00977710471354651
0.246534606	0.134798282	0.041118346	0.00985854372862602
0.077674482	0.050782114	0.009529604	0.00987409873079357
0.056478427	0.022201482	0.010335253	0.00987484367030709
0.036999068	0.022280084	0.005188001	0.0099300730060415
0.393578761	0.237982757	0.044918165	0.00993317287094041
0.346350264	0.206996573	0.042637125	0.0099636431943911
0.292320617	0.140605782	0.045950666	0.00999311999719116
0.468959383	0.373644590	0.032345716	0.0100341186230366
0.610706338	0.485744498	0.037740583	0.0100453230123969
0.281519511	0.166305598	0.033015543	0.0100605182937431
0.801831885	0.645174314	0.053006664	0.0100645958207867
0.056088944	0.035155587	0.005873060	0.0100719376669719
0.062624456	0.035416500	0.007723110	0.0100851908017756
0.055536947	0.022428887	0.010079012	0.0100906561708347
0.026361130	0.018310856	0.002846732	0.0100920115298989
0.042298637	0.031962452	0.003648844	0.0101135018397214
0.927319547	0.811845664	0.035991659	0.0101324698735556
0.051007570	0.038563135	0.003888622	0.0101359322110964
0.679866906	0.522136014	0.052830007	0.0101519882643568
0.086583327	0.030998217	0.014959242	0.0101826117009646
0.109313685	0.049787874	0.016743740	0.0101906452589251
0.028778442	0.019593163	0.003396246	0.0102031256950977
0.401303711	0.285516153	0.037574392	0.0102798276992168
0.385465366	0.241772953	0.045821223	0.0102926755732333
0.862904149	0.826768916	0.014187649	0.0103001578837347
0.055115560	0.025305107	0.009486669	0.0103139230146947

0.133530837	0.042289792	0.031546619	0.0103143221611778
0.082064331	0.038475762	0.015468619	0.0103816129307003
0.035725409	0.018825189	0.005132993	0.0104157449276407
0.797920769	0.568790530	0.068506750	0.0104195983228382
0.116116113	0.031327750	0.029980991	0.0104698281062308
0.058314051	0.039702673	0.006175174	0.0104701924667676
0.946402306	0.863341822	0.027274993	0.0104720847391008
0.081061298	0.041509864	0.011430162	0.0104876203727328
0.191243293	0.058623258	0.041919070	0.0105131824294302
0.294215843	0.170714763	0.037552870	0.0105558443364878
0.087033507	0.046515103	0.013691035	0.0105629677744195
0.598995852	0.501097788	0.037290109	0.0106188574090355
0.265696423	0.158679286	0.034556769	0.0106320188337165
0.123183325	0.038860961	0.025790590	0.0106517777286844
0.820975182	0.622110247	0.057796965	0.0106943891488435
0.090090146	0.049497690	0.011344155	0.0107058177701763
0.975853136	0.912849426	0.024293416	0.0107139967535994
0.555271337	0.335012355	0.080767095	0.0107224245010747
0.058967576	0.025602862	0.010672208	0.0107929123883957
0.083539825	0.054705238	0.009518770	0.0108061714219806
0.100350938	0.032701890	0.020353795	0.0108614539594914
0.047077423	0.026200219	0.006420175	0.0108713437938901
0.164422783	0.118714590	0.014767037	0.0108913107589098
0.136228579	0.087854101	0.014984875	0.0108967758545014
0.536724828	0.320520683	0.065545087	0.0109234436067838
0.063698302	0.036224897	0.009402417	0.0109590919913225
0.275386859	0.090795434	0.053538616	0.0109618556139898
0.849705349	0.705113002	0.040115137	0.0109635105802742
0.042740329	0.024191463	0.006172290	0.0109722568372489
0.222376426	0.158797610	0.019174687	0.0109782231812894
0.649451880	0.509668250	0.046827659	0.0109824816313896
0.255007874	0.152508543	0.032902766	0.0109911551476162
0.857802420	0.714992089	0.044927535	0.0110181135274549
0.748728839	0.519304642	0.071798355	0.01102195801289
0.622105968	0.485435785	0.045505230	0.0110318229501526
0.200682134	0.096855151	0.036003974	0.0110411963904553
0.057870909	0.026704602	0.008891678	0.0110457596267907
0.056926923	0.038562123	0.006423121	0.0110480712825528
0.890099782	0.827456541	0.020234891	0.0110524982496344
0.598040645	0.502177853	0.036192181	0.011058343475443
0.065315911	0.032347698	0.010435225	0.0110629277853895
0.594816263	0.526693265	0.026002868	0.0110672525512063
0.117111546	0.040132426	0.026887116	0.0110742379444401
0.435589459	0.027741037	0.153535756	0.0110815448823318

0.040674778	0.025919512	0.005669556	0.0110976284630234
0.398245209	0.317451519	0.031079616	0.0110986765864316
0.061004144	0.032667990	0.008741413	0.0111018463568288
0.649768322	0.531501563	0.041066915	0.0111242835199216
0.048575235	0.032939356	0.004727950	0.0111479166424721
0.379671802	0.314775737	0.024598486	0.0111619392652865
0.077668725	0.051062298	0.008767939	0.0111801191569837
0.885382277	0.775756501	0.032346252	0.0111854908169093
0.033666966	0.020231271	0.005268030	0.0111861816667145
0.736163314	0.475987424	0.077910171	0.0111961284337485
0.691776000	0.469112824	0.079024828	0.0112261742436354
0.029959423	0.019109808	0.003531077	0.0112295676436983
0.036064293	0.021434068	0.005627617	0.0112504581275262
0.044602666	0.024365604	0.006001963	0.0112625393559863
0.583491264	0.466816610	0.042520597	0.0112737305592917
0.044983295	0.021448376	0.007148945	0.0112810149953801
0.845318596	0.731447105	0.035796316	0.0112813043782678
0.577716519	0.443359962	0.049132919	0.0113157577431967
0.858229379	0.538552780	0.106929073	0.01131629789641
0.033621255	0.020844611	0.004376924	0.0113303814446836
0.032374361	0.017284024	0.004668951	0.0113710817403088
0.326830232	0.249075109	0.023500180	0.0113768442269883
0.515902839	0.398053020	0.046826343	0.0113876670373999
0.056941238	0.027212603	0.009374123	0.0113980842015069
0.294344408	0.162976381	0.037539080	0.0114443302639066
0.870986405	0.781980236	0.028109225	0.01145240494447
0.115026613	0.030291706	0.025127476	0.011462609205687
0.195126659	0.115687617	0.027757486	0.0114755425279654
0.342811303	0.202020004	0.040577479	0.011535667674465
0.032383537	0.018566733	0.005086020	0.0115373840562061
0.020307318	0.014776713	0.001888718	0.0115410872096678
0.080498725	0.038310144	0.012799903	0.0115577731976707
0.038436077	0.023363622	0.004876023	0.0116172625848296
0.037683070	0.022398968	0.004979924	0.0116189762745299
0.078149611	0.046110657	0.009540921	0.0116330132291625
0.045483460	0.028313138	0.005311312	0.0116737035799851
0.881769147	0.799895628	0.022917369	0.0116900338326406
0.034052692	0.013722080	0.005483142	0.0116905689880478
0.839032284	0.729434212	0.041088432	0.0116967364997204
0.065881363	0.031911774	0.010172383	0.0117001233660369
0.036570159	0.026180519	0.003969483	0.0117220708891978
0.066398067	0.029089821	0.010508657	0.0117329337107057
0.033671078	0.024625791	0.002847848	0.0117649448000604
0.052537731	0.030060366	0.006346275	0.0118084819124397

0.817140678	0.631790620	0.069360724	0.0118127444651489
0.642246917	0.503099234	0.042394288	0.0118192036300283
0.605297514	0.435352892	0.065950913	0.0118218846013322
0.030975450	0.017446755	0.004188510	0.0118247802159619
0.630621432	0.511064203	0.035523176	0.0118291403838693
0.077528160	0.048725241	0.009139939	0.0118480635898772
0.785909360	0.649284432	0.040028058	0.0118916010931889
0.101201096	0.054086224	0.014623122	0.0119004946900997
0.681710851	0.578395830	0.030233617	0.011926346027672
0.861109561	0.763456277	0.030849106	0.0119353141737797
0.104130290	0.070485341	0.010058986	0.0119844737871682
0.506296382	0.386099844	0.040401794	0.0119917886875261
0.026120806	0.014624552	0.003524761	0.0119976253072952
0.269810602	0.087326794	0.051552954	0.012022588313815
0.053770113	0.029688891	0.007313235	0.0120623473537956
0.766890057	0.575915990	0.066132940	0.0120845299570347
0.287914902	0.071971699	0.070381662	0.0121255337953534
0.054958379	0.029401335	0.009370424	0.0121358492342352
0.049945127	0.026129761	0.006050774	0.0121481103768215
0.792816536	0.569963510	0.066278522	0.0121731725270265
0.089909891	0.060819360	0.009299304	0.0121851435025592
0.064274303	0.045519492	0.006808525	0.0121981963217834
0.727169883	0.566369540	0.055302660	0.0122039560971396
0.904137992	0.451322091	0.161325535	0.012213165757263
0.901006645	0.855055577	0.014277057	0.0122215637113872
0.133806886	0.052758737	0.025203771	0.0122391814851779
0.776394523	0.600919338	0.047085705	0.0122811641321825
0.057501647	0.035130997	0.008139937	0.012288429230616
0.098608450	0.034842537	0.019297416	0.0122958000069324
0.126843354	0.035908399	0.028568300	0.0123006183793962
0.052198373	0.027745664	0.007117579	0.0123259150017849
0.041729269	0.022080832	0.006035803	0.0123689955866849
0.073268427	0.034541929	0.013294877	0.0123846624741132
0.105656853	0.043295304	0.020997611	0.0124175804206354
0.028239765	0.014731998	0.004069899	0.0124285380882129
0.591892830	0.411532703	0.054596799	0.0124358456389534
0.559706778	0.042376195	0.189000528	0.0124419397617333
0.077438040	0.038906914	0.011836354	0.0124517454511608
0.109002402	0.033575869	0.021525256	0.0124830790848686
0.266782673	0.185710069	0.026066829	0.0125096622325411
0.102723074	0.066003892	0.012703515	0.0125108566650284
0.277162811	0.122145009	0.053941018	0.0125367791540097
0.812700985	0.696253472	0.042756718	0.0125644200797123
0.318628675	0.164849598	0.053303216	0.0125680906880675

0.837484951	0.734341687	0.036201393	0.012575044389519
0.043147073	0.023940382	0.005896343	0.0125922032924224
0.577633158	0.472063659	0.040649782	0.0125925307720392
0.638390395	0.516642934	0.048407598	0.012593142883343
0.095970884	0.049827085	0.013733880	0.0126113602222935
0.042949774	0.023038740	0.007076135	0.0126527513198183
0.861163696	0.753436775	0.039721869	0.0126618656166578
0.077648800	0.037120938	0.012176939	0.0126751717707927
0.642725546	0.547157259	0.038152472	0.012689704542032
0.041698167	0.018926970	0.007280001	0.0127904497058827
0.147678613	0.075136162	0.025839050	0.0127950458741316
0.616905528	0.496740141	0.035857111	0.012801227520402
0.431953269	0.321057643	0.031172784	0.0128165349943791
0.292449219	0.187899066	0.028080144	0.0128536002552634
0.142441995	0.039823870	0.033735870	0.0128609894599651
0.714257707	0.599084200	0.034083642	0.0129153927782218
0.212232624	0.126391032	0.027527339	0.0129331799635819
0.612615227	0.491507855	0.044146636	0.0129403539742148
0.473596092	0.384916551	0.025432508	0.0129690169821104
0.044103649	0.023664510	0.007008211	0.0129715473196529
0.718632489	0.613947546	0.036076732	0.01297158267867
0.186636307	0.106591918	0.023081728	0.0129794605969626
0.126834879	0.060103079	0.019294731	0.0129887171475041
0.058399728	0.018994982	0.011375572	0.0129904819008938
0.945373562	0.870102922	0.021281648	0.0129946189369663
0.065092650	0.033810047	0.009738420	0.013013814143915
0.039957622	0.024795780	0.005717342	0.0130165613741333
0.936732423	0.912543332	0.007889994	0.0130317918415069
0.887257215	0.804850381	0.025407798	0.0130671330427135
0.934281093	0.866295460	0.025653175	0.0130679993754135
0.870392051	0.796110931	0.025106922	0.0130740462764813
0.068822183	0.044858810	0.007576892	0.013083649265912
0.039865528	0.026160980	0.004348366	0.0131480052787449
0.615266929	0.485257846	0.039460372	0.0131780525092279
0.655377571	0.481952514	0.050292086	0.0132316581640422
0.058770546	0.037345171	0.007411179	0.0132529899770915
0.497722588	0.396884965	0.032316740	0.0133098622018432
0.047338818	0.024634092	0.007018915	0.0133776057087192
0.118432559	0.062614654	0.014974504	0.0133948298693172
0.583097739	0.419748822	0.053728876	0.0134049081698191
0.043906108	0.025459340	0.006250519	0.0134607374933148
0.083015955	0.049672191	0.009119658	0.0134817123878879
0.381703525	0.287711499	0.038770965	0.0135413883337619
0.934833092	0.875493622	0.021437050	0.013546398826381

0.090412868	0.039206387	0.017211228	0.0135467435930743
0.378852457	0.259231842	0.049702128	0.0136068045761788
0.042662666	0.029991473	0.004141572	0.0136107084748401
0.555135337	0.434518006	0.041585539	0.0136274937361757
0.059899284	0.030830570	0.008878768	0.0136477975273466
0.385283249	0.317962426	0.022253149	0.0136620597137666
0.486839193	0.273915563	0.063562624	0.0136820691785222
0.053061540	0.024974710	0.008224137	0.0137370111148541
0.162775500	0.108336120	0.018970640	0.0137828751608167
0.069358664	0.027548457	0.012655631	0.0137946484955701
0.041420970	0.029128581	0.004444139	0.0138111401224058
0.064142315	0.033396875	0.008961891	0.0138746677812961
0.188298041	0.075647489	0.034119864	0.0138937724333454
0.897152841	0.801873803	0.027923202	0.0139178338542446
0.077336584	0.040563625	0.012616404	0.013929304979506
0.049666196	0.030087304	0.007021629	0.0139872061286985
0.967449183	0.928093132	0.010946051	0.0139971125888957
0.075951599	0.050438723	0.007914039	0.0140260082810358
0.061416305	0.029994669	0.010752087	0.0140786394687324
0.951883705	0.822935962	0.042387641	0.0140860471374726
0.052473874	0.026850521	0.007527653	0.0140956978195886
0.064930810	0.046431223	0.005974194	0.014096945907987
0.932643931	0.802856082	0.038277977	0.0141127738297068
0.058523160	0.031569555	0.009107757	0.014125094581279
0.704649753	0.587521148	0.037128537	0.0141428300352917
0.503785438	0.425460821	0.026083804	0.0141483167333825
0.048293168	0.022657233	0.008016417	0.0141593934465501
0.033549773	0.020613369	0.004220724	0.0141809291865593
0.556501265	0.417610846	0.048458067	0.0141934938466915
0.129039621	0.050254210	0.025338176	0.0142307678972818
0.094419939	0.049914016	0.013365367	0.0142615153314823
0.078157335	0.043882136	0.010667207	0.0142770219148602
0.088523701	0.039899801	0.013751414	0.0142774453137203
0.560882832	0.424245331	0.045479144	0.0143603143841563
0.033891294	0.024098296	0.003697278	0.0143675609558583
0.031985098	0.020123516	0.004207260	0.0144006249117848
0.889935875	0.797944040	0.037605659	0.0144042181616274
0.408130283	0.033438120	0.115473114	0.0144401260080885
0.038749421	0.019817599	0.005215426	0.0144416842966274
0.172130090	0.057610889	0.034490976	0.0144533541896119
0.092462668	0.051630040	0.013887342	0.0144632544672928
0.309314356	0.138777059	0.048403125	0.0145029199217631
0.647741018	0.325518101	0.107089467	0.0145251705474976
0.891714293	0.792328050	0.027837398	0.0145319620034342

0.037806276	0.018525596	0.006244633	0.0145333201871787
0.046724712	0.022415347	0.006944517	0.0145388261210704
0.654627699	0.534890708	0.040770322	0.0145485591115111
0.819379496	0.735988725	0.026187339	0.0145496982145299
0.873769548	0.771279099	0.035968127	0.0146465895426525
0.028764341	0.015707178	0.003932001	0.0146539241441419
0.878173451	0.390581141	0.168926440	0.0146647322318738
0.094323860	0.036078569	0.019362040	0.0147155442919125
0.116389848	0.047634111	0.023089210	0.0147371120577864
0.877383073	0.768166626	0.034182934	0.0147643759601096
0.171963438	0.129375207	0.014715920	0.0148047113137349
0.633119701	0.517072090	0.034427547	0.0148686008621637
0.062692046	0.032116898	0.009055578	0.0148700021584226
0.604449376	0.476435352	0.036297830	0.0148975607658835
0.845739708	0.647526678	0.051712302	0.0149123627946811
0.538298955	0.405248734	0.042967149	0.014944118337814
0.881367711	0.820769960	0.018036539	0.0149750142192571
0.078045943	0.044379610	0.010811886	0.0149830621992241
0.903019473	0.828670784	0.023649883	0.0150770642182023
0.414081516	0.251512094	0.045495941	0.0150776083269005
0.152123412	0.036830390	0.037992915	0.0150787586370775
0.189013828	0.101242590	0.025261693	0.0151115520588994
0.044011649	0.025133784	0.005805767	0.0151154804950426
0.028067493	0.020248283	0.002220322	0.0151357348345225
0.927669334	0.875374443	0.019067984	0.0151615918744014
0.069954292	0.039498234	0.009410748	0.0151855456428489
0.913430842	0.847005946	0.022276823	0.0152244677856536
0.087417557	0.061636073	0.008450224	0.0152280831927608
0.034979122	0.020225133	0.004748556	0.0152403589966886
0.059047991	0.025244624	0.011091267	0.0152591285081583
0.615249904	0.358376911	0.089122061	0.01526538406443
0.151540236	0.048442930	0.036261534	0.0152753480314431
0.901759399	0.859538631	0.016145895	0.015289671696643
0.032721204	0.023170242	0.003074015	0.015313100603716
0.034201399	0.024173284	0.003314333	0.0153788930459028
0.900665017	0.820135415	0.026613864	0.0153802932541655
0.091278046	0.061453297	0.009775071	0.015396048384191
0.766698182	0.581850537	0.055337197	0.0154496254349854
0.276522085	0.064349835	0.061986915	0.0154985321971333
0.438434827	0.333953410	0.034845524	0.0155105889220407
0.597513742	0.398396101	0.065759751	0.0155148080023595
0.063268579	0.041326132	0.007034607	0.0155309159349235
0.933746801	0.897552235	0.010708985	0.0156131079328072
0.854558920	0.706014595	0.048499698	0.0156255371857397

0.944278291	0.863101202	0.026653671	0.0156474031520071
0.501606627	0.392642043	0.036149576	0.015657325833747
0.039661948	0.020860623	0.004962572	0.0156582576386549
0.029991597	0.019691851	0.003174405	0.0156598545748711
0.393067345	0.201463297	0.059840332	0.0156719075842699
0.775350489	0.607231881	0.047772920	0.015733855046715
0.462796836	0.356856432	0.032226481	0.015741813558636
0.463139307	0.284185498	0.057162805	0.0157577805864692
0.911766548	0.772196347	0.049362583	0.0157846529653851
0.037572986	0.023095116	0.005324156	0.0158141887377854
0.678884185	0.513208564	0.051486477	0.0158205991167677
0.250540257	0.167942723	0.027740303	0.0158691666070084
0.090601771	0.042799938	0.015826370	0.0158739107339358
0.027236051	0.019127914	0.002847221	0.0158784819923256
0.037868689	0.019651640	0.005392855	0.0158822351977671
0.920169884	0.868202318	0.018726197	0.0158889151023383
0.067360219	0.048071729	0.005316585	0.0158977727000711
0.788706623	0.718191866	0.021276621	0.0159341413593638
0.224682040	0.068226166	0.048069439	0.0159353809116365
0.883882644	0.808970510	0.025422141	0.015954770350122
0.298109905	0.186788251	0.032031975	0.0159569827992992
0.111718307	0.053902666	0.016201353	0.0159669136380321
0.653510113	0.511969511	0.049415394	0.0159677075134756
0.901751456	0.844862112	0.019279537	0.0160795044293739
0.179058642	0.066825113	0.034011418	0.0160953671710708
0.059313026	0.024955751	0.012112168	0.0161353268475498
0.085279497	0.047601227	0.011576569	0.0161617550487077
0.029456481	0.017958309	0.003799650	0.0161676272184153
0.273404613	0.204635136	0.023238667	0.0162403392949001
0.712242847	0.589654174	0.036422205	0.0162640623045844
0.616998484	0.430443665	0.061342050	0.0162644145775189
0.076634536	0.047570472	0.008591337	0.0163150985182054
0.852826070	0.762073564	0.031283027	0.0163155975915668
0.045393853	0.021699917	0.007106022	0.0163185797994461
0.863328524	0.753010382	0.029871364	0.0163245534081944
0.100929450	0.046821445	0.017970654	0.0164275302277267
0.066988077	0.038633512	0.008310208	0.0164538830600373
0.162819909	0.070049365	0.028515862	0.0164575567519985
0.070703937	0.045839126	0.007190885	0.0164931542437109
0.200141037	0.099929716	0.036383373	0.0165280683675859
0.602005764	0.499364075	0.035496387	0.0165368345404129
0.165527819	0.044326375	0.035048317	0.0165401558062758
0.194263119	0.116967427	0.021752159	0.0165579903632684
0.053005898	0.023193652	0.009695283	0.0165723639613846

0.902610023	0.775833520	0.042271325	0.0165806025069886
0.924956879	0.859865506	0.019468425	0.0166079966123164
0.123372016	0.051780185	0.020824350	0.0166329254498305
0.060105581	0.018551300	0.012133967	0.0166669044582211
0.049417832	0.032700569	0.005644437	0.0167119641393648
0.045336227	0.030357411	0.004923265	0.0167269730026977
0.623980033	0.400492546	0.072327348	0.0167592283852675
0.045766542	0.033323471	0.003846263	0.016804508932337
0.057478067	0.029462820	0.008447600	0.0168112423586213
0.048219029	0.026990430	0.007651036	0.0168315399322573
0.065218619	0.027371602	0.011614870	0.0168428200974618
0.193446796	0.110768510	0.030807216	0.0168492091225589
0.890792877	0.756335532	0.045667996	0.0169010715042102
0.022266288	0.009894054	0.003428320	0.0169792878699405
0.433633823	0.297298357	0.047328525	0.0169916732932521
0.091627421	0.055529904	0.012933361	0.0170327448111731
0.355542730	0.250474666	0.033131412	0.0170328851592054
0.141479419	0.056812354	0.025362025	0.0170378810340645
0.045424338	0.025109584	0.007124036	0.0170625553876888
0.074619422	0.035498900	0.012937779	0.0170769663553516
0.043975429	0.028432825	0.005463920	0.0170785109713521
0.234351474	0.111386875	0.049524715	0.0171138321230984
0.921450768	0.877531175	0.013659587	0.0171457722451265
0.155508462	0.043418459	0.035708293	0.0172035249881696
0.051326539	0.024763804	0.007474357	0.0172041602284123
0.128135342	0.066270012	0.017687843	0.0172132019468246
0.070281371	0.032510414	0.014962235	0.0172431305627162
0.671128430	0.460597518	0.076259022	0.0172435161079628
0.115964292	0.033015463	0.026039387	0.0172558726279873
0.064121430	0.037372726	0.007969363	0.0172629694408135
0.086317392	0.045120226	0.011525197	0.0172662998138155
0.051778554	0.020822705	0.009125345	0.0172755176349298
0.469228831	0.301525596	0.057353254	0.017287531430011
0.946887526	0.909239266	0.012954818	0.017290717193531
0.533285384	0.335077280	0.069031556	0.017316106648626
0.319452777	0.184578273	0.043264767	0.0173209257372065
0.679344499	0.411453152	0.089641772	0.0173453303436508
0.230920755	0.153409751	0.025287612	0.0173675084974973
0.906260477	0.844755804	0.017190489	0.0173773371748529
0.169255658	0.038857315	0.037724152	0.0173786553550802
0.112807820	0.083044919	0.010417998	0.0173849017473668
0.689537106	0.620489242	0.025081169	0.0174078270602434
0.215116715	0.043559566	0.056374136	0.0174536550048945
0.030217526	0.020627079	0.003364570	0.0174566631128767

0.593191568	0.526549812	0.020593103	0.0174634061163213
0.042674706	0.020397729	0.007406155	0.0174760306463017
0.044296835	0.023353646	0.007921370	0.01749664199873
0.055063892	0.023381611	0.008905151	0.0175129544394268
0.098317468	0.064395005	0.010752201	0.017529188515731
0.072457760	0.033512662	0.013470565	0.0175534476111795
0.025416938	0.014987574	0.003193015	0.0175543875552053
0.790136515	0.705675072	0.028265607	0.0175826749699646
0.055634256	0.027673807	0.009030871	0.0176014848118168
0.034032288	0.021423669	0.004526548	0.0176325055299367
0.295634214	0.159715571	0.051576668	0.0176330557600655
0.942137205	0.833737937	0.035592541	0.0176397561594563
0.189596167	0.039858148	0.044301960	0.0176518087438882
0.051298990	0.028284007	0.007268022	0.0177041570264211
0.889165871	0.759587815	0.045970215	0.0177897396386503
0.682406096	0.605083832	0.029530189	0.0177978013556044
0.065926104	0.035115278	0.010587620	0.0178086041617898
0.046778789	0.024238341	0.007105389	0.0178093172482624
0.077347632	0.040329123	0.011954917	0.0178862604417203
0.590542744	0.472739813	0.044567509	0.0179058421552474
0.274147616	0.104250349	0.059804483	0.017920714779506
0.236360868	0.066959318	0.056549529	0.0179410266949
0.107926760	0.030264131	0.025367350	0.0180002003330232
0.852393867	0.617824690	0.073684404	0.018010622169506
0.141617788	0.040055989	0.032336391	0.0180260734862198
0.051084811	0.032146599	0.006931684	0.0180508093997591
0.934180544	0.880552942	0.017342934	0.0180543223015553
0.047993285	0.026451089	0.006978265	0.0180902797681355
0.035885078	0.024768970	0.003679362	0.0181250396230996
0.079839765	0.032597432	0.015276357	0.0181306012013818
0.024623610	0.012170567	0.003569975	0.0181386904213569
0.961164780	0.934530519	0.007871753	0.0181901018335691
0.845351649	0.609045528	0.077849484	0.0182056692709797
0.072904727	0.029876932	0.013631239	0.0182357722873271
0.095478845	0.048481167	0.014502425	0.0182866536359791
0.038412278	0.019382169	0.006406905	0.0182903208138258
0.067865191	0.037504421	0.008872301	0.0183117296038247
0.287481453	0.166932822	0.038352184	0.0184018088717534
0.034374722	0.019308813	0.004853321	0.0184028160527863
0.024309165	0.013638686	0.002750397	0.0184046003618536
0.663136540	0.488375368	0.062343224	0.018405675611449
0.079837378	0.035895186	0.013601694	0.0184183431740862
0.219185374	0.149942239	0.021853442	0.0184294677605037
0.048036164	0.028014984	0.006062551	0.0184330340138221

0.056748354	0.026373795	0.008883673	0.0184830471081177
0.365873725	0.208023412	0.054339409	0.0184978103201081
0.257936528	0.136481439	0.032824038	0.0185134803884548
0.167082052	0.068793013	0.031345806	0.0185267553815361
0.156156759	0.036577863	0.036600123	0.0185464618411662
0.108308978	0.065262230	0.013583018	0.0185546852402677
0.026748303	0.014640122	0.003458441	0.0186052563394888
0.334190720	0.026671713	0.095532575	0.0186079551082607
0.618125751	0.379625535	0.074323512	0.0186254121378312
0.939755775	0.893084185	0.014251084	0.0186465133167657
0.065706424	0.036930975	0.008010376	0.0186497189910696
0.219360308	0.050394915	0.062538242	0.0186506622429712
0.865770649	0.726573949	0.045644853	0.0186628031382139
0.523632584	0.377209970	0.047802357	0.0186658842428484
0.520802554	0.442826385	0.029089598	0.0187088372416391
0.904431827	0.838552586	0.019716868	0.0187130261224002
0.041069724	0.019225630	0.007009369	0.0187474143578305
0.256730255	0.127421861	0.039084467	0.0187616755835191
0.064816039	0.037647477	0.007646274	0.0187946783400624
0.334280909	0.225365950	0.035155402	0.0188277637112496
0.030645523	0.022482480	0.002739167	0.0188337408451209
0.737709683	0.628895563	0.034812126	0.0188751531220698
0.067150103	0.036067905	0.008698572	0.018945581580445
0.162081230	0.043300258	0.036055258	0.0189631987847183
0.090163981	0.034814567	0.018850621	0.0189808327611648
0.294794923	0.178413007	0.038028859	0.0190319351380265
0.061223699	0.033492273	0.008465187	0.019036445960507
0.057601940	0.023953977	0.009694506	0.01904043934841
0.068400326	0.035764367	0.009981954	0.0190841263551371
0.959858908	0.735151027	0.065613878	0.0191130334536164
0.045919338	0.020299774	0.007077525	0.0191159372888811
0.077214033	0.042728148	0.010424114	0.0191176439732258
0.310944185	0.215113175	0.030064693	0.0191355944574729
0.217222039	0.105697503	0.043815163	0.0191406469764807
0.055630371	0.026628856	0.011055965	0.0191510402439741
0.044809181	0.023310137	0.006421746	0.0191543171250895
0.225603016	0.119114452	0.038910348	0.019178034953461
0.327382443	0.217255257	0.031449713	0.0191970654702102
0.043621845	0.019935517	0.007517097	0.0192248526731047
0.933937031	0.830640392	0.031982504	0.0192304573084408
0.602916269	0.417885020	0.054543639	0.0192503003913688
0.929357396	0.763372459	0.050456218	0.0192514051671632
0.029574340	0.015832783	0.004993791	0.0192629785641115
0.368353629	0.212484255	0.048241555	0.0192727748979549

0.028716705	0.014875867	0.004445601	0.01930018001945
0.034676876	0.016459626	0.005555883	0.0193212825534146
0.127547119	0.049696734	0.022864412	0.0193598218629756
0.050198312	0.026844159	0.007140354	0.0193670243296601
0.088554974	0.052004068	0.009715031	0.0193880507377385
0.032426385	0.019134358	0.003562811	0.0194018839528257
0.910258369	0.800433307	0.042241530	0.0194087112462135
0.068858981	0.039833572	0.008341209	0.0194118880425691
0.028623747	0.018089603	0.003720807	0.0194287664966954
0.662212556	0.598618818	0.020807384	0.0194535080900368
0.568749990	0.382027391	0.055875779	0.0194641921132821
0.851933339	0.727292734	0.041583999	0.0194689582091724
0.652589173	0.555208750	0.035563384	0.0195928559009242
0.552008034	0.417519696	0.044227923	0.0196329097707488
0.048443525	0.023056679	0.007370186	0.0196687727221828
0.031862754	0.017502934	0.003756798	0.0196793710596799
0.919907103	0.811946421	0.031105898	0.0197361141044458
0.031491253	0.024621140	0.002031724	0.0197376047584795
0.037289171	0.021549542	0.005277945	0.0197415615664726
0.038549070	0.024513568	0.004863132	0.0197510957213361
0.092275726	0.061318294	0.009608997	0.0197764945010874
0.828594734	0.632065154	0.056730602	0.019793634160922
0.045792992	0.026698351	0.006230980	0.019816752539884
0.081923540	0.028748425	0.018875702	0.0198252893933087
0.055371997	0.025704116	0.008217276	0.0198349512828378
0.572515032	0.400321270	0.057863825	0.0198483117307047
0.094764274	0.069179227	0.008217328	0.0198643503321247
0.929840327	0.828686096	0.030483749	0.019930102299894
0.140594893	0.038614035	0.029754198	0.019979287244229
0.916928897	0.835465192	0.027874019	0.019980204848161
0.084124197	0.051452941	0.010195929	0.0199856452539193
0.046784564	0.025951142	0.007827857	0.0200066202989552
0.038165381	0.021636511	0.004859743	0.0200395836514961
0.235100439	0.106890042	0.039934257	0.0200419957008359
0.481065417	0.415042248	0.023838117	0.0200886922868783
0.081509221	0.036422942	0.013261132	0.0201636056614196
0.280567405	0.162944744	0.037925118	0.0201647730428493
0.398375366	0.212655778	0.067436000	0.0201988524335274
0.052835054	0.028052204	0.006673114	0.0202070954917292
0.436505960	0.342853892	0.028753006	0.0202167180770222
0.197909454	0.111843089	0.025998020	0.0202555704192492
0.080159800	0.041777126	0.010725740	0.0202598500578568
0.807315893	0.692498584	0.045053084	0.0202908855337593
0.910274929	0.866303104	0.016208953	0.0203461432325641

0.576841223	0.234283163	0.107072676	0.0203466988238976
0.572052508	0.443888944	0.040469703	0.020347649835562
0.069023224	0.040292655	0.009886486	0.0203539520085513
0.140312114	0.062849099	0.022375387	0.0203636774975198
0.285242240	0.206949345	0.029552333	0.02037239971529
0.972798719	0.950743281	0.006949552	0.0204267967220429
0.907544449	0.710473900	0.066803051	0.0204300875155645
0.734679387	0.566336096	0.056838335	0.0204372213653452
0.145226054	0.050582676	0.030512910	0.0205127452643226
0.086936119	0.048315355	0.011452719	0.0205131703414813
0.167661753	0.107992368	0.019710858	0.0205343619910105
0.047164340	0.026284219	0.007860750	0.0205438995775583
0.081149441	0.035780029	0.014593501	0.0205540963056721
0.173464591	0.116592276	0.018503535	0.0205781200419207
0.049421558	0.024466447	0.007681728	0.0205898406973485
0.102369311	0.044892619	0.016913290	0.0205963139308257
0.038973790	0.019495587	0.006848888	0.0206107662547134
0.152959751	0.083650320	0.021255708	0.0206462813025285
0.241517936	0.044068104	0.061301027	0.020649277470168
0.642352647	0.401710210	0.069949367	0.0206597234172772
0.068882526	0.035284846	0.010319808	0.0206776279089226
0.436755128	0.339975685	0.026286932	0.0207072479665658
0.041937101	0.022706709	0.006361353	0.020722446100184
0.087982530	0.056417866	0.010343495	0.0207405087718742
0.749334067	0.589835487	0.056665175	0.0207489458851122
0.146265162	0.085000736	0.020580057	0.0207593496442144
0.759256347	0.565236952	0.062093513	0.020807055538979
0.750550460	0.601969317	0.051329732	0.0209594575657255
0.812227092	0.699827757	0.044212213	0.0210740494282119
0.070324786	0.033023816	0.012496333	0.0211064664105535
0.097625612	0.040637936	0.020523342	0.021107380273932
0.060443907	0.036437434	0.007964689	0.021112367174516
0.030278326	0.018578256	0.003216240	0.0212304500369141
0.094946335	0.047351286	0.014906246	0.0212513265108336
0.029810912	0.018243843	0.003629695	0.0212523561223342
0.108395713	0.047932641	0.017503436	0.0212582190604331
0.080101154	0.041147308	0.012577654	0.0212989024690266
0.040887822	0.022930363	0.005266027	0.0212999097133083
0.087550195	0.053788948	0.011828959	0.0213056271037075
0.039659962	0.020584097	0.005234137	0.0213608011960965
0.115350134	0.036124909	0.024962561	0.0213736703489097
0.129766281	0.055777144	0.022779076	0.0213919700399425
0.039677664	0.020186305	0.005444447	0.0214045990047118
0.931211610	0.878893002	0.016081427	0.0214209737236636

0.067744666	0.049790743	0.005131980	0.0214504319131176
0.615472777	0.526901897	0.027541353	0.0214699957903696
0.072164102	0.043824993	0.008484396	0.021478326099803
0.084650566	0.043716317	0.013133659	0.0214805148829942
0.672076399	0.575358274	0.029952075	0.0215466545431954
0.246614329	0.067779494	0.053989500	0.0215598474208037
0.752951042	0.659931937	0.034976191	0.021606317804244
0.083306230	0.022965140	0.017526202	0.0216652491420836
0.393252084	0.303496704	0.025299011	0.0216730335961471
0.069692161	0.035627495	0.009145304	0.0216748943614515
0.208731602	0.138771144	0.022588530	0.0217078048405256
0.122936141	0.057587577	0.019697050	0.021724602887619
0.519319624	0.381771424	0.042814734	0.0217844104128742
0.917379155	0.837943804	0.023929398	0.0217925034278365
0.048484108	0.034709716	0.004714254	0.0218003039564082
0.189257482	0.045570530	0.039780841	0.0219264063292433
0.118497231	0.082234648	0.013795305	0.021943583189404
0.926835598	0.872620472	0.016798094	0.0219436954336862
0.468158593	0.363579620	0.034594283	0.0219622310172265
0.037626249	0.017493487	0.006615056	0.0220051303265305
0.325360424	0.098308838	0.067830319	0.0220074398680968
0.083472903	0.047212498	0.011765112	0.0220628591261218
0.049551276	0.022882210	0.009076149	0.0221016233103918
0.071582331	0.039172989	0.009233897	0.0221542536921559
0.091648508	0.064246094	0.008175278	0.0221692272541724
0.198341834	0.028754988	0.051409314	0.0222227582865891
0.084860841	0.041721543	0.012760599	0.0222623463119753
0.597781005	0.442548371	0.046452408	0.0222673376669344
0.083694017	0.034978576	0.018087982	0.0222723721310591
0.054948774	0.029354932	0.008015911	0.0222848986627869
0.272252245	0.135443170	0.043777613	0.0222864053735494
0.075295289	0.040916608	0.010481364	0.0223060597482635
0.641921000	0.540650159	0.029956531	0.0223504021623599
0.047322491	0.025481741	0.007701757	0.0223609456742544
0.051244990	0.029667867	0.006460820	0.0223891569357358
0.808842161	0.535554458	0.089631191	0.0224029205588696
0.498151873	0.400401041	0.035513276	0.02241111389106
0.045704140	0.024261288	0.006483813	0.0224381249985383
0.042299390	0.025332674	0.006279312	0.0224653499514859
0.909332269	0.666064212	0.067395393	0.0225024059682912
0.095932847	0.051460975	0.013508822	0.022521465976724
0.098332869	0.031552678	0.018430010	0.0225443703713969
0.183015013	0.050297025	0.038697267	0.0225557616249685
0.057134775	0.023738432	0.009155395	0.0226062019867361

0.058773118	0.031759488	0.007492411	0.0226730239324073
0.653999079	0.423107659	0.079722285	0.02268373616423
0.031940697	0.020441472	0.004107670	0.0227160259642141
0.060261143	0.032855544	0.007667733	0.0227634501499984
0.224149879	0.155845264	0.022425610	0.0227790084683036
0.161111513	0.015739912	0.043929573	0.0227811198890778
0.304187075	0.173519897	0.038500139	0.0227943217772808
0.030185992	0.017614392	0.003542465	0.0228263464810172
0.905588206	0.703792223	0.067286438	0.0228281908308987
0.041376480	0.022153780	0.005960901	0.0229278676694015
0.271774155	0.175027169	0.034915052	0.0229485493858093
0.033165951	0.018660872	0.004157590	0.0229551740693952
0.051112977	0.018675697	0.009515564	0.0229601297284006
0.062720149	0.040220439	0.007716061	0.0229667070075937
0.289266414	0.084101250	0.080694041	0.0229803660153069
0.043745610	0.021116208	0.007223750	0.0230466495367227
0.914085254	0.850824995	0.021981007	0.0230630190259586
0.339488338	0.068704549	0.076549277	0.0230913315491318
0.224835256	0.096560018	0.033444204	0.0231020921746631
0.964001858	0.918664429	0.012289135	0.023155922448092
0.037899729	0.021204211	0.005896442	0.0231840354688223
0.226621615	0.063530639	0.051068868	0.0232163748502045
0.069412924	0.041960605	0.009464902	0.023247531001848
0.071373153	0.048458634	0.006507464	0.0232648129831277
0.088870161	0.030824654	0.016252874	0.0232778227674924
0.052062301	0.028593972	0.006840169	0.0232940625309216
0.055610221	0.031721296	0.007458964	0.023299676391563
0.379014683	0.243885003	0.041331937	0.0233901109191998
0.678000048	0.579604554	0.035634094	0.0234157156108586
0.031607110	0.020118308	0.003600895	0.0234422606968295
0.141697970	0.062847292	0.029804474	0.0234500405591922
0.058174645	0.030066075	0.009242647	0.0234728582328781
0.958531220	0.872095450	0.029592162	0.0235018358080887
0.070570605	0.039938331	0.010010538	0.0235273712349329
0.060007601	0.035598168	0.006917290	0.0235536464761444
0.103446081	0.033310898	0.018972415	0.0235716014207425
0.220446849	0.123688818	0.025972023	0.0236631632731551
0.822419116	0.720148921	0.036516081	0.0236661264037515
0.875332233	0.761919821	0.033895189	0.0237052213851015
0.160682798	0.059079635	0.034016896	0.0237099643303683
0.840398772	0.626654152	0.064564068	0.0237110845758604
0.414451869	0.297647254	0.036061948	0.0237193989803057
0.065458760	0.046077327	0.005739097	0.023719917709485
0.225210413	0.087186376	0.048960246	0.0237314816162468

0.132457109	0.034277610	0.028024059	0.0237554900212291
0.079990552	0.039607539	0.012857612	0.0237859738261489
0.094003405	0.051042097	0.013836410	0.0238138252128177
0.349867572	0.273037267	0.027386738	0.0238957030673844
0.061373682	0.032331065	0.008698417	0.0239052450562804
0.032658306	0.018167929	0.004250649	0.0239060153051376
0.596169374	0.474903596	0.045428532	0.0240083123956663
0.873355763	0.717887395	0.043148561	0.0240501891326704
0.105950925	0.045013212	0.018192327	0.0240628372922371
0.145660456	0.042903311	0.032807292	0.0241531588568506
0.905362985	0.799155758	0.027930934	0.0241598883783852
0.066520708	0.029892020	0.011674665	0.0241764809837784
0.071664239	0.037554090	0.010867081	0.0242324619922844
0.088235441	0.056050932	0.010141833	0.024238710644857
0.795254868	0.531371307	0.082490088	0.0242488716734136
0.233130075	0.065960328	0.044055235	0.0242494550256661
0.644610426	0.494635106	0.040142075	0.0242756315241321
0.267542183	0.097528299	0.050083924	0.0243595176850158
0.147259268	0.092836086	0.020498507	0.0244165040751384
0.604191461	0.304357447	0.095735085	0.0244445144633443
0.047479175	0.024349541	0.008210137	0.0244550861281557
0.084804294	0.052964001	0.009474241	0.0245133581683542
0.760976937	0.603137021	0.050017513	0.0246070946660192
0.915225140	0.872659778	0.014856681	0.0246169245226548
0.782290633	0.676390798	0.038948971	0.0246633368285593
0.172291254	0.099872883	0.022749108	0.0246664175915794
0.572105683	0.420143331	0.051969080	0.0246695562156559
0.579552100	0.349550702	0.070593750	0.0246805468842675
0.050899019	0.021349602	0.008429742	0.0247077987201352
0.670878500	0.448788292	0.063106654	0.0247215031297704
0.043584246	0.024445799	0.006510227	0.0248292233224738
0.141190380	0.062409072	0.024362700	0.0248358902463987
0.067214713	0.040363909	0.007752781	0.0248809997399016
0.062269645	0.034820959	0.009489544	0.0248873467293712
0.095693177	0.059026525	0.010749038	0.0249299075918746
0.249237379	0.174297918	0.028890712	0.0249448590374974
0.055237608	0.028584464	0.007915116	0.0249471063934398
0.075736099	0.045532851	0.009613066	0.0249505999964187
0.680165438	0.578421287	0.034517836	0.024958806881042
0.366781849	0.188359880	0.057785666	0.0249618627323756
0.060839961	0.030183698	0.010349265	0.0249992594532006
0.424915352	0.293003225	0.040579395	0.0250552020484065
0.054748981	0.022848030	0.008607211	0.0250553819366593
0.053146045	0.023934368	0.007594462	0.0250594620917861

0.048852265	0.025315261	0.007044982	0.0250805407428363
0.108883398	0.032541829	0.023577303	0.025096860816334
0.641811838	0.490627991	0.042438367	0.0251185641687526
0.872659647	0.828968015	0.014065951	0.0251389586934203
0.551838368	0.389516740	0.052529789	0.0251596109238522
0.079227551	0.020274041	0.018506052	0.0251662704229684
0.059821413	0.040220549	0.005995372	0.0251692529929303
0.661724599	0.503557511	0.054253634	0.0252500561972246
0.099624510	0.044282433	0.018192612	0.0252659216616001
0.253654572	0.082009089	0.053046925	0.0252741285844369
0.602384711	0.420649785	0.065149078	0.0253064509146545
0.144308822	0.059244295	0.025172553	0.025403125904113
0.114789373	0.070044440	0.014754470	0.0254624775932606
0.069712010	0.033836045	0.010069720	0.0255098018300053
0.146709531	0.066220842	0.023936579	0.0256232034633737
0.046244300	0.026892292	0.005335912	0.0256329188345413
0.334845672	0.233836949	0.032956426	0.0256642952123516
0.435771038	0.246577386	0.059889657	0.0256690035079852
0.190261716	0.125293695	0.025021827	0.0257385872598453
0.042983092	0.018078940	0.007234524	0.0258738991730055
0.084061540	0.046009320	0.011332973	0.025883360859801
0.936674395	0.828815413	0.034029986	0.0259426280901095
0.049825110	0.026476682	0.007111358	0.0259473539787406
0.931050383	0.865506056	0.021193093	0.0259660565821265
0.208257543	0.139094280	0.018743955	0.0259780108258298
0.933133489	0.864050182	0.020661434	0.0259828651349175
0.144225429	0.036301705	0.029595858	0.0259885834809514
0.678975996	0.543305906	0.039067235	0.0260237454597098
0.047289515	0.016356390	0.009084003	0.0260352028298974
0.033600785	0.017946257	0.005772933	0.0260402420240661
0.846977388	0.714397512	0.049392063	0.0260806741697822
0.910095378	0.826938795	0.032791582	0.0261537464308866
0.054672121	0.032522841	0.007712863	0.0261581432305053
0.908531628	0.872217268	0.012864593	0.0261836536912497
0.057879704	0.037969032	0.006785854	0.0262006196592861
0.069595448	0.024314429	0.012325449	0.0262318909262019
0.045974606	0.023992781	0.006540757	0.0263005111610317
0.037671787	0.018612305	0.006015477	0.0263160558294526
0.073283087	0.029333174	0.014589237	0.0263236054373952
0.070747386	0.036571809	0.009720261	0.0263430789705593
0.068552828	0.037960624	0.009832045	0.026380311890739
0.608358567	0.479787824	0.042750222	0.026396426201033
0.876725144	0.793000575	0.035747863	0.0264503392257902
0.819538056	0.728301271	0.032521780	0.0265579711712736

0.322698834	0.192534803	0.042217151	0.0266081241253712
0.222548299	0.058869948	0.049037414	0.0266465520479392
0.184023496	0.076109827	0.031860443	0.0266541951050365
0.848808650	0.659615375	0.063513687	0.0266795993093905
0.840901397	0.751742432	0.031272507	0.0266842871121545
0.383259554	0.180988991	0.061017123	0.0266944338870939
0.187633459	0.053169593	0.036605167	0.0267851938860927
0.691592540	0.598083499	0.029806545	0.0268097514476637
0.645059931	0.460833800	0.061634839	0.026847253629015
0.308558444	0.202846352	0.036101047	0.0269195750653631
0.530997918	0.277726618	0.085127866	0.0270507982361238
0.113304920	0.037335346	0.021565601	0.0270915735031159
0.974618948	0.943384640	0.009089930	0.0271000753196522
0.345397025	0.266037845	0.029028004	0.0271092992852302
0.043050791	0.024055207	0.006070443	0.0271160176986566
0.700654854	0.464458117	0.063916335	0.0271577574502937
0.183667720	0.030725603	0.044654776	0.0271946067270135
0.054283473	0.033929253	0.006823024	0.0272378206465397
0.159700305	0.053293398	0.037033686	0.0272538300540407
0.793887908	0.590907424	0.065912930	0.0272732589138
0.928834903	0.744395192	0.056176152	0.0273155763641395
0.117188536	0.068462690	0.015864126	0.0273438406380795
0.043813167	0.030230597	0.004900014	0.0273501233861733
0.078676634	0.047840258	0.009532490	0.0274006408267307
0.219997946	0.128057400	0.033738146	0.0274580831525768
0.790829333	0.590623588	0.055914163	0.027494900473348
0.306996500	0.176605601	0.037050362	0.0275073175413154
0.680156205	0.570489440	0.037762642	0.0275405326588198
0.180900255	0.070279565	0.038533997	0.0275657710713657
0.067722153	0.045360491	0.006447832	0.0275688377411303
0.065790298	0.031671014	0.009965508	0.0275852093002778
0.325508085	0.252342186	0.021623432	0.027592591067299
0.907243371	0.784408524	0.041197282	0.0276016865650595
0.146049485	0.101740587	0.013941722	0.0276385963746257
0.780627708	0.635368510	0.049334637	0.0276413404215691
0.033002751	0.018229861	0.004586208	0.0276821059157054
0.101890950	0.068012129	0.011757790	0.027706946157545
0.077002096	0.052658741	0.008898119	0.0277268833559013
0.633947487	0.536546343	0.032568883	0.0278003840210977
0.038979288	0.021283085	0.005257931	0.0278135840760132
0.139002928	0.041294231	0.032160588	0.0278316153599249
0.250880551	0.089856035	0.054420801	0.0278370261221242
0.716113616	0.647043113	0.027535701	0.0278827570558474
0.116065500	0.066455057	0.014726571	0.0278828459867182

0.754919875	0.624173956	0.044235608	0.0279038774370337
0.634041315	0.429765110	0.084122907	0.0279278746723432
0.871932624	0.686647401	0.064169042	0.0279521271360062
0.058770012	0.034902879	0.007942189	0.0279667555535214
0.053126446	0.029552098	0.007156421	0.027967731183522
0.836749669	0.646601984	0.062717539	0.0279687318231935
0.296925507	0.125314608	0.055001891	0.0280114275474113
0.460392583	0.283036701	0.051313077	0.0280123269280203
0.244349197	0.095579688	0.049400769	0.0280674754895327
0.089926071	0.043737300	0.014505865	0.0280826690735083
0.050590297	0.028443672	0.007329363	0.0280955380573825
0.038659831	0.022980611	0.004822702	0.0280991196644428
0.124783231	0.070996533	0.020503132	0.0281072555297951
0.045670340	0.022978356	0.007033281	0.0281188691818509
0.193756165	0.108260015	0.025725742	0.0281353417579964
0.270374450	0.054595833	0.061509394	0.0281415612887181
0.045364238	0.024099644	0.007268241	0.02820756463859
0.062770978	0.022773805	0.012412127	0.0282148871417114
0.878004430	0.812914694	0.019219617	0.0282394089465142
0.927093231	0.877824735	0.014694808	0.0282445804667189
0.095872227	0.056381726	0.014857023	0.0282608617222549
0.339770499	0.162782256	0.052281392	0.0283181698331215
0.056910800	0.026867644	0.010206437	0.0283364626729311
0.082770497	0.030060771	0.015674031	0.0284778819404799
0.089711770	0.054144080	0.010760466	0.0285192915704629
0.148989476	0.045257807	0.029677323	0.0285822759561813
0.031229238	0.018649130	0.003697492	0.0287065455298823
0.103061670	0.053072926	0.015428496	0.0287344256882171
0.053996116	0.027196406	0.007499019	0.0287822052708811
0.043801413	0.022231235	0.005663365	0.0288389392049734
0.783597001	0.655616852	0.038567156	0.0288454814339084
0.058086581	0.035511593	0.007142696	0.028965792938431
0.040934002	0.030895933	0.003391333	0.0289905329953721
0.155448940	0.073843271	0.025822123	0.0290035674222525
0.029084390	0.018288883	0.003351630	0.0290048310449249
0.040175458	0.028575234	0.004166671	0.0290245902653814
0.150726300	0.100318838	0.016296693	0.0290830221295296
0.361295444	0.270199230	0.034353656	0.0291410425175539
0.073905168	0.027364463	0.012141688	0.0291728472900066
0.156253294	0.077903290	0.022412446	0.0291728933406473
0.062548537	0.030652103	0.010518971	0.0292035862598421
0.902833352	0.816490654	0.025525684	0.0293059253896136
0.077805065	0.040957699	0.010603538	0.0293135497957495
0.475339980	0.061218654	0.124665522	0.0293254101344079

0.910226171	0.808744288	0.038412378	0.0293739211177577
0.871207339	0.798819358	0.023083745	0.0293755342731696
0.892264428	0.770302459	0.040208815	0.0293818760500654
0.097549674	0.057521055	0.013750041	0.0294123337956057
0.065355071	0.030291927	0.011421322	0.0294362216849351
0.422447679	0.344124641	0.028509816	0.0294501235228506
0.794690296	0.663959571	0.037986953	0.0294826102131253
0.102289726	0.058773377	0.011873978	0.0294943809786658
0.046723398	0.020109375	0.008650087	0.0295861624730535
0.856637212	0.721559722	0.044927541	0.0295939532635465
0.687766455	0.555845235	0.051952152	0.0296204982653498
0.060303878	0.030331786	0.009524155	0.0296335687808198
0.187689659	0.043429473	0.055921950	0.0296820207780706
0.080180086	0.052103054	0.009229955	0.0296944123770175
0.896657353	0.772339324	0.037454449	0.0296961336015509
0.033410233	0.020549588	0.004672923	0.0297018204488313
0.118011258	0.055207696	0.017504187	0.0297337611717554
0.914449235	0.827604774	0.024646321	0.0297355164886444
0.117637460	0.045725254	0.021362407	0.0297570567513781
0.849325354	0.776725431	0.022488997	0.0298464242501323
0.046171296	0.024470790	0.007011076	0.0298836323240802
0.108900375	0.048922942	0.020552166	0.029885737419159
0.277860956	0.072935979	0.066063833	0.0298936372683832
0.057924075	0.031044689	0.008730872	0.0298986358902837
0.030189715	0.020440845	0.003337435	0.029911135307631
0.903973641	0.848495750	0.017202130	0.030001719301012
0.889129250	0.743263164	0.044797576	0.0300215194764539
0.526444666	0.407192324	0.041002700	0.0301027548216017
0.139518019	0.066655763	0.021296997	0.0301656845976562
0.068182731	0.041475916	0.007971704	0.0301722320156723
0.236732229	0.113602032	0.037189587	0.0302080296629943
0.037267334	0.021278301	0.004464811	0.030251648991772
0.667195706	0.451808485	0.065895246	0.0302918149635874
0.134848994	0.077247666	0.015662352	0.0303435932144545
0.834885192	0.536098682	0.082959870	0.0303508156175993
0.508507080	0.275386558	0.080488502	0.0303667455783365
0.074810194	0.029082329	0.015219399	0.0303933901120575
0.099050051	0.041061726	0.019553890	0.0304014726431437
0.280003326	0.175842923	0.037032107	0.0304112713828593
0.748991257	0.519918309	0.079487987	0.0304126062174018
0.050512430	0.018405836	0.008965380	0.0304145966636317
0.057928950	0.034636576	0.006445833	0.0304269795470584
0.063453366	0.033294819	0.007549710	0.0304430332299824
0.043567651	0.027330356	0.004985892	0.0304640780687239

0.037863428	0.022965320	0.004822184	0.0304889808713128
0.049700271	0.035288905	0.005527656	0.0305033333898914
0.027500172	0.016739094	0.003004944	0.0305312120077129
0.303226942	0.067738515	0.066156407	0.0305700953265343
0.903850593	0.716275858	0.058594200	0.0306211101416124
0.055265186	0.030346120	0.008914170	0.0306388296562019
0.646439086	0.495190747	0.049414999	0.0306443768478588
0.136414821	0.066621928	0.018505317	0.0306517946455678
0.091212130	0.072396121	0.007593405	0.0307052502273187
0.553824309	0.434765023	0.040981749	0.0307286480131575
0.660351320	0.543250342	0.037470836	0.0308082680812982
0.928168531	0.815156098	0.038992695	0.0308119782101639
0.382265953	0.237684391	0.044650147	0.030862884630402
0.650768003	0.404371411	0.066741589	0.0308735633068709
0.706129754	0.577866459	0.038966388	0.0309143028575335
0.037079381	0.020135719	0.005123959	0.0309405801133405
0.040896654	0.026146196	0.005080859	0.0309422667457324
0.091621473	0.047966165	0.012193313	0.0309471883605986
0.076018752	0.054438969	0.007147732	0.0309666015624582
0.861069115	0.736775744	0.038712650	0.0309704927772881
0.035709083	0.022813127	0.004013043	0.0309725607237392
0.899257403	0.800530479	0.029797879	0.0310043432105328
0.047643792	0.027137114	0.008208899	0.0310430730397713
0.039004436	0.022915297	0.005515062	0.0310742102013133
0.077412025	0.032782191	0.013676444	0.0310998822517278
0.180590845	0.048804781	0.036119970	0.0311428848376905
0.041235298	0.020260427	0.006354320	0.0311546724568469
0.094482376	0.054132156	0.013036751	0.031165175682614
0.397368129	0.032495822	0.107538482	0.0311753523435123
0.660921028	0.459025857	0.064351044	0.0312245882400205
0.914776796	0.871021577	0.016734648	0.0313103074852464
0.679896856	0.529442474	0.055210591	0.0313527493412049
0.562070621	0.441368492	0.041761906	0.0313627378357401
0.885272117	0.744243677	0.050905973	0.0314398290730144
0.918172158	0.783523114	0.039669269	0.0314457898168414
0.941887389	0.878913348	0.017804891	0.0314484094430771
0.656358036	0.375569953	0.075268411	0.0314532793287818
0.166281633	0.042729197	0.038585639	0.0314754807860885
0.583217792	0.335798766	0.078352989	0.031481621485447
0.493920562	0.380423277	0.043596667	0.0315261059008997
0.431875193	0.241798815	0.068073496	0.0315622255927166
0.174963045	0.121247273	0.018496047	0.0315966080266857
0.932312550	0.815494290	0.035565439	0.0316124503074682
0.900828753	0.775250183	0.041153974	0.0316254332148791

0.026672554	0.016541661	0.003074434	0.0316438301542488
0.059425833	0.033140250	0.008439834	0.0317155447842439
0.322969387	0.176281378	0.041543533	0.0317591487394932
0.182002053	0.064597879	0.040954072	0.0317680904644287
0.072618862	0.043319406	0.011036014	0.0317818655685939
0.386982653	0.317587946	0.025536131	0.0317838891642357
0.128838374	0.054987109	0.021159626	0.031831994365364
0.037222687	0.018701530	0.005257680	0.0318328777699987
0.049489142	0.024828070	0.008004658	0.0318394735000194
0.175754293	0.047847015	0.041342239	0.0318560542456852
0.028680585	0.015965286	0.004346515	0.0318729261509329
0.059296697	0.027083970	0.010162376	0.0318793190957185
0.136954959	0.064142823	0.021921138	0.031935326542527
0.141180425	0.082327979	0.016733464	0.0319557702438481
0.355472601	0.027709204	0.158743002	0.0319567998525433
0.042132703	0.023828493	0.005357410	0.031982499398067
0.069929399	0.044549447	0.009774875	0.0320076680706314
0.822783384	0.648964829	0.059377185	0.0320728273509032
0.051041742	0.027390216	0.006437988	0.0320876143004798
0.035511590	0.020061681	0.005327676	0.0320959200799968
0.087916200	0.063412069	0.008038565	0.0321058465758498
0.870314264	0.504278008	0.132411719	0.0321552660169134
0.952867232	0.925326366	0.008913533	0.0321872553941352
0.760145735	0.620252022	0.045336115	0.0322070296276401
0.084220836	0.050499798	0.011839585	0.0322190463216666
0.055402749	0.025303072	0.010082924	0.0322243833028259
0.943872562	0.795164693	0.047129213	0.0322314758120628
0.085195448	0.039158926	0.012935121	0.0323274835294557
0.684265851	0.179811757	0.143806617	0.0323522508400141
0.760823178	0.654574273	0.034155927	0.0323584272760439
0.909636607	0.817024306	0.032592544	0.0323733723549653
0.057099829	0.019585968	0.012316388	0.0323878921624669
0.806106144	0.709111385	0.029429153	0.0324319076586112
0.029971540	0.022226291	0.002299926	0.0325100977261119
0.778666798	0.658429183	0.038300117	0.0325158235924078
0.039170972	0.020905242	0.005942482	0.0325374077052648
0.897858233	0.807901357	0.030731509	0.0325591267142531
0.075460052	0.036961711	0.011572129	0.032560963984423
0.130036139	0.061134397	0.019249369	0.0325683495941493
0.863008064	0.752049895	0.034490979	0.0325804530504468
0.849706451	0.731661402	0.037240054	0.0325888327977569
0.842035568	0.787984894	0.019201127	0.0326015292019592
0.471674735	0.055255036	0.157373545	0.0326273426051868
0.132121789	0.033111687	0.028387986	0.0326567446070592

0.039298033	0.027377616	0.004299452	0.032658409768729
0.129959616	0.054087810	0.025288620	0.0327542820908278
0.137579421	0.107792709	0.011128750	0.0328544153136874
0.047879591	0.024192845	0.008031006	0.03290095835876
0.119105909	0.085447930	0.009813309	0.0329168545520188
0.145065626	0.069042702	0.026153040	0.032955074072084
0.051963768	0.034571817	0.006039806	0.0329913480165571
0.170033371	0.124813356	0.016552534	0.0330481433229936
0.093361713	0.037364755	0.016424666	0.033117064990972
0.545163009	0.413944915	0.037491092	0.0331171376523352
0.039327949	0.023955570	0.004934749	0.0331188328576273
0.297793457	0.186564630	0.038827234	0.0331219402435072
0.824922706	0.673178055	0.049590137	0.0331673969409156
0.124210510	0.065270300	0.020607219	0.033186604501665
0.659984501	0.390165389	0.086381782	0.0332107924043196
0.090688229	0.057484362	0.012703083	0.0332417022170227
0.043149944	0.029117399	0.004597281	0.0332444425506062
0.084767028	0.057229342	0.008228440	0.0332902876559071
0.209390375	0.136009452	0.019879988	0.0333229787990137
0.041853188	0.023125368	0.006397442	0.0333334037125297
0.939228450	0.824676001	0.039052218	0.0333623743070216
0.033176588	0.019034008	0.005179790	0.0333776281494835
0.049455298	0.031725932	0.005064397	0.033456769723924
0.586096671	0.367752212	0.086315586	0.0334700943192499
0.046847028	0.032301907	0.004329091	0.0335103089443767
0.515357606	0.376336288	0.048070326	0.0335621360004858
0.085323150	0.041647887	0.013485704	0.0335802402504857
0.261671526	0.139648998	0.034389418	0.0336045491297123
0.948290243	0.887861506	0.019824144	0.0336064443108954
0.039471974	0.021638457	0.005098492	0.0336751591187276
0.791274594	0.704513274	0.027831635	0.0336805491248776
0.096407370	0.028439220	0.021876991	0.0337347691051245
0.046992451	0.029156778	0.004804876	0.0337477820324679
0.659541444	0.502473880	0.051603587	0.0338074138036306
0.951911578	0.860282359	0.028627090	0.0338119184178014
0.153716241	0.031073532	0.042495961	0.0338155838009359
0.296346791	0.075482551	0.066530274	0.0338516588903938
0.063875521	0.030985374	0.008756435	0.0339669786860434
0.203296434	0.050230538	0.044271069	0.0339736589277801
0.374050858	0.247836822	0.035540175	0.0339769653975255
0.022721007	0.015978631	0.002421385	0.0340429193972979
0.526216092	0.309097159	0.072818039	0.0341602489478647
0.344256805	0.262380475	0.023201001	0.0341942571765204
0.225278275	0.159835182	0.022171857	0.0341958023226013

0.677295911	0.518673954	0.060096050	0.0343676212323385
0.542747882	0.392430404	0.059069699	0.0343884442649538
0.413316237	0.300661873	0.036101424	0.0344089433844698
0.090575206	0.066604085	0.008555965	0.0344255361950382
0.286237879	0.193995580	0.027550531	0.0344347513698579
0.646940769	0.467741076	0.052008806	0.0344746307401967
0.070338209	0.041135965	0.008954472	0.034493997078544
0.123219436	0.071299841	0.019204305	0.0345486927986527
0.039993184	0.025556392	0.005051649	0.0345509627742076
0.071304406	0.046830178	0.007963747	0.0345732143205267
0.908238707	0.862071284	0.014098522	0.0347599221321301
0.911691140	0.866128356	0.015148534	0.0347836452827844
0.090097404	0.032374482	0.017721944	0.0349199963948511
0.832557502	0.712689920	0.043098093	0.0349332907981361
0.047994385	0.028488113	0.006105790	0.0349567782038591
0.035798075	0.020346969	0.005892068	0.0350709721698564
0.164638888	0.040243195	0.032660534	0.0350861641461137
0.123815245	0.053548865	0.019878161	0.0351693202942509
0.409275353	0.236624449	0.067855106	0.0352038024404823
0.154480422	0.079432884	0.024201231	0.0352277841534566
0.953355190	0.926112521	0.007910795	0.0352412383332
0.901812204	0.841607920	0.017876651	0.0352916166231785
0.655922926	0.538699987	0.037646035	0.0352928582604551
0.047298687	0.024928615	0.006394605	0.035294716751857
0.726127706	0.594774986	0.049574229	0.0353194756277851
0.043500595	0.021211148	0.006464289	0.0353347347460882
0.239612647	0.106323229	0.041934474	0.0354055830135955
0.025295976	0.017371533	0.002447047	0.035454865200427
0.051754368	0.034644262	0.005673488	0.0354604102392939
0.086574049	0.038173606	0.014461565	0.0354864468346835
0.919263866	0.799271686	0.037106371	0.0355710712202166
0.947810996	0.819037221	0.045456222	0.0355720903326989
0.069701549	0.044680508	0.008151186	0.0355740226334119
0.051565584	0.025137334	0.007865548	0.0355806299349259
0.052954521	0.028677919	0.006317608	0.0356348661400371
0.924824462	0.771862871	0.048330266	0.0356472857429619
0.947451234	0.880748153	0.019131601	0.0356587836641657
0.580285878	0.446615440	0.042603230	0.0356827514545313
0.466622315	0.373266968	0.028639965	0.0356863330354813
0.062350024	0.035451585	0.008437987	0.0356985928717757
0.298624877	0.204804136	0.027114027	0.0357022953788968
0.066786195	0.032518434	0.010154950	0.0357035542186572
0.615329905	0.533623885	0.029312940	0.0357245716258991
0.045865987	0.025778181	0.005780375	0.035729582904216

0.782542284	0.672115278	0.031771043	0.0357604288996363
0.904171884	0.840799732	0.019138163	0.0358154331387065
0.091988307	0.029964930	0.018491913	0.0358163874019912
0.613129778	0.467717150	0.050871412	0.0358353223739908
0.859701439	0.710748594	0.046989710	0.0358588939569511
0.059927120	0.036677998	0.006860410	0.0358595008039915
0.173594455	0.068825261	0.031076064	0.0358636743181931
0.823128388	0.703574228	0.036907099	0.0358904006217258
0.023438615	0.014731895	0.002766625	0.0359304512247796
0.928440370	0.817127502	0.037400549	0.0360085478937931
0.100642068	0.057933770	0.014716722	0.0360699880187916
0.838378192	0.756131273	0.028741166	0.0360783722859076
0.854721324	0.727185912	0.044762979	0.0360929403114298
0.839181446	0.575387431	0.105547693	0.0361182783749765
0.905869290	0.022168503	0.332181940	0.0361433500808944
0.050504473	0.037853202	0.004612314	0.036160597857321
0.164633872	0.099628868	0.018855839	0.0361693217848966
0.927740277	0.843637851	0.028447422	0.0361837453042964
0.077273824	0.053786122	0.006638597	0.036238344771747
0.035081175	0.014964294	0.005624955	0.036244575691246
0.928026832	0.841167255	0.025828255	0.0362921683102653
0.976914319	0.961402938	0.005634012	0.0363986815013882
0.062698170	0.039532740	0.007811788	0.0364045369008427
0.914659207	0.854547066	0.020582582	0.0364695522310623
0.035167582	0.018287799	0.005466734	0.0366442044051336
0.154239718	0.094885482	0.021518844	0.0366646395949828
0.065043324	0.039889880	0.007627760	0.0367148166537261
0.967209163	0.934626155	0.012026001	0.0367573225255921
0.869662103	0.677469258	0.063374713	0.036816181738972
0.450298089	0.355707268	0.035767729	0.0368169607962198
0.085324188	0.049095616	0.010922097	0.0369196389533276
0.785415827	0.618231113	0.054532254	0.0369202302389802
0.734789579	0.589379671	0.043161691	0.0369232420069052
0.644231537	0.426945696	0.063234595	0.0369430880393921
0.054995313	0.025592004	0.007622968	0.0370289395939517
0.169493382	0.064653014	0.030278675	0.0370940844882481
0.074963489	0.035848245	0.013243889	0.0371092040746896
0.050275404	0.022718166	0.008176313	0.0371212582067897
0.571005453	0.403862974	0.057970401	0.0371426782588395
0.923430588	0.872071117	0.015828225	0.0371619310970204
0.063733740	0.039731728	0.008218807	0.0371919439682726
0.738493034	0.599504674	0.042260476	0.0372140766739302
0.054336468	0.027673830	0.008865347	0.0372458669981535
0.432508749	0.234219706	0.056258650	0.0372908118404454

0.236656430	0.114637945	0.042734040	0.0373951364985916
0.035512169	0.021257022	0.004367629	0.0374194198410582
0.900914719	0.863952029	0.011974544	0.0374235238749047
0.902594750	0.819422112	0.026979977	0.0374740414196152
0.257091268	0.128343003	0.041878628	0.0374764935529838
0.073716115	0.053028780	0.006084024	0.0374972037007006
0.077410862	0.025341766	0.015384989	0.0375426013898698
0.040800696	0.024469259	0.005209352	0.0375689071791689
0.891790928	0.807306242	0.026306212	0.0375852238969858
0.495943521	0.385186685	0.039358501	0.0376001380241695
0.087265513	0.028820752	0.017005457	0.037624812606834
0.522324299	0.263136655	0.079680979	0.0376310566693901
0.082905502	0.047800892	0.010573094	0.0376329805272878
0.219873251	0.122721721	0.027611622	0.0376493855791978
0.682109608	0.552900399	0.039601241	0.0376580512809444
0.761635224	0.638142857	0.042637244	0.0376708694038773
0.436110428	0.313653323	0.038084083	0.0376831718055553
0.232985938	0.112984178	0.035618899	0.037705802922835
0.145207222	0.058459669	0.030551478	0.0377293311305231
0.085154447	0.042486304	0.014815424	0.0377488948118288
0.026136149	0.015380919	0.003543383	0.0377592240138774
0.526380039	0.317475473	0.063337367	0.037803000694241
0.093833128	0.043714350	0.015520822	0.0378417408219003
0.073519583	0.030399073	0.014110974	0.0378512036631321
0.047315023	0.022811849	0.008250169	0.0378526621247873
0.109480783	0.049546051	0.019275794	0.0379126778343919
0.401628164	0.336229272	0.021468678	0.0379787631376982
0.140087450	0.065310897	0.022915299	0.0379981447280848
0.069465882	0.044733032	0.008707896	0.0379982574656
0.165303912	0.077443760	0.029231286	0.0380115821540902
0.052765921	0.021784029	0.008299351	0.0380841136669909
0.105490135	0.046402907	0.019738388	0.0380965335124833
0.544118788	0.316814434	0.063305741	0.0381576802052766
0.728020484	0.540838911	0.066741510	0.0381916265694515
0.496762771	0.417547790	0.026602514	0.0382390525639072
0.903587782	0.810671987	0.034292547	0.038272662126851
0.105856850	0.050736313	0.015716695	0.0382882737536702
0.105178712	0.051510866	0.015431240	0.0383611014052339
0.034804098	0.017408127	0.004728233	0.0384064576802605
0.237940280	0.155597651	0.025284618	0.0384886729178449
0.931070211	0.888059321	0.013447275	0.0384888671239876
0.025866707	0.018125163	0.003120193	0.0384903750856603
0.031728233	0.018424620	0.004336309	0.0384935949096852
0.071626092	0.036540491	0.012508713	0.0385050560017011

0.070159357	0.046975231	0.006902654	0.0385176966273369
0.052085914	0.023057001	0.007976140	0.0385374916693764
0.045035948	0.025706659	0.007111164	0.0385485135498246
0.866512966	0.731767488	0.042128261	0.0385726557854818
0.054323044	0.037741441	0.004757675	0.038607912955172
0.904227994	0.776274031	0.043887248	0.0386175039466262
0.888186924	0.749108253	0.042514958	0.0386217685780932
0.130437909	0.061765026	0.024772907	0.0387353025932836
0.851851676	0.658989856	0.065029143	0.0387959951118491
0.149816524	0.027989992	0.037116946	0.0388095645217985
0.675144279	0.310951491	0.118251651	0.0388867365960683
0.042120645	0.023377409	0.005722646	0.0389092850604766
0.075966294	0.044175066	0.009361663	0.038925256982818
0.103344300	0.045315992	0.017889087	0.0389341363448653
0.811005578	0.712917535	0.037867590	0.0390584607602788
0.033296573	0.016770260	0.005329667	0.0391016470976953
0.864040862	0.782357580	0.026875277	0.0391718024981034
0.919977002	0.849587061	0.021055202	0.0391898879003681
0.053749617	0.021787316	0.009477623	0.0391900855643242
0.504398529	0.229216972	0.086573279	0.0392155880405792
0.093277200	0.038867678	0.015261006	0.039218665167531
0.072556429	0.032044485	0.012282395	0.0392253488924034
0.945931784	0.893402246	0.016268759	0.0392415532947315
0.053269493	0.023215055	0.008647608	0.0392558319130536
0.840778690	0.682562127	0.049795591	0.0392758283159446
0.289332561	0.188626920	0.031891199	0.0392925317840909
0.215505235	0.156435003	0.019275407	0.0392964084512347
0.179804966	0.085145885	0.027261639	0.0393124044480366
0.432575459	0.232678721	0.080600333	0.0393160728681123
0.795069012	0.588429100	0.069023245	0.0393166531463282
0.058906050	0.032047716	0.009419113	0.0393226929617628
0.502460916	0.368995210	0.044820278	0.0393418135237928
0.078814701	0.048268154	0.011661820	0.039403074509299
0.056604234	0.038021705	0.006191944	0.039511977690834
0.202669221	0.134491738	0.021390953	0.0395283108672444
0.361026531	0.162425464	0.060075840	0.0395970519112402
0.863085689	0.680720983	0.056507401	0.0396055493212054
0.916462034	0.867903701	0.012817495	0.0396150751434131
0.053189358	0.026492441	0.008130383	0.0396208318633633
0.648774980	0.415888717	0.069065907	0.0396456816460992
0.132212211	0.074152107	0.019029948	0.039672037256461
0.872400981	0.793102314	0.022739516	0.0396897779489423
0.313406567	0.163973942	0.045062449	0.039692318286054
0.836186844	0.597610237	0.080416524	0.0397192913272051

0.318617288	0.266554829	0.020348108	0.0397430410865826
0.932671135	0.873511892	0.020351239	0.0397590441951658
0.870574925	0.792580795	0.027292370	0.0397780514823545
0.887539047	0.823056184	0.021738329	0.0397809764573206
0.050115374	0.027526088	0.006783601	0.0397972100770926
0.273044113	0.139216565	0.045982682	0.0398223200856571
0.105301289	0.068444827	0.010037437	0.0398578512271589
0.059716991	0.028554292	0.009854575	0.0398885796136641
0.936105961	0.870281134	0.019767602	0.0399640316493085
0.469569080	0.278010292	0.057061404	0.0399694286930429
0.076186808	0.050386017	0.007307817	0.0399867346286039
0.324726445	0.145409233	0.046644226	0.0399904712669771
0.040440190	0.020357790	0.006623365	0.0399910824027184
0.759445947	0.665841962	0.037035582	0.0400697829034908
0.225927323	0.115095340	0.036574779	0.0400799196748593
0.815278433	0.614442650	0.056537212	0.0401535760961708
0.058203544	0.025897779	0.010512949	0.0401601135648273
0.280812592	0.170307773	0.042355723	0.0401655273553589
0.142856997	0.059895981	0.023679050	0.0401666410257491
0.075588533	0.022498828	0.016870195	0.0401948999273516
0.973702851	0.954089289	0.006258466	0.0402629559037859
0.206760613	0.143947688	0.022898649	0.0402671145618735
0.629709838	0.513144547	0.033333882	0.0403074412728818
0.888605622	0.676510804	0.056850749	0.0403541423919142
0.281500293	0.096300131	0.056175629	0.0403706846338586
0.328506317	0.209327637	0.040515002	0.0403918154443545
0.083808445	0.054072764	0.010566762	0.0403997247588659
0.081528023	0.022057101	0.020420779	0.040503834552423
0.221336865	0.069612930	0.057047711	0.0405355716102564
0.941678073	0.867116567	0.019811244	0.0405902924011564
0.077323275	0.047522909	0.008892581	0.0406119354991279
0.084498944	0.046472788	0.012336360	0.0406252985086161
0.799161991	0.664010653	0.038001104	0.0406504751388831
0.103322587	0.068589169	0.011837381	0.040689976758356
0.653607079	0.438485794	0.071580074	0.0407370960736855
0.592223419	0.421096278	0.053697774	0.0408155220096959
0.899634085	0.805928869	0.032374025	0.0408170552727578
0.229314377	0.132981958	0.033006884	0.0408599828457688
0.700822257	0.599425665	0.027944434	0.0408780498682287
0.215552633	0.149560021	0.022053378	0.0409050096400192
0.445144315	0.336670687	0.039847751	0.0409257946917247
0.644104938	0.577669394	0.021069628	0.0409550762398428
0.829138346	0.769864215	0.018939979	0.0410301992576804
0.348691490	0.279400195	0.025330593	0.0410308569423322

0.029513644	0.018625010	0.003135990	0.0410580624337968
0.162484355	0.052525857	0.037930564	0.041067114467273
0.296690307	0.083821316	0.061385453	0.0411191424549695
0.720083948	0.563016292	0.044272227	0.0411400790387821
0.603657274	0.439386785	0.058922053	0.0411638353757309
0.258132672	0.197307044	0.020161037	0.041184870623187
0.873575693	0.665918029	0.060034402	0.0412751387794675
0.586979311	0.325566184	0.100247617	0.0412764780635064
0.090440279	0.034508012	0.014525379	0.0413185180079358
0.882389013	0.750774838	0.041249518	0.0413203349511876
0.863415647	0.805077673	0.017487357	0.041366043959073
0.325280319	0.207712248	0.042995292	0.0414545637239868
0.812221312	0.621483920	0.064716186	0.0414815844037837
0.923693648	0.782621832	0.051939700	0.0414825443856114
0.048351228	0.026704391	0.008091390	0.0416593378451759
0.759010884	0.517160352	0.072182563	0.0416944071426689
0.077821965	0.032172496	0.013526396	0.0416977595975059
0.081230589	0.058097862	0.006595495	0.0417311904419612
0.043636984	0.023848567	0.005577635	0.0419563075005889
0.771742596	0.582425224	0.057661391	0.0419765244932789
0.961818459	0.904575426	0.019636720	0.0419841878560594
0.134475096	0.064388017	0.023941593	0.0419879748381212
0.716505936	0.513357298	0.062412634	0.0419897479828588
0.245394112	0.141351588	0.029414440	0.0420579937823045
0.572685483	0.449464314	0.040235113	0.0420685303523304
0.035501151	0.022551420	0.004597861	0.0420967869774232
0.927401454	0.797869700	0.048719029	0.042119286995499
0.048344424	0.029003226	0.006161155	0.0421224265489651
0.714720873	0.584779684	0.042215337	0.0421417140529151
0.086081533	0.040187177	0.015240900	0.0421526198834121
0.415322807	0.273138838	0.040071139	0.0421651480161392
0.363450735	0.249603648	0.041731458	0.0421716357781838
0.097372119	0.040530713	0.016960392	0.0422106730464168
0.048835041	0.023502992	0.008547110	0.0422176700990142
0.356429903	0.270283410	0.026344744	0.0422766775240321
0.032109809	0.017763340	0.004262736	0.0423127717432205
0.794885346	0.707500221	0.026482965	0.042317883645269
0.043392946	0.023990100	0.005277172	0.0423617757694512
0.800147901	0.595846781	0.064075248	0.0423621914682373
0.260038259	0.124246709	0.039115447	0.0423920918549063
0.041522831	0.027788501	0.003865522	0.0424328368509317
0.034096137	0.022861695	0.004107542	0.042440205095373
0.098173891	0.070758041	0.009553192	0.0424455991585117
0.852814756	0.657083091	0.056799860	0.0424559869267136

0.555504729	0.404102612	0.052562121	0.0424767898106538
0.909114673	0.843037368	0.018867786	0.0425421905744148
0.602567302	0.436674651	0.053555300	0.04257064122455
0.597654669	0.459375619	0.040588583	0.0426433353216031
0.089106287	0.037855921	0.015535389	0.0426710429172142
0.667873665	0.482052586	0.053497676	0.0426758221236604
0.728160335	0.503134215	0.075217968	0.0427268295419281
0.903974169	0.734622685	0.064890869	0.0427586633431344
0.461996536	0.335270413	0.036896469	0.0428369825373404
0.041371196	0.024566389	0.005564509	0.0428572817633706
0.139656539	0.050840647	0.027548511	0.0428926527164674
0.091848277	0.036923450	0.020957218	0.0428949409273452
0.821769509	0.679346661	0.045209325	0.0429501778694935
0.206276554	0.128504744	0.027150492	0.0429822735477829
0.795203054	0.730912913	0.020416673	0.0430001926466466
0.245578592	0.118560566	0.046636789	0.0430539151829511
0.030469286	0.015338550	0.005108685	0.0431048882832744
0.406347960	0.326563166	0.026764066	0.0432249498428488
0.491191346	0.289264950	0.054661670	0.0432536640228035
0.852854935	0.529838921	0.085938913	0.043295590280085
0.873850609	0.693975826	0.051587422	0.0433160746965177
0.057010711	0.034539483	0.007795696	0.0433618366551909
0.056900596	0.026408499	0.008755472	0.0433809041362177
0.106160727	0.056893008	0.015196818	0.0434086129314063
0.771752940	0.564219069	0.064784404	0.04341318704602
0.502132719	0.374610669	0.043849688	0.0434214676085632
0.216709177	0.128451392	0.031429873	0.043437917017095
0.196687504	0.081189655	0.041212885	0.0434603478760993
0.062469450	0.033127722	0.009812278	0.0435096529155847
0.244174018	0.181852982	0.018753659	0.043526916540427
0.102501521	0.070154504	0.011619463	0.0435437260770734
0.574126522	0.430764793	0.039489908	0.0435647181247213
0.063534688	0.033939576	0.008567101	0.0435722370751206
0.579271914	0.438690553	0.042145053	0.0435731708488255
0.936960444	0.900900555	0.011490700	0.043636691636388
0.938093029	0.868352579	0.022228786	0.0436669208458859
0.448099706	0.356401905	0.025016350	0.0436710476150255
0.043867777	0.015031268	0.007976084	0.0436943545902449
0.918569237	0.838128141	0.022478666	0.0437734740871296
0.059154015	0.033427816	0.007217148	0.0438785064265632
0.878450998	0.773525491	0.033629728	0.0438931031342022
0.767039698	0.583423441	0.055973955	0.0439399116267096
0.154136909	0.052329586	0.032250482	0.0439976260067308
0.059716518	0.033031247	0.007595890	0.0440346812231822

0.069955777	0.036022435	0.009957943	0.0440649074614006
0.721924181	0.536700752	0.062127667	0.0440859172688522
0.968580893	0.871760699	0.030656672	0.0441090538567763
0.039218866	0.021860633	0.005508740	0.0441203633178474
0.063367542	0.024897435	0.010912659	0.0441631080247719
0.890477295	0.616264338	0.088298448	0.044198770484108
0.067834875	0.033173757	0.011129933	0.0442338017888396
0.826375982	0.559472772	0.073214772	0.0442529051912646
0.074105929	0.038031150	0.010423345	0.0442695250196799
0.051902320	0.032005006	0.007115582	0.0442832248836251
0.455787662	0.365873672	0.028413273	0.044290074208245
0.071436496	0.038873164	0.009271833	0.0443138255976198
0.509878180	0.408244892	0.031333912	0.0443205499775305
0.276585565	0.100935355	0.059681166	0.0444092553963332
0.179623682	0.095687131	0.023619909	0.0444137446976809
0.416706217	0.288883751	0.041940004	0.0444654371963357
0.743673564	0.488878911	0.082181592	0.0444937222493857
0.908080342	0.831151580	0.023576546	0.0445006918744937
0.367377179	0.207658184	0.047022311	0.0445369815176731
0.849851340	0.751263904	0.031503068	0.0445643574498618
0.075452001	0.039448436	0.009672382	0.0445681917144318
0.035560256	0.023720924	0.004075428	0.0445759172728998
0.125043635	0.047019284	0.027053888	0.0446717294552177
0.054882512	0.033334183	0.007332207	0.0446906988454089
0.091784131	0.026914547	0.020587724	0.0447018065237594
0.322474355	0.231156826	0.033135918	0.04471166439797
0.148279373	0.063682953	0.024120958	0.0447139338138007
0.048297395	0.021633155	0.008704556	0.0447446541694946
0.249078980	0.126110274	0.038894567	0.0447601093450916
0.754778620	0.598546302	0.044469020	0.0447951237664278
0.049598351	0.034833569	0.005640157	0.0448420815377944
0.937921310	0.911693769	0.009412728	0.0448449936186108
0.181225240	0.063119302	0.035351091	0.0448582487163826
0.122715823	0.031685461	0.029496614	0.0448750521548888
0.713047229	0.605138935	0.034079109	0.0450086399539053
0.280626186	0.156871446	0.031384199	0.0450455410680043
0.136604299	0.049451830	0.025657827	0.0452112351004229
0.465170812	0.315960197	0.048905948	0.0452832967187147
0.516251541	0.262372107	0.085454638	0.0453540122217673
0.834699835	0.691539021	0.048839644	0.0454421557860923
0.087063018	0.031172339	0.016853660	0.0454452700648533
0.073350331	0.041678283	0.011908873	0.0454990956691151
0.495442976	0.377291957	0.041288296	0.0454999660806468
0.869307968	0.746700459	0.037548722	0.0455184431964726

0.090248505	0.030256905	0.018903411	0.0455188257053724
0.044226987	0.027425343	0.005680669	0.0455476692190288
0.046696830	0.025993311	0.005981297	0.0455560159733007
0.041813734	0.028517844	0.003961290	0.0455614573524487
0.069759609	0.030062977	0.011911767	0.0456125325835192
0.079827697	0.045981445	0.010190189	0.0456487145047129
0.107460158	0.037004507	0.023862986	0.0456650672198723
0.103755621	0.030129798	0.021583351	0.04567618796845
0.032302237	0.012897563	0.005433546	0.0457985856765189
0.525564528	0.235531738	0.084898237	0.0458505148353248
0.087894004	0.047417077	0.012212486	0.0458521705886168
0.111002352	0.033569879	0.019730418	0.0458564077143121
0.066378611	0.029156262	0.011184608	0.0459015454530176
0.659585181	0.497276026	0.052410443	0.0459190274306282
0.950666684	0.885236759	0.023676581	0.0459270728680236
0.807155988	0.614560221	0.056008016	0.0459860120185126
0.764743638	0.669310635	0.034475750	0.0459891870453052
0.778986907	0.624695562	0.054078820	0.0460314217871448
0.925000176	0.868429395	0.019565268	0.0460520472398753
0.869526721	0.647201356	0.079496287	0.0461028860920069
0.022147037	0.013410286	0.003080543	0.046124189970438
0.958902346	0.899590306	0.019531787	0.0461247490949789
0.939180957	0.897089627	0.013461247	0.0461584069339548
0.072289028	0.040624959	0.011758646	0.0462062458803172
0.067617851	0.028808230	0.012104272	0.0462149837429115
0.595715792	0.426226761	0.055929448	0.0462320077584919
0.115688939	0.024573194	0.026698085	0.0462761590342182
0.112308642	0.059530612	0.017716545	0.0463123467555401
0.130754421	0.052556099	0.023470528	0.0464482700200989
0.049109587	0.022280929	0.008030086	0.0465341512081049
0.078917184	0.046555717	0.009903105	0.0465639544424913
0.035071417	0.023453959	0.004017926	0.0467553255876441
0.733028052	0.612498860	0.038884395	0.0467638160213345
0.888986594	0.793397552	0.029358698	0.0468363634227325
0.163526765	0.047026287	0.034589815	0.0468995504754023
0.903914689	0.790491917	0.039597508	0.046908088709969
0.204776123	0.116673298	0.029894407	0.0469168702341546
0.754022271	0.531493104	0.078591260	0.0469524211024526
0.066736048	0.029081837	0.013251983	0.0469532932996103
0.103508055	0.030106780	0.021484306	0.0470969685383492
0.426291717	0.312563203	0.038391114	0.0470975146142256
0.650826242	0.444817441	0.063486701	0.0470996412834659
0.823049876	0.638468795	0.056025858	0.0471103454013543
0.047865494	0.022900877	0.008621092	0.0471115926448972

0.101324035	0.041310386	0.016179374	0.0471173462277193
0.529946494	0.395900693	0.041868930	0.0471406587732726
0.043688730	0.029239301	0.004976591	0.0471730504411402
0.029385956	0.017520170	0.003467086	0.0472091441611551
0.606839162	0.478138734	0.044151583	0.0472586286002657
0.718487046	0.623106057	0.032312419	0.0472587989731534
0.046469325	0.029180035	0.005988037	0.0472688541977104
0.796023708	0.657247947	0.047468997	0.04736969037645
0.547546988	0.400965708	0.044610790	0.047414730364128
0.742495887	0.555309309	0.050976898	0.0474258084074531
0.086977555	0.057734271	0.009258667	0.047502575788548
0.659630310	0.497939845	0.044167233	0.0476035476846887
0.785870774	0.658594179	0.041815326	0.047712647792188
0.826911794	0.658028150	0.064917461	0.0477129420339362
0.198983194	0.107181413	0.028265427	0.047817630873715
0.172874391	0.062379648	0.033564479	0.0478354404255053
0.068469071	0.037108533	0.011281786	0.0479376705760988
0.324333403	0.243767970	0.024425722	0.0479580918950901
0.384361152	0.300940059	0.027862293	0.0480260468544265
0.934281532	0.890721532	0.012725459	0.0480339832327335
0.210680263	0.141277939	0.026990031	0.0480420384574054
0.074803304	0.040215168	0.012028208	0.0480645454916346
0.141145140	0.085858684	0.015997637	0.0481135810978321
0.037023541	0.022983118	0.004079171	0.0481482808064864
0.774127489	0.493220253	0.082691421	0.0482374923024455
0.845793278	0.712262649	0.041782907	0.0482512611239366
0.874030053	0.823222339	0.016640728	0.0483407924182921
0.057167619	0.029119518	0.007328584	0.0483628862572969
0.082173889	0.037603703	0.014367689	0.0483638548460149
0.046151621	0.029898320	0.005264437	0.0483924915638751
0.616250451	0.425278131	0.055820516	0.0484626765952426
0.144929000	0.039748957	0.030760317	0.0485064111958001
0.060557227	0.032021746	0.008619066	0.0486216175061583
0.129704117	0.049835522	0.023214517	0.0486458141845022
0.074610469	0.039298224	0.012493762	0.0486494220837098
0.106582522	0.063873343	0.012159260	0.0486759558285448
0.040503978	0.026459555	0.004926146	0.0487074653935655
0.125658477	0.059509743	0.017686246	0.0487137651481747
0.686041801	0.559468031	0.038576694	0.0487364234046789
0.346439293	0.215707570	0.040856585	0.0487627732836765
0.096601061	0.052974853	0.013146879	0.0487726558395335
0.067499394	0.029602504	0.011723975	0.0488112721635818
0.643191575	0.547678847	0.029014433	0.048882099252789
0.062591016	0.031795352	0.009394161	0.0489034199951553

0.063505777	0.018075929	0.012473254	0.04893212970358
0.719845018	0.532601296	0.052042259	0.0489901802437244
0.929526140	0.868228350	0.020624662	0.0490854865219844
0.052390809	0.020278341	0.009590076	0.0491417582427899
0.462336557	0.279893386	0.054270845	0.0491492400098768
0.510153089	0.314982570	0.058104782	0.0491780245372582
0.220524488	0.136895841	0.029227628	0.0491879475208447
0.052543266	0.023468627	0.009258105	0.0492243663423264
0.087756622	0.051248687	0.010818015	0.0493007681584402
0.571965469	0.438878928	0.048868811	0.0493027994624731
0.490619989	0.265693733	0.082126240	0.0493425500268512
0.398495420	0.118299137	0.080939140	0.0493526086873914
0.923195413	0.876335376	0.020733015	0.0493651016447277
0.042754254	0.026510185	0.004415747	0.0493805973921936
0.464179271	0.381606198	0.024296114	0.0493936300496554
0.885909226	0.283252595	0.280296405	0.0494467206599497
0.084574610	0.037282581	0.013570664	0.0494857554907995
0.053278191	0.027295006	0.009702363	0.049575528415524
0.413482501	0.248246031	0.048278890	0.0496434480259802
0.059655034	0.035943639	0.007157033	0.0496839960607885
0.053403167	0.023129607	0.008992341	0.049734425739527
0.027996728	0.014127226	0.003837333	0.0498457310750152
0.701277101	0.553467891	0.051122909	0.0498883097426093
0.075995415	0.038589816	0.012383080	0.0499025720752024
0.802537811	0.560578132	0.075447824	0.0499117237573219
0.385220228	0.041516040	0.102233947	0.0499162274738246
0.059555499	0.033638212	0.008398277	0.0499764269064447
0.586233703	0.426650588	0.048007061	0.0499833560259752

Name	CHR	Probe_SNPs	Probe_SNPs_	UCSC_RefGer	UCSC_RefGer	UCSC_RefGer
cg22662482	11			FEN1;C11orf1	NM_004111;	5'UTR;TSS15C
cg08643007	2			ATF2;ATF2;M	NM_001880;	1stExon;5'UT
cg01109243	16			NDE1;MIR484	NM_0011439	TSS1500;TSS1
cg07925542	16			MIR484;NDE1	NR_030159;N	Body;1stExon
cg01522721	19			MIR1181;CDC	NR_031592;N	TSS1500;TSS1
cg00735591	19			DNM2;DNM2	NM_004945;	TSS200;TSS20
cg13770529	17			CCDC55;MIR4	NM_032141;	TSS200;TSS15
cg01629329	20			NOP56;MIR1	NM_006392;	1stExon;TSS2
cg10271981	19			MIR1181;CDC	NR_031592;N	TSS1500;TSS2
cg03655940	16			MIR484;NDE1	NR_030159;N	Body;1stExon
cg10248302	11			MIR611;FEN1	NR_030342;N	TSS200;5'UTF
cg20041381	1			C1orf27;C1or	NM_0011642	TSS1500;TSS1
cg10387807	1			C1orf27;C1or	NM_0011642	TSS1500;TSS1
cg02690648	8			PINX1;MIR13	NM_017884;	Body;TSS200
cg11168235	3			MIR548G;C3c	NR_031662;N	Body;TSS200
cg21019820	3			MIR191;NDU	NR_029690;N	TSS1500;5'UT
cg11581046	19			MIR639;TECR	NR_030369;N	TSS1500;TSS1
cg06333167	2			PRKRA;DFNB5	NM_0011395	TSS1500;TSS2
cg02585417	12			MIR618;LIN7A	NR_030349;N	TSS1500;Body
cg25102782	2			PRKRA;DFNB5	NM_0011395	TSS1500;TSS2
cg12480658	2			ATF2;MIR933	NM_001880;	TSS1500;TSS1
cg08737296	20			MIR124-3	NR_029670	TSS1500
cg14278808	8	rs75492345		LOC157627;N	NR_024281;N	TSS1500;TSS2
cg17602882	19			MIR1181;CDC	NR_031592;N	TSS200;TSS20
cg27294431	1			TPR;C1orf27;	NM_003292;	TSS1500;TSS2
cg19484886	12			C12orf61;MIF	NM_175895;	TSS1500;TSS2
cg10470963	22			ANKRD54;MI	NM_138797;	TSS1500;TSS2
cg18654971	5			MIR449C;CDC	NR_031572;N	TSS1500;TSS1
cg13149127	1			C1orf61;MIR9	NM_006365;	5'UTR;TSS15C
cg22050893	2			MIR548N;FKE	NR_031666;N	Body;Body;Bc
cg01656221	2			MIR548N	NR_031666	Body
cg17992509	1			MIR760	NR_030621	TSS200
cg00485047	16			NDE1;MIR484	NM_0011439	TSS1500;TSS1
cg13343238	15			MIR548H4;GI	NR_031680;N	Body;5'UTR
cg25851152	10			MIR146B	NR_030169	TSS1500
cg00328051	14			DIO3;MIR124	NM_001362;	TSS1500;TSS2
cg17774634	6			C6orf167;MIF	NM_198468;	5'UTR;Body
cg02975060	14			MIR1185-2	NR_031571	TSS1500
cg17002091	14			SLC25A29;MI	NM_0010393	1stExon;TSS1
cg22537343	19			MIR525	NR_030192	TSS1500

cg12959622	20			SNORD12B;C:	NR_003695;N	TSS1500;Body
cg05249271	14			MIR496;MIR1	NR_030176;N	TSS1500;TSS2
cg22725901	19			MIR518C	NR_030199	TSS1500
cg19045894	2			ATF2;MIR933	NM_001880;	TSS1500;TSS1
cg06401019	5			FAM172A;FA	NR_028080;N	Body;Body;Bo
cg08445226	3			C3orf26;MIR5	NM_032359;	Body;Body
cg01068014	13			MIR17HG;MI	NR_027349;N	Body;Body
cg04288299	4			WHSC2;MIR9	NM_005663;	Body;TSS150(
cg13285968	2			ZNF385B;MIF	NM_152520;	5'UTR;TSS20(
cg02662576	14			MIR495	NR_030175	TSS200
cg26106778	6			RING1;MIR21	NM_002931;	TSS1500;TSS1
cg10866755	2			ZNF385B;MIF	NM_152520;	5'UTR;TSS20(
cg16204151	19	rs60314070		MIR523	NR_030193	TSS1500
cg04735310	17			MIR196A1	NR_029582	TSS1500
cg08515869	4			MIR573	NR_030299	TSS1500
cg03894789	5			MIR874;KLHL	NR_030588;N	TSS200;Body
cg12778580	11			MIR1237;RPS	NR_031602;N	TSS1500;Body
cg07439409	6			MIR548H3;C6	NR_031679;N	Body;TSS150(
cg15987431	1		rs61740910	MIR1182;FAN	NR_031593;N	TSS200;Body
cg23002708	19	rs34998465		ATG4D;MIR1:	NM_032885;	Body;TSS150(
cg19945937	14			MIR665	NR_030617	Body
cg07987890	15			MIR548H4	NR_031680	Body
cg02895602	14	rs76713753		MIR1185-2	NR_031571	TSS200
cg01630479	22			MIR1306;DG(	NR_031706;N	TSS1500;5'UT
cg24823137	19			MIR372;MIR3	NR_029865;N	TSS1500;TSS2
cg12530503	15			MIR9-3	NR_029692	TSS200
cg02287710	14			DIO3;MIR124	NM_001362;	TSS200;TSS15
cg19646028	19			MIR7-3;C19o	NR_029607;N	TSS1500;Body
cg08131204	22			MIR1281	NR_031694	TSS1500
cg12885549	2			MIR548N	NR_031666	Body
cg17109533	19			MIR515-2;MI	NR_030187;N	TSS1500;TSS1
cg13965908	11			PPFIA1;PPFIA	NM_003626;	Body;Body;TS
cg04184179	5			MIR874;KLHL	NR_030588;N	TSS200;Body
cg18384960	14			MIR299;MIR4	NR_029841;N	TSS1500;TSS2
cg22420044	7			MCM7;MCM7	NM_005916;	Body;Body;TS
cg18588811	14			MIR377	NR_029869	TSS1500
cg26039305	11			MIR670	NR_031577	TSS1500
cg18319687	5			MIR1229;MG	NR_031598;N	TSS200;Body;
cg10822545	19			MIR181D;MIF	NR_030179;N	TSS200;Body
cg06083642	7			CHPF2;MIR67	NM_019015;	Body;TSS150(
cg00565075	22			MIR659;EIF3L	NR_030396;N	TSS1500;TSS1

cg08508227	20			C20orf166;M	NM_178463;	Body;TSS200
cg16621560	13			MIR548F5;DC	NR_031646;N	Body;Body
cg15085006	3			DHX30;DHX3(	NM_138615;	Body;Body;TS
cg26171815	5			MIR580;LMBI	NR_030306;N	TSS1500;5'UT
cg10715527	8			ANK1;MIR48(	NM_000037;	3'UTR;TSS200
cg07126399	14			MIR412;MIR5	NR_030155;N	TSS1500;TSS1
cg23257859	7	rs2923266		MIR550-2	NR_030320	TSS200
cg14892570	19			MIR1470;WIZ	NR_031716;N	TSS1500;5'UT
cg01572694	17			MIR10A	NR_029608	TSS1500
cg17594424	15	rs11555369	rs1049531	MIR1282;SER	NR_031695;N	TSS200;3'UTF
cg00283662	19			MIR642;GIPR	NR_030372;N	TSS200;Body
cg11032038	16		rs80101105	MIR1826	NR_031727	TSS200
cg18645493	1			MIR190B	NR_030600	TSS1500
cg19537184	19			MIR520C;MIF	NR_030198;N	TSS1500;TSS2
cg16572540	19			MIR24-2	NR_029497	TSS200
cg09393453	19			MIR517B;MIF	NR_030205;N	Body;TSS150(
cg16865908	11			LOC399959;N	NR_024430;N	Body;Body
cg09484214	19			MIR372;MIR3	NR_029865;N	TSS200;TSS15
cg09522706	7			MCM7;MCM7	NM_005916;	Body;Body;TS
cg01273384	20			MIR1257	NR_031658	TSS1500
cg09994773	1			MIR34A	NR_029610	TSS1500
cg11915671	19			TUBB4;MIR22	NM_006087;	Body;TSS200
cg20333067	17			ZNF207;MIR6	NM_0010322	TSS1500;TSS2
cg27633139	4			MIR548I2	NR_031688	TSS1500
cg07429629	14			MIR494	NR_030174	TSS1500
cg16745104	20			MIR941-1;MI	NR_030637;N	TSS1500;TSS1
cg22806002	11			BTG4;MIR34(	NM_017589;	TSS200;TSS15
cg24260710	22			MIR33A;SREE	NR_029507;N	TSS1500;Body
cg04155485	7			MIR589;FBXL	NR_030318;N	TSS1500;Body
cg20206204	12			MIR614	NR_030345	TSS1500
cg04114269	3			C3orf26;FILIP	NM_032359;	Body;5'UTR;B
cg08264885	8		rs78882264	POLR3D;MIR3	NM_001722;	Body;TSS150(
cg12846583	3			MIR1324	NR_031714	TSS1500
cg01729066	9			MIR600;C9or	NR_030331;N	Body;Body
cg07236884	12			MIR614	NR_030345	TSS200
cg00328284	8			ANK1;ANK1;A	NM_020476;	Body;Body;Bc
cg01521220	17	rs77574787		MIR1203;SKA	NR_031607;N	Body;Body;Bc
cg09538129	7			MIR25;MCM7	NR_029498;N	TSS1500;Body
cg02616186	1			NAV1;MIR12	NM_0011677	Body;TSS200;
cg00944580	1			DAB1;MIR548	NM_021080;	5'UTR;TSS150
cg01983504	20			PANK2;MIR1(	NM_153638;	Body;TSS200;

cg24616828	7		MIR96	NR_029512	TSS200
cg11235787	17		MIR195	NR_029712	Body
cg26916936	11		LOC399959;N	NR_024430;N	Body;TSS200
cg01514668	11		MIR129-2	NR_029697	TSS1500
cg01965047	16		PKD1;MIR122	NM_0010099	Body;TSS150(
cg18340059	14		RTL1;MIR431	NM_0011348	1stExon;TSS2
cg19356389	6		RING1;MIR21	NM_002931;	TSS1500;TSS1
cg26180383	8		MIR1207;PVT	NR_031612;N	TSS1500;Body
cg01644741	20		MIR1259;SNC	NR_031660;N	Body;Body;Bc
cg27495572	2	rs11896779	TTN;TTN;TTN	NM_133378;	Body;Body;Bc
cg25105745	1		MIR197	NR_029583	TSS1500
cg14431528	14		MIR453;MIR4	NR_029969;N	TSS1500;TSS1
cg15212137	19		MIR519A1	NR_030218	TSS1500
cg05468584	10		MIR202	NR_030170	TSS200
cg09148270	11		BTG4;C11orf8	NM_017589;	TSS1500;TSS1
cg14148088	14		MIR494	NR_030174	TSS200
cg03964851	5	rs77830550	MIR1974;C5o	NR_031738;N	TSS200;5'UTR
cg05066959	8	rs72638959	ANK1;ANK1;A	NM_020476;	Body;Body;Bc
cg14184693	15		SERF2;MIR12	NM_0010181	Body;Body
cg01734628	6		RDBP;SKIV2L;	NM_002904;	Body;TSS150(
cg09422614	19		CACNG8;MIR	NM_031895;	Body;TSS150(
cg12974668	6		MIR1275	NR_031681	TSS1500
cg21300318	19		MIR7-3;C19o	NR_029607;N	TSS1500;Body
cg01244514	14		MIR494	NR_030174	TSS200
cg26332552	3		MIR922;KIAA	NR_030627;N	TSS1500;Body
cg14674124	10	rs78846868	MIR202	NR_030170	TSS200
cg24082174	3		SNORA63;MII	NR_002586;N	TSS1500;TSS1
cg19348206	10		MIR202	NR_030170	TSS200
cg16704590	20		C20orf199;C2	NR_003605;N	Body;Body;TS
cg09763180	10		MIR202	NR_030170	TSS200
cg11902380	12		MIR1228;LRP	NR_031597;N	TSS1500;Body
cg12029639	13		MIR548F5;NE	NR_031646;N	Body;Body;TS
cg03549146	16		MIR140;WWI	NR_029681;N	TSS200;Body;
cg06237697	17		MIR365-2	NR_029856	TSS200
cg16383389	1		MIR760	NR_030621	Body
cg27617225	8		CPSF1;MIR12	NM_013291;	Body;TSS200
cg12446939	1		ASH1L;MIR55	NM_018489;	Body;TSS150(
cg23068797	19		DNM2;MIR19	NM_004945;	Body;TSS150(
cg19273756	17		SKA2;SKA2;M	NM_182620;	Body;Body;TS

Relation_to_I	DMR	Enhancer	CONT1	CONT2	CONT3	CONT4
S_Shore		NA	0.020081987	0.016656580	0.022576118	0.020730079
Island		NA	0.015667878	0.016804944	0.018756226	0.022210022
.500;5'UTR		NA	0.027968080	0.020501608	0.023168285	0.022339365
;5'UTR;TSS200		NA	0.017252039	0.023937871	0.015456373	0.017017360
Island		NA	0.017777390	0.019823577	0.014612273	0.015350735
Island		NA	0.023586105	0.024279664	0.020895129	0.016823852
Island		NA	0.022995723	0.024557346	0.021431194	0.026311146
Island		NA	0.030334216	0.024905751	0.017763166	0.014367370
Island		NA	0.018227038	0.022591813	0.019642475	0.025313483
;5'UTR;TSS1500		NA	0.027513338	0.018275248	0.019085640	0.021327418
Island		NA	0.023096929	0.033705024	0.022071265	0.027751256
Island		NA	0.029605287	0.036820453	0.036783205	0.035420253
N_Shore		NA	0.019144480	0.022260689	0.019316880	0.024203923
		NA	0.030193293	0.035563438	0.028881646	0.026300771
Island		NA	0.026220508	0.020606083	0.022064642	0.023321147
Island		NA	0.025840935	0.020897375	0.023993847	0.023619582
Island		NA	0.030279195	0.030455016	0.029913069	0.026506560
Island		NA	0.033769518	0.031979214	0.028075365	0.036042176
Island	DMR	NA	0.035465610	0.031469238	0.022082291	0.027854577
Island		NA	0.029786126	0.021477580	0.025487568	0.024750865
Island		NA	0.043381009	0.035212510	0.040123442	0.043232631
Island		NA	0.039209792	0.037945690	0.038353308	0.026329060
Island		NA	0.033056443	0.041531331	0.041150253	0.037961859
Island		NA	0.040974484	0.025090475	0.029710346	0.024343749
Island		NA	0.023024216	0.029313263	0.029925139	0.021097640
Island		NA	0.041026054	0.032272462	0.032704599	0.027582861
S_Shore		NA	0.050790499	0.040916177	0.037359158	0.049975928
S_Shore		TRUE	0.053143890	0.059111402	0.044445962	0.043184300
Island		TRUE	0.047090486	0.040163661	0.042411288	0.045455739
N_Shore		NA	0.037960891	0.040536119	0.030763368	0.039642227
Island	DMR	NA	0.055174369	0.060636571	0.058952194	0.055690526
Island		NA	0.055087265	0.073701581	0.063607993	0.058849323
.500;5'UTR		NA	0.039851622	0.038286796	0.031896941	0.029424940
Island		NA	0.048498324	0.036689557	0.028535794	0.030693061
S_Shelf		TRUE	0.072077994	0.083767789	0.071283014	0.064422270
Island		NA	0.037950127	0.041726826	0.036080463	0.033734597
N_Shore		NA	0.037371177	0.046061557	0.032357670	0.038566987
		NA	0.904479393	0.933816348	0.913396767	0.914194002
Island		NA	0.048226686	0.040000226	0.046191921	0.048997349
		NA	0.910760169	0.925376903	0.887678314	0.886833742

S_Shore	NA		0.056544618	0.066097004	0.049764854	0.057423351
200	NA		0.897462396	0.933810578	0.899928970	0.896311338
	NA		0.891204876	0.909260607	0.889066180	0.871080187
S_Shore	NA		0.050713772	0.062283266	0.077428918	0.058269315
Island	NA		0.027366758	0.044432910	0.020810074	0.033764345
Island	NA		0.080047799	0.083905551	0.071151009	0.078138113
Island	NA		0.059895171	0.050774531	0.054557564	0.050550761
S_Shore	NA		0.913516902	0.926711589	0.875613162	0.928087516
N_Shore	NA		0.083099079	0.080915426	0.072768480	0.067383200
	NA		0.884847759	0.915981413	0.873463170	0.877548106
N_Shore	NA		0.076507769	0.073867634	0.053140238	0.054437809
Island	NA		0.044879592	0.046145224	0.041395606	0.044457896
	NA		0.884892887	0.901631069	0.866941956	0.862775750
Island	NA		0.135006862	0.129533553	0.121465604	0.117485519
	NA		0.906495777	0.899155608	0.890822844	0.872400769
	NA		0.870559134	0.892957336	0.900528071	0.885732834
N_Shore	NA		0.856560149	0.862606849	0.855895865	0.854145920
S_Shore	NA		0.068773384	0.062169880	0.058395704	0.066832338
		TRUE	0.867056902	0.892797429	0.871409626	0.856171295
0	NA		0.848014924	0.878946143	0.871910163	0.870124723
		TRUE	0.857071256	0.883742296	0.859063943	0.872331455
Island	NA		0.125325128	0.154908493	0.131028557	0.134469198
	NA		0.897562752	0.918057250	0.877568410	0.873531534
N_Shore	NA		0.830959300	0.812023246	0.761731170	0.811593228
200;TSS1500	NA		0.819048199	0.897939582	0.845041934	0.852043927
Island		TRUE	0.107105794	0.149158561	0.106979147	0.124387086
Island	NA		0.052975406	0.073751009	0.088513762	0.101176782
y	NA		0.084685600	0.128424872	0.098527654	0.117998291
N_Shore	NA		0.044680595	0.059179646	0.053458438	0.042564536
S_Shore	NA		0.037880388	0.051204669	0.046409626	0.041318161
1500	NA		0.903961979	0.919868115	0.870995067	0.834791085
S1500	NA		0.829942425	0.858483832	0.779891175	0.801656604
	NA		0.845548331	0.852939983	0.824396535	0.857071530
200	NA		0.838562392	0.837535504	0.789655413	0.817766463
S200	NA		0.150477791	0.202593119	0.128279025	0.140670413
	NA		0.812377952	0.837525741	0.791796398	0.832447923
		TRUE	0.124436115	0.139032792	0.084042415	0.152483721
S_Shore	NA		0.807004263	0.845354254	0.805736992	0.816100662
S_Shore	NA		0.780872201	0.879518480	0.818780791	0.853007660
S_Shore	NA		0.388677225	0.414830197	0.337740987	0.361484364
N_Shore	NA		0.096217527	0.117560831	0.136475424	0.133921486

		NA		0.840651712	0.853285236	0.820408833	0.847435304
			TRUE	0.780522799	0.765041336	0.771230733	0.752449614
N_Shore		NA		0.837835262	0.828113717	0.766312602	0.815762186
N_Shore		NA		0.828446685	0.802962048	0.796798646	0.838690243
);Body;3'UTR;Body;Body;Bo		NA		0.821809515	0.798528690	0.750335983	0.819784976
N_Shore		NA		0.805699650	0.823841293	0.748278699	0.798345570
S_Shelf		NA		0.705434020	0.720020018	0.665894984	0.698528357
'R	CDMR	NA		0.223703286	0.226530677	0.161150247	0.166967121
N_Shore	RDMR	NA		0.325206088	0.350210222	0.306912914	0.312926694
S_Shore		NA		0.586503673	0.649172609	0.578161103	0.572062282
N_Shelf		NA		0.841549204	0.834665121	0.799349937	0.779566295
Island		NA		0.579759563	0.583918750	0.495896441	0.497759418
		NA		0.683685060	0.686566961	0.670375823	0.658049117
!00		NA		0.404272329	0.403717216	0.381660048	0.362541450
S_Shelf		NA		0.599916270	0.662931519	0.558720929	0.624874302
)		NA		0.423320623	0.436654471	0.386402840	0.365245378
			TRUE	0.058683843	0.080164992	0.069153274	0.047010595
;00		NA		0.705541756	0.799895716	0.792065150	0.742516338
;S200;Body		NA		0.270185959	0.314069061	0.214080766	0.246232759
		NA		0.460429046	0.467097474	0.407668484	0.353112692
		NA		0.793646886	0.854205895	0.831231398	0.804493567
Island		NA		0.562002160	0.592288408	0.515801895	0.517667830
N_Shore		NA		0.316824966	0.335811537	0.294533699	0.309572869
		NA		0.542854950	0.524974248	0.486414315	0.503033873
		NA		0.804157720	0.822024718	0.808187944	0.805755561
N_Shore		NA		0.307665681	0.331581594	0.345643387	0.393168761
N_Shore		NA		0.315545459	0.337638594	0.294684315	0.316978140
/		NA		0.718549031	0.750298275	0.619773962	0.671629637
S_Shore		NA		0.670318751	0.717375039	0.625049478	0.613789030
		NA		0.709764868	0.831022022	0.776885330	0.795513930
ody;5'UTR			TRUE	0.737716799	0.755371074	0.690173396	0.709373090
S_Shore		NA		0.407552298	0.425764172	0.388515507	0.393047241
		NA		0.746523206	0.736383938	0.753674737	0.710023803
		NA		0.241062689	0.223738924	0.167570435	0.151673949
			TRUE	0.544521464	0.662860846	0.539848708	0.576831525
ody;Body;Body;Body;Body;T		NA		0.727547794	0.699015182	0.719229954	0.748961833
ody		NA		0.507219889	0.466953652	0.451689492	0.374715722
/;Body;TSS200;TSS1500		NA		0.439401774	0.467902672	0.371820906	0.455286292
Body		NA		0.768636202	0.776088773	0.683879713	0.698336932
)0		NA		0.646964121	0.742805582	0.755722449	0.731370591
Body;Body			TRUE	0.468246186	0.528771328	0.411593326	0.429221964

N_Shelf		NA		0.619169013	0.620317940	0.549157067	0.529257196
S_Shelf		NA		0.492285170	0.507969031	0.414857177	0.425628433
			TRUE	0.066528889	0.083200621	0.083587578	0.067433294
N_Shore	CDMR		TRUE	0.077790590	0.082967596	0.089736641	0.085770397
Island		NA		0.432798289	0.474855627	0.364367324	0.459256567
N_Shore		NA		0.553118622	0.583111489	0.500861364	0.491020910
N_Shore		NA		0.167160914	0.201724093	0.150945658	0.157404552
y			TRUE	0.757838717	0.731057063	0.765757220	0.748180995
S_Shore	RDMR	NA		0.389846394	0.378102448	0.354883991	0.322067676
ody;Body;Body		NA		0.773203223	0.808425166	0.759249624	0.720330825
		NA		0.501693370	0.586360385	0.553165693	0.566107914
.500;TSS200			TRUE	0.738873058	0.796005660	0.682992781	0.688695389
		NA		0.601300501	0.666259253	0.604380235	0.570652209
		NA		0.631426479	0.635955515	0.601103005	0.562201072
N_Shore		NA		0.323546926	0.356354924	0.277538368	0.268608594
		NA		0.777354544	0.742230392	0.722039610	0.717967518
;5'UTR		NA		0.657099514	0.707457614	0.568261354	0.682545397
ody;Body;Body;Body;Body;T		NA		0.770869801	0.844389543	0.910892516	0.881622576
S_Shore		NA		0.497735421	0.515140873	0.510079027	0.463730553
N_Shore		NA		0.684633985	0.703934715	0.633836629	0.619246823
N_Shore		NA		0.524682174	0.638409569	0.666151954	0.660447816
		NA		0.478429778	0.563571450	0.442007598	0.537420680
y		NA		0.218814417	0.319168082	0.217510540	0.291601275
		NA		0.566487481	0.635372509	0.552858509	0.609753666
y;Body		NA		0.716635960	0.705489162	0.748209696	0.747326439
		NA		0.763241562	0.804200037	0.776685986	0.754931399
S_Shore	RDMR	NA		0.369974858	0.442613072	0.321275661	0.375764846
		NA		0.497669583	0.554195709	0.439563498	0.403313077
S_Shore	RDMR	NA		0.458437315	0.518768404	0.470684041	0.446593738
		NA		0.679864211	0.720727591	0.656116392	0.665915883
y		NA		0.453868597	0.297615035	0.377244628	0.414432330
N_Shore	RDMR	NA		0.337832473	0.423247068	0.370383435	0.263324512
Body		NA		0.979105800	0.982017122	0.974426764	0.969636836
		NA		0.952134545	0.964630721	0.950773834	0.947843617
Island		NA		0.053956770	0.034766948	0.048659913	0.055113802
S_Shelf		NA		0.969437737	0.967997943	0.967482456	0.958119848
)		NA		0.948150686	0.946198505	0.925469515	0.949014531
);Body;Body;Body		NA		0.795467768	0.847906031	0.813162535	0.860331360
N_Shelf		NA		0.801179179	0.852797530	0.829629712	0.753283555

CONT5	CONT6	CONT7	CONT8	CONT9	DA1	DA2
0.023844927	0.025334590	0.024586415	0.025837752	0.023250725	0.023433390	0.026012643
0.016830285	0.021474076	0.022948674	0.021792394	0.020785708	0.023784845	0.021479513
0.018997640	0.025434018	0.021115870	0.019267683	0.022785497	0.028062786	0.022688815
0.019992166	0.022741515	0.017650369	0.021138137	0.017638498	0.019471047	0.017446755
0.016607144	0.019548987	0.018889149	0.016812559	0.016466229	0.018825542	0.028910550
0.023892128	0.027172182	0.022029522	0.025919123	0.025360447	0.024346309	0.024561492
0.019244574	0.025583466	0.027578937	0.030796691	0.032106200	0.030648630	0.032671214
0.015251647	0.026790851	0.017180364	0.022416535	0.023910202	0.025485287	0.028286581
0.024653621	0.029677052	0.023770710	0.026925058	0.031804188	0.024055207	0.028834717
0.020050709	0.023801563	0.020532931	0.021047090	0.023553877	0.030447722	0.028681542
0.024880364	0.024219737	0.023397828	0.020940725	0.026674919	0.029269255	0.032362350
0.032867431	0.044941548	0.041891937	0.039496712	0.041819297	0.042730221	0.038841785
0.018253191	0.024605306	0.025288430	0.024729754	0.021869456	0.029515087	0.035493348
0.024655584	0.038313797	0.031343343	0.035875438	0.027698770	0.028014984	0.036709174
0.019182358	0.028419651	0.023610400	0.036048486	0.018901803	0.019225630	0.025952657
0.028369031	0.041527825	0.026012288	0.033341822	0.032812956	0.021633155	0.046965381
0.027946860	0.029343320	0.031611738	0.029467013	0.032311717	0.030890563	0.033219426
0.027777848	0.037569153	0.024426143	0.031665208	0.039077059	0.033470549	0.042851325
0.032004992	0.045709593	0.030949009	0.046904734	0.040525850	0.038390967	0.036991389
0.026170617	0.028693265	0.031823348	0.026057400	0.030603632	0.043535515	0.039079528
0.035584526	0.039738152	0.047886752	0.034345668	0.047650356	0.043041521	0.042425462
0.024633963	0.044413490	0.034557534	0.047204789	0.036428171	0.037374871	0.042134609
0.035991315	0.045148422	0.050443723	0.052848165	0.040512807	0.047711099	0.050904287
0.024107696	0.035985086	0.026447917	0.031356585	0.027232291	0.027689091	0.045053912
0.029536889	0.028093019	0.021730852	0.022407737	0.032129696	0.033238237	0.038027808
0.035143328	0.042941569	0.038505660	0.043281261	0.042310508	0.046583881	0.045298367
0.041079523	0.048120508	0.054498851	0.046643400	0.052412910	0.044285614	0.068624178
0.047900717	0.057482333	0.050873922	0.058063651	0.056156720	0.061791821	0.067966335
0.046863764	0.040649271	0.047866598	0.046475106	0.052819374	0.056697304	0.053772836
0.026319439	0.032132690	0.049519360	0.037002100	0.037569471	0.041080960	0.037893309
0.063792395	0.074168461	0.062282399	0.068107730	0.065207540	0.062064066	0.066273546
0.069347049	0.061390554	0.058701366	0.063892466	0.071553471	0.082072601	0.070742871
0.032383500	0.036342912	0.026746577	0.035492236	0.034199072	0.043038631	0.045858124
0.035454989	0.047616540	0.037964893	0.053533004	0.038702756	0.029892020	0.042300009
0.063326458	0.066922174	0.069072881	0.078067984	0.086644724	0.094126763	0.092351226
0.025608876	0.041812364	0.033963206	0.050828929	0.035961970	0.034541929	0.042507384
0.026537115	0.082712582	0.046710866	0.051224548	0.037916592	0.043353615	0.042523947
0.881950281	0.894215966	0.912974077	0.929380736	0.914988115	0.915118933	0.920440597
0.045242048	0.057463823	0.051288645	0.055012145	0.060466724	0.047513717	0.070539764
0.888103400	0.876580530	0.895438493	0.926349456	0.906800385	0.910514977	0.913713952

0.053007374	0.071996956	0.065656424	0.064183890	0.054585026	0.072347975	0.062350822
0.909206880	0.894999930	0.892435149	0.903248463	0.914663186	0.918576522	0.911185123
0.887230182	0.874033867	0.885631384	0.886131901	0.886794150	0.885098086	0.918577422
0.053091725	0.082453475	0.061594992	0.057351560	0.057027743	0.057587577	0.075642463
0.025098828	0.054009175	0.048371113	0.029268805	0.023679727	0.036732976	0.042039046
0.061658678	0.084061122	0.079255866	0.089613314	0.074478133	0.089835557	0.097381115
0.074591729	0.065116966	0.071033622	0.083130668	0.075118509	0.053902666	0.073191898
0.885521946	0.909181573	0.897698577	0.935399197	0.920413611	0.936559567	0.930577359
0.032137618	0.066136207	0.078745943	0.072409191	0.083386174	0.098976530	0.097093235
0.892347964	0.868110155	0.884166285	0.896646419	0.919750985	0.900869475	0.919385411
0.058570617	0.073650508	0.076729788	0.081392619	0.063825157	0.062849099	0.086690601
0.046245515	0.058884337	0.059393552	0.068988232	0.061959383	0.056389468	0.094662207
0.853034462	0.879067557	0.866581101	0.897682509	0.914206137	0.908441055	0.898805317
0.122618056	0.121319995	0.127057269	0.131701389	0.152014371	0.129375207	0.141140162
0.899930617	0.870045367	0.895043002	0.878605103	0.903058315	0.903095352	0.912339355
0.887524488	0.855574981	0.869495113	0.905268471	0.906434466	0.926835598	0.892779146
0.873646410	0.845201343	0.835559688	0.862295293	0.867264726	0.880228390	0.871765870
0.043929448	0.083291261	0.068828705	0.066388479	0.068277471	0.068579869	0.077386134
0.859506110	0.862611903	0.864488376	0.899440945	0.883263287	0.890231091	0.898730503
0.852836869	0.820547489	0.857563495	0.866991544	0.859991010	0.866960018	0.894736857
0.878181845	0.822467076	0.863046180	0.834129252	0.856159058	0.890621177	0.875227279
0.134017692	0.161479817	0.179104112	0.148595011	0.194573228	0.151813685	0.149560021
0.883813613	0.860742694	0.880413577	0.883492006	0.888792604	0.907392030	0.912687747
0.789618231	0.835166757	0.784963616	0.783578383	0.802966568	0.827321078	0.805077673
0.857989454	0.861602393	0.867709441	0.895987004	0.838900258	0.889794642	0.873570151
0.128547661	0.143897945	0.140941133	0.114078456	0.145621345	0.128912827	0.148857610
0.088403949	0.120195861	0.088243932	0.078949216	0.046357875	0.104238256	0.126210355
0.098678653	0.159224947	0.103635636	0.150779925	0.129062809	0.107992368	0.156848113
0.033486000	0.068942301	0.042906055	0.056132445	0.043919400	0.048830911	0.056775441
0.029938492	0.068943331	0.042352538	0.061508694	0.055652713	0.043807722	0.053104967
0.816908900	0.828120778	0.867012209	0.904162562	0.861182801	0.925000176	0.899807737
0.835763743	0.772814643	0.797577251	0.816176049	0.837318437	0.847669294	0.835493929
0.853797858	0.841543928	0.849889332	0.888981262	0.887909917	0.893743295	0.874377929
0.856641008	0.795585682	0.823720556	0.839956387	0.855033548	0.856747579	0.865365659
0.095758563	0.107370413	0.153799157	0.173659527	0.155246015	0.151688367	0.189438838
0.863214756	0.859908587	0.847253662	0.866417233	0.836306982	0.817538389	0.874414851
0.208384635	0.138108991	0.148155870	0.130157680	0.145470166	0.139377773	0.181271857
0.830050392	0.771317583	0.791506031	0.838829021	0.842846853	0.819696458	0.845539736
0.820837909	0.834709687	0.829861548	0.859423570	0.821170829	0.852979713	0.848213303
0.384080427	0.372500851	0.360700004	0.380531356	0.378670304	0.386759474	0.394379954
0.123713938	0.139500325	0.113453787	0.141841504	0.131220697	0.142081037	0.119881482

0.824029176	0.798973977	0.796538954	0.810400541	0.862832786	0.908082817	0.859705357
0.804434982	0.693024476	0.748811501	0.760472099	0.779501043	0.794258697	0.790282257
0.830379404	0.787710653	0.828848055	0.856736204	0.824330340	0.836982800	0.845662090
0.873817217	0.817064467	0.824113697	0.815271631	0.821289539	0.847557016	0.887363492
0.830538620	0.780245063	0.807439487	0.822959486	0.836528606	0.783460730	0.847930242
0.785949998	0.771644452	0.778604739	0.817814514	0.828605746	0.817048364	0.823232676
0.734036070	0.703023802	0.693236240	0.722762707	0.720129575	0.752062462	0.711338420
0.199296555	0.205403716	0.198607979	0.202811802	0.179910060	0.187899066	0.235206456
0.295286014	0.343185625	0.306757092	0.353945662	0.355475346	0.350530481	0.331915339
0.621131106	0.577166283	0.595292897	0.590553852	0.634475993	0.629892914	0.609233878
0.794865223	0.736289393	0.823856556	0.759831754	0.809425391	0.825575723	0.837800945
0.535610452	0.561827780	0.590171294	0.601098735	0.596133006	0.623989237	0.598072148
0.663674055	0.646527508	0.641158982	0.687423320	0.713439857	0.690931120	0.706840795
0.377745353	0.440639671	0.387124757	0.369313875	0.433891436	0.402822188	0.377291957
0.612108160	0.586238097	0.614946863	0.653742548	0.707357646	0.658966428	0.649600626
0.386248598	0.406869613	0.400181606	0.408825698	0.424205337	0.406782267	0.400401041
0.044318973	0.110956978	0.067414796	0.090340680	0.050032931	0.063736016	0.120660114
0.783107651	0.756859229	0.799923438	0.824446470	0.801166286	0.856005729	0.781508496
0.249199872	0.217452560	0.236251978	0.266551675	0.259657888	0.277180866	0.263403480
0.465704183	0.342768552	0.407209905	0.411787263	0.445114685	0.463233821	0.417547790
0.826989351	0.858473965	0.840757390	0.833933618	0.790309117	0.866898656	0.853878554
0.495212858	0.495876460	0.505082904	0.550904514	0.560253978	0.576765759	0.571129684
0.308164812	0.307984725	0.308415632	0.303258133	0.305227038	0.313131200	0.347946437
0.523305995	0.491154980	0.512573720	0.566876462	0.544437780	0.594816263	0.530524571
0.870557819	0.852320505	0.785183161	0.842585645	0.802534246	0.850688300	0.869265384
0.280579297	0.328295948	0.376692363	0.335100977	0.318571423	0.315960197	0.465170812
0.293063046	0.307406468	0.311252420	0.312138253	0.345774399	0.354113740	0.335282846
0.691817087	0.650407707	0.635208660	0.733995306	0.697336110	0.732556444	0.721523073
0.695288131	0.631256106	0.707172137	0.655934205	0.704091148	0.712564847	0.748203477
0.799914318	0.765162774	0.710899747	0.795111543	0.745304706	0.787211084	0.801298163
0.708185962	0.739438468	0.732375462	0.778018055	0.784492670	0.767899840	0.774894989
0.376116295	0.358145433	0.385109075	0.491775795	0.457212045	0.455409260	0.400705299
0.736686292	0.687182916	0.724177990	0.708824630	0.805444070	0.748108450	0.734341687
0.133401509	0.129569450	0.192745898	0.203073817	0.189658567	0.207199624	0.244456392
0.583240144	0.553950460	0.609097449	0.609013335	0.627393449	0.614677060	0.605083832
0.753087233	0.725513921	0.731571862	0.734637232	0.770800045	0.739159356	0.759605865
0.370502820	0.358194380	0.390815121	0.427751031	0.465882616	0.521562709	0.442683900
0.500233280	0.449890717	0.409130780	0.430915968	0.421725551	0.456266910	0.471322605
0.708689722	0.695200342	0.707103289	0.785230474	0.716747238	0.773078438	0.756607350
0.775981211	0.815161654	0.752220941	0.699141539	0.789537904	0.785643742	0.769999087
0.552455084	0.434326980	0.498871194	0.469241103	0.471608331	0.471416205	0.528816157

0.674483593	0.532407308	0.557239207	0.550718464	0.576087103	0.595880984	0.678975996
0.451572528	0.455059755	0.471653793	0.439388621	0.474244690	0.488978515	0.559916780
0.046114886	0.149111290	0.091180670	0.098959869	0.071695378	0.075277442	0.121233670
0.063856584	0.137576148	0.082806656	0.151336228	0.097750225	0.074525934	0.117504898
0.478300915	0.460973609	0.426237767	0.436583993	0.416341492	0.451184111	0.482145813
0.537974189	0.484234553	0.490535814	0.498214813	0.534672144	0.543745515	0.603348251
0.122809505	0.170123806	0.163953336	0.202328973	0.174834404	0.181934557	0.210517271
0.822594584	0.753971338	0.772018842	0.735410192	0.795457033	0.815046034	0.785898563
0.319944276	0.296782676	0.321518060	0.405665700	0.358652723	0.358840458	0.413003726
0.734673252	0.796721224	0.770913112	0.856589465	0.802723188	0.788595240	0.832036001
0.492301693	0.502818159	0.482562013	0.588112098	0.600788737	0.618672842	0.555446589
0.768264255	0.730058658	0.737091176	0.681868711	0.749488313	0.756059853	0.838264231
0.529210321	0.654116174	0.625728672	0.645274292	0.683886549	0.601969317	0.607060503
0.546544836	0.506143818	0.439413035	0.608854373	0.613870018	0.630588249	0.618915812
0.297781433	0.303237723	0.331266447	0.353839153	0.327414080	0.312379890	0.380961676
0.778059179	0.806578822	0.707235956	0.842235585	0.744032732	0.821518260	0.847760917
0.795684262	0.684747092	0.678226710	0.633559070	0.652395471	0.772438360	0.784202785
0.930200365	0.890537982	0.914440223	0.694615925	0.896434738	0.945470616	0.929150141
0.492161267	0.508843448	0.478414260	0.511610088	0.529920847	0.509863161	0.500485123
0.641105980	0.692574284	0.659252644	0.705649963	0.676209115	0.676155298	0.692648032
0.655444383	0.643876551	0.658194422	0.613659565	0.724233904	0.621386532	0.700272359
0.485561642	0.492871638	0.539292769	0.574615184	0.636829551	0.547157259	0.555014495
0.288094342	0.328902425	0.297265101	0.313571191	0.250116010	0.201463297	0.366940649
0.673517516	0.673855493	0.586920584	0.592082277	0.604428852	0.641599828	0.689155892
0.821971995	0.788309172	0.760915746	0.715444036	0.795363643	0.765393668	0.818121597
0.813133682	0.693345357	0.582105090	0.745053320	0.752816195	0.798572011	0.768050994
0.438878769	0.378369432	0.307861990	0.479438150	0.368828086	0.335077280	0.533285384
0.385791462	0.372708115	0.377250955	0.472622177	0.500947596	0.556184502	0.444647435
0.542052213	0.571732309	0.512517776	0.535147897	0.550231136	0.518883225	0.671448142
0.645775350	0.562981426	0.474393541	0.659094088	0.701047345	0.720463775	0.682918214
0.541306875	0.239843392	0.411584811	0.457466995	0.440403476	0.417674517	0.499658227
0.374736263	0.273106737	0.350971655	0.324564475	0.365907699	0.413394868	0.391942513
0.980388962	0.963121517	0.975514939	0.974706019	0.974587851	0.976914319	0.965958048
0.961044362	0.941991311	0.949960333	0.944598201	0.953136955	0.943509195	0.952570998
0.043528723	0.037082531	0.032470884	0.031421371	0.025969677	0.032557166	0.034871711
0.961169808	0.955680246	0.965250535	0.956774447	0.968283290	0.967209163	0.936891048
0.959121648	0.889433977	0.926141545	0.903226291	0.935750321	0.947810996	0.929787224
0.926851808	0.873541112	0.804321213	0.803247652	0.813564898	0.838959906	0.694326976
0.660064219	0.662444865	0.716598860	0.741711120	0.783702185	0.709938757	0.680094536

DA3	DA4	DA5	DA6	DA7	DA8	DA9
0.032721204	0.023170242	0.025016019	0.026659339	0.026400674	0.025288646	0.029205357
0.024859824	0.029959423	0.019109808	0.026793531	0.019535416	0.022659096	0.027179256
0.030850476	0.031687567	0.018229861	0.026357624	0.023179811	0.033002751	0.028934922
0.030975450	0.024125942	0.020992963	0.022402812	0.024523293	0.029456727	0.025758738
0.024116715	0.028766974	0.019013871	0.021990307	0.017457640	0.019886793	0.022540231
0.024558625	0.027567997	0.022930363	0.030413727	0.029536014	0.028065472	0.040887822
0.033671078	0.027814271	0.029472362	0.032862431	0.030702938	0.032152931	0.033316663
0.024098296	0.033891294	0.024745837	0.033286080	0.024828849	0.024541467	0.024753340
0.034317088	0.043050791	0.029455136	0.025281331	0.025931063	0.035563211	0.033603924
0.026813103	0.032432151	0.025196872	0.026602777	0.019945674	0.031866246	0.032979304
0.033620013	0.031385342	0.019817599	0.037307488	0.032002894	0.030981602	0.028110044
0.048190627	0.051963768	0.034571817	0.041324920	0.047727838	0.051608150	0.038025924
0.030246590	0.035400315	0.017752445	0.026173590	0.027250371	0.026763629	0.028069152
0.045989948	0.036036969	0.032065644	0.035251944	0.037531808	0.041838588	0.036022523
0.041069724	0.040559758	0.027928020	0.036565939	0.026860788	0.031003777	0.029243994
0.042122872	0.035716792	0.032613405	0.026764484	0.031619848	0.048297395	0.031908097
0.042054229	0.036319167	0.027177409	0.047110208	0.030530792	0.044602714	0.046037269
0.057478067	0.035538837	0.029462820	0.050351567	0.039333258	0.044451770	0.038242865
0.040008684	0.048388441	0.026408499	0.046425755	0.038738631	0.056900596	0.051435250
0.039814934	0.046306502	0.026404120	0.034527689	0.031060093	0.031513166	0.034318554
0.052444139	0.051968942	0.046587730	0.059831109	0.046670015	0.052025101	0.058593919
0.058938342	0.045066623	0.038486314	0.039483478	0.029156262	0.046884072	0.066378611
0.049186103	0.055688303	0.038633512	0.046363037	0.046391859	0.049417432	0.066988077
0.047051796	0.041379857	0.040506324	0.038319282	0.035319491	0.041420303	0.025244717
0.046258418	0.044854298	0.037741052	0.022413197	0.031557746	0.040038868	0.028863331
0.051894223	0.062692046	0.045943980	0.036061149	0.032116898	0.043162679	0.049851251
0.072618862	0.070456671	0.047813465	0.047542374	0.043319406	0.059532409	0.055552128
0.068235789	0.059395762	0.050438723	0.055103685	0.069400183	0.075951599	0.055037593
0.059083956	0.075635716	0.051692361	0.056325649	0.046658535	0.047058527	0.055588629
0.055727724	0.057079702	0.031911774	0.047249149	0.044172030	0.053305891	0.065881363
0.078063631	0.085647123	0.054144080	0.078125643	0.070216210	0.075565724	0.089711770
0.081471644	0.066825756	0.084069993	0.072954946	0.068212880	0.061636073	0.075810276
0.063803524	0.047590421	0.035749430	0.041904539	0.035463911	0.046377260	0.050442298
0.048893280	0.066115618	0.046683305	0.066520708	0.041367256	0.058230505	0.057101906
0.080705909	0.088412269	0.094764274	0.076172844	0.069179227	0.080738857	0.084153045
0.059529565	0.069661161	0.053074945	0.043356468	0.039287749	0.039988304	0.073268427
0.097372119	0.063694050	0.040530713	0.061661715	0.045195772	0.059435925	0.066253473
0.937921310	0.921142388	0.911693769	0.915724492	0.925081687	0.933938607	0.936661129
0.081262900	0.078242447	0.082341081	0.050055293	0.053910735	0.059544336	0.059730146
0.917438643	0.890721532	0.912812496	0.918336081	0.906106706	0.928548384	0.934281532

0.071783355	0.102119772	0.064120856	0.057983574	0.052556099	0.073832244	0.069620020
0.926307532	0.878913348	0.910975160	0.923792629	0.935319024	0.932386996	0.941887389
0.905867527	0.875859623	0.873511892	0.901141629	0.899351125	0.921934516	0.932671135
0.068606106	0.071635456	0.068716297	0.122936141	0.090957820	0.064393938	0.099250449
0.066970519	0.079227551	0.020274041	0.061610766	0.037704596	0.055219780	0.070187072
0.099941678	0.114155662	0.081290146	0.079962078	0.084817904	0.097213760	0.102669203
0.094845364	0.090982194	0.073084654	0.078187944	0.073621811	0.077810109	0.095622298
0.945931784	0.921698928	0.893402246	0.931375956	0.907304480	0.929960959	0.936153899
0.103986700	0.114789373	0.083086909	0.074071320	0.079372864	0.075765814	0.070044440
0.902426399	0.877824735	0.901608720	0.906337023	0.911924111	0.915148801	0.926859081
0.090096149	0.070930440	0.081436701	0.086134299	0.066222330	0.104797124	0.140312114
0.073761751	0.085643870	0.063645547	0.063447623	0.054458471	0.067224447	0.088000877
0.891318473	0.887043474	0.889888740	0.872071117	0.900350469	0.918065720	0.913489758
0.139165504	0.171963438	0.139599097	0.146182691	0.133940032	0.151640379	0.159487511
0.929491451	0.895639750	0.885747576	0.918172662	0.908465255	0.903826206	0.917520806
0.918662684	0.896212253	0.872620472	0.901725204	0.899405088	0.906601201	0.918117239
0.880050707	0.855761478	0.855055577	0.879760164	0.871726512	0.890422765	0.901006645
0.094636193	0.098854518	0.078690785	0.092578809	0.074498566	0.096309655	0.087373316
0.898805127	0.871138190	0.841167255	0.911688701	0.873116915	0.916613444	0.928026832
0.861074310	0.872217216	0.864400575	0.887961195	0.850824995	0.890233804	0.914085254
0.885514754	0.860204558	0.838552586	0.883803600	0.884638935	0.904431827	0.896851389
0.191290525	0.188222443	0.169877094	0.162543106	0.155039827	0.197045011	0.215552633
0.919776409	0.877257778	0.894312960	0.903948437	0.894574591	0.914743254	0.930379214
0.844439503	0.829625875	0.826550633	0.812823216	0.813629541	0.815261984	0.814945736
0.899297179	0.849587061	0.872914015	0.879891546	0.880018674	0.864668465	0.908614541
0.138299244	0.187759928	0.132726833	0.146968903	0.125293695	0.151791014	0.184802806
0.111211717	0.103329323	0.073843271	0.155448940	0.078812482	0.076199965	0.124454798
0.142353560	0.164998017	0.120841129	0.159000450	0.144241613	0.130485976	0.167661753
0.133530837	0.051701543	0.042289792	0.106307113	0.062913926	0.087891540	0.114686577
0.169255658	0.064685078	0.038857315	0.102003942	0.060310254	0.068743739	0.084265285
0.914033365	0.868429395	0.905653516	0.872922393	0.898739773	0.868454377	0.903118484
0.851830735	0.836243732	0.798819358	0.864399389	0.814562239	0.843405449	0.871207339
0.925705591	0.839438708	0.828815413	0.888229438	0.869307113	0.936674395	0.903425483
0.909636607	0.819339625	0.826995889	0.854849826	0.817024306	0.852994662	0.908062945
0.116673298	0.191703022	0.204776123	0.195865664	0.161808595	0.143944975	0.203583071
0.910663432	0.817127502	0.840328987	0.875784164	0.873536373	0.898606313	0.928440370
0.181020481	0.159148405	0.132981958	0.144920515	0.149128335	0.218676499	0.229314377
0.850684700	0.836998065	0.808970510	0.867588178	0.822489937	0.870980152	0.870548792
0.917379155	0.857853926	0.837943804	0.869255504	0.845359113	0.860718330	0.892760634
0.455787662	0.408836177	0.375581444	0.396528978	0.365873672	0.434803526	0.427988510
0.140006408	0.163833935	0.194076836	0.149700850	0.180314500	0.190984829	0.136887485

0.910095378	0.827504401	0.826938795	0.829633066	0.836037683	0.845834569	0.865457990
0.802037250	0.789406818	0.756131273	0.761344541	0.763257252	0.838378192	0.836702918
0.841884066	0.841270734	0.850935928	0.845007459	0.851892664	0.827456541	0.885154836
0.863604100	0.846776136	0.822047402	0.826357430	0.843527218	0.859517149	0.883110589
0.860446835	0.776274031	0.788327927	0.872144267	0.864019723	0.874715531	0.904227994
0.801834620	0.867451347	0.796110931	0.821999174	0.824253481	0.870392051	0.848958572
0.790136515	0.705675072	0.709214655	0.732187843	0.734715526	0.746166606	0.771058403
0.207243473	0.292449219	0.242226230	0.231295958	0.207181466	0.224911049	0.240196356
0.347738595	0.375407564	0.332315981	0.373032092	0.312563203	0.350028913	0.426227700
0.665111296	0.637397377	0.559468031	0.664728952	0.596975204	0.686041801	0.665713566
0.843022543	0.824476156	0.813924497	0.826984628	0.799155758	0.835110909	0.905362985
0.593023618	0.615525866	0.593900896	0.568424024	0.547678847	0.573356778	0.620282204
0.738096222	0.668411404	0.655616852	0.783597001	0.701303465	0.690441309	0.750107189
0.414172823	0.446885745	0.426677848	0.391857327	0.421491507	0.469256600	0.495442976
0.677840435	0.631767894	0.630841847	0.623106057	0.666522431	0.681361604	0.707460664
0.467126523	0.456711429	0.402867039	0.460032866	0.416996494	0.451516101	0.498151873
0.231676781	0.075964595	0.080519766	0.100101074	0.082502889	0.126017082	0.112227701
0.889129250	0.810244267	0.763476169	0.846455123	0.831100345	0.743263164	0.836263220
0.288636161	0.301058671	0.297223250	0.290980001	0.277672558	0.306744161	0.312959609
0.468263573	0.472695418	0.420097356	0.492407509	0.442265145	0.496762771	0.462873761
0.868425967	0.838092921	0.794715384	0.868332983	0.867488260	0.891276177	0.914403600
0.584824798	0.591284870	0.551013300	0.573633595	0.526901897	0.545582348	0.607500526
0.320547329	0.387378410	0.334817626	0.375843057	0.330298396	0.341380901	0.405264851
0.582375350	0.555907219	0.537048110	0.581143787	0.526693265	0.588828267	0.559357071
0.910662296	0.774810111	0.834422396	0.885197513	0.856593152	0.884914387	0.905319214
0.332520578	0.358182858	0.392094078	0.388696245	0.322544133	0.403347429	0.436695534
0.345759241	0.384666673	0.365940435	0.334633267	0.325622042	0.341320298	0.407834084
0.712041907	0.657247947	0.669149196	0.790216269	0.703545984	0.735336398	0.776616092
0.686003543	0.634061761	0.624695562	0.743693840	0.691999394	0.778986907	0.739533167
0.870986405	0.809212549	0.781980236	0.802665841	0.805058842	0.815674793	0.854732041
0.816965566	0.765739622	0.722724215	0.783100496	0.787624728	0.790345148	0.814096770
0.420107488	0.461285983	0.368995210	0.497930724	0.469724639	0.484120242	0.502460916
0.776171379	0.837484951	0.751104940	0.800130405	0.749637021	0.801656510	0.772421350
0.166305598	0.231892017	0.236091403	0.281519511	0.189855430	0.222089862	0.240697385
0.617025366	0.651347970	0.615429637	0.605952322	0.634533890	0.651086885	0.682406096
0.803741313	0.765063669	0.728902514	0.820585679	0.760433315	0.797062068	0.857112706
0.400965708	0.464027630	0.455849756	0.547546988	0.471314804	0.421055851	0.488607440
0.483039841	0.462575189	0.466793541	0.467488105	0.443888944	0.503856812	0.572052508
0.765553598	0.731447105	0.732181498	0.845318596	0.754824294	0.792096819	0.798742069
0.873355763	0.717887395	0.755806665	0.814438279	0.820787045	0.799741312	0.830088735
0.616250451	0.487667044	0.425278131	0.536583756	0.528645607	0.597227409	0.530714715

0.655268118	0.628557632	0.543305906	0.651881002	0.603635780	0.652157856	0.648807671
0.446131010	0.493130968	0.420143331	0.537171096	0.483553989	0.567915287	0.572105683
0.339488338	0.084398173	0.068704549	0.127065187	0.126965040	0.126987692	0.137661944
0.277860956	0.092294325	0.072935979	0.181613553	0.129140109	0.154874656	0.224304531
0.533556306	0.443635358	0.424017769	0.497695002	0.403862974	0.529914628	0.558152265
0.545078424	0.544601614	0.560696049	0.602042781	0.522404118	0.613175264	0.588630379
0.198095745	0.220917967	0.249724711	0.270100849	0.175105830	0.192663005	0.308074416
0.862243786	0.783898520	0.753224464	0.876879458	0.839004978	0.827272172	0.854615308
0.439809140	0.377691134	0.397772844	0.429086943	0.440254190	0.395119768	0.420780323
0.887331466	0.818111825	0.804215059	0.828442806	0.794708655	0.848311494	0.861622384
0.529442474	0.537049087	0.534844868	0.664090477	0.626671818	0.585794959	0.679896856
0.841485020	0.761110070	0.762835980	0.775031124	0.755583564	0.773772811	0.790598361
0.679190542	0.660832930	0.629755916	0.713897453	0.682880017	0.750550460	0.711093120
0.585918509	0.628730089	0.578421287	0.638500774	0.595420488	0.666727291	0.659827370
0.284185498	0.349772643	0.333356212	0.463139307	0.431018324	0.436854596	0.367142242
0.826471925	0.703792223	0.710715694	0.860632825	0.779222094	0.905588206	0.880079133
0.774468715	0.715850877	0.595846781	0.682846616	0.681909884	0.780962618	0.800147901
0.935125310	0.859520577	0.945924829	0.951883705	0.940577213	0.919992350	0.927988088
0.555337514	0.546240088	0.500336854	0.609547785	0.564116384	0.598580728	0.627314990
0.764145700	0.696909988	0.622110247	0.820975182	0.730594060	0.738795409	0.785215646
0.731002336	0.590907424	0.643181654	0.732245900	0.770139058	0.708771444	0.793887908
0.567658342	0.547540698	0.607731034	0.573401224	0.592226444	0.634078227	0.639253827
0.393067345	0.391234650	0.293499224	0.389278670	0.355754406	0.384429601	0.356935958
0.752892088	0.575915990	0.625318218	0.669718398	0.621530900	0.766867535	0.766890057
0.904914052	0.719962907	0.744706110	0.899096368	0.835923391	0.877945480	0.898062742
0.795043136	0.855941832	0.764498188	0.865180409	0.792052761	0.860663923	0.837459508
0.531461850	0.418780558	0.365838546	0.467836584	0.437746435	0.504880819	0.504745907
0.445451463	0.603651477	0.520005828	0.508473456	0.493174743	0.495963642	0.572493144
0.736163314	0.518817836	0.475987424	0.565474997	0.591608451	0.601928630	0.642691842
0.661948267	0.771334842	0.716989575	0.725499717	0.698568923	0.712892586	0.768021326
0.496472448	0.462599323	0.411532703	0.498745462	0.470789995	0.591892830	0.537851625
0.455479794	0.570126312	0.410873178	0.501830705	0.384857785	0.580857279	0.340273220
0.970079201	0.961402938	0.962747086	0.965032243	0.971858252	0.974325767	0.966609414
0.938983711	0.944320263	0.934369032	0.925326366	0.937224572	0.952867232	0.944357027
0.027235115	0.038002954	0.038549070	0.026963313	0.024513568	0.027603383	0.028698189
0.955899377	0.945541177	0.934626155	0.966411940	0.964653804	0.958505460	0.946504539
0.819037221	0.921408089	0.930453353	0.819289667	0.904157348	0.901877415	0.886891547
0.869662103	0.741736779	0.768838332	0.677469258	0.824428682	0.786408504	0.831143805
0.579380554	0.727045421	0.808842161	0.585065582	0.763550756	0.535554458	0.610571390

DA10	mean.CONT	mean.DA	mean.diff	mean.quot.lo	max.CONT	min.CONT
0.030465860	0.022544353	0.026837337	-0.00429298	-0.17876157	0.025837752	0.016656580
0.026522290	0.019696690	0.024188300	-0.00449161	-0.203200561	0.022948674	0.015667878
0.028097750	0.022397561	0.027109236	-0.00471167	-0.19589310	0.027968080	0.018997640
0.025014934	0.019202703	0.024016866	-0.00481416	-0.22014832	0.023937871	0.015456373
0.020956436	0.017320894	0.022246506	-0.00492561	-0.23913816	0.019823577	0.014612273
0.031819291	0.023328684	0.028468711	-0.00514002	-0.206921151	0.027172182	0.016823852
0.024625791	0.025622809	0.030793831	-0.00517102	-0.19554972	0.032106200	0.019244574
0.025318965	0.021435567	0.026923600	-0.00548803	-0.23214541	0.030334216	0.014367370
0.024952042	0.024733937	0.030504451	-0.00577051	-0.22173449	0.031804188	0.018227038
0.023320366	0.021687535	0.027828576	-0.00614104	-0.25556102	0.027513338	0.018275248
0.038749421	0.025193116	0.031360601	-0.00616748	-0.232963871	0.033705024	0.020940725
0.048064997	0.037738458	0.044305005	-0.00656654	-0.18593319	0.044941548	0.029605287
0.032164952	0.022185790	0.028882948	-0.00669715	-0.27271372	0.025288430	0.018253191
0.048036164	0.030980676	0.037749774	-0.00676909	-0.22055014	0.038313797	0.024655584
0.036171827	0.024263898	0.031458211	-0.00719431	-0.27496860	0.036048486	0.018901803
0.041815375	0.028490629	0.035945680	-0.007455051	-0.25542197	0.041527825	0.020897375
0.036859278	0.029759387	0.037480105	-0.00772071	-0.25602760	0.032311717	0.026506560
0.034705072	0.032264631	0.040588613	-0.008323981	-0.25936181	0.039077059	0.024426143
0.048033727	0.034773988	0.043172194	-0.00839820	-0.24801116	0.046904734	0.022082291
0.031169471	0.027205600	0.035772957	-0.00856735	-0.298975701	0.031823348	0.021477580
0.046682993	0.040795005	0.050027093	-0.00923208	-0.240927161	0.047886752	0.034345668
0.054357884	0.036563977	0.045826106	-0.00926212	-0.26172564	0.047204789	0.024633963
0.062537336	0.042071591	0.051382105	-0.00931051	-0.23732162	0.052848165	0.033056443
0.047012763	0.029472070	0.038899753	-0.00942768	-0.308995011	0.040974484	0.024107696
0.036564268	0.026362050	0.035955722	-0.00959367	-0.33781097	0.032129696	0.021097640
0.056983417	0.037307589	0.047058789	-0.00975119	-0.27037748	0.043281261	0.027582861
0.059221291	0.046866328	0.056896640	-0.010030311	-0.23435909	0.054498851	0.037359158
0.059920474	0.052262544	0.062324196	-0.01006165	-0.216113861	0.059111402	0.043184300
0.056163438	0.045532810	0.055867695	-0.01033488	-0.24623067	0.052819374	0.040163661
0.043812995	0.036827296	0.047811490	-0.01098419	-0.30400651	0.049519360	0.026319439
0.077671793	0.062668021	0.073748359	-0.01108033	-0.20474030	0.074168461	0.055174369
0.087417557	0.064014563	0.075121460	-0.01110689	-0.20171372	0.073701581	0.055087265
0.042482636	0.033847177	0.045271077	-0.01142389	-0.33404075	0.039851622	0.026746577
0.056667152	0.039743213	0.051377176	-0.01163396	-0.30320256	0.053533004	0.028535794
0.084382970	0.072842810	0.084498738	-0.01165592	-0.18991858	0.086644724	0.063326458
0.047029292	0.037518595	0.050224522	-0.01270592	-0.34185885	0.050828929	0.025608876
0.057709082	0.044384344	0.057773041	-0.01338869	-0.31752012	0.082712582	0.026537115
0.929359283	0.911043965	0.924708220	-0.01366425	-0.02124605	0.933816348	0.881950281
0.062622610	0.050321063	0.064576303	-0.01425524	-0.30605542	0.060466724	0.040000226
0.929273588	0.900435710	0.916174789	-0.01573907	-0.02472734	0.926349456	0.876580530

0.130754421	0.059917722	0.075746914	-0.01582919	-0.29442656	0.071996956	0.049764854
0.929504218	0.904674099	0.920884794	-0.01621069	-0.02534483	0.933810578	0.892435149
0.920209702	0.886714815	0.903422266	-0.01670745	-0.02663272	0.909260607	0.871080187
0.071429207	0.062246085	0.079115546	-0.01686946	-0.30275770	0.082453475	0.050713772
0.039722327	0.034089082	0.050968867	-0.01687978	-0.46765130	0.054009175	0.020810074
0.105424986	0.078034398	0.095269209	-0.01723481	-0.25794425	0.089613314	0.061658678
0.111718307	0.064974391	0.082296725	-0.01732233	-0.29988154	0.083130668	0.050550761
0.943336926	0.910238230	0.927630210	-0.01739197	-0.02701166	0.935399197	0.875613162
0.093426485	0.070775702	0.089061367	-0.01828566	-0.29440114	0.083386174	0.032137618
0.927093231	0.890318029	0.908947699	-0.01862967	-0.02954804	0.919750985	0.868110155
0.081301591	0.068013571	0.087077045	-0.01906347	-0.31540507	0.081392619	0.053140238
0.068865972	0.052483260	0.071610023	-0.01912676	-0.38527663	0.068988232	0.041395606
0.923430588	0.880757048	0.900290471	-0.01953342	-0.03129498	0.914206137	0.853034462
0.170060522	0.128689180	0.148255454	-0.01956627	-0.19039998	0.152014371	0.117485519
0.928948621	0.890617489	0.910324703	-0.01970721	-0.03122846	0.906495777	0.870045367
0.924758985	0.886008322	0.905771787	-0.01976346	-0.03147598	0.906434466	0.855574981
0.884929926	0.857019583	0.877070803	-0.02005122	-0.03298468	0.873646410	0.835559688
0.088344226	0.065209630	0.085725207	-0.02051557	-0.34798147	0.083291261	0.043929448
0.908575089	0.872971764	0.893809315	-0.02083755	-0.03365112	0.899440945	0.856171295
0.913833925	0.858547373	0.881632815	-0.02308544	-0.03784517	0.878946143	0.820547489
0.898427350	0.858465818	0.881827346	-0.02336152	-0.03829537	0.883742296	0.822467076
0.168530540	0.151500137	0.174947488	-0.02344735	-0.19558031	0.194573228	0.125325128
0.928711235	0.884886049	0.908378365	-0.02349231	-0.03738466	0.918057250	0.860742694
0.863415647	0.801400055	0.825309088	-0.02390903	-0.04189673	0.835166757	0.761731170
0.919977002	0.859584688	0.883833328	-0.02424863	-0.03967930	0.897939582	0.819048199
0.190261716	0.128968570	0.153567457	-0.02459888	-0.23512711	0.149158561	0.106979147
0.116934345	0.082063088	0.107068345	-0.02500525	-0.34665629	0.120195861	0.046357875
0.153700340	0.119002043	0.144812332	-0.02581028	-0.26312648	0.159224947	0.084685600
0.066159503	0.049474379	0.077108718	-0.02763433	-0.55054880	0.068942301	0.033486000
0.076307641	0.048356512	0.076134160	-0.02777764	-0.56169184	0.068943331	0.029938492
0.903851199	0.867444833	0.896001042	-0.02855620	-0.04620428	0.919868115	0.816908900
0.867075375	0.814402684	0.843070684	-0.02866799	-0.04931608	0.858483832	0.772814643
0.893856751	0.855786519	0.885357412	-0.02957089	-0.04845235	0.888981262	0.824396535
0.870745725	0.828272995	0.858176282	-0.02990328	-0.05056785	0.856641008	0.789655413
0.193262342	0.145317114	0.175274429	-0.02995731	-0.25444697	0.202593119	0.095758563
0.855325787	0.838583248	0.869176617	-0.03059336	-0.05109681	0.866417233	0.791796398
0.184883327	0.141141376	0.172072353	-0.03093097	-0.26861320	0.208384635	0.084042415
0.883882644	0.816527339	0.847737917	-0.03121057	-0.05347435	0.845354254	0.771317583
0.861655857	0.833131408	0.864411934	-0.03128052	-0.05255558	0.879518480	0.780872201
0.424655633	0.375468413	0.407119503	-0.03165109	-0.11384812	0.414830197	0.337740987
0.159788896	0.125989502	0.157755626	-0.03176612	-0.30286586	0.141841504	0.096217527

0.891308719	0.828284058	0.860059877	-0.031775819	-0.053675497	0.862832786	0.796538954
0.803912064	0.761720954	0.793571126	-0.031850172	-0.058346448	0.804434982	0.693024476
0.890099782	0.819558714	0.851634690	-0.032075976	-0.054732240	0.856736204	0.766312602
0.890228171	0.824272686	0.857008870	-0.032736184	-0.055527741	0.873817217	0.796798646
0.841927217	0.807574492	0.841347450	-0.033772957	-0.058397855	0.836528606	0.750335983
0.824612958	0.795420518	0.829589417	-0.034168899	-0.059941757	0.828605746	0.748278699
0.762706611	0.707007308	0.741526211	-0.034518903	-0.067835598	0.734036070	0.665894984
0.239130865	0.196042382	0.230774014	-0.034731631	-0.224738565	0.226530677	0.161150247
0.426291717	0.327767295	0.362605158	-0.034837863	-0.141618003	0.355475346	0.295286014
0.650142733	0.600502200	0.636470575	-0.035968375	-0.082588212	0.649172609	0.572062282
0.831874461	0.797710986	0.834328860	-0.036617874	-0.063965866	0.841549204	0.736289393
0.643191575	0.560241715	0.597744519	-0.037502803	-0.091891395	0.601098735	0.495896441
0.717358364	0.672322298	0.710270372	-0.037948074	-0.078085197	0.713439857	0.641158982
0.493926190	0.395656237	0.433982516	-0.038326278	-0.130245192	0.440639671	0.362541450
0.718487046	0.624537370	0.664595503	-0.040058132	-0.088317568	0.707357646	0.558720929
0.485287527	0.404217130	0.444587316	-0.040370186	-0.134170213	0.436654471	0.365245378
0.098485768	0.068675229	0.109189179	-0.040513949	-0.599271873	0.110956978	0.044318973
0.835593152	0.778391337	0.819303892	-0.040912554	-0.072988938	0.824446470	0.705541756
0.322450001	0.252631391	0.293830876	-0.041199485	-0.210229122	0.314069061	0.214080766
0.455915400	0.417876921	0.459206254	-0.041329333	-0.133026381	0.467097474	0.342768552
0.911979747	0.826004576	0.867549225	-0.041544648	-0.069969214	0.858473965	0.790309117
0.615472777	0.532787890	0.574410955	-0.041623065	-0.106594690	0.592288408	0.495212858
0.361540978	0.309977046	0.351814919	-0.041837872	-0.177283481	0.335811537	0.294533699
0.587744761	0.521736258	0.564443866	-0.042707608	-0.111455077	0.566876462	0.486414315
0.871223185	0.821478591	0.864309594	-0.042831002	-0.072465112	0.870557819	0.785183161
0.366106650	0.335255492	0.378131851	-0.042876359	-0.168882464	0.393168761	0.280579297
0.386388789	0.314942344	0.358156141	-0.043213797	-0.180134012	0.345774399	0.293063046
0.796023708	0.685446197	0.729425702	-0.043979504	-0.088466285	0.750298275	0.619773962
0.775326713	0.668919336	0.713506921	-0.044587585	-0.091766643	0.717375039	0.613789030
0.822948589	0.769953249	0.815176854	-0.045223605	-0.081315705	0.831022022	0.709764868
0.801327621	0.737238331	0.782471900	-0.045233568	-0.084791316	0.784492670	0.690173396
0.491302031	0.409248651	0.455204179	-0.045955528	-0.150057915	0.491775795	0.358145433
0.831829159	0.734324620	0.780288585	-0.045963965	-0.086447613	0.805444070	0.687182916
0.254594298	0.181388360	0.227470152	-0.046081792	-0.311243102	0.241062689	0.129569450
0.682183652	0.589639709	0.635972671	-0.046332962	-0.107377206	0.662860846	0.539848708
0.783807217	0.734485006	0.781547370	-0.047062363	-0.088432897	0.770800045	0.699015182
0.497569312	0.423747192	0.471118410	-0.047371218	-0.149537588	0.507219889	0.358194380
0.539658232	0.438478660	0.486694269	-0.048215608	-0.147318760	0.500233280	0.371820906
0.809225367	0.726656965	0.775907514	-0.049250548	-0.093366580	0.785230474	0.683879713
0.783809467	0.745433999	0.795155749	-0.049721749	-0.091962169	0.815161654	0.646964121
0.514381192	0.473815055	0.523698067	-0.049883011	-0.141568122	0.552455084	0.411593326

0.632560261	0.578759655	0.629103121	-0.05034346	-0.11836991	0.674483593	0.529257196
0.526972788	0.459184355	0.509601945	-0.05041758	-0.14725192	0.507969031	0.414857177
0.138633177	0.084201386	0.134641521	-0.05044013	-0.61866155	0.149111290	0.046114886
0.147032735	0.096621229	0.147208767	-0.05058753	-0.56018695	0.151336228	0.063856584
0.571005453	0.438857287	0.489516968	-0.05065968	-0.15427686	0.478300915	0.364367324
0.583440644	0.519304878	0.570716304	-0.05141142	-0.13373459	0.583111489	0.484234553
0.188183548	0.167920582	0.219531790	-0.05161120	-0.36746056	0.202328973	0.122809505
0.784111505	0.764698443	0.818219479	-0.05352103	-0.09637829	0.822594584	0.731057063
0.364650638	0.349718216	0.403700916	-0.05398270	-0.20172093	0.405665700	0.296782676
0.881335987	0.780314342	0.834471092	-0.05415674	-0.09562144	0.856589465	0.720330825
0.626176988	0.541545562	0.595808696	-0.05426313	-0.13538221	0.600788737	0.482562013
0.802392162	0.730370889	0.785713317	-0.05534242	-0.10400057	0.796005660	0.681868711
0.729216995	0.620089801	0.676644725	-0.05655492	-0.12400637	0.683886549	0.529210321
0.680165438	0.571723572	0.628321531	-0.05659795	-0.13394954	0.635955515	0.439413035
0.364486168	0.315509739	0.372329656	-0.05681991	-0.23211640	0.356354924	0.268608594
0.837535864	0.759748260	0.817331714	-0.05758345	-0.10407918	0.842235585	0.707235956
0.727157616	0.673330721	0.731583215	-0.05825249	-0.11802460	0.795684262	0.568261354
0.822935962	0.859333741	0.917856879	-0.05852313	-0.09399214	0.930200365	0.694615925
0.586419355	0.500848420	0.559824198	-0.05897577	-0.15762161	0.529920847	0.463730553
0.752489654	0.668493793	0.728003922	-0.05951012	-0.12129286	0.705649963	0.619246823
0.746065076	0.642788926	0.703785969	-0.06099704	-0.12887495	0.724233904	0.524682174
0.642725546	0.527844477	0.590678710	-0.06283423	-0.15940446	0.636829551	0.442007598
0.328222849	0.280560376	0.346082665	-0.06552228	-0.29337422	0.328902425	0.217510540
0.686905343	0.610586321	0.679679425	-0.06909310	-0.15229403	0.673855493	0.552858509
0.785606029	0.755518428	0.824973234	-0.06945480	-0.12529284	0.821971995	0.705489162
0.818524647	0.742834737	0.815598741	-0.07276400	-0.13310757	0.813133682	0.582105090
0.500822250	0.387000541	0.460047561	-0.07304702	-0.24366576	0.479438150	0.307861990
0.557943603	0.444895797	0.519798929	-0.07490313	-0.21990882	0.554195709	0.372708115
0.589069140	0.511796092	0.591207300	-0.07941120	-0.20437638	0.571732309	0.446593738
0.773598321	0.640657314	0.723223555	-0.08256624	-0.17235522	0.720727591	0.474393541
0.528204450	0.403751793	0.491542158	-0.08779036	-0.27760540	0.541306875	0.239843392
0.361771366	0.342674924	0.441140702	-0.09846577	-0.35523845	0.423247068	0.263324512
0.976619571	0.974833979	0.969154684	0.005679294	0.008343748	0.982017122	0.963121517
0.952359663	0.951790431	0.942588806	0.009201625	0.013868977	0.964630721	0.941991311
0.031174117	0.040330069	0.031016859	0.009313210	0.295203558	0.055113802	0.025969677
0.960403354	0.963355146	0.953664602	0.009690543	0.014435181	0.969437737	0.955680246
0.928077165	0.931389669	0.898879003	0.032510666	0.050703776	0.959121648	0.889433977
0.806796559	0.837599375	0.783977090	0.053622285	0.094285144	0.926851808	0.795467768
0.718206695	0.755712358	0.671825031	0.083887327	0.167400977	0.852797530	0.660064219

sd.CONT	max.DA	min.DA	sd.DA	diffmeth.p.val (Welch's t-test)
0.002937134	0.032721204	0.023170242	0.003074015	0.015313100603716
0.002723130	0.029959423	0.019109808	0.003531077	0.0112295676436983
0.002906707	0.033002751	0.018229861	0.004586208	0.0276821059157054
0.002889343	0.030975450	0.017446755	0.004188510	0.0118247802159619
0.001823872	0.028910550	0.017457640	0.003984612	0.00348197718459
0.003098827	0.040887822	0.022930363	0.005266027	0.0212999097133083
0.004174229	0.033671078	0.024625791	0.002847848	0.0117649448000604
0.005554129	0.033891294	0.024098296	0.003697278	0.0143675609558583
0.004378147	0.043050791	0.024055207	0.006070443	0.0271160176986566
0.002847324	0.032979304	0.019945674	0.004262718	0.00313073373276736
0.003835419	0.038749421	0.019817599	0.005215426	0.0144416842966274
0.004820338	0.051963768	0.034571817	0.006039806	0.0329913480165571
0.002717449	0.035493348	0.017752445	0.005145137	0.00486504552906888
0.004698027	0.048036164	0.028014984	0.006062551	0.0184330340138221
0.005409267	0.041069724	0.019225630	0.007009369	0.0187474143578305
0.006393048	0.048297395	0.021633155	0.008704556	0.0447446541694946
0.001762386	0.047110208	0.027177409	0.007113096	0.00783775794044591
0.004881668	0.057478067	0.029462820	0.008447600	0.0168112423586213
0.008234692	0.056900596	0.026408499	0.008755472	0.0433809041362177
0.003277186	0.046306502	0.026404120	0.006249213	0.00198007364991104
0.005140444	0.059831109	0.042425462	0.005999809	0.00506114617254402
0.007399965	0.066378611	0.029156262	0.011184608	0.0459015454530176
0.006465264	0.066988077	0.038633512	0.008310208	0.0164538830600373
0.005774618	0.047051796	0.025244717	0.007531635	0.00732094155290743
0.004238021	0.046258418	0.022413197	0.007228665	0.0029065750127275
0.005629302	0.062692046	0.032116898	0.009055578	0.0148700021584226
0.005862903	0.072618862	0.043319406	0.011036014	0.0317818655685939
0.006002234	0.075951599	0.050438723	0.007914039	0.0140260082810358
0.003969459	0.075635716	0.046658535	0.008071997	0.0047212137837253
0.006650749	0.065881363	0.031911774	0.010172383	0.0117001233660369
0.006046559	0.089711770	0.054144080	0.010760466	0.0285192915704629
0.006332267	0.087417557	0.061636073	0.008450224	0.0152280831927608
0.004194382	0.063803524	0.035463911	0.008083424	0.00106323276640853
0.008428150	0.066520708	0.029892020	0.011674665	0.0241764809837784
0.008298351	0.094764274	0.069179227	0.008217328	0.0198643503321247
0.006953317	0.073268427	0.034541929	0.013294877	0.0123846624741132
0.016248424	0.097372119	0.040530713	0.016960392	0.0422106730464168
0.016055669	0.937921310	0.911693769	0.009412728	0.0448449936186108
0.006445932	0.082341081	0.047513717	0.012844042	0.00767879359319268
0.017799512	0.934281532	0.890721532	0.012725459	0.0480339832327335

0.007349279	0.130754421	0.052556099	0.023470528	0.0464482700200989
0.013034267	0.941887389	0.878913348	0.017804891	0.0314484094430771
0.010845712	0.932671135	0.873511892	0.020351239	0.0397590441951658
0.010741747	0.122936141	0.057587577	0.019697050	0.021724602887619
0.011946876	0.079227551	0.020274041	0.018506052	0.0251662704229684
0.008205272	0.114155662	0.079962078	0.011121290	0.00407490154469958
0.011756738	0.111718307	0.053902666	0.016201353	0.0159669136380321
0.020326953	0.945931784	0.893402246	0.016268759	0.0392415532947315
0.015850092	0.114789373	0.070044440	0.014754470	0.0254624775932606
0.017948509	0.927093231	0.877824735	0.014694808	0.0282445804667189
0.010638358	0.140312114	0.062849099	0.022375387	0.0203636774975198
0.009843623	0.094662207	0.054458471	0.013680090	0.0017725775108131
0.020447127	0.923430588	0.872071117	0.015828225	0.0371619310970204
0.010400076	0.171963438	0.129375207	0.014715920	0.0148047113137349
0.013626783	0.929491451	0.885747576	0.013941473	0.00874117854851126
0.017632423	0.926835598	0.872620472	0.016798094	0.0219436954336862
0.011458592	0.901006645	0.855055577	0.014277057	0.0122215637113872
0.010456419	0.098854518	0.068579869	0.010324650	0.000977327810837946
0.015329261	0.928026832	0.841167255	0.025828255	0.0362921683102653
0.017286550	0.914085254	0.850824995	0.021981007	0.0230630190259586
0.019801071	0.904431827	0.838552586	0.019716868	0.0187130261224002
0.023550017	0.215552633	0.149560021	0.022053378	0.0409050096400192
0.016065333	0.930379214	0.877257778	0.016545992	0.00728229148787424
0.023804795	0.863415647	0.805077673	0.017487357	0.041366043959073
0.025515341	0.919977002	0.849587061	0.021055202	0.0391898879003681
0.016791283	0.190261716	0.125293695	0.025021827	0.0257385872598453
0.022746440	0.155448940	0.073843271	0.025822123	0.0290035674222525
0.025161273	0.167661753	0.107992368	0.019710858	0.0205343619910105
0.010803645	0.133530837	0.042289792	0.031546619	0.0103143221611778
0.012229537	0.169255658	0.038857315	0.037724152	0.0173786553550802
0.036483605	0.925000176	0.868429395	0.019565268	0.0460520472398753
0.028581833	0.871207339	0.798819358	0.023083745	0.0293755342731696
0.020842541	0.936674395	0.828815413	0.034029986	0.0259426280901095
0.023822071	0.909636607	0.817024306	0.032592544	0.0323733723549653
0.032574691	0.204776123	0.116673298	0.029894407	0.0469168702341546
0.024574012	0.928440370	0.817127502	0.037400549	0.0360085478937931
0.032388050	0.229314377	0.132981958	0.033006884	0.0408599828457688
0.025202272	0.883882644	0.808970510	0.025422141	0.015954770350122
0.028465850	0.917379155	0.837943804	0.023929398	0.0217925034278365
0.021446735	0.455787662	0.365873672	0.028413273	0.044290074208245
0.014806248	0.194076836	0.119881482	0.024650738	0.00455716949207176

0.023992651	0.910095378	0.826938795	0.032791582	0.0261537464308866
0.030759078	0.838378192	0.756131273	0.028741166	0.0360783722859076
0.027109488	0.890099782	0.827456541	0.020234891	0.0110524982496344
0.022446650	0.890228171	0.822047402	0.024252444	0.00965412804894876
0.027520699	0.904227994	0.776274031	0.043887248	0.0386175039466262
0.026657130	0.870392051	0.796110931	0.025106922	0.0130740462764813
0.020280571	0.790136515	0.705675072	0.028265607	0.0175826749699646
0.022826456	0.292449219	0.187899066	0.028080144	0.0128536002552634
0.023325478	0.426291717	0.312563203	0.038391114	0.0470975146142256
0.027665779	0.686041801	0.559468031	0.038576694	0.0487364234046789
0.034741326	0.905362985	0.799155758	0.027930934	0.0241598883783852
0.041000572	0.643191575	0.547678847	0.029014433	0.048882099252789
0.022829655	0.783597001	0.655616852	0.038567156	0.0288454814339084
0.027396725	0.495442976	0.377291957	0.041288296	0.0454999660806468
0.044493910	0.718487046	0.623106057	0.032312419	0.0472587989731534
0.022417146	0.498151873	0.400401041	0.035513276	0.0224111389106
0.022081996	0.231676781	0.063736016	0.047525804	0.00639560439979331
0.036798044	0.889129250	0.743263164	0.044797576	0.0300215194764539
0.030353335	0.322450001	0.263403480	0.017996042	0.0051383069663515
0.046574460	0.496762771	0.417547790	0.026602514	0.0382390525639072
0.024855305	0.914403600	0.794715384	0.035108579	0.0057412785369628
0.034524314	0.615472777	0.526901897	0.027541353	0.0214699957903696
0.011348860	0.405264851	0.313131200	0.030080262	0.00564533533043482
0.026524824	0.594816263	0.526693265	0.026002868	0.0110672525512063
0.027852096	0.910662296	0.774810111	0.039316475	0.00781105510948452
0.034040175	0.465170812	0.315960197	0.048905948	0.0452832967187147
0.017455668	0.407834084	0.325622042	0.027114642	0.00446518505303296
0.044855057	0.796023708	0.657247947	0.047468997	0.04736969037645
0.039205560	0.778986907	0.624695562	0.054078820	0.0460314217871448
0.041385171	0.870986405	0.781980236	0.028109225	0.01145240494447
0.031798372	0.816965566	0.722724215	0.027394094	0.00502770283089389
0.042383983	0.502460916	0.368995210	0.044820278	0.0393418135237928
0.033832492	0.837484951	0.734341687	0.036201393	0.012575044389519
0.038865253	0.281519511	0.166305598	0.033015543	0.0100605182937431
0.041131112	0.682406096	0.605083832	0.029530189	0.0177978013556044
0.020923501	0.857112706	0.728902514	0.039072103	0.00675972519338566
0.052473120	0.547546988	0.400965708	0.044610790	0.047414730364128
0.036632301	0.572052508	0.443888944	0.040469703	0.020347649835562
0.038828828	0.845318596	0.731447105	0.035796316	0.0112813043782678
0.049920116	0.873355763	0.717887395	0.043148561	0.0240501891326704
0.046514049	0.616250451	0.425278131	0.055820516	0.0484626765952426

0.049059921	0.678975996	0.543305906	0.039067235	0.0260237454597098
0.030445917	0.572105683	0.420143331	0.051969080	0.0246695562156559
0.028899967	0.339488338	0.068704549	0.076549277	0.0230913315491318
0.028815855	0.277860956	0.072935979	0.066063833	0.0298936372683832
0.035379707	0.571005453	0.403862974	0.057970401	0.0371426782588395
0.034354535	0.613175264	0.522404118	0.031327929	0.00730440546707238
0.024569620	0.308074416	0.175105830	0.043388666	0.00330832710830921
0.029105970	0.876879458	0.753224464	0.040603945	0.00388042624768213
0.036913796	0.440254190	0.358840458	0.029786865	0.00469728191952805
0.041296579	0.887331466	0.788595240	0.034689503	0.00532885390521587
0.046659575	0.679896856	0.529442474	0.055210591	0.0313527493412049
0.039624560	0.841485020	0.755583564	0.032226000	0.00396731733406187
0.049153428	0.750550460	0.601969317	0.051329732	0.0209594575657255
0.065421497	0.680165438	0.578421287	0.034517836	0.024958806881042
0.031021150	0.463139307	0.284185498	0.057162805	0.0157577805864692
0.044890839	0.905588206	0.703792223	0.067286438	0.0228281908308987
0.060927560	0.800147901	0.595846781	0.064075248	0.0423621914682373
0.078058669	0.951883705	0.822935962	0.042387641	0.0140860471374726
0.020250318	0.627314990	0.500336854	0.045966013	0.00429556436140157
0.031619646	0.820975182	0.622110247	0.057796965	0.0106943891488435
0.053275848	0.793887908	0.590907424	0.065912930	0.0272732589138
0.059566296	0.642725546	0.547157259	0.038152472	0.012689704542032
0.041966708	0.393067345	0.201463297	0.059840332	0.0156719075842699
0.043005757	0.766890057	0.575915990	0.066132940	0.0120845299570347
0.040077017	0.904914052	0.719962907	0.068935751	0.00865643141317885
0.069645608	0.865180409	0.764498188	0.037627032	0.00392656087478859
0.056714564	0.533285384	0.335077280	0.069031556	0.017316106648626
0.064884381	0.603651477	0.444647435	0.052880816	0.0097324672797686
0.043829475	0.736163314	0.475987424	0.077910171	0.0111961284337485
0.076252589	0.773598321	0.661948267	0.038012533	0.0036234438823891
0.089910231	0.591892830	0.411532703	0.054596799	0.0124358456389534
0.050477491	0.580857279	0.340273220	0.084192120	0.00380934850334658
0.005761969	0.976914319	0.961402938	0.005634012	0.0363986815013882
0.007246989	0.952867232	0.925326366	0.008913533	0.0321872553941352
0.010444560	0.038549070	0.024513568	0.004863132	0.0197510957213361
0.005452763	0.967209163	0.934626155	0.012026001	0.0367573225255921
0.022953741	0.947810996	0.819037221	0.045456222	0.0355720903326989
0.043471456	0.869662103	0.677469258	0.063374713	0.036816181738972
0.068300036	0.808842161	0.535554458	0.089631191	0.0224029205588696

KEGG pathway	p-value
Glycosaminoglycan biosynthesis - chondroitin sulfate	0.00000000000000
ErbB signaling pathway	0.0000000000000781
mTOR signaling pathway	0.00000006746155
Insulin signaling pathway	0.0000000779425
TGF-beta signaling pathway	0.00000268675
Acute myeloid leukemia	0.00001637666
Dorso-ventral axis formation	0.00003311146
Dopaminergic synapse	0.00004679336
Neurotrophin signaling pathway	0.00005811483
Gap junction	0.00007844055
Wnt signaling pathway	0.0001367145
Transcriptional misregulation in cancer	0.0003077745
Endocrine and other factor-regulated calcium reabsorption	0.0003916736
Glioma	0.0005503835
PI3K-Akt signaling pathway	0.0007617474
Prostate cancer	0.0007926991
Focal adhesion	0.0008046906
MAPK signaling pathway	0.001499682
Colorectal cancer	0.00183516
Long-term potentiation	0.002195478
Long-term depression	0.002274003
Endometrial cancer	0.002487843
Adherens junction	0.00264312
Fc gamma R-mediated phagocytosis	0.0027016
Chronic myeloid leukemia	0.003697233
Mucin type O-Glycan biosynthesis	0.005987828
GnRH signaling pathway	0.007581879
Pathways in cancer	0.01260286
Hepatitis B	0.01410429
Glutamatergic synapse	0.01583876
Regulation of actin cytoskeleton	0.01789224
Non-small cell lung cancer	0.01850767
Axon guidance	0.03099447
Pancreatic cancer	0.03459055
Serotonergic synapse	0.03734403
Notch signaling pathway	0.03924985
HTLV-I infection	0.04108313

**ErbB signaling pathway (hsa04012)**	**p value**
hsa-miR-34b-3p|microT-CDS	0.00000079
hsa-miR-34c-3p|microT-CDS	0.00002045
hsa-miR-144-3p|microT-CDS	0.00229325
hsa-miR-34b-5p|microT-CDS	0.00308727
hsa-miR-34a-5p|microT-CDS	0.00808990
hsa-miR-146b-3p|microT-CDS	0.01554508
hsa-miR-146b-5p|microT-CDS	0.01784331
hsa-miR-181d|microT-CDS	0.02009958
hsa-miR-451a|microT-CDS	0.02149957
hsa-miR-34c-5p|microT-CDS	0.02552946
hsa-miR-181c-5p|microT-CDS	0.02763107

#genes	#miRNAs
4	5
33	11
23	8
31	7
25	5
14	4
10	7
36	5
33	7
24	4
36	5
20	3
10	4
15	4
61	5
17	5
45	4
35	2
20	4
20	7
18	3
17	6
15	4
14	3
17	4
3	2
14	2
40	2
30	4
16	2
46	5
14	3
26	3
19	4
22	3
9	4
21	1

# target genes	Gene Name	Gene Ensembl id
7	CBLB;SOS1;G	ENSG00000114423;ENSG00000115904;ENSG0000010
6	GSK3B;PAK2;	ENSG00000082701;ENSG00000180370;ENSG0000011
9	GSK3B;PIK3C	ENSG00000082701;ENSG00000051382;ENSG00000101
5	MYC;NRG3;S	ENSG00000136997;ENSG00000185737;ENSG00000197
6	ERBB2;AREG	ENSG00000141736;ENSG00000205595;ENSG00000051
5	BRAF;PAK7;R	ENSG00000157764;ENSG00000101349;ENSG00000132
4	CAMK2D;NR	ENSG00000145349;ENSG00000213281;ENSG00000143
5	CAMK2D;CRK	ENSG00000145349;ENSG00000167193;ENSG00000117
1	EREG	ENSG00000124882
3	ERBB2;AREG	ENSG00000141736;ENSG00000205595;ENSG00000169
5	CRK;PIK3R3;	ENSG00000167193;ENSG00000117461;ENSG00000117

9458;ENSG00000065559;ENSG00000177885;ENSG00000178568;ENSG00000108443
4423;ENSG00000115904;ENSG00000109458;ENSG00000177885
1349;ENSG00000146648;ENSG00000107643;ENSG00000109458;ENSG00000117020;ENSG00000[illegible]
7122;ENSG00000178568;ENSG00000109339
1382;ENSG00000109321;ENSG00000166501;ENSG00000169032
2155;ENSG00000175634;ENSG00000166501
3322;ENSG00000178568
7461;ENSG00000115904;ENSG00000108443

9032
7020;ENSG00000169032;ENSG00000108443

198793;ENSG00000178568
